# Global burden of bacterial antimicrobial resistance in 2019: a systematic analysis

**DOI:** 10.1016/S0140-6736(21)02724-0

**Published:** 2022-02-12

**Authors:** Antimicrobial Resistance Collaborators, Christopher JL Murray, Kevin Shunji Ikuta, Fablina Sharara, Lucien Swetschinski, Gisela Robles Aguilar, Authia Gray, Chieh Han, Catherine Bisignano, Puja Rao, Eve Wool, Sarah C Johnson, Annie J Browne, Michael Give Chipeta, Frederick Fell, Sean Hackett, Georgina Haines-Woodhouse, Bahar H Kashef Hamadani, Emmanuelle A P Kumaran, Barney McManigal, Ramesh Agarwal, Samuel Akech, Samuel Albertson, John Amuasi, Jason Andrews, Aleskandr Aravkin, Elizabeth Ashley, Freddie Bailey, Stephen Baker, Buddha Basnyat, Adrie Bekker, Rose Bender, Adhisivam Bethou, Julia Bielicki, Suppawat Boonkasidecha, James Bukosia, Cristina Carvalheiro, Carlos Castañeda-Orjuela, Vilada Chansamouth, Suman Chaurasia, Sara Chiurchiù, Fazle Chowdhury, Aislinn J Cook, Ben Cooper, Tim R Cressey, Elia Criollo-Mora, Matthew Cunningham, Saffiatou Darboe, Nicholas P J Day, Maia De Luca, Klara Dokova, Angela Dramowski, Susanna J Dunachie, Tim Eckmanns, Daniel Eibach, Amir Emami, Nicholas Feasey, Natasha Fisher-Pearson, Karen Forrest, Denise Garrett, Petra Gastmeier, Ababi Zergaw Giref, Rachel Claire Greer, Vikas Gupta, Sebastian Haller, Andrea Haselbeck, Simon I Hay, Marianne Holm, Susan Hopkins, Kenneth C Iregbu, Jan Jacobs, Daniel Jarovsky, Fatemeh Javanmardi, Meera Khorana, Niranjan Kissoon, Elsa Kobeissi, Tomislav Kostyanev, Fiorella Krapp, Ralf Krumkamp, Ajay Kumar, Hmwe Hmwe Kyu, Cherry Lim, Direk Limmathurotsakul, Michael James Loftus, Miles Lunn, Jianing Ma, Neema Mturi, Tatiana Munera-Huertas, Patrick Musicha, Marisa Marcia Mussi-Pinhata, Tomoka Nakamura, Ruchi Nanavati, Sushma Nangia, Paul Newton, Chanpheaktra Ngoun, Amanda Novotney, Davis Nwakanma, Christina W Obiero, Antonio Olivas-Martinez, Piero Olliaro, Ednah Ooko, Edgar Ortiz-Brizuela, Anton Yariv Peleg, Carlo Perrone, Nishad Plakkal, Alfredo Ponce-de-Leon, Mathieu Raad, Tanusha Ramdin, Amy Riddell, Tamalee Roberts, Julie Victoria Robotham, Anna Roca, Kristina E Rudd, Neal Russell, Jesse Schnall, John Anthony Gerard Scott, Madhusudhan Shivamallappa, Jose Sifuentes-Osornio, Nicolas Steenkeste, Andrew James Stewardson, Temenuga Stoeva, Nidanuch Tasak, Areerat Thaiprakong, Guy Thwaites, Claudia Turner, Paul Turner, H Rogier van Doorn, Sithembiso Velaphi, Avina Vongpradith, Huong Vu, Timothy Walsh, Seymour Waner, Tri Wangrangsimakul, Teresa Wozniak, Peng Zheng, Benn Sartorius, Alan D Lopez, Andy Stergachis, Catrin Moore, Christiane Dolecek, Mohsen Naghavi

## Abstract

**Background:**

Antimicrobial resistance (AMR) poses a major threat to human health around the world. Previous publications have estimated the effect of AMR on incidence, deaths, hospital length of stay, and health-care costs for specific pathogen–drug combinations in select locations. To our knowledge, this study presents the most comprehensive estimates of AMR burden to date.

**Methods:**

We estimated deaths and disability-adjusted life-years (DALYs) attributable to and associated with bacterial AMR for 23 pathogens and 88 pathogen–drug combinations in 204 countries and territories in 2019. We obtained data from systematic literature reviews, hospital systems, surveillance systems, and other sources, covering 471 million individual records or isolates and 7585 study-location-years. We used predictive statistical modelling to produce estimates of AMR burden for all locations, including for locations with no data. Our approach can be divided into five broad components: number of deaths where infection played a role, proportion of infectious deaths attributable to a given infectious syndrome, proportion of infectious syndrome deaths attributable to a given pathogen, the percentage of a given pathogen resistant to an antibiotic of interest, and the excess risk of death or duration of an infection associated with this resistance. Using these components, we estimated disease burden based on two counterfactuals: deaths attributable to AMR (based on an alternative scenario in which all drug-resistant infections were replaced by drug-susceptible infections), and deaths associated with AMR (based on an alternative scenario in which all drug-resistant infections were replaced by no infection). We generated 95% uncertainty intervals (UIs) for final estimates as the 25th and 975th ordered values across 1000 posterior draws, and models were cross-validated for out-of-sample predictive validity. We present final estimates aggregated to the global and regional level.

**Findings:**

On the basis of our predictive statistical models, there were an estimated 4·95 million (3·62–6·57) deaths associated with bacterial AMR in 2019, including 1·27 million (95% UI 0·911–1·71) deaths attributable to bacterial AMR. At the regional level, we estimated the all-age death rate attributable to resistance to be highest in western sub-Saharan Africa, at 27·3 deaths per 100 000 (20·9–35·3), and lowest in Australasia, at 6·5 deaths (4·3–9·4) per 100 000. Lower respiratory infections accounted for more than 1·5 million deaths associated with resistance in 2019, making it the most burdensome infectious syndrome. The six leading pathogens for deaths associated with resistance (*Escherichia coli*, followed by *Staphylococcus aureus, Klebsiella pneumoniae, Streptococcus pneumoniae, Acinetobacter baumannii*, and *Pseudomonas aeruginosa*) were responsible for 929 000 (660 000–1 270 000) deaths attributable to AMR and 3·57 million (2·62–4·78) deaths associated with AMR in 2019. One pathogen–drug combination, meticillin-resistant *S aureus*, caused more than 100 000 deaths attributable to AMR in 2019, while six more each caused 50 000–100 000 deaths: multidrug-resistant excluding extensively drug-resistant tuberculosis, third-generation cephalosporin-resistant *E coli, carbapenem-resistant* A baumannii, *fluoroquinolone-resistant E coli*, carbapenem-resistant *K pneumoniae*, and third-generation cephalosporin-resistant *K pneumoniae*.

**Interpretation:**

To our knowledge, this study provides the first comprehensive assessment of the global burden of AMR, as well as an evaluation of the availability of data. AMR is a leading cause of death around the world, with the highest burdens in low-resource settings. Understanding the burden of AMR and the leading pathogen–drug combinations contributing to it is crucial to making informed and location-specific policy decisions, particularly about infection prevention and control programmes, access to essential antibiotics, and research and development of new vaccines and antibiotics. There are serious data gaps in many low-income settings, emphasising the need to expand microbiology laboratory capacity and data collection systems to improve our understanding of this important human health threat.

**Funding:**

Bill & Melinda Gates Foundation, Wellcome Trust, and Department of Health and Social Care using UK aid funding managed by the Fleming Fund.

**Research in context**

**Evidence before this study**

To identify previous estimates of antimicrobial resistance (AMR) burden before this study, we did a systematic review and consulted with content experts. We searched the evidence available in PubMed for published works that evaluate exposure to antimicrobial resistant organisms (bacteria only) and evaluated all human-focused publications with more than ten cases, all genders, and all age groups. From these findings, we extracted study type, pathogen–drug combinations, counterfactuals, locations, methods, outcomes, and population. Extensive literature exists estimating incidence, deaths, hospital length of stay, and health-care costs associated with AMR from a small number of drug-resistant infections in select locations. There is widespread agreement that AMR poses a serious potential threat to human health around the world. The Review on Antimicrobial Resistance, published in 2016, estimated that as many as 10 million people could die annually from AMR by 2050. Recent estimates of the burden of drug-resistant infections covering several pathogens have also been published for the USA, Thailand, the EU and European Economic Area, and several other locations, as well as estimates for several pathogen–drug combinations for a wider range of locations. To our knowledge, however, there have been no comprehensive estimates covering all locations and a broad range of pathogens and pathogen–drug combinations.

**Added value of this study**

This study is the most comprehensive analysis of the burden of AMR to date, producing estimates for 204 countries and territories, 23 bacterial pathogens, and 88 pathogen–drug combinations, in 2019. This study uses major methodological innovations to provide important new insights into the AMR burden. Additionally, since this analysis builds on estimates of disease incidence, prevalence, and mortality from the Global Burden of Diseases, Injuries, and Risk Factors Study 2019, our findings on the burden of bacterial AMR can be compared with other causes of death, offering crucial context on the magnitude of the burden of this important health issue. Improvements to the input data and models compared with previous publications make our AMR estimates the most robust of any to date. Finally, this study is the first to quantify the burden of AMR using two different AMR counterfactual scenarios.

**Implications of all the available evidence**

Our estimates indicate that bacterial AMR is a health problem whose magnitude is at least as large as major diseases such as HIV and malaria, and potentially much larger. Bacterial AMR is a problem in all regions; we estimated that, in 2019, the highest rates of AMR burden were in sub-Saharan Africa. Six pathogens accounted for 73·4% (95% uncertainty interval 66·9–78·8) of deaths attributable to bacterial AMR. Seven pathogen–drug combinations each caused more than 50 000 deaths, highlighting the importance of developing policies that specifically target the deadliest pathogen–drug combinations, particularly through expansion of infection prevention and control programmes, improving access to essential second-line antibiotics where needed, and through vaccine and antibiotic development. Additionally, our comprehensive data collection effort shows that high-quality data on infectious disease, pathogens, and AMR are only sparsely available in many low-income settings. Both preventing bacterial AMR and increasing microbiological laboratory and data collection capacity to improve scientific understanding of this health threat should be a very high priority for global health policy makers.

## Introduction

Bacterial antimicrobial resistance (AMR)—which occurs when changes in bacteria cause the drugs used to treat infections to become less effective—has emerged as one of the leading public health threats of the 21st century. The Review on Antimicrobial Resistance, commissioned by the UK Government, argued that AMR could kill 10 million people per year by 2050.^1^, ^2^ Although these forecasts have been criticised by some,^3^, ^4^ WHO and numerous other groups and researchers agree that the spread of AMR is an urgent issue requiring a global, coordinated action plan to address.^5^, ^6^, ^7^, ^8^ Information about the current magnitude of the burden of bacterial AMR, trends in different parts of the world, and the leading pathogen–drug combinations contributing to bacterial AMR burden is crucial. If left unchecked, the spread of AMR could make many bacterial pathogens much more lethal in the future than they are today.

One major challenge to tackling AMR is understanding the true burden of resistance, particularly in locations where surveillance is minimal and data are sparse. Extensive literature exists estimating the effects of AMR on incidence, deaths, hospital length of stay, and health-care costs for select pathogen–drug combinations in specific locations,^1^, ^2^, ^6^, ^9^, ^10^, ^11^, ^12^ but, to our knowledge, no comprehensive estimates covering all locations and a broad range of pathogens and pathogen–drug combinations have ever been published. For instance, the US Centers for Disease Control and Prevention (CDC) published a 2019 report on AMR infections and deaths in the USA for 18 AMR threats using surveillance data,^6^ while Cassini and colleagues^10^ estimated the burden of eight bacterial pathogens and 16 pathogen–drug combinations in the EU and European Economic Area for 2007–15. Likewise, Lim and colleagues estimated the burden of multidrug resistance in six bacterial pathogens in Thailand in 2010,^11^ and Temkin and colleagues estimated the incidence of *Escherichia coli* and *Klebsiella pneumoniae* resistant to third-generation cephalosporins and carbapenems in 193 countries in 2014.^12^

Although these publications are important contributions to the body of work on AMR, they are insufficient to understand the global burden of AMR and identify and target the highest priority pathogens in different locations. Additionally, existing studies have generally considered only one measure of AMR burden.^13^ Because we do not know the extent to which drug-resistant infections would be replaced by susceptible infections or by no infection in a scenario in which all drug resistance was eliminated, it is important to quantify the burden on the basis of both these counterfactual scenarios.

In this study, we present the first global estimates of the burden of bacterial AMR covering an extensive set of pathogens and pathogen–drug combinations using consistent methods for both counterfactual scenarios.

## Methods

### Overview

We developed an approach for estimating the burden of AMR that makes use of all available data and builds on death and incidence estimates for different underlying conditions from the Global Burden of Diseases, Injuries, and Risk Factors Study (GBD) 2019, which provides age-specific and sex-specific estimates of disease burden for 369 diseases and injuries in 204 countries and territories in 1990–2019.^14^ Our approach can be divided into ten estimation steps that occur within five broad modelling components (a flowchart of the estimation steps is given in the appendix p 123). First, we obtained data from multiple data sources, including from published studies (eg, microbiology data, inpatient data, data on multiple causes of death, and pharmaceutical sales data) and directly from collaborators on the Global Research on Antimicrobial Resistance project,^15^ members of the GBD Collaborator Network, and other data providers.

We estimated the disease burdens associated with and attributable to AMR for 12 major infectious syndromes (lower respiratory infections and all related infections in the thorax; bloodstream infections; peritoneal and intra-abdominal infections; meningitis and other bacterial CNS infections; typhoid, paratyphoid, and invasive non-typhoidal *Salmonella* spp; urinary tract infections and pyelonephritis; diarrhoea; tuberculosis [not including tuberculosis associated with HIV]; bacterial infections of the skin and subcutaneous systems; endocarditis and other cardiac infections; infections of bones, joints, and related organs; and gonorrhoea and chlamydia) and one residual category, 23 bacterial pathogens, 18 drug categories or combinations of drugs for which there is resistance, and 88 pathogen–drug combinations (appendix pp 45–46). We modelled all-age and age-specific deaths and disability-adjusted life-years (DALYs) for 204 countries and territories, and we present aggregated estimates for 21 GBD regions, seven GBD super-regions, and globally in 2019 (a full list of GBD locations by region is available in the appendix pp 100–05).^16^

For the first counterfactual scenario—where all drug-resistant infections are replaced by susceptible infections—we estimated only deaths and DALYs directly attributable to resistance. For the second counterfactual scenario—where all drug-resistant infections are replaced by no infection—we estimated all deaths and DALYs associated with resistant infection. Estimates of AMR burden based on each counterfactual are useful in different ways for informing the development of potential intervention strategies to control AMR.^13^, ^17^, ^18^

### Input data

We used several data collection strategies. Through our large collaborator networks, we obtained datasets not previously available for AMR research, including hospital and laboratory data, as well as datasets published previously and those outlined in research articles.^19^ Each component of the estimation process had different data requirements and, as such, the input data used for each modelling component differed. The diverse data sought included the following sources: pharmaceutical companies that run surveillance networks, diagnostic laboratories, and clinical trial data; high-quality data from researchers including large multisite research collaborations, smaller studies, clinical trials, and well established research institutes based in low-income and middle-income countries (LMICs); data from public and private hospitals and public health institutes providing diagnostic testing; global surveillance networks; enhanced surveillance systems; national surveillance systems; and surveillance systems for specific organisms such as *Mycobacterium tuberculosis* and *Neisseria gonorrhoeae* (all sources are listed by data type in the appendix pp 8–15).

**Figure 1:**
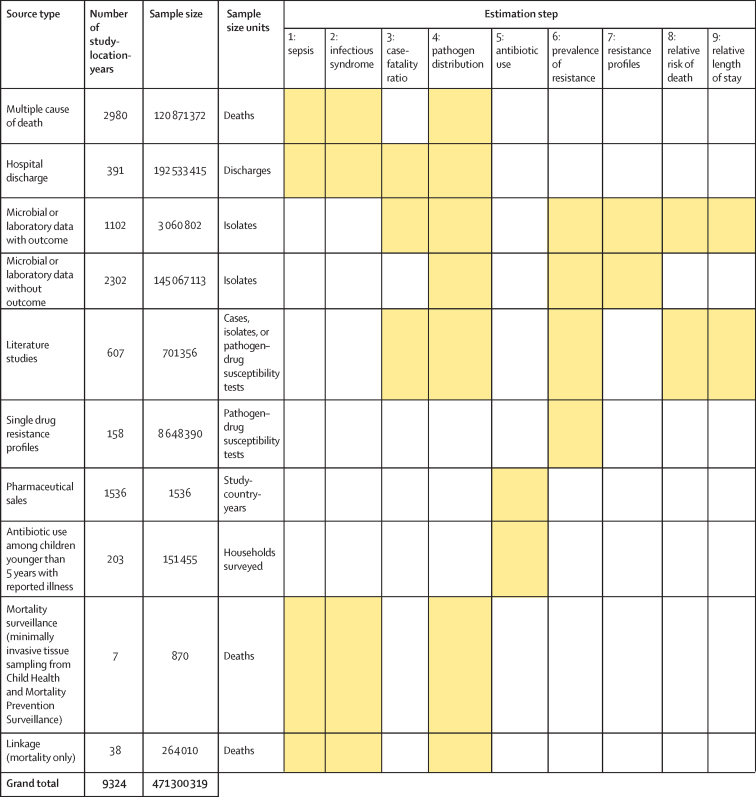
Data inputs by source type Total sample size for each source type, regardless of specific inclusion criteria for a given estimation step. Individual isolates that were tested multiple times for resistance to different antibiotics are listed only once here whenever isolates were identified uniquely in the data. For datasets where isolates could not be uniquely identified across pathogen–drug combinations, such as some antimicrobial resistance surveillance systems, some isolates might be double counted. Yellow boxes indicate that the source type was used in that estimation step. A full list of data sources included in this study, organised by data type, is included in the appendix (pp 8–15).

Figure 1 shows a summary of the distinct data types gathered and for which estimation step each data type was used. Also shown in figure 1 is the number of unique study-location-years and individual records or isolates available for each data type. Location-years of data refer to unique GBD locations and years for which we have records or isolates. In total, 471 million individual records or isolates covering 7585 study-location-years were used as input data to the estimation process. Table 1 shows the number of individual records or isolates used and number of countries covered in each of the five broad modelling components separately by GBD region. Two of five components included data from every GBD region and two of five included data from 19 of 21 GBD regions. Our models of sepsis and infectious syndrome were the most geographically sparse, covering 16 countries from ten regions; the input data for these models were highly detailed microdata that are only sparsely available. However, our framework for estimating the total envelope of infectious syndrome mortality used GBD cause-specific mortality estimates to minimise reliance on these sparse data.

**Table 1:** Data included in each modelling component by region and the fraction of countries represented in each region

	Component 1: sepsis and infectious syndrome models*	**Fraction of countries represented in component 1**	**Component 2: case-fatality ratio**	**Fraction of countries represented in component 2**	**Component 3: pathogen distribution**	**Fraction of countries represented in component 3**	**Component 4: fraction of resistance**†	**Fraction of countries represented in component 4**	**Component 5: relative risk**	**Fraction of countries represented in component 5**
Andean Latin America	0	0/3	1784	2/3	12 010	2/3	538 644	3/3	4338	2/3
Australasia	320 909	1/2	94 818	1/2	6 294 677	2/2	4 653 832	2/2	5211	2/2
Caribbean	0	0/19	2858	5/19	6225	5/19	68 078	10/19	529	1/19
Central Asia	0	0/9	43 852	2/9	2785	1/9	304 341	9/9	6065	1/9
Central Europe	0	0/13	371 112	10/13	627 844	11/13	3 148 864	13/13	397 885	10/13
Central Latin America	8 130 066	2/9	3 932 601	9/9	11 641 626	8/9	829 686	9/9	20 210	5/9
Central sub-Saharan Africa	0	0/6	0	0/6	770	2/6	40 243	6/6	0	0/6
East Asia	1 189 309	1/3	385 443	2/3	257 522	2/3	2 501 536	3/3	185 980	2/3
Eastern Europe	0	0/7	118 754	4/7	64 212	5/7	968 565	7/7	102 904	4/7
Eastern sub-Saharan Africa	292	3/15	6388	4/15	68 791	9/15	474 280	14/15	3436	2/15
High-income Asia Pacific	0	0/4	135 907	3/4	99 042	3/4	18 909 332	4/4	7577	3/4
High-income North America	84 520 574	2/3	7 184 424	3/3	7 255 147	2/3	32 205 001	3/3	14 071 025	2/3
North Africa and Middle East	0	0/21	209 479	13/21	53 833	16/21	531 120	21/21	90 079	10/21
Oceania	0	0/18	0	0/18	20	1/18	4297	12/18	0	0/18
South Asia	54	1/5	77 811	4/5	51 810	4/5	1 413 840	5/5	97 131	4/5
Southeast Asia	0	0/13	195 087	9/13	91 259	8/13	3 128 014	12/13	172 947	8/13
Southern Latin America	0	0/3	200 665	3/3	73 512	2/3	740 385	3/3	5000	1/3
Southern sub-Saharan Africa	4 696 789	1/6	80 717	2/6	4 699 304	2/6	910 509	6/6	1051	1/6
Tropical Latin America	17 224 511	1/2	3 988 611	1/2	20 956 932	2/2	286 450	2/2	6443	1/2
Western Europe	10 599 906	2/24	94 506 554	20/24	105 183 184	21/24	18 909 732	21/24	932 016	21/24
Western sub-Saharan Africa	83	2/19	26 985	9/19	21 896	10/19	369 482	18/19	14 880	2/19

Total sample size and fraction of countries covered for each modelling component by GBD region. The units for sample size are deaths for sepsis and infectious syndrome models; cases for case-fatality ratios; cases, deaths, or isolates for pathogen distribution; pathogen–drug tests for fraction of resistance; and pathogen–drug tests for relative risk. Sample sizes reflect model-specific selection criteria, resulting in lower totals for the sepsis, infectious syndrome, case-fatality ratio, and pathogen distribution models in this table than those in figure 1. Totals for fraction of resistance and relative risk are higher in this table than in figure 1 because of the difference in units for certain source types, such as microbial data (isolates in figure 1, pathogen–drug tests here). Several data sources inform multiple components; therefore, data points should not be summed across a row as that will lead to duplication. More information on the data types used and the components that they inform is presented in the appendix (pp 8–15). GBD=Global Burden of Diseases, Injuries, and Risk Factors Study.

* The data points listed in the sepsis and infectious syndrome models include only sources used to determine the fraction of sepsis in non-communicable diseases; maternal, neonatal, and nutritional diseases; and injuries, as well as the distribution of infectious syndromes; final estimates of the number of deaths in each infectious syndrome were generated by multiplying the fractions of sepsis and infection syndromes on GBD 2019 death estimates; GBD 2019 death estimates include 7417 sources with 28 106 location-years of data for under-5 mortality and 7355 sources with over 7322 location-years of data.

† For sources in the fraction of resistance modelling component, de-duplication across antibiotic resistance tests was not possible, leading to potential double counting, as seen in the high-income Asia Pacific region.

All data inputs for the models were empirical data, not modelled estimates, except for a custom meta-analysis of vaccine probe data that we did to estimate the fraction of pneumonia caused by *Streptococcus pneumoniae* (appendix pp 37–38). All study-level covariates for models, such as age and sex, were extracted from empirical data. All country-level covariates were modelled estimates that were produced previously for GBD 2019,^20^, ^21^ or those that were modelled by Browne and colleagues.^22^ We describe data inputs for each of ten estimation steps in greater detail in the following subsections and in the appendix (pp 17–18, 31, 34–35, 44, 54). Data input citations are available online.

### Estimation steps one and two: deaths in which infection played a role by infectious syndrome

First, to define the number of deaths where infection plays a role, we used GBD 2019 cause of death estimates^14^ to determine the number of deaths by age, sex, and location for which either the underlying cause of death was infectious or—for non-communicable, maternal, neonatal, nutritional, and injury deaths—for which the pathway to death was through sepsis. Sepsis is defined as a life-threatening organ dysfunction due to a dysregulated host response to infection.^23^ The methods used to estimate infectious underlying causes of death and sepsis deaths have been published previously^14^, ^24^ and are summarised in the appendix (pp 17–18).

In estimation step one, we used data for multiple causes of death covering 121 million deaths, 5·54 million hospital discharges with discharge status of death, and 264 000 records of multiple causes of death linked to hospital records from ten countries and territories, as well as 870 deaths from Child Health and Mortality Prevention Surveillance (CHAMPS) sites across six countries (appendix pp 17–18), to develop random effects logistic regression models to predict the fraction of sepsis occurring in each communicable, maternal, neonatal, and nutritional underlying cause of death; non-communicable underlying cause of death; and injury underlying cause of death. This approach follows the methods validated by many researchers in sepsis epidemiology^25^, ^26^, ^27^, ^28^ and used by Rudd and colleagues.^24^

We then multiplied the fraction of sepsis predicted from the logistic regression models onto GBD cause-specific mortality estimates to determine the mortality envelope for our analysis. Our mortality envelope consisted of all deaths in which infection played a role, which included all sepsis deaths with non-infectious underlying causes, plus all deaths with an infectious underlying cause in GBD 2019 (appendix pp 21–23).

In estimation step two, we used details on the pathways of disease provided in multiple causes of death and hospital discharge data in a second stage of random effects logistic regression models to further subdivide deaths in which infection played a role into 12 major infectious syndromes and one residual category. These regressions predicted the proportion of sepsis-related deaths that were caused by a given infectious syndrome separately for each communicable, maternal, neonatal, and nutritional underlying cause of death; non-communicable underlying cause of death; and injury underlying cause of death. We used this fraction to subdivide sepsis deaths with non-infectious underlying causes into specific infectious syndromes. For underlying causes of death that are themselves infectious, all deaths were assigned to their single corresponding infectious syndrome (eg, the GBD cause “lower respiratory infections” was assigned to the infectious syndrome “lower respiratory infections and all related infections in the thorax”; appendix pp 21–23).

Due to the pathogen distributions varying substantially for hospital-acquired and community-acquired infections in two infectious syndromes—lower respiratory and thorax infections and urinary tract infections—we further estimated the subdivision of these syndromes into community-acquired and hospital-acquired infections (appendix pp 17–30; table with community-acquired and hospital-acquired subdivisions presented on pp 24–25).

### Incidence of infectious syndromes disaggregated by age, sex, and location

For the nine infectious syndromes in this study that were estimated as one or more causes of death and disability in GBD 2019 (lower respiratory and thorax infections; CNS infections; typhoid, paratyphoid, and invasive non-typhoidal *Salmonella* spp; urinary tract infections; diarrhoea; tuberculosis; bacterial skin infections; cardiac infections; and gonorrhoea and chlamydia), we used GBD 2019 incidence estimates as a baseline for infectious syndrome incidence (appendix p 16).^14^ To this baseline, we added the number of incident cases of each infectious syndrome that co-occurred with underlying non-communicable diseases (NCDs); maternal, neonatal, and nutritional diseases (MNNDs); and injuries, which we calculated by dividing the number of infectious syndrome deaths that occurred with underlying NCDs, MNNDs, and injuries (by age, sex, location, and GBD cause) by syndrome-specific and pathogen-specific case-fatality ratios (CFRs; estimation described in the following subsection). Bloodstream infections, bone and joint infections, and intra-abdominal infections are not estimated in GBD, so for these infectious syndromes, we exclusively used the number of incident cases of each infectious syndrome that co-occurred with underlying NCDs, MNNDs, and injuries to estimate incidence (appendix pp 56–60).

### Estimation steps three and four: pathogen distribution for deaths and incident cases

To estimate the pathogen distribution of each infectious syndrome separately for deaths and incident cases for each age, sex, and location, we made use of multiple data sources. For estimation step three, we took data that linked pathogen-specific disease incidence to deaths to develop models for pathogen-specific CFRs that varied by age, location, and syndrome. We used the Bayesian meta-regression tool MR-BRT^29^ to estimate CFRs as a function of the Healthcare Access and Quality Index and various bias covariates (appendix pp 31–34).^21^ These CFRs allowed us to integrate sources that reported pathogen distribution only for deaths and those that reported only incidence by mapping the reported deaths by pathogen into implied cases by pathogen. After mapping, we had 157 million isolates and cases from 118 countries and territories to estimate the pathogen distribution of each infectious syndrome (estimation step four), with each dataset including a unique spectrum of pathogens and groups of pathogens. To incorporate all these heterogeneous data, we used a new modelling environment, termed multinomial estimation with partial and composite observations. This modelling environment allows for the inclusion of covariates in the network analysis^29^ and for Bayesian prior probability distributions to be incorporated. To model the infectious syndrome pathogen distribution comprehensively, we estimated, where applicable, the incidence and death proportions attributable to viral, fungal, parasitic, and bacterial pathogens; however, AMR burden was calculated only for selected bacteria for which resistance is clinically relevant and sufficient data are available. More details on this approach are provided in the appendix (pp 34–44).

### Estimation steps five to seven: prevalence of resistance by pathogen

We used data from 52·8 million isolates to analyse the proportion of phenotypic AMR for each pathogen—the proportion of infections that were drug resistant, hereafter referred to as prevalence of resistance—for 88 pathogen–drug combinations. We chose these 88 combinations by first creating an exhaustive list of all clinically relevant combinations for which we had any data and then eliminating combinations that did not meet minimum data availability and computational feasibility requirements for accurate statistical modelling (appendix pp 59–60).

For the pathogen–drug combinations in the 2014 WHO AMR global report on surveillance,^30^ as well as fluoroquinolone and multidrug resistance in *Salmonella enterica* serotypes Typhi and Paratyphi, we supplemented microbial datasets from collaborators and surveillance networks with aggregate microbiology data from systematic reviews and published surveillance reports. The number of positive isolates identified for each pathogen–drug combination is shown in the appendix (pp 90–91). Clinical and Laboratory Standard Institute (CLSI) guidelines were used to define minimum inhibitory concentration breakpoints when these minimums were provided. When only a phenotypic disk interpretation was available, we used the interpretation as provided. We used two categories of susceptibility: susceptible and non-susceptible. The non-susceptible group includes isolates reported as “non-susceptible”, “intermediate”, and “resistant”. To account for bias in resistance data provided by tertiary care facilities, we adjusted tertiary rates of resistance by crosswalking them to data from non-tertiary and mixed facilities using MR-BRT as described in the appendix (pp 45–48).^31^

We used a two-stage spatiotemporal modelling framework to estimate the prevalence of resistance in each pathogen–drug combination by location for 2018. Given the many challenges to data collection and reporting caused by the COVID-19 pandemic,^32^, ^33^ as well as our collaborators' process of data collation and cleaning, we were unable to collect more contemporary data; we assumed no change in prevalence of resistance for 2019. First, we fitted a stacked ensemble model between the input data and selected covariates from the list of plausible and health-related covariates available in GBD 2019 (appendix pp 48–49, 92–93); the estimates from the stacked ensemble model were then inputted into a spatiotemporal Gaussian process regression model^31^ to smooth the estimates in space and time. The exceptions to this modelling approach were multidrug-resistant (MDR) excluding extensively drug-resistant (XDR) tuberculosis and XDR tuberculosis, for which published GBD 2019 estimates were already available.^14^

Given the strong relationship between antibiotic consumption levels and the proliferation of resistance, we modelled antibiotic consumption at the national level to use as a covariate in the stacked ensemble model of prevalence of resistance. We analysed data from 65 Demographic and Health Surveys and 138 Multiple Indicator Cluster Surveys using model-based geostatistics to quantify antibiotic usage in LMICs. These LMIC-specific estimates of antibiotic usage were combined with pharmaceutical sales data from IQVIA, WHO, and the European Centre for Disease Prevention and Control (ECDC) by use of an ensemble spatiotemporal Gaussian process regression model to produce a location-year covariate on antibiotic consumption for all 204 countries and territories included in this study.^22^ Additional details on our estimation method for prevalence of resistance are available in the appendix (pp 44–53).

To account for multidrug resistance, we used line-level microbiology data that tested multiple antibiotics for the same isolate to produce Pearson correlation coefficients of the co-occurrence of resistance to different antibiotics. With these Pearson correlations and our prevalence of resistance estimates, we used an optimisation-based approach to solve for multivariate binomial distributions that define the prevalence of resistance of every combination of resistance to the antibiotics analysed. Every such distribution was characterised by a contingency table specifying probabilities of all combinations of resistance and susceptibility among the antibiotics analysed. The observed prevalence of each drug overall and Pearson correlations between drugs provided noisy partial observations of combinations of these entries. We optimised over the space of such contingency tables to find the nearest feasible distribution given the data, producing, for each pathogen, a set of resistance profiles: the proportions of bacteria with each combination of resistance and susceptibility among all the antibiotics analysed (appendix pp 48–49).

### Estimation steps eight and nine: relative risk of death for drug-resistant infection compared with drug-sensitive infections

Using data from 164 sources representing 511 870 patients with known outcome and resistance information, we estimated the relative risk of death for each pathogen–drug combination for a resistant infection compared with that of a drug-sensitive infection using MR-BRT. Because of data sparsity, we assumed the relative risk was the same for every syndrome, location, and age group; the assumptions on location and age group risk are consistent with those in the estimation process previously used by Cassini and colleagues.^10^ We used a two-stage nested mixed effects meta-regression model to estimate relative risk of death for each pathogen–drug combination that was adjusted for age, admission diagnosis, hospital-acquired versus community-acquired infection, and site of infection (appendix pp 54–56). For the non-fatal excess risk, we estimated the relative increase in length of stay associated with a resistant infection compared with that of a drug-sensitive infection, adjusted for length of stay prior to culture being drawn. Data on length of stay were available from 59 sources representing 455 906 admissions. We used the same modelling framework for excess length of stay as we used for relative risk of death. Due to data sparsity on the excess risk of death associated with drug-resistant *N gonorrhoeae*, we did not produce a fatal estimate for this pathogen.

To produce burden estimates of multiple pathogen–drug combinations that were mutually exclusive within a given pathogen (and thus could be added), we produced a population-attributable fraction (PAF) for each resistance profile with resistance to at least one drug (appendix pp 56–60). The PAF represents the proportional reduction in deaths or years lived with disability (YLDs) that would occur if all infections with the resistance profile of interest were instead susceptible to all antibiotics included in the analysis. When two or more antibiotics were resistant in a single profile, we used the relative risk for the antibiotic class that was the largest as the relative risk for calculating the PAF:

$$PAF=\frac{R_{kd}(RR_{kD}-1)}{1+\sum_{d=1}^{n}R_{kd}(RR_{kD}-1)}$$

Where *R* is prevalence of resistance, *RR* is relative risk, *K* is a pathogen with *d=1, …, n* resistance profiles with resistance to at least one antibiotic class, and *D* is the antibiotic class in profile *d* with the highest relative risk (appendix pp 56–60).

### Estimation step ten: computing burden attributable to drug resistance and burden associated with drug-resistant infections

We computed two counterfactuals to estimate the drug-resistant burden: the burden attributable to bacterial AMR based on the counterfactual of drug-sensitive infection and the burden associated with bacterial AMR based on the counterfactual of no infection (appendix pp 56–60). Briefly, to estimate the burden attributable to AMR, we first calculated the deaths attributable to resistance by taking the product of deaths for each underlying cause, the proportion of these deaths in which infection played a role, the proportion of infectious deaths attributable to each infectious syndrome, the proportion of infectious syndrome deaths attributable to each pathogen, and the mortality PAF for each resistance profile. We used previously described GBD methods^14^ to convert age-specific deaths into years of life lost (YLLs) using the standard counterfactual life expectancy at each age.^34^ To calculate attributable YLDs, we took the product of the infectious syndrome incidence, the proportion of infectious syndrome incident cases attributable to each pathogen, YLDs per incident case, and the non-fatal PAF. For resistance profiles that had resistance to more than one antibiotic class, we redistributed burden to the individual antibiotic classes proportionally on the basis of excess risk, providing a mutually exclusive burden for each pathogen–drug combination (appendix pp 56–60). To calculate DALYs, we took the sum of YLLs and YLDs. To estimate the overall AMR burden of the drug-sensitive counterfactual, we added the burden estimates of all the pathogen–drug combinations.

The approach for calculating the fatal burden associated with AMR was identical to that for fatal burden attributable to AMR, except we replaced the mortality PAF for each resistance profile with the prevalence of resistance in deaths. For the number of incident infections associated with resistance, we took the product of infectious syndrome incidence, the proportion of infectious incident cases attributable to each pathogen, and the prevalence of resistance in incident cases. On the basis of these death and incidence estimates, we then computed YLLs, YLDs, and DALYs associated with drug-resistant infections. We calculated YLLs using the same methods used to calculate YLLs attributable to AMR. We converted incidence into YLDs using a YLDs per incident case ratio for each infectious syndrome based on a proxy GBD cause (a simplified YLD calculation compared with the standard sequelae-based method; appendix pp 56–60). Finally, we calculated DALYs by summing YLLs and YLDs. To estimate the overall AMR burden of this counterfactual, we repeated the described calculations with the prevalence of resistance to one or more antibiotics estimated and summed across all pathogens.

### Uncertainty analysis and out-of-sample validation

Following previously described GBD methods,^14^ we propagated uncertainty from each step of the analysis into the final estimates of deaths and infections attributable to and associated with drug resistance by taking the 25th and 975th of 1000 draws from the posterior distribution of each quantity of interest. Out-of-sample validity estimates are provided in the appendix for our models of sepsis (pp 25–30), infectious syndrome distribution (pp 25–30), pathogen distribution (pp 43–44), prevalence of resistance (pp 51–53), and relative risk (pp 55–56).

### Role of the funding source

The funders of the study had no role in study design, data collection, data analysis, data interpretation, or the writing of the report.

## Results

We estimated that, in 2019, 1·27 million deaths (95% uncertainty interval [UI] 0·911–1·71) were directly attributable to resistance (ie, based on the counterfactual scenario that drug-resistant infections were instead drug susceptible) in the 88 pathogen–drug combinations evaluated in this study. On the basis of a counterfactual scenario of no infection, we estimated that 4·95 million deaths (3·62–6·57) were associated with bacterial AMR globally in 2019 (including those directly attributable to AMR). Table 2 provides estimates of deaths, YLLs, and DALYs from AMR for each counterfactual.

**Table 2:** Deaths, YLLs, YLDs, and DALYs (in counts and all-age rates) associated with and attributable to bacterial antimicrobial resistance, globally and by GBD super-region, 2019

	**Associated with resistance**				**Attributable to resistance**			
	Deaths	YLLs	DALYs	YLDs	Deaths	YLLs	DALYs	YLDs
**Counts, thousands**								
Global	4950 (3620–6570)	189 000 (145 000–245 000)	192 000 (146 000–248 000)	2290 (1520–3450)	1270 (911–1710)	47 600 (35 000–63 400)	47 900 (35 300–63 700)	275 (161–439)
Central Europe, eastern Europe, and central Asia	283 (190–403)	7530 (5240–10 500)	7630 (5320–10 600)	102 (69–140)	73·7 (48·7–105)	1980 (1350–2790)	1990 (1360–2800)	9·95 (4·79–16·8)
High income	604 (434–824)	10 100 (6960–14 200)	10 300 (7040–14 400)	123 (79·7–183)	141 (98·6–197)	2390 (1620–3400)	2410 (1640–3420)	20·2 (12·7–31·2)
Latin America and Caribbean	338 (243–453)	9550 (6770–12 900)	9640 (6830–13 100)	97·2 (63·2–146)	84·3 (60·3–117)	2370 (1660–3310)	2380 (1680–3330)	16 (9·79–24·9)
North Africa and Middle East	256 (174–362)	9970 (6880–13 900)	10 100 (6970–14 000)	116 (73·4–176)	68·3 (45·6–99)	2590 (1770–3700)	2610 (1790–3720)	20·7 (12–33·5)
South Asia	1390 (1030–1830)	58 900 (44 800–76 300)	59 900 (45 700–77 500)	1000 (638–1550)	389 (273–538)	16 000 (11 500–21 600)	16 100 (11 600–21 700)	111 (58·5–188)
Southeast Asia, east Asia, and Oceania	1020 (678–1460)	27 500 (18 700–38 600)	27 900 (19 100–39 100)	437 (256–776)	254 (167–369)	6830 (4620–9840)	6870 (4670–9890)	45·6 (25–80·1)
Sub-Saharan Africa	1070 (847–1340)	65 800 (51 400–83 600)	66 200 (51 800–84 000)	416 (270–599)	255 (196–331)	15 400 (11 700–19 900)	15 500 (11 800–20 000)	51·1 (30·2–81·8)
**Rates, per 100 000**								
Global	64·0 (46·8–84·9)	2448·1 (1868·9–3170·3)	2477·7 (1889·9–3199·1)	29·6 (19·7–44·5)	16·4 (11·8–22·0)	615·1 (452·4–819·1)	618·7 (455·7–823·2)	3·6 (2·1–5·7)
Central Europe, eastern Europe, and central Asia	67·7 (45·4–96·6)	1802·5 (1253·9–2515·1)	1826·9 (1274·5–2545·4)	24·4 (16·5–33·6)	17·6 (11·7–25·3)	474·3 (323·0–667·3)	476·7 (325·2–671·0)	2·4 (1·1–4·0)
High income	55·7 (40·1–76·0)	935·3 (641·9–1310·1)	946·7 (649·8–1327·2)	11·3 (7·3–16·9)	13·0 (9·1–18·2)	220·4 (149·9–314·0)	222·3 (151·5–315·9)	1·9 (1·2–2·9)
Latin America and Caribbean	57·9 (41·6–77·6)	1633·8 (1158·7–2215·9)	1650·5 (1169·0–2236·6)	16·6 (10·8–25·0)	14·4 (10·3–20·0)	405·3 (284·8–566·6)	408·1 (286·9–570·0)	2·7 (1·7–4·3)
North Africa and Middle East	42·0 (28·7–59·5)	1637·5 (1130·4–2283·2)	1656·6 (1145·2–2300·9)	19·1 (12·1–28·9)	11·2 (7·5–16·3)	425·6 (291·2–608·4)	429·0 (293·7–611·5)	3·4 (2·0–5·5)
South Asia	76·8 (57·2–101·2)	3262·6 (2482·4–4228·2)	3318·1 (2532·9–4291·7)	55·4 (35·4–86·0)	21·5 (15·1–29·8)	885·8 (636·3–1194·6)	892·0 (643·1–1200·2)	6·2 (3·2–10·4)
Southeast Asia, east Asia, and Oceania	47·1 (31·4–67·7)	1272·6 (866·8–1789·0)	1292·8 (884·7–1811·4)	20·2 (11·8–35·9)	11·7 (7·8–17·1)	316·1 (213·9–455·7)	318·2 (216·1–458·0)	2·1 (1·2–3·7)
Sub-Saharan Africa	98·9 (78·6–124·2)	6105·3 (4770·2–7749·1)	6143·9 (4802·8–7792·2)	38·6 (25·1–55·6)	23·7 (18·2–30·7)	1432·0 (1084·6–1848·1)	1436·7 (1090·0–1853·5)	4·7 (2·8–7·6)

DALYs=disability-adjusted life-years. GBD=Global Burden of Diseases, Injuries, and Risk Factors Study. YLDs=years lived with disability. YLLs=years of life lost.

**Figure 2:**
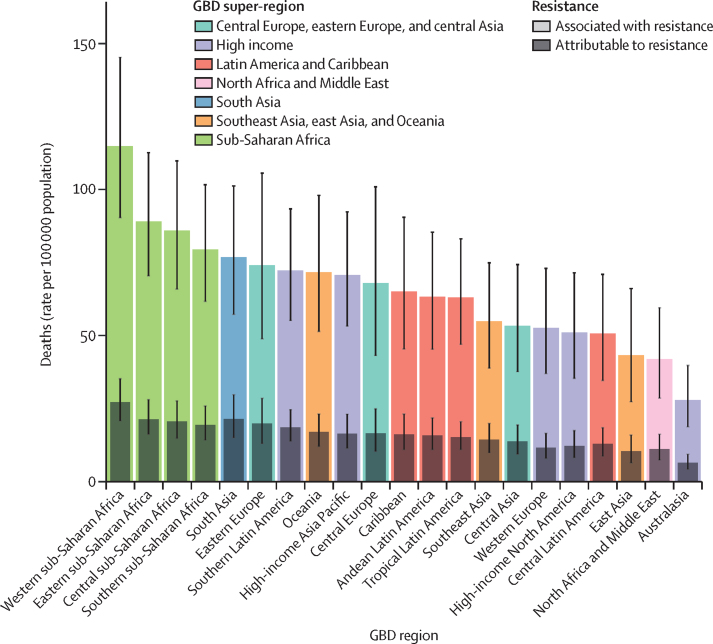
All-age rate of deaths attributable to and associated with bacterial antimicrobial resistance by GBD region, 2019 Estimates were aggregated across drugs, accounting for the co-occurrence of resistance to multiple drugs. Error bars show 95% uncertainty intervals. GBD=Global Burden of Diseases, Injuries, and Risk Factors Study.

We estimated that among the 21 GBD regions, Australasia had the lowest AMR burden in 2019, with 6·5 deaths per 100 000 (95% UI 4·3–9·4) attributable to AMR and 28·0 deaths per 100 000 (18·8–39·9) associated with AMR in 2019 (figure 2). Western sub-Saharan Africa had the highest burden, with 27·3 deaths per 100 000 (20·9–35·3) attributable to AMR and 114·8 deaths per 100 000 (90·4–145·3) associated with AMR. Five regions had all-age death rates associated with bacterial AMR higher than 75 per 100 000: all four regions of sub-Saharan Africa and south Asia. Although sub-Saharan Africa had the highest all-age death rate attributable to and associated with AMR, the percentage of all infectious deaths attributable to AMR was lowest in this super-region (appendix p 97).

**Figure 3:**
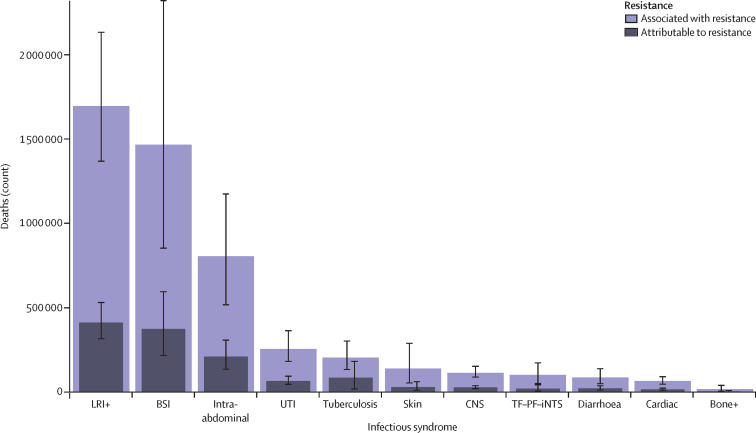
Global deaths (counts) attributable to and associated with bacterial antimicrobial resistance by infectious syndrome, 2019 Estimates were aggregated across drugs, accounting for the co-occurrence of resistance to multiple drugs. Error bars show 95% uncertainty intervals. Does not include gonorrhoea and chlamydia because we did not estimate the fatal burden of this infectious syndrome. Bone+=infections of bones, joints, and related organs. BSI=bloodstream infections. Cardiac=endocarditis and other cardiac infections. CNS=meningitis and other bacterial CNS infections. Intra-abdominal=peritoneal and intra-abdominal infections. LRI+=lower respiratory infections and all related infections in the thorax. Skin=bacterial infections of the skin and subcutaneous systems. TF–PF–iNTS= typhoid fever, paratyphoid fever, and invasive non-typhoidal *Salmonella* spp. UTI=urinary tract infections and pyelonephritis.

Three infectious syndromes dominated the global burdens attributable to and associated with AMR in 2019: lower respiratory and thorax infections, bloodstream infections, and intra-abdominal infections (figure 3). Combined, these three syndromes accounted for 78·8% (95% UI 70·8–85·2) of deaths attributable to AMR in 2019; lower respiratory infections alone accounted for more than 400 000 attributable deaths and 1·5 million associated deaths (figure 3).

**Figure 4:**
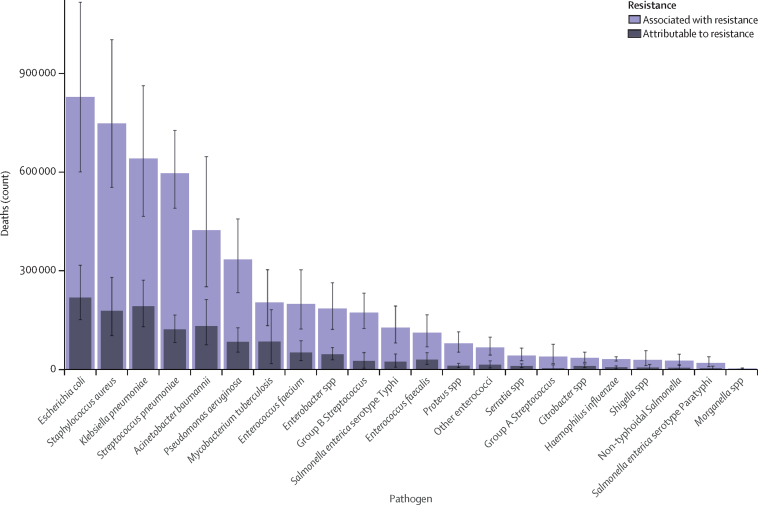
Global deaths (counts) attributable to and associated with bacterial antimicrobial resistance by pathogen, 2019 Estimates were aggregated across drugs, accounting for the co-occurrence of resistance to multiple drugs. Error bars show 95% uncertainty intervals.

In 2019, six pathogens were each responsible for more than 250 000 deaths associated with AMR (figure 4): *E coli, Staphylococcus aureus, K pneumoniae, S pneumoniae, Acinetobacter baumannii,* and *Pseudomonas aeruginosa*, by order of number of deaths. Together, these six pathogens were responsible for 929 000 (95% UI 660 000–1 270 000) of 1·27 million deaths (0·911–1·71) attributable to AMR and 3·57 million (2·62–4·78) of 4·95 million deaths (3·62–6·57) associated with AMR globally in 2019. Six more pathogens were each responsible for between 100 000 and 250 000 deaths associated with AMR: *M tuberculosis, Enterococcus faecium, Enterobacter* spp*, Streptococcus agalactiae* (group B *Streptococcu*s), *S* Typhi, and *Enterococcus faecalis.* For deaths attributable to AMR, *E coli* was responsible for the most deaths in 2019, followed by *K pneumoniae, S aureus, A baumannii, S pneumoniae,* and *M tuberculosis.*

**Figure 5:**
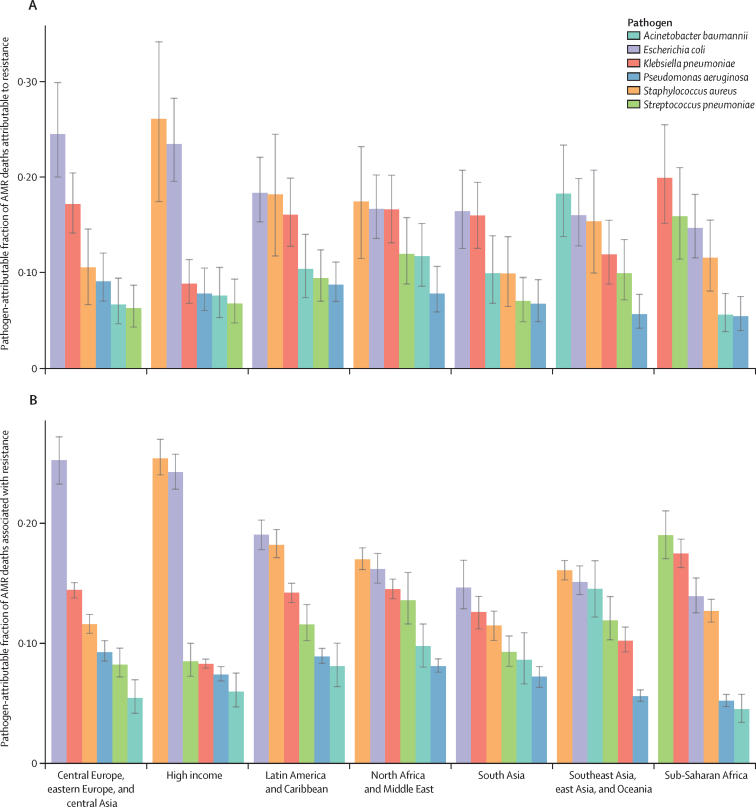
Pathogen-attributable fraction of deaths attributable to (A) and associated with (B) bacterial AMR for the six leading pathogens by GBD super-region, 2019 Error bars show 95% uncertainty intervals. AMR=antimicrobial resistance. GBD=Global Burden of Diseases, Injuries, and Risk Factors Study.

The share of AMR burden caused by each of the six leading pathogens differed substantially across GBD super-regions. In the high-income super-region, approximately half of the fatal AMR burden was linked to two pathogens: *S aureus* (constituting 26·1% [95% UI 17·4–34·1] of deaths attributable to AMR and 25·4% [24·1–27·0] of deaths associated with AMR) and *E coli* (constituting 23·4% [19·5–28·2] of deaths attributable to AMR and 24·3% [22·9–25·8] of deaths associated with AMR; figure 5). By contrast, in sub-Saharan Africa, the leading pathogens were distinct from those of the high-income super-region, and each represented a smaller share of the AMR burden; *S pneumoniae* contributed to 15·9% (11·4–21·0) of the deaths attributable to AMR and 19·0% (17·1–21·1) of the deaths associated with AMR, whereas *K pneumoniae* contributed to 19·9% (15·1–25·4) of the deaths attributable to AMR and 17·5% (16·3–18·7) of the deaths associated with AMR.

**Figure 6:**
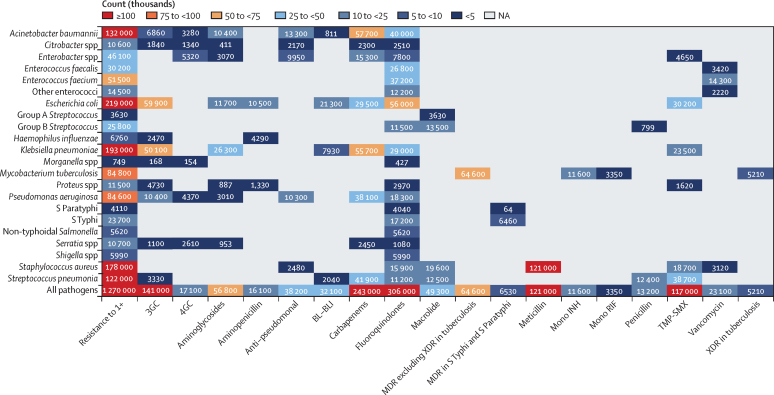
Global deaths (counts) attributable to bacterial antimicrobial resistance by pathogen–drug combination, 2019 For this figure, only deaths attributable to resistance, not deaths associated with resistance, are shown due to the very high levels of correlation for resistance patterns between some drugs. 3GC=third-generation cephalosporins. 4GC=fourth-generation cephalosporins. Anti-pseudomonal=anti-pseudomonal penicillin or beta-lactamase inhibitors. BL-BLI=β-lactam or β-lactamase inhibitors. MDR=multidrug resistance. Mono INH=isoniazid mono-resistance. Mono RIF=rifampicin mono-resistance. NA=not applicable. Resistance to 1+=resistance to one or more drug. *S* Paratyphi=*Salmonella enterica* serotype Paratyphi. *S* Typhi=*S enterica* serotype Typhi. TMP-SMX=trimethoprim-sulfamethoxazole. XDR=extensive drug resistance.

In 2019, meticillin-resistant *S aureus* was the one pathogen–drug combination in our analysis with more than 100 000 deaths and 3·5 million DALYs attributable to resistance (figure 6; appendix pp 121–22, 129). Six more pathogen–drug combinations each caused between 50 000 and 100 000 resistance-attributable deaths in 2019: MDR excluding XDR tuberculosis, third-generation cephalosporin-resistant *E coli*, carbapenem-resistant *A baumannii*, fluoroquinolone-resistant *E coli*, carbapenem-resistant *K pneumoniae*, and third-generation cephalosporin-resistant *K pneumoniae* (figure 6). In the next tier of pathogen–drug combinations, ten combinations each caused between 25 000 and 50 000 deaths attributable to AMR. Four of these ten combinations included fluoroquinolone resistance, three included carbapenem resistance, and two had trimethoprim-sulfamethoxazole resistance.

In the appendix, we present the equivalent AMR findings for DALYs instead of deaths (pp 124–29), as well as the burden attributable to and associated with specific pathogen–drug combinations by age group (neonatal, post-neonatal, age 1–4 years, and age 5 years or older) and super-region (pp 106–18).

**Figure 7:**
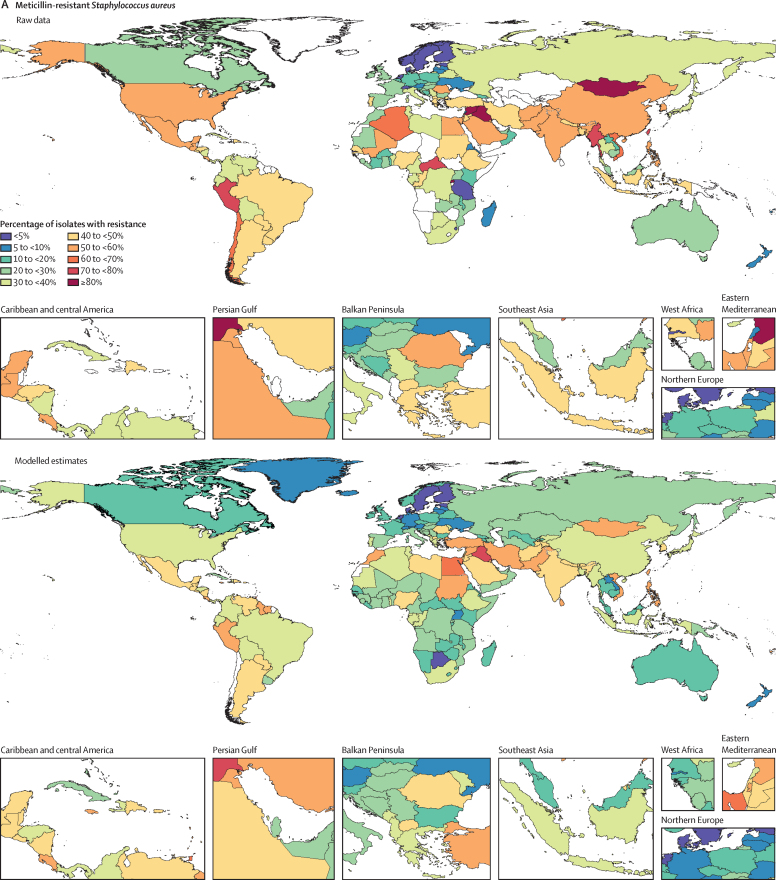

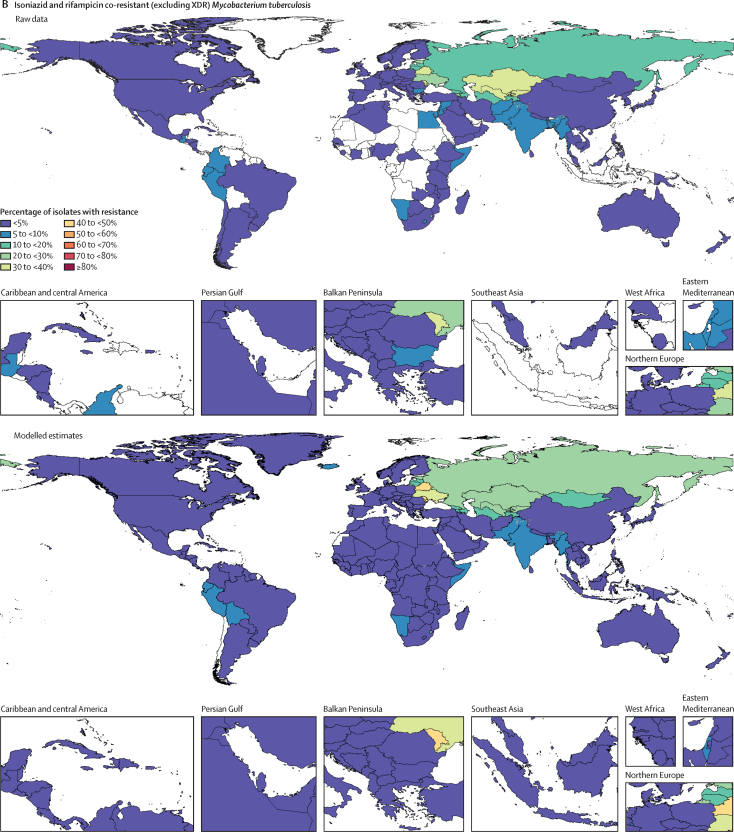

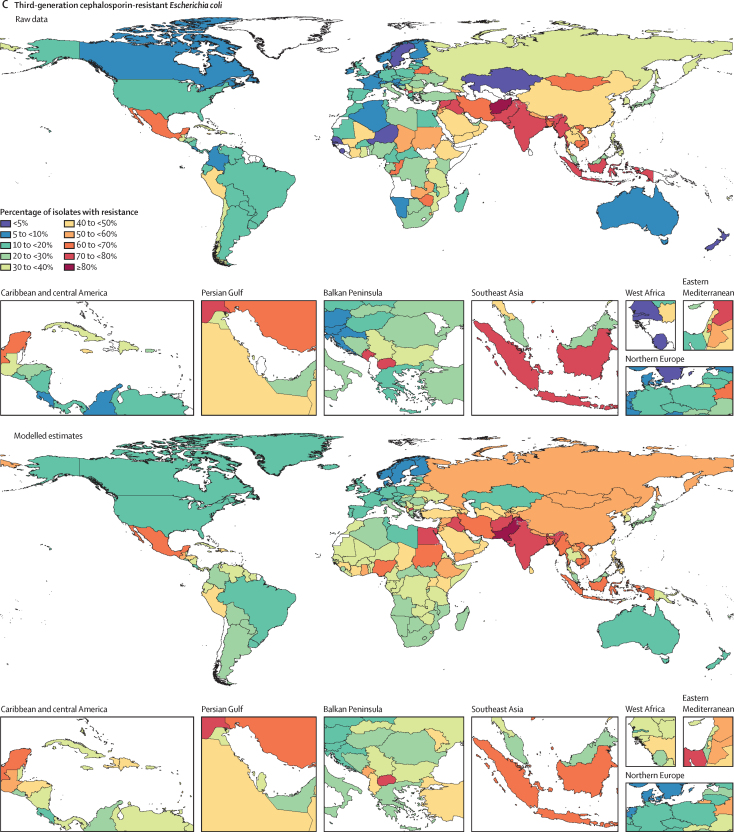

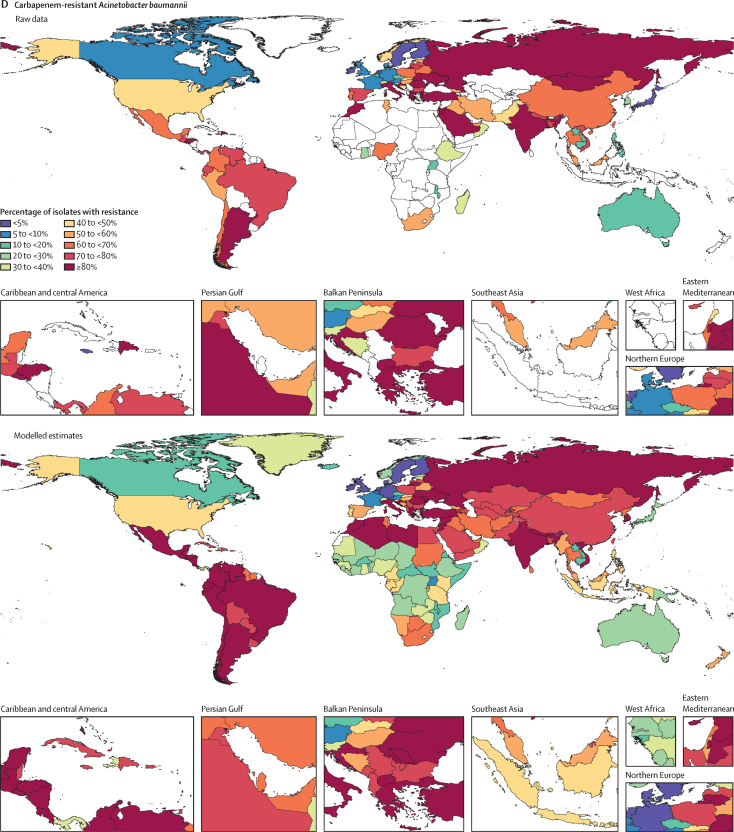

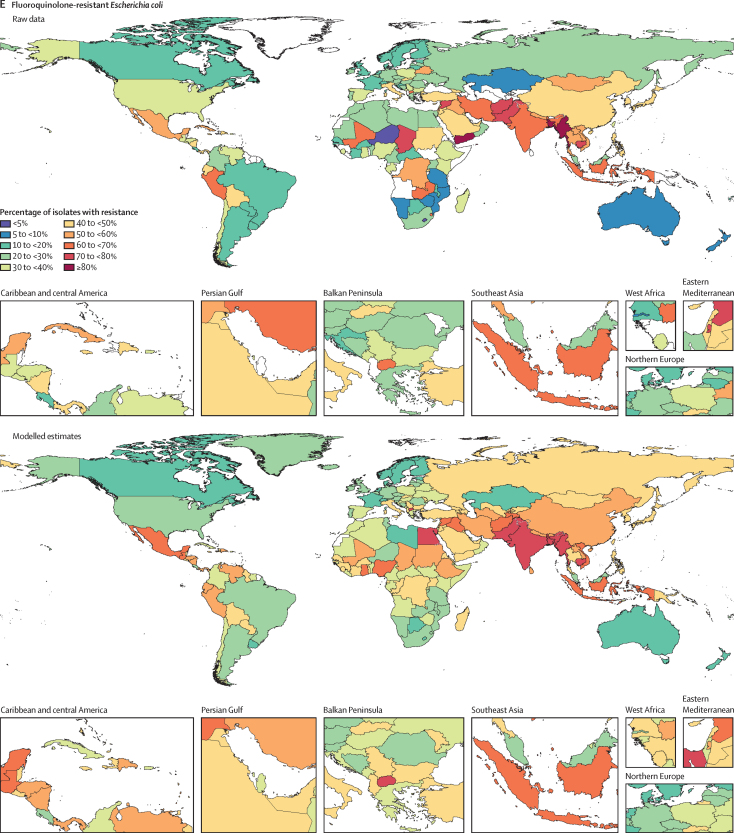

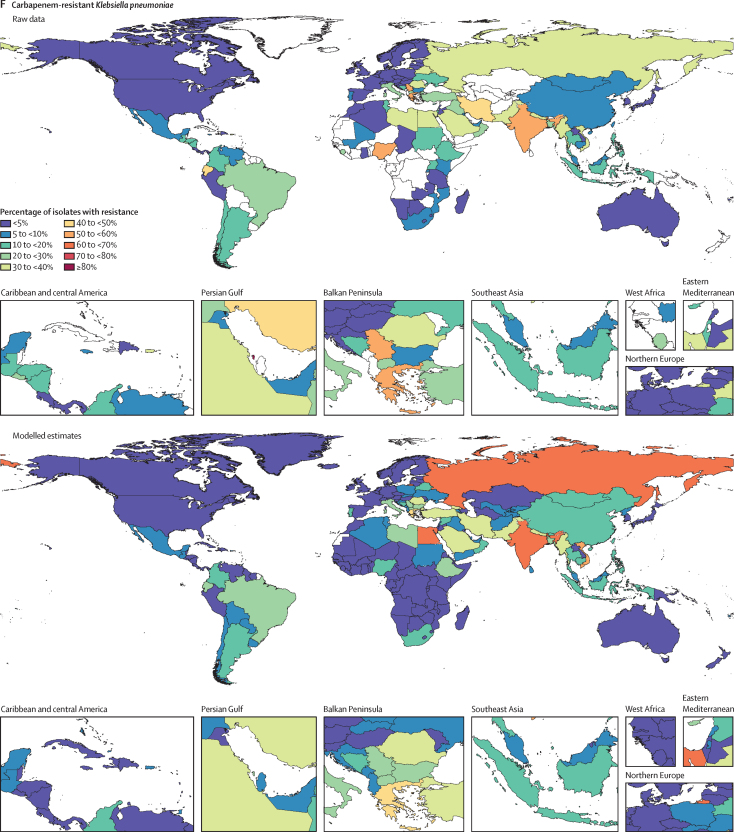

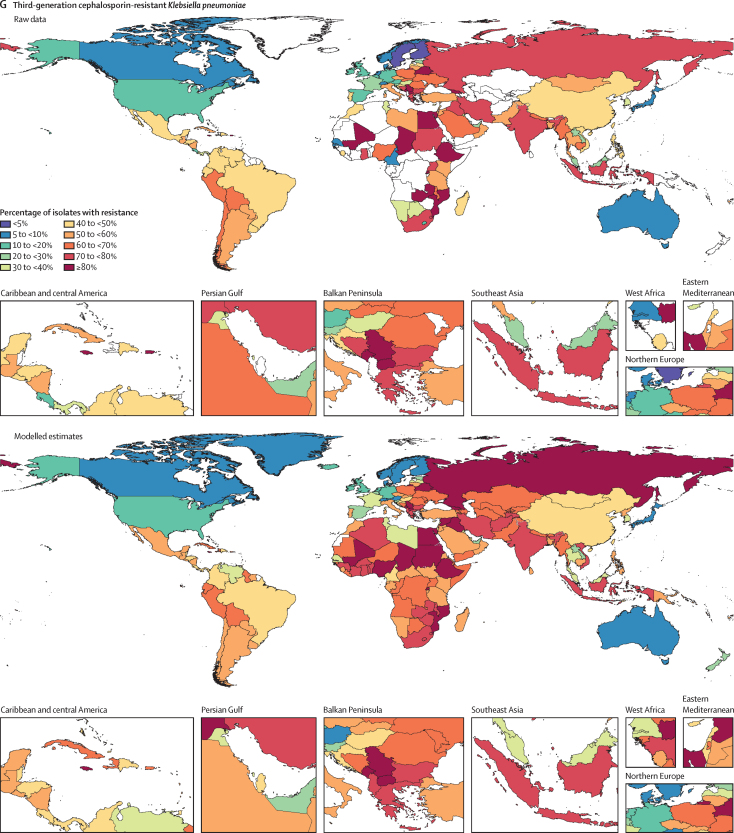
Raw data and modelled estimates for the percentage of pathogen isolates that are resistant by country and territory, 2019 Meticillin-resistant *Staphylococcus aureus* (A), isoniazid and rifampicin co-resistant (excluding XDR) *Mycobacterium tuberculosis* (B), third-generation cephalosporin-resistant *Escherichia coli* (C), carbapenem-resistant *Acinetobacter baumannii* (D), fluoroquinolone-resistant *E coli* (E), carbapenem-resistant *Klebsiella pneumoniae* (F), and third-generation cephalosporin-resistant *K pneumoniae* (G). Locations with no data or modelled estimates are presented in white. XDR=extensively drug resistant.

Among the seven leading pathogen–drug combinations for deaths attributable to resistance, the proportion of isolates estimated to be resistant varied substantially by country and territory (figure 7A–G). For meticillin-resistant *S aureus*, resistance was generally highest (60% to less than 80%) in countries in north Africa and the Middle East (eg, Iraq and Kuwait) and lowest (less than 5%) in several countries in Europe and sub-Saharan Africa (figure 7A). For isoniazid and rifampicin co-resistant (MDR excluding XDR) *M tuberculosis*, isolate resistance was highest (primarily 10% to less than 30%) in eastern Europe and under 5% in many countries around the world (figure 7B). To show where data are available and how the modelled estimates differ from the input data, figure 7 also shows the raw, unadjusted prevalence of resistance for each of the seven leading pathogen–drug combinations.

## Discussion

The global burden associated with drug-resistant infections assessed across 88 pathogen–drug combinations in 2019 was an estimated 4·95 million (95% UI 3·62–6·57) deaths, of which 1·27 million (0·911–1·71) deaths were directly attributable to drug resistance. In other words, if all drug-resistant infections were replaced by no infection, 4·95 million deaths could have been prevented in 2019, whereas if all drug-resistant infections were replaced by drug-susceptible infections, 1·27 million deaths could have been prevented. Compared with all underlying causes of death in GBD 2019, AMR would have been the third leading GBD Level 3 cause of death in 2019, on the basis of the counterfactual of no infection; only ischaemic heart disease and stroke accounted for more deaths that year.^14^ Using the counterfactual of susceptible infection, AMR would have been the 12th leading GBD Level 3 cause of death globally, ahead of both HIV and malaria (more information on GBD causes by level presented in the appendix pp 18, 67–75).^14^ By any metric, bacterial AMR is a leading global health issue.^12^ Additionally, our analysis showed that AMR all-age death rates were highest in some LMICs, making AMR not only a major health problem globally but a particularly serious problem for some of the poorest countries in the world.

All six of the leading pathogens contributing to the burden of AMR in 2019 (*E coli, S aureus, K pneumoniae, S pneumoniae, A baumannii*, and *P aeruginosa*) have been identified as priority pathogens by WHO^34^ and AMR has been highlighted in the political arena through the Global Action Plan on AMR,^8^ the UN Interagency Coordination Group,^35^ the One Health Global Leaders Group,^36^ and several others. However, only one of these pathogens has been the focus of a major global health intervention programme—*S pneumoniae,* primarily through pneumococcal vaccination.^37^ Furthermore, the first Sustainable Development Goal^38^ indicator for antimicrobial resistance was only proposed in 2019, and this indicator (3.d.2) is very limited in scope.^39^, ^40^ Our findings, which—to our knowledge—are the most comprehensive estimates of the burden of bacterial AMR to date, clearly show that drug resistance in each of these leading pathogens is a major global health threat that warrants more attention, funding, capacity building, research and development, and pathogen-specific priority setting from the broader global health community.

Resistance to fluoroquinolones and β-lactam antibiotics (ie, carbapenems, cephalosporins, and penicillins)—antibiotics often considered first line for empirical therapy of severe infections^41^—accounted for more than 70% of deaths attributable to AMR across pathogens. In 2017, WHO published a priority list for developing new and effective antibiotic treatments. The list was intended to inform research and development priorities related to new antibiotics and put the most emphasis on pathogens with multidrug resistance that cause severe and often deadly infections in health-care and nursing home settings. Although the intention of this list was to set new antibiotic research and development priorities rather than identify the most burdensome pathogen–drug combinations, its utility in dictating priorities has still been limited by the absence of a global assessment of the burden of bacterial AMR. Only five of the seven pathogen–drug combinations that we estimated to have caused the most deaths attributable to bacterial AMR in 2019 are currently on the list; MDR tuberculosis and fluoroquinolone-resistant *E coli* are not included.^34^ Additionally, meticillin-resistant *S aureus*—the leading pathogen–drug combination in our analysis for attributable deaths in 2019—is listed as “high” but not “critical” priority.^34^ WHO has explained that the absence of MDR tuberculosis from its priority list is because it has already been established globally as a top priority for innovative treatments, but this exclusion remains a source of considerable debate.^42^, ^43^ Although many factors were considered in producing the WHO priority list, these new estimates of the global burden of specific pathogen–drug combinations can inform future work on WHO priority pathogen–drug combinations.

Intervention strategies for addressing the challenge of bacterial AMR fall into five main categories. First, the principles of infection prevention and control remain a foundation for preventing infections broadly and a cornerstone in combating the spread of AMR.^44^ These include both hospital-based infection prevention and control programmes focused on preventing health-care-acquired infections, and community-based programmes focused on water, sanitation, and hygiene. Community-based programmes are particularly important in LMICs where the AMR burden is highest and clean water and sanitation infrastructure is weak; sustained support for these programmes is an essential element of combating AMR.

Second, preventing infections through vaccinations is paramount for reducing the need for antibiotics. Vaccines are available for only one of the six leading pathogens (*S pneumoniae*), although new vaccine programmes are underway for *S aureus, E coli,* and others.^45^ Vaccination programmes are an important strategy for preventing *S pneumoniae,*^46^ and vaccine development is crucial for pathogens that currently have no vaccine. Other vaccines, such as the influenza or rotavirus vaccines, also play a role in preventing febrile illness, which can lead to a reduction in antibiotic prescribing and can reduce AMR emergence even for pathogens without vaccines.^45^

Third, reducing exposure to antibiotics unrelated to treating human disease is an important potential way to reduce risk. Increased use of antibiotics in farming has been identified as a potential contributor to AMR in humans,^2^, ^47^, ^48^, ^49^ although the direct causal link remains controversial.^50^, ^51^

Fourth, minimising the use of antibiotics when they are not necessary to improve human health—such as treating viral infections—should be prioritised. To this end, building infrastructure that allows clinicians to diagnose infection accurately and rapidly is crucial so that antimicrobial use can be narrowed or stopped when appropriate.^52^ The notion of antibiotic stewardship remains a core strategy in most national and international AMR management plans, although barriers to implementing stewardship programmes in LMICs should be addressed.^53^, ^54^

Fifth, maintaining investment in the development pipeline for new antibiotics—and access to second-line antibiotics in locations without widespread access—is essential. In the past few decades, investments have been small compared with those in other public health issues with similar or less impact.^55^ Given the global importance of bacterial AMR, more assessment of which policies have worked, and where, is urgently needed.

Many might expect that with higher antibiotic consumption in high-resource settings, the burden of bacterial AMR would be correspondingly higher in those settings. We found, however, that the highest rates of death were in sub-Saharan Africa and south Asia. High bacterial AMR burdens are a function of both the prevalence of resistance and the underlying frequency of critical infections such as lower respiratory infections, bloodstream infections, and intra-abdominal infections, which are higher in these regions.^14^ Other drivers of the observed higher burden in LMICs include the scarcity of laboratory infrastructure making microbiological testing unavailable to inform treatment to stop or narrow antibiotics,^56^ the inappropriate use of antibiotics driven by insufficient regulations and ease of acquisition,^57^ inadequate access to second-line and third-line antibiotics, counterfeit or substandard antibiotics that can drive resistance,^52^, ^58^, ^59^ and poor sanitation and hygiene.^60^, ^61^, ^62^

The higher burden in low-resource health systems highlights the importance—both for the management of individual patients and for the surveillance of AMR—of well developed national action plans and laboratory infrastructure in all regions and countries. The pattern of AMR varies geographically, with different pathogens and pathogen–drug combinations dominating in different locations. Our regional estimates could prove useful for tailoring local responses as a one size fits all approach might be inappropriate. Although antibiotic stewardship is a foundational aspect for preventing the spread of AMR, limiting access to antibiotics is not a suitable response to AMR in all settings. In fact, it could be argued that an increase in access to antibiotics would decrease the AMR burden in some locations where second-line antibiotics are unavailable and would be lifesaving; this might well be the case in western sub-Saharan Africa. By contrast, limiting access to antibiotics in south Asia through stewardship programmes might be the appropriate response for that region because antibiotic overuse or misuse is believed to be a major driver of AMR there.^58^ AMR is a global problem and one that requires both global action and nationally tailored responses.

This study evaluated both the burden of bacterial infections associated with drug resistance and the burden directly attributable to drug resistance.^13^ At the global level, the difference is nearly four-times that attributable to AMR. We estimated both measures of burden because there is insufficient evidence to determine the extent to which drug-resistant infections would be replaced by no infection or susceptible infection if drug resistance was eliminated. Some evidence from the spread of meticillin-resistant *S aureus* and meticillin-susceptible *S aureus* suggests that drug-resistant infections do not simply replace drug-susceptible infections,^63^, ^64^ but this finding might not generalise to all other pathogens and other mechanisms of resistance.

Both measures are informative in different ways. For instance, when considering the specific burden of each pathogen–drug combination, we believe that the burden attributable to resistance is more appropriate because very high levels of co-resistance among some drugs lead to many deaths being duplicated across drugs when considering burden associated with resistance. When thinking about the role of vaccination to combat AMR, the no-infection counterfactual is more appropriate because infections would be eliminated, whereas interventions based on antimicrobial stewardship might be better informed by the susceptible infection counterfactual because some resistant bacteria might be replaced by susceptible bacteria.^22^ In either case, the magnitude of the global bacterial AMR problem is very large and likely bounded by the two measures.

Our ability to compare our estimates with previous estimates is somewhat limited. The only global burden estimates for AMR are from the Review on Antimicrobial Resistance,^1^ which did not provide death estimates by pathogen–drug combination, making direct comparison challenging. The Review on Antimicrobial Resistance estimated 700 000 deaths in 2014 attributable to resistance to six pathogens: HIV, tuberculosis, malaria, *S aureus, E coli*, and *K pneumoniae*. We produced estimates for four of those pathogens—tuberculosis, *S aureus, E coli,* and *K pneumoniae*—and estimated 670 000 deaths attributable to resistance to those pathogens in 2019.

Cassini and colleagues^10^ produced an estimate for the EU of 16 pathogen–antibiotic combinations in 2015. We produced estimates for 11 of these 16 combinations; we did not estimate colistin resistance in *E coli, P aeruginosa,* or *A baumannii* because of the paucity of data on colistin resistance in LMICs, or multidrug resistance in *P aeruginosa* or *A baumannii* because of our approach to MDR infections. For the 11 pathogen–drug combinations that overlap, Cassini and colleagues estimated approximately 30 000 deaths and 796 000 DALYs caused by resistance in the EU in 2015. For these same 11 pathogen–drug combinations, we estimated 23 100 deaths (95% UI 14 600–34 600) and 393 000 DALYs (246 000–595 000) attributable to bacterial AMR for western and central Europe combined. Cassini and colleagues used a mix of both counterfactuals to inform their estimates, so it is expected that their EU estimate is somewhat higher than ours for the susceptible counterfactual. This comparison is not perfect because there is not complete overlap in the locations included in western and central Europe and EU member countries (ie, Switzerland is included in our estimate and not in the EU designation, whereas Estonia is in the EU but is part of our eastern Europe region; appendix pp 100–05), but it offers some idea of how our estimates compare with those of previous publications.

Some of our estimates might be unexpected and deserve special attention, particularly the high burden in sub-Saharan Africa and the burden of carbapenem-resistant *A baumannii*. Although we estimated sub-Saharan Africa to be the super-region with the lowest percentage of infectious deaths attributable to AMR (appendix p 97), the rate of deaths in which infection plays a role was so much greater in sub-Saharan Africa than in other super-regions that it overcame a relatively low prevalence of resistance and was the super-region with the highest estimated AMR burden in 2019.

Regarding carbapenem-resistant *A baumannii*, we estimated that it was the fourth leading pathogen–drug combination globally for 2019, responsible for slightly fewer deaths than third-generation cephalosporin-resistant *E coli*. At first glance, this finding seems to contrast with other estimates such as those from Cassini and colleagues or the CDC, who have estimated the burden of carbapenem-resistant *A baumannii* to be substantially lower than that of third-generation cephalosporin-resistant *E coli*.^6^, ^10^ When assessed by super-region, however, our results are much more consistent with the published literature: similar to the CDC and ECDC, we found the burden of third-generation cephalosporin-resistant *E coli* to exceed that of carbapenem-resistant *A baumannii* in high-income settings, whereas the inverse pattern was found in south Asia, where a higher relative burden of carbapenem-resistant *A baumannii* than that in high-income regions has been documented.^11^ Our global burden was strongly influenced by this higher relative burden of carbapenem-resistant *A baumannii* in south Asia and other LMICs.

Our estimate for the burden of resistance is confined to the 88 pathogen–drug combinations we analysed. Expanding our resistance analysis to more pathogen–drug combinations—particularly adding viruses, parasites, and fungi—would increase our estimate of the burden and could alter some of the results reported, depending on the correlation structure of resistance between the newly added and original 88 pathogen–drug combinations. It would provide a more thorough account of the threat of AMR and improve the accuracy of our estimates for the combinations reported here that share a high degree of co-resistance with combinations not yet analysed.

This study has several limitations, the most important being the sparsity of data from many LMICs on the distribution of pathogens by infectious syndrome, the prevalence of resistance for key pathogen–drug combinations, and the number of deaths involving infection; and the severe scarcity of data linking laboratory results to outcomes such as death. 19 of 204 countries and territories had no data available for any of our modelling components. Limited availability of data in some parts of the world was particularly consequential for the prevalence of resistance and relative risk modelling components; we assumed that the relative risk for each pathogen–drug combination, as well as the correlation structure of resistance between drugs, was the same in every location, age, and infectious syndrome. This might underestimate the AMR burden for LMICs, since the relative risk might be higher in locations where fewer second-line and third-line antibiotics are available. Assuming a single relative risk for all infectious syndromes is a potentially strong assumption; it is not immediately clear what direction this biases results, but it might lead to overestimation. Another substantial assumption we made due to insufficient linked data was that the relative risk of death or length of stay for infection from an MDR organism was assumed to be equal to the highest individual relative risk among the drugs assessed. This mostly likely underestimates the relative risk of MDR infections because fewer effective antibiotic options remain as resistance accumulates. In light of data sparsity, we made several additional methodological assumptions (appendix pp 17–60). Despite scarcity, our estimates are informed by data from all regions (figure 7, table 1). These figures, and the appendix (pp 26–30, 43–44, 52–53, and 56, which provides out-of-sample model validation), suggest that our modelled estimates fit the data, where available.

Our analysis echoes that of another paper in highlighting critical AMR data gaps in several regions.^65^ There are many well described barriers to good-quality clinical bacteriology in LMICs, and proper quality assurance and quality-control measures are crucial for quality care and accurate laboratory-based surveillance.^66^ Many lab-based surveillance systems are not linked to patient diagnoses or outcomes, limiting the inferences that are possible to obtain from such data. Selection bias in how samples get incorporated into surveillance systems; scarcity of laboratory facilities to test for AMR and other challenges in identifying AMR;^17^ insufficient data linking prevalence of resistance to infectious syndrome, underlying cause, and outcome; barriers to sharing data that have been collected; and other data-linking and data optimisation issues continue to complicate the assessment and interpretation of the results in many cases.

A second limitation of our study was the several potential sources of bias we noted when combining and standardising data from a wide variety of providers. Our estimates of the proportion of infections that were community acquired versus hospital acquired for lower respiratory and thorax infections and urinary tract infections were based on the coding of data from multiple causes of death and hospital discharge data. This approach could lead to misclassification, since the criteria used in this coding are not strictly related to community versus hospital acquisition. In future iterations of the project, we hope to improve on the identification of community-acquired and hospital-acquired infections.

Additionally, no universal laboratory standard exists to demarcate resistance versus susceptibility, and we often had to defer to laboratory interpretation to classify the isolates in our data, resulting in heterogeneous classification. Whenever possible, we classified resistance using the most recent CLSI guidelines based on the minimum inhibitory concentrations provided in the data; however, CLSI breakpoints have changed over time, and many datasets did not provide sufficient detail to allow for retrospective reanalysis of the data.^67^

Finally, there is a possibility of selection bias in passive microbial surveillance data, particularly if cultures are not routinely drawn. It might be that, in certain locations, cultures are drawn only if a patient does not respond to initial antibiotic therapy, which might lead to an overestimate of the prevalence of resistance. Furthermore, in LMICs, hospital microbial data might skew towards more urban populations or more severe disease, which might not be representative of the broader population. We also received various data from tertiary care facilities; although we adjusted for bias in the prevalence of resistance data collected from these sources, much of our data came from mixed-classification or unclassifiable facilities, so it is possible that we did not fully adjust for all potential tertiary bias. Further limitations specific to each modelling component can be found in the appendix (pp 119–20).

Despite these limitations, this study is the most comprehensive analysis of bacterial AMR burden to date, reflecting the best and widest range of available data and the use of models that have been tested and iterated over years of GBD analysis to incorporate disparate data sources. Individually, these sources do not fully address the burden of AMR but, when used collectively, they provide a more complete estimate with robust geographical coverage. To our knowledge, our study is the first to report burden both attributable to and associated with AMR for an extensive list of pathogens and pathogen–drug combinations, with global and regional findings based on estimates for 204 countries and territories. In the future, these estimates could be used to better inform treatment guidelines. The dominant bacterial pathogens for a given infectious syndrome and the antibiotics that would offer effective treatment could be identified using the data for this study, which, along with estimates of pathogen–drug burden, could be used to inform empirical syndromic treatment guidelines tailored to a specific location.

Our analysis clearly shows that bacterial AMR is a major global health problem. It poses the largest threat to human health in sub-Saharan Africa and south Asia, but it is important in all regions. A diverse set of pathogens are involved, and resistance is high for multiple classes of essential agents, including beta-lactams and fluoroquinolones. Efforts to build laboratory infrastructure are paramount to addressing the large and universal burden of AMR, by improving the management of individual patients and the quality of data in local and global AMR surveillance and bolstering national AMR plans of action. Enhanced infrastructure would also expand AMR research in the future to evaluate the indirect effects of AMR, such as the effect of AMR on perioperative prophylaxis or prophylaxis of infections in transplant recipients, the effects of AMR on transmission, the impact and prevalence of specific variants evaluated through genotypic epidemiology, and more. Identifying strategies that can work to reduce the burden of bacterial AMR—either across a wide range of settings or those that are specifically tailored to the resources available and leading pathogen–drug combinations in a particular setting—is an urgent priority.

## Data sharing

Citations for the data used in the study can be accessed from the Global Health Data Exchange AMR website. Access to the data are also provided as data use agreements permit.

## Declaration of interests

E Ashley reports that Lao-Oxford-Mahosot Hospital—Wellcome Trust Research Unit received financial support from the Global Research on Antimicrobial Resistance Project (GRAM) to extract and prepare data for the present manuscript. J Bielicki reports grants from the European and Developing Countries Clinical Trials Partnership, Horizon 2020, and Swiss National Science Foundation, and a contract from the National Institute for Health Research (NIHR), outside of the submitted work; and consulting fees from Shionogi and Sandoz and speaking fees from Pfizer and Sandoz, outside the submitted work. C Carvalheiro reports financial support for the present manuscript from the Global Antibiotic Research and Development Partnership, who provided payments to Fundação de Apoio ao Ensino, Pesquisa e Assistência of the Clinical Hospital of the Faculty of Medicine of Ribeirão Preto, University of São Paulo, Brazil. S Dunachie reports financial support for the present manuscript from UL Flemming Fund at the Department of Health and Social Care, the Bill & Melinda Gates Foundation, and the Wellcome Trust; a paid membership role for the Wellcome Trust Vaccines Advisory Selection Panel Vaccines and AMR in November, 2019; and an unpaid role as an expert adviser to WHO's Global Antimicrobrial Resistance Surveillance System, from November, 2018 onwards, outside the submitted work. A Haselbeck reports support for the present manuscript from the Bill & Melinda Gates Foundation (OPP1205877). C Lim was supported by the Wellcome Trust Training Fellowship between September, 2017 and March 2020 (206736/Z/17/Z), outside the submitted work. M Mussi-Pinhata reports support for the present manuscript from research from grant funding from Fondazione PENTA—Onlus and the Clinical Trial Manager Global Antibiotic R&D Partnership (GARDP). P Newton reports support for the present manuscript from research grant funding from the Wellcome Trust. J Robotham is a member of the UK Government Advisory Committee on Antimicrobial Prescribing Resistance and Healthcare Associated Infections, outside the submitted work. J Scott reports that the London School of Hygiene & Tropical Medicine (LSHTM) received financial support from Emory University to support CHAMPS projects in Ethiopia for the present manuscript; reports a paid fellowship from the Wellcome Trust, research grants from Gavi, the Vaccine Alliance, and NIHR paid to LSHTM, and an African research leader fellowship paid to LSHTM by the Medical Research Council, outside the submitted work; and reports being a member of the data safety and monitoring board for PATH Vaccines Solutions for SII PCV10 in The Gambia. J Sifuentes-Osornio reports financial support from Oxford University for the present manuscript; research grants from Oxford, CONACYT, Sanofi, and Novartis, outside of the study; consulting fees from Senosiain and speaker fees from Merck, outside of the study; and membership of the Sanofi advisory board of COVID-19 Vaccine Development, which is currently in progress, outside of the study. A J Stewardson reports grants or contracts from Merck, Sharp, & Dohme paid to Monash University, Melbourne, outside of the study. P Turner reports grants, consulting fees, and support for attending meetings or travel from Wellcome Trust, outside the study. H van Doorn reports grants or contracts from the University of Oxford and is the principal investigator for the Fleming Fund pilot grant; and he is a board member of Wellcome Trust's Surveillance and Epidemiology of Drug Resistant Infections. T Walsh reports financial support from the Bill & Melinda Gates Foundation for the BARNARDS (neonatal sepsis and mortality) study for the present manuscript. All other authors declare no competing interests.
