
JOURNAL INFORMATION
==============================
NLM Title Abbreviation: Lancet
Journal ID: Lancet
NLM Journal ID: 2985213R

Lancet (London, England)

ISSN: 0140-6736
EISSN: 1474-547X

ARTICLE INFORMATION
==============================
PMCID: PMC9547118
PMID: 36183727
DOI: 10.1016/S0140-6736(21)02653-2
Article ID: S0140-6736(21)02653-2
Article version: 1
Subjects: Department of Error

Department of Error

Print publication date: 2022 Oct 1
Volume: 400
Issue: 10358
First page: 1102
Last page: 1102
Copyright: © 2021 The Author(s). Published by Elsevier Ltd.
Copyright year: 2022
Copyright holder: Elsevier Ltd
License: This is an open access article under the CC BY license (http://creativecommons.org/licenses/by/4.0/).
License URL: https://creativecommons.org/licenses/by/4.0/

Erratum for: Global burden of bacterial antimicrobial resistance in 2019: a systematic analysis 399 10325 19 1 2022 629 655 Lancet (London, England) 10.1016/S0140-6736(21)02724-0 PMC8841637 35065702

==============================
Antimicrobial Resistance Collaborators. Global burden of bacterial antimicrobial resistance in 2019: a systematic analysis. Lancet 2022; 399: 629–55—In this Article, the Antimicrobial Resistance Collaborators list and affiliations have been updated. These corrections have been made to the online version as of Sept 29, 2022.

