# Supplementary material for: Global burden of bacterial antimicrobial resistance in 2019: a systematic analysis
Source: Lancet. Author manuscript; Available in PMC 2022 Feb 21. (PMC8841637; doi:10.1016/S0140-6736(21)02724-0)

THE LANCET

Supplementary appendix

This appendix formed part of the original submission and has been peer reviewed. We post it as supplied by the authors.

Supplement to: Antimicrobial Resistance Collaborators. Global burden of bacterial antimicrobial resistance in 2019: a systematic analysis. *Lancet* 2022; published online Jan 20. https://doi.org/10.1016/S0140-6736(21)02724-0.

Appendix 1: supplementary methods and results to “Global burden of bacterial antimicrobial resistance in 2019”

This appendix provides further methodological details and supplementary results for “Global burden of bacterial antimicrobial resistance in 2019”.

Table of contents

List of tables and figures 4

Section 1: List of abbreviations 6

Section 2: Data sources....8

Section 2.1: Multiple causes of death and vital registration (MCoD-VR) 8

Section 2.2: Hospital discharge 8

Section 2.3: Microbial data with outcome....9

Section 2.4: Microbial data without outcome....10

Section 2.5: Literature studies 11

Section 2.6: Single drug-resistance profiles 14

Section 2.7: Pharmaceutical sales and antibiotic use 15

Section 2.8: Mortality surveillance 15

Section 2.9: Linkage (mortality only) 15

Section 3: Summary of GBD 2019 estimation process....16

Section 3.1: GBD 2019 cause of death estimation process 16

Section 3.2: GBD 2019 non-fatal estimation process....16

Section 4: Deaths where infection plays a role and infectious syndrome estimation 17

Section 4.1: Input data....17

Section 4.2: Data processing 18

Section 4.3: Mapping the data....18

Section 4.4: Intermediate cause and infectious syndrome mapping hierarchy....18

Section 4.5: First pathway: deaths where infection plays a role....21

Section 4.6: Second pathway: fraction of deaths where infection plays a role by infectious syndrome in each GBD cause…………… 23

Section 4.7: Model validation 25

Section 5: Case fatality ratios 31

Section 5.1: Input data....31

Section 5.2: Data processing 31

Section 5.3: Modelling overview 31

Section 5.4 Modelling framework....32

Section 5.5 Predictions and uncertainty 34

Section 6: Pathogen distribution....34

Section 6.1: Input data....34

Section 6.2: Data processing 35

Section 6.3: Modelling framework....39

Section 6.4: Exceptions and special handling 42

Section 6.5: Model validation 43

Section 7: Prevalence of resistance..44
Section 7.1: Input data..44
Section 7.2: Data processing..45
Section 7.3: Modelling framework..48
Section 7.4: Covariates..49
Section 7.5: Resistance profiles..49
Section 7.6: Model validation..51
Section 8: Relative risk..54
Section 8.1 Input data..54
Section 8.2: Data processing..54
Section 8.3 Modelling overview..54
Section 8.4 Model validation..55
Section 9: Counterfactuals and AMR estimation..56
Section 9.1: Estimating AMR burden with counterfactual of no infection..56
Section 9.2: Estimating AMR burden with counterfactual of infection with susceptible organism..58
Section 9.3: Excluded combinations..59
Section 10. Analysis of total AMR burden decomposed by infection and resistance..60
Section 11. GATHER compliance..61
Section 12: References..61
Section 13: Author contributions..65
Managing the estimation or publications process..65
Writing the first draft of the manuscript..65
Primary responsibility for applying analytical methods to produce estimates..65
Primary responsibility for seeking, cataloguing, extracting, or cleaning data; designing or coding figures and tables………….. 65
Providing data or critical feedback on data sources..65
Developing methods or computational machinery..65
Providing critical feedback on methods or results..65
Drafting the work or revising it critically for important intellectual content..66
Extracting, cleaning, or cataloging data; designing or coding figures and tables..66
Managing the overall research enterprise..66
Section 14: Appendix tables and figures..67

List of tables and figures

Table S1. GBD cause hierarchy with levels 67

Table S2: List of International Classification of Diseases (ICD) codes mapped to the Global Burden of Disease cause list for causes of death 76

Table S3. List of infectious syndromes mapped to International Classification of Diseases (ICD) codes 84

Table S4. Summary of case fatality ratio data 87

Table S5. Case fatality ratio modelling framework by pathogen and syndrome 88

Table S6. Summary of pathogen distribution data....89

Table S7. Summary of prevalence of resistance data 90

Table S8. Covariates for each core pathogen–drug combination (excluding 3GC-resistant N. gonorrhoeae) 92

Table S9. Summary of relative risk data....94

Table S10. Relative risk estimates for sterile sources of specimen across 86 pathogen–drug combinations 96

Table S11. Decomposition of fatal antimicrobial resistance burden by GBD region, 2019 97

Table S12. GATHER checklist....98

Table S13. GBD location hierarchy with levels 100

Table S14. Deaths and DALYs (in counts and age-specific rates) associated with and attributable to bacterial antimicrobial resistance by pathogen–drug combination; by age group; 2019; central Europe, eastern Europe, and central Asia super-region....106

Table S15. Deaths and DALYs (in counts and age-specific rates) associated with and attributable to bacterial antimicrobial resistance by pathogen–drug combination, by age group, 2019, high-income super-region 108

Table S16. Deaths and DALYs (in counts and age-specific rates) associated with and attributable to bacterial antimicrobial resistance by pathogen–drug combination, by age group, 2019, Latin America and the Caribbean super-region....109

Table S17. Deaths and DALYs (in counts and age-specific rates) associated with and attributable to bacterial antimicrobial resistance by pathogen–drug combination, by age group, 2019, north Africa and the Middle East super-region....111

Table S18. Deaths and DALYs (in counts and age-specific rates) associated with and attributable to bacterial antimicrobial resistance by pathogen–drug combination, by age group, 2019, south Asia super-region 113

Table S19. Deaths and DALYs (in counts and age-specific rates) associated with and attributable to bacterial antimicrobial resistance by pathogen–drug combination; by age group; 2019; southeast Asia, east Asia, and Oceania super-region....115

Table S20. Global deaths and DALYs (in counts and age-specific rates) associated with and attributable to bacterial antimicrobial resistance by pathogen–drug combination; by age group; 2019; sub-Saharan Africa super-region....117

Table S21. Limitations of each primary modelling component....119

Table S22. Global deaths and DALYs (in counts and all-age rates) associated with and attributable to bacterial antimicrobial resistance by pathogen–drug combination, 2019....121

Figure S1. Flowchart of antimicrobial resistance fatal and non-fatal estimation steps....123

Figure S2. All-age rate of DALYs attributable to and associated with bacterial antimicrobial resistance by GBD region, 2019....124

Figure S3. Global DALYs (in counts) attributable to and associated with bacterial antimicrobial resistance by infectious syndrome, 2019 125

Figure S4. Global DALYs (in counts) attributable to and associated with bacterial antimicrobial resistance by pathogen, 2019 126

Figure S5. Pathogen-attributable fraction of DALYs attributable to (A) and associated with (B) bacterial antimicrobial resistance for the six leading pathogens by GBD super-region, 2019 127

Figure S6. Global DALYs (in counts) attributable to bacterial antimicrobial resistance by pathogen–drug combination, 2019 129

Figure S7. Vital Registration and Verbal Autopsy data availability by country and territory, 1980−2018 130

Section 1: List of abbreviations

Abbreviation	Full phrase
AGAR	Australian Group on Antimicrobial Resistance
AHC	Angkor Hospital for Children
AMASS	AutoMated tool for Antimicrobial resistance Surveillance System
AMR	antimicrobial resistance
APUA	Alliance for the Prudent Use of Antibiotics
ARSP	Antimicrobial Resistance Surveillance Program
ATLAS	Antimicrobial Testing Leadership and Surveillance
AURA	Antimicrobial Use and Resistance in Australia
AWARE	Assessing Worldwide Antimicrobial Resistance Evaluation
BARNARDS	Burden of Antibiotic Resistance in Neonates from Developing Societies
BD	Becton, Dickinson, and Company
BSI	bloodstream infections
CAESAR	Central Asian and European Surveillance of Antimicrobial Resistance
CAI	community-acquired infection
CDC	Centers for Disease Control and Prevention
CFR	case fatality ratio
CHAIN	Childhood Acute Illness and Nutrition
CHAMPS	Child Health and Mortality Prevention Surveillance
cIAI	complicated intra-abdominal infection
COMRU	Cambodia Oxford Medical Research Unit
CTMRF	CHILDS Trust Medical Research Foundation
cUTI	complicated urinary tract infection
DALYs	Disability-adjusted life-years
DHS	Demographic Health Surveys
EARS-Net	European Antimicrobial Resistance Surveillance Network
ECDC	European Centre for Disease Prevention and Control
GAM	generalised additive models
GBD	Global Burden of Diseases, Injuries, and Risk Factors Study
GBS	group B *Streptococcus*
GLASS	Global Antimicrobial Resistance Surveillance System

GLM	generalised linear model
GPR	Gaussian process regression
HAI	hospital-acquired infection
HAQ Index	Healthcare Access and Quality Index
HHS	U.S. Department of Health and Human Services
ICD	International Classification of Diseases
ICU	intensive care unit
INFORM	International Network for Optimal Resistance Monitoring
INICC	International Nosocomial Infection Control Consortium
iNTS	invasive non-typhoidal Salmonella
IORD	Infections in Oxfordshire Research Database
IQVIA	IMS Health and Quintiles
JANIS	Japan Nosocomial Infections Surveillance
KEMRI	Kenya Medical Research Institute
LRI	lower respiratory infection
MCoD	multiple causes of death data
MEPCO	multinomial estimation of partial and composite observations
MICS	Multiple Indicators Cluster Surveys
MITS	minimally invasive tissue sampling
MR-BRT	meta-regression—Bayesian, regularised, trimmed
MRC	Medical Research Council
NARMS	National Antimicrobial Resistance Monitoring System for Enteric Bacteria
NICD	National Institute for Communicable Diseases
OUCRU	Oxford University Clinical Research Unit
PPS HAI	Point Prevalence Survey on Nosocomial Infections and Antibiotic Use
PRISMA	Preferred Reporting Items for Systematic Reviews and Meta-Analyses
SDI	Socio-demographic Index
SEV	summary exposure value
SGUL-GARPEC	St. George's Hospital, University of London - Global Antimicrobial Resistance, Prescribing and Efficacy Among Neonates and Children
SOAR	Survey on Antibiotic Resistance
ST-GPR	spatiotemporal Gaussian process regression

TB	tuberculosis
TESSy	The European Surveillance System
TEST	Tigecycline Evaluation Surveillance Trial
TSAP	Typhoid Fever Surveillance in Africa Program
UI	uncertainty interval
UPCH	Cayetano Heredia University
USDA	U.S. Department of Agriculture
UTI	urinary tract infection
VR	vital registration
WHO	World Health Organization
WRP	Walter Reed Project
YLDs	years lived with disability
YLLs	years of life lost

Section 2: Data sources

The data used for this study can be categorised into the following types: multiple causes of death (MCoD), hospital discharge, linkage, mortality surveillance, literature reviews, , microbial, single drug-resistance profiles, pharmaceutical sales, and antibiotic use data; as well as estimates from the Global Burden of Diseases, Injuries, and Risk Factors Study (GBD) 2019.[1] More detailed information on data inputs are available at http://ghdx.healthdata.org/record/ihme-data/global-bacterial-antimicrobial-resistance-burden-estimates-2019.

Section 2.1: Multiple causes of death and vital registration (MCoD-VR)

Multiple cause of death (MCoD) data is a type of vital registration obtained from death certificates that contain the underlying cause of death, intermediate and immediate causes of death, and contributing conditions. MCoD data differ from other vital registration (VR) sources, because many countries have VR systems that only document the underlying cause of death. MCoD data were used in the sepsis, infectious syndrome, and pathogen distribution component models and data processing, and modelling methods can be found in sections 4 and 6. MCoD-VR data came from the following sources.

- United States National Vital Statistics System
- Brazil Mortality Information System
- National Institute of Statistics (Italy)
- Statistics South Africa
- National Institute of Statistics and Geography (Mexico)
- National Administrative Department of Statistics (Colombia)
- Taiwan Ministry of Health and Welfare

Section 2.2: Hospital discharge

Hospital admissions and discharge data are data sources collected from inpatient hospital and other clinical settings. These data include information on the primary and secondary diagnosis for each patient, as applicable, and were obtained from the sources listed below. Hospital data were used in the sepsis, infectious syndrome, pathogen

distribution, and case fatality ratio component models and data processing, and modelling methods can be found in sections 4–6.

- USA National Hospital Discharge Survey
- USA State Inpatient Databases
- Brazil Hospital Information System
- Italy Hospital Inpatient Discharges
- Sistema Automatizado de Egresos Hospitalarios (Mexico)
- Austria Hospital Inpatient Discharges
- New Zealand National Minimum Dataset
- Canada Discharge Abstract Database

Section 2.3: Microbial data with outcome

Microbial data are data sources from hospital and lab networks that collect pathogen cultures from patients. The cultures are tested for both pathogen and the pathogen's resistance to antibiotics. The culture results are linked to patient outcome, diagnoses, or both. Microbial data without these outcomes or diagnoses are listed in section 2.4. These data also include the specimen from which the pathogen was isolated and whether the infection was community- or hospital-acquired, if available. When hospital versus community acquisition was not specified, we used the difference between admission or diagnosis date and the specimen collection date, and if 48 hours or fewer had passed between those two dates, then the infection was assumed to be community-acquired. We assumed the infection was hospital-acquired when more than 48 hours had passed, consistent with CDC/National Healthcare Safety Network guidelines.[2] Microbial data with outcome were used in the case fatality ratio, pathogen distribution, prevalence of resistance, and relative risk component models and data processing, and modelling methods can be found in sections 5–8. Microbiology data types, with outcome and diagnoses were obtained from the sources below.

- **USA Becton, Dickinson, and Co. (BD) Insights, Research and Analytics Database microbiology test and in-patient hospital data:** data procured by BD via MedMined. Covers a range of regions in the United States from 2011 to 2017.
- **UK Infections in Oxfordshire Research Database (IORD):** patient microbiology and episodes data from Oxford University Hospitals NHS Foundation Trust.
- **International Nosocomial Infection Control Consortium (INICC) surveillance online system:** data from the INICC data collection software. ICU patient microbiology and hospital data from 50 countries across Latin America, Asia, the Middle East, eastern Europe, and Africa from 2009 to 2020.
- **Bulgaria antimicrobial resistance data**: Medical University of Varna in Varna, Bulgaria. Covers 2014–2020.
- **St. George's Hospital, University of London - Global Antimicrobial Resistance, Prescribing and Efficacy Among Neonates and Children (SGUL-GARPEC) Project bloodstream infection data:** Penta-sponsored global surveillance network focusing on neonatal and paediatric antimicrobial resistance and the organisms causing blood stream infections.
- **Burden of Antibiotic Resistance in Neonates from Developing Societies (BARNARDS)**: BARNARDS includes locations in Nigeria, South Africa, Pakistan, Rwanda, Bangladesh, Ethiopia and India from 2015 to 2018.
- **Lao-Oxford-Mahosot Hospital-Wellcome Trust Research Unit (LOMWRU). Children and adults with fever, inpatient admissions:** information from children and adults with fever who were admitted as inpatients between 1996 and 2019 to Mahosot Hospital, Vientiane, Laos. Microbial analysis was carried out by the Microbiology Laboratory at Mahosot Hospital.
- **Kumasi Centre for Collaborative Research in Tropical Medicine (KCCR), Kumasi, Ghana together with the Bernhard Nocht Institute for Tropical Medicine. Data on children and adults admitted in hospital with fever:** information from children and adults with fever admitted as inpatients at the Bernhard Nocht Institute for Tropical Medicine in Ghana between 2007 and 2015.
- **Vietnam Hospital for Tropical Diseases, Ho Chi Minh City. Hospital-acquired infections in ICU patients:** prospective observational study at the Oxford University Clinical Research Unit (OUCRU) in the

Ho Chi Minh City Hospital for Tropical Diseases, Vietnam from November 2014 to January 2016 to assess the ICU-acquired colonisation and infections among adult patients with more than 48 hours of ICU stay.

- **Medical Research Council (MRC) Unit The Gambia. Diagnostic antimicrobial susceptibility testing:** information on hospital admission and discharge, pathogens cultured, resistance susceptibility test and antibiotics prescribed between 2005 and 2015 from the MCR Unit The Gambia, now part of the London School of Hygiene and Tropical Medicine.
- **Cambodia Oxford Medical Research Unit (COMRU) and Angkor Hospital for Children (AHC). Suspected invasive bacterial infection hospitalisations:** reports children aged 0–21 years who were hospitalised with suspected invasive bacterial infection between 2015 and 2018.
- **Taiwan hospital-acquired infections and outcomes:** infectious disease surveillance linked to vital registration from Taiwan (province of China).
- **Childhood Acute Illness and Nutrition (CHAIN) Network antimicrobial resistance data:** CHAIN Network study informs on hospitalised children under 2 years old with acute illness in Bangladesh, Burkina Faso, Pakistan, Kenya, Malawi, and Uganda.
- **Lima, Peru Cayetano Heredia University (UPCH) antimicrobial resistance data:** data from UPCH hospital sites across Lima, Peru with discharge disposition for infectious pulmonary disease
- **Jordan King Abdulla University Hospital culture and sensitivity tests:** information on inpatients at the King Abdulla University Hospital in 2020 part of the Jordan University of Science and Technology.
- **Iran antimicrobial resistance in burn patients and identified in blood, cerebrospinal fluid, and urine cultures:** data from inpatients across different hospital sites in Iran between 2016 and 2020.
- **Dhaka, Bangladesh Bangabandhu Sheik Mujib Medical University hospital inpatient data:** data from 201 inpatients in 2017 at the Bangabandhu Sheikh Mujib Medical University, Dhaka, Bangladesh.
- **Chiangrai Prachanukroh Hospital, Chiangrai Clinical Research Unit and Mahidol Oxford Tropical Medicine Research Unit:** data from inpatients with positive cultures at the Chiangrai Prachanukroh Hospital from 2017 to 2019.
- **KEMRI/US Army Medical Research Directorate**
- **Chennai, India Kanchi Kamakoti CHILDS Trust Medical Research Foundation (CTMRF) hospital inpatient data**

Section 2.4: Microbial data without outcome

Microbial data were also obtained from laboratories, which do not necessarily link to patients' hospital records nor information on their discharge disposition. These sources report specimen or site of infection, pathogens isolated, antimicrobial susceptibility tests, age and gender and other demographic characteristics. This information proved useful to inform pathogen distribution and prevalence of resistance component models and data processing, and modelling methods can be found in sections 6 and 7. Microbial data without outcome and diagnoses were obtained from the sources below.

- **SENTRY:** SENTRY Antimicrobial Surveillance Program established by JMI Labs in 1997. Sites are in the USA, Europe, Latin America, parts of Asia, and the Western Pacific
- **Germany National Point Prevalence Survey on Nosocomial Infections and Antibiotic Use (PPS HAI):** Point Prevalence Survey for 2016 data reporting the pathogen distribution for hospital-acquired infections.
- **Madagascar – Fondation Merieux:** data collected from inpatients with positive culture admitted in three hospital sites in Madagascar, funded by Fondation Merieux.
- **AMASS:** data collected in an automated tool by Oxford Tropical Network Research Units.
- **The European Surveillance System (TESSy):** managed by the European Centre for Disease Prevention and Control (ECDC), provided data from the following surveillance systems:
    - European Antimicrobial Resistance Surveillance Network (EARS-Net)
    - Food-and Waterborne Diseases and Zoonoses Surveillance Network.
    - Invasive Pneumococcal Disease Surveillance Network, including discharge disposition.
    - Gonococcal Antimicrobial Surveillance Programme.

- Healthcare Associated Infections Surveillance Network (ICU protocol), including discharge disposition.
- European Tuberculosis Surveillance Network
- European Surveillance of Antimicrobial Consumption Network

For the European Union/European Economic Area (EU/EEA), data were obtained from the Euroepan Surveillance System (TESSy) as provided by Austria, Belgium, Croatia, Cyprus, Czechia, Denmark, Estonia, Finland, France, Germany, Greece, Hungary, Iceland, Ireland, Italy, Latvia, Luxembourg, Malta, Netherlands, Norway, Poland, Portugal, Romania, Slovakia, Slovenia, Spain, Sweden, and the United Kingdom, and released by the European Centre for Disease Prevention and Control (ECDC).

- **Pfizer ATLAS Programme:** the Antimicrobial Testing Leadership and Surveillance (ATLAS) database includes the Tigecycline Evaluation Surveillance Trial (TEST), the Assessing Worldwide Antimicrobial Resistance Evaluation (AWARE) and the International Network for Optimal Resistance Monitoring (INFORM) programs. The study spans in coverage across more than 70 countries between 2004 and 2017.
- **Malawi Queen Elizabeth Hospital microbiology tests of blood specimens:** microbiology tests of blood specimens from inpatients at the Queen Elizabeth Hospital in Malawi from 1998 to 2016, part of the Institute of Infection and Global Health, University of Liverpool in collaboration with the Malawi-Liverpool-Wellcome Trust and the Wellcome Trust Sanger Institute.
- **Central African Republic National Laboratory of Clinical Biology and Public Health:** data collected by the Laboratoire National de Biologie Clinique et de Sante Publique in Central African Republic between 2017 and 2020.
- **The Ethiopian AMR surveillance:** conducted from July 2018 to July 2020 across sentinel surveillance sites and the National AMR Surveillance Coordinating Centre for the Ethiopian Public Health Institute.
- **Lancet Labs:** data obtained from Lancet Laboratories, a network of private laboratories across different sites in Africa.
- **The Typhoid Fever Surveillance in Africa Program (TSAP):** was established by the International Vaccine Institute to obtain comparable incidence data on typhoid fever and invasive non-typhoidal Salmonella disease in Ghana, Burkina Faso, Ethiopia, Guinea Bissau, Kenya, Madagascar, Senegal, South Africa, Sudan, and Tanzania.
- **Invasive *Salmonella* infections at multiple surveillance sites in the Democratic Republic of the Congo study:** data published as part of the study on invasive *Salmonella* infections at multiple surveillance sites in the Democratic Republic of the Congo between 2011 and 2014.
- **Suva, Fiji Colonial War Memorial Hospital:** Information on sequential *S. aureus* and Enterobacterial bloodstream infections at the Colonial War Memorial Hospital (analysis by Monash University) in Suva, Fiji, between 21 July 2020 and 29 October 2020.
- **World Health Organization (WHO) Global Tuberculosis Programme**
- **Germany EARS-Net surveillance data 2017–2018**
- **WHO Meningitis surveillance:** sentinel hospital surveillance of suspected meningitis cases among children under 5 years old and positive cultures, provided by the World Health Organization (WHO) Global Rotavirus, Invasive Bacterial Vaccine Preventable Diseases Surveillance Network Collaboration from 2008 to 2020.
- **United States Active Bacterial Core Surveillance (ABCs) Reports:** case reports on healthcare-associated Infections and community interface infections from the Emerging Infections Program Network coordinated by the Center for Disease Control and Prevention (CDC).

Section 2.5: Literature studies

We conducted literature searches to obtain input data for the following components in the analysis: maternal and neonatal sepsis aetiology, lower respiratory infections (LRIs) aetiology, urinary tract infections (UTIs) aetiology, skin infections aetiology, meningitis aetiology and case fatality, intra-abdominal infection aetiology, bone and joint infections aetiology, prevalence of resistance, relative risk and length of stay. Literature searches were performed on

PubMed using the following search strings, and extracted studies covered the time range 1980–2020. The search string for these searches can be found below. Literature was used in the case fatality ratio, pathogen distribution, prevalence of resistance and relative risk component models and data processing, and modelling methods can be found in sections 5–8. Literature studies were also used as input into the modelling of the antibiotic usage covariate.[3]

Section 2.5.1: Maternal sepsis, neonatal sepsis, and LRI aetiology

Aetiology terms, combined with OR:

- Infection (Infect*)
- Microbiology (Microbiolog*
- Aetiology (Aetiolog*)
- Etiology (Etiolog*)
- Virology (Virolog*)
- Bacteriology (Bacteriolog*)
- Fungus (fung*)

AND

Syndrome terms, combined with OR:
Maternal Sepsis

- puerperal sepsis (puerper* sepsis)
- maternal sepsis (matern* sepsis)
- puerperal septicaemia (puerper* septicaemia, American spelling too - septicemia)
- maternal septicaemia (matern* septicaemia, American spelling too - septicemia)
- puerperal infection (puerper* infection)
- maternal infection (matern* infection)
- puerperal bacteraemia (puerper* bacteraemia, American spelling too - bacteremia)
- maternal bacteraemia (matern* bacteraemia, American spelling too - bacteremia)

Neonatal Sepsis

- Neonatal sepsis (Neonat* sepsis within 3 or 5 words of each other)
- Neonatal septicaemia (Neonat* septicaemia within 3 or 5 words of each other, American spelling too - septicemia)
- Infant sepsis (Infant* sepsis)
- Infant septicaemia (Infant* septicaemia, American spelling too - septicemia)
- Neonatal bacteraemia (Neonat* bacteraemia, American spelling too - bacteremia)
- Infant bacteraemia (Infant* bacteraemia, American spelling too - bacteremia)

Lower respiratory infections

- LRI
- Lower respiratory infection
- LRTI
- Lower respiratory tract infection
- Pneumonia

Section 2.5.2: Urinary tract infections aetiology

("complicated"[Title/Abstract] OR "uncomplicated"[Title/Abstract]) AND (("Cystitis/etiology"[majr:noexp] OR "Cystitis/microbiology"[majr:noexp]) OR ("Pyelonephritis/etiology"[marj:noexp] OR "Pyelonephritis/microbiology"[majr:noexp]) OR ("Urinary Tract Infections/etiology"[majr:noexp] OR "Urinary Tract Infections/microbiology"[majr:noexp])) OR ("Urinary tract infections"[tiab] AND ("etiology"[tiab] OR "microbiology"[tiab]))

Section 2.5.3: Skin infections aetiology

(("Cellulitis/epidemiology"[majr:noexp] OR "Cellulitis/etiology"[majr:noexp] OR "Cellulitis/microbiology"[majr:noexp]) OR ("Pyoderma/epidemiology"[majr:noexp] OR "Pyoderma/etiology"[marj:noexp] OR "Pyoderma/microbiology"[majr:noexp]) OR

"Pressure Ulcer/microbiology"[majr:noexp])

Section 2.5.4: Intra-abdominal infection aetiology
(("Peritonitis/epidemiology"[majr:noexp] OR "Peritonitis /etiology"[majr:noexp] OR "Peritonitis /microbiology"[majr:noexp]) OR ("Intraabdominal infections/epidemiology"[majr:noexp] OR "Intraabdominal infections /etiology"[marj:noexp] OR "Intraabdominal infections /microbiology"[majr:noexp]) OR ("abdominal abscess/epidemiology"[majr:noexp] OR " abdominal abscess /etiology"[majr:noexp] OR "abdominal abscess/microbiology"[majr:noexp]))

Section 2.5.5: Bone and joint infections aetiology
("Osteomyelitis/etiology"[majr:noexp] OR "Osteomyelitis/microbiology"[majr:noexp] NOT 'chronic') OR ("Arthritis, infectious/etiology"[marj:noexp] OR "Arthritis, infectious/microbiology"[majr:noexp] NOT ‘lyme’)

Section 2.5.6: Meningitis infection aetiology
((meningitis[title]) AND (1990/05/01[PDat] : 2018/12/31[PDat]) AND ((etiolog*[title/abstract]) AND Humans[MeSH Terms])

Section 2.5.7: Relative risk studies for specific drug-bug combinations
("Acinetobacter baumannii"[MeSH Terms] AND "carbapenem resistance"[All Fields]) OR ("Acinetobacter baumannii"[MeSH Terms] AND "carbapenem resistant"[All Fields])

('Escherichia coli'[MeSH Terms] AND 'carbapenem resistance'[All Fields]) OR ('Escherichia coli'[MeSH Terms] AND 'carbapenem resistant'[All Fields])

('Escherichia coli'[MeSH Terms] AND 'fluoroquinolone resistance'[All Fields]) OR ('Escherichia coli'[MeSH Terms] AND 'fluoroquinolone resistant'[All Fields])

('Escherichia coli'[MeSH Terms] AND 'third generation cephalosporin'[All Fields]) OR ('Escherichia coli'[MeSH Terms] AND ESBL OR extended-spectrum beta lactamase'[All Fields])

('Klebsiella pneumoniae'[MeSH Terms] AND 'third generation cephalosporin'[All Fields]) OR ('Klebsiella pneumoniae'[MeSH Terms] AND 'ESBL OR extended-spectrum beta lactamase'[All Fields])

('Klebsiella pneumoniae'[MeSH Terms] AND 'carbapenem resistance'[All Fields]) OR ('Klebsiella pneumoniae'[MeSH Terms] AND 'carbapenem resistant'[All Fields])

('Streptococcus pneumoniae'[MeSH Terms] AND 'penicillin resistance'[All Fields]) OR ('Streptococcus pneumoniae'[MeSH Terms] AND 'penicillin resistant'[All Fields])

('Pseudomonas aeruginosa'[MeSH Terms] AND 'carbapenem resistant'[All Fields] AND 'mortality' [MeSH Terms]) OR ('Pseudomonas aeruginosa'[MeSH Terms] AND 'carbapenem resistant' AND 'mortality' [All Fields])

('Enterococcus faec*'[MeSH Terms] AND 'vancomycin-resistant'[All Fields])

("haemophilus influenzae"[MeSH Terms] AND ("penicillin resistance"[MeSH Terms] OR ("penicillin"[All Fields] AND "resistance"[All Fields]) OR "penicillin resistance"[All Fields])) AND ("mortality"[Subheading] OR "mortality"[All Fields] OR "mortality"[MeSH Terms])

("streptococcus agalactiae"[MeSH Terms] AND ("azithromycin resistance"[MeSH Terms] OR ("azithromycin "[All Fields] AND "resistance"[All Fields]) OR " azithromycin resistance"[All Fields] OR "penicillin resistance"[MeSH Terms] OR ("penicillin"[All Fields] AND "resistance"[All Fields]) OR "penicillin resistance"[All Fields] OR "clindamycin resistance"[MeSH Terms] OR ("clindamycin"[All Fields] AND "resistance"[All Fields]) OR "erythromycin resistance"[All Fields] OR "erythromycin resistance"[MeSH Terms] OR ("erythromycin"[All Fields] AND "resistance"[All Fields]) OR "clindamycin resistance"[All Fields]) AND ("mortality"[Subheading] OR "mortality"[All Fields] OR "mortality"[MeSH Terms])

Section 2.5.8: Prevalence of resistance for specific organisms

Medical Subject Heading (MeSH) terms with free text terms in the title and abstract fields for Escherichia coli, Klebsiella pneumoniae, Streptococcus pneumoniae and Staphylococcus aureus with the terms for antimicrobial drug resistance (resistan*, suscept*, surveil*, etc), limited from 1990 up to the search date. The search was undertaken on MEDLINE, Ovid Embase, Global Health, Cochrane Library.

Medical Subject Headings (MeSH) and free text terms for the pathogens of interest (e.g. S. Typhi, S. Paratyphi A, enteric fever) with terms for antimicrobial resistance (e.g. resistan*, suscept*, surveil*). The search was undertaken on MEDLINE, Ovid Embase, Global Health, Cochrane Library, Scopus, Web of Science-Core Collection and LILACS regional WHO database.

Medical Subject Heading (MeSH) terms with free text terms in the title and abstract fields for non-typhoidal Salmonella or Salmonellosis (non-typhi or nontyph or non-typh Salmonel…) with the terms for antimicrobial drug resistance (resistan*, suscept*, surveil*, etc) and invasive (blood stream infection, septicaemia etc), limited from 1990 up to the search date. The search was undertaken on MEDLINE, Ovid Embase, Global Health, Cochrane Library, Scopus, Web of Science-Core Collection and LILACS regional WHO.

Medical Subject Heading (MeSH) terms with free text terms in the title and abstract fields for Shigella or Shigellosis with the terms for antimicrobial drug resistance (resistan*, suscept*, surveil*, etc), limited from 1990 up to the search date. The search was undertaken on MEDLINE, Ovid Embase, Global Health, Cochrane Library, Scopus, Web of Science-Core Collection and LILACS regional WHO database.

Medical Subject Heading (MeSH) terms with free text terms in the title and abstract fields for Neisseria gonorrhoeae, with the terms for antimicrobial drug resistance (resistan*, suscept*, surveil*, etc), MDR, XDR, limited from 1990 up to the search date. The search was undertaken on MEDLINE, Ovid Embase, Global Health, Cochrane Library, Scopus, Web of Science-Core Collection and LILACS regional WHO database.

Section 2.6: Single drug-resistance profiles

Data sources used to inform single drug resistance profiles were obtained from surveillance networks and aggregated reports where the full antibiogram of a pathogen for all drugs tested is not reported. Data from these sources generally do not include any individual records linked to a patient outcome. They are used to inform current and past resistance trends for specific pathogen–drug combinations. Single drug resistance data were used in the prevalence of resistance component model and data processing, and modelling methods can be found in section 7. The data sources for single drug resistance profiles were obtained from the sources below.

- **GLASS:** Global Antimicrobial Resistance Surveillance System by WHO
- **CAESAR:** Central Asian and European Surveillance of Antimicrobial Resistance (CAESAR) is a network of national AMR surveillance systems and includes 19 countries in the WHO European Region that are not part of EARS-Net.
- **Japan Nosocomial Infections Surveillance (JANIS):** is a national surveillance program designed to provide basic information on the incidence and prevalence of nosocomial infections and antimicrobial-resistant bacteria in Japanese medical settings. Data available from 2013.
- **NARMS:** The National Antimicrobial Resistance Monitoring System for Enteric Bacteria (NARMS) is a collaboration of agencies within The U.S. Department of Health and Human Services (HHS) (FDA and CDC) and the U.S. Department of Agriculture (USDA). It tracks enteric bacteria and selected animal pathogens and their resistance to antimicrobials, and data is available from 1997 onwards.
- **SOAR:** Survey on Antibiotic Resistance (SOAR) sponsored by GSK.
- **ReLAVRA and SIREVA:** The Latin American Network for Antimicrobial Resistance Surveillance (ReLAVRA by its Spanish acronym) and the Serotype and Antimicrobial Resistance Surveillance Program (SIREVA by its English acronym) which are coordinated by the Pan-American Health Organization (WHO/PAHO)
- **SMART:** Study for Monitoring Antimicrobial Resistance Trends which monitors complicated intra-abdominal infections (cIAIs), complicated urinary tract infections (cUTIs) and respiratory infections worldwide, funded by Merck & Co.

- **South Africa National Institute for Communicable Diseases (NICD):** Aggregated data from South Africa's AMR surveillance in public healthcare centres which is submitted to the GLASS.
- **Surveillance on Invasive Pulmonary Disease by the New Zealand Public Health Action.**
- **Surveillance of Antimicrobial Resistance in Hospital Acquired Infection by Kendokteran Laboratorium, Indonesia**
- **Alliance for the Prudent Use of Antibiotics (APUA), Nepal**
- **Hospital Civil de Guadalajara Fray Antonio Alcalde, Mexico**
- **Australian Group on Antimicrobial Resistance (AGAR)**
- **Antimicrobial Resistance Surveillance Program (ARSP)**
- **Antimicrobial Use and Resistance in Australia (AURA)**
- **Australian Government Department of Health**
- **Canadian Antimicrobial Resistance Surveillance System**
- **The China Antimicrobial Surveillance Network**
- **National Surveillance of Antimicrobial Resistance, Malaysia**
- **Pakistan Antimicrobial Resistant Network**

Section 2.7: Pharmaceutical sales and antibiotic use

These data were used to model the antibiotic use covariate, which was used as an input in the prevalence of resistance models; full details on this model can be found in section 7. Pharmaceutical sales and antibiotic use data were obtained from the following sources.

- **IMS Health and Quintiles (IQVIA):** antibiotic sales data for 77 countries between 2000 and 2018..
- **Demographic Health Surveys (DH):** households health surveys carried out across more than 90 countries, they include questions on antibiotic usage among those who had cough or diarrhoea in a period of two weeks before the survey.
- **Multiple Indicators Cluster Surveys (MICS):** households health surveys carried out across more than 90 countries, they include questions on antibiotic usage among those who had cough or diarrhoea in a period of two weeks before the survey.
- **European Surveillance of Antimicrobial Consumption Network (ESAC-NET):** antibiotic consumption data for 5 countries over 101 country-years
- **WHO report on surveillance of antibiotic consumption: 2016-2018 early implementation:** report on antibiotic consumption for 21 countries over 21 country-years

Section 2.8: Mortality surveillance

Mortality surveillance data were used in the sepsis, syndrome, and pathogen distribution models; full details on these models can be found in sections 4 and 6. Mortality surveillance data came from the source listed below.

- **Child Health and Mortality Prevention Surveillance (CHAMPS):** Under-5 mortality surveillance sites in South Africa, Mali, Bangladesh, Kenya, Ethiopia, and Mozambique. Researchers use minimally invasive tissue sampling (MITS) to gather information about pathogens involved and are able to discern a more accurate cause of death.

Section 2.9: Linkage (mortality only)

Linkage data were used in sepsis and infectious syndrome models; full details on these models can be found in section 4. Mortality-only linkage data include:

- **Italy Friuli-Venezia Giulia MCoD data**
- **New Zealand linked national minimum dataset to mortality collection data**

Section 3: Summary of GBD 2019 estimation process

A comprehensive description of data sources, data quality, statistical modelling and analyses for GBD 2019 have been reported elsewhere.[1] A brief summary of the fatal and non-fatal estimation processes are briefly summarised below.

Section 3.1: GBD 2019 cause of death estimation process

The overarching steps for the fatal estimation process for each age, sex, location, and year are to first estimate all-cause mortality rates, then calculate cause-specific mortality rates, and finally scale the cause-specific mortality rates to the all-cause mortality rates for internal consistency. First, all-cause mortality is estimated using 7417 sources as data inputs for under-5 mortality estimation and 7355 sources as data inputs for adult mortality estimation. ST-GPR was used to produce estimates of HIV-free mortality rate for every location-year after adjusting for completeness and other known biases in the input data. Added to this HIV-free mortality rate are the HIV-specific mortality rate and deaths from fatal discontinuities, or shocks, which are events that are stochastic in nature and cannot be modelled, such as natural disasters and conflicts. GBD then estimated the cause-specific mortality rates of 301 diseases and injuries. This cause of death analysis utilizes 19 354 sources covering 2525 country years in the cause of death (CoD) database. There are eight types of data sources in the CoD database: vital registration, verbal autopsy,[4] cancer registry, police records, sibling history, surveillance, survey/census, and minimally invasive tissue sampling (MITS) diagnoses. VR is considered the most comprehensive source of cause of death data, but less than half the world's population has deaths captured in a VR system (appendix figure S6), so causes of death statistics are supplemented with other data types. These various data sources are largely ICD coded causes of death and use heterogenous ICD versions so are standardised to GBD causes of death. Once standardised and adjusted for known biases due to ICD classification changes,[5] garbage coding,[5–7] HIV correction,[8] stochastic noise,[1] and completeness,[9] causes of death are modelled using CODEm[10] to determine the cause fraction for each underlying cause of death by age, sex, year, and location. CODEm provides an ensemble prediction based on a combination of candidate models that vary across outcome and covariate combinations chosen for out-of-sample predictive performance. Because each cause is modelled independently, it is possible the sum of these models will not equal the all-cause mortality estimates, so cause-specific results are run through the CoDCorrect process to make cause-specific and all-cause mortality estimates internally consistent. This process rescales cause-specific estimates to the all-cause mortality envelope.

Section 3.2: GBD 2019 non-fatal estimation process

Non-fatal health outcomes are estimated using DisMod-MR 2.1, a Bayesian-regression analytical tool that synthesises various data inputs to produce estimates of disease incidence and prevalence. The data used for this analysis include systematic reviews done at the Institute of Health Metrics and Evaluation (IHME), data from household surveys including the demographic and health surveys, multiple indicator cluster surveys, living standards measurement surveys, reproductive health surveys, administrative claims data, inpatient hospital discharge records, outpatient hospital data, disease registries, programme-level data on disease burden from government agencies, surveillance system data on disease burden, and sources suggested to us by in-country collaborators and surveys identified in major multinational survey data catalogues such as the WHO Central data catalog. 51 272 sources were used for this analysis, 31 499 reporting incidence and 19 773 reporting prevalence. Data from these sources are extracted. Pre-modelling bias adjustments are made using crosswalking to account for various sources of bias, such as heterogeneous case definitions and methods of measurement. The pre-modelling bias adjustments are made using the MR-BRT environment, a meta-regression tool that allows for Bayesian priors, regularization, and trimming and has been described in greater detail previously.[11] Using these bias-adjusted data an estimate of prevalence and incidence for each cause is produced using the DisMod-MR 2.1 modelling framework. DisMod-MR 2.1 accepts all available data on mortality, incidence, prevalence, and remission and uses a compartmental model to enforce consistency between all quantities.

Section 4: Deaths where infection plays a role and infectious syndrome estimation

Section 4.1: Input data

Section 4.1.1: Multiple causes of death

MCoD data are individual-based records that provide underlying causes of death and two or more intermediate causes in the chain of death. Additionally, each record includes age, sex, residence, and the date of death.

Section 4.1.2: Hospital record with multiple diagnoses and discharge status of death

This type of data is an individual-based hospital record of a patient that provides the main diagnosis and two or more additional diagnoses. Additionally, each record includes age, sex, residence, date of admission, date of discharge, and outcome (dead or alive). Only hospital discharges with discharge status of death were used in this component model, since we aimed to estimate the fraction of deaths that involve infection and the infectious syndrome distribution of those deaths.

Section 4.1.3: Linkage data

Linkage data are generated using probabilistic methods in a defined population that link individual-based hospital data to individual-based MCoD data. Linkage data offer a wider dataset that includes main diagnosis, other diagnoses, underlying cause of death, and intermediate causes of death in the chain.

Section 4.1.4: Mortality surveillance (Child Health and Mortality Prevention Surveillance [CHAMPS])

The CHAMPS network tracks the causes of under-5 mortality and stillbirths at sites in sub-Saharan Africa and south Asia through epidemiological surveillance of under-5 deaths and stillbirths utilising minimally invasive tissue sampling (MITS), laboratory diagnostics including conventional and advanced histopathology and molecular screening of various pathogens, verbal autopsy, and available clinical and demographic data.

Table 4.1.5: Input different data point for calculation of fraction of death by sepsis in different underlying causes

Location	Data type	Years	Year range	Deaths
United States	MCoD	38	1980–2017	82,453,798
	Hospital data with fatal outcome	31	1980–2010	2,028,371
	Linkage data			
Brazil	MCoD	19	1999–2017	16,930,050
	Hospital data with fatal outcome	2	2015–2016	294,461
	Linkage data			
Italy	MCoD	13	2003–2015	7,640,383
	Hospital data with fatal outcome	12	2005–2016	2,385,430
	Linkage data	16	2003–2018	112,555
South Africa	MCoD	20	1997–2016	4,696,348
	Hospital data with fatal outcome			
	Linkage data			
Mexico	MCoD	8	2009–2016	4,336,713
	Hospital data with fatal outcome	7	2003–2009	168,582
	Linkage data			
Colombia	MCoD	20	1998–2017	3,624,771
	Hospital data with fatal outcome			
	Linkage data			
Taiwan (province of China)	MCoD	10	2007–2016	1,189,309
	Hospital data with fatal outcome			
	Linkage data			
Austria	MCoD			

	Hospital data with fatal outcome	14	2001–2014	461,538
	Linkage data			
New Zealand	MCoD			
	Hospital data with fatal outcome	18	2000–2017	169,454
	Linkage data	11	2000–2010	151455
Canada	MCoD			
	Hospital data with fatal outcome	16	1994–2009	38,405
	Linkage data			
CHAMPS Surveillance Sites	MITS	3	2017–2019	870
Total	MCoD	128	1980–2017	120,871,372
	Hospital data with fatal outcome	100	1980–2017	5,546,241
	Linkage data	27	2000–2018	264,010
	MITS	3	2017–2019	870

Section 4.2: Data processing

Data for the USA, Brazil, Italy, South Africa, and Mexico were extracted at the subnational level by GBD 2019 age groups, sex, year, and causes of death and/or diagnoses, while data for the remaining countries and territories were analysed at the national level. This allowed us to expand the location-years of data that we had for each Socio-demographic Index (SDI)[12] value.

Section 4.3: Mapping the data

Prepared data were mapped to GBD causes. The GBD cause list is a mutually exclusive and collectively exhaustive list of diseases and injuries. The GBD cause list is organised hierarchically to accommodate different purposes and needs of various users. The first two levels aggregate causes into general groupings. At Level 1, there are three cause groups: communicable, maternal, neonatal, and nutritional diseases (Group 1 diseases); non-communicable diseases (Group 2); and injuries (Group 3). These Level 1 aggregates are subdivided at Level 2 of the hierarchy into 22 cause groupings (eg, neonatal disorders, neurological disorders, and transport injuries). The disaggregation into Levels 3 and 4 contains the finest level of detail for causes captured in GBD 2019. See section 14, table S1 for the full GBD cause hierarchy by level.

The underlying cause of death or main diagnosis for each record in the data was mapped to a GBD cause. After the mapping of underlying cause, we used the GBD 2019 garbage code redistribution algorithm (see appendix 1, section 2.4 in Vos et al.[1]) to ensure that all deaths had a plausible and specific underlying cause of death. The redistribution of garbage codes for underlying causes of death followed the same age and sex restrictions as GBD 2019. We did not redistribute garbage codes in the chain causes because the concept of a garbage code applies only to plausible underlying cause of death (see Rudd et al.[13] and appendix 1, section 2.5 in Vos et al.[1]).

Section 4.4: Intermediate cause and infectious syndrome mapping hierarchy

Section 4.4.1: Intermediate cause mapping

Within our modelling framework, an infectious syndrome is the infection directly responsible for sepsis and serves as the bridge between the underlying cause of death and sepsis. Infectious syndromes can be both underlying causes of death and intermediate causes of death.

For mapping underlying and intermediate causes of death and hospital diagnoses to sepsis and infectious syndromes, we designed a new map, called “AMR, sepsis, and infectious syndrome map”. This map is a list of mutually exclusive and collectively exhaustive infectious syndromes that we divided into four levels to form the infectious syndrome hierarchy.

Each level of infectious syndrome is mutually exclusive and collectively exhaustive. Furthermore, the infectious syndrome hierarchy is internally consistent across any metric (eg, number, cause fraction)—aggregating across

Level 3 syndromes gives us Level 2 syndromes, aggregating the Level 2 syndromes gives us Level 1 syndromes, and the total of Level 1 syndromes is equal to the value of sepsis (figure 4.4.2.1).

Level 0: All International Classification of Diseases 9th (ICD-9) or 10th revision (ICD-10) coded deaths divided into three groups:

- Explicit sepsis (A40, R65.2 in ICD-10 and 039 in ICD-9): Any death has specific ICD code for sepsis in the MCoD chain or hospital diagnoses was considered explicit sepsis[13]
- Implicit sepsis: Any death that has an infectious disease code in the underlying cause or cause chain and a specific organ dysfunction code was considered implicit sepsis
- Non-sepsis: Any death that does not meet either of the two above criteria (section 14, tables S2, S3)

Of the estimated infection-related deaths with explicit sepsis or implicit sepsis and infectious diseases, 59.4% occur with communicable, maternal, neonatal, and nutritional underlying causes of death. 38.9% infection related deaths occur with non-communicable disease as the underlying cause of death, and 1.7% occur with injuries as the underlying cause of death.

Level 1: All implicit and explicit sepsis deaths were divided into 12 Level 1 infectious syndromes and an "other" category (table 4.4.1.1).

Table 4.4.1.1: Level 1 of infectious syndromes

	**Infectious syndrome**
1	Bacterial infections of the skin and subcutaneous systems
2	Bloodstream infections
3	Gonorrhoea and chlamydia
4	Diarrhoea
5	Endocarditis and other cardiac infections
6	Infections of bones, joints, and related organs
7	Lower respiratory infections and all related infections in the thorax
8	Meningitis and other bacterial central nervous system infections
9	Peritoneal and intra-abdominal infections
10	Tuberculosis
11	Typhoid, paratyphoid, and invasive non-typhoidal Salmonella
12	Urinary tract infections and pyelonephritis
13	Other infections

Level 2: Each Level 1 infectious syndrome was divided into Level 2 infectious syndromes based on the pathogen type (eg, bacterial, fungal, viral) causing the infection. Examples include specified bacterial, unspecified bacterial, fungal, viral, and unspecified pathogen.

Level 3: Each specified bacterial infectious syndrome in Level 2 was divided to Level 3 infectious syndromes by the bacterium causing infection. Table S3 (section 14) shows this list and bacterial hierarchy.

Figure 4.4.2.1. Infectious syndrome hierarchy

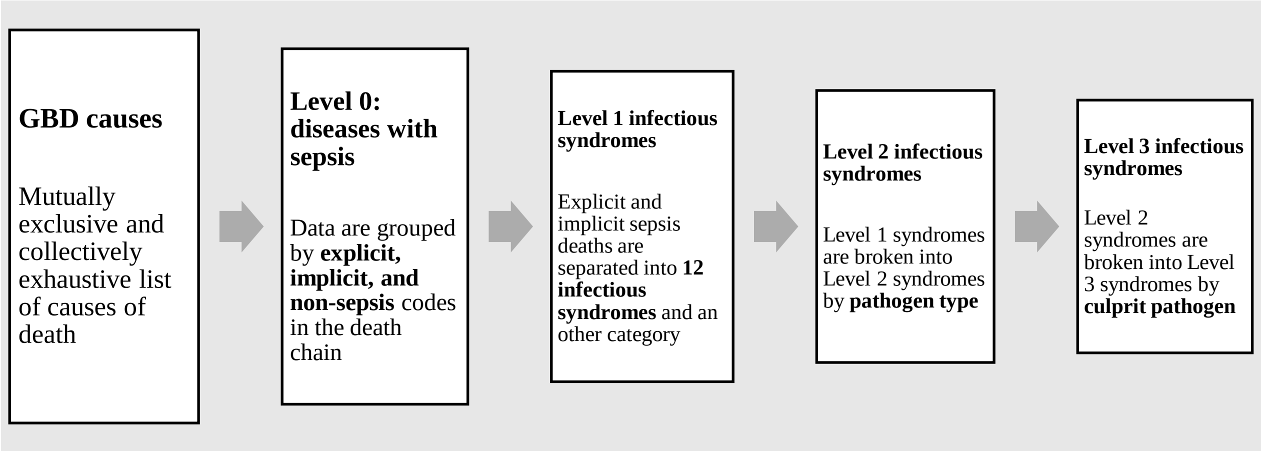

Section 4.4.3: Informative ranking

Due to our data often having multiple diagnoses associated with each record, a single case of sepsis could potentially map to multiple candidate infectious syndromes. Because multiple infectious syndrome assignments pose a risk of double counting, we employed an informative ranking hierarchy. The informative ranking allowed us to determine the infectious syndrome that provided the most information on the culprit pathogen. The goal of this hierarchy was to produce the most accurate pathogen burden estimate such that when there were multiple infectious syndromes, we prioritised the syndrome with the most distinctive distribution. For example, bloodstream infections (BSIs) are common infections in sepsis but there is often an earlier source of the infection such as a UTI, cellulitis, or LRI, and each has a unique pathogen distribution that provides more information than the distribution of BSI. In the event that a patient record reflected both BSI and LRI, we would assign the infectious syndrome based on the pathogen distribution that would be the most proximal aetiologic syndrome, LRI (see table 4.4.3.1).

Table 4.4.3.1. Level 1 Infectious syndrome informative ranking hierarchy

Organised from most informative (top) to least (bottom).

**Level 1 infectious syndrome informative ranking hierarchy**
Meningitis and other bacterial central nervous system infections
Endocarditis and other cardiac infections
Peritoneal and intra-abdominal infections
Lower respiratory infections and all related infections in the thorax
Bacterial infections of the skin and subcutaneous systems
Infections of bone, joints, and related organs
Diarrhoea
Urinary tract infections and pyelonephritis
Other infections
Bloodstream infections

Section 4.4.4: Two modelling pathways

After mapping the underlying and chain causes of death, our database went through two separate modelling pathways. The first model estimated the fraction of deaths that are sepsis-related in each GBD cause; these sepsis-related deaths for non-infectious GBD causes were combined with GBD deaths for infectious causes to create the total envelope of all deaths where infection plays a role. The second pathway estimated each infectious syndrome as a fraction of sepsis-related mortality in each GBD cause. In the last step of infectious syndrome estimation, the fractions of sepsis by Level 1 infectious syndromes were squeezed to sum to one so as to not exceed the sepsis mortality envelope and multiplied by the sepsis estimate in each GBD cause by country and territory, age, and sex in

2019.

Section 4.5: First pathway: deaths where infection plays a role

Section 4.5.1: Sepsis model

We used a mixed-effects binomial logistic regression to model the logit of the fraction of sepsis-related deaths by GBD cause-age-sex-location, consistent with the modelling approach used by Rudd et al.[13] Sex and Healthcare Access and Quality Index (HAQ Index)[14] were included as covariates and a nested random effect on underlying cause of death was included. A separate model was run for each GBD 2019 age group (0–6, 7–27, 28-364 [days], 1–4, 5–9, 10–14, 15–19, 20–24, 25–29, 30–34, 35–39, 40–44, 45–49, 50–54, 55–59, 60–64, 65–69, 70–74, 75–79, 80–84, 85–89, 90–94, 95+ [years]):

$$sepsis\ related\ deaths \sim B(total\ deaths, sepsis\ fraction) \qquad (4.5.1.1)$$

$$logit(sepsis\ fraction) = \beta_0 + \beta_1 * HAQ\ Index + \beta_2 * sex + \pi_{level\ 1, level\ 2}$$

Where $\pi_{level\ 1, level\ 2}$ is a nested random effect on underlying cause of death. The nested random-effect's structure in the model on underlying cause of death allowed the prediction of sepsis fractions where data were limited by borrowing information from diseases within the same group. There were 22 groups of underlying causes of death, each categorised by physiological relatedness. We produced our predictions and uncertainty intervals (UIs) by generating 1000 draws from the normal distribution of the fixed coefficients, separately for each GBD location, age group, sex, and cause in 2019. The means of our results were used for the point estimates and the 95% UIs were delineated using the 2.5th and 97.5th percentiles of the draws. Uncertainty is attributable to sample size variability between data sources, data availability, and model specifications.

All underlying causes of death that are infectious diseases were included in the model; however, for these causes we used the GBD death estimates rather than the modelled sepsis estimate, since infection inherently plays a role in these deaths even if the pathway doesn't include sepsis. These causes and their associated infectious syndromes are listed in table 4.5.1.1.

Table 4.5.1.1. Underlying causes that are infectious diseases and their corresponding syndromes

Cause name	Infectious syndrome
Appendicitis	Peritoneal and intra-abdominal infections
Bacterial skin diseases	Bacterial infections of the skin and subcutaneous systems
Chlamydial infection	Gonorrhoea and chlamydia
Diarrhoeal diseases	Diarrhoea
Endocarditis	Endocarditis and other cardiac infections
Gonococcal infection	Gonorrhoea and chlamydia
Invasive non-typhoidal Salmonella	Typhoid, paratyphoid, and invasive non-typhoidal Salmonella
Lower respiratory infections	Lower respiratory infections and all related infections in the thorax
Maternal sepsis and other maternal infections	Bloodstream infections
Meningitis	Meningitis and other bacterial central nervous system infections
Neonatal sepsis and other neonatal infections	Bloodstream infections
Paratyphoid fever	Typhoid, paratyphoid, and invasive non-typhoidal Salmonella
Tuberculosis	Tuberculosis
Typhoid fever	Typhoid, paratyphoid, and invasive non-typhoidal Salmonella
Upper respiratory infections	Lower respiratory infections and all related infections in the thorax
Urinary tract infections and interstitial nephritis	Urinary tract infections and pyelonephritis

For all other causes, we calculated the number of sepsis-related deaths in 2019 by multiplying our predictions of cause-, age group-, sex-, year-, and location-specific sepsis fractions by GBD 2019 death estimates. Finally, we aggregated our results to arrive at regional and global sepsis-related mortality in non-infectious underlying causes of death, which we combined with the GBD infectious disease deaths estimates to create the mortality envelope of all deaths related to infection.

For transparency, histograms of the available input data by HAQ Index are shown below. MCoD input data is used to estimate the proportion of non-infectious disease that involves sepsis, while the GBD mortality data for Group 1

causes (communicable, maternal, neonatal, and nutritional diseases) is inclusive and representative of the input data used to estimate mortality associated with primary infection underlying cause.

Figure 4.5.1.1. MCoD input data by HAQ Index

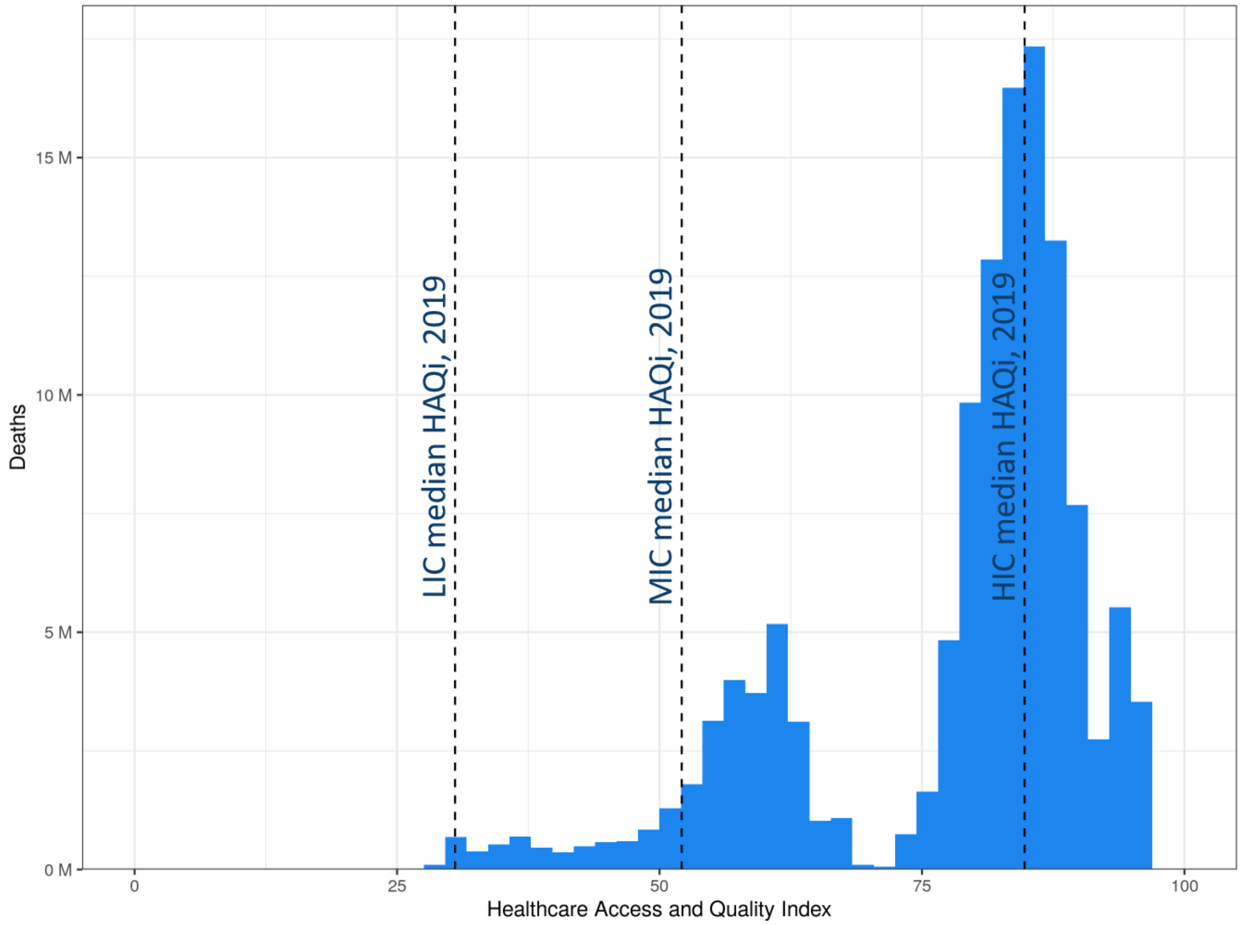

Figure 4.5.1.2. GBD mortality input data for Group 1 (communicable, maternal, neonatal, and nutritional diseases) by HAQ Index

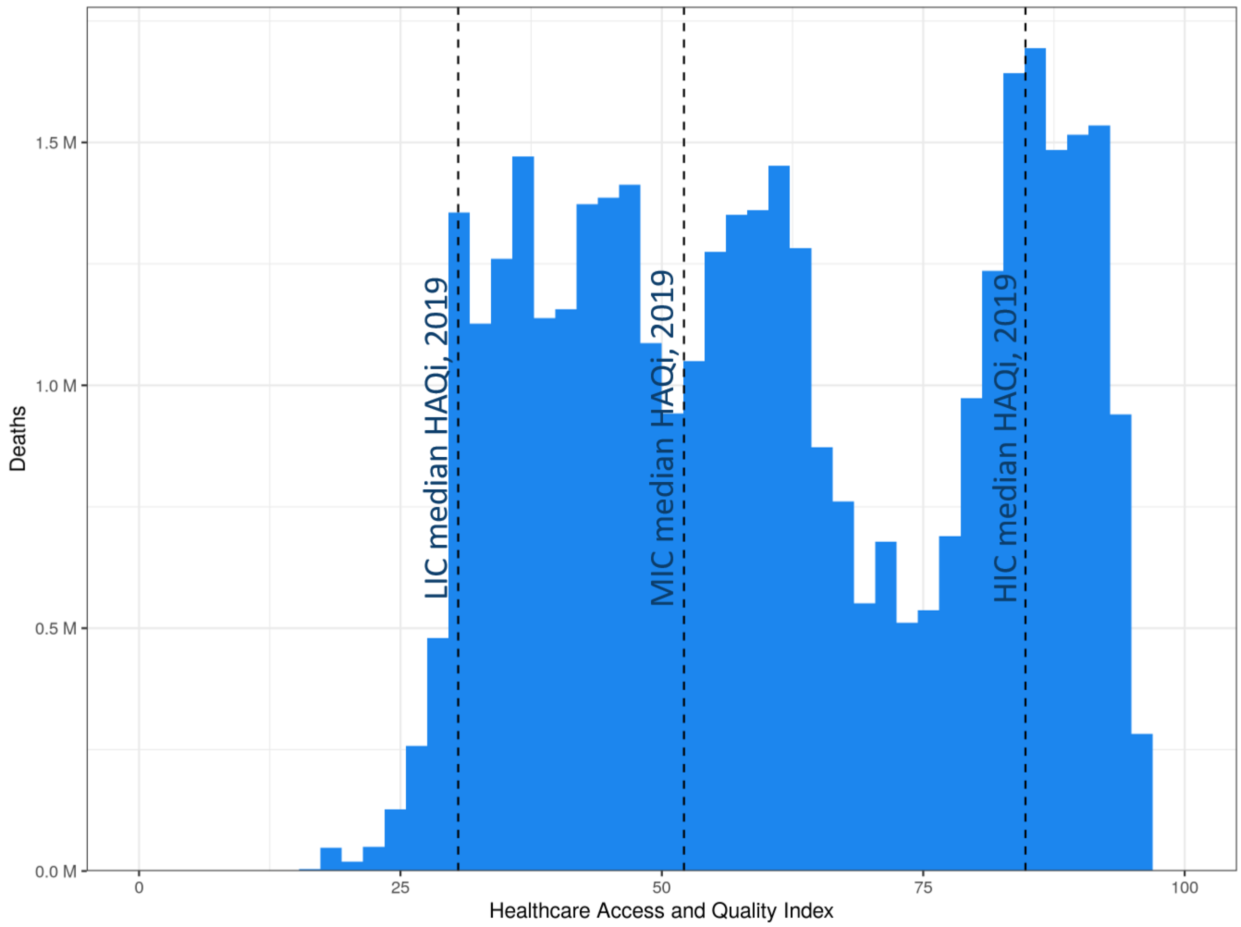

HIC = high-income country, LIC = low-income country, MIC = middle-income country

Section 4.6: Second pathway: fraction of deaths where infection plays a role by infectious syndrome in each GBD cause

We used a mixed-effects binomial logistic regression to model the logit of the infectious syndrome fraction of sepsis-related mortality by GBD cause. The model covariates varied by infectious syndrome (table 4.6.1). All models included HAQ Index as a covariate and most included a summary exposure value (SEV) scalar calculated for GBD 2019.

The pathogen distribution for hospital-acquired infections (HAIs) and community-acquired infections (CAIs) differs markedly for some infectious syndromes.[15–20] To more accurately estimate the burden of pathogens responsible for infection, we separated infectious syndromes into hospital-acquired and community-acquired for LRI+ and UTI. For all ICD-coded administrative datasets (hospital discharge, MCoD, and linkage), we assumed that an infection was community-acquired if it was the primary diagnosis or underlying cause of death. Similarly, an infection was considered hospital-acquired if it was not the primary diagnosis or underlying cause of death. We recognise that this is a strong assumption that will not always be correct; however, there is no established method for determining HAI versus CAI in administrative data.[21,22] We considered it to be more important to estimate hospital- and community-acquired separately to account for their distinct pathogen distributions despite the strong assumptions involved. We present the fraction of all infectious syndrome deaths in 2019 that our model predicted to be hospital-acquired for LRI+ and UTI in table 4.6.2 for transparency.

Table 4.6.1: Infectious syndrome model covariates and age groups

Infectious syndrome	Covariates	Age groups modelled
Bloodstream infections	HAQ Index[14]<br>Sex<br>SEV scalar of maternal sepsis[23]<br>SEV scalar of neonatal sepsis[23]	GBD 2019 age groups
Infections of bone, joints, and related organs	HAQ Index<br>Sex	0–9, 10–14, 15–19, 20–24, 25–39, 40–44, 45–49, 50–54, 55–59, 60–64. 65–69, 70–74, 75–79, 80–84, 85–89, 90–94, 95+
Endocarditis and other cardiac infections	HAQ Index<br>Sex<br>SEV scalar of age-standardised endocarditis[23]	GBD 2019 age groups
Meningitis and other bacterial central nervous system infections	HAQ Index<br>Sex<br>SEV scalar of age-standardised meningitis[23]	GBD 2019 age groups
Diarrhoea	HAQ Index<br>Sex<br>SEV scalar of age-standardised diarrhoea[23]	GBD 2019 age groups
Other infections	HAQ Index<br>Sex	GBD 2019 age groups
Peritoneal and intra-abdominal infections	HAQ Index<br>Sex	GBD 2019 age groups
CAI lower respiratory infections and all related infections in the thorax	HAQ Index<br>Sex<br>SEV scalar of age-standardised LRIs[23]	Neonatal, Post neonatal–5, 5–69, 70+
HAI lower respiratory infections and all related infections in the thorax	HAQ Index<br>Sex<br>SEV scalar of age-standardised LRIs	Neonatal, Post neonatal–5, 5–69, 70+
Bacterial infections of the skin and subcutaneous systems	HAQ Index<br>Sex<br>SEV scalar of age-standardised no access to handwashing facility[23]	GBD 2019 age groups
CAI urinary tract infections and pyelonephritis	HAQ Index<br>Sex<br>SEV scalar of age-standardised no access to handwashing facility	0–39, 40+
HAI urinary tract infections and pyelonephritis	HAQ Index<br>Sex<br>SEV scalar of age-standardised no access to handwashing facility	0–39, 40+

CAI=community-acquired infection. HAI=hospital-acquired infection. HAQ Index=Healthcare Access and Quality Index. LRI=lower respiratory infection. SEV=summary exposure value. SEVs are a risk-weighted prevalence calculated based on exposure, from 0, where the entire population (among the age groups where exposure is possible) is exposed at the minimum risk level, to 100, where the entire population is exposed at the maximum risk exposure level.

Table 4.6.2: Model predictions for proportion of deaths in 2019 that were hospital-acquired by GBD super-region and infectious syndrome

Infectious syndrome	Super-region	Proportion (95% UI)
Lower respiratory infections and all related infections in the thorax	Global	17.4% (13.1 - 22.4)
	Southeast Asia, east Asia, and Oceania	23.3% (18.0 - 28.9)

	Central Europe, eastern Europe, and central Asia	27.9% (20.5 - 35.8)
	High-income	17.8% (12.5 - 24.8)
	Latin America and Caribbean	24.8% (19.1 - 30.7)
	North Africa and Middle East	22.2% (17.7 - 26.8)
	South Asia	16.4% (13.0 - 20.1)
	Sub-Saharan Africa	9.3% (6.9 - 12.8)
Urinary tract infections and pyelonephritis	Global	39.1% (32.4 - 61.5)
	Southeast Asia, east Asia, and Oceania	64.5% (48.4 - 74.7)
	Central Europe, eastern Europe, and central Asia	48.4% (32.4 - 61.5)
	High-income	27.2% (15.8 - 40.7)
	Latin America and Caribbean	28.5% (20.2 - 40.6)
	North Africa and Middle East	73.0% (58.2 - 80.0)
	South Asia	29.9% (20.2 - 40.7)
	Sub-Saharan Africa	24.9% (18.0 - 31.1)

The infectious syndrome models were specified as mixed-effects binomial logistic regressions, one for each infectious syndrome and age group:

$$syndrome\ related\ deaths \sim B(total\ sepsis\ deaths, syndrome\ fraction) \tag{4.6.2.1}$$

$$logit(syndrome\ fraction) = \beta_0 + \beta * X + \pi_{level\ 1, level\ 2}$$

where β and X are vectors of length $n + 1$ for n covariates and $\pi_{level\ 1, level\ 2}$ is a nested random effect on underlying cause of death. The granularity of the age groups estimated for each infectious syndrome was chosen based on the age pattern of the infectious syndrome and the limitations of data sparsity.

As in the first pathway, we derived our predictions and UIs by generating 1000 draws from the normal distribution of the fixed coefficients separately for each GBD location, age group, sex, and cause in 2019. We used the means of our results for the point estimates and the 95% UIs were delineated using the 2.5th and 97.5th percentiles of the draws.

Section 4.6.3: Aggregation to the sepsis mortality envelope

We calculated the number of deaths attributable to each infectious syndrome in 2019 by multiplying our predictions of cause-, age group-, sex-, year-, and location-specific infectious syndrome fractions by our sepsis-mortality estimates from the first pathway. All infectious syndrome fractions were squeezed to sum to one prior to multiplication in order to ensure that we did not exceed the sepsis mortality envelope.

Finally, we aggregated our results to arrive at regional and global sepsis-related mortality by infectious syndrome.

Section 4.6.4: Infectious syndromes using GBD 2019 results

Out of the 12 explicit Level 1 infectious syndromes included in our hierarchy, we excluded (i) tuberculosis (TB), (ii) typhoid, paratyphoid, and invasive non-typhoidal Salmonella, and (iii) gonorrhoea and chlamydia from our binomial mixed-effects linear regression model. Instead, we used the published results from GBD 2019[1] for these causes of death, as we believe the GBD 2019 estimates fully represent these infectious syndromes because they are usually not intermediate causes of death.

Section 4.7: Model validation

Infectious syndrome modelling aims to predict which cases of infection belong to a specific infectious syndrome, which is a multi-class classification problem. We therefore use the Area Under the Receiver Operating Characteristics (ROC) Curve (AUC) to evaluate model performance. The ROC Curve is determined by the sensitivity (or true positive rate) and the specificity (or false positive rate) of the model, and a higher AUC score

indicates that the model is capable of discerning between the different categories. Accuracy is a related measure which considers the proportion of true positives and true negatives predicted by the model with respect to the total number of predictions.

The out-of-sample strategy for this validation excluded 20% of the sample on each iteration. Table 4.7.1 reports the Accuracy and AUC score[24] for each of the age groups within the infectious syndrome models and table 4.7.2 reports the same metrics for the sepsis models. 99% of the models have an AUC score between 0.7 and 1, indicating an overall excellent performance of this modelling framework.

Table 4.7.1: Accuracy and AUC score for out-of-sample validation of infectious syndromes models

Model	Age group name	Accuracy	AUC score
Bacterial infections of the skin and subcutaneous systems	1 to 4	1.00	0.87
Bacterial infections of the skin and subcutaneous systems	10 to 14	0.99	0.87
Bacterial infections of the skin and subcutaneous systems	15 to 19	0.98	0.90
Bacterial infections of the skin and subcutaneous systems	20 to 24	0.99	0.93
Bacterial infections of the skin and subcutaneous systems	25 to 29	0.99	0.94
Bacterial infections of the skin and subcutaneous systems	30 to 34	0.99	0.95
Bacterial infections of the skin and subcutaneous systems	35 to 39	0.99	0.94
Bacterial infections of the skin and subcutaneous systems	40 to 44	0.98	0.94
Bacterial infections of the skin and subcutaneous systems	45 to 49	0.98	0.93
Bacterial infections of the skin and subcutaneous systems	5 to 9	0.99	0.87
Bacterial infections of the skin and subcutaneous systems	50 to 54	0.98	0.92
Bacterial infections of the skin and subcutaneous systems	55 to 59	0.97	0.92
Bacterial infections of the skin and subcutaneous systems	60 to 64	0.97	0.92
Bacterial infections of the skin and subcutaneous systems	65 to 69	0.97	0.92
Bacterial infections of the skin and subcutaneous systems	70 to 74	0.98	0.92
Bacterial infections of the skin and subcutaneous systems	75 to 79	0.98	0.93
Bacterial infections of the skin and subcutaneous systems	80 to 84	0.98	0.94
Bacterial infections of the skin and subcutaneous systems	85 to 89	0.98	0.95
Bacterial infections of the skin and subcutaneous systems	90 to 94	0.98	0.96
Bacterial infections of the skin and subcutaneous systems	95 plus	0.98	0.97
Bacterial infections of the skin and subcutaneous systems	Early Neonatal	0.99	0.94
Bacterial infections of the skin and subcutaneous systems	Late Neonatal	0.99	0.98
Bacterial infections of the skin and subcutaneous systems	Post Neonatal	1.00	0.89
Bloodstream infections	1 to 4	0.91	0.95
Bloodstream infections	10 to 14	0.85	0.92
Bloodstream infections	15 to 19	0.84	0.91
Bloodstream infections	20 to 24	0.89	0.94
Bloodstream infections	25 to 29	0.92	0.94
Bloodstream infections	30 to 34	0.93	0.94
Bloodstream infections	35 to 39	0.92	0.93
Bloodstream infections	40 to 44	0.90	0.92
Bloodstream infections	45 to 49	0.89	0.90
Bloodstream infections	5 to 9	0.87	0.94

Bloodstream infections	50 to 54	0.88	0.89
Bloodstream infections	55 to 59	0.88	0.87
Bloodstream infections	60 to 64	0.88	0.87
Bloodstream infections	65 to 69	0.89	0.87
Bloodstream infections	70 to 74	0.90	0.88
Bloodstream infections	75 to 79	0.91	0.89
Bloodstream infections	80 to 84	0.92	0.91
Bloodstream infections	85 to 89	0.93	0.92
Bloodstream infections	90 to 94	0.94	0.93
Bloodstream infections	95 plus	0.94	0.95
Bloodstream infections	Early Neonatal	0.94	0.96
Bloodstream infections	Late Neonatal	0.95	0.96
Bloodstream infections	Post Neonatal	0.93	0.96
CAI lower respiratory infections and all related infections in the thorax	5 to 69	0.99	0.99
CAI lower respiratory infections and all related infections in the thorax	70+ years	0.99	1.00
CAI lower respiratory infections and all related infections in the thorax	Neonatal	0.95	0.96
CAI lower respiratory infections and all related infections in the thorax	Post Neonatal to 5	0.99	1.00
CAI urinary tract infections and pyelonephritis	0 to 39	1.00	1.00
CAI urinary tract infections and pyelonephritis	40 plus	1.00	1.00
Diarrhoea	1 to 4	0.99	1.00
Diarrhoea	10 to 14	0.99	0.99
Diarrhoea	15 to 19	0.99	0.99
Diarrhoea	20 to 24	0.99	1.00
Diarrhoea	25 to 29	0.99	1.00
Diarrhoea	30 to 34	0.99	1.00
Diarrhoea	35 to 39	0.99	1.00
Diarrhoea	40 to 44	0.99	0.99
Diarrhoea	45 to 49	0.99	0.99
Diarrhoea	5 to 9	0.99	0.99
Diarrhoea	50 to 54	0.99	0.98
Diarrhoea	55 to 59	0.99	0.97
Diarrhoea	60 to 64	0.99	0.97
Diarrhoea	65 to 69	0.99	0.96
Diarrhoea	70 to 74	0.99	0.96
Diarrhoea	75 to 79	0.99	0.97
Diarrhoea	80 to 84	0.99	0.97
Diarrhoea	85 to 89	0.99	0.98
Diarrhoea	90 to 94	0.99	0.98
Diarrhoea	95 plus	0.99	0.99
Diarrhoea	Early Neonatal	1.00	1.00
Diarrhoea	Late Neonatal	1.00	1.00

Diarrhoea	Post Neonatal	0.98	0.99
Endocarditis and other cardiac infections	1 to 4	0.99	0.94
Endocarditis and other cardiac infections	10 to 14	0.99	0.97
Endocarditis and other cardiac infections	15 to 19	0.99	0.96
Endocarditis and other cardiac infections	20 to 24	0.99	0.96
Endocarditis and other cardiac infections	25 to 29	0.99	0.97
Endocarditis and other cardiac infections	30 to 34	0.99	0.97
Endocarditis and other cardiac infections	35 to 39	0.99	0.97
Endocarditis and other cardiac infections	40 to 44	0.99	0.97
Endocarditis and other cardiac infections	45 to 49	0.99	0.96
Endocarditis and other cardiac infections	5 to 9	0.99	0.95
Endocarditis and other cardiac infections	50 to 54	0.99	0.96
Endocarditis and other cardiac infections	55 to 59	0.99	0.95
Endocarditis and other cardiac infections	60 to 64	0.99	0.95
Endocarditis and other cardiac infections	65 to 69	0.99	0.95
Endocarditis and other cardiac infections	70 to 74	0.99	0.96
Endocarditis and other cardiac infections	75 to 79	0.99	0.96
Endocarditis and other cardiac infections	80 to 84	0.99	0.97
Endocarditis and other cardiac infections	85 to 89	0.99	0.98
Endocarditis and other cardiac infections	90 to 94	0.99	0.98
Endocarditis and other cardiac infections	95 plus	0.99	0.98
Endocarditis and other cardiac infections	Early Neonatal	0.99	0.98
Endocarditis and other cardiac infections	Late Neonatal	0.99	0.98
Endocarditis and other cardiac infections	Post Neonatal	0.99	0.89
HAI lower respiratory infections and all related infections in the thorax	5 to 69	0.96	0.89
HAI lower respiratory infections and all related infections in the thorax	70+ years	0.96	0.89
HAI lower respiratory infections and all related infections in the thorax	Neonatal	0.99	0.50
HAI lower respiratory infections and all related infections in the thorax	Post Neonatal to 5	0.97	0.94
HAI urinary tract infections and pyelonephritis	0 to 39	0.99	0.77
HAI urinary tract infections and pyelonephritis	40 plus	0.99	0.86
Infections of bone, joints, and related organs	0 to 9	0.99	0.94
Infections of bone, joints, and related organs	10 to 14	0.99	0.95
Infections of bone, joints, and related organs	15 to 19	0.99	0.88
Infections of bone, joints, and related organs	20 to 24	0.99	0.82
Infections of bone, joints, and related organs	25 to 29	0.99	0.85
Infections of bone, joints, and related organs	30 to 34	0.99	0.83
Infections of bone, joints, and related organs	35 to 39	0.99	0.85
Infections of bone, joints, and related organs	40 to 44	0.99	0.84
Infections of bone, joints, and related organs	45 to 49	0.99	0.84
Infections of bone, joints, and related organs	50 to 54	0.99	0.88
Infections of bone, joints, and related organs	55 to 59	0.99	0.89

Infections of bone, joints, and related organs	60 to 64	0.99	0.90
Infections of bone, joints, and related organs	65 to 69	0.99	0.90
Infections of bone, joints, and related organs	70 to 74	0.99	0.91
Infections of bone, joints, and related organs	75 to 79	0.99	0.92
Infections of bone, joints, and related organs	80 to 84	0.99	0.93
Infections of bone, joints, and related organs	85 to 89	0.99	0.94
Infections of bone, joints, and related organs	90 to 94	0.99	0.94
Infections of bone, joints, and related organs	95 plus	0.99	0.95
Meningitis and other bacterial central nervous system infections	1 to 4	0.98	0.98
Meningitis and other bacterial central nervous system infections	10 to 14	0.97	0.98
Meningitis and other bacterial central nervous system infections	15 to 19	0.98	0.97
Meningitis and other bacterial central nervous system infections	20 to 24	0.99	0.97
Meningitis and other bacterial central nervous system infections	25 to 29	0.99	0.98
Meningitis and other bacterial central nervous system infections	30 to 34	0.99	0.98
Meningitis and other bacterial central nervous system infections	35 to 39	0.99	0.97
Meningitis and other bacterial central nervous system infections	40 to 44	0.99	0.96
Meningitis and other bacterial central nervous system infections	45 to 49	0.99	0.96
Meningitis and other bacterial central nervous system infections	5 to 9	0.97	0.97
Meningitis and other bacterial central nervous system infections	50 to 54	0.99	0.94
Meningitis and other bacterial central nervous system infections	55 to 59	0.99	0.93
Meningitis and other bacterial central nervous system infections	60 to 64	0.99	0.93
Meningitis and other bacterial central nervous system infections	65 to 69	0.99	0.92
Meningitis and other bacterial central nervous system infections	70 to 74	0.99	0.92
Meningitis and other bacterial central nervous system infections	75 to 79	0.99	0.91
Meningitis and other bacterial central nervous system infections	80 to 84	0.99	0.92
Meningitis and other bacterial central nervous system infections	85 to 89	0.99	0.92
Meningitis and other bacterial central nervous system infections	90 to 94	0.99	0.93
Meningitis and other bacterial central nervous system infections	95 plus	0.99	0.92
Meningitis and other bacterial central nervous system infections	Early Neonatal	0.99	0.99
Meningitis and other bacterial central nervous system infections	Late Neonatal	1.00	0.99
Meningitis and other bacterial central nervous system infections	Post Neonatal	0.99	0.98
Peritoneal and intra-abdominal infections	1 to 4	0.99	0.97
Peritoneal and intra-abdominal infections	10 to 14	0.97	0.96
Peritoneal and intra-abdominal infections	15 to 19	0.96	0.96
Peritoneal and intra-abdominal infections	20 to 24	0.97	0.97
Peritoneal and intra-abdominal infections	25 to 29	0.98	0.98
Peritoneal and intra-abdominal infections	30 to 34	0.98	0.98
Peritoneal and intra-abdominal infections	35 to 39	0.98	0.98
Peritoneal and intra-abdominal infections	40 to 44	0.97	0.97
Peritoneal and intra-abdominal infections	45 to 49	0.97	0.96
Peritoneal and intra-abdominal infections	5 to 9	0.98	0.97

Peritoneal and intra-abdominal infections	50 to 54	0.96	0.95
Peritoneal and intra-abdominal infections	55 to 59	0.96	0.95
Peritoneal and intra-abdominal infections	60 to 64	0.96	0.95
Peritoneal and intra-abdominal infections	65 to 69	0.96	0.95
Peritoneal and intra-abdominal infections	70 to 74	0.97	0.96
Peritoneal and intra-abdominal infections	75 to 79	0.97	0.97
Peritoneal and intra-abdominal infections	80 to 84	0.98	0.98
Peritoneal and intra-abdominal infections	85 to 89	0.98	0.98
Peritoneal and intra-abdominal infections	90 to 94	0.98	0.98
Peritoneal and intra-abdominal infections	95 plus	0.99	0.98
Peritoneal and intra-abdominal infections	Early Neonatal	0.99	0.97
Peritoneal and intra-abdominal infections	Late Neonatal	0.99	0.97
Peritoneal and intra-abdominal infections	Post Neonatal	0.98	0.94

Table 4.7.2: Accuracy and AUC score for out-of-sample validation of sepsis models

**Model**	**Age group name**	**Accuracy**	**AUC score**
Sepsis	1 to 4	0.89	0.93
Sepsis	10 to 14	0.90	0.92
Sepsis	15 to 19	0.95	0.94
Sepsis	20 to 24	0.95	0.94
Sepsis	25 to 29	0.94	0.95
Sepsis	30 to 34	0.93	0.94
Sepsis	35 to 39	0.93	0.93
Sepsis	40 to 44	0.93	0.91
Sepsis	45 to 49	0.93	0.88
Sepsis	5 to 9	0.89	0.92
Sepsis	50 to 54	0.93	0.86
Sepsis	55 to 59	0.94	0.84
Sepsis	60 to 64	0.94	0.83
Sepsis	65 to 69	0.94	0.83
Sepsis	70 to 74	0.95	0.84
Sepsis	75 to 79	0.95	0.85
Sepsis	80 to 84	0.96	0.87
Sepsis	85 to 89	0.96	0.88
Sepsis	90 to 94	0.96	0.90
Sepsis	95 plus	0.96	0.92
Sepsis	Early Neonatal	0.91	0.87
Sepsis	Late Neonatal	0.87	0.88
Sepsis	Post Neonatal	0.88	0.89

Section 5: Case fatality ratios

Section 5.1: Input data

Case fatality ratios (CFRs) were modelled for the pathogens and infectious syndromes of interest using all available data detailing the organism responsible for infection, the infectious syndrome, and patient outcome. This included hospital and microbial data, totaling 19.7 million isolates and cases, as shown in table S4 (section 14). We additionally included 52 907 cases from literature sources for CNS infections, which had been previously extracted for a systematic review in GBD.

Section 5.2: Data processing

All input data sources were processed as described in sections 6.2.1–6.2.4 and section 6.2.7 and pathogens of interest were chosen as described in section 6.2.5. Input data for the CFR models were aggregated based on data source, year, GBD location, and age group (as well as hospital/community acquired status, in the case of the lower respiratory and urogenital infectious models). For lower respiratory and blood stream infections, for which CFRs could be vastly different in neonates, we modelled the following age groups: neonatal, post-neonatal–5 years, 5–50 years, 50–70 years, and 70 years and older. For all other infectious syndromes, we modelled the following age groups: neonatal–5 years, 5–50 years, 50–70 years, and 70 years and older. We excluded from the analysis any source-location-year-age with fewer than five cases and zero deaths.

To allow us to implement linear models, CFRs were logit-transformed. We used the delta method to compute the standard error of CFRs in logit space. To incorporate data with zero deaths, or with an equal number of deaths and cases, we applied a 1% offset, such that the CFRs for data with zero deaths was represented as 1% and the CFR for data with an equal number of deaths and cases was represented as 99%.

Section 5.3: Modelling overview

Pathogen-specific CFRs were modelled separately by infectious syndrome and were calculated as a function of HAQ Index and age. We used the HAQ Index to extrapolate CFRs determined from the input data, which often had a broad but not comprehensive geographic scope, to all 204 GBD countries and territories. To account for heterogeneity across the sources of input data, we implemented a mixed-effects meta-regression framework, modelling data source as a random effect. We further incorporated a binary fixed-effect denoting whether the data source only included intensive care unit (ICU) patients, for which CFRs were expected to be higher.

The pathogens of interest for each infectious syndrome were determined by prevalence in the data and expert opinion, with the goal of modelling approximately 90% of specified-pathogens associated with each infectious syndrome (see section 6.2.5). Because each data source generally reported only a set of the pathogens we evaluated in our research, the input data for the pathogens varied in geographic coverage; nearly all pathogens were well reported in high-income areas, but some pathogens were not well represented in the smaller subset of data we collected from low- and middle-income locations.

For those pathogens with 'rich' data, defined by our method as having at least ten high-quality data points below a moderate HAQ Index (0.7), we modelled a unique effect of HAQ Index, achieved by interacting the HAQ Index fixed-effect with the pathogen-specific fixed-effect. This process, referred to from here on as the 'interaction model,' allowed the relative deadliness of pathogens to vary depending on a location's HAQ Index. For those pathogens with fewer than 10 high quality data points below 0.7 HAQ Index, or those whose results in the interaction models indicated an unrealistically large influence of HAQ Index (eg, 70% CFR in low HAQ Index countries, 1% CFR in high HAQ Index countries), we modelled a pathogen-specific intercept with an HAQ Index fixed-effect shared across the pathogens. As a consequence of the single fixed-effect on HAQ Index, a pathogen that was predicted to be the deadliest in low HAQ Index countries would also be predicted to be the deadliest in high HAQ Index countries in these 'intercept models.' To estimate the CFRs for other known bacteria, which either were not selected as a pathogen of interest or lacked sufficient data for inclusion in the intercept models, we pooled all bacterial data together and estimated a single CFR curve from age, HAQ Index, and the data source heterogeneity covariates. Thus, up to three models were run for each infectious syndrome:

1) an interaction model including data for all data rich pathogens and 'other specified bacteria' (which was included to inform the overall influence of HAQ Index on CFR, predictions were only generated for the data rich pathogens),
2) an intercept model including data for data rich and data sparse pathogens, as well as 'other specified bacteria' (predictions were only generated for the data sparse pathogens), and
3) an 'other bacteria' model that included data for all bacterial pathogens (predictions were generated by HAQ Index and age, without any pathogen specific term).

Table S5 (section 14) details which CFR model framework was used to assess the pathogens for each infectious syndrome. Whenever needed, the CFR for any bacterial pathogen "not explicitly modelled" was estimated using the 'other bacteria' model for subsequent steps of our modelling processes.

For some infectious syndromes, the relative deadliness of a pathogen may be strongly determined by either the age of the patient or whether the infection was community- or hospital-acquired. For bloodstream infections, we ran two distinct sets of CFR models, one for neonates (0-27 days) and another for post neonates, to capture the differing dynamics of pathogen deadliness in these two populations. As is done for our other modelling processes, we also separate community-acquired and hospital-acquired cases in our CFR models for lower respiratory and urogenital infections. Because some data sources did not provide enough information to infer whether an infection was community- or hospital-acquired, but still included important information on the relative pathogenesis and the difference in CFRs across varying HAQ indices, infections of unknown origin were included in both the community-acquired and hospital-acquired models for these two syndromes. Any bias in these 'unknown origin' infections was adjusted for using a binary fixed-effect representing an 'unknown origin' infection, and predictions were generated for the community- and hospital-acquired infections only.

Section 5.4 Modelling framework

The data were analysed using a meta-analytic mixed effects structure. The main model can be specified as follows:

$$logit(y_i) = X_i\beta + u_i 1 + \epsilon_i, \qquad \epsilon_i \sim N(0, \Sigma_i), \quad u_i \sim N(0, \gamma) \qquad (5.4.1)$$

where

- y_i contains CFRs for data source i
- Design matrix X_i contains as columns the following covariates
    - in all models:
        - HAQ Index
        - dummy-coded indicator for age group
        - dummy-coded ICU indicator for data source (1 if data source only compiles information on ICU patients, 0 if a mix between ICU/non-ICU patients)
    - in 'interaction' and 'intercept' models:
        - dummy-coded indicator for pathogen
    - in 'interaction' models only:
        - interaction between pathogen and HAQ Index (product of dummy-coded pathogen columns and HAQ Index)
    - in models evaluating community/hospital acquired infection (LRI+, UTI):
        - dummy-coded variable indicating source of infection (1 if unknown source, 0 if community OR hospital acquired, depending on whether the model is evaluating community or hospital infections)
- β are fixed effect multipliers
- ϵ_i are observation error terms with known variances
- u_i are data source-specific random intercepts with unknown covariance γ

The underlying program used to fit the model (meta-regression, Bayesian, regularized, trimmed [MR-BRT]) is described elsewhere.[11] The program allows specification of priors on γ and β.

- Prior on γ, data source random effect: Many input data-sources cover only a single country, leading to low variability in HAQ Index within each data-source. Such collinearity adversely influenced the accuracy of the estimated effect of HAQ Index, which was instrumental in extrapolating trends from the input data to global results. To emphasise the contribution of HAQ Index over data-source in the modelled estimates, we implemented a strong Gaussian prior (mean 0, standard error 0.001) on γ.
- Prior on β for HAQ Index: There were a handful of cases in which the estimated effect of HAQ Index on CFRs given our data was clinically implausible. For skin and neonatal bloodstream infections, we had very limited data from low HAQ Index locations, with available data indicating a very intense influence of HAQ Index. Initial model results for these syndromes indicated more than 10-fold higher CFRs in low HAQ Index countries relative to high HAQ Index countries. To attenuate the effect of HAQ Index in these models we implemented a Gaussian prior on the HAQ Index β with mean 0 and standard error 0.2.

  Similarly, the peritoneal and intra-abdominal infection CFR models did not have enough input data from low HAQ Index countries to estimate a sensible HAQ Index-CFR trend; initial models indicated very strong positive associations between CFR and HAQ Index such that the CFR in low HAQ Index countries were nearly zero. To amend this, we implemented a Gaussian prior on the HAQ Index β with mean equal to the coefficient estimate for HAQ Index in the adult BSI models. The standard error for this prior was 0.2.

  For the urogenital infection models (which were ran separately for community- and hospital-acquired infections) and those for hospital-acquired lower respiratory infections, there was substantial collinearity between HAQ Index and the indicator variable for infections of unknown origin; data that did not indicate the origin of infection were generally sourced from countries with much lower HAQ Indices and much higher baseline CFRs. To emphasize the attribution of this effect to HAQ Index, rather than 'unknown infection origin,' we implemented a Gaussian prior on the HAQ Index β. The mean of this prior was centered at a value estimated for the coefficient of HAQ Index on CFR from weighted simple linear regression, with the weights equal to the inverse of the standard error of the CFRs. The standard error for this prior was 0.2.

Table 5.4.1: Number of data points and parameters estimated in each case fatality ratio model

Infectious syndrome	Sub-model	CFR model type	Data points (source-location-years)	Estimated parameters
CNS	-	Interaction	2094	13
CNS	-	Intercept	2756	15
CNS	-	Other	2756	7
Intra-abdominal	-	Intercept	639	12
Intra-abdominal	-	Other	639	6
LRI+	Community-acquired	Intercept	10485	23
LRI+	Community-acquired	Other	10485	8
LRI+	Hospital-acquired	Intercept	10122	23
LRI+	Hospital-acquired	Other	10122	8
Skin	-	Intercept	1866	15
Skin	-	Other	1897	6
Bone+	-	Intercept	432	12
Bone+	-	Other	432	5
UTI	Community-acquired	Intercept	1596	20
UTI	Community-acquired	Other	1596	7
UTI	Hospital-acquired	Intercept	1844	20
UTI	Hospital-acquired	Other	1844	7
BSI	Neonatal	Intercept	1413	18

BSI	Neonatal	Other	1271	3
BSI	Non-neonatal	Interaction	7468	24
BSI	Non-neonatal	Intercept	10842	25
BSI	Non-neonatal	Other	10842	6
Diarrhoea	-	Intercept	4041	14
Diarrhoea	-	Other	3525	6

BSI = Bloodstream infections. CNS = Meningitis and other bacterial central nervous system infections. LRI+ = Lower respiratory infections and all related infections in the thorax. Intra-abdominal = Peritoneal and intra-abdominal infections. Skin = Bacterial infections of the skin and subcutaneous systems. UTI = Urinary tract infections and pyelonephritis. Bone+ = Infections of bones, joints, and related organs.

Section 5.5 Predictions and uncertainty

Predictions for 2019 CFRs were generated for each country, age group, and pathogen as a function of each country's HAQ Index, assuming mixed ICU/non-ICU patients and, in the case of models for UTI and LRI+, that the infection was community- or hospital-acquired (in contrast to infections of unknown origin). For pathogens with insufficient data to estimate a syndrome-specific CFR, we predicted out using the 'other bacteria' CFR associated with the infectious syndrome. Importantly, all of the CFRs we calculate by infectious syndrome are independent of that syndrome's underlying cause.

Uncertainty estimates were generated using asymptotic uncertainty intervals. Specifically, for the model, the posterior uncertainty for the coefficients β is Gaussian, with mean and variance given below:

$$\hat{\beta} = \left(\sum_i X_i^T V_i^{-1} X_i\right)^{-1} \left(\sum_i X_i^T V_i^{-1} y_i\right) \qquad (5.5.1)$$

$$Var(\hat{\beta}) = \left(\sum_i X_i^T V_i^{-1} X_i\right)^{-1} \qquad (5.5.2)$$

where

$$V_i = 11^T + \hat{\gamma} I \qquad (5.5.2)$$

The variance-covariance matrix was used to obtain 1000 draws for the coefficients, which are then used to get intervals for the predictions.

Section 6: Pathogen distribution

Section 6.1: Input data

With this model, we aimed to estimate the distribution of pathogens causing each infectious syndrome. To get input data for this model, we gathered all available data sources described in section 2 that meet the following criteria:

- Sufficient diagnosis (for patient- or admission-level datasets) or sample specimen type (for isolate- or culture-level datasets) information for us to determine the infectious syndrome
- Information on which pathogen(s) caused the infection or which pathogen(s) were detected in an infectious sample, as determined through culture or genomic-based methods
- Did not have a strongly biased sampling framework across pathogens (for example, did not deliberately sample until 100 cases of every pathogen of interest had been obtained)

The input data source types that met these criteria were:

- Multiple causes of death data
- Hospital discharge
- Linkage data
- Microbial data with and without outcome information
- Literature studies from the aetiology literature reviews
- Mortality surveillance (Child Health and Mortality Prevention Surveillance [CHAMPS])

From these sources combined, there was a total of 30.4 million isolates and cases. Table S6 (section 14) provides a detailed breakdown of this total by pathogen.

Section 6.2: Data processing

Section 6.2.1: Extraction and standardisation

We extracted and standardised the location, year, age, sex, diagnoses, specimen type, pathogens, and hospital- and community-acquired (HAI and CAI) status of each record in every dataset. HAI or CAI status in microbial data was determined as described in section 2.3, while in MCoD, hospital discharge, and linkage data, a record was considered CAI if the infectious syndrome was the primary or underlying diagnosis and HAI otherwise, as described in section 4. These datasets report a variety of metrics, including deaths, admissions, cases, cultures, and isolates. While these metrics are not completely comparable (for example, a single patient may often have multiple cultures taken during a single hospital admission), we chose to standardise them into two categories: “deaths,” for any unit associated with an outcome of death, and “cases,” for any unit regardless of outcome. We assigned a unique identifier, sample ID, to track each unique unit of analysis whenever a dataset included enough line-level data to make this possible. We did not track the relationship between sample ID and patient or admission, in many cases because this was not possible; an improvement to future analyses may be to track this information and account for multiple isolates or cultures from a single admission. The majority of the data informing culprit pathogen were from microbiological analysis of various isolates, but we also considered antigen testing, such as the urinary strep antigen, and polymerase chain reaction (PCR)-based testing when assigning the pathogen responsible for infection.

Section 6.2.2: Assigning infectious syndrome

After standardising the data, we mapped every sample ID or tabulated figure in the data to infectious syndrome based on its diagnoses and specimen type. Infectious syndrome was assigned first based on any diagnosis associated with a given sample ID or tabulated figure. For samples IDs or tabulated figures with multiple diagnoses and/or an underlying diagnosis, we followed the rules laid out in section 4 for assigning infectious syndrome based on multiple causes. If a dataset contained no diagnoses or the diagnoses provided no information on infectious syndrome, we assigned infectious syndrome based on specimen type (table 6.2.2.1). This is an imprecise method because a patient may have a sample taken from an organ system that is not the site of their primary infection, most commonly from the blood. Finally, if neither diagnosis nor specimen information provided information on infectious syndrome, we assigned infectious syndrome based on pathogen for a select number of pathogens (table 6.2.2.2).

Table.6.2.2.1: Syndrome assignment based on standardised specimen types

Standard specimen	Assigned to syndrome
Blood	Bloodstream infections
Bone & joint	Infections of bones, joints, and related organs
Catheter	Bacterial infections of the skin and subcutaneous systems
Cerebrospinal fluid	Meningitis and other bacterial central nervous system infections
Gastrointestinal tract & bowel	Diarrhoea
Urinary tract infection	Urinary tract infections and pyelonephritis
Intra-abdominal	Peritoneal and intra-abdominal infections
Rectal/stool	Diarrhoea
Lower respiratory	Lower respiratory infections and all related infections in the thorax
Skin	Bacterial infections of the skin and subcutaneous systems
Upper respiratory	Other infections
Urogenital	Other infections
Other and unspecified specimens	No infectious syndrome

Table 6.2.2.2: Syndrome assignment based on pathogen for entries lacking diagnostic and specimen information

Pathogen	Assigned to syndrome
Salmonella Typhi	Typhoid, paratyphoid, and invasive non-typhoidal Salmonella
Salmonella Paratyphi	Typhoid, paratyphoid, and invasive non-typhoidal Salmonella
Salmonella Typhi or Paratyphi	Typhoid, paratyphoid, and invasive non-typhoidal Salmonella
Non-typhoidal *Salmonella* species	Typhoid, paratyphoid, and invasive non-typhoidal Salmonella
Mycobacterium tuberculosis	Tuberculosis
Neisseria meningitidis	Central nervous system infections
Neisseria gonorrhoeae	Gonorrhoea and chlamydia

Section 6.2.3: Contaminants and no aetiology detected

Some pathogens cause disease so rarely or are so commonly contaminants that we considered them to be contaminants, unlikely to be the true cause of disease. Examples include many *Corynebacterium* species and *Staphylococcus epidermidis*. We dropped all such contaminants from the analysis, as well as any record listed by treating clinicians in the data as a contaminant. Contaminants are identified at the most-detailed species or serotype level reported in the data; thus, in the broad pathogen categories that are eventually modelled, like fungi in LRI+, specific contaminant species have already been removed.

We also dropped from the analysis all records where no pathogen was detected, or the patient diagnosis indicated an unspecified bacterium. This assumes that the distribution of pathogens among cases with known aetiology are the same as those with unknown aetiology; in other words, that the probability of detection is the same for every pathogen. This assumption may break down if certain pathogens are more difficult to detect than others, or in cases where a pathogen is irregularly tested for within a laboratory.

Section 6.2.4: Polymicrobial infections

A single infection may be caused by multiple bacteria, and the co-occurrence of several bacteria can have significant effects on the treatment and outcome of disease. Some of our line-level data sources report multiple pathogens per individual record, allowing us to quantify the extent of polymicrobial infection. Other data sources tabulate over pathogen with no linking clinical information, thereby masking this information, or do not report the co-occurrence of additional bacteria.

For data sources where multiple pathogens were listed per sample ID, we classified these cases according to the following criteria. First, if a case contained more than one of “unspecified bacteria,” “virus,” “fungus,” and another pathogen(s), we chose to drop all these pathogens except the one(s) most likely to be responsible for disease, with the following ranking from most to least likely:

1. Another pathogen(s)
2. Unspecified bacteria
3. Virus
4. Fungus

This was to drop co-occurrence profiles that we consider to be uninformative, like a viral infection co-occurring with a fungal infection. For example, for a sample ID with pathogens *Escherichia coli*, *Acinetobacter baumannii*, and a virus, we would drop the virus and retain both the *E. coli* and *A. baumannii*. After applying this drop, we considered any sample ID that contained more than one pathogen to be polymicrobial. Polymicrobial was treated as a distinct pathogen category in all further analysis. We did not estimate the exact composition of the pathogens comprising the polymicrobial category for a given infectious syndrome. Furthermore, it can be difficult to determine the clinical meaning of a polymicrobial result, as it is unclear which pathogen or if any pathogen is ultimately responsible for the infection. For these reasons, we were unable to include any AMR burden from polymicrobial infections in our final results. This possibly underestimates the burden of AMR by hiding infections caused by resistant pathogens of interest in the polymicrobial category.

By standardising all datasets that report polymicrobial infections into distinct mono-pathogen and poly-pathogen categories, we created an inconsistency between these datasets and datasets that do not report the co-occurrence of pathogens. For example, a dataset that reports the co-occurrence of *E. coli* and *A. baumannii* would be standardised into three groups, mono-*E.coli*, mono-*A. baumannii*, and co-occuring *E. coli* and *A. baumannii*, while a dataset that reports *E. coli* and *A. baumannii* separately would have two categories that both have some unknown overlap. In order to allow us to use both data types, we chose to assume that the relative prevalences of pathogens in datasets that do not report co-occurrence would be comparable to their mono-pathogenic counterparts in datasets that do report co-occurrence. This assumes that the co-occurrence of pathogens is random and is not correlated for certain pathogens. We did not have sufficient data to fully test the validity of this assumption, given that few datasets report the full universe of pathogens which may co-occur.

Section 6.2.5: Selecting pathogens for estimation
For each infectious syndrome, we selected roughly 10–20 pathogens to estimate explicitly in the pathogen distribution based on the following criteria:

- The prevalence of each pathogen in the raw data
- Clinical knowledge about the primary etiologies of each infectious syndrome
- The amount of available data, which limits the number of pathogens that can be estimated successfully

In addition to the n pathogens for a given syndrome that we estimate explicitly, we also included an "other specified pathogens" category for every infectious syndrome, to which we mapped all other aetiologies identified in the data. Thus, the set of estimated pathogens for each infectious syndrome is mutually exclusive and collectively exhaustive of all possible aetiologies. Polymicrobial infections were either estimated explicitly or included in the "other" category, making all explicitly estimated individual pathogens mono-pathogenic. In addition to these criteria, we also considered the following factors:

- Since we were ultimately interested in estimating the burden of AMR in bacteria, we erred on the side of estimating bacteria with strong evidence of AMR, rather than bacteria with low evidence of AMR or non-bacterial aetiologies.
- Clinically relevant aetiologies differ from syndrome to syndrome, and we were unable to estimate all pathogens explicitly in every syndrome due to a lack of data. Therefore, the "other" pathogen category is composed of slightly different pathogens for every infectious syndrome, and can occasionally contain pathogens that are explicitly estimated for another infectious syndrome. We attempted to mitigate this by including bacteria with strong evidence of AMR in the estimation of all infectious syndromes whenever possible.
- We included enough explicitly estimated pathogens to ensure that the "other" category remained below 10% for all infectious syndromes.

For a list of pathogens covered in each infectious syndrome model, please refer to table 6.3.2.

Section 6.2.6: Estimating unbiased other and polymicrobial categories
One of the central challenges of estimating pathogen distributions was that not every data source tested for or reported every possible aetiology of a given infectious syndrome. For example, many literature studies on the aetiologies of meningitis only report on bacterial aetiologies. Some surveillance systems, like the US Centers for Disease Control and Prevention (CDC) Active Bacterial Core surveillance (ABCs), only collect data on certain pathogens of interest. Only certain pathogens are referenced explicitly in the International Classification of Diseases (ICD), limiting which pathogens can be identified from ICD-based data types like MCoD and hospital discharge. Finally, some datasets reported only a subset of the pathogens that we are interested in for a given infectious syndrome, reporting the remaining aetiologies in an aggregate "other" category. These practices have led to inconsistencies in the "other" and "polymicrobial" categories across data sources. Datasets can either over or under-report "other," and datasets that report fewer specific pathogens will automatically report fewer polymicrobial infections.

To address this problem, we maintained a list of data sources that we believe have sufficient testing and reporting to give unbiased estimates of other and polymicrobial for all syndromes. We dropped any data on polymicrobial or other that did not come from these data sources. These data sources all had a complete sampling framework (eg, they do not limit the scope of aetologies that they test for) and reported their results without any deliberate aggregation. While we believe this list provided an accurate starting place for the estimation of other and polymicrobial, future work to improve this method would involve a more detailed analysis of sampling framework and reporting categories in each dataset, specific to each infectious syndrome.

There were two major exceptions to this method for handling "other specified pathogens." First, determining the pathogenic aetiology of LRI with microbiology represents challenges that have been well described previously.[25,26] In order to account for this limitation, we utilised a vaccine probe design to inform the *Streptococcus pneumoniae* cause fraction of LRI, consistent with the approach used in the GBD aetiology estimation process.[27,28] In brief, we

extracted the vaccine efficacy of the pneumococcal vaccine against all pneumonia from 18 vaccine probe studies with randomised-control trial, before-after, and cohort designs among children and adults. We then calculated the PAF of pneumonia due to *S. pneumoniae* in each study ($Strep\ Base\ PAF$) based on these vaccine efficacies ($VE_{all\ pnuemonia}$), the vaccine efficacy of pneumococcal vaccine against vaccine-type pneumococcal pneumonia as pooled from three studies (two in children and one in adults) (VE_{vtpp}), the percentage of the population covered by the pneumococcal vaccine as modelled in GBD (100% for RCTs) (Cov_{PCV3}),[29] and the percent of serotypes covered by the vaccine[30] ($Cov_{serotype}$) (equation 6.2.6.1). We modelled a global age-specific PAF for *S. pneumoniae* based on these data in the MR-BRT environment and finally adjusted this PAF based on the vaccine coverage in children in every GBD location in 2019 and optimal vaccine efficacy in children ($Strep\ Final\ PAF$) (equation 6.2.6.2). In adults (age 5+), we assumed the effects of vaccination on adults would be primarily indirect from vaccination in children, and included an adjustment factor on the vaccine efficacy to account for this, derived from Grijalva et al.[31]

$$Strep\ Base\ PAF = \frac{VE_{all\ pneumonia}}{VE_{vtpp}Cov_{PCV3}Cov_{serotype}} \quad (6.2.6.1)$$

$$Strep\ Final\ PAF = \frac{Strep\ Base\ PAF\left(1 - Cov_{PCV3}Cov_{serotype}VE_{PCV3\ Optimal}\right)}{1 - (Strep\ Base\ PAF)Cov_{PCV3}Cov_{serotype}VE_{PCV3\ Optimal}} \quad (6.2.6.2)$$

In this vaccine probe analysis, ($1 - Strep\ Final\ PAF$) is not consistent with the “other” category in our model, since it includes all non-*S. pneumoniae* aetiologies. We retained all of the data from the vaccine probe analysis as two categories, *S. pneumoniae* and “not *S. pneumoniae*” and addressed the inconsistencies between them and our other data using our modelling framework.

The second major exception involves several literature studies on the proportion of neonatal bacterial meningitis caused by *Streptococcus agalactiae* (Group B *Streptococcus;* GBS). We found that these literature studies were important to our estimation of the pathogen distribution of neonatal meningitis, which is distinct from other age groups because of its high proportion of GBS. However, these studies either only reported or were only extracted with two categories, GBS and “other bacterial, not GBS.” We retained both these categories and addressed the inconsistencies between them and our other data using our modelling framework.

Section 6.2.7: Age-sex splitting

We standardised age and sex across all datasets to the following most-detailed groups using the GBD causes of death age-sex splitting algorithm for age:[1] 0–6, 7–27, and 28–364 days, and 1–4, 5–9, 10–14, 15–19, 20–24, 25–29, 30–34, 35–39, 40–44, 45–49, 50–54, 55–59, 60–64, 65–69, 70–74, 75–79, 80–84, 85–89, 90–94, 95+ years; and sex: male and female. This algorithm is based on the assumption that age-sex pattern of the death or case rate for a given infectious syndrome or pathogen is inherent to the pathology of the disease and is therefore constant across location and year.

To apply the algorithm, we first calculated distinct age-sex weights for every infectious syndrome and pathogen, separately for deaths and cases. These weights are the aggregate death and case rates across all datasets that report every detailed age-sex group. If we were to use a dataset that only reported some of the detailed age-sex groups, then the unreported age-sex groups would be biased downwards in the weight distribution. Calculating rates based on raw data counts could lead to extremely low rates, since we are typically comparing the entire population of a given location-year to deaths or cases captured within a single study, hospital, or surveillance system. Since the age-sex splitting algorithm only relies on the relative distribution of the weights, however, rather than their absolute level, this bias ultimately had no effect. For any infectious syndrome or pathogen combination for which we did not have enough data to create plausible age-sex weights, we used a set of all-pathogen weights for that infectious syndrome instead.

Since we split cases and deaths independently, it is possible for a detailed age-sex group produced by the splitting algorithm to contain fewer cases than deaths. When this occurred, we capped the deaths to match the cases. For

future improvement, a possible solution to this problem may be to split deaths, survivors, and cases without indication of outcome separately.

Section 6.2.8: Standardising measures

The input data sources reported a variety of combinations of measures, including some that reported deaths only, some that reported cases only, and some that reported both cases and deaths. In order to standardise these measures to cases, we estimated infectious syndrome- and pathogen-specific CFRs (see section 5) and used these CFRs to convert all deaths-only datasets to cases. For any infectious syndrome or pathogen combination for which we did not have enough data to estimate plausible CFRs, we used a set of all-bacteria CFRs for that infectious syndrome instead. All modelling was done in case space.

Several of our microbial databases came exclusively from ICUs and were therefore heavily biased towards severe illness. In order to mitigate this bias, we dropped all information on cases in ICU-only datasets and recalculated implied cases based on reported deaths and our CFRs. No similar adjustment was made to attempt to account for biases between hospitalised and un-hospitalised populations, although we did account for HAI versus CAI for two infectious syndromes—LRI and thorax infections and UTI—within our modelling framework. The use of hospital-based data to calculate both pathogen-specific case fatality ratios and pathogen distributions biases our estimate of the distribution of pathogens in incident cases towards more severe disease, particularly for less-severe infectious syndromes like lower respiratory infections; adjusting for this bias would improve the accuracy of our non-fatal estimates

Section 6.2.9: Year adjustments

Over the course of this study, we received a total of approximately 57 000 individual records from 2020 from seven microbial and mortality surveillance data sources. Additionally, we found a total of eight literature studies on pathogen distributions from 1975–1979 that were appropriate for inclusion. We included these 2020 and 1975–1979 data as inputs into our case fatality ratio and pathogen distribution models with the nearest year of covariate estimates available (2019 and 1980 respectively) in order to maximise data availability. While the COVID-19 pandemic had a significant impact on LRI disease burden in 2020, we found a total of only seven viral LRI and thorax infections reported in 2020 in our input data, and so we believe that the inclusion of these data did not significantly skew our estimates of the pathogen distribution of LRI in 2019.

Section 6.3: Modelling framework

Section 6.3.1: Overview

To model the distribution of pathogens for each infectious syndrome, we developed a method for the multinomial estimation of partial and compositional observations (MEPCO). We assumed that the aetiologies of a given infectious syndrome followed a multinomial distribution. Due to inconsistencies in which pathogens are tested for and reported by different data sources, each data source contained partial observations of the possible outcomes of the underlying multinomial distribution. Certain data sources like the vaccine probe estimates and the GBS neonatal meningitis studies represent compositional observations, where pathogens like "not *S. pneumoniae*" and "other bacterial, not GBS" represent aggregates of more detailed pathogens.

In order to use both partial and compositional data, we constructed a network model with the dependent variable as the log ratio of cases between different pathogens and estimated over a flexible parameterisation of multinomial parameters using a maximum likelihood approach. Consider a given infectious syndrome with a multinomial distribution of n mutually exclusive, collectively exhaustive aetiologies with probabilities $p = (p_1, \ldots, p_n)$, so that each $p_j \in (0,1)$ and $\sum_j p_j = 1$. The likelihood of an observation of $c = (c_1, \ldots, c_n)$, where c_j = number of cases of pathogen j in a total sample of N infections ($\sum_j c_j = N$), is:

$$P(c|p) = N! \prod_{j=1}^{n} \frac{p_j^{c_j}}{c_j!} \qquad (6.3.1.1)$$

We modelled the probabilities using a composition of a link function with a linear predictor:

$$p_{i,j} = \exp\left(x_{i,j}^T \beta_j\right) \tag{6.3.1.2}$$

for observations i, a vector of covariates $x_{i,j}$, and a vector of coefficientsβ_j for each pathogen j. Table 6.3.2 shows the covariates used for infectious syndrome model; a typical specification included an intercept term, HAQ Index, a categorical age group dummy for large age bins, and any relevant vaccine coverage proportions by country. However, we did not observe these probabilities directly. Rather, we observed ratios between sums of these probabilities, which reduce to ratios between sums of cases within each study. These observations therefore take the form:

$$y_i = \frac{cases\ of\ pathogen\ A}{cases\ of\ pathogen\ B} = \frac{\sum_{j=1}^{n} w_{i,j}^a \exp\left(x_{i,j}^T \beta_j\right)}{\sum_{j=1}^{n} w_{i,j}^b \exp\left(x_{i,j}^T \beta_j\right)} \tag{6.3.1.3}$$

where $w_{i,j}^a$ is a weight of 0 or 1 that selects the mutually exclusive, collectively exhaustive most-detailed pathogens that make up observed pathogen A, which may be a composite observation. For example, for the "other bacterial, non-GBS" pathogen, $w_{i,j}$ would be 1 for *Staphyloccocus aureus, S. pneumoniae, Haemophilus influenzae, Neisseria meningitidis, Listeria monocytogenes, K. pneumoniae, E. coli*, and other pathogens and 0 for GBS and virus. We dropped all observations where either the numerator or denominator had 0 observed cases in order to make this calculation and a forthcoming log transform possible. This may bias the model towards overestimating less common pathogens.

It is not possible to infer all coefficients β_j from the observations, since they are all relative. However, if we fix all of the coefficients for one pathogen to 0 as a reference group, then we obtain a well-posed inverse problem, as long as there is enough data to estimate the remaining coefficients. Without loss of generality, we assumed $\beta_1 = 0$ for all elements and obtain estimates of the remaining $\beta_2, \ldots, \beta_n$ by minimising the sum of the residuals between log-transformed observations y and corresponding log-transformed predictions from equation 6.3.1.3:

$$\min_{\beta_2,\ldots,\beta_n} f(\beta) \coloneqq \sum_i \frac{1}{\sigma_i^2}\left[\ln(y_i) - \ln\left(\sum_{j=1}^{n} w_{i,j}^a\, exp\left(x_{i,j}^T \beta_j\right)\right) + \ln\left(\sum_{j=1}^{n} w_{i,j}^b\, exp\left(x_{i,j}^T \beta_j\right)\right)\right]^2 \tag{6.3.1.4}$$

where σ_i^2 are variances corresponding to the data points. Equation 6.3.4 is a nonlinear likelihood minimisation problem that that we optimised using a standard implementation of the Gauss-Newton method.[32] We then re-normalised the optimal coefficients to obtain final predictions of the probabilities of each pathogen:

$$p_{i,j} = \frac{\exp\left(x_{i,j}^T \beta_j\right)}{\sum_{\hat{j}} \exp\left(x_{i,\hat{j}}^T \beta_{\hat{j}}\right)} \tag{6.3.1.5}$$

To quantify the uncertainty of this estimate, we used asymptotic statistics to obtain the posterior distribution of $(\beta_2, \ldots, \beta_n)$. Specifically, using the Gauss-Newton Hessian approximation gave us the asymptotic information matrix for all β_j except for the reference pathogen, allowing us to sample draws of $\beta = (\beta_1 = 0, \beta_2, \ldots, \beta_n)$. For each β draw and given feature x, we obtained a corresponding draw of p using equation 6.3.1.5.

Finally, to convert $p_{i,j}$ for a given demographic group i from case space to deaths space, we transformed using our CFR estimate for demographic i:

$$p_{i,j}^{deaths} = \frac{p_{i,j} \times CFR_i}{\sum_{\hat{j}} p_{i,\hat{j}} \times CFR_i} \tag{6.3.1.6}$$

This network regression with covariates framework allowed us to use partial and composite data that reported on one or only a few pathogens, or that reported multiple pathogens aggregated together. Networks, however, can be unstable with sparse data and stable estimates have in some cases required the use of Bayesian priors in these models. In particular, we imposed Gaussian priors with mean 0 and non-zero variance on all coefficients except

intercepts, to bias the model away from spurious effects driven by data sparsity. These priors were based on expert opinion and can improved with further empirical validation in the future. Table 6.3.4 provides a list of these priors.

Table 6.3.2: Pathogens assessed, covariates, and age groups for each infectious syndrome

Infectious syndrome	Pathogens assessed	Model covariates	Age groups
Bloodstream infections	Acinetobacter baumannii, Citrobacter spp., Enterobacter spp., Enterococcus faecalis, Enterococcus faecium, other enterococci, Escherichia coli, fungus, group A Streptococcus, group B Streptococcus, Klebsiella pneumoniae, Neisseria meningitidis, non-typhoidal Salmonella, polymicrobial, Proteus spp., Pseudomonas aeruginosa, Salmonella Typhi, Serratia spp., Staphylococcus aureus, Streptococcus pneumoniae	HAQ Index,[14] age group, age-standardised proportion of intravenous drug use,[23] proportion coverage by PCV3 vaccine,[33] indicator variable for Europe	Neonatal, Post-neonatal–5, 5–50, 50–70, 70+
Infections of bones, joints, and related organs	Enterococcus faecalis, Enterococcus faecium, other enterococci, Escherichia coli, group A Streptococcus, group B Streptococcus, Klebsiella pneumoniae, Pseudomonas aeruginosa, Staphylococcus aureus	HAQ Index, age group	Under 5, 5–50, 50–70, 70+
Endocarditis and other cardiac infections	See bloodstream infection pathogens	Not explicitly modelled. Pathogen distribution for bloodstream infections is used.	Neonatal, Post-neonatal–5, 5–50, 50–70, 70+
Diarrhoea	Adenovirus, Aeromonas spp., Amebiasis, Campylobacter spp., Clostridium difficile, cryptosporidium, enteropathogenic Escherichia coli, enterotoxigenic Escherichia coli, non-typhoidal Salmonella, norovirus, rotavirus, Shigella spp., Vibrio cholerae	Not modelled here. GBD diarrhoea aetiology estimates are used.	GBD most detailed age groups
Lower respiratory infections and all related infections in the thorax	Acinetobacter baumannii, Chlamydia spp., Enterobacter spp., Escherichia coli, fungus, group B Streptococcus, Haemophilus influenzae, Klebsiella pneumoniae, Legionella spp., Mycoplasma spp., polymicrobial, Pseudomonas aeruginosa, Staphylococcus aureus, Streptococcus pneumoniae, virus	HAQ Index, proportion coverage by PCV3 vaccine, proportion coverage by Hib3 vaccine,[33] age group, HAI/CAI	Neonatal, Post-neonatal–5, 5–50, 50–70, 70+
Meningitis and other bacterial central nervous system infections	Escherichia coli, group B Streptococcus, Haemophilus influenzae, Klebsiella pneumoniae, Listeria monocytogenes, Neisseria meningitidis, Staphylococcus aureus, Streptococcus pneumoniae, virus	HAQ Index, proportion coverage by PCV3 vaccine, proportion coverage by Hib3 vaccine, age group, proportion of population covered by ’10-’15 MenAfriVac rollout[1,34]	Neonatal, Post-neonatal–5, 5-50, 50-70, 70+
Peritoneal and intra-abdominal infections	Citrobacter spp., Enterobacter spp., Enterococcus faecalis, Enterococcus faecium, Escherichia coli, Klebsiella pneumoniae, other Klebsiella species, Proteus spp., Pseudomonas aeruginosa, Serratia spp., Staphylococcus aureus	HAQ Index, age group	Under 5, 5–50, 50–70, 70+
Bacterial infections of the skin and subcutaneous systems	Acinetobacter baumannii, Enterobacter spp., Enterococcus faecalis, other enterococci, Escherichia coli, group A Streptococcus, group B Streptococcus, Klebsiella pneumoniae, Proteus spp., Pseudomonas aeruginosa, Staphylococcus aureus	HAQ Index, age group	Under 5, 5–50, 50–70, 70+
Urinary tract infections and pyelonephritis	Acinetobacter baumannii, Citrobacter spp., Enterobacter spp., Enterococcus faecalis, Enterococcus faecium, other enterococci, Escherichia coli, group B Streptococcus, Klebsiella pneumoniae, Morganella spp., Proteus spp., Providencia spp., Pseudomonas aeruginosa, Serratia spp., Staphylococcus aureus	HAQ Index, age group, HAI/CAI	Under 5, 5–50, 50–70, 70+

Group A *Streptococcus* = *Streptococcus pyogenes.* Group B *Streptococcus* = *Streptococcus agalactiae.* HAQ Index = Healthcare Access and Quality Index. HAI/CAI = hospital-acquired infection/community-acquired infection.

Table 6.3.3: Number of data points and parameters in each pathogen distribution model

Infectious syndrome	Subtype	Number of data points	Number of parameters
LRI+		158967	135
BSI		126417	180
Skin		1105	55
CNS	Neonatal	25615	81
CNS	Post neonatal	25579	81
UTI		23662	96
Bone+		1870	45
Intra-abdominal		2458	55

BSI = Bloodstream infections. CNS = Meningitis and other bacterial central nervous system infections. LRI+ = Lower respiratory infections and all related infections in the thorax. Intra-abdominal = Peritoneal and intra-abdominal infections. Skin = Bacterial infections of the skin and subcutaneous systems. UTI = Urinary tract infections and pyelonephritis. Bone+ = Infections of bones, joints, and related organs.

Table 6.3.4. Gaussian prior standard deviations for non-intercept coefficients for each pathogen distribution model

Infectious syndrome	Sub-type	Gaussian prior standard deviation
BSI		0.1
CNS	Neonatal	0.1
CNS	Non-neonatal	0.1
LRI+		0.1
Intra-abdominal		0.3
Skin		0.3
UTI		0.02 for *A. baumannii*/HAQ Index coefficient<br>0.1 for all others
Bone+		0.5

BSI = Bloodstream infections. CNS = Meningitis and other bacterial central nervous system infections. LRI+ = Lower respiratory infections and all related infections in the thorax. Intra-abdominal = Peritoneal and intra-abdominal infections. Skin = Bacterial infections of the skin and subcutaneous systems. UTI = Urinary tract infections and pyelonephritis. Bone+ = Infections of bones, joints, and related organs.

Section 6.4: Exceptions and special handling

There were several notable exceptions and special handling decisions made for each individual pathogen distribution model. We hope to address many of these exceptions with more sustainable methods in our future work.

Section 6.4.1: Cardiac infections

For cardiac infections, we used the pathogen distribution for bloodstream infections rather than estimating specific distributions for these syndromes, due to a lack of complete literature reviews on the aetiologies and case-fatality rates of these syndromes. We consider this to be a serious limitation of our methodology, but do not anticipate that is seriously impactful on our final estimates, since cardiac infections are the third-lowest syndrome by deaths and years lived with disability (YLDs), comprising just 1.33% (95% UI 1.02–1.67) of all deaths associated with AMR.

Section 6.4.2: Diarrhoea

In diarrhoea patients, cultures of specimens taken from the gastrointestinal tract, bowels, rectum, or stool are almost always affected by contaminants or pathogens that are not the cause of diarrhoea. For this reason, we believe that our input data and modelling framework are not able to accurately capture the aetiologies of diarrhoea. We chose to use GBD estimates of the aetiologies of diarrhoea in deaths instead of running our own model.[35] These estimates are based on the odds ratio of having diarrhea given the detection of a pathogen, obtained from the Global Enteric Multicenter Study, therefore removing the influence of any pathogen that does not increase the risk of diarrhea.

A major limitation of using this study is that the GBD diarrhoea aetiology estimates are population attributable fractions (PAFs) for each pathogen. These PAFs may add to greater than 1 and the authors made no attempt to quantify the extent of co-occurrence of pathogens. This is inconsistent with the pathogen distribution estimation method used in our study, which quantifies polymicrobial infections and estimates all pathogens as mono-infections. In order to avoid duplication of cases in our framework, we had to make some assumptions about the co-occurrence of pathogens in diarrhoea. We chose to normalise the PAFs to 1 for any demographic where the sum of GBD diarrhoea aetiology PAFs was greater than 1. This assumed that co-occurrence of pathogens was random and that

the "other" pathogens category was negligible in these demographics. We made no adjustment to demographics where the PAFs added to less than 1. To convert the fatal PAFs to a distribution of aetologies in incidence, we rescaled the distribution according to our estimates of the pathogen-specific case fatality ratios of diarrhea, calculated as described in section 5.

Section 6.4.3: Bacterial infections of the skin and subcutaneous systems

Certain skin and subcutaneous samples are easily affect by contaminants, colonization, and other pathogens that are not the cause of infection. For this reason, we considered microbial data and mortality surveillance to be too difficult to extract meaningful aetiology information from, and instead used only ICD-coded databases (multiple cause of death, hospital discharge, and linkage data) and literature studies as inputs into our model of the pathogen distribution of skin infections.

Section 6.4.4: Lower respiratory infections and all related infections in the thorax

We dropped all data on *S. pneumoniae* for community-acquired LRI and thorax infections in non-neonatal age groups except our estimates from the vaccine probe analysis. Our model also predicted a high fraction of polymicrobial in neonates for community-acquired infections for this infectious syndrome based off of only 1 study, CHAMPS. We found this to be implausible and so dropped polymicrobial from the estimates for this age group in community-acquired infections and renormalized the proportions for all other pathogens to 1.

Section 6.4.5: Peritoneal and intra-abdominal infections

Because dedicated anaerobic cultures were not routinely performed for peritoneal samples, we dropped all anaerobes observed in the data for and excluded anaerobes as an etiology of intra-abdominal infections.

Section 6.4.6: Meningitis and other bacterial central nervous system infections

Due to the unique pattern of meningitis in neonates, particularly the high prevalence of GBS, we modeled neonatal and adult central nervous syndrome infections separately.

Section 6.4.7: Infectious syndromes not modelled

For three infectious syndromes, we did not run a pathogen distribution model. These syndromes are all caused by distinct pathogens whose individual burdens are already estimated in GBD as separate causes of death. For these syndromes, we simply used GBD estimates (table 6.4.6.1)

Table 6.4.6.1: Infectious syndromes for which we used GBD estimates to obtain the pathogen distribution

**Infectious syndrome**	**Pathogens**	**GBD causes**
Typhoid, paratyphoid, and invasive non-typhoidal Salmonella	*Salmonella* Typhi	Typhoid fever
	Salmonella Paratyphi	Paratyphoid fever
	Non-typhoidal *Salmonella*	Invasive non-typhoidal Salmonella
Tuberculosis	*Mycobacterium tuberculosis*	Tuberculosis
Gonorrhoea and chlamydia	*Neisseria gonorrhoeae*	Gonococcal infection
	Chlamydia trachomatis	Chlamydial infection

Section 6.5: Model validation

To assess model validity, we calculated the root mean square error (RMSE) and coefficient of determination (R^2) for each pathogen distribution model in proportion space for both in-sample and out-of-sample predictions (Table 6.5.1). Proportions were predicted for each observation using the specific denominator observed from that study. For example, if a given study reported on only *E. coli* and *S. pneumoniae*, the predictions for model validation for this study were calculated as proportions of the total for *E. coli* and *S. pneumoniae*. In order to calculate out-of-sample fit, we perform non-exhaustive cross-validation, with each round of the validation holding out 1 country of data at a time. This leave-one-country-out approach simulates the prediction task of estimating the pathogen distribution of a country for which we have no data.

R^2 ranges from 0.784 to 0.867 in-sample and from 0.755 to 0.837 out of sample, indicating good model fit with only modest losses when data are moved out of sample. RMSE ranges from 0.129 to 0.149 in-sample and from 0.141 to

0.159 out of sample. Given that the data are expected to vary from the model predictions according to the observation-level variance, and the fact that the RMSEs are relatively consistent between in-sample and out-of-sample, these RMSEs are reasonable. Overall, these metrics show that these models have good fit and good out-of-sample predictive ability.

Table 6.5.1 In-sample and out-of-sample validation metrics for pathogen distribution models

Infectious syndrome	Model type	R^2		RMSE	
		In sample	Out of sample	In sample	Out of sample
Bacterial infections of the skin and subcutaneous systems		0.808	0.771	0.129	0.141
Bloodstream infections		0.822	0.785	0.128	0.141
Infections of bones, joints, and related organs		0.858	0.837	0.141	0.151
Lower respiratory infections and all related infections in the thorax		0.810	0.780	0.142	0.153
Meningitis and other bacterial central nervous system infections	Neonatal	0.858	0.803	0.134	0.158
	Non-neonatal	0.867	0.822	0.129	0.150
Peritoneal and intra-abdominal infections		0.815	0.812	0.147	0.148
Urinary tract infections and pyelonephritis		0.784	0.755	0.149	0.159

Out of sample metrics calculated using leave-one-country-out cross validation

Section 7: Prevalence of resistance

Section 7.1: Input data

We identified line level and aggregate data on the prevalence of resistance in bacterial pathogens, which were linked to the country and year in which the infection was acquired, from datasets obtained from pharmaceutical companies, surveillance networks, academic institutions, and individual hospitals (see section 2). In total, we gathered over 52.8 million test results for the 88 pathogen–drug combinations we assessed. Table S7 provides a detailed breakdown of this total by pathogen–drug combination.

We supplemented microbiological data with systematic reviews following the Preferred Reporting Items for Systematic Reviews and Meta-Analyses (PRISMA) guidelines,[36] to collect resistance data published from countries and territories where surveillance systems do not routinely collect data to ensure extensive coverage of the pathogen–drug combinations thought to contribute the greatest burden of drug resistant infections, which we termed core pathogen–drug combinations (table 7.2.1). Data on the prevalence of AMR in these pathogen–drug combinations were extracted from published literature and compiled into comprehensive datasets. The systematic reviews followed similar methodologies; a detailed description can be found either in published literature (*S.* Typhi and *S.* Paratyphi[37]) or in the corresponding PROSPERO records (*E.* coli, *K. pneumoniae, S. aureus* and *S. pneumoniae* PROSPERO registration CRD42019145148; *Shigella* species PROSPERO registration CRD42019127603; iNTS PROSPERO registration CRD42020189935; *N. gonorrhoeae* SPF unique identifier osf.io/4vy5n). The *S.* Typhi and *S.* Paratyphi A systematic review was expanded to include non-blood culture isolates for the current analysis.

Forms were created, and screening and data extraction were completed using web-based systematic review software (DistillerSR, Evidence Partners, Ottawa, Canada) for all pathogens except *Salmonella*, for which a smaller number of manuscripts were identified.

To more comprehensively account for the burden of AMR in bacteria, we also estimated the prevalence of resistance for 71 supplementary pathogen–drug combinations for which we did not conduct a systematic literature review. Data for these supplementary combinations were extracted from the datasets obtained from pharmaceutical companies, academic institutes, and individual hospitals using the same processing procedure as was used for the core pathogen–drug combinations. The list of supplementary combinations is presented in table 7.2.2.

For the prevalence of drug resistance in *Mycobacterium tuberculosis* for multi-drug resistance (MDR, characterised by isoniazid and rifampicin co-resistance) excluding extensive drug resistance (XDR, characterised by resistance to isoniazid, rifampicin, and fluoroquinolone, as well as either aminoglycosides or capreomycin) and XDR, we used previously published GBD results.[1] Notably, GBD MDR excluding XDR TB estimates and the MDR/rifampin mono-resistant TB estimate from WHO differ, primarily because HIV/TB cases are included as part of WHO TB estimates. GBD adjusts the miscoding of deaths cause by HIV and TB in locations with high prevalence of both diseases, such as South Africa, assigning more deaths to HIV/TB (which are attributed to HIV) and these methodological differences lead to lower MDR TB mortality across GBD burden estimates. An additional difference in estimates for MDR is that WHO includes rifampicin mono-resistance as part of their MDR TB figures.

Section 7.2: Data processing

The prevalence of resistance for each pathogen–drug combination was calculated for each data source, by country and year. Whenever possible, we classified resistance using the most recent CLSI guidelines based on the MICs provided in the data. When MICs were unavailable, we deferred to lab interpretation to classify the isolates. All isolates determined to have intermediate resistance were classified as resistant. To determine the prevalence of resistance to a class of antibiotics (eg, fluoroquinolones), resistance to any one of the antibiotics in the class was sufficient to classify an isolate as resistant for line level data (ie, susceptibility data for individual isolates). For aggregate data (ie, the proportion of isolates resistant to various antibiotics), the highest prevalence of resistance to any antibiotic in the class was selected. Multidrug resistance in *Salmonella* species was defined as concurrent resistance to ampicillin/amoxicillin, chloramphenicol, and trimethoprim-sulfamethoxazole; and fluoroquinolone resistance was defined as ciprofloxacin minimum inhibitory concentration of 0.125μg/ml or higher, or nalidixic acid resistance (CLSI breakpoint for *Salmonella* spp. were updated in 2012 to include 0.125 μg/ml as isolates with 'decreased ciprofloxacin susceptibility', and we have considered these as resistant). Nalidixic acid resistance was also used as a proxy for fluoroquinolone non-susceptibility for *Shigella* species.

Table 7.2.1: Core pathogen–drug combinations

Pathogen	Antimicrobial
Escherichia coli	Third-generation cephalosporins<br>Fluoroquinolones
Klebsiella pneumoniae	Third-generation cephalosporins<br>Carbapenems
Staphylococcus aureus	Methicillin
Streptococcus pneumoniae	Penicillin
Salmonella Typhi & Paratyphi A	Multidrug resistance<br>Fluoroquinolones
Invasive non-typhoidal *Salmonella*	Fluoroquinolones
Shigella species	Fluoroquinolones
Neisseria gonorrhoeae	Third-generation cephalosporins
Mycobacterium tuberculosis	Isoniazid mono-resistance, Rifampicin mono-resistance

Table 7.2.2: Supplementary pathogen–drug combinations

Pathogen	Antimicrobial
Acinetobacter baumannii	Aminoglycosides, Anti-pseudomonal penicillin/Beta-lactamase inhibitors, Beta-lactam/Beta-lactamase inhibitors, Carbapenems, Third-generation cephalosporins, Fourth-generation cephalosporins, Fluoroquinolones
Citrobacter species	Aminoglycosides, Anti-pseudomonal penicillin/Beta-lactamase inhibitors, Carbapenems, Third-generation cephalosporins, Fourth-generation cephalosporins, Fluoroquinolones
Enterobacter species	Aminoglycosides, Anti-pseudomonal penicillin/Beta-lactamase inhibitors, Carbapenems, Fourth-generation cephalosporins, Fluoroquinolones, Trimethoprim-Sulfamethoxazole
Enterococcus faecalis	Fluoroquinolones, Vancomycin
Enterococcus faecium	Fluoroquinolones, Vancomycin
Enterococcus species	Fluoroquinolones, Vancomycin

Escherichia coli	Aminoglycosides, Aminopenicillin, Beta-lactam/Beta-lactamase inhibitors, Carbapenems, Trimethoprim-Sulfamethoxazole
Group A *Streptococcus*	Macrolide
Group B *Streptococcus*	Fluoroquinolones, Macrolide, Penicillin
Haemophilus influenzae	Aminopenicillin, Third-generation cephalosporins
Klebsiella pneumoniae	Aminoglycosides, Beta-lactam/Beta-lactamase inhibitors, Fluoroquinolones, Trimethoprim-Sulfamethoxazole
Morganella species	Third-generation cephalosporins, Fourth-generation cephalosporins, Fluoroquinolones
Neisseria gonorrhoeae	Fluoroquinolones
Proteus species	Aminoglycosides, Aminopenicillins, Third-generation cephalosporins, Fluoroquinolones, Trimethoprim-Sulfamethoxazole
Pseudomonas aeruginosa	Aminoglycosides, Anti-pseudomonal penicillin/Beta-lactamase inhibitors, Carbapenems, Third-generation cephalosporins, Fourth-generation cephalosporins, Fluoroquinolones
Serratia species	Aminoglycosides, Anti-pseudomonal penicillin/Beta-lactamase inhibitors, Carbapenems, Third-generation cephalosporins, Fourth-generation cephalosporins, Fluoroquinolones
Staphylococcus aureus	Fluoroquinolones, Macrolide, Trimethoprim-Sulfamethoxazole, Vancomycin
Streptococcus pneumoniae	Beta-lactam/Beta-lactamase inhibitors, Carbapenems, Third-generation cephalosporins, Fluoroquinolones, Macrolide, Trimethoprim-Sulfamethoxazole

Group A *Streptococcus* = *Streptococcus pyogenes*. Group B *Streptococcus* = *Streptococcus agalactiae*

To account for biased level of resistance found in tertiary care settings, we reviewed all input data used for the prevalence of resistance estimation and classified each data source as either tertiary, non-tertiary, or unknown/mixed designation, which was a commonly used classification for large resistance surveillance networks which don't report on the hospitals they collect data from. We located datasets that either provided facility information at the line-level or reported samples from exclusively tertiary or non-tertiary facilities. Where possible, we used tertiary/non-tertiary assignments from the data providers. When no assignments were available, we classified sites as tertiary, primary, and secondary by following the definitions provided by Jamison et al.[38] in table 7.2.3. We first considered hospital name when classifying. If the name did not include any of the terms listed in table 7.2.3, we searched the facility website for self-designations of tertiary/non-tertiary (most preferred), number of specialties, and bed-size. We classified facilities with vague names and with no websites or websites with insufficient information as "mixed/unknown"; data from these facilities could contain both tertiary and non-tertiary samples. Finally, we grouped primary and secondary facilities together in the non-tertiary category.

Table 7.2.3: Definitions and terms for different levels of hospital

**Disease Control Priorities Project: terminology and definitions**	**Alternative terms commonly found in the literature**
Primary-level hospital: few specialties—mainly internal medicine, obstetrics and gynecology, pediatrics, and general surgery, or just general practice; limited laboratory services available for general but not specialized pathological analysis	District hospital<br>Rural hospital<br>Community hospital<br>General hospital
Secondary-level hospital: highly differentiated by function with 5 to 10 clinical specialties; size ranges from 200 to 800 beds; often referred to as a provincial hospital	Regional hospital<br>Provincial hospital (or equivalent administrative area such as county)<br>General hospital
Tertiary-level hospital: highly specialized staff and technical equipment— for example, cardiology, intensive care unit, and specialized imaging units; clinical services highly differentiated by function; could have teaching activities; size ranges from 300 to 1,500 beds	National hospital<br>Central hospital<br>Academic or teaching or university hospital

For systematic review data collected from sub-Saharan Africa, we referred to Maina et al.[39] who identified and defined health facilities at each service delivery level (primary to tertiary) in sub-Saharan Africa using both information from health sector policies and strategic plans for each country in the region. They also undertook further comparative/validation analyses to cross reference the completeness/robustness of their classifications against the corresponding number of facilities reported at each level in the most current country-level health sector strategic plans and other health sector reports. Using this hierarchy by country (please see online table 2 in Maina et al.) we classified facilities in sub-Saharan Africa from the systematic review data as tertiary versus non-tertiary.

The proportion of data classified as originating from a tertiary facility differed substantially by super region, ranging from 0.3% of cases in the high-income super-region to 25.0% of cases in sub-Saharan Africa; this stark difference reaffirmed the importance of adjusting the data. To create robust inputs for the crosswalk, data were aggregated by source, year, tertiary/non-tertiary status, and super-region. Because there was no reliable way to determine the mix of hospital types in mixed/unknown data, this data was grouped with non-tertiary. We chose to cluster this data with non-tertiary rather than omit it, as, for some super-regions, the proportion of definitively non-tertiary data was very low (e.g., 0.05% for high-income). After aggregating the data in this way, we created a set of matched pairs, matching every tertiary data point to non-tertiary data for the same pathogen–drug combination from the same super-region collected within 5 years from one another.

Because the degree of bias in resistance between tertiary and non-tertiary data could vary across different parts of the world, we ran a separate crosswalk for each super region and pathogen–drug *super group* combination. Certain bacteria and antimicrobials were clustered into super groups to provide the models with more robust input data, though, crucially, while a given model would contain several pathogen–drug combinations in its inputs, every matched pair was made comparing tertiary and non-tertiary values for the same combination. Bacteria were classified as follows:

Table 7.2.4: Pathogens in each pathogen super group

Pathogen super group	Incorporated pathogens
Gram-negatives	*Enterococcus faecalis, Enterococcus faecium, Enterococcus* spp., Group A *Streptococcus*, Group B *Streptococcus, Staphylococcus aureus, Streptococcus pneumoniae*
Enterobacterales	*Citrobacter* spp., *Enterobacter* spp., *Escherichia coli, Haemophilus influenzae, Klebsiella pneumoniae, Proteus* spp., *Serratia* spp.
Pseudomonadales	*Acinetobacter baumannii, Pseudomonas aeruginosa*

Notably, some pathogens were excluded from the tertiary crosswalk procedure because it was believed that infections with such pathogens would be robust to tertiary care bias. These pathogens were: *Mycobacterium tuberculosis*, *Neisseria gonorrhoeae*, non-typhoidal *Salmonella*, *Salmonella paratyphi*, *Salmonella typhi*, and *Shigella* spp. Data for *Morganella* spp. was not crosswalked as there was no input data for that pathogen from tertiary facilities.

Only one group of antimicrobials was clustered to create an antimicrobial super group, the β-lactam group, which was comprised of: aminopenicillin, anti-pseudomonal penicillin, β-lactamase inhibitors, carbapenems, third and fourth generation cephalosporins, methicillin, and penicillin. All other antibiotic classes (aminoglycosides, fluoroquinolones, macrolides, sulfanoamides, and vancomycin) each individually comprised their own antimicrobial super group.

To allow us to implement linear models, resistance values were logit-transformed. We used the delta method to compute the standard error of the prevalence of resistance in logit space. To incorporate data with zero resistance, or with complete resistance, we applied a 0.1% offset, such that the prevalence of resistance for data with zero resistance was represented as 0.1% and the prevalence of resistance for data with total resistance was represented as 99.9%. We then used the MR-BRT modelling framework to estimate the logit difference of tertiary and non-tertiary data for each super region-pathogen/antimicrobial ‘super combination,’ including a random effect for each pathogen–drug combination within the super combination and employing a positivity prior to enforce the constraint that the tertiary data exceed or be equal to the non-tertiary data. For the super region-pathogen/antimicrobial super combinations with sparse input data (fewer than 250 matched pairs) we instead used global estimated logit

differences between tertiary and non-tertiary data for that pathogen/antimicrobial super combination, employing the same positivity prior.

After modelling the difference between tertiary and non-tertiary data, we implemented the models to adjust all the country-level tertiary input data that was indicated as biased. We then used the adjusted prevalence of resistance estimates from tertiary care facilities and unadjusted prevalence of resistance from non-tertiary/mixed care facilities as data inputs for the prevalence of resistance models. As was done before, resistance values were offset prior to logit-transformation to allow the use of linear models; data with zero resistance or complete resistance was offset by 2%. Exceptions to this offset were made for two combinations, *Staphylococcus aureus*/vancomycin and Group B *Streptococcus*/penicillin, which were anticipated to often have values beneath 2% resistance. For these combinations, we applied a 0.5% offset instead.

Section 7.3: Modelling framework

The prevalence of AMR in each pathogen–drug combination was modelled separately. For the core combinations, excluding *N. gonorrhoeae*/3GC, we selected a range of spatially- and temporally-explicit health and socio-demographic-related covariates with biologically plausible associations to the prevalence of AMR in each pathogen from the Global Health Data Exchange (http://ghdx.healthdata.org/), and from published literature.[3] This list was narrowed down by fitting a lasso penalised regression model between the data and the covariates for each dataset (using the 'glmnet' package version 3.0.2 in R version 3.6.1) and selecting the most influential covariates in each of the pathogen–drug models to be taken forward. For the supplementary pathogen–drug combinations and *N. gonorrhoeae*/3GC, we utilised a standard set of covariates for all models: HAQ Index, pigs per capita (as a proxy for antibiotic use in animal husbandry), mean temperature, and antibiotic consumption of the antibiotic class relevant to each pathogen–drug combination. Determining more individualised sets of covariates for each of these supplementary pathogen–drug combinations is an ongoing focus for future extensions of this research.

Due to the high heterogeneity of the input datasets, we outliered data points found to have the most extreme values for the prevalence of resistance. An initial generalised linear model (GLM) was fit to the data and covariates and input data points that lay outside of two times the median absolute deviation from the modelled estimate for each location were determined to be outliers and removed. The GLM was fit with nested random effects based on the GBD super-region, region, and country or territory to capture spatial effects, and was fit using the 'lme4' package version 1.1-21 in R version 3.6.1.

After the removal of extreme values, the datasets were used to fit spatiotemporal statistical models of the prevalence of AMR. Firstly, we used a stacked ensemble model to fit the associations between selected covariates and data. For each of the pathogen–drug combinations, we considered the following child models for inclusion: generalised additive models (GAM), penalised regression models (elastic-net, ridge, lasso), random forest, cubist, and neural-networks. Models were fit in R version 3.6.1, using the packages 'CARET' version 6.085, 'mgcv' version 1.8.31, and 'glmnet' version 3.0.2. We fit the child models using five-fold cross validation for each combination and selected the best performing, non-correlated child models based on the out-of-sample predictive performance (final covariates for each pathogen–drug combination are shown in table S8). We then calculated the R^2-weighted mean of the estimates of the child models, constraining the coefficients to sum to one, and used these ensemble estimates to fit a spatiotemporal Gaussian process regression (ST-GPR) model for each pathogen–drug combination.

ST-GPR is described in detail elsewhere.[1] In brief, spatial and temporal weights were applied to the residuals of the stacked ensemble model; these were then added to the modelled estimates to smooth them in time and space. A Gaussian process regression (GPR) was then fit, and the mean prevalence of AMR was calculated from 1000 draws of the GPR for each location and year with endemic disease. The 1000 draws of the model were taken through to the next stage of calculations to propagate uncertainty throughout.

Section 7.4: Covariates

Appendix table S8 shows all of the covariates used to model the core pathogen–drug combinations and provides citations detailing the methods used to estimate these covariates. For certain covariates, we provide a brief summary of the estimation method below.

Mean temperature: Temperature data are obtained from Climatic Research Unit, University of East Anglia.[40] For each GBD geography, the temperature and population datasets (rasters) are cropped to just cover said geography. The population weighted mean temperature is calculated for each pixel of data in that geography, and then those values are aggregated to produce a single value for the geography. This is repeated for each country-year.

Pigs per capita: Data on the global distribution of pigs were obtained from the Food and Agriculture Organization of the United Nations.[41,42] The raw pig count raster is cropped to GBD geography. Total number of pigs is calculated by summing all pixels in that geography, the result is then divided by the total population of that geography to calculate pigs per capita. This is repeated for each country-year.

Population density: First, population density per pixel was calculated by dividing the population count per pixel by the land area per pixel. The result raster is then thresholded for those pixels where there are more than 1000 people per sq km. The result is multiplied by population, and then the area summed to get a total population in a geography in that bin. Finally this value is divided by total population in the geography to get the final proportion result.

Oral rehydration: Data on oral rehydration, defined as the proportion of children under 5 who had diarrhea in the last 2 weeks who received oral rehydration (ORS) treatment, were collected from multi-country population health surveys such as the Demographic and Health Surveys (DHS), Multiple Indicator Cluster Surveys (MICS), Reproductive Health Surveys (RHS), and Living Standards Measure Study (LSMS) survey, as well some country-specific surveys (see http://ghdx.healthdata.org/gbd-2019/data-input-sources). Only nationally representative datasets are included, and the most extreme 10% of the data is removed from each quintile of Socio-demographic Index (SDI) or HAQ Index. A forward stepwise method is used to select covariates, resulting in SDI and antenatal care (1 visit) coverage being selected. Finally, ST-GPR is run using a mixed effects stage 1 regression with fixed effects on SDI and antenatal care coverage and random effects for super-regions, regions, and countries. Predictions are made for all GBD geographies.

Section 7.5: Resistance profiles

To accurately assess the burden associated with resistance to each antibiotic, we needed to first understand the landscape of multidrug-resistant bacteria, for which the burden would be shared across several antibiotics. We therefore estimated, for each bacteria studied, a set of 'resistance profiles' characterized as the probabilities for each possible combination of resistance/susceptibility for all of the antibiotics analyzed. For example, for a bacterium for which we assessed three antibiotics, we would estimate eight probabilities: SSS, SSR, SRS, RSS, SRR, RSR, RRS, and RRR (S – susceptible, R – resistant). These probabilities encompass the entire set of possibilities of resistance for the bacterium, and sum to 1.

For a pathogen for which we assessed n antibiotics, resistance profiles were estimated by optimising over a $2^n - 1$-dimensional probability simplex with $\frac{n(n+1)}{2}$ linear constraints. Every such set of resistance profiles corresponds to a full specification of a multivariate binomial distribution. The target set of constraints were as follows:

- The inferred marginal probability of resistance for each antibiotic (the prevalence of resistance to an antibiotic irrespective of all others analyzed) exactly matches the estimates from our prevalence of resistance models. Since there are n antibiotics, this set comprises n constraints.
- The inferred pairwise likelihood of co-resistance for each pair of antibiotics exactly matches the likelihood inferred from the marginal probability of each antibiotic in the pair, and the Pearson correlation of resistance between the two antibiotics observed across all of the laboratory data we compiled. These represent $\frac{n^2-n}{2}$ additional constraints.

The input format for these constraints for an example case with n = 3 is shown in figure 7.5.1.

Figure 7.5.1: Example input matrix for calculating resistance profiles for a pathogen with 3 antibiotic classes (A,B,C)

Prev(A)	Prev(A&B)	Prev(A&C)
-	Prev(B)	Prev(B&C)
-	-	Prev(C)

Prev(X): prevalence of resistance of antibiotic X from ST-GPR model, by location and draw

Prev(X&Y): prevalence of resistance in both X and Y, back calculated from Prev(X), Prev(Y) and the Pearson correlation of X&Y in the lab data with multiple resistance screens

In the *n*=2 case, the number of constraints in our framework (3) is equal to the number of unknowns in the probability simplex ($2^n - 1 = 3$), and therefore at most one set of resistance profiles is possible. For all larger values of *n*, however, the number of unknowns exceeds the number of constraints, and there are infinite potential resistance profiles. Thus, our resistance profiles are generated by solving for a single sample from the probability simplex formed under the established constraints of marginal resistance and co-resistance.

There is no a priori guarantee that the observables generate a feasible solution. To prevent the constraints from delineating an infeasible probability simplex (for example, an input suggesting the individual resistances to antibiotics A and B are both above 90% but the probability of co-resistance to A and B is below 10%), we solved an optimization problem that identified, for each input matrix, the closest feasible set of input constraints and a corresponding set of resistance profiles that fits these constraints. The 1-simplex in any dimension is specified by

$$\Delta \coloneqq \{p: \quad 0 \le p_i \le 1,\ \sum p_i = 1\} \qquad (7.5.1.1)$$

Each marginal observation and each pairwise co-resistance corresponds to a linear constraint, where a sum over a subset of the p in the simplex should be a given value v_i:

$$\mathrm{m_i^T} p = v_i \qquad (7.5.1.2)$$

where $\mathrm{m_i}$ is a ‘mask vector’ of zeros and ones, used to pick out the appropriate summands. Overall, there are $\frac{n(n+1)}{2}$ such affine constraints. The optimisation problem we solve is to find the nearest feasible simplex given these constraints:

$$\min_{p\in\Delta} f(p) \coloneqq \sum_{i=1}^{n(n+1)/2} \frac{1}{\sigma_i^2}(\, m_i^T p - v_i)\hat{}2 \qquad (7.5.1.3)$$

Where $\frac{1}{\sigma_i^2}$ can be used to provide importance weights for the data. This is a least squares problem with linear equality and inequality constraints (corresponding to the simplex), and can be solved very efficiently even for relatively large n (such as 10 co-occurring antibiotic classes). The result is guaranteed to return the probability simplex closest to the specified constraint, even if the original set of constraints is infeasible, and corresponding set of resistance profiles that fits this nearest simplex.

To propagate uncertainty, we repeat this procedure for each of the 1,000 draws we estimate for prevalence of antibiotic resistance. To generate the *i*-th draw of our resistance profiles, we input the *i*-th draw of the marginal probability of resistance for each antibiotic analyzed for a given pathogen into the probability simplex optimization algorithm. Updating the marginal probabilities of resistance in turn influences the probabilities of co-resistance, and each element of the input we feed the algorithm is unique to the *i*-th draw. The optimization is also initialized randomly for every draw. This process is implemented for each GBD country, resulting in 1000 resistance profiles for each country for each pathogen in our analysis.

It is important to note that while we produce resistance profiles unique to each country, the Pearson correlations of co-resistance that we derive from the input data are assumed to be constant across location, sex, and infectious syndrome. Due to data sparsity, we cannot currently identify co-resistance patterns in several locations (particularly LMICs) with insufficient or non-existent line-level data; indeed, the data sources providing multiple resistance tests for a single isolate are among the most detailed of those we collected for this research and require exceptional data quality standards that are not easily achieved throughout the world. Identifying differences in patterns of co-resistance by location or infectious syndrome is of considerable interest in the future.

Section 7.6: Model validation

Validation of prevalence of resistance modelling occurs in two instances. For the ensemble estimates, machine-learning candidate models are validated using five random holdout sets, and we select models correlated below a Pearson correlation coefficient threshold of 0.8 which showed the best performance based on the R^2 predictive validity for the out-of-sample predictions. These intermediary results are not reported in this paper because they do not pertain to the final prevalence of resistance estimate.

We then validate the entire ensemble ST-GPR process by calculating in-sample and out-of-sample accuracy metrics. Accuracy is measured as the proportion of correctly classified resistant/susceptible isolates based on the modelled estimate and the raw data's prevalence of resistance. As a written example, if there were 10 isolates with 50% resistance in the raw data and the model predicted 60% resistance for that location, we would have 5 correctly classified resistant samples (true positives), 1 incorrectly classified resistant sample (false positive), and 4 correctly classified susceptible samples (true negatives), for 90% accuracy. For out-of-sample cross-validation, we withheld, at the outset of the ensemble modelling process, a set of countries with data as a holdout group: for the core-combinations we withheld 20% of countries each iteration, for 5 total holdout sets, while for the supplementary-combinations we withheld 10% of countries each iteration, for 10 holdout sets. By holding out all of the data for a set of countries, our out-of-sample accuracy metrics reflect the potential model fit we have for countries that have no input data in the entire prevalence of resistance process. Table 7.6.1 reports the accuracy metric for each pathogen–drug combination. Our in-sample accuracy values range from 77.2% to 99.8%, while our out-of-sample accuracy values range from 57.1% to 99.7%.

Table 7.6.1: In-sample and out-of-sample accuracy estimates for prevalence of resistance models

Pathogen	Antibiotic class	IS accuracy	OOS accuracy
Acinetobacter baumannii	3GC	0.98	0.892
Acinetobacter baumannii	4GC	0.964	0.88
Acinetobacter baumannii	AG	0.772	0.645
Acinetobacter baumannii	Anti-pseudomonal	0.96	0.816
Acinetobacter baumannii	BL-BLI	0.969	0.636
Acinetobacter baumannii	CP	0.901	0.768
Acinetobacter baumannii	FQ	0.964	0.821
Citrobacter spp.	3GC	0.987	0.922
Citrobacter spp.	4GC	0.991	0.974
Citrobacter spp.	AG	0.993	0.959
Citrobacter spp.	Anti-pseudomonal	0.984	0.912
Citrobacter spp.	CP	0.995	0.973
Citrobacter spp.	FQ	0.989	0.967
Enterobacter spp.	4GC	0.982	0.941
Enterobacter spp.	AG	0.989	0.976
Enterobacter spp.	Anti-pseudomonal	0.984	0.939
Enterobacter spp.	CP	0.988	0.973
Enterobacter spp.	FQ	0.991	0.98
Enterobacter spp.	TMP-SMX	0.996	0.883
Enterococcus faecalis	FQ	0.982	0.856
Enterococcus faecalis	Vanco	0.993	0.987
Enterococcus faecium	FQ	0.992	0.985
Enterococcus faecium	Vanco	0.976	0.703
Escherichia coli	3GC	0.977	0.973
Escherichia coli	AG	0.979	0.96
Escherichia coli	Aminopenicillin	0.959	0.915
Escherichia coli	BL-BLI	0.932	0.909
Escherichia coli	CP	0.988	0.972
Escherichia coli	FQ	0.983	0.98
Escherichia coli	TMP-SMX	0.907	0.903
Group A *Streptococcus*	Macrolide	0.987	0.863
Group B *Streptococcus*	FQ	0.993	0.731
Group B *Streptococcus*	Macrolide	0.986	0.907
Group B *Streptococcus*	PCN	0.997	0.992
Haemophilus influenzae	3GC	0.995	0.981
Haemophilus influenzae	Aminopenicillin	0.958	0.849
Klebsiella pneumoniae	3GC	0.981	0.985
Klebsiella pneumoniae	AG	0.983	0.901
Klebsiella pneumoniae	BL-BLI	0.979	0.832
Klebsiella pneumoniae	CP	0.987	0.992
Klebsiella pneumoniae	FQ	0.935	0.755
Klebsiella pneumoniae	TMP-SMX	0.973	0.816
Morganella spp.	3GC	0.922	0.834
Morganella spp.	4GC	0.974	0.917
Morganella spp.	FQ	0.928	0.839
Mycobacterium tuberculosis (new)	Mono INH	0.993	0.966
Mycobacterium tuberculosis (new)	Mono RIF	0.996	0.987
Mycobacterium tuberculosis (retreated)	Mono INH	0.982	0.968

Mycobacterium tuberculosis (retreated)	Mono RIF	0.994	0.966
Neisseria gonorrhoeae	3GC	0.987	0.982
Neisseria gonorrhoeae	FQ	0.972	0.841
Non-typhoidal *Salmonella*	FQ	0.911	0.967
Other enterococci	FQ	0.928	0.829
Other enterococci	Vanco	0.966	0.924
Proteus spp.	3GC	0.993	0.981
Proteus spp.	AG	0.989	0.906
Proteus spp.	Aminopenicillin	0.991	0.571
Proteus spp.	FQ	0.966	0.842
Proteus spp.	TMP-SMX	0.997	0.986
Pseudomonas aeruginosa	3GC	0.949	0.908
Pseudomonas aeruginosa	4GC	0.988	0.96
Pseudomonas aeruginosa	AG	0.977	0.95
Pseudomonas aeruginosa	Anti-pseudomonal	0.975	0.895
Pseudomonas aeruginosa	CP	0.977	0.912
Pseudomonas aeruginosa	FQ	0.986	0.95
Salmonella Paratyphi	FQ	0.839	0.74
Salmonella Paratyphi	MDR	0.962	0.96
Salmonella Typhi	FQ	0.83	0.8
Salmonella Typhi	MDR	0.87	0.77
Serratia spp.	3GC	0.984	0.946
Serratia spp.	4GC	0.992	0.951
Serratia spp.	AG	0.987	0.965
Serratia spp.	Anti-pseudomonal	0.958	0.894
Serratia spp.	CP	0.991	0.986
Serratia spp.	FQ	0.987	0.953
Shigella spp.	FQ	0.934	0.86
Staphylococcus aureus	FQ	0.984	0.924
Staphylococcus aureus	Macrolide	0.945	0.934
Staphylococcus aureus	Methicillin	0.975	0.883
Staphylococcus aureus	TMP-SMX	0.995	0.992
Staphylococcus aureus	Vanco	0.998	0.997
Streptococcus pneumoniae	3GC	0.98	0.953
Streptococcus pneumoniae	BL-BLI	0.982	0.956
Streptococcus pneumoniae	CP	0.981	0.966
Streptococcus pneumoniae	FQ	0.949	0.925
Streptococcus pneumoniae	Macrolide	0.984	0.884
Streptococcus pneumoniae	PCN	0.945	0.901
Streptococcus pneumoniae	TMP-SMX	0.97	0.884

3GC = Third-generation cephalosporins. 4GC = Fourth-generation cephalosporins. AG = Aminoglycosides. Anti-pseudomonal = Anti-pseudomonal penicillin/Beta-Lactamase inhibitors. BL-BLI = Beta Lactam/Beta-lactamase inhibitors. CP = Carbapenems. FQ = Fluoroquinolones. MDR excluding XDR in TB = Multi-drug resistance excluding extensive drug resistance in TB. MDR in S. Typhi and Paratyphi = Multi-drug resistance in Salmonella Typhi and Paratyphi. Mono INH = Isoniazid mono-resistance. Mono RIF = Rifampicin mono-resistance. PCN = Penicillin. TMP-SMX = Trimethoprim-Sulfamethoxazole. Vanco = Vancomycin. XDR in TB = Extensive drug resistance in TB.

Section 8: Relative risk

Section 8.1 Input data

The input data for the relative risk estimation step included literature data that provided relative risk of death for resistant and susceptible organisms and hospital-based microbiology surveillance data linked to outcomes, as well as other clinical parameters (eg, demographics, diagnoses). Published studies were identified from a recent meta-analysis performed by Cassini and colleagues.[43]

The data inputs for the excess duration estimates were literature data that reported on length of stay for resistant and susceptible organisms and hospital-based microbiology surveillance data that were linked to outcomes as well as various other clinical parameters (eg, demographics, diagnoses). The number of days between a positive specimen date and discharge date was used to obtain the mean duration of infection. We considered days elapsed between admission and discharge as mean duration of stay if this was the only piece of information provided in the study. We also considered median duration of infection or median duration of stay if the study only provided this piece of information.

Section 8.2: Data processing

There were 9.4 million possible samples from 73 countries to inform our relative risk of death estimates. Many of the 9.4 million potential samples were further de-duplicated as part of the modeling process (modelling details in 7.3). A detailed breakdown of this 9.4 million by pathogen–drug is in table S9 (section 14). Relative risk estimates were extracted from primary literature as were study characteristics that described the adjustments made by the study. When no adjustments were made, or an adjusted odds ratio was presented, we extracted the crude relative risk. For hospital data that contained admission diagnoses, diagnoses were mapped to GBD Level 2 causes. Admission diagnoses were mapped to GBD causes using ICD codes when provided; when admission diagnoses were free-text entries, they were mapped using two expert reviews.

Section 8.3 Modelling overview

The measure of excess risk used to estimate the fatal burden of AMR was the relative risk of death from an infection with a pathogen resistant to the antibiotic of interest as compared to an infection of the same site with the same organism that was susceptible to the antibiotic of interest. The relative risk estimate was produced after adjusting for various potential confounders including age, admission diagnosis (mapped to GBD causes), site of culture, and hospital versus community onset. Because of data sparsity, a single measure of relative risk was estimated for each pathogen–drug combination, representing a global estimate for all sites of infection and all underlying causes.

When data availability allowed it, relative risk from hospital-based microbiology surveillance data was estimated after adjusting for age, admission diagnosis, site of culture, and hospital- versus community-acquired infection, otherwise a crude relative risk was used. The adjusted estimates of relative risks were then included with the crude relative risks in a two-stage nested mixed effects meta-regression model using MR-BRT. The stage one model was a meta-regression for each antibiotic class, which was used to produce a prior for the stage two model. We considered study-specific adjustments such as age of patients, admission diagnosis, site of culture and hospital-versus community acquired infection as potential covariates to be included in the second stage. Covariate selection was based on a set of log-linear models with a range of Lasso penalty parameters, and only statistically significant covariates were selected. The stage two model was run for each antibiotic class with a random effect for pathogen and fixed effects for study level characteristics that described whether the relative risk estimate from a study or dataset adjusted for each parameter using the prior from the stage one model for the antibiotic class.

$$Relative\ Risk_{pathogen_n drug_d} = \beta_0 + \beta_d \cdot x + u_{pathogen_n} + \epsilon_d \qquad (8.3.1)$$

Where x is a bias covariate, $u_{pathogen_n}$ is a random effect for pathogen n within an antibiotic class, ϵ_K is the measurement error, d is antibiotic class and β and X are vectors of length $i + 1$ for i covariates. From this stage two model, we produced 1000 draws to estimate the relative risk of death and uncertainty attributable to resistance for each pathogen–drug combination.

For non-fatal burden estimation, we estimated the excess duration attributable to resistance—comparing the length of hospital stay for an infection with a pathogen resistant to the antibiotic of interest to an infection of the same site with the same organism that was susceptible to the antibiotic of interest. For community-acquired infections the entire duration of length of stay was attributed to the infection, for hospital-acquired infections we used the time from first positive culture to time of discharge to estimate length of stay. To address the potential confounding effect of longer admissions resulting in higher probability of acquiring resistant infections, we adjusted the relative length of stay obtained from patient level data for the number of hospital days prior to culture positivity. We observed a generally lower relative length of stay when we applied this adjustment, which was expected. We then used the same two-stage nested mixed effects meta-regression modelling framework described for fatal estimation to produce a relative length of stay attributable to resistance for each pathogen–drug combination. One exception to this estimation process was *Neisseria gonorrhoeae*, which had too little data to produce an estimate on the impact of resistance on duration of illness. As a result, we produced a YLD estimate based on the excess duration of illness for a given antibiotic class. This was a 1.29 fold increase in duration for fluoroquinolones-resistant bacteria and a 1.43 fold increase in duration for third-generation cephalosporin-resistant bacteria.

The analysis of relative risk followed the definitions of the prevalence of resistance step (section 7) as closely as possible. Both analyses identified resistance to a given antibiotics class if the isolate had an intermediate or resistant interpretation to any one of the antibiotics in that given class. But the analysis of relative risk diverged from the analysis of prevalence of resistance in the following circumstances. First, the relative risk step included molecular resistance testing if this was the only data provided by a study, eg, β-lactamase or MecA positive pathogens; this could potentially misclassify some resistant organisms as sensitive if they had an alternate mechanism for resistance, such as a porin alteration leading to carbapenem resistance. Second, the relative risk estimate produced was for sterile sites of infection, as there was limited data from non-sterile sites. Third, it was not possible to assess relative risk of multidrug-resistant pathogens because of limited data availability and because it did not fit in the modelling strategy at the antibiotic class level. Instead, the relative risk of each of the components of multidrug-resistant pathogens was calculated and the antibiotic class with the highest relative risk was used; for *Salmonella* Typhi this was relative risk to Trimethoprim-Suflamethoxazole. Fourth, we had limited availability of data on fatalities attributable to *Salmonella* Paratyphi and *Shigella* species; as a result, we used fatal relative risk estimates from *Salmonella* Typhi as a proxy. Fifth, there were limited data on fatalities attributable to resistant *N. gonorrhoeae*, so we excluded the fatal estimate for this pathogen. Finally, the relative risk of *Mycobacterium tuberculosis* was assessed for multidrug and extensively drug-resistant infections as reported previously in GBD. Estimates of relative risk of death for sterile sources of specimen across 88 pathogen–drug combinations are given in table S10.

Section 8.4 Model validation

We report three summary metrics to evaluate the relative risk of death models: the root-mean squared error (RMSE), the Mean Average Error (MAE) and the percent coverage of observed data within the full variance of the model. These three metrics were calculated using the real relative risk ratio in the whole sample of data and also by holding out 25% of the sample within antibiotic class in 4 iterations. Table 8.4.1 provides details for each of the antibiotic class model evaluated. Large MAE and RMSE values indicate that observed data deviates from the mean model estimate. We also see a large proportion of the data (82% and more) falls within the total variance of each model estimates. This indicates that large deviations from the mean estimate coincide with large variances of the data observed.

Table 8.4.1: In-sample and out-of-sample performance metrics for relative risk of death models

Antibiotic class	MAE		RMSE		Coverage	
	in-sample	out-of-sample	in-sample	out-of-sample	in-sample	out-of-sample
Vancomycin	0.71	0.65	1.33	0.94	82%	82%
Fluoroquinolones	0.73	0.75	1.61	1.52	88%	90%
Third-generation cephalosporins	0.75	0.76	1.63	1.49	93%	93%
Macrolide	0.71	0.73	1.25	1.24	95%	94%
Methicillin	0.65	0.67	1.3	1.13	96%	94%
Penicillin	0.48	0.69	0.83	0.97	96%	98%
Carbapenem	0.64	0.64	1.54	1.29	98%	97%
Aminoglycosides	0.37	0.41	0.61	0.58	100%	100%
Aminopenicillin	0.9	0.86	1.82	1.68	100%	100%
Anti-pseudomonal penicillin/Beta-Lactamase inhibitors	0.76	0.8	1.76	1.64	100%	100%
Beta Lactam/Beta-lactamase inhibitors	0.36	0.3	0.61	0.37	100%	100%
Fourth-generation cephalosporins	1.22	1.54	3.21	3.46	100%	100%
Trimethoprim-Sulfamethoxazole	0.64	0.6	1.17	0.97	100%	100%

This approach for relative risk estimation had a number of limitations; most were attributable to data sparsity. First, it is likely that the impact of resistance on mortality is different across locations. In locations where overall health-care access and quality are very poor, the impact of resistance may be smaller because the management of susceptible infections is sub-optimal. On the other hand, in locations where broad, second- and third-line antimicrobials are not available, one would expect the impact of resistance to be greater. Second, it is possible that the relative risk of death attributable to resistance is different across anatomical sites of infection because of variable penetrance of antibiotics to different anatomical locations. As we continue efforts to expand data collection and reporting, we hope to be able to address these limitations in future iterations.

Section 9: Counterfactuals and AMR estimation

Section 9.1: Estimating AMR burden with counterfactual of no infection

We computed two counterfactuals to estimate the drug-resistant burden. First, we estimated the burden of AMR using the counterfactual of no infection. We estimated the fatal burden of individual pathogen–drug combinations by taking the product of the deaths for each underlying cause, fraction of deaths related to infection, infectious syndrome fraction, fatal pathogen fraction, and fatal prevalence of resistance and then summed across all infectious syndromes and underlying causes:

$$Deaths\ with\ Resistance_{Kd} = \sum_{J}\sum_{L} D_J \times S_J \times M_{LJ} \times P_{LK} \times R_{Kd} \qquad (9.1.1)$$

where D = deaths, S = fraction related to infection, M = infectious syndrome fraction, P = fatal pathogen fraction, R = fatal prevalence of resistance, J = cause, L = syndrome, K = pathogen, d = drug. To produce an estimate of deaths with resistance to any antibiotic estimated, we employed the same formula but used the fatal prevalence of resistance to any antibiotic using the resistance profiles, described previously. We calculated the fatal prevalence of resistance R for a given drug d based on the non-fatal prevalence of resistance R' and relative risk of death RR for this drug:

$$R_{Kd} = \frac{R'_{Kd}RR_{Kd}}{(1 - R'_{kd}) + R'_{Kd}RR_{Kd}} \qquad (9.1.2)$$

We calculated the fatal prevalence of resistance to any antibiotic estimated based on the non-fatal prevalences of each resistance profile, incorporating all resistance profiles δ that are resistant to at least 1 drug with corresponding relative risks RR_{Kd^*}, determined by the method described below (section 9.2):

$$R_{K,all\ drugs} = \frac{\sum_{\delta} R'_{K\delta} RR_{Kd^*}}{\left(1 - \sum_{\delta} R'_{K\delta}\right) + \sum_{\delta} R'_{K\delta} RR_{Kd^*}} \tag{9.1.3}$$

We then estimated YLLs using standard GBD methods to convert age-sex specific deaths into YLLs.[1]

For the non-fatal estimate, we first estimated the incidence of each infectious syndrome in each underlying cause. For infectious underlying causes (table 9.1.1), we simply used the incidence estimated in GBD. For non-infectious underlying causes, we divided the infectious syndrome deaths ($D_j \times S_J \times M_{LJ}$) by the syndrome- and pathogen-specific CFRs calculated in section 5, aggregated across pathogen using the nonfatal pathogen distribution P' calculated above.

$$Incidence_{JL} = \frac{D_J S_J M_{LJ}}{\sum_K CFR_{LK} P'_{LK}} \tag{9.1.4}$$

Table 9.1.1: Infectious GBD causes for which we used GBD YLD and incidence estimates

Infectious syndrome	GBD cause
Meningitis and other bacterial central nervous system infections	Meningitis
Lower respiratory infections and other related infections in the thorax	Lower respiratory infections
Bacterial infections of the skin and subcutaneous systems	Pyoderma<br>Cellulitis<br>Decubitis ulcer
Urinary tract infections and pyelonephritis	Urinary tract infections
Bloodstream infections	Maternal sepsis<br>Neonatal sepsis
Diarrhoea	Diarrhea
Tuberculosis	Tuberculosis
Typhoid, paratyphoid, and invasive non-typhoidal Salmonella	Typhoid fever<br>Paratyphoid fever<br>Invasive non-typhoidal Salmonella
Gonorrhoea and chlamydia	Chlamydia<br>Gonococcal infection

We then took the product of the infectious syndrome incidence, the non-fatal pathogen fraction, and the non-fatal prevalence of resistance and summed across all infectious syndromes and underlying causes to get incidence with resistance for every pathogen and drug. As with the fatal estimate, to produce an estimate of incident infections with resistance to any antibiotic, we used the same formula and used the non-fatal prevalence of resistance to any antibiotic estimated from the resistance profiles.

We then calculated YLDs for each pathogen. For some GBD causes, we simply used the GBD YLD estimates and multiplied them by the corresponding nonfatal pathogen distribution (table 8.1.2) For all other causes, we multiplied together the infectious syndrome incidence, the non-fatal pathogen fraction, and a syndrome-specific YLDs per incident case rate, calculated using a proxy cause from GBD.[1] To estimate the YLDs per incident case rate, we extracted GBD incidence and YLD estimates for the proxy causes and divided the YLDs by the incidence for each age, sex, and location. Three infectious syndromes are not estimated in the GBD, and therefore have no standard sequelae or disability weights: bloodstream infections, intra-abdominal infections, and bone and joint infections. For the proxy causes for these three syndromes, we used the closest approximate disease as determined by a group of experts in infectious diseases and epidemiology (table 9.1.2). This approach is a significant limitation of the study and should be improved in future work.

Table 9.1.2. Proxy causes used to calculate YLDs per incidence case rate for each infectious syndrome

**Infectious syndrome**	**Proxy cause**
Meningitis and other bacterial central nervous system infections	Meningitis
Endocarditis and other cardiac infections	Endocarditis
Peritoneal and intra-abdominal infections	Paralytic ileus and intestinal obstruction
Lower respiratory infections and all related infections in the thorax	Lower respiratory infections
Bacterial infections of the skin and subcutaneous systems	Bacterial skin diseases
Infections of bones, joints, and related organs	Bacterial skin diseases
Diarrhoea	Diarrhoea
Bloodstream infections	Maternal sepsis – *Extrapolated to males*
Urinary tract infections and pyelonephritis	Urinary tract infections

To get the YLDs associated with resistance for each pathogen, we used the non-fatal prevalences of resistance for each drug and resistance profile and relative length of stay (LOS) for each pathogen–drug combination to calculate the fraction of YLDs associated with resistance for each pathogen, using equations analogous to equations 9.1.2 and 9.1.3. We multiplied this fraction by the YLDs for each pathogen to get YLDs associated with resistance to each pathogen–drug combination and YLDs associated with resistance any antibiotics estimated. We then added YLLs and YLDs to produce the DALY estimate for burden associated with resistance.

Section 9.2: Estimating AMR burden with counterfactual of infection with susceptible organism

For the second counterfactual—comparing resistant to susceptible infections—we calculated mutually exclusive pathogen–drug estimates. To do this, we first estimated the population attributable fraction of deaths ($Mortality\ PAF$) for each resistance profile with resistance to at least 1 drug, δ. The inputs for the PAF were the non-fatal prevalence of the given resistance profile, $R'_{K\delta}$, and the relative risk of death for resistant infection compared to susceptible infection for each drug, RR_{kd}. Because of data sparsity, we were unable to calculate the relative risk for every possible resistance profile, and so instead used the highest relative risk of all of the drugs in the resistance profile. For example, if for a resistance profile of resistant to penicillin and fluoroquinolones, the relative risk was 1.1 for penicillin and 1.4 for fluoroquinolones, we would use a relative risk of 1.4 for this profile. The mortality PAF is calculated as a multi-category exposure:

$$Mortality\ PAF_{K\delta} = \frac{R'_{K\delta}(RR_{Kd^*} - 1)}{1 + \sum_{\delta} R'_{K\delta}(RR_{Kd^*} - 1)} \qquad (9.2.1)$$

where d* is the drug in the resistance profile δ with the highest relative risk.

We then took the product of the deaths for each underlying cause, fraction of deaths related to infection, infectious syndrome fraction, fatal pathogen fraction, and the mortality PAF for each resistance profile to get the deaths attributable to resistance for every resistance profile:

$$Deaths\ due\ to\ Resistance_{K\delta} = \sum_{J}\sum_{L} D_J \times S_J \times M_{LJ} \times P_{LK} \times Mortality\ PAF_{K\delta} \qquad (9.2.2)$$

When the resistance profile described resistance to more than one antibiotic, the deaths were then distributed to the component pathogen–drug combinations based on the excess risk of the pathogen–drug combination divided by the sum of the excess risk of all pathogen–drug combinations in the resistance profile. For a resistance profile δ with resistance to drugs $i = 1, \ldots, n$:

$$Redistribution\ Weight_{Kd_i} = \frac{RR_{Kd_i} - 1}{\sum_{\hat{\imath}}(RR_{Kd_i} - 1)} \qquad (9.2.3)$$

For co-resistance amongst beta-lactam antibiotics (i.e. carbapenems, 4GC, 3GC, antipseudomonal, BL/BLI, aminopenicillins, and penicillin), we used a different approach to redistributing burden. Similar to Cassini et al., we applied a hierarchy such that the burden was categorically attributed to the broadest beta-lactam antibiotic, rather than split the burden between multiple beta-lactam antibiotics.[43] We used the hierarchy in table 9.2.1 to assign burden in the presence of co-occurring beta-lactam resistance. When a pathogen was resistant to multiple beta-

lactams and a non-beta-lactam antibiotic, we first applied the hierarchy to determine the 'highest' beta-lactam resistance and then generated redistribution weights using only the 'highest' beta-lactam and the non-beta-lactams. We then used these attributable death estimates to estimate YLLs using standard GBD methods to convert age-sex specific deaths to YLLs.

A similar approach was taken to estimate non-fatal burden for the counterfactual of antibiotic-susceptible infection. We first assumed that antibiotic resistance has no effect on the attack rate of pathogens; therefore, there are 0 incident cases attributable to resistance and all non-fatal burden comes from increased length of illness. To quantify the extent of this increased length of illness, we first produced a length of stay (LOS) PAF for each resistance profile using the non-fatal prevalence of resistance and relative LOS for resistant infections as compared to susceptible infections in a method analogous to equation 9.2.1. Because of data sparsity, we were unable to calculate the relative LOS for every resistance profile, and so instead used the relative LOS for the drug with the highest relative LOS in the profile. We then took the product of the YLDs for each infectious syndrome, the non-fatal pathogen distribution, and the LOS PAF to produce attributable YLD estimates. This assumes that the attributable LOS PAF is equally applicable to all sequelae, which is an assumption made because of a lack of data on the impact of resistance on the likelihood of different sequelae and the duration of specific sequelae. We then added YLLs and YLDs to produce an estimate of DALYs attributable to resistance.

Table 9.2.1 Beta-lactam hierarchy

Rank	Antibiotic class
1	Carbapenem
2	Antipseudomonal Penicillin/Beta-lactamase Inhibitor
3	Fourth Generation Cephalosporin
4	Third Generation Cephalosporin
5	Beta-lactam/Beta-lactamase Inhibitor
6	Aminopenicillin
7	Penicillin

Because of the optimisation approach used to derive each resistance profile, the prevalence of resistance to for a given pathogen–drug as modelled using ensemble ST-GPR (section 7.3), R'_{Kd}, will not necessarily be exactly equal to the sum of all resistance profiles $R'_{K\delta}$ that include resistance to drug d. Due to this inconsistency, in extremely rare cases, an estimate of AMR burden in the susceptible counterfactual may slightly exceed the corresponding estimate of AMR burden in the no infection counterfactual for a specific pathogen–drug. We consider the ensemble ST-GPR estimate to be more accurate than the resistance profiles, since the latter are based on Pearson correlations of multidrug resistance that are calculated from limited microdata and generalized to all locations. For this reason, we cap all individual pathogen–drug estimates of burden for the susceptible counterfactual, which are based on the resistance profiles, to the burden for the no infection counterfactual, which are based on the ensemble ST-GPR estimates.

Section 9.3: Excluded combinations

Although our approach attempted to be exhaustive and include all clinically-relevant pathogen–drug combinations, there are two combinations included in the WHO priority list for which we could not produce an estimate. The first is clarithromycin resistance in *Helicobacter pylori* and the second is fluoroquinolone resistance in *Campylobacter* species. These were excluded due to limited data availability as highlighted by a recent study in the European Union that found that, as of 2019, no member countries had implemented publicly accessible, mandatory reporting surveillance programmes for these two pathogen–drug combinations.[44] *H. pylori* and *Campylobacter* spp are commonly diagnosed without culture so resistance profiles are uncommon in passive surveillance systems. The burden of *H. pylori* is not currently estimated in GBD, though some of the consequent diseases are, like peptic ulcer disease and gastric cancer. Producing a burden estimate of *H. pylori was* outside the scope of this work, and without a pathogen burden estimate, we could not produce an estimate of the burden attributable to clarithromycin-resistant

H. pylori. In contrast, GBD does produce an estimate on the burden of *Campylobacter* spp. There were, however, too few data to produce an estimate on the excess risk of death or duration associated with fluoroquinolone resistance and limited data to inform a global prevalence of resistance estimate. Given these limitations, we did not produce burden estimates for clarithromycin-resistant *H. pylori* or fluoroquinolone-resistant *Campylobacter* spp. Because of the lack of data on risk of death associated with drug-resistant *Neisseria gonorrhoeae*, we were unable to produce an estimate of the fatal burden of resistance so produce only a non-fatal estimate. Many potential pathogen–drug combinations were excluded due to the spectrum of antimicrobial activity (ie, Vancomycin and *E. coli*), intrinsic resistance (eg, BL/BLI resistance in *Pseudomonas aeruginosa*) or resistance that is exceedingly common (eg, penicillin resistance in *S. aureus*); these combinations were decided by a group of experts in infectious diseases, microbiology, epidemiology, and population health. There was insufficient data to produce a global estimate for many pathogen–drug combinations of interest, such as aminopenicillin resistance in *Enterococcus* spp, fluoroquinolone resistance in *Acinetobacter baumannii*, or colistin resistance in any pathogen estimated. This is largely due to either a lack of regional data to inform the prevalence of resistance component or a lack of microbial data linked to outcomes to inform the measure of excess risk component. A final constraint was the computational burden of estimating more than seven antibiotic classes for a single pathogen. Because of the approach to co-resistance described in section 7.4, each antibiotic class added led to an exponential increase in the computation needs and anything above seven antibiotic classes was not tenable. As additional data are made available, we plan to add clinically relevant combinations and iterate on the computational approach so that we can describe the burden of bacterial AMR more comprehensively.

Section 10. Analysis of total AMR burden decomposed by infection and resistance

Appendix table S11 shows our estimates of the fatal burden of AMR by GBD region, decomposed into several key components: all-cause mortality, the fraction of all deaths that involve infection (infection fraction), the fraction of deaths involving infection that are associated with resistance (prevalence of resistance), and the fraction of deaths involving infection that are attributable to resistance (PAF). The number of deaths associated with resistance is the product of all-cause deaths, infection fraction, and resistance-associated fraction, while the number of deaths attributable to resistance is the product of all-cause deaths, infection fraction, and resistance-attributable fraction. Decomposing our results into these components shows how the burden of AMR is variously influenced by overall regional death rates, the prevalence of infectious disease, and the prevalence of resistance in different regions of the world.

While the regions of sub-Saharan Africa have among the highest death rates per 100,000 person-years associated with and attributable to AMR in the world, the majority of this burden is driven by the high rates of infectious disease in these regions, rather than the prevalence of resistance itself. The GBD regions of central, eastern, southern, and western sub-Saharan Africa have the four leading infectious fractions, at 48.0% (95% UI 40.2 - 56.1), 50.7% (43.1 – 58.8), 45.6% (38.4 – 52.7), and 53.5% (45.4 – 62.0) respectively, but also have the bottom 4 fraction of infectious deaths that are associated with AMR, 27.0% (24.3 – 30.1), 27.9% (25.6 – 30.1), 18.6% (16.6 – 20.9), and 28.3% (25.4 – 30.8) respectively. In contrast, the three regions with the lowest death rate associated with AMR, Australasia, north Africa and the Middle East, and east Asia, have significantly higher fractions of infectious deaths that are associated with AMR than sub-Saharan Africa (31.0% (29.2 – 32.8), 45.3% (42.6 – 47.7), and 47.1% (44.6 – 49.2)), but relatively low infectious fractions (12.7% (8.9 – 17.6), 18.1% (13.2 – 24.2), and 12.2% (8.2 – 17.5)). The burden of AMR can also be analysed through the lens of death counts, in which case the large populations of east Asia and south Asia combined with relatively high infection fraction in south Asia (31.8% [26.0 – 39.2]) and relatively high fraction of infectious deaths that are associated with AMR (36.4% [30.8 – 41.4] in south Asia, 47.1% [44.6 – 49.2] in east Asia), result in these regions having the most deaths associated with and attributable to AMR in 2019.

Analysing infection and AMR as distinct the drivers of AMR burden has critical consequences for any policy intervention. Investments in infection control and prevention (IPC), water, sanitation, and hygiene, and vaccination can be made in areas with high infection fraction, while efforts in antibiotic stewardship can be enhanced in areas with high prevalence of resistance. Notably, our estimates show that rates of infectious disease are ultimately a

stronger driver of the burden of AMR than the prevalence of resistance itself, and should therefore be addressed first and foremost in any effort to reduce the burden of AMR.

Section 11. GATHER compliance

This study complies with GATHER recommendations.[45] We have documented the steps in our analytical procedures and detailed the data sources used. See table S12 for the GATHER checklist. The GATHER recommendations can be found on the GATHER website.

Section 12: References

1 Vos T, Lim SS, Abbafati C, *et al.* Global burden of 369 diseases and injuries in 204 countries and territories, 1990–2019: a systematic analysis for the Global Burden of Disease Study 2019. *The Lancet* 2020; **396**: 1204–22.

2 National Healthcare Safety Network (NHSN) patient safety component manual. CDC, 2021 https://www.cdc.gov/nhsn/pdfs/pscmanual/pcsmanual_current.pdf.

3 Browne A, Chipeta M, Haines-Woodhouse G, et al. Global antibiotic consumption in humans, 2000 to 2018: a spatial modelling study. *Lancet Planet Health* 2021.

4 Lozano R, Lopez AD, Atkinson C, Naghavi M, Flaxman AD, Murray CJ. Performance of physician-certified verbal autopsies: multisite validation study using clinical diagnostic gold standards. *Popul Health Metr* 2011; **9**: 32.

5 Naghavi M, Makela S, Foreman K, O'Brien J, Pourmalek F, Lozano R. Algorithms for enhancing public health utility of national causes-of-death data. *Popul Health Metr* 2010; **8**: 9.

6 Johnson SC, Cunningham M, Dippenaar IN, *et al.* Public health utility of cause of death data: applying empirical algorithms to improve data quality. *BMC Med Inform Decis Mak* 2021; **21**: 175.

7 Iburg KM, Mikkelsen L, Adair T, Lopez AD. Are cause of death data fit for purpose? evidence from 20 countries at different levels of socio-economic development. *PLOS ONE* 2020; **15**: e0237539.

8 Birnbaum JK, Murray CJ, Lozano R. Exposing misclassified HIV/AIDS deaths in South Africa. *Bull World Health Organ* 2011; **89**: 278–85.

9 Murray CJL, Rajaratnam JK, Marcus J, Laakso T, Lopez AD. What can we conclude from death registration? Improved methods for evaluating completeness. *PLOS Med* 2010; **7**: e1000262.

10 Foreman KJ, Lozano R, Lopez AD, Murray CJ. Modeling causes of death: an integrated approach using CODEm. *Popul Health Metr* 2012; **10**: 1.

11 Zheng P, Barber R, Sorensen RJD, Murray CJL, Aravkin AY. Trimmed constrained mixed effects models: formulations and algorithms. *J Comput Graph Stat* 2021; **0**: 1–13.

12 Wang H, Abbas KM, Abbasifard M, *et al.* Global age-sex-specific fertility, mortality, healthy life expectancy (HALE), and population estimates in 204 countries and territories, 1950–2019: a comprehensive demographic analysis for the Global Burden of Disease Study 2019. *The Lancet* 2020; **396**: 1160–203.

13 Rudd KE, Johnson SC, Agesa KM, *et al.* Global, regional, and national sepsis incidence and mortality, 1990–2017: analysis for the Global Burden of Disease Study. *The Lancet* 2020; **395**: 200–11.

14 Fullman N, Yearwood J, Abay SM, *et al.* Measuring performance on the Healthcare Access and Quality Index for 195 countries and territories and selected subnational locations: a systematic analysis from the Global Burden of Disease Study 2016. *The Lancet* 2018; **391**: 2236–71.

15 Weiner LM, Webb AK, Limbago B, *et al.* Antimicrobial-resistant pathogens associated with healthcare-associated infections: summary of data reported to the National Healthcare Safety Network at the Centers for Disease Control and Prevention, 2011-2014. *Infect Control Hosp Epidemiol* 2016; **37**: 1288–301.

16 Jones RN. Microbial etiologies of hospital-acquired bacterial pneumonia and ventilator-associated bacterial pneumonia. *Clin Infect Dis Off Publ Infect Dis Soc Am* 2010; **51 Suppl 1**: S81-87.

17 Jain S, Self WH, Wunderink RG, *et al.* Community-acquired pneumonia requiring hospitalization among U.S. adults. *N Engl J Med* 2015; **373**: 415–27.

18 Johansson N, Kalin M, Tiveljung-Lindell A, Giske CG, Hedlund J. Etiology of community-acquired pneumonia: increased microbiological yield with new diagnostic methods. *Clin Infect Dis Off Publ Infect Dis Soc Am* 2010; **50**: 202–9.

19 Czaja CA, Scholes D, Hooton TM, Stamm WE. Population-based epidemiologic analysis of acute pyelonephritis. *Clin Infect Dis Off Publ Infect Dis Soc Am* 2007; **45**: 273–80.

20 Nicolle LE. A practical guide to antimicrobial management of complicated urinary tract infection. *Drugs Aging* 2001; **18**: 243–54.

21 Goto M, Ohl ME, Schweizer ML, Perencevich EN. Accuracy of administrative code data for the surveillance of healthcare-associated infections: a systematic review and meta-analysis. *Clin Infect Dis Off Publ Infect Dis Soc Am* 2014; **58**: 688–96.

22 Wright SB, Huskins WC, Dokholyan RS, Goldmann DA, Platt R. Administrative databases provide inaccurate data for surveillance of long-term central venous catheter-associated infections. *Infect Control Hosp Epidemiol* 2003; **24**: 946–9.

23 Murray CJL, Aravkin AY, Zheng P, *et al.* Global burden of 87 risk factors in 204 countries and territories, 1990–2019: a systematic analysis for the Global Burden of Disease Study 2019. *The Lancet* 2020; **396**: 1223–49.

24 Mandrekar JN. Receiver operating characteristic curve in diagnostic test assessment. *J Thorac Oncol* 2010; **5**: 1315–6.

25 Ewig S, Schlochtermeier M, Göke N, Niederman MS. Applying sputum as a diagnostic tool in pneumonia: limited yield, minimal impact on treatment decisions. *Chest* 2002; **121**: 1486–92.

26 Ogawa H, Kitsios GD, Iwata M, Terasawa T. Sputum gram stain for bacterial pathogen diagnosis in community-acquired pneumonia: a systematic review and Bayesian meta-analysis of diagnostic accuracy and yield. *Clin Infect Dis* 2020; **71**: 499–513.

27 Feikin DR, Scott JAG, Gessner BD. Use of vaccines as probes to define disease burden. *Lancet Lond Engl* 2014; **383**: 1762–70.

28 Troeger C, Blacker B, Khalil IA, *et al.* Estimates of the global, regional, and national morbidity, mortality, and aetiologies of lower respiratory infections in 195 countries, 1990–2016: a systematic analysis for the Global Burden of Disease Study 2016. *Lancet Infect Dis* 2018; **18**: 1191–210.

29 Troeger C, Blacker B, Khalil IA, *et al.* Estimates of the global, regional, and national morbidity, mortality, and aetiologies of lower respiratory infections in 195 countries, 1990–2016: a systematic analysis for the Global Burden of Disease Study 2016. *Lancet Infect Dis* 2018; **18**: 1191–210.

30 Johnson HL, Deloria-Knoll M, Levine OS, *et al.* Systematic evaluation of serotypes causing invasive pneumococcal disease among ahildren under five: The Pneumococcal Global Serotype Project. *PLOS Med* 2010; **7**: e1000348.

31 Grijalva CG, Nuorti JP, Arbogast PG, Martin SW, Edwards KM, Griffin MR. Decline in pneumonia admissions after routine childhood immunisation with pneumococcal conjugate vaccine in the USA: a time-series analysis. *The Lancet* 2007; **369**: 1179–86.

32 Nocedal J, Wright S. Numerical optimization, 2nd edn. New York: Springer-Verlag, 2006 DOI:10.1007/978-0-387-40065-5.

33 Galles NC, Liu PY, Updike RL, *et al.* Measuring routine childhood vaccination coverage in 204 countries and territories, 1980–2019: a systematic analysis for the Global Burden of Disease Study 2020, Release 1. *The Lancet* 2021; **398**: 503–21.

34 Preziosi M, Department of Immunization, Vaccines, and Biologicals (World Health Organization). Update on meningococcal A vaccine development and introduction. Presented at: WHO Product Development for Vaccines Advisory Committee Meeting (PD-VAC). Geneva, Switzerland: WHO, 2015.

35 Troeger C, Forouzanfar M, Rao PC, *et al.* Estimates of global, regional, and national morbidity, mortality, and aetiologies of diarrhoeal diseases: a systematic analysis for the Global Burden of Disease Study 2015. *Lancet Infect Dis* 2017; **17**: 909–48.

36 Moher D, Liberati A, Tetzlaff J, Altman DG, The PRISMA Group. Preferred Reporting Items for Systematic Reviews and Meta-Analyses: the PRISMA statement. *PLoS Med* 2009; **6**: e1000097.

37 Browne AJ, Kashef Hamadani BH, Kumaran EAP, *et al.* Drug-resistant enteric fever worldwide, 1990 to 2018: a systematic review and meta-analysis. *BMC Med* 2020; **18**: 1.

38 Jamison DT, Breman JG, Measham AR, *et al.*, editors. Disease control priorities in developing countries second edition. Washington DC and New York: Oxford University Press and The World Bank, 2006.

39 Maina J, Ouma PO, Macharia PM, *et al.* A spatial database of health facilities managed by the public health sector in sub Saharan Africa. *Sci Data* 2019; **6**: 134.

40 Climatic Research Unit, University of East Anglia. CRU TS v. 3.23. Clim. Res. Unit. 2015; published online Sept. https://crudata.uea.ac.uk/cru/data/hrg/cru_ts_3.23/ (accessed Sept 27, 2021).

41 Gilbert M, Nicolas G, Cinardi G, *et al.* Global pigs distribution in 2010. 2018. DOI:10.7910/DVN/33N0JG.

42 Gilbert M, Nicolas G, Cinardi G, *et al.* Global distribution data for cattle, buffaloes, horses, sheep, goats, pigs, chickens and ducks in 2010. *Sci Data* 2018; **5**: 180227.

43 Cassini A, Högberg LD, Plachouras D, *et al.* Attributable deaths and disability-adjusted life-years caused by infections with antibiotic-resistant bacteria in the EU and the European Economic Area in 2015: a population-level modelling analysis. *Lancet Infect Dis* 2019; **19**: 56–66.

44 Rajendran NB, Mutters NT, Marasca G, *et al.* Mandatory surveillance and outbreaks reporting of the WHO priority pathogens for research & discovery of new antibiotics in European countries. *Clin Microbiol Infect* 2020; **26**: 943.e1-943.e6.

45 Stevens GA, Alkema L, Black RE, *et al.* Guidelines for Accurate and Transparent Health Estimates Reporting: the GATHER statement. *The Lancet* 2016; **388**: e19–23.

46 Bennett A, Bisanzio D, Yukich JO, *et al.* Population coverage of artemisinin-based combination treatment in children younger than 5 years with fever and Plasmodium falciparum infection in Africa, 2003-2015: a modelling study using data from national surveys. *Lancet Glob Health* 2017; **5**: e418–27.

47 Stanaway JD, Afshin A, Gakidou E, *et al.* Global, regional, and national comparative risk assessment of 84 behavioural, environmental and occupational, and metabolic risks or clusters of risks for 195 countries and territories, 1990–2017: a systematic analysis for the Global Burden of Disease Study 2017. *The Lancet* 2018; **392**: 1923–94.

48 Kaufmann D, Kraay A. Worldwide Governance Indicators. World Bank. 2019. https://info.worldbank.org/governance/wgi/ (accessed Aug 25, 2021).

49 GBD 2020 Health Financing Collaborator Network. Tracking development assistance for health and for COVID-19: a review of development assistance, government, out-of-pocket, and other private spending on health for 204 countries and territories, 1990–2050. *Lancet* 2021.

50 Fullman N, Yearwood J, Abay SM, *et al.* Measuring performance on the Healthcare Access and Quality Index for 195 countries and territories and selected subnational locations: a systematic analysis from the Global Burden of Disease Study 2016. *The Lancet* 2018; **391**: 2236–71.

51 Friedman J, York H, Graetz N, *et al.* Measuring and forecasting progress towards the education-related SDG targets. *Nature* 2020; **580**: 636–9.

52 Shaddick G, Thomas ML, Amini H, *et al.* Data integration for the assessment of population exposure to ambient air pollution for Global Burden of Disease assessment. *Environ Sci Technol* 2018; **52**: 9069–78.

53 GBD 2019 Human Resources for Health Collaborators. Measuring the availability of human resources for health and its relationship to universal health coverage: estimates for 204 countries and territories from 1990 to 2019. *Lancet* in review.

54 Lozano R, Fullman N, Abate D, *et al.* Measuring progress from 1990 to 2017 and projecting attainment to 2030 of the health-related Sustainable Development Goals for 195 countries and territories: a systematic analysis for the Global Burden of Disease Study 2017. *The Lancet* 2018; **392**: 2091–138.

Section 13: Author contributions

Managing the estimation or publications process

Christopher J L Murray, Kevin Shunji Ikuta, Catherine Bisignano, Puja Rao, Eve Wool, Barney McManigal, Alan D Lopez, Simon I Hay, Andy Stergachis, Catrin E Moore, Christiane Dolecek, and Mohsen Naghavi.

Writing the first draft of the manuscript

--Christopher J L Murray, Kevin Shunji Ikuta, Fablina Sharara, Lucien Swetschinski, Gisela Robles Aguilar, Authia Gray, Chieh Han, Catherine Bisignano, Eve Wool, Annie J Browne, Michael Give Chipeta, Frederick Fell, Sean Hackett, Georgina Haines-Woodhouse, Bahar H Kashef Hamadani, Emmanuelle A P Kumaran, Benn Sartorius, Catrin E Moore, Christiane Dolecek, and Mohsen Naghavi.

Primary responsibility for applying analytical methods to produce estimates

Kevin Shunji Ikuta, Fablina Sharara, Lucien Swetschinski, Gisela Robles Aguilar, Authia Gray, Chieh Han, Sarah C Johnson, Annie J Browne, Michael Give Chipeta, Frederick Fell, Sean Hackett, Georgina Haines-Woodhouse, Bahar H Kashef Hamadani, Emmanuelle A P Kumaran, Benn Sartorius, Catrin E Moore, and Christiane Dolecek.

Primary responsibility for seeking, cataloguing, extracting, or cleaning data; designing or coding figures and tables

Fablina Sharara, Lucien Swetschinski, Gisela Robles Aguilar, Authia Gray, Chieh Han, Sarah C Johnson, Annie J Browne, Michael Give Chipeta, Frederick Fell, Sean Hackett, Georgina Haines-Woodhouse, Bahar H Kashef Hamadani, Emmanuelle A P Kumaran, Barney McManigal, Catrin E Moore, Christiane Dolecek and Mohsen Naghavi

Providing data or critical feedback on data sources

Christopher J L Murray, Kevin Shunji Ikuta, Gisela Robles Aguilar, Michael Give Chipeta, Georgina Haines-Woodhouse, Bahar H Kashef Hamadani, Emmanuelle A P Kumaran, Barney McManigal, Ramesh Agarwal, Samuel Akech, John Amuasi, Jason Andrews, Elizabeth Ashley, Freddie Bailey, Stephen Baker, Adrie Bekker, Adhisivam Bethou, Julia Bielicki, Suppawat Boonkasidecha, James Bukosia, Cristina Carvalheiro, Carlos Castañeda-Orjuela, Vilada Chansamouth, Suman Chaurasia, Sara Chiurchiù, Fazle Chowdhury, Aislinn J Cook, Tim R Cressey, Elia Criollo-Mora, Saffiatou Darboe, Nicholas P J Day, Maia De Luca, Klara Dokova, Angela Dramowski, Susanna J Dunachie, Tim Eckmanns, Daniel Eibach, Amir Emami, Nicholas Feasey, Natasha Fisher-Pearson, Karen Forrest, Denise Garrett, Petra Gastmeier, Ababi Zergaw Giref, Rachel Claire Greer, Vikas Gupta, Sebastian Haller, Andrea Haselbeck, Simon I Hay, Marianne Holm, Susan Hopkins, Jan Jacobs, Daniel Jarovsky, Fatemeh Javanmardi, Meera Khorana, Fiorella Krapp, Ralf Krumkamp, Ajay Kumar, Hmwe H Kyu, Direk Limmathurotsakul, Michael James Loftus, Neema Mturi, Patrick Musicha, Marisa Marcia Mussi-Pinhata, Tomoka Nakamura, Ruchi Nanavati, Sushma Nangia, Paul Newton, Chanpheaktra Ngoun, Davis Nwakanma, Christina W Obiero, Antonio Olivas-Martinez, Ednah Ooko, Edgar Ortiz-Brizuela, Anton Yariv Peleg, Carlo Perrone, Nishad Plakkal, Alfredo Ponce-de-Leon, Mathieu Raad , Tanusha Ramdin, Amy Riddell, Tamalee Roberts, Julie Victoria Robotham, Anna Roca, Neal Russell, Jesse Schnall, John Anthony Gerard Scott, Madhusudhan Shivamallappa, Jose Sifuentes-Osornio, Andrew James Stewardson, Temenuga Stoeva, Nidanuch Tasak, Areerat Thaiprakong, Guy Thwaites, Claudia Turner, Paul Turner, H Rogier van Doorn, Sithembiso Velaphi, Huong Vu, Timothy Walsh, Seymour Waner, Tri Wangrangsimakul, Teresa Wozniak, Alan D Lopez, Andy Stergachis, Catrin E Moore, Christiane Dolecek, and Mohsen Naghavi.

Developing methods or computational machinery

Christopher J L Murray, Kevin Shunji Ikuta, Fablina Sharara, Lucien Swetschinski, Gisela Robles Aguilar, Authia Gray, Sarah C Johnson, Annie J Browne, Michael Give Chipeta, Georgina Haines-Woodhouse, Emmanuelle A P Kumaran, Aleskandr Aravkin, Hmwe H Kyu, Kristina E Rudd, Nicolas Steenkeste, Sithembiso Velaphi, Peng Zheng, Benn Sartorius, Andy Stergachis, and Mohsen Naghavi.

Providing critical feedback on methods or results

Christopher J L Murray, Kevin Shunji Ikuta, Fablina Sharara, Lucien Swetschinski, Chieh Han, Sarah C Johnson, Annie J Browne, Michael Give Chipeta, Georgina Haines-Woodhouse, Bahar H Kashef Hamadani, Emmanuelle A P Kumaran, Ramesh Agarwal, Samuel Akech, Aleskandr Aravkin, Elizabeth Ashley, Freddie Bailey, Buddha

Basnyat, Rose Bender, Julia Bielicki, Sara Chiurchiù, Ben Cooper, Klara Dokova, Angela Dramowski, Susanna J Dunachie, Tim Eckmanns, Daniel Eibach, Petra Gastmeier, Sebastian Haller, Simon I Hay, Marianne Holm, Susan Hopkins, Kenneth C Iregbu, Tomislav Kostyanev, Ralf Krumkamp, Hmwe H Kyu, Cherry Lim, Michael James Loftus, Neema Mturi, Tatiana Munera-Huertas, Ruchi Nanavati, Chanpheaktra Ngoun, Antonio Olivas-Martinez, Ednah Ooko, Edgar Ortiz-Brizuela, Mathieu Raad, Julie Victoria Robotham, Kristina E Rudd, Neal Russell, Jesse Schnall, Madhusudhan Shivamallappa, Temenuga Stoeva, Paul Turner, Huong Vu, Tri Wangrangsimakul, Peng Zheng, Benn Sartorius, Alan D Lopez, Andy Stergachis, Catrin E Moore, Christiane Dolecek and Mohsen Naghavi.

Drafting the work or revising it critically for important intellectual content

Christopher J L Murray, Kevin Shunji Ikuta, Fablina Sharara, Lucien Swetschinski, Gisela Robles Aguilar, Authia Gray, Chieh Han, Catherine Bisignano, Puja Rao, Eve Wool, Annie J Browne, Michael Give Chipeta, Georgina Haines-Woodhouse, Bahar H Kashef Hamadani, Emmanuelle A P Kumaran, Jason Andrews, Freddie Bailey, Julia Bielicki, Cristina Carvalheiro, Carlos Castañeda-Orjuela, Fazle Chowdhury, Ben Cooper, Tim R Cressey, Saffiatou Darboe, Angela Dramowski, Susanna J Dunachie, Sebastian Haller, Susan Hopkins, Kenneth C Iregbu, Niranjan Kissoon, Elsa Kobeissi, Hmwe H Kyu, Michael James Loftus, Marisa Marcia Mussi-Pinhata, Paul Newton, Piero Olliaro, Ednah Ooko, Anton Yariv Peleg, Kristina E Rudd, Neal Russell, Andrew James Stewardson, Timothy Walsh, Tri Wangrangsimakul, Alan D Lopez, Catrin E Moore, Christiane Dolecek and Mohsen Naghavi.

Extracting, cleaning, or cataloging data; designing or coding figures and tables

Kevin Shunji Ikuta, Fablina Sharara, Lucien Swetschinski, Gisela Robles Aguilar, Authia Gray, Chieh Han, Sarah C Johnson, Annie J Browne, Michael Give Chipeta, Frederick Fell, Sean Hackett, Georgina Haines-Woodhouse, Bahar H Kashef Hamadani, Emmanuelle A P Kumaran, Barney McManigal, Samuel Albertson, Freddie Bailey, Elia Criollo-Mora, Matthew Cunningham, Susanna J Dunachie, Natasha Fisher-Pearson, Rachel Claire Greer, Kenneth C Iregbu, Elsa Kobeissi, Miles Lunn, Jianing Ma, Patrick Musicha, Davis Nwakanma, Ednah Ooko, Edgar Ortiz-Brizuela, Carlo Perrone, Alfredo Ponce-de-Leon, Prénom Raad, Amy Riddell, Tamalee Roberts, Jesse Schnall, Nidanuch Tasak, Areerat Thaiprakong, Avina Vongpradith, Tri Wangrangsimakul, Catrin E Moore, Christiane Dolecek and Mohsen Naghavi.

Managing the overall research enterprise

Christopher J L Murray, Kevin Shunji Ikuta, Puja Rao, Eve Wool, Ramesh Agarwal, Adrie Bekker, Nicholas P J Day, Susanna J Dunachie, Simon I Hay, Ruchi Nanavati, Amanda Novotney, Nicolas Steenkeste, Benn Sartorius, Alan D Lopez, Catrin E Moore, and Mohsen Naghavi.

Section 14: Appendix tables and figures

Table S1. GBD cause hierarchy with levels

Cause	level
All causes	0
Communicable, maternal, neonatal, and nutritional diseases	1
HIV/AIDS and sexually transmitted infections	2
HIV/AIDS	3
HIV/AIDS–drug-susceptible tuberculosis	4
HIV/AIDS–multidrug-resistant TB without extensive drug resistance	4
HIV/AIDS–extensively drug-resistant tuberculosis	4
HIV/AIDS resulting in other diseases	4
Sexually transmitted infections excluding HIV	3
Syphilis	4
Chlamydial infection	4
Gonococcal infection	4
Trichomoniasis	4
Genital herpes	4
Other sexually transmitted infections	4
Respiratory infections and tuberculosis	2
Tuberculosis	3
Latent tuberculosis infection	4
Drug-susceptible tuberculosis	4
Multidrug-resistant TB without extensive drug resistance	4
Extensively drug-resistant tuberculosis	4
Lower respiratory infections	3
Upper respiratory infections	3
Otitis media	3
Enteric infections	2
Diarrhoeal diseases	3
Typhoid and paratyphoid	3
Typhoid fever	4
Paratyphoid fever	4
Invasive non-typhoidal Salmonella (iNTS)	3
Other intestinal infectious diseases	3
Neglected tropical diseases and malaria	2
Malaria	3
Chagas disease	3
Leishmaniasis	3
Visceral leishmaniasis	4
Cutaneous and mucocutaneous leishmaniasis	4
African trypanosomiasis	3
Schistosomiasis	3
Cysticercosis	3
Cystic echinococcosis	3
Lymphatic filariasis	3
Onchocerciasis	3

Trachoma	3
Dengue	3
Yellow fever	3
Rabies	3
Intestinal nematode infections	3
Ascariasis	4
Trichuriasis	4
Hookworm disease	4
Food-borne trematodiases	3
Leprosy	3
Ebola virus disease	3
Zika virus disease	3
Guinea worm disease	3
Other neglected tropical diseases	3
Other infectious diseases	2
Meningitis	3
Encephalitis	3
Diphtheria	3
Whooping cough	3
Tetanus	3
Measles	3
Varicella and herpes zoster	3
Acute hepatitis	3
Acute hepatitis A	4
Acute hepatitis B	4
Acute hepatitis C	4
Acute hepatitis E	4
Other unspecified infectious diseases	3
Maternal and neonatal disorders	2
Maternal disorders	3
Maternal haemorrhage	4
Maternal sepsis and other maternal infections	4
Maternal hypertensive disorders	4
Maternal obstructed labor and uterine rupture	4
Maternal abortion and miscarriage	4
Ectopic pregnancy	4
Indirect maternal deaths	4
Late maternal deaths	4
Maternal deaths aggravated by HIV/AIDS	4
Other maternal disorders	4
Neonatal disorders	3
Neonatal preterm birth	4
Neonatal encephalopathy due to birth asphyxia and trauma	4
Neonatal sepsis and other neonatal infections	4
Haemolytic disease and other neonatal jaundice	4

Other neonatal disorders	4
Nutritional deficiencies	2
Protein-energy malnutrition	3
Iodine deficiency	3
Vitamin A deficiency	3
Dietary iron deficiency	3
Other nutritional deficiencies	3
Non-communicable diseases	1
Neoplasms	2
Lip and oral cavity cancer	3
Nasopharynx cancer	3
Other pharynx cancer	3
Oesophageal cancer	3
Stomach cancer	3
Colon and rectum cancer	3
Liver cancer	3
Liver cancer due to hepatitis B	4
Liver cancer due to hepatitis C	4
Liver cancer due to alcohol use	4
Liver cancer due to NASH	4
Liver cancer due to other causes	4
Gallbladder and biliary tract cancer	3
Pancreatic cancer	3
Larynx cancer	3
Tracheal, bronchus, and lung cancer	3
Malignant skin melanoma	3
Non-melanoma skin cancer	3
Non-melanoma skin cancer (squamous-cell carcinoma)	4
Non-melanoma skin cancer (basal-cell carcinoma)	4
Breast cancer	3
Cervical cancer	3
Uterine cancer	3
Ovarian cancer	3
Prostate cancer	3
Testicular cancer	3
Kidney cancer	3
Bladder cancer	3
Brain and central nervous system cancer	3
Thyroid cancer	3
Mesothelioma	3
Hodgkin lymphoma	3
Non-Hodgkin lymphoma	3
Multiple myeloma	3
Leukaemia	3
Acute lymphoid leukaemia	4

Chronic lymphoid leukaemia	4
Acute myeloid leukaemia	4
Chronic myeloid leukaemia	4
Other leukaemia	4
Other malignant neoplasms	3
Other neoplasms	3
Myelodyspastic, myeloproliferative, and other haematopoietic neoplasms	4
Benign and in situ intestinal neoplasms	4
Benign and in situ cervical and uterine neoplasms	4
Other benign and in situ neoplasms	4
Cardiovascular diseases	2
Rheumatic heart disease	3
Ischaemic heart disease	3
Stroke	3
Ischaemic stroke	4
Intracerebral haemorrhage	4
Subarachnoid haemorrhage	4
Hypertensive heart disease	3
Non-rheumatic valvular heart disease	3
Non-rheumatic calcific aortic valve disease	4
Non-rheumatic degenerative mitral valve disease	4
Other non-rheumatic valve diseases	4
Cardiomyopathy and myocarditis	3
Myocarditis	4
Alcoholic cardiomyopathy	4
Other cardiomyopathy	4
Atrial fibrillation and flutter	3
Aortic aneurysm	3
Peripheral artery disease	3
Endocarditis	3
Other cardiovascular and circulatory diseases	3
Chronic respiratory diseases	2
Chronic obstructive pulmonary disease	3
Pneumoconiosis	3
Silicosis	4
Asbestosis	4
Coal workers pneumoconiosis	4
Other pneumoconiosis	4
Asthma	3
Interstitial lung disease and pulmonary sarcoidosis	3
Other chronic respiratory diseases	3
Digestive diseases	2
Cirrhosis and other chronic liver diseases	3
Cirrhosis and other chronic liver diseases due to hepatitis B	4
Cirrhosis and other chronic liver diseases due to hepatitis C	4

Cirrhosis and other chronic liver diseases due to alcohol use	4
Cirrhosis and other chronic liver diseases due to NAFLD	4
Cirrhosis and other chronic liver diseases due to other causes	4
Upper digestive system diseases	3
Peptic ulcer disease	4
Gastritis and duodenitis	4
Gastro-oesophageal reflux disease	4
Appendicitis	3
Paralytic ileus and intestinal obstruction	3
Inguinal, femoral, and abdominal hernia	3
Inflammatory bowel disease	3
Vascular intestinal disorders	3
Gallbladder and biliary diseases	3
Pancreatitis	3
Other digestive diseases	3
Neurological disorders	2
Alzheimer's disease and other dementias	3
Parkinson's disease	3
Idiopathic epilepsy	3
Multiple sclerosis	3
Motor neuron disease	3
Headache disorders	3
Migraine	4
Tension-type headache	4
Other neurological disorders	3
Mental disorders	2
Schizophrenia	3
Depressive disorders	3
Major depressive disorder	4
Dysthymia	4
Bipolar disorder	3
Anxiety disorders	3
Eating disorders	3
Anorexia nervosa	4
Bulimia nervosa	4
Autism spectrum disorders	3
Attention-deficit/hyperactivity disorder	3
Conduct disorder	3
Idiopathic developmental intellectual disability	3
Other mental disorders	3
Substance use disorders	2
Alcohol use disorders	3
Drug use disorders	3
Opioid use disorders	4
Cocaine use disorders	4

Amphetamine use disorders	4
Cannabis use disorders	4
Other drug use disorders	4
Diabetes and kidney diseases	2
Diabetes mellitus	3
Diabetes mellitus type 1	4
Diabetes mellitus type 2	4
Chronic kidney disease	3
Chronic kidney disease due to diabetes mellitus type 1	4
Chronic kidney disease due to diabetes mellitus type 2	4
Chronic kidney disease due to hypertension	4
Chronic kidney disease due to glomerulonephritis	4
Chronic kidney disease due to other and unspecified causes	4
Acute glomerulonephritis	3
Skin and subcutaneous diseases	2
Dermatitis	3
Atopic dermatitis	4
Contact dermatitis	4
Seborrhoeic dermatitis	4
Psoriasis	3
Bacterial skin diseases	3
Cellulitis	4
Pyoderma	4
Scabies	3
Fungal skin diseases	3
Viral skin diseases	3
Acne vulgaris	3
Alopecia areata	3
Pruritus	3
Urticaria	3
Decubitus ulcer	3
Other skin and subcutaneous diseases	3
Sense organ diseases	2
Blindness and vision loss	3
Glaucoma	4
Cataract	4
Age-related macular degeneration	4
Refraction disorders	4
Near vision loss	4
Other vision loss	4
Age-related and other hearing loss	3
Other sense organ diseases	3
Musculoskeletal disorders	2
Rheumatoid arthritis	3
Osteoarthritis	3

Osteoarthritis hip	4
Osteoarthritis knee	4
Osteoarthritis hand	4
Osteoarthritis other	4
Low back pain	3
Neck pain	3
Gout	3
Other musculoskeletal disorders	3
Other non-communicable diseases	2
Congenital birth defects	3
Neural tube defects	4
Congenital heart anomalies	4
Orofacial clefts	4
Down syndrome	4
Turner syndrome	4
Klinefelter syndrome	4
Other chromosomal abnormalities	4
Congenital musculoskeletal and limb anomalies	4
Urogenital congenital anomalies	4
Digestive congenital anomalies	4
Other congenital birth defects	4
Urinary diseases and male infertility	3
Urinary tract infections	4
Urolithiasis	4
Benign prostatic hyperplasia	4
Male infertility	4
Other urinary diseases	4
Gynaecological diseases	3
Uterine fibroids	4
Polycystic ovarian syndrome	4
Female infertility	4
Endometriosis	4
Genital prolapse	4
Premenstrual syndrome	4
Other gynaecological diseases	4
Haemoglobinopathies and haemolytic anemias	3
Thalassaemias	4
Thalassaemias trait	4
Sickle cell disorders	4
Sickle cell trait	4
G6PD deficiency	4
G6PD trait	4
Other haemoglobinopathies and haemolytic anaemias	4
Endocrine, metabolic, blood, and immune disorders	3
Oral disorders	3

Caries of deciduous teeth	4
Caries of permanent teeth	4
Periodontal diseases	4
Edentulism and severe tooth loss	4
Other oral disorders	4
Sudden infant death syndrome	3
Injuries	1
Transport injuries	2
Road injuries	3
Pedestrian road injuries	4
Cyclist road injuries	4
Motorcyclist road injuries	4
Motor vehicle road injuries	4
Other road injuries	4
Other transport injuries	3
Unintentional injuries	2
Falls	3
Drowning	3
Fire, heat, and hot substances	3
Poisonings	3
Poisoning by carbon monoxide	4
Poisoning by other means	4
Exposure to mechanical forces	3
Unintentional firearm injuries	4
Other exposure to mechanical forces	4
Adverse effects of medical treatment	3
Animal contact	3
Venomous animal contact	4
Non-venomous animal contact	4
Foreign body	3
Pulmonary aspiration and foreign body in airway	4
Foreign body in eyes	4
Foreign body in other body part	4
Environmental heat and cold exposure	3
Exposure to forces of nature	3
Other unintentional injuries	3
Self-harm and interpersonal violence	2
Self-harm	3
Self-harm by firearm	4
Self-harm by other specified means	4
Interpersonal violence	3
Physical violence by firearm	4
Physical violence by sharp object	4
Sexual violence	4
Physical violence by other means	4

Conflict and terrorism	3
Executions and police conflict	3

Table S2: List of International Classification of Diseases (ICD) codes mapped to the Global Burden of Disease cause list for causes of death		
**Cause**	**ICD10**	**ICD9**
Communicable, maternal, neonatal, and nutritional diseases	A00-A00.9, A01.0-A14, A15-A28.9, A32-A39.9, A48.1-A48.2, A48.4-A48.5, A50-A58, A60-A60.9, A63-A63.8, A65-A65.0, A68-A70, A74, A74.8-A75.9, A77-A96.9, A98-A98.8, B00-B06.9, B10-B10.8, B15-B16.2, B17.0, B17.2, B19.1, B20-B27.9, B29.4, B33-B33.1, B33.3-B33.8, B47-B48.8, B50-B54.0, B55.0, B56-B57.5, B60-B60.8, B63, B65-B67.9, B69-B72.0, B74.3-B75, B77-B77.9, B83-B83.8, B90-B91, B94.1, B95-B95.5, B97.4-B97.6, C58-C58.0, D50.1-D50.8, D51-D52.0, D52.8-D53.9, D70.3, D89.3, E00-E02, E40-E46.9, E51-E61.9, E63-E64.0, E64.2-E64.9, F02.1, F02.4, F07.1, G00.0-G00.8, G03-G03.8, G04-G05.8, G14-G14.6, G21.3, H70-H70.9, I00, I02, I02.9, I98.0-I98.1, J00-J02.8, J03-J03.8, J04-J04.2, J05-J05.1, J06.0-J06.8, J09-J15.8, J16-J16.9, J20-J21.9, J36-J36.0, J91.0, K52.1, K67.0-K67.8, K75.3, K76.3, K77.0, K93.0-K93.1, M03.1, M12.1, M49.0-M49.1, M73.0-M73.1, M89.6, N74.1, N96, N98-N98.9, O00-O07.9, O09-O16.9, O20-O26.9, O28-O36.9, O40-O48.1, O60-O77.9, O80-O92.7, O96-O98.6, O98.8-P04.2, P04.5-P05.9, P07-P15.9, P19-P22.9, P23.0-P23.4, P24-P29.9, P35-P37.2, P37.5-P39.9, P50-P61.9, P70-P70.1, P70.3-P72.9, P74-P78.9, P80-P81.9, P83-P84, P90-P94.9, P96, P96.3-P96.4, P96.8, R19.7, U04-U04.9, U06-U06.9, U82-U89, Z16-Z16.3	001-001.9, 002.0-029, 032-034.9, 036-036.3, 036.5-037.9, 040, 040.1-041.0, 042-066.9, 070.0-070.2, 071-075.9, 078.3-078.7, 079-079.7, 080-083.9, 084.0-084.5, 084.7-084.9, 085.0, 086-088, 088.8-088.9, 090-101.6, 104-104.9, 120-124.9, 125.4-125.9, 127-127.1, 128-129.0, 136-136.2, 137-139.0, 181-181.9, 244.2, 260-263.9, 265-269.9, 281.0-281.9, 320.0-320.8, 321-323.9, 381-383.9, 390-390.9, 392, 392.9, 425.6, 460-464.4, 464.8-464.9, 465.0-465.8, 466-469, 470.0, 475-475.9, 476.9, 480-482.8, 483.0-483.9, 484.0-484.7, 487-489, 630-636.9, 638-638.9, 640-679.1, 716.0, 730.4-730.6, 760-760.6, 760.8-768, 768.2-770, 770.1-775.0, 775.4-779.3, 779.6-779.8, V09-V09.9
HIV/AIDS and sexually transmitted infections	A50-A58, A60-A60.9, A63-A63.8, B20-B24.9, B63, F02.4, I98.0, K67.0-K67.2, M03.1, M73.0-M73.1	042-044.9, 054.1, 090-099.9
HIV/AIDS	B20-B24.9, F02.4	042-044.9
HIV/AIDS–drug-susceptible tuberculosis	B20.0	
HIV/AIDS–multidrug-resistant tuberculosis without extensive drug resistance		
HIV/AIDS–extensively drug-resistant tuberculosis		
HIV/AIDS resulting in other diseases	B20, B20.1-B24.9, F02.4	042-044.9
Sexually transmitted infections excluding HIV	A50-A58, A60-A60.9, A63-A63.8, B63, I98.0, K67.0-K67.2, M03.1, M73.0-M73.1	054.1, 090-099.9
Syphilis	A50-A53.9, I98.0, K67.2, M03.1, M73.1	090-097.9
Chlamydial infection	A55-A56.8, K67.0	
Gonococcal infection	A54-A54.9, K67.1, M73.0	098-098.9
Other sexually transmitted infections	A57-A58, A63-A63.8, B63	099-099.9
Respiratory infections and tuberculosis	A10-A14, A15-A19.9, A48.1, A70, B90-B90.9, B97.4-B97.6, H70-H70.9, J00-J02.8, J03-J03.8, J04-J04.2, J05-J05.1, J06.0-J06.8, J09-J15.8, J16-J16.9, J20-J21.9, J36-J36.0, J91.0, K67.3, K93.0, M49.0, N74.1, P23.0-P23.4, P37.0, U04-U04.9, U84.3	010-019.9, 034.0, 079.6, 137-137.9, 138.0-138.9, 381-383.9, 460-464.4, 464.8-464.9, 465.0-465.8, 466-469, 470.0, 475-475.9, 476.9, 480-482.8, 483.0-483.9, 484.1-484.2, 484.6-484.7, 487-489, 730.4-730.6
Tuberculosis	A10-A14, A15-A19.9, B90-B90.9, K67.3, K93.0, M49.0, N74.1, P37.0, U84.3	010-019.9, 137-137.9, 138.0-138.9, 730.4-730.6
Drug-susceptible tuberculosis	A10-A14, A15-A19.9, B90-B90.9, K67.3, K93.0, M49.0, N74.1, P37.0	010-019.9, 137-137.9, 138.0-138.9, 730.4-730.6
Multidrug-resistant tuberculosis without extensive drug resistance	U84.3	
Extensively drug-resistant tuberculosis		
Lower respiratory infections	A48.1, A70, B97.4-B97.6, J09-J15.8, J16-J16.9, J20-J21.9, J91.0, P23.0-P23.4, U04-U04.9	079.6, 466-469, 470.0, 480-482.8, 483.0-483.9, 484.1-484.2, 484.6-484.7, 487-489
Upper respiratory infections	J00-J02.8, J03-J03.8, J04-J04.2, J05-J05.1, J06.0-J06.8, J36-J36.0	034.0, 460-464.4, 464.8-464.9, 465.0-465.8, 475-475.9, 476.9
Otitis media	H70-H70.9	381-383.9
Enteric infections	A00-A00.9, A01.0-A09.9, A80-A80.9, K52.1, R19.7	001-001.9, 002.0-009.9, 045-045.9, 138
Diarrheal diseases	A00-A00.9, A02-A02.0, A02.8-A07, A07.2-A07.4, A08-A09.9, K52.1, R19.7	001-001.9, 003.8-006.9, 007.4-007.8, 008.2-009.9
Typhoid and paratyphoid	A01.0-A01.4	002.0-002.9
Typhoid fever	A01.0	002.0
Paratyphoid fever	A01.1-A01.4	002.1-002.9
Invasive non-typhoidal Salmonella (iNTS)	A02.1-A02.2	003-003.7
Other intestinal infectious diseases	A07.0-A07.1, A07.8-A07.9, A80-A80.9	007-007.3, 007.9-008.1, 045-045.9, 138
Neglected tropical diseases and malaria	A68-A68.9, A69.2-A69.9, A75-A75.9, A77-A79.9, A82-A82.9, A90-A96.9, A98-A98.8, B33.0-B33.1, B50-B54.0, B55.0, B56-B57.5, B60-B60.8, B65-B67.9, B69-B72.0, B74.3-B75, B77-B77.9, B83-B83.8, K93.1, P37.1, U06-U06.9	060-061.8, 065-066.9, 071-071.9, 080-083.9, 084.0-084.5, 084.7-084.9, 085.0, 086-088, 088.8-088.9, 120-124.9, 125.4-125.9, 127-127.1, 128-129.0, 425.6
Malaria	B50-B54.0	084.0-084.5, 084.7-084.9
Leprosy	A30-A30.9	030-030.9
Chagas disease	B57-B57.5, K93.1	086-086.2, 086.9, 425.6
Leishmaniasis	B55.0	085.0
Visceral leishmaniasis	B55.0	085.0
African trypanosomiasis	B56-B56.9	086.3-086.5
Schistosomiasis	B65-B65.9	120-120.9
Cysticercosis	B69-B69.9	123.1
Cystic echinococcosis	B67-B67.4, B67.8-B67.9	122-122.4, 122.8-122.9
Dengue	A90-A91.9	061-061.8
Yellow fever	A95-A95.9	060-060.9
Rabies	A82-A82.9	071-071.9
Intestinal nematode infections	B77-B77.9	127.0
Ascariasis	B77-B77.9	127.0
Ebola virus disease	A98.4	
Zika virus disease	U06-U06.9	
Other neglected tropical diseases	A68-A68.9, A69.2-A69.9, A75-A75.9, A77-A79.9, A92-A94.0, A96-A96.9, A98-A98.3, A98.5-A98.8, B33.0-B33.1, B60-B60.8, B67.5-B67.7, B70-B71.9, B74.3-B75, B83-B83.8, P37.1	065-066.9, 080-083.9, 087-088, 088.8-088.9, 122.5-122.7, 123-123.0, 123.2-124.9, 125.4-125.6, 125.9, 127, 127.1, 128-129.0

Table S2: List of International Classification of Diseases (ICD) codes mapped to the Global Burden of Disease cause list for causes of death

Cause	ICD10	ICD9
Other infectious diseases	A20-A28.9, A32-A39.9, A48.2, A48.4-A48.5, A65-A65.0, A69-A69.1, A74, A74.8-A74.9, A81-A81.9, A83-A89.9, B00-B06.9, B10-B10.8, B15-B16.2, B17.0, B17.2, B19.1, B25-B27.9, B29.4, B33, B33.3-B33.8, B47-B48.8, B91, B94.1, B95-B95.5, D70.3, D89.3, F02.1, F07.1, G00.0-G00.8, G03-G03.8, G04-G05.8, G14-G14.6, G21.3, I00, I02, I02.9, I98.1, K67.8, K75.3, K76.3, K77.0, M49.1, M89.6, P35-P35.9, P37, P37.2, P37.5-P37.9, U82-U84, U85-U89, Z16-Z16.3	020-029, 032-034, 034.1-034.9, 036-036.3, 036.5-037.9, 040, 040.1-041.0, 046-054.0, 054.2-059.9, 062-064.9, 070.0-070.2, 072-075.9, 078.3-078.7, 079-079.5, 079.7, 100-101.6, 104-104.9, 136-136.2, 139-139.0, 320.0-320.8, 321-323.9, 390-390.9, 392, 392.9, 484.0, 484.3-484.5, 771.0-771.3, V09-V09.9
Meningitis	A39-A39.9, A87-A87.9, G00.0-G00.8, G03-G03.8	036-036.3, 036.5-036.9, 047-049.9, 320.0-320.8, 321-322.9
Encephalitis	A83-A86.4, B94.1, F07.1, G04-G05.8, G21.3	062-064.9, 139.0, 323, 323.4-323.9
Diphtheria	A36-A36.9	032-032.9
Whooping cough	A37-A37.9	033-033.9, 484.3
Tetanus	A33-A35.0	037-037.9, 771.3
Measles	B05-B05.9	055-055.9, 484.0
Varicella and herpes zoster	B01-B02.9, P35.8	052-053.9
Acute hepatitis	B15-B16.2, B17.0, B17.2, B19.1, P35.3	070.0-070.2
Acute hepatitis A	B15-B15.9	070.0-070.1
Acute hepatitis B	B16-B16.2, B17.0, B19.1, P35.3	070.2
Acute hepatitis C		
Acute hepatitis E	B17.2	
Other unspecified infectious diseases	A20-A28.9, A32-A32.9, A38-A38.9, A48.2, A48.4-A48.5, A65-A65.0, A69-A69.1, A74, A74.8-A74.9, A81-A81.9, A88-A89.9, B00-B00.9, B03-B04, B06-B06.9, B10-B10.8, B25-B27.9, B29.4, B33, B33.3-B33.8, B47-B48.8, B91, B95-B95.5, D70.3, D89.3, F02.1, G14-G14.6, I00, I02, I02.9, I98.1, K67.8, K75.3, K76.3, K77.0, M49.1, M89.6, P35-P35.2, P35.9, P37, P37.2, P37.5-P37.9, U82-U84, U85-U89, Z16-Z16.3	020-029, 034, 034.1-034.9, 040, 040.1-041.0, 046-046.9, 050-051.9, 054-054.0, 054.2-054.9, 056-059.9, 072-075.9, 078.3-078.7, 079-079.5, 079.7, 100-101.6, 104-104.9, 136-136.2, 139, 323.0-323.3, 390-390.9, 392, 392.9, 484.4-484.5, 771.0-771.2, V09-V09.9
Maternal and neonatal disorders	C58-C58.0, N96, N98-N98.9, O00-O07.9, O09-O16.9, O20-O26.9, O28-O36.9, O40-O48.1, O60-O77.9, O80-O92.7, O96-O98.6, O98.8-P04.2, P04.5-P05.9, P07-P15.9, P19-P22.9, P24-P29.9, P36-P36.9, P38-P39.9, P50-P61.9, P70-P70.1, P70.3-P72.9, P74-P78.9, P80-P81.9, P83-P84, P90-P94.9, P96, P96.3-P96.4, P96.8	181-181.9, 630-636.9, 638-638.9, 640-679.1, 760-760.6, 760.8-768, 768.2-770, 770.1-771, 771.4-775.0, 775.4-779.3, 779.6-779.8
Maternal disorders	C58-C58.0, N96, N98-N98.9, O00-O07.9, O09-O16.9, O20-O26.9, O28-O36.9, O40-O48.1, O60-O77.9, O80-O92.7, O96-O98.6, O98.8-O99.9	181-181.9, 630-636.9, 638-638.9, 640-679.1
Maternal haemorrhage	O20-O20.9, O43.2, O44-O46.9, O62-O62.9, O67-O67.9, O70, O72-O72.3	640-641.9, 661-661.9, 665, 666-666.9
Maternal sepsis and other maternal infections	O23-O23.9, O85-O86.8, O91-O91.2	659.3, 670-670.9
Maternal hypertensive disorders	O10-O16.9	642-642.9
Maternal obstructed labor and uterine rupture	O32-O33.9, O64-O66.9, O71-O71.9	652-653.9, 660-660.9, 665.0-665.3
Maternal abortion and miscarriage	N96, O01-O07.9	630-632.9, 634-636.9, 638-638.9, 646.3
Ectopic pregnancy	O00-O00.9	633-633.9
Indirect maternal deaths	O24-O25.3, O98-O98.6, O98.8-O99.9	646-646.2, 646.4-649.9
Late maternal deaths	O96-O97.9	
Maternal deaths aggravated by HIV/AIDS		
Other maternal disorders	C58-C58.0, N98-N98.9, O09-O09.9, O21-O22.9, O26-O26.9, O28-O31.8, O34-O36.9, O40-O43.1, O43.8-O43.9, O47-O48.1, O60-O61.9, O63-O63.9, O68-O69.9, O70.0-O70.9, O73-O77.9, O80-O84.9, O87-O90.9, O92-O92.7	181-181.9, 643-645.2, 650-651.9, 654-659.2, 659.4-659.9, 662-664.9, 665.4-665.9, 667-669.9, 671-679.1
Neonatal disorders	P00-P04.2, P04.5-P05.9, P07-P15.9, P19-P22.9, P24-P29.9, P36-P36.9, P38-P39.9, P50-P61.9, P70-P70.1, P70.3-P72.9, P74-P78.9, P80-P81.9, P83-P84, P90-P94.9, P96, P96.3-P96.4, P96.8	760-760.6, 760.8-768, 768.2-770, 770.1-771, 771.4-775.0, 775.4-779.3, 779.6-779.8
Neonatal preterm birth	P01.0-P01.1, P07-P07.3, P22-P22.9, P25-P28.9, P61.2, P77-P77.9	761.0-761.1, 765-765.9, 769-769.9, 770.2-770.9, 776.6, 777.5-777.6
Neonatal encephalopathy due to birth asphyxia and trauma	P01.7, P02-P03.9, P10-P15.9, P20-P21.9, P24-P24.9, P90-P91.9	761.7-763.9, 767-768, 768.2-768.9, 770.1, 772.1-772.9, 779.0-779.2
Neonatal sepsis and other neonatal infections	P36-P36.9, P38-P39.9	771.4-771.9
Hemolytic disease and other neonatal jaundice	P55-P59.9	773-774.9
Other neonatal disorders	P00-P01, P01.2-P01.6, P01.8-P01.9, P04-P04.2, P04.5-P05.9, P08-P09, P19-P19.9, P29-P29.9, P50-P54.9, P60-P61.1, P61.3-P61.9, P70-P70.1, P70.3-P72.9, P74-P76.9, P78-P78.9, P80-P81.9, P83-P84, P92-P94.9, P96, P96.3-P96.4, P96.8	760-760.6, 760.8-761, 761.2-761.6, 764-764.9, 766-766.9, 770, 771, 772-772.0, 775-775.0, 775.4-776.5, 776.7-777.4, 777.7-779, 779.3, 779.6-779.8
Nutritional deficiencies	D50.1-D50.8, D51-D52.0, D52.8-D53.9, E00-E02, E40-E46.9, E51-E61.9, E63-E64.0, E64.2-E64.9, M12.1	244.2, 260-263.9, 265-269.9, 281.0-281.9, 716.0
Protein-energy malnutrition	E40-E46.9, E64.0	260-263.9
Other nutritional deficiencies	D51-D52.0, D52.8-D53.9, E00-E02, E51-E61.9, E63-E64, E64.2-E64.9, M12.1	244.2, 265-269.9, 281.0-281.9, 716.0

Table S2: List of International Classification of Diseases (ICD) codes mapped to the Global Burden of Disease cause list for causes of death		
**Cause**	**ICD10**	**ICD9**
Non-communicable diseases	A46-A46.0, A66-A67.9, B18-B18.9, B33.2, B86, C00-C13.9, C15-C22.8, C23-C25.9, C30-C34.9, C37-C38.8, C40-C41.9, C43-C45.9, C47-C54.9, C56-C57.8, C60-C63.8, C64-C67.9, C68.0-C68.8, C69.0-C69.8, C70-C73.9, C75-C75.8, C81-C86.6, C88-C91.0, C91.2-C91.3, C91.6, C92-C92.6, C93-C93.1, C93.3, C93.8, C94-C96.9, D00.1-D00.2, D01.0-D01.3, D02.0-D02.3, D03-D06.9, D07.0-D07.2, D07.4-D07.5, D09.0, D09.2-D09.3, D09.8, D10.0-D10.7, D11-D12.9, D13.0-D13.7, D14.0-D14.3, D15-D16.9, D22-D27.9, D28.0-D28.7, D29.0-D29.8, D30.0-D30.8, D31-D36, D36.1-D36.7, D37.1-D37.5, D38.0-D38.5, D39.1-D39.2, D39.8, D40.0-D40.8, D41.0-D41.8, D42-D43.9, D44.0-D44.8, D45-D47.9, D48.0-D48.6, D49.2-D49.4, D49.6, D52.1, D55-D58.9, D59.0-D59.3, D59.5-D59.6, D60-D61.9, D63.1, D64.0, D66-D67, D68.0-D69.8, D70-D70.2, D70.4-D75.8, D76-D78.8, D86-D86.9, D89-D89.2, E03-E07.1, E09-E11.9, E15.0, E16.0-E16.9, E20-E34, E34.1-E34.8, E36-E36.8, E65-E68, E70-E85.2, E88-E89.9, F00-F02.0, F02.2-F02.3, F02.8-F03.9, F10-F16.9, F18-F18.9, F24, F50.0-F50.5, G10-G13.8, G20-G20.9, G21.0-G21.1, G23-G26.0, G30-G31.9, G35-G37.9, G40-G41.9, G45-G46.8, G47.3, G61-G61.9, G62.1, G70-G73.7, G90-G90.9, G93.7, G95-G95.9, G97-G97.9, H05.0-H05.1, I01-I01.9, I02.0, I05-I09.9, I11-I13.9, I20-I25.9, I27.0-I27.2, I28-I28.9, I30-I31.1, I31.8-I37.8, I38-I41.9, I42.1-I42.8, I43-I43.9, I47-I48.9, I51.0-I51.4, I60-I63.9, I65-I66.9, I67.0-I67.3, I67.5-I67.7, I68.0-I68.2, I69.0-I69.3, I70.2-I70.8, I71-I73.9, I77-I89.9, I95.2-I95.3, I97-I98, I98.2, I98.9, J30-J35.9, J37-J39.9, J41-J46.9, J60-J63.8, J65-J68.9, J70-J70.9, J82, J84-J84.9, J91, J91.8-J92.9, J95-J95.9, K20-K20.9, K22-K22.6, K22.8-K29.9, K31-K31.8, K35-K38.9, K40-K46.9, K50-K52.0, K52.2-K52.9, K55-K62.9, K63.5, K64-K64.9, K66.8, K67, K68, K70-K70.3, K71.7, K73-K75, K75.1-K75.2, K75.4-K76.2, K76.4-K77, K77.8, K80-K83.9, K85-K86.9, K90-K91.9, K92.8, K93.8-K95.8, L00-L05.9, L08-L08.9, L10-L14.0, L51-L51.9, L88-L89.9, L93-L93.2, L97-L98.4, M00-M03.0, M03.2-M03.6, M05-M09.8, M30-M36.8, M40-M43.1, M65-M65.0, M71.0-M71.1, M72.5-M72.6, M80-M82.8, M86.3-M86.4, M87-M87.1, M88-M89.0, M89.5, M89.7-M89.9, N00-N08.8, N10-N12.9, N13.6, N14-N16.8, N18-N18.9, N20-N23.0, N25-N28.1, N29-N30.3, N30.8-N32.0, N32.3-N32.4, N34-N34.3, N36-N36.9, N39-N39.2, N41-N41.9, N44-N44.0, N45-N45.9, N49-N49.9, N60-N60.9, N65-N65.1, N72-N72.0, N75-N77.8, N80-N81.9, N83-N83.9, N84.0-N84.1, N87-N87.9, N99-N99.9, P04.3-P04.4, P70.2, P96.0-P96.2, P96.5, Q00-Q07.9, Q10.4-Q18.9, Q20-Q28.9, Q30-Q36, Q37-Q45.9, Q50-Q87.8, Q89-Q89.8, Q90-Q93.9, Q95-Q99.8, R50.2, R78.0-R78.5, R95-R95.9, X45-X45.9, X65-X65.9, Y15-Y15.9	035-035.9, 036.4, 102-103.9, 133-133.6, 135-135.9, 140-148.9, 150-155.1, 155.3-158.9, 160-164.9, 170-175.9, 180-180.9, 182-183.8, 184.0-184.4, 184.8, 185-186.9, 187.1-187.8, 188-188.9, 189.0-189.8, 190-190.8, 191-193.9, 194.1-194.8, 200-204.0, 204.2, 205-205.3, 206-206.1, 207-208.9, 209.0-209.1, 209.4-209.5, 210.0-210.9, 211.0-211.8, 212.0-212.8, 213-213.9, 217-220.9, 221.0-221.8, 222.0-222.8, 223.0-223.8, 224-228.9, 229.0, 229.8, 230.1-230.8, 231.0-231.2, 232-232.9, 233.0-233.2, 233.4-233.5, 233.7, 234.0-234.8, 235.0, 235.4, 235.6-235.8, 236.0-236.2, 236.4-236.5, 236.7, 237-237.3, 237.5-237.9, 238.0-238.9, 239.2-239.4, 239.6, 240-243.9, 244.0-244.1, 244.3-244.8, 245-246.9, 251-259.1, 259.3-259.9, 270-273.9, 275-276, 277-277.2, 277.4-277.9, 278.0-278.8, 282-284.9, 286-286.5, 286.7-289.0, 289.4-289.7, 290-292.9, 294.1-294.9, 303-303.9, 304.0-304.8, 305.0, 305.2-305.8, 307.1, 327.2-327.8, 330-331.2, 331.5-332.0, 333-337.9, 340-341.9, 345-345.9, 349, 349.2-349.8, 353.8-353.9, 356-356.9, 357.0-357.1, 357.3-357.7, 358-359.9, 376.0-376.1, 391-391.9, 392.0, 393-398.9, 402-404.9, 410-414.9, 416.0-416.1, 417-417.9, 420-423, 423.1-423.9, 424.0-424.3, 424.8, 425.0-425.3, 425.5, 425.7-425.8, 427.0-427.3, 427.6-427.8, 429.0, 430-435.9, 437.0-437.2, 437.4-437.8, 440.2, 440.4, 441-443.9, 446-457, 457.1-457.9, 459, 459.1-459.3, 470, 470.9-474.9, 476-476.1, 477-479, 491-493.9, 495-504.9, 506-506.9, 508-509, 515, 516-517.8, 518.6-518.7, 518.9, 519.0-519.4, 530-530.0, 530.2-530.6, 531-536.1, 536.4, 537-537.6, 537.8, 538-543.9, 550-553.6, 555-558.9, 560-560.3, 560.8-560.9, 562-562.1, 564-564.7, 565-566.9, 569.0-569.7, 571-571.9, 572.2-573.0, 573.4-577.9, 579-583.9, 585-585.9, 588-590.9, 592-593.8, 594-599.6, 599.8, 601-602.9, 604-604.9, 608.2, 610-610.9, 617-618.9, 620-620.9, 621.4-621.9, 622.1-622.7, 629-629.8, 680-689, 694-695.5, 707-707.9, 710-711.9, 714-714.3, 714.8-714.9, 730.1, 732-732.9, 733.0-733.1, 740-749.0, 749.2-758.9, 759.0-759.8, 760.7, 775.1-775.3, 779.4-779.5, 788.0, 790.3, 798-798.0, E850, E860
Neoplasms	C00-C13.9, C15-C22.8, C23-C25.9, C30-C34.9, C37-C38.8, C40-C41.9, C43-C45.9, C47-C54.9, C56-C57.8, C60-C63.8, C64-C67.9, C68.0-C68.8, C69.0-C69.8, C70-C73.9, C75-C75.8, C81-C86.6, C88-C91.0, C91.2-C91.3, C91.6, C92-C92.6, C93-C93.1, C93.3, C93.8, C94-C96.9, D00.1-D00.2, D01.0-D01.3, D02.0-D02.3, D03-D06.9, D07.0-D07.2, D07.4-D07.5, D09.0, D09.2-D09.3, D09.8, D10.0-D10.7, D11-D12.9, D13.0-D13.7, D14.0-D14.3, D15-D16.9, D22-D24.9, D26.0-D27.9, D28.0-D28.1, D28.7, D29.0-D29.8, D30.0-D30.8, D31-D36, D36.1-D36.7, D37.1-D37.5, D38.0-D38.5, D39.1-D39.2, D39.8, D40.0-D40.8, D41.0-D41.8, D42-D43.9, D44.0-D44.8, D45-D47.9, D48.0-D48.6, D49.2-D49.4, D49.6, K62.0-K62.1, K63.5, N60-N60.9, N84.0-N84.1, N87-N87.9	140-148.9, 150-155.1, 155.3-158.9, 160-164.9, 170-175.9, 180-180.9, 182-183.8, 184.0-184.4, 184.8, 185-186.9, 187.1-187.8, 188-188.9, 189.0-189.8, 190-190.8, 191-193.9, 194.1-194.8, 200-204.0, 204.2, 205-205.3, 206-206.1, 207-208.9, 209.0-209.1, 209.4-209.5, 210.0-210.9, 211.0-211.8, 212.0-212.8, 213-213.9, 217-217.8, 219.0, 220-220.9, 221.0-221.8, 222.0-222.8, 223.0-223.8, 224-228.9, 229.0, 229.8, 230.1-230.8, 231.0-231.2, 232-232.9, 233.0-233.2, 233.4-233.5, 233.7, 234.0-234.8, 235.0, 235.4, 235.6-235.8, 236.1-236.2, 236.4-236.5, 236.7, 237-237.3, 237.5-237.9, 238.0-238.9, 239.2-239.4, 239.6, 569.0, 610-610.9, 622.1-622.2, 622.7
Lip and oral cavity cancer	C00-C08.9, D10.0-D10.5, D11-D11.9	140-145.9, 210.0-210.6, 235.0
Nasopharynx cancer	C11-C11.9, D10.6	147-147.9, 210.7-210.9
Other pharynx cancer	C09-C10.9, C12-C13.9, D10.7	146-146.9, 148-148.9
Oesophageal cancer	C15-C15.9, D00.1, D13.0	150-150.9, 211.0, 230.1
Stomach cancer	C16-C16.9, D00.2, D13.1, D37.1	151-151.9, 211.1, 230.2
Colon and rectum cancer	C18-C21.9, D01.0-D01.3, D12-D12.9, D37.3-D37.5	153-154.9, 209.1, 209.5, 211.3-211.4, 230.3-230.6, 569.0
Liver cancer	C22-C22.8, D13.4	155-155.1, 155.3-155.9, 211.5
Liver cancer due to hepatitis B		
Liver cancer due to hepatitis C		
Liver cancer due to alcohol use		
Liver cancer due to NASH		
Hepatoblastoma	C22.2	
Liver cancer due to other causes (internal)		
Gallbladder and biliary tract cancer	C23-C24.9, D13.5	156-156.9
Pancreatic cancer	C25-C25.9, D13.6-D13.7	157-157.9, 211.6-211.7
Larynx cancer	C32-C32.9, D02.0, D14.1, D38.0	161-161.9, 212.1, 231.0, 235.6
Tracheal, bronchus, and lung cancer	C33-C34.9, D02.1-D02.3, D14.2-D14.3, D38.1	162-162.9, 212.2-212.3, 231.1-231.2, 235.7
Malignant skin melanoma	C43-C43.9, D03-D03.9, D22-D23.9, D48.5	172-172.9
Non-melanoma skin cancer	C44-C44.9, D04-D04.9, D49.2	173-173.9, 222.4, 232-232.9, 238.2
Non-melanoma skin cancer (squamous-cell carcinoma)	C44-C44.9, D04-D04.9, D49.2	173-173.9, 222.4, 232-232.9, 238.2
Soft tissue and other extraosseous sarcomas	C49-C49.9	171-171.9
Malignant neoplasm of bone and articular cartilage	C40-C41.9	170-170.9
Breast cancer	C50-C50.9, D05-D05.9, D24-D24.9, D48.6, D49.3	174-175.9, 217-217.8, 233.0, 238.3, 239.3, 610-610.9
Cervical cancer	C53-C53.9, D06-D06.9, D26.0	180-180.9, 219.0, 233.1, 622.1-622.2, 622.7
Uterine cancer	C54-C54.9, D07.0-D07.2, D26.1-D26.9	182-182.9, 233.2
Ovarian cancer	C56-C56.9, D27-D27.9, D39.1	183-183.0, 220-220.9, 236.2
Prostate cancer	C61-C61.9, D07.5, D29.1, D40.0	185-185.9, 222.2, 236.5
Testicular cancer	C62-C62.9, D29.2-D29.8, D40.1-D40.8	186-186.9, 222.0, 222.3, 236.4
Kidney cancer	C64-C65.9, D30.0-D30.1, D41.0-D41.1	189.0-189.1, 189.5-189.6, 223.0-223.1
Bladder cancer	C67-C67.9, D09.0, D30.3, D41.4-D41.8, D49.4	188-188.9, 223.3, 233.7, 236.7, 239.4
Brain and central nervous system cancer	C70-C72.9	191-192.9
Eye cancer	C69.0-C69.8	190-190.8
Retinoblastoma	C69.2	190.5
Other eye cancers	C69.0-C69.1, C69.3-C69.8	190-190.4, 190.6-190.8

Table S2: List of International Classification of Diseases (ICD) codes mapped to the Global Burden of Disease cause list for causes of death

Cause	ICD10	ICD9
Neuroblastoma and other peripheral nervous cell tumors	C47-C47.9	
Thyroid cancer	C73-C73.9, D09.3, D09.8, D34-D34.9, D44.0	193-193.9, 226-226.9
Mesothelioma	C45-C45.9	
Hodgkin lymphoma	C81-C81.9	201-201.9
Non-Hodgkin lymphoma	C82-C86.6, C96-C96.9	200-200.9, 202-202.9
Burkitt lymphoma	C83.7-C83.8	200.2
Other non-Hodgkin lymphoma	C82-C83.6, C83.9-C86.6, C96-C96.9	200-200.1, 200.3-200.9, 202-202.9
Multiple myeloma	C88-C90.9	203-203.9
Leukaemia	C91-C91.0, C91.2-C91.3, C91.6, C92-C92.6, C93-C93.1, C93.3, C93.8, C94-C95.9	204-204.0, 204.2, 205-205.3, 206-206.1, 207-208.9
Acute lymphoid leukaemia	C91.0, C91.2-C91.3, C91.6	204.0, 204.2
Chronic lymphoid leukaemia		
Acute myeloid leukaemia	C92.0, C92.3-C92.6, C93.0, C94.0, C94.2, C94.4-C94.5	205.0, 205.2-205.3, 206.0, 207.0, 207.2-207.8
Chronic myeloid leukaemia	C92.1-C92.2	205.1
Other leukaemia	C93.1, C93.3, C93.8, C94.1, C94.3, C94.6-C95.9	206.1, 207.1, 207.9-208.9
Other malignant neoplasms (internal)	C17-C17.9, C30-C31.9, C37-C38.8, C48-C48.9, C4A, C51-C52.9, C57-C57.8, C60-C60.9, C63-C63.8, C66-C66.9, C68.0-C68.8, C75-C75.8, D07.4, D09.2, D13.2-D13.3, D14.0, D15-D16.9, D28.0-D28.1, D28.7, D29.0, D30.2, D30.4-D30.8, D31-D31.9, D35-D35.2, D35.5-D36, D36.1-D36.7, D37.2, D38.2-D38.5, D39.2, D39.8, D41.2-D41.3, D44.1-D44.8, D48.0-D48.4	152-152.9, 158-158.9, 160-160.9, 163-164.9, 183.2-183.8, 184.0-184.4, 184.8, 187.1-187.8, 189.2-189.4, 189.8, 194.1-194.8, 209.0, 209.4, 211.2, 211.8, 212.0, 212.4-212.8, 213-213.9, 221.0-221.8, 222.1, 222.8, 223.2, 223.8, 224-224.9, 227-228.9, 229.0, 229.8, 230.7-230.8, 233.4-233.5, 234.0-234.8, 235.4, 235.8, 236.1, 238.0-238.1, 239.2
Other neoplasms	D32-D33.9, D35.3-D35.4, D42-D43.9, D45-D47.9, D49.6, K62.0-K62.1, K63.5, N60-N60.9, N84.0-N84.1, N87-N87.9	225-225.9, 237-237.3, 237.5-237.9, 238.4-238.9, 239.6
Myelodysplastic, myeloproliferative, and other haematopoietic neoplasms	D45-D47.9	238.4-238.9
Cardiovascular diseases	B33.2, G45-G46.8, I01-I01.9, I02.0, I05-I09.9, I11-I11.9, I20-I25.9, I27.0, I27.2, I28-I28.9, I30-I31.1, I31.8-I37.8, I38-I41.9, I42.1-I42.8, I43-I43.9, I47-I48.9, I51.0-I51.4, I60-I63.9, I65-I66.9, I67.0-I67.3, I67.5-I67.6, I68.0-I68.2, I69.0-I69.3, I70.2-I70.8, I71-I73.9, I77-I83.9, I86-I89.0, I89.9, I98, K75.1	036.4, 391-391.9, 392.0, 393-398.9, 402-402.9, 410-414.9, 416.0, 417-417.9, 420-423, 423.1-423.9, 424.0-424.3, 424.8, 425.0-425.3, 425.5, 425.7-425.8, 427.0-427.3, 427.6-427.8, 429.0, 430-435.9, 437.0-437.2, 437.5-437.8, 440.2, 440.4, 441-443.9, 447-454.9, 456, 456.3-457, 457.1, 457.8-457.9, 459, 459.1-459.3
Rheumatic heart disease	I01-I01.9, I02.0, I05-I09.9	391-391.9, 392.0, 393-398.9
Ischaemic heart disease	I20-I25.9	410-414.9
Stroke	G45-G46.8, I60-I63.9, I65-I66.9, I67.0-I67.3, I67.5-I67.6, I68.1-I68.2, I69.0-I69.3	430-435.9, 437.0-437.2, 437.5-437.8
Ischaemic stroke	G45-G46.8, I63-I63.9, I65-I66.9, I67.2-I67.3, I67.5-I67.6, I69.3	433-435.9, 437.0-437.1, 437.5-437.8
Intracerebral haemorrhage	I61-I62, I62.1-I62.9, I68.1-I68.2, I69.1-I69.2	431-432.9, 437.2
Subarachnoid hemorrhage	I60-I60.9, I62.0, I67.0-I67.1, I69.0	430-430.9
Hypertensive heart disease	I11-I11.9	402-402.9
Non-rheumatic valvular heart disease	I34-I37.8	424.0-424.3, 424.8
Non-rheumatic calcific aortic valvular heart disease	I35-I35.9	424.1
Non-rheumatic degenerative mitral valvular heart disease	I34-I34.9	424.0
Other non-rheumatic valvular heart diseases	I36-I37.8	424.2-424.3, 424.8
Cardiomyopathy and myocarditis	B33.2, I40-I41.9, I42.1-I42.8, I43-I43.9, I51.4	422-422.9, 425.0-425.3, 425.5, 425.7-425.8, 429.0
Myocarditis	B33.2, I40-I41.9, I51.4	422-422.9
Alcoholic cardiomyopathy	I42.6	425.5
Other cardiomyopathy	I42.1-I42.5, I42.7-I42.8, I43-I43.9	425.0-425.3, 425.7-425.8, 429.0
Pulmonary arterial hypertension	I27.0, I27.2	416.0
Atrial fibrillation and flutter	I48-I48.9	427.3
Aortic aneurysm	I71-I71.9	441-441.9
Peripheral artery disease	I70.2-I70.8, I73-I73.9	440.2, 440.4, 443.0-443.9
Endocarditis	I33-I33.9, I38-I39.9	421-421.9
Other cardiovascular and circulatory diseases (internal)	I28-I28.9, I30-I31.1, I31.8-I32.8, I47-I47.9, I51.0-I51.3, I68.0, I72-I72.9, I77-I83.9, I86-I89.0, I89.9, I98, K75.1	036.4, 417-417.9, 420-420.9, 423, 423.1-423.9, 427.0-427.2, 427.6-427.8, 442-443, 447-454.9, 456, 456.3-457, 457.1, 457.8-457.9, 459, 459.1-459.3
Chronic respiratory diseases	D86-D86.2, D86.9, G47.3, J30-J35.9, J37-J39.9, J41-J46.9, J60-J63.8, J65-J68.9, J70, J70.8-J70.9, J82, J84-J84.9, J91, J91.8-J92.9	135-135.9, 327.2-327.8, 470, 470.9-474.9, 476-476.1, 477-479, 491-493.9, 495-504.9, 506-506.9, 508-509, 515, 516-517.8, 518.6, 518.9, 519.1-519.4
Chronic obstructive pulmonary disease	J41-J44.9	491-492.9, 496-499
Pneumoconiosis	J60-J63.8, J65-J65.0, J92.0	500-504.9
Silicosis	J62-J62.9	502-502.9, 503.0, 503.9
Asbestosis	J61-J61.0, J92.0	501
Coal workers pneumoconiosis	J60-J60.0	500-500.9, 501.0-501.9
Other pneumoconiosis	J63-J63.8, J65-J65.0	503, 503.1, 504-504.9
Asthma	J45-J46.9	493-493.9
Interstitial lung disease and pulmonary sarcoidosis	D86-D86.2, D86.9, J84-J84.9	135-135.9, 515, 516-516.9
Other chronic respiratory diseases	G47.3, J30-J35.9, J37-J39.9, J66-J68.9, J70, J70.8-J70.9, J82, J91, J91.8-J92, J92.9	327.2-327.8, 470, 470.9-474.9, 476-476.1, 477-479, 495-495.9, 506-506.9, 508-509, 517-517.8, 518.6, 518.9, 519.1-519.4
Digestive diseases	B18-B18.9, I84-I85.9, I98.2, K20-K20.9, K22-K22.6, K22.8-K29.9, K31-K31.8, K35-K38.9, K40-K42.9, K44-K46.9, K50-K52, K52.2-K52.9, K55-K62, K62.2-K62.6, K62.8-K62.9, K64-K64.9, K66.8, K67, K68, K70-K70.3, K71.7, K73-K75, K75.2, K75.4-K76.2, K76.4-K77, K77.8, K80-K83.9, K85-K86.9, K90-K90.9, K92.8, K93.8, M09.1	455-455.9, 456.0-456.2, 530-530.0, 530.2-530.6, 531-536.1, 537-537.6, 537.8, 538, 540-543.9, 550-551.1, 551.3-552.1, 552.3-553.6, 555-558.9, 560-560.3, 560.8-560.9, 562-562.1, 564-564.1, 564.5-564.7, 565-566.9, 569.1-569.5, 569.7, 571-571.9, 572.2-573.0, 573.4-577.9, 579-579.2, 579.4-579.9
Cirrhosis and other chronic liver diseases	B18-B18.9, I85-I85.9, I98.2, K70-K70.3, K71.7, K73-K75, K75.2, K75.4-K76.2, K76.4-K76.9, K77.8	456.0-456.2, 571-571.9, 572.2-573.0, 573.4-573.9
Cirrhosis and other chronic liver diseases due to hepatitis B		
Cirrhosis and other chronic liver diseases due to hepatitis C		
Cirrhosis and other chronic liver diseases due to alcohol use		

Table S2: List of International Classification of Diseases (ICD) codes mapped to the Global Burden of Disease cause list for causes of death		
**Cause**	**ICD10**	**ICD9**
Cirrhosis and other chronic liver diseases due to NAFLD		
Cirrhosis and other chronic liver diseases due to other causes		
Upper digestive system diseases	K25-K29.9	531-535.9
Peptic ulcer disease	K25-K28.9	531-534.9
Gastritis and duodenitis	K29-K29.9	535-535.9
Appendicitis	K35-K37.9, K38.3-K38.9	540-542.9
Paralytic ileus and intestinal obstruction	K56-K56.9	560-560.3, 560.8-560.9
Inguinal, femoral, and abdominal hernia	K40-K42.9, K44-K46.9	550-551.1, 551.3-552.1, 552.3-553.0, 553.6
Inflammatory bowel disease	K50-K52, K52.8-K52.9, M09.1	555-556.9, 558-558.9, 569.5
Vascular intestinal disorders	K55-K55.9	557-557.9
Gallbladder and biliary diseases	K80-K83.9	574-576.9
Pancreatitis	K85-K86.9	577-577.9, 579.4
Other digestive diseases	I84-I84.9, K20-K20.9, K22-K22.6, K22.8-K24, K31-K31.8, K38-K38.2, K52.2-K52.3, K57-K62, K62.2-K62.6, K62.8-K62.9, K64-K64.9, K66.8, K67, K68, K77, K90-K90.9, K92.8, K93.8	455-455.9, 530-530.0, 530.2-530.6, 536-536.1, 537-537.6, 537.8, 538, 543-543.9, 553.1-553.3, 562-562.1, 564-564.1, 564.5-564.7, 565-566.9, 569.1-569.4, 569.7, 579-579.2, 579.8-579.9
Neurological disorders	F00-F02.0, F02.2-F02.3, F02.8-F03.9, G10-G13.8, G20-G20.9, G23-G24, G24.1-G25.0, G25.2-G25.3, G25.5, G25.8-G26.0, G30-G31.1, G31.8-G31.9, G35-G37.9, G40-G41.9, G61-G61.9, G70-G71.1, G71.3-G72, G72.2-G73.7, G90-G90.9, G95-G95.9, M33-M33.9	290-290.9, 294.1-294.9, 330-331.2, 331.5-332.0, 333-337.9, 340-341.9, 345-345.9, 349, 349.2-349.8, 353.8-353.9, 356-356.9, 357.0-357.1, 357.3-357.4, 357.7, 358-359.9, 775.2
Alzheimer's disease and other dementias	F00-F02.0, F02.8-F03.9, G30-G31.1, G31.8-G31.9	290-290.9, 294.1-294.9, 331-331.2
Parkinson's disease	F02.3, G20-G20.9	332-332.0
Idiopathic epilepsy	G40-G41.9	345-345.9
Multiple sclerosis	G35-G35.9	340-340.9
Motor neuron disease	G12.2-G12.9	335-335.2, 335.8-335.9
Other neurological disorders	F02.2, G10-G12.1, G13-G13.8, G23-G24, G24.1-G25.0, G25.2-G25.3, G25.5, G25.8-G26.0, G36-G37.9, G61-G61.9, G70-G71.1, G71.3-G72, G72.2-G73.7, G90-G90.9, G95-G95.9, M33-M33.9	330-330.9, 331.5-331.9, 333-334.9, 335.3, 336-337.9, 341-341.9, 349, 349.2-349.8, 353.8-353.9, 356-356.9, 357.0-357.1, 357.3-357.4, 357.7, 358-359.9, 775.2
Mental disorders	F24, F50.0-F50.5	307.1
Eating disorders	F50.0-F50.5	307.1
Anorexia nervosa	F50.0-F50.1	307.1
Bulimia nervosa	F50.2-F50.5	
Substance use disorders	E24.4, F10-F16.9, F18-F18.9, G31.2, G62.1, G72.1, P04.3-P04.4, P96.1, Q86.0, R78.0-R78.5, X45-X45.9, X65-X65.9, Y15-Y15.9	291-292.9, 303-303.9, 304.0-304.8, 305.0, 305.2-305.8, 357.5, 760.7, 790.3, E850, E860
Alcohol use disorders	E24.4, F10-F10.9, G31.2, G62.1, G72.1, P04.3, Q86.0, R78.0, X45-X45.9, X65-X65.9, Y15-Y15.9	291-291.9, 303-303.9, 305.0, 357.5, 790.3, E860
Drug use disorders	F11-F16.9, F18-F18.9, P04.4, P96.1, R78.1-R78.5	292-292.9, 304.0-304.8, 305.2-305.8, 760.7, E850
Opioid use disorders	F11-F11.9, P96.1, R78.1	304.0, 305.5
Cocaine use disorders	F14-F14.9, R78.2	304.2, 305.6
Amphetamine use disorders	F15-F15.9	304.4, 305.7
Other drug use disorders	F13-F13.9, F16-F16.9, F18-F18.9, P04.4, R78.3-R78.5	292-292.9, 304.1, 304.5-304.8, 305.3-305.4, 305.8, 760.7
Diabetes and kidney diseases	D63.1, E10-E11.9, I12-I13.9, N00-N08.8, N15.0, N18-N18.9, P70.2, Q61-Q62.8	403-404.9, 580-583.9, 585-585.9, 589-589.9, 753-753.3, 775.1
Diabetes mellitus	E10-E10.1, E10.3-E11.1, E11.3-E11.9, P70.2	775.1
Diabetes mellitus type 1	E10-E10.1, E10.3-E10.9, P70.2	775.1
Diabetes mellitus type 2	E11-E11.1, E11.3-E11.9	
Chronic kidney disease	D63.1, E10.2, E11.2, I12-I13.9, N02-N08.8, N15.0, N18-N18.9, Q61-Q62.8	403-404.9, 581-583.9, 585-585.9, 589-589.9, 753-753.3
Chronic kidney disease due to diabetes mellitus type 1	E10.2	
Chronic kidney disease due to diabetes mellitus type 2	E11.2	
Chronic kidney disease due to hypertension	I12-I13.9	403-404.9
Chronic kidney disease due to glomerulonephritis	N03-N06.9	581-583.9
Chronic kidney disease due to other and unspecified causes	N02-N02.9, N07-N08.8, N15.0, Q61-Q62.8	589-589.9, 753-753.3
Acute glomerulonephritis	N00-N01.9	580-580.9
Skin and subcutaneous diseases	A46-A46.0, A66-A67.9, B86, D86.3, I89.1-I89.8, L00-L05.9, L08-L08.9, L10-L14.0, L51-L51.9, L88-L89.9, L97-L98.4, M72.5-M72.6	035-035.9, 102-103.9, 133-133.6, 457.2-457.3, 680-689, 694-695.3, 707-707.9
Bacterial skin diseases	A46-A46.0, A66-A67.9, I89.1-I89.8, L00-L05.9, L08-L08.9, L88, L97-L98.4, M72.5-M72.6	035-035.9, 102-103.9, 457.2-457.3, 680-689
Cellulitis	L03-L03.9, M72.5-M72.6	681-682.9
Pyoderma	A46-A46.0, A66-A67.9, I89.1-I89.8, L00-L02.9, L04-L05.9, L08-L08.9, L88, L97-L98.4	035-035.9, 102-103.9, 457.2-457.3, 680-680.9, 683-689
Decubitus ulcer	L89-L89.9	707-707.9
Other skin and subcutaneous diseases	D86.3, L10-L14.0, L51-L51.9	694-695.3
Musculoskeletal disorders	I27.1, I67.7, L93-L93.2, M00-M03.0, M03.2-M03.6, M05-M09.0, M09.2-M09.8, M30-M32.9, M34-M36.8, M40-M43.1, M65-M65.0, M71.0-M71.1, M80-M82.8, M86.3-M86.4, M87-M87.0, M88-M89.0, M89.5, M89.7-M89.9	416.1, 437.4, 446-446.9, 695.4-695.5, 710-711.9, 714-714.3, 714.8-714.9, 730.1, 732-732.9, 733.0-733.1
Rheumatoid arthritis	M05-M06.9, M08.0-M08.8	714-714.3, 714.8-714.9
Other musculoskeletal disorders	I27.1, I67.7, L93-L93.2, M00-M03.0, M03.2-M03.6, M07-M08, M08.9-M09.0, M09.2-M09.8, M30-M32.9, M34-M36.8, M40-M43.1, M65-M65.0, M71.0-M71.1, M80-M82.8, M86.3-M86.4, M87-M87.0, M88-M89.0, M89.5, M89.7-M89.9	416.1, 437.4, 446-446.9, 695.4-695.5, 710-711.9, 730.1, 732-732.9, 733.0-733.1

Table S2: List of International Classification of Diseases (ICD) codes mapped to the Global Burden of Disease cause list for causes of death		
Cause	ICD10	ICD9
Other non-communicable diseases	D25-D26, D28.2, D52.1, D55-D58.9, D59.0-D59.3, D59.5-D59.6, D60-D61.9, D64.0, D66-D67, D68.0-D69.8, D70-D70.2, D70.4-D75.8, D76-D78.8, D86.8, D89-D89.2, E03-E07.1, E09-E09.9, E15.0, E16.0-E16.9, E20-E24.3, E24.8-E34, E34.1-E34.8, E36-E36.8, E65-E68, E70-E85.2, E88-E89.9, G21.0-G21.1, G24.0, G25.1, G25.4, G25.6-G25.7, G71.2, G72.0, G93.7, G97-G97.9, I95.2-I95.3, I97-I97.9, I98.9, J70.0-J70.5, J95-J95.9, K43-K43.9, K52.0, K62.7, K91-K91.9, K94-K95.8, M87.1, N10-N12.9, N13.6, N14-N15, N15.1-N16.8, N20-N23.0, N25-N28.1, N29-N30.3, N30.8-N32.0, N32.3-N32.4, N34-N34.3, N36-N36.9, N39-N39.2, N41-N41.9, N44-N44.0, N45-N45.9, N49-N49.9, N65-N65.1, N72-N72.0, N75-N77.8, N80-N81.9, N83-N83.9, N99-N99.9, P96.0, P96.2, P96.5, Q00-Q07.9, Q10.4-Q18.9, Q20-Q28.9, Q30-Q36, Q37-Q45.9, Q50-Q60.6, Q63-Q86, Q86.1-Q87.8, Q89-Q89.8, Q90-Q93.9, Q95-Q99.8, R50.2, R95-R95.9	218-219, 219.1-219.9, 236.0, 240-243.9, 244.0-244.1, 244.3-244.8, 245-246.9, 251-259.1, 259.3-259.9, 270-273.9, 275-276, 277-277.2, 277.4-277.9, 278.0-278.8, 282-284.9, 286-286.5, 286.7-289.0, 289.4-289.7, 357.6, 518.7, 519.0, 536.4, 539-539.9, 551.2, 552.2, 564.2-564.4, 569.6, 579.3, 588-588.9, 590-590.9, 592-593.8, 594-599.6, 599.8, 601-602.9, 604-604.9, 608.2, 617-618.9, 620-620.9, 621.4-621.9, 622.3-622.6, 629-629.8, 740-749.0, 749.2-752.9, 753.4-758.9, 759.0-759.8, 775.3, 779.4-779.5, 788.0, 798-798.0
Congenital birth defects	G71.2, P96.0, Q00-Q07.9, Q10.4-Q18.9, Q20-Q28.9, Q30-Q36, Q37-Q45.9, Q50-Q60.6, Q63-Q86, Q86.1-Q87.8, Q89-Q89.8, Q90-Q93.9, Q95-Q99.8	740-749.0, 749.2-752.9, 753.4-758.9, 759.0-759.8
Neural tube defects	Q00-Q01.9, Q05-Q05.9	740-741.9, 742.0
Congenital heart anomalies	Q20-Q28.9	745-747.9
Orofacial clefts	Q35-Q36, Q37-Q37.9	749-749.0, 749.2-749.9
Down syndrome	Q90-Q90.9	758.0
Other chromosomal abnormalities	Q87-Q87.8, Q91-Q93.9, Q95-Q95.9, Q97-Q97.9, Q99-Q99.8	758, 758.1-758.6, 758.8-758.9
Congenital musculoskeletal and limb anomalies	Q65-Q79, Q79.6-Q79.9	742.5, 754-756.5, 756.8-756.9
Urogenital congenital anomalies	P96.0, Q50-Q60.6, Q63-Q64.9	752-752.9, 753.4-753.9
Digestive congenital anomalies	Q38-Q45.9, Q79.0-Q79.5	750-751.9, 756.6-756.7
Other congenital birth defects	G71.2, Q02-Q04.9, Q06-Q07.9, Q10.4-Q18.9, Q30-Q34.9, Q80-Q86, Q86.1-Q86.8, Q89-Q89.8	742, 742.1-742.4, 742.8-744.9, 748-748.9, 757-757.9, 759.0-759.8
Urinary diseases and male infertility	N10-N12.9, N13.6, N15, N15.1-N16.8, N20-N23.0, N25-N28.1, N29-N30.3, N30.8-N32.0, N32.3-N32.4, N34-N34.3, N36-N36.9, N39-N39.2, N41-N41.9, N44-N44.0, N45-N45.9, N49-N49.9	588-588.9, 590-590.9, 592-593.8, 594-598.1, 598.8-599.6, 599.8, 601-602.9, 604-604.9, 608.2, 788.0
Urinary tract infection and interstitial nephritis	N10-N12.9, N13.6, N15, N15.1-N16.8, N30-N30.3, N30.8-N30.9, N34-N34.3, N39.0-N39.2	590-590.9, 595-595.9, 597-597.9, 599.0
Urolithiasis	N20-N23.0	592-592.9, 594-594.9, 788.0
Other urinary diseases	N25-N28.1, N29-N29.8, N31-N32.0, N32.3-N32.4, N36-N36.9, N39, N41-N41.9, N44-N44.0, N45-N45.9, N49-N49.9	588-588.9, 593-593.8, 596-596.9, 598-598.1, 598.8-599, 599.1-599.6, 599.8, 601-602.9, 604-604.9, 608.2
Gynaecological diseases	D25-D26, D28.2, E28.2, N72-N72.0, N75-N77.8, N80-N81.9, N83-N83.9	218-219, 219.1-219.9, 236.0, 256.4, 617-618.9, 620-620.9, 621.4-621.9, 622.3-622.6, 629-629.8
Uterine fibroids	D25-D26, D28.2	218-219, 219.1-219.9, 236.0
Endometriosis	N80-N80.9	617-617.9
Genital prolapse	N81-N81.9	618-618.9
Other gynaecological diseases	N72-N72.0, N75-N77.8, N83-N83.9	620-620.9, 621.4-621.9, 622.3-622.6, 629-629.8
Haemoglobinopathies and haemolytic anaemias	D55-D58.9, D59.1, D59.3, D59.5, D60-D61.9, D64.0	282-284.9
Thalassaemias	D56-D56.9	282.4-282.5
Sickle cell disorders	D57-D57.8	282.6
G6PD deficiency	D55-D55.2	282.2-282.3
Other haemoglobinopathies and haemolytic anaemias	D55.3-D55.9, D58-D58.9, D59.1, D59.3, D59.5, D60-D61.9, D64.0	282-282.1, 282.7-284.9
Endocrine, metabolic, blood, and immune disorders	D52.1, D59.0, D59.2, D59.6, D66-D67, D68.0-D69.8, D70-D70.2, D70.4-D75.8, D76-D78.8, D86.8, D89-D89.2, E03-E07.1, E09-E09.9, E15.0, E16.0-E16.9, E20-E24.3, E24.8-E28.1, E28.3-E34, E34.1-E34.8, E36-E36.8, E65-E68, E70-E85.2, E88-E89.9, G21.0-G21.1, G24.0, G25.1, G25.4, G25.6-G25.7, G72.0, G93.7, G97-G97.9, I95.2-I95.3, I97-I97.9, I98.9, J70.0-J70.5, J95-J95.9, K43-K43.9, K52.0, K62.7, K91-K91.9, K94-K95.8, M87.1, N14-N14.4, N65-N65.1, N99-N99.9, P96.2, P96.5, R50.2	240-243.9, 244.0-244.1, 244.3-244.8, 245-246.9, 251-256.3, 256.8-259.1, 259.3-259.9, 270-273.9, 275-276, 277-277.2, 277.4-277.9, 278.0-278.8, 286-286.5, 286.7-289.0, 289.4-289.7, 357.6, 518.7, 519.0, 536.4, 539-539.9, 551.2, 552.2, 564.2-564.4, 569.6, 579.3, 598.2, 775.3, 779.4-779.5
Sudden infant death syndrome	R95-R95.9	798-798.0
Injuries	L55-L55.9, L56.3, L56.8-L56.9, L58-L58.9, N30.4, U00-U03, V00-V86.9, V87.2-V87.3, V88.2-V88.3, V90-V98.8, W00-W46.2, W49-W62.9, W64-W70.9, W73-W75.9, W77-W81.9, W83-W94.9, W97.9, W99-X06.9, X08-X39.9, X47-X48.9, X50-X54.9, X57-X58.9, X60-X64.9, X66-X83.9, X85-Y08.9, Y35-Y84.9, Y87.0-Y87.1, Y88-Y88.3, Y89.0-Y89.1	349.0-349.1, 457.0, E800-E807, E830-E838, E840-E849, E856-E857, E861-E865, E867-E869, E870-E876, E878-E879, E880-E886, E888-E928, E930-E979, E990-E999
Transport injuries	V00-V86.9, V87.2-V87.3, V88.2-V88.3, V90-V98.8	E800-E807, E830-E838, E840-E849
Road injuries	V01-V04.9, V06-V80.9, V82-V82.9, V87.2-V87.3	
Pedestrian road injuries	V01-V04.9, V06-V09.9	
Cyclist road injuries	V10-V19.9	
Motorcyclist road injuries	V20-V29.9	
Motor vehicle road injuries	V30-V79.9, V87.2-V87.3	
Other road injuries	V80-V80.9, V82-V82.9	
Other transport injuries	V00-V00.8, V05-V05.9, V81-V81.9, V83-V86.9, V88.2-V88.3, V90-V98.8	E800-E807, E830-E838, E840-E849
Unintentional injuries	L55-L55.9, L56.3, L56.8-L56.9, L58-L58.9, N30.4, W00-W46.2, W49-W62.9, W64-W70.9, W73-W75.9, W77-W81.9, W83-W94.9, W97.9, W99-X06.9, X08-X39.9, X47-X48.9, X50-X54.9, X57-X58.9, Y40-Y84.9, Y88-Y88.3	349.0-349.1, 457.0, E856-E857, E861-E865, E867-E869, E870-E876, E878-E879, E880-E886, E888-E928, E930-E949
Falls	W00-W19.9	E880-E886, E888
Drowning	W65-W70.9, W73-W74.9	E910
Fire, heat, and hot substances	X00-X06.9, X08-X19.9	E890-E899, E924
Poisonings	X47-X48.9	E856-E857, E861-E865, E867-E869
Poisoning by carbon monoxide	X47-X47.9	E862, E868-E869
Poisoning by other means	X48-X48.9	E856-E857, E861, E863-E865, E867
Exposure to mechanical forces	W20-W38.9, W40-W43.9, W45.0-W45.2, W46-W46.2, W49-W52	E916-E922
Unintentional firearm injuries	W32-W34.9	E922

Table S2: List of International Classification of Diseases (ICD) codes mapped to the Global Burden of Disease cause list for causes of death

Cause	ICD10	ICD9
Other exposure to mechanical forces	W20-W31.9, W35-W38.9, W40-W43.9, W45.0-W45.2, W46-W46.2, W49-W52	E916-E921
Adverse effects of medical treatment	N30.4, Y40-Y84.9, Y88-Y88.3	349.0-349.1, 457.0, E870-E876, E878-E879, E930-E949
Animal contact	W52.0-W62.9, W64-W64.9, X20-X29.9	E905-E906
Venomous animal contact	X20-X29.9	E905
Non-venomous animal contact	W52.0-W62.9, W64-W64.9	E906
Foreign body	W44-W45, W45.3-W45.9, W75-W75.9, W78-W80.9, W83-W84.9	E911-E915
Pulmonary aspiration and foreign body in airway	W75-W75.9, W78-W80.9, W83-W84.9	E911-E913
Foreign body in other body part	W44-W45, W45.3-W45.9	E914-E915
Environmental heat and cold exposure	L55-L55.9, L56.3, L56.8-L56.9, L58-L58.9, W88-W94.9, W97.9, W99-W99.9, X30-X32.9, X39-X39.9	E900-E902, E926
Exposure to forces of nature	X33-X38.9	E907-E909
Still Born	P95-P95.9	768.0-768.1
Other unintentional injuries	W39-W39.9, W77-W77.9, W81-W81.9, W85-W87.9, X50-X54.9, X57-X58.9	E903-E904, E923, E925, E927-E928
Self-harm and interpersonal violence	U00-U03, X60-X64.9, X66-X83.9, X85-Y08.9, Y35-Y38.9, Y87.0-Y87.1, Y89.0-Y89.1	E950-E979, E990-E999
Self-harm	X60-X64.9, X66-X83.9, Y87.0	E950-E959
Self-harm by firearm	X72-X74.9	E955
Self-harm by other specified means	X60-X64.9, X66-X71.9, X75-X83.9, Y87.0	E950-E954, E956-E959
Interpersonal violence	X85-Y08.9, Y87.1	E960-E969
Physical violence by firearm	X93-X95.9	E965
Physical violence by sharp object	X99-X99.9	E966
Physical violence by other means	X85-X92.9, X96-X98.9, Y00-Y04.9, Y06-Y08.9, Y87.1	E961-E964, E967-E969
Conflict and terrorism	U00-U03, Y36-Y38.9, Y89.1	E979, E990-E999
Police conflict and executions	Y35-Y35.9, Y89.0	E970-E978
Garbage Code (GBD Level 1)	A40-A41.9, A48.0, A48.3, A49.0-A49.1, A59-A59.9, A71-A71.9, A74.0, B07-B07.9, B30-B30.9, B35-B36.9, B85-B85.4, B87-B88.9, B94.0, D50-D50.0, D50.9, D62-D63.0, D63.8-D64, D64.1-D65.9, D68, D69.9, E15, E16, E50-E50.9, E64.1, E85.3-E87.6, E87.8-E87.9, F06.2-F06.4, F07.2, F09-F09.9, F19-F23.9, F25-F49, F51-F99.0, G06-G08.0, G32-G32.8, G43-G44.2, G44.4-G44.8, G47-G47.2, G47.4-G47.9, G50-G60.9, G62-G62.0, G62.2-G65.2, G80-G83.9, G89-G89.4, G91-G91.2, G91.4-G93, G93.1-G93.2, G93.4-G93.6, G94.0-G94.8, G99-H05, H05.2-H69.9, H71-H99, I26-I26.9, I31.2-I31.4, I46-I46.9, I50.0-I50.4, I76, I95-I95.1, I95.8-I95.9, J69-J69.9, J80-J80.9, J81.0, J85-J85.3, J86-J86.9, J93-J93.1, J93.8-J93.9, J94.2, J96-J96.9, J98.1-J98.3, K00-K19, K30, K65-K66.1, K66.9, K68.1-K68.9, K71-K71.6, K71.8-K72.9, K75.0, L20-L30.9, L40-L50.9, L52-L54.8, L56-L56.2, L56.4-L56.5, L57-L57.9, L59-L68.9, L70-L76.8, L80-L87.9, L90-L92.9, L94-L96, L98.5-L99.8, M04, M10-M12.0, M12.2-M29, M37-M39, M43.2-M49, M49.2-M64, M65.1-M71, M71.2-M72.4, M72.8-M73, M73.8-M79.9, M83-M86.2, M86.5-M86.9, M87.2-M87.9, M89.1-M89.4, M90-M99.9, N17-N17.9, N19-N19.9, N32.1-N32.2, N32.8-N33.8, N35-N35.9, N37-N37.8, N39.3-N39.8, N42-N43.4, N44.1-N44.8, N46-N48.9, N50-N53.9, N61-N64.9, N82-N82.9, N91-N91.5, N95, N95.1-N95.9, N97-N97.9, R02-R02.9, R03.1, R07.0, R08-R09, R09.3, R11-R12.0, R14-R19.6, R19.8-R23, R23.1-R30.9, R32-R50.1, R50.8-R57.9, R58.0-R72.9, R74-R78, R78.6-R94.8, R96-R99.9, U05, U07-U81, U89.9-U99, X40-X44.9, X46-X46.9, X49-X49.9, Y10-Y14.9, Y16-Y19.9, Z00-Z15.8, Z17-unsp.	038-038.9, 040.0, 041.1, 076-078.2, 110-111.9, 125-125.3, 126-126.9, 127.2-127.9, 131-132.9, 133.8-134.9, 136.6, 139.1, 139.9, 247-248, 264-264.9, 274-274.9, 276.0-276.5, 276.7-276.9, 277.3, 280-281, 285-285.9, 286.6, 289.1-289.3, 293, 294-294.0, 295-302.9, 305, 305.9-307.0, 307.2-307.4, 307.6-319.9, 324-327.1, 328-329, 338-339.1, 339.3-339.8, 342-344.9, 346-346.9, 350-353.6, 354-355.9, 360-362, 362.1-376, 376.2-380.9, 384-389.9, 415-415.9, 423.0, 424, 424.4-424.5, 424.9, 427.5, 427.9-428.9, 437.3, 458-458.9, 459.0, 507-507.9, 510-510.9, 512-513.9, 518.1-518.2, 520-529.9, 536.3, 536.8-536.9, 537.7, 537.9, 564.8-564.9, 567-568.9, 570-570.9, 572-572.1, 573.1-573.3, 584-584.9, 586-587.9, 603-603.9, 605-608.1, 608.3-609, 611-612.1, 615-616.9, 619-619.9, 621-621.3, 622-622.0, 622.8-623.6, 623.8-624.5, 624.8-628.9, 629.9, 690-693.9, 695.8-706.9, 708-709.9, 712-713.8, 715-716, 716.2-721.6, 721.8-730.0, 730.2-730.3, 730.7-731.9, 733, 733.2-734.2, 737-738, 738.2-739.9, 780-782.4, 782.6-784.6, 784.9, 785.4-786, 786.6, 786.8, 787, 787.3-788, 788.3-789, 789.1-789.2, 789.5, 790-790.1, 790.4-796.1, 796.3-797.9, 798.1-799, 799.2-799.9, 999.0-999.9, E851-E855, E858, E866, E980-E982, V01-V08, V10-uns
Garbage Code (GBD Level 2)	A14.9, A29-A30.9, A45-A45.9, A47-A48, A48.8-A49, A49.3-A49.9, A61-A62, A72-A73, A76, A97, B08-B09, B11-B14, B28-B29, B31-B32.4, B34-B34.9, B61-B62, B68-B68.9, B73-B74.2, B76-B76.9, B78-B81.8, B84, B92-B94, B94.8-B94.9, B95.6-B97.3, B97.7-B99.9, D59, D59.4, D59.8-D59.9, F17-F17.9, G44.3, G91.3, G93.0, G93.3, I10-I10.9, I15-I15.9, I27, I27.8-I27.9, I50, I50.8-I50.9, I67.4, I70-I70.1, I70.9, I74-I75.8, J81, J81.1, J90-J90.0, J94-J94.1, J94.8-J94.9, K92.0-K92.2, N70-N71.9, N73-N74.0, N74.2-N74.8, R03-R03.0, R04-R06.9, R09.0-R09.2, R09.8-R10.9, R13-R13.9, R23.0, R58, S00-T98.3, W47-W48, W63, W71-W72, W76-W76.9, W82, W95-W97, W98, X07, X55-X56, X59-X59.9, Y20-Y34.9, Y86-Y87, Y87.2, Y89, Y89.9-Y99.9	000-000.9, 030-030.9, 041.2-041.9, 067-069, 078.8-078.9, 079.8-079.9, 089-089.9, 105-109.9, 119, 136.8-136.9, 139.8, 304, 304.9, 305.1, 339.2, 401-401.9, 405-405.9, 416, 416.2-416.9, 440-440.1, 440.3, 440.8-440.9, 444-445.8, 490-490.9, 494-494.9, 511-511.9, 514-514.9, 515.0-515.9, 518-518.0, 518.3-518.5, 518.8, 536.2, 578-578.9, 599.7, 613-614.9, 714.4, 716.1, 721.7, 735-736.9, 738.0-738.1, 784.7-784.8, 786.3, 787.0-787.2, 789.0, 789.3-789.4, 789.6-789.9, 796.2, 799.0-799.1, 800-999, E000-E80, E83, E839, E85, E859, E87, E877, E88, E887, E929, E983-E985, E988-E989

Table S2: List of International Classification of Diseases (ICD) codes mapped to the Global Burden of Disease cause list for causes of death

Cause	ICD10	ICD9
Garbage Code (GBD Level 3)	A01, A31-A31.9, A42-A44.9, A49.2, A64-A64.0, A99-A99.0, B17, B17.1, B17.8-B17.9, B19-B19.0, B19.2-B19.9, B37-B46.9, B49-B49.9, B55, B55.1-B55.9, B58-B59.9, B89, B94.2, C14-C14.9, C22.9, C26-C29, C35-C36, C39-C39.9, C42, C46-C46.9, C55-C55.9, C57.9, C59, C63.9, C68, C68.9, C74-C74.9, C75.9-C80.9, C87, C97-D00.0, D01, D01.4-D02, D02.4-D02.9, D07, D07.3, D07.6-D09, D09.1, D09.7, D09.9-D10, D10.9, D13, D13.9-D14, D14.4, D17-D21.9, D28, D28.9-D29, D29.9-D30, D30.9, D36.0, D36.9-D37.0, D37.6-D38, D38.6-D39.0, D39.7, D39.9-D40, D40.9-D41, D41.9, D44, D44.9, D48, D48.7-D49.1, D49.5, D49.7-D49.9, D54, D75.9, D79-D85, D87-D88, D89.8-D99, E07.8-E08.9, E17-E19, E34.0, E34.9-E35.8, E37-E39, E47-E49., E62, E69, E87.7, E90-E998, F04-F06.1, F06.5-F07.0, F07.8-F08, F50, F50.8-F50.9, G09-G09.9, G15-G19, G21, G21.2, G21.4-G22.0, G27-G29, G33-G34, G38-G39., G42, G48-G49, G66-G69, G74-G79, G84-G88, G93.8-G94, G96-G96.9, G98-G98.9, I00.0, I03-I04., I14-I14., I16-I19, I29-I29.9, I44-I45.9, I49-I49.9, I51, I51.6-I59, I90-I94, I96-I96.9, I98.4-I98.8, I99-ID5.9, J02.9, J03.9, J04.3, J06, J06.9, J40-J40.9, J47-J59, J71-J79, J81.9, J83, J85.9, J87-J89, J90.9, J93.6, J97-J98.0, J98.4-J99.8, K21-K21.9, K22.7, K31.9-K34, K39, K47-K49, K53-K54, K63-K63.4, K63.8-K63.9, K69, K70.4-K70.9, K78-K79, K84, K87-K89, K92, K92.9-K93, K96-K99, L06-L07, L09, L15-L19, L31-L39, L69, L77-L79, N09, N13-N13.5, N13.7-N13.9, N24, N28.8-N28.9, N38, N39.9-N40.9, N54-N59, N66-N69, N78-N79, N84, N84.2-N86, N88-N90.9, N92-N94.9, N95.0, O08-O08.9, O17-O19, O27, O37-O39, O49-O59, O78-O79, O93-O95.9, P06, P16-P18, P30-P34.2, P40-P49, P62-P69, P73, P79, P82, P85-P89, P96.9-P99.9, Q08-Q10.3, Q19, Q29-Q29., Q36.0-Q36.9, Q46-Q49, Q88, Q89.9, Q94, Q99.9-R01.2, R07, R07.1-R07.9, R31-R31.9	002, 031-031.9, 039-039.9, 070, 070.4-070.9, 085, 085.1-085.9, 088.0-088.7, 112-118.9, 130-130.9, 136.3-136.5, 149-149.9, 155.2, 159-159.9, 165-169, 176-179.9, 183.9-184, 184.5, 184.9, 187, 187.9, 189, 189.9, 190.9, 195-199.9, 209, 209.2-209.3, 209.6-210, 211, 211.9-212, 212.9, 214-216.9, 221, 221.9-222, 222.9-223, 223.9, 229, 229.1, 229.9-230.0, 230.9-231, 231.8-231.9, 233, 233.3, 233.6, 233.9-234, 234.9-235, 235.1-235.3, 235.5, 235.9-236, 236.3, 236.6, 236.9, 237.4, 239-239.1, 239.5, 239.7-239.9, 249-249.9, 259.2, 276.6, 278, 279-279.9, 293.0-293.9, 331.3-331.4, 332.1-332.9, 347-348.9, 349.9, 357, 357.8-357.9, 399-400.0, 406-409.4, 418-419.9, 426-427, 427.4, 429, 429.2-429.9, 459.5-459.9, 464.5, 465, 465.9, 505-505.9, 519, 519.8-519.9, 530.1, 530.7-530.9, 544-549, 553.8-553.9, 559-559.0, 560.4-560.7, 561, 562.2-563, 569, 569.8-569.9, 591-591.9, 593.9, 599.9-600.9, 623.7, 624.6, 637-637.9, 639-639.9, 749.1, 759, 759.9, 779.9, 782.5, 785-785.3, 786.0-786.2, 786.4-786.5, 786.7, 786.9, 788.1-788.2, E986-E987
Garbage Code (GBD Level 4)	B16.9, B64, B82-B82.9, B83.9, C69, C69.9, C91.1, C91.4-C91.5, C91.7-C91.9, C92.7-C92.9, C93.2, C93.5-C93.7, C93.9, E12-E14.9, G00, G00.9-G02.8, G03.9, I37.9, I42-I42.0, I42.9, I51.5, I64-I64.9, I67, I67.8-I68, I68.8-I69, I69.4-I69.9, J07-J08, J15.9, J17-J19.6, J22-J29, J64-J64.9, P23, P23.5-P23.9, P37.3-P37.4, R73-R73.9, V87-V87.1, V87.4-V88.1, V88.4-V89.9, V99-V99.0, X84-X84.9, Y09-Y09.9, Y85-Y85.9	070.3, 084, 084.6, 194-194.0, 194.9, 204.1, 204.5-204.9, 205.8-205.9, 206.2-206.9, 238, 244, 244.9, 250-250.9, 289.8-289.9, 307.5, 320, 320.9, 357.2, 362.0, 425, 425.4, 425.9, 429.1, 436-437, 437.9-439.6, 482.9-483, 484, 484.8-486.9, 770.0, 790.2, E808-E829

Table S3. List of infectious syndromes mapped to International Classification of Diseases (ICD) codes — **Infectious Syndrome to ICD Codes (ICD9)**

#	Level	L0	L1	L2	L3	ICD Codes for all level	L0	L1	L2	L3	ICD Codes for all level (ICD9)
1	0	explicit sepsis				A02.1, A20.7, A21.7, A22.7, A26.7, A28.2, A32.7, A40-A41.9, A42.7, A48.8, A50-A50.0, B37.7, I76, O03.0, O03.5, O04.5, O08.0, O23-O23.9, O41.1, O75.3, O85-O86.8, O88.3, O91-O91.2, O98, P36-P37, R65.2	explicit sepsis				038-038.9, 670-670.9, 771.4-771.9, 995.9
2	0	implicit sepsis				A00-A02.0, A02.2-A20.3, A20.8-A21.3, A21.8-A22.2, A22.8-A26.0, A26.8-A28.1, A28.8-A32.1, A32.8-A39.9, A42-A42.2, A42.8-A48.5, A49-A49.9, A50.1-B37.6, B37.8-B99.9, D65-D65.9, D69.5, D70.3, D89.3, E87.2-E87.9, F02.1, F02.4, F07.1, G00-G08.0, G14-G14.6, G21.3, G93.4, H05.0-H05.1, H60.2, H62-H62.3, H65-H68.0, H70-H70.9, H73.0, H75-H75.0, I00, I01-I02.9, I30, I30.1, I33-I33.9, I38-I41.9, I46-I46.9, I80-I82.9, I87.0, I89.1, I95.1-I96.9, I98.0-I98.1, J00-J06.9, J09-J22.9, J31-J32.9, J35-J37.1, J39.0-J39.1, J40-J42.6, J80-J80.9, J85-J85.3, J86-J86.9, J91.0, J95.2-J95.3, J96-J96.9, K04-K05.5, K35-K37.9, K38.3-K38.9, K40-K42.9, K44-K46.9, K50-K52, K52.1-K52.9, K55-K57.9, K61-K61.4, K63.0-K63.1, K65-K65.9, K67-K69, K72-K72.9, K75.0-K75.1, K75.3, K76.3, K77.0, K80-K83.9, K93.0-K93.1, L00-L08.9, L30.3, L88-L89.9, L97-L98.4, M00-M01.8, M09.1, M46.2-M46.5, M49.0-M49.1, M65.0-M65.1, M71.0-M71.1, M72.5-M72.6, M73.0-M73.8, M86-M86.9, M89.6, N00-N01.9, N10-N12.9, N13.6, N15, N15.1-N17.9, N30-N30.9, N34-N34.3, N39.0-N39.2, N41-N41.9, N45-N45.9, N49-N49.9, N70-N77.8, N98.0, O04.0, O05.0, O05.5, O06.0, O06.5, O07.0, O07.5, O98.0-O98.9, P00.2, P22-P23.9, P35-P35.9, P37.0-P39.9, R02-R02.9, R09.2, R19.7, R40.0-R40.4, R55-R55.0, R57-R57.9, R65.0, R83.5, R84.5, R85.5, R86.5, R87.5, T80.2, T81.4, T82.6-T82.7, T83.5-T83.6, T84.5-T84.7, T85.7, T87.4, T88.0, U04.9, U06-U07.2, U82-U89.9, Y62-Y62.9, Y95, Z03.0, Z11.1, Z16-Z16.3, Z21-Z22.9	implicit sepsis				001-018.9, 020-037.9, 039-104.9, 110-139.9, 275.8-276.9, 286.6, 293-294, 320-326.9, 347-347.9, 376.0-376.1, 381-383.9, 390-392.9, 420-424.9, 425.6, 427.5, 451-453.9, 457-458.9, 460-469, 470.0, 472-476.9, 480-491.9, 510-511.9, 513-513.9, 519.1-519.9, 522-523.9, 540-542.9, 550-551.1, 551.3-552.1, 552.3-553.1, 553.3-558.0, 558.2-558.9, 560-560.3, 560.8-560.9, 562-562.1, 567-567.9, 569.5, 572.0-572.1, 574-576.9, 584-584.9, 590-590.9, 595-595.9, 597-597.9, 599.0, 601-601.9, 604-604.9, 614-616.9, 634.0, 635-635.0, 635.5, 636.0, 636.5, 637-637.0, 637.5, 638.0, 639.0, 647-647.9, 658.4, 659.3, 669.1, 671.2-671.5, 672-672.0, 675-675.9, 680-689, 707-707.9, 711-711.9, 730-730.9, 768.9-769, 770.0, 771-771.3, 780-780.3, 785.4-785.5, 790.7, 798-799.2, 996.6
4	1		Diarrhoea			A00-A00.9, A02-A02.0, A02.8-A09.9, K52.1-K52.3, R19.7		Diarrhoea			001-001.9, 003.8-009.9, 558.2-558.9
5	2			Diarrhoea due to specific bacteria		A00-A00.9, A02-A02.0, A02.8-A03.9, A04.0-A04.7			Diarrhoea due to specific bacteria		001-001.9, 003.8-004.9, 007.4-007.7, 008.0-008.3
6	3				Diarrhoea due to vibrio cholerae	A00-A00.9				Diarrhoea due to vibrio cholerae	001-001.9
7	3				Diarrhoea due to salmonella enterica	A02-A02.0, A02.8-A02.9				Diarrhoea due to salmonella enterica	003.8-003.9
8	3				Diarrhoea due to shigella spp	A03-A03.9				Diarrhoea due to shigella spp	004-004.9
9	3									Diarrhoea due to enteropathogenic escherichia coli	008.0
10	3				Diarrhoea due to enteropathogenic escherichia coli	A04.0					
11	3				Diarrhoea due to Enterotoxigenic escherichia coli	A04.1-A04.4					
12	3				Diarrhoea due to campylobacter	A04.5					
13	3				Diarrhoea due to yersinia enterocolitica	A04.6					
14	3				Diarrhoea due to clostridium difficile	A04.7					
15	3									Diarrhoea due to proteus spp	008.3
16	3									Diarrhoea due to paracolon bacilli	008.1
17	3									Diarrhoea due to aerobacter aerogenes	008.2
18	2			Diarrhoea due to unspecified bacteria		A04, A04.8-A05, A05.8-A05.9, K52.1-K52.3, R19.7					
19	2			Diarrhoea due to food borne pathogen or intoxication		A05.0-A05.5			Diarrhoea due to food borne pathogen or intoxication		005.0-005.9
20	3				Diarrhoea due to food borne illness staphylococcus aureus	A05.0				Diarrhoea due to food borne illness staphylococcus aureus	005.0, 005.8-005.9
21	3				Diarrhoea due to food borne illness clostridium botulinum	A05.1				Diarrhoea due to food borne illness clostridium botulinum	005.1
22	3				Diarrhoea due to food borne illness clostridium perfringens	A05.2				Diarrhoea due to food borne illness clostridium perfringens	005.2-005.3
23	3				Diarrhoea due to food borne illness parahaemolyticus	A05.3				Diarrhoea due to food borne illness parahaemolyticus	005.4
24	3				Diarrhoea due to food borne illness bacillus cereus	A05.4					
25	3				Diarrhoea due to food borne illness vibrio vulnificus	A05.5					
26	2			Diarrhoea due to protozoal		A06-A07.9			Diarrhoea due to protozoal		006-006.9, 007.1
27	3				Diarrhoea due to amebiasis	A06-A06.9				Diarrhoea due to amebiasis	006-006.9
28	3									Diarrhoea due to giardiasis	007.1
29	3				Diarrhoea due to cryptosporidiosis	A07.2				Diarrhoea due to cryptosporidiosis	007.4, 007.7
30	3				Diarrhoea due to isosporiasis	A07.3					
31	3				Diarrhoea due to cyclosporiasis	A07.4				Diarrhoea due to cyclosporiasis	007.5
32	2			Diarrhoea due to various		A08-A08.9					
33	3				Diarrhoea due to rotavirus	A08.0					
34	3				Diarrhoea due to norovirus	A08.1					
35	3				Diarrhoea due to adenovirus	A08.2					
36	3				Diarrhoea due to viral other	A08.3-A08.9					
37	2			Diarrhoea to unspecified Pathogen		A09-A09.9			Diarrhoea to unspecified Pathogen		005, 007-007.0, 007.2-007.3, 007.8-008, 008.4-009.9, 558.2-558.9
38	1		Typhoid, paratyphoid and Invasive Non-typhoidal Salmonella (iNTS)			A01-A01.4, A02.2, A02.1		Typhoid, paratyphoid and Invasive Non-typhoidal Salmonella (iNTS)			002-003.7
39	2			Typhoid or paratyphoid unspecified		A01			Typhoid or paratyphoid unspecified		002, 002.9
40	2			Typhoid fever		A01.0			Typhoid fever		002.0
41	3				typhoid fever salmonella typhi	A01.0				typhoid fever salmonella typhi	002.0
42	2			Paratyphoid fever		A01.1-A01.4			Paratyphoid fever		002.1-002.4
43	3				paratyphoid fever salmonella paratyphi	A01.1-A01.4				paratyphoid fever salmonella paratyphi	002.1-002.4
44	2			Invasive Non-typhoidal Salmonella (iNTS)		A02.2, A02.1			Invasive Non-typhoidal Salmonella (iNTS)		003-003.7
45	3				Invasive Non-typhoidal Salmonella (iNTS)	A02.2, A02.1				Invasive Non-typhoidal Salmonella (iNTS)	003.2-003.7
46	1		Tuberculosis			A10-A19.9, K67.3, K93.0, M49.0, N74.1-N74.2, O98.0, P37.0, U84.3		Tuberculosis			010-018.9, 320.4, 647.3, 730.4-730.6
47	2			Respiratory tuberculosis		A10-A16.9			Respiratory tuberculosis		010-013
48	2			Tuberculosis of nervous system		A17-A17.9			Tuberculosis of nervous system		013.0-013.9, 320.4
49	2			Tuberculosis of intestines, peritoneum and mesenteric glands		K67.3, K93.0			Tuberculosis of intestines, peritoneum and mesenteric glands		014-014.9
50	2			Tuberculosis of other organs		A18-A18.9, M49.0, P37.0, U84.3			Tuberculosis of other organs		015-015.9, 017-017.9, 647.3, 730.4-730.6
51	2			Tuberculosis of genitourinary system		N74.1-N74.2, O98.0			Tuberculosis of genitourinary system		016-016.9
52	2			Milliary Tuberculosis		A19-A19.9			Milliary Tuberculosis		018-018.9
53	1		Bloodstream infections			A20-A20.3, A20.8-A21.3, A21.8-A22.2, A22.8-A26.0, A26.8-A28.1, A28.8-A28.9, A30-A32.0, A32.8-A35.0, A38-A38.9, A39.1-A39.9, A20.7, A21.7, A22.7, A26.7, A28.2, A32.7, A40-A41.9, A48.8, A50-A50.0, I76, O03.5, O04.5, O08.0, O23, O23.9, O85-O86.8, O88.3, O91-O91.2, P36-P36.9, R65.2, A44-A45.9, A47-A48, A48.3-A48.5, A49-A49.9, A50.1-A53.9, A65-A65.0, A68-A69.9, A72-A79.9, B20.1, B94.9-B96.8, B98-B99.9, I98.0-I98.1, K04-K05.5, M49.1, O04.0, O05.0, O05.5, O06.0, O06.5, O07.0, O07.5, O98.1, P37.2, P37.8-P39.9, T80.2, T83.5-T83.6, T85.7, T87.4, T88.0, U82-U84, U85-U89.9, Y62-Y62.9, Y95, Z03.0, Z11.1, Z16-Z16.3, Z22-Z22.4, Z22.8-Z22.9		Bloodstream infections			020-031.9, 034, 034.1-034.9, 038-038.9, 670-670.9, 771.4-771.9, 995.9, 036.2-037.9, 040-041.9, 076.0-076.9, 078.3, 080-083.9, 087-088.8, 090-097.9, 100-101.6, 634.0, 635-635.0, 636.0, 637-637.0, 638.0, 639.0, 647, 647.8-647.9, 658.4, 659.3, 671.2-671.5, 672-672.0, 675-675.9, 771-771.3, 790.7, 996.6, E872-E872.9
54	2			Bloodstream infections due to specified bacteria		A20.7, A21.7, A22.7, A26.7, A32.7, A40-A40.9, A41.0-A41.5, A50-A50.0			Bloodstream infections due to specified bacteria		038.0-038.4
55	2			Bloodstream infections due to specified bacteria		A20-A20.3, A20.8-A21.3, A21.8-A22.2, A22.8-A25, A26-A26.0, A26.8-A27.9, A30-A32.0, A32.8-A35.0, A38-A38.9, A39.1-A39.9, A44-A44.9, A48.5, A49.0-A49.3, A50.1-A53.9, A65-A65.0, A68-A69.9, A72-A79.9, B95.0-B95.8, B96.0-B96.7, I98.0, M49.1, O98.1			Bloodstream infections due to specified bacteria		020-031.9, 034, 034.1-034.9, 036.2-037.9, 041-041.2, 041.4-041.8, 076.0-076.9, 080-083.9, 087-088.8, 090-097.9, 100-101.6
56	3				Bloodstream infections due to bacillus-anthracis	A22.7					
57	3				Bloodstream infections due to yersinia pestis	A20-A20.3, A20.8-A20.9, A20.7				Bloodstream infections due to yersinia pestis	020-020.8
58	3				Bloodstream infections due to francisella tularensis	A21-A21.3, A21.8, A21.7				Bloodstream infections due to francisella tularensis	020.9-022
59	3				Bloodstream infections due to erysipelothrix rhusiopathiae	A26.7					
60	3				Bloodstream infections due to anthracis	A21.9-A22.2, A22.8-A22.9				Bloodstream infections due to anthracis	022.0-022.6
61	3				Bloodstream infections due to brucella	A23-A23.9, M49.1				Bloodstream infections due to brucella	022.8-023.9
62	3				Bloodstream infections due to burkholderia pseudomallei	A24-A25				Bloodstream infections due to burkholderia pseudomallei	024-025.9
63	3				Bloodstream infections due to erysipelothrix rhusiopathiae	A26-A26.0, A26.8-A26.9				Bloodstream infections due to erysipelothrix rhusiopathiae	027.1
64	3				Bloodstream infections due to listeria	A27-A27.9, A32-A32.0, A32.8-A32.9, A32.7				Bloodstream infections due to listeria	027.0
65	3				Bloodstream infections due to anaerobes	A41.4				Bloodstream infections due to anaerobes	038.3
66	3				Bloodstream infections due to non tuberculous mycobacteria	A30-A31.9				Bloodstream infections due to non tuberculous mycobacteria	031.0-031.2
67	3				Bloodstream infections due to clostridium tetani	A33-A35.0				Bloodstream infections due to clostridium tetani	037-037.9
68	3				Bloodstream infections due to group a strep	A38-A38.9, B95.0, A40-A40.0				Bloodstream infections due to group a strep	034, 034.1-034.9
69	3				Bloodstream infections due to neisseria meningitidis	A39.1-A39.9				Bloodstream infections due to neisseria meningitidis	036.2-036.9
70	3				Bloodstream infections due to bartonella	A44-A44.9					
71	3				Bloodstream infections due to clostridium botulinum	A48.5					
72	3				Bloodstream infections due to staphylococcus aureus	A49.0, B95.6-B95.8, A41.0-A41.2				Bloodstream infections due to staphylococcus aureus	041.1, 038.1
73	3				Bloodstream infections due to group b strep	A49.1, B95.1, A40.1-A40.3					
74	3				Bloodstream infections due to haemophilus influenzae	A49.2, B96.3, A41.3				Bloodstream infections due to haemophilus influenzae	041.5
75	3				Bloodstream infections due to mycoplasma	A49.3, B96.0-B96.1					
76	3				Bloodstream infections due to treponema pallidum	A50.1-A53.9, A65-A65.0, I98.0, O98.1, A50-A50.0				Bloodstream infections due to treponema pallidum	090-097.9, 100
77	3				Bloodstream infections due to rickettsias spp	A68-A69.9, A75-A79.9					
78	3				Bloodstream infections due to chlamydia spp	A72-A74.9				Bloodstream infections due to chlamydia spp	076.0-076.9
79	3				Bloodstream infections due to enterococcus spp	B95.2					
80	3				Bloodstream infections due to streptococcus pneumoniae	B95.3				Bloodstream infections due to streptococcus pneumoniae	041.2, 038.2
81	3				Bloodstream infections due to unspecified streptococcus	B95.4-B95.5, A40.8-A40.9				Bloodstream infections due to unspecified streptococcus	041.0
82	3				Bloodstream infections due to due to klebsiella spp	B96.2					
83	3				Bloodstream infections due to proteus spp	B96.4				Bloodstream infections due to proteus spp	041.6
84	3				Bloodstream infections due to Pseudomonas aeruginosa	B96.5					
85	3				Bloodstream infections due to bacteroides fragilis	B96.6				Bloodstream infections due to escherichia coli	041.4
86	3				Bloodstream infections due to clostridium perfringens	B96.7					
87	3									Bloodstream infections due to pseudomonas spp	041.7
88	3									Bloodstream infections due to borrelia recurrentis	080-083.9, 087-088, 088.8, 100.0-101.6
89	3									Bloodstream infections due to non typhoidal salmonellae	003.1
90	3				Bloodstream infections due to gram negative other	A41.5				Bloodstream infections due to gram negative other	038.4
91	2			Bloodstream infections due to other specified bacteria		A25.0-A25.9, A28-A28.1, A28.8-A28.9, A28.2			Bloodstream infections due to other specified bacteria		078.3
92	2			Bloodstream infections due to unspecified bacteria		A45-A45.9, A47-A48, A49, A49.8-A49.9, B20.1, B94.9-B95, B96, B96.8, B98-B99.9, I98.1, K04-K05.5, O04.0, O05.0, O05.5, O06.0, O06.5, O07.0, O07.5, T80.2, T83.5-T83.6, T85.7, T87.4, T88.0, U82-U84, U85-U89.9, Y62-Y62.9, Y95, Z03.0, Z11.1, Z16-Z16.3, Z22-Z22.4, Z22.8-Z22.9, A41.6-A41.9, A48.8, I76, O03.5, O04.5, O08.0, O23, O23.9, O85-O86.8, O88.3, O91-O91.2, R65.2			Bloodstream infections due to unspecified bacteria		634.0, 635-635.0, 636.0, 637-637.0, 638.0, 639.0, 647, 647.8-647.9, 658.4, 659.3, 671.2-671.5, 672-672.0, 675-675.9, 670-670.9, E872-E872.9
93	2			Bloodstream infections unspecified pathogen		A41			Bloodstream infections unspecified pathogen		040-040.8, 041.3, 041.9, 790.7, 996.6, 038, 038.5-038.9
94	3									Bloodstream infections due to staph aureus with toxin production	040.9
95	2			Bloodstream infections due to group a strep with toxin production		A48.3-A48.4					
96	3				Bloodstream infections due to unspecified bacteria with toxin production	A48.3-A48.4					
97	2			Neonatal bloodstream infections due to specified bacteria		P36-P36.9, P37.2, P37.8-P39.9			Neonatal bloodstream infections due to specified bacteria		771.3
98	3				Neonatal bloodstream infections due to group a strep	P36.0, P36.1					
99	3				Neonatal bloodstream infections due to staphylococcus aureus	P36.2-P36.3					
100	3				Neonatal bloodstream infections due to escherichia coli	P36.4					

	Level	L0	L1	L2	L3	ICD Codes for all level	L0	L1	L2	L3	ICD Codes for all level
**Table S3. List of infectious syndromes mapped to International Classification of Diseases (ICD) codes**							**Infectious Syndrome to ICD Codes (ICD9)**				
	**Infectious syndrome name and level**					**Infectious syndrome name and ICD code**	**Infectious syndrome name and level**				**Infectious syndrome name and ICD code**
101	3				Neonatal bloodstream infections due to anaerobes	P36.5					
102	3				Neonatal bloodstream infections due to listeria	P37.2					
103	3				Neonatal bloodstream infections due to unspecified bacteria	P36				Neonatal bloodstream infections due to unspecified bacteria	771-771.2, 771.4-771.9
104	1		Other and non bacterial infectious			A29, A36-A36.9, A42-A42.2, A42.8-A43.9, A42.7, B37.7, P37, A54.3, A54.5-A54.6, A59-A63.8, A71-A71.9, A80-A86.4, A88-B20.0, B20.2-B34.1, B34.3-B37.6, B37.8-B94.8, B97-B97.1, B97.3, B97.7-B97.8, F07.1, G04-G05.8, G14-G14.6, G21.3, H05.1, H60.2, H62-H62.3, H65-H68.0, H70-H70.9, H73.0, H75-H75.0, I02.9, J00-J06.9, J31-J32.9, J35-J37.1, J39.0-J39.1, J85-J85.3, J86-J86.9, J91.0, K93.1, M89.6, N72-N72.0, N75-N77.8, O98.4-O98.7, P35-P35.9, P37.1, P37.3-P37.5, T81.4, T82.6-T82.7, T84.5-T84.7, U06-U06.9, Z21-Z21.0, Z22.5-Z22.6		Upper respiratory infections and otitis due to corynebacterium diphtheriae			032-032.9, 034.0, 042-046.9, 050-072.9, 074-076, 077-078.2, 078.4-079.5, 079.8-079.9, 084-086.9, 088.9-089.9, 098.4, 098.6-098.7, 099, 099.4-099.9, 110-134.9, 136-139.9, 323-323.9, 381-383.9, 425.6, 460-465.9, 472-476.9, 522-523.9, 616-616.9, 647.4-647.6
105	2			Non bacterial protozoal infections		A59-A59.9, B50-B64, P37.1, P37.3-P37.4			Non bacterial protozoal infections		084-086.9, 089-089.9, 647.4
106	2			Non bacterial protozoal infections		P37					
107	2			Non bacterial fungal infections		B65-B94, B94.2-B94.8, H05.1, K93.1, U06-U06.9			Non bacterial fungal infections		088.9, 120-134.9, 136-137.9, 138.0-139, 139.1-139.9, 425.6
108	2			non bacterial fungal infections		B35-B36.9, P37.5			non bacterial fungal infections		110-119
109	2			non bacterial viral infections		A60-A63.8, B01-B20.0, B20.2-B34.1, B34.3-B34.9, B94.1, B97-B97.1, B97.3, B97.7-B97.8, J91.0, M89.6, O98.4-O98.7, P35-P35.9, Z21-Z21.0, Z22.5-Z22.6			non bacterial viral infections		042-046.9, 050-061.8, 070-072.9, 138, 647.5-647.6
110	2			Other opportunistic infatious		A29, A42-A42.2, A42.8-A43.9, B37-B37.6, B37.8-B49.9, T81.4, T82.6-T82.7, T84.5-T84.7, A42.7, B37.7					
112	2			Viral encephalitis		A80-A86.4, A88-B00.9, F07.1, G04-G05.8, G14-G14.6, G21.3			Viral encephalitis		062-069, 139.0, 323-323.9
113	2			Other locl infectious		A54.3, A54.5-A54.6, A71-A71.9, B94.0, N72-N72.0, N75-N77.8			Other locl infectious		076, 098.4, 098.6-098.7, 099, 099.4-099.9, 522-523.9, 616-616.9
114	2			Upper respiratory infections and otitis due to specified bacteria		A36-A36.9, I02.9, J02.0, J03.0			Upper respiratory infections and otitis due to specified bacteria		034.0
115	3				Upper respiratory infections and otitis due to corynebacterium diphtheriae	A36-A36.9				Upper respiratory infections and otitis due to corynebacterium diphtheriae	032-032.9
116	3				Upper respiratory infections and otitis due to group a strep	I02.9, J02.0, J03.0					
117	2			Upper respiratory infections and otitis due to unspecified bacteria		H60.2, H62-H62.3, H65-H68.0, H70-H70.9, H73.0, H75-H75.0			Upper respiratory infections and otitis due to unspecified bacteria		381-383.9
118	2			Upper respiratory infections and otitis due to unspecified bacteria		J36-J36.0, J39.0-J39.1, J85-J85.3, J86-J86.9			Upper respiratory infections and otitis due to unspecified bacteria		464.5, 465, 465.9, 472-474.9, 476-476.1
119	2									Upper respiratory infections and otitis due to Virus	074-075.9, 077-077.9, 078.8, 079.8-079.9
120	2			Upper respiratory infections and otitis due to unspecified pathogen		J00-J02, J02.8-J03, J03.8-J06.9, J31-J32.9, J35-J35.9, J37-J37.1			Upper respiratory infections and otitis due to unspecified pathogen		460-464.4, 464.8-464.9, 465.0-465.8, 475-475.9, 476.9
121	2									Urogenital infections due to virus	078-078.2, 078.4-078.7, 078.9-079.5
122	1		Meningitis and other bacterial central nervous system abases			A32.1, A39-A39.0, A87-A87.9, G00-G03.9, G06-G08.0, R83.5		Meningitis and other bacterial central nervous system abases			036-036.1, 047-049.9, 320-320.3, 320.5-322.9, 324-326.9
123	2			Meningitis due to specific bacteria		A32.1, A39-A39.0, G00.0-G00.3			Meningitis due to specific bacteria		036-036.1, 320.0-320.3, 320.5-320.8
124	3				Meningitis due to listeria	A32.1					
125	3				Meningitis due to neisseria meningitidis	A39-A39.0				Meningitis due to neisseria meningitidis	036-036.1, 320.5-320.8
126	3				Meningitis due to haemophilus	G00.0				Meningitis due to haemophilus	320.0
127	3				Meningitis due to streptococcus pneumoniae	G00.1				Meningitis due to streptococcus pneumoniae	320.1
128	3				Meningitis due to group b strep	G00.2				Meningitis due to group b strep	320.2
129	3				Meningitis due to staphylococcus aureus	G00.3				Meningitis due to staphylococcus aureus	320.3
130	2			Meningitis due to virus		A87-A87.9, G03.0			Meningitis due to virus		047-049.9
131	2			Meningitis due to unspecified pathogen		G00, G00.8, G01-G02.8, G03.1-G03.9, G06-G08.0, R83.5			Meningitis due to unspecified pathogen		320, 320.9-321.8, 322.0-322.9, 324-326.9
132	2			Meningitis due to unspecific bacteria		G00.9, G03			Meningitis due to unspecific bacteria		322
133	1		Respiratory infectious			A37-A37.9, A48.1-A48.2, A70, B34.2, B97.2, B97.4-B97.6, J09-J22.9, J40-J42.6, P00.2, P22-P23.9, R84.5, U04.9, U07-U07.2		Respiratory infectious			033-033.9, 039.1, 073-073.9, 079.6-079.7, 466-469, 470.0, 480-491.9, 510-511.9, 513-513.9, 519.2, 519.8-519.9, 770.0
134	2			Lower respiratory infections due to specific bacteria		A37-A37.9, A48.1-A48.2, A70, J13-J14.0, J15.0-J15.7, J16.0, J20.0-J20.2, P23.1-P23.5			Lower respiratory infections due to specific bacteria		033-033.9, 039.1, 073-073.9, 466-469, 470.0, 481-481.9, 482.0-482.4, 483.0-483.1, 484.2-484.5
135	3				Lower respiratory infections due to bordetella pertussis	A37-A37.9				Lower respiratory infections due to bordetella pertussis	033-033.9, 484.3
136	3				Lower respiratory infections due to legionella spp	A48.1-A48.2					
137	3									Lower respiratory infections due to actinomyces	039.1
138	3				Lower respiratory infections due to chlamydia spp	A70, J16.0, P23.1				Lower respiratory infections due to chlamydia spp	073-073.9, 483.1, 484.2
139	3				Lower respiratory infections due to streptococcus pneumoniae	J13-J13.9, J15.4, J20.2				Lower respiratory infections due to streptococcus pneumoniae	481-481.9, 482.3
140	3				Lower respiratory infections due to haemophilus influenzae	J14-J14.0, J20.1				Lower respiratory infections due to haemophilus influenzae	482.2
141	3				Lower respiratory infections due to klebsiella pneumoniae	J15.0				Lower respiratory infections due to klebsiella pneumoniae	482.0
142	3									Lower respiratory infections due to pseudomonas spp	482.1
143	3				Lower respiratory infections due to Pseudomonas aeruginosa	J15.1, P23.5					
144	3				Lower respiratory infections due to staphylococcus aureus	J15.2, P23.2				Lower respiratory infections due to staphylococcus aureus	482.4
145	3				Lower respiratory infections due to group b strep	J15.3, P23.3					
146	3				Lower respiratory infections due to escherichia coli	J15.5, P23.4					
147	3				Lower respiratory infections due to mycoplasma	J15.7, J20.0				Lower respiratory infections due to mycoplasma	483.0
148	3									Lower respiratory infections due to francisella-tularensis	484.4
149	3									Lower respiratory infections due to bacillus-anthracis	484.5
150	2			Lower respiratory infections due to unspecified Bacteria		J15, J15.8-J16, J16.8-J17, J17.1, J18-J20, J20.9-J21, J21.8-J22.9, J40-J42.6, P23.6-P23.9, R84.5			Lower respiratory infections due to unspecified Bacteria		482, 482.8-483, 483.8-484, 484.8-486.9, 490-491.9
151	3				Lower respiratory infections due to other gram negative bacteria	J15.6					
152	2			Lower respiratory infections due to virus		B34.2, B97.2, B97.4-B97.6, J09-J12.9, J17.0, J17.2-J17.8, J20.3-J20.8, J21.0-J21.1, P23.0, U04.9, U07-U07.2			Lower respiratory infections due to virus		079.6-079.7, 480-480.9, 484.0-484.1, 487-489
153	3				Lower respiratory infections due to coronaviruses	B34.2, B97.2, J12.8					
154	3				Lower respiratory infections due to syncytial virus (rsv)	B97.4, J12.1, J20.5, J21.0					
155	3				Lower respiratory infections due to influenza viruses	J09-J11.8					
156	3				Lower respiratory infections due to other virus	J12, J12.3, J12.9, J17.0, J17.2-J17.8, J20.3, J20.7-J20.8, J21.1					
157	3				Lower respiratory infections due to adenoviruses	J12.0					
158	3				Lower respiratory infections due to parainfluenza viruses	J12.2, J20.4					
159	3				Lower respiratory infections due to rhinoviruses	J20.6					
160	3									Lower respiratory infections due to fungi	484.6-484.7
161	3									Lung abscess and pyothorax due to unspecified pathogen	510-511.9, 513-513.9
162	2			Lower respiratory infections due to unspecified pathogen		P00.2, P22-P23			Lower respiratory infections due to unspecified pathogen		519.2, 519.8-519.9, 770.0
163	1		Skin Bacterial Infectious			A46-A46.0, A48.0, A66-A67.9, H05.0, I89.1, I96-I96.9, K61-K61.4, L00-L08.9, L30.3, L88-L89.9, L97-L98.1, L98.4, M72.5-M72.6, R02-R02.9		Skin Bacterial Infectious			035-035.9, 039-039.0, 039.3-039.9, 102-104.9, 376.0-376.1, 457.1-457.9, 680-689, 707-707.9, 785.4
164	2			Bacterial infections of skin and subcutaneous system due to specific bacteria		A46-A46.0, A48.0, A66-A67.9, L00			Bacterial infections of skin and subcutaneous system due to specific bacteria		035-035.9, 039-039.0, 039.3-039.9, 102-104.9
165	3				Bacterial infections of skin and subcutaneous system due to group a strep	A46-A46.0				Bacterial infections of skin and subcutaneous system due to group a strep	035-035.9
166	3				Bacterial infections of skin and subcutaneous system due to clostridium perfringens	A48.0					
167	3				Bacterial infections of skin and subcutaneous system due to treponema pallidum	A66-A66.9				Bacterial infections of skin and subcutaneous system due to treponema pallidum	102-102.9, 104-104.9
168	3				Bacterial infections of skin and subcutaneous system due to treponema carateum	A67-A67.9					
169	3				Bacterial infections of skin and subcutaneous system due to staphylococcus aureus	L00					
170	3									Bacterial infections of skin and subcutaneous system due to yersinia pestis	103-103.9
171	3									Bacterial infections of skin and subcutaneous system due to other specific bacteria	039.0
172	2			Bacterial infections of skin and subcutaneous system due to unspecified bacteria		H05.0, I89.1, I96-I96.9, K61-K61.4, L01-L08.9, L30.3, L88-L89.9, L97-L98.1, L98.4, M72.5-M72.6, R02-R02.9			Bacterial infections of skin and subcutaneous system due to unspecified bacteria		376.0-376.1, 457.1-457.9, 680-689, 707-707.9, 785.4
173	1				chlamydia and gonorrheae	A54-A54.2, A54.8-A56.8		chlamydia and gonorrheae			098-098.3, 098.8-098.9, 099.1, 099.3
174	1		infections of bone, joints and related part			A54.4, M00-M01.8, M46.2-M46.5, M65.0-M65.1, M71.0-M71.1, M73.0-M73.8, M86-M86.9		infections of bone, joints and related part			098.5, 711-711.9, 730-730.3, 730.7-730.9
175	2			infections of bone, joints and related part due to specific bacteria		A54.4, M00-M00.2			infections of bone, joints and related part due to specific bacteria		098.5
176	3				infections of bone, joints and related part due to neisseria gonorrheae	A54.4				infections of bone, joints and related part due to neisseria gonorrheae	098.5
177	3				infections of bone, joints and related part due to staphylococcus aureus	M00-M00.0					
178	3				infections of bone, joints and related part due to streptococcus pneumoniae	M00.1					
179	3				infections of bone, joints and related part due to group a strep	M00.2					
180	2			infections of bone, joints and related part due to unspecified bacteria		M00.8-M01.8, M46.2-M46.5, M65.0-M65.1, M71.0-M71.1, M73.0-M73.8, M86-M86.9			infections of bone, joints and related part due to unspecified bacteria		711-711.9, 730-730.3, 730.7-730.9
181	1		Urinary tract infections and nephritis			A57-A58, A64-A64.0, N10-N12.9, N13.6, N15, N15.1-N16.8, N30-N30.9, N34-N34.3, N39.0-N39.2, N41-N41.9, N45-N45.9, N49-N49.9, O23.0-O23.5		Urinary tract infections and nephritis			099.0, 099.2, 590-590.9, 595-595.9, 597-597.9, 599.0, 601-601.9, 604-604.9, 647.2
182	2			Urinary tract infections and nephritis due to specific bacteria		A57-A58			Urinary tract infections and nephritis due to specific bacteria		099.0, 099.2
183	3				Urinary tract infections and nephritis due to haemophilus ducreyi	A57-A57.0				Urinary tract infections and nephritis due to haemophilus ducreyi	099.0
184	3				Urinary tract infections and nephritis due to klebsiella granulomatis	A58				Urinary tract infections and nephritis due to klebsiella granulomatis	099.2
185	2			Urinary tract infections and nephritis due to unspecified bacteria		A64-A64.0, N10-N12.9, N13.6, N15, N15.1-N16.8, N30-N30.9, N34-N34.3, N39.0-N39.2, N41-N41.9, N45-N45.9, N49-N49.9, O23.0-O23.5			Urinary tract infections and nephritis due to unspecified bacteria		590-590.9, 595-595.9, 597-597.9, 599.0, 601-601.9, 604-604.9, 647.2
186	1		Endocarditis and other cardiac infections			I00, I01-I02.0, I30, I30.1, I33-I33.9, I38-I41.9		Endocarditis and other cardiac infections			390-392.9, 420-424.9, 457
187	2			Endocarditis due to specific bacteria		I00, I01-I02.0			Endocarditis due to specific bacteria		390-392.9, 421-421.9
188	3				Endocarditis due to group a strep	I00, I01-I02.0				Endocarditis due to group a strep	390-392.9
189	2			Endocarditis due to unspecific bacteria		I30, I30.1, I33-I33.9, I38-I41.9			Endocarditis due to unspecific bacteria		420-420.9, 422-424.9, 457
190	1		Peritoneal and intra abdomen infections			K35-K37.9, K38.3-K38.9, K40-K42.9, K44-K46.9, K50-K52, K52.8-K52.9, K55-K57.9, K63.0-K63.1, K65-K65.9, K67-K67.2, K67.8-K69, K75.0-K75.1, K75.3, K76.3, K77.0, K80-K83.9, M09.1, N70-N71.9, N73-N74.0, N74.3-N74.8, N98.0, O98.2-O98.3, O98.8-O98.9, R65.0, R85.5, R86.5, R87.5; O03.0, O41.1, O75.3, O98		Peritoneal and intra abdomen infections			039.2, 540-542.9, 550-551.1, 551.3-552.1, 552.3-553.1, 553.3-558.0, 560-560.3, 560.8-560.9, 562-562.1, 567-567.9, 569.5, 572.0-572.1, 574-576.9, 614-615.9, 647.0-647.1
191	2			Peritoneal and intra abdomen infections due to specific bacteria		K67.0-K67.2			Peritoneal and intra abdomen infections due to specific bacteria		039.2
192	3				Peritoneal and intra abdomen infections due to chlamydia spp	K67.0					
193	3				Peritoneal and intra abdomen infections due to neisseria gonorrheae	K67.1				Peritoneal and intra abdomen infections due to neisseria gonorrheae	647.1
194	3									Peritoneal and intra abdomen infections due to actinomyces	039.2
195	3				Peritoneal and intra abdomen infections due to treponema pallidum	K67.2					
196	2			Peritoneal and intra abdomen infections due to due to specific bacteria		N74.3-N74.4, O98.2			Peritoneal and intra abdomen infections due to due to specific bacteria		647.1

Table S3. List of infectious syndromes mapped to International Classification of Diseases (ICD) codes							Infectious Syndrome to ICD Codes (ICD9)				
	Infectious syndrome name and level					Infectious syndrome name and ICD code	Infectious syndrome name and level				Infectious syndrome name and ICD code
	Level	L0	L1	L2	L3	ICD Codes for all level	L0	L1	L2	L3	ICD Codes for all level
197	2			Peritoneal and intra abdomen infections due to unspecified Bacteria		K35-K37.9, K38.3-K38.9, K40-K42.9, K44-K46.9, K50-K52, K52.8-K52.9, K55-K57.9, K63.0-K63.1, K65-K65.9, K67, K67.8-K69, K75.0-K75.1, K75.3, K76.3, K77.0, K80-K83.9, M09.1, N70-N71.9, N73-N74.0, N74.8, N98.0, O98.3, O98.8-O98.9, R65.0, R85.5, R86.5, R87.5, O03.0, O41.1, O75.3, O98			Peritoneal and intra abdomen infections due to unspecified Bacteria		540-542.9, 550-551.1, 551.3-552.1, 552.3-553.1, 553.3-558.0, 560-560.3, 560.8-560.9, 562-562.1, 567-567.9, 569.5, 572.0-572.1, 574-576.9, 614-615.9, 647.0
199	0	No Sepsis, No infectious diseases and unspecified				C00-D64.9, D66-D69.4, D69.6-D70.2, D70.4-D89.2, D89.8-E87.1, E88-F02.0, F02.2-F02.3, F02.8-F07.0, F07.2-F99.0, G09-G13.8, G15-G21.2, G21.4-G93.3, G93.5-H05, H05.2-H60.1, H60.3-H61.9, H62.4-H62.8, H68.1-H69.9, H71-H73, H73.1-H74.9, H75.8-H99, I00.0, I03-I29.9, I30.0, I30.8-I32.8, I34-I37.9, I42-I45.9, I47-I75.8, I77-I79.8, I83-I87, I87.1-I89.0, I89.8-I95.0, I97-I98, I98.2-ID5.9, J07-J08, J23-J30.9, J33-J34.9, J38-J39, J39.2-J39.9, J42.9-J79, J81-J84.9, J85.9, J87-J91, J91.8-J95.1, J95.4-J95.9, J97-K03.9, K05.6-K34, K38-K38.2, K39, K43-K43.9, K47-K49, K52.0, K53-K54, K58-K60.5, K62-K63, K63.2-K64.9, K66-K66.9, K70-K71.9, K73-K75, K75.2, K75.4-K76.2, K76.4-K77, K77.8-K79, K84-K93, K93.8-K99, L09-L30.2, L30.4-L87.9, L90-L96, L98.5-L99.8, M02-M09.0, M09.2-M46.1, M46.8-M49, M49.2-M65, M65.2-M71, M71.2-M72.4, M72.8-M73, M74-M85.9, M87-M89.5, M89.7-M99.9, N02-N09, N13-N13.5, N13.7-N14.4, N15.0, N18-N29.8, N31-N33.8, N35-N39, N39.3-N40.9, N42-N44.8, N46-N48.9, N50-N69, N78-N98, N98.1-O03, O03.1-O03.4, O03.6-O04, O04.1-O04.4, O04.6-O05, O05.1-O05.4, O05.6-O06, O06.1-O06.4, O06.6-O07, O07.1-O07.4, O07.6-O08, O08.1-O22.9, O24-O41.0, O41.8-O75.2, O75.4-O84.9, O87-O88.2, O88.8-O90.9, O92-O97.9, O99-P00.1, P00.3-P21.9, P24-P34.2, P40-R01.2, R03-R09.1, R09.3-R19.6, R19.8-R40, R41-R54.9, R56-R56.9, R58-R65, R65.1, R66-R83.4, R83.6-R84.4, R84.6-R85.4, R85.6-R86.4, R86.6-R87.4, R87.6-T80.1, T80.3-T81.3, T81.5-T82.5, T82.8-T83.4, T83.7-T84.4, T84.8-T85.6, T85.8-T87.3, T87.5-T88, T88.1-U03, U05, U08-U81, U90-Y61.9, Y63-Y94, Y96-Z03, Z03.1-Z11.0, Z11.2-Z15.8, Z17-Z20.9, Z23-ZB0, D65-D65.9, D69.5, D70.3, D89.3, E87.2-E87.9, F02.1, F02.4, G93.4, I46-I46.9, I80-I82.9, I87.0, I95.1-I95.9, J80-J80.9, J95.2-J95.3, J96-J96.9, K72-K72.9, L98.2-L98.3, N00-N01.9, N17-N17.9, R09.2, R40.0-R40.4, R55-R55.0, R57-R57.9, T78-T80.1, T80.3-T81.3, T81.5-T82.5, T82.8-T83.4, T83.7-T84.4, T84.8-T85.6, T85.8-T87.3, T87.5-T88, T88.1-T98.3, Y40-Y61.9, Y63-Y84.9, Y88-Y88.3, S00-T76.9	No Sepsis, No infectious diseases and unspecified				000-000.9, 019-019.9, 105-109.9, 140-275.5, 277-286.5, 286.7-292.9, 294.0-319.9, 327-346.9, 348-376, 376.2-380.9, 384-389.9, 393-419.9, 425-425.5, 425.7-427.4, 427.6-450, 454-456.9, 459-459.9, 470, 470.9-471.9, 477-479, 492-509, 512-512.9, 514-519.0, 520-521.9, 524-539.9, 543-549, 551.2, 552.2, 553.2, 558.1, 559-559.0, 560.4-560.7, 561, 562.2-566.9, 568-569.4, 569.6-572, 572.2-573.9, 577-583.9, 585-589.9, 591-594.9, 596-596.9, 598-599, 599.1-600.9, 602-603.9, 605-613, 617-634, 634.1-634.9, 635.1-635.4, 635.6-636, 636.1-636.4, 636.6-636.9, 637.1-637.4, 637.6-638, 638.1-639, 639.1-646.9, 648-658.3, 658.8-659.2, 659.4-669.0, 669.2-669.9, 671-671.1, 671.8-671.9, 673-674.9, 676-679.1, 690-706.9, 708-710.9, 712-729.9, 731-768.7, 769.0-770, 770.1-770.9, 772-779.9, 780.4-785.3, 785.6-790.6, 790.8-797.9, 799.3-995.8, 996-996.5, 996.7-E871.9, E873-E999.1, 800-995.8, E000-E871.9, E873-E930, E950-E999.1, 996-996.5, 996.7-999.9, E930.0-E949.9, 135-135.9, 275.8-276.9, 286.6, 293-294, 347-347.9, 427.5, 451-453.9, 457.0, 458-458.9, 519.1, 519.3-519.4, 584-584.9, 635.5, 636.5, 637.5, 669.1, 768.9-769, 780-780.3, 785.5, 798-799.2, 135-135.9, 275.8-276.9, 286.6, 293-294, 347-347.9, 427.5, 451-453.9, 457.0, 458-458.9, 519.1, 519.3-519.4, 584-584.9, 635.5, 636.5, 637.5, 669.1, 768.9-769, 780-780.3, 785.5, 798-799.2

Table S4. Summary of case fatality ratio data

Pathogen	Hospital	Literature	Microbiology	Total
Acinetobacter baumannii			287390	287390
Adenovirus	3904			3904
Aeromonas spp.			280	280
Campylobacter spp.	24330		62	24392
Chlamydia spp.	14362			14362
Citrobacter spp.			156376	156376
Clostridium difficile	415011		7171	422182
Cryptosporidium	2010			2010
Entamoeba histolytica	9438			9438
Enterobacter spp.			730786	730786
Enterococcus faecalis			177234	177234
Enterococcus faecium			242584	242584
Other enterococci	6771		15062	21833
Escherichia coli	430064		2393259	2823323
Fungi	702794		6052	708846
Group A *Streptococcus*	280314		80761	361075
Group B *Streptococcus*	165579	2726	172333	340638
Haemophilus influenzae	56781	2134	197294	256209
Klebsiella pneumoniae	52983		1212459	1265442
Other *Klebsiella* species	39855		140357	180212
Legionella spp.	1396		54	1450
Listeria monocytogenes	5502		41	5543
Morganella spp.			87403	87403
Mycobacterium tuberculosis	102402		3032	105434
Mycoplasma spp.	53762			53762
Neisseria gonorrhoeae	11483		5540	17023
Neisseria meningitidis	9505	20952	110	30567
Non-typhoidal *Salmonella*	6194		172130	178324
Norovirus	8266			8266
Other pathogens	920212		357838	1278050
Polybacterial			92	92
Proteus spp.	41032		405684	446716
Providencia spp.			47268	47268
Pseudomonas aeruginosa	21773		1010873	1032646
Rotavirus	22818			22818
Salmonella Paratyphi	818		364	1182
Salmonella Typhi	5010		5088	10098
Serratia spp.	6497		260684	267181
Shigella spp.	4273		11361	15634
Staphylococcus aureus	932990		2545845	3478835
Streptococcus pneumoniae	214761	27095	441302	683158
Vibrio cholerae	740		6	746
Viruses	3903630		2	3903632
TOTAL	8477260	52907	11174177	19704344

Table S5. Case fatality ratio modelling framework by pathogen and syndrome

BSI = bloodstream infections. CNS = meningitis and other bacterial central nervous system infections. LRI+ = lower respiratory infections and all related infections in the thorax. Intra-abdominal = peritoneal and intra-abdominal infections. Skin = bacterial infections of the skin and subcutaneous systems. UTI = urinary tract infections and pyelonephritis.

	**BSI (neonatal)**	**BSI (post-neonatal and older)**	**CNS**	**Diarrhoea**	**LRI+ (community- acquired)**	**LRI+ (hospital-acquired)**	**Intra-abdominal**	**Skin**	**UTI (community-acquired)**	**UTI (hospital-acquired)**
Acinetobacter baumannii	**intercept**	**interaction**	not explicitly modelled	not explicitly modelled	**intercept**	**intercept**	not explicitly modelled	**intercept**	**intercept**	**intercept**
Campylobacter spp.	not explicitly modelled	not explicitly modelled	not explicitly modelled	**intercept**	not explicitly modelled	not explicitly modelled	not explicitly modelled	not explicitly modelled	not explicitly modelled	not explicitly modelled
Chlamydia spp.	not explicitly modelled	not explicitly modelled	not explicitly modelled	not explicitly modelled	**intercept**	not explicitly modelled	not explicitly modelled	not explicitly modelled	not explicitly modelled	not explicitly modelled
Citrobacter spp.	not explicitly modelled	**intercept**	not explicitly modelled	not explicitly modelled	not explicitly modelled	not explicitly modelled	not explicitly modelled	not explicitly modelled	**intercept**	**intercept**
Clostridium difficile	not explicitly modelled	not explicitly modelled	not explicitly modelled	**intercept**	not explicitly modelled	not explicitly modelled	not explicitly modelled	not explicitly modelled	not explicitly modelled	not explicitly modelled
Cryptosporidium	not explicitly modelled	not explicitly modelled	not explicitly modelled	**intercept**	not explicitly modelled	not explicitly modelled	not explicitly modelled	not explicitly modelled	not explicitly modelled	not explicitly modelled
Entamoeba histolytica	not explicitly modelled	not explicitly modelled	not explicitly modelled	**intercept**	not explicitly modelled	not explicitly modelled	not explicitly modelled	not explicitly modelled	not explicitly modelled	not explicitly modelled
Enterobacter spp.	**intercept**	**interaction**	not explicitly modelled	not explicitly modelled	**intercept**	**intercept**	**intercept**	**intercept**	**intercept**	**intercept**
Enterococcus faecalis	not explicitly modelled	**intercept**	not explicitly modelled	not explicitly modelled	not explicitly modelled	not explicitly modelled	not explicitly modelled	not explicitly modelled	not explicitly modelled	not explicitly modelled
Enterococcus faecium	not explicitly modelled	**intercept**	not explicitly modelled	not explicitly modelled	not explicitly modelled	not explicitly modelled	**intercept**	not explicitly modelled	**intercept**	**intercept**
Other Enterococci	not explicitly modelled	**intercept**	not explicitly modelled	not explicitly modelled	not explicitly modelled	not explicitly modelled	not explicitly modelled	**intercept**	not explicitly modelled	not explicitly modelled
Escherichia coli	**intercept**	**interaction**	**intercept**	**intercept**	**intercept**	**intercept**	**intercept**	**intercept**	**intercept**	**intercept**
Fungus	**intercept**	**interaction**	not explicitly modelled	not explicitly modelled	**intercept**	**intercept**	not explicitly modelled	not explicitly modelled	not explicitly modelled	not explicitly modelled
Group A *Streptococcus*	**intercept**	not explicitly modelled	not explicitly modelled	not explicitly modelled	**intercept**	not explicitly modelled	not explicitly modelled	**intercept**	not explicitly modelled	not explicitly modelled
Group B *Streptococcus*	**intercept**	**interaction**	**intercept**	not explicitly modelled	**intercept**	**intercept**	not explicitly modelled	not explicitly modelled	not explicitly modelled	not explicitly modelled
Haemophilus influenzae	not explicitly modelled	not explicitly modelled	**interaction**	not explicitly modelled	**intercept**	**intercept**	not explicitly modelled	not explicitly modelled	not explicitly modelled	not explicitly modelled
Klebsiella pneumoniae	**intercept**	**interaction**	**intercept**	not explicitly modelled	**intercept**	**intercept**	**intercept**	**intercept**	**intercept**	**intercept**
Legionella spp.	not explicitly modelled	not explicitly modelled	not explicitly modelled	not explicitly modelled	**intercept**	**intercept**	not explicitly modelled	not explicitly modelled	not explicitly modelled	not explicitly modelled
Listeria monocytogenes	not explicitly modelled	not explicitly modelled	**intercept**	not explicitly modelled	not explicitly modelled	not explicitly modelled	not explicitly modelled	not explicitly modelled	not explicitly modelled	not explicitly modelled
Moraxella spp.	not explicitly modelled	not explicitly modelled	not explicitly modelled	not explicitly modelled	**intercept**	not explicitly modelled	not explicitly modelled	not explicitly modelled	not explicitly modelled	not explicitly modelled
Morganella spp.	not explicitly modelled	not explicitly modelled	not explicitly modelled	not explicitly modelled	not explicitly modelled	not explicitly modelled	not explicitly modelled	not explicitly modelled	**intercept**	**intercept**
Mycoplasma spp.	not explicitly modelled	not explicitly modelled	not explicitly modelled	not explicitly modelled	**intercept**	**intercept**	not explicitly modelled	not explicitly modelled	not explicitly modelled	not explicitly modelled
Neisseria meningitidis	**intercept**	**intercept**	**intercept**	not explicitly modelled	not explicitly modelled	not explicitly modelled	not explicitly modelled	not explicitly modelled	not explicitly modelled	not explicitly modelled
Polybacterial	**intercept**	**interaction**	not explicitly modelled	not explicitly modelled	**intercept**	**intercept**	not explicitly modelled	not explicitly modelled	not explicitly modelled	not explicitly modelled
Proteus spp.	not explicitly modelled	not explicitly modelled	not explicitly modelled	not explicitly modelled	not explicitly modelled	not explicitly modelled	not explicitly modelled	**intercept**	**intercept**	**intercept**
Providencia spp.	not explicitly modelled	not explicitly modelled	not explicitly modelled	not explicitly modelled	not explicitly modelled	not explicitly modelled	not explicitly modelled	not explicitly modelled	**intercept**	**intercept**
Pseudomonas aeruginosa	not explicitly modelled	**interaction**	not explicitly modelled	not explicitly modelled	**intercept**	**intercept**	**intercept**	**intercept**	**intercept**	**intercept**
Salmonella Typhi	not explicitly modelled	not explicitly modelled	not explicitly modelled	not explicitly modelled	not explicitly modelled	not explicitly modelled	not explicitly modelled	not explicitly modelled	not explicitly modelled	not explicitly modelled
Non-typhoidal *Salmonella*	not explicitly modelled	**intercept**	not explicitly modelled	**intercept**	not explicitly modelled	not explicitly modelled	not explicitly modelled	not explicitly modelled	not explicitly modelled	not explicitly modelled
Serratia spp.	**intercept**	**intercept**	not explicitly modelled	not explicitly modelled	not explicitly modelled	not explicitly modelled	not explicitly modelled	not explicitly modelled	**intercept**	**intercept**
Shigella spp.	not explicitly modelled	not explicitly modelled	not explicitly modelled	**intercept**	not explicitly modelled	not explicitly modelled	not explicitly modelled	not explicitly modelled	not explicitly modelled	not explicitly modelled
Staphylococcus aureus	**intercept**	**interaction**	**intercept**	**intercept**	**intercept**	**intercept**	**intercept**	**intercept**	**intercept**	**intercept**
Streptococcus pneumoniae	**intercept**	**intercept**	**interaction**	not explicitly modelled	**intercept**	**intercept**	not explicitly modelled	not explicitly modelled	not explicitly modelled	not explicitly modelled
Vibrio cholerae	not explicitly modelled	not explicitly modelled	not explicitly modelled	**intercept**	not explicitly modelled	not explicitly modelled	not explicitly modelled	not explicitly modelled	not explicitly modelled	not explicitly modelled
Virus	not explicitly modelled	not explicitly modelled	**interaction**	**intercept**	**intercept**	**intercept**	not explicitly modelled	not explicitly modelled	not explicitly modelled	not explicitly modelled

Table S6. Summary of pathogen distribution data							
**Pathogen**	**CHAMPS**	**Hospital**	**Linkage**	**Literature**	**MCOD**	**Microbiology**	**Total**
Acinetobacter baumannii	162			2449		95614	98225
Chlamydia spp.		14362	7	481	730	1	15581
Citrobacter spp.				3330		64006	67336
Enterobacter spp.	9			9357		263748	273114
Enterococcus faecalis	13			13858		338708	352579
Enterococcus faecium	11			1464		411508	412983
Other enterococci	2	6771	44	12749	1	7351	26918
Escherichia coli	79	430064	482	205847	4628	4964020	5605120
Fungi	41	702794	334	8276	92562	6405	810412
Group A *Streptococcus*	9	280314	258	1947	48148	34637	365313
Group B *Streptococcus*	16	165579	203	8656	31183	8898	214535
Haemophilus influenzae	42	56781	310	5900	7831	25840	96704
Klebsiella pneumoniae	259	52983	144	22780	19126	1369389	1464681
Other *Klebsiella* species		39855	116	17240	28	13800	71039
Legionella spp.		1396	35	112	3726	54	5323
Listeria monocytogenes	5	5502	38	221	10416	41	16223
Morganella spp.				1480		63705	65185
Mycobacterium tuberculosis	6	102402	126	945	661806	3063	768348
Mycoplasma spp.		53762	56	1061	2722		57601
Neisseria gonorrhoeae		11483	1	115	220	5162	16981
Neisseria meningitidis	3	9505	107	10921	14304	5719	40559
Non-typhoidal *Salmonella*	9	6194	17	472	2002	34478	43172
Other bacteria	130	920212	1202	49723	299761	143107	1414135
Polybacterial	1			4565		115	4681
Proteus spp.	2	41032	52	19493	180	328345	389104
Providencia spp.				592		35144	35736
Pseudomonas aeruginosa	26	21773	541	17358	25725	819446	884869
Salmonella Paratyphi	1	818			29	365	1213
Salmonella Typhi		5010	1	4	573	10892	16480
Serratia spp.		6497	3	1360		96314	104174
Staphylococcus aureus	57	932990	1936	19521	227064	3109956	4291524
Streptococcus pneumoniae	100	214761	909	11044	130684	623928	981426
Viruses	164	3903630	2779	46049	2064708	4	6017334
TOTAL	1147	7986470	9701	499370	3648157	12883763	25028608

Table S7. Summary of prevalence of resistance data

Pathogen	Antibiotic Class	Literature	Microbiology	Surveillance report	Total
Acinetobacter baumannii	Aminoglycosides		52393		52393
Acinetobacter baumannii	Anti-pseudomonal penicillin/Beta-lactamase inhibitors		15723		15723
Acinetobacter baumannii	Beta-lactam/Beta-lactamase inhibitors		20205		20205
Acinetobacter baumannii	Carbapenem		43437		43437
Acinetobacter baumannii	Fluoroquinolones		38766		38766
Acinetobacter baumannii	Fourth-generation cephalosporins		26953		26953
Acinetobacter baumannii	Third-generation cephalosporins		49034		49034
Citrobacter spp.	Aminoglycosides		194936	4996	199932
Citrobacter spp.	Anti-pseudomonal penicillin/Beta-lactamase inhibitors		229701	2931	232632
Citrobacter spp.	Carbapenem		273016	6907	279923
Citrobacter spp.	Fluoroquinolones		240317	5862	246179
Citrobacter spp.	Fourth-generation cephalosporins		126201	2931	129132
Citrobacter spp.	Third-generation cephalosporins		353349	8793	362142
Enterobacter spp.	Aminoglycosides		1050287	6737	1057024
Enterobacter spp.	Anti-pseudomonal penicillin/Beta-lactamase inhibitors		766210	6072	772282
Enterobacter spp.	Carbapenem		891579	14916	906495
Enterobacter spp.	Fluoroquinolones		785309	12809	798118
Enterobacter spp.	Fourth-generation cephalosporins		417357		417357
Enterobacter spp.	Trimethoprim-Sulfamethoxazole		437597		437597
Enterococcus faecalis	Fluoroquinolones		637781		637781
Enterococcus faecalis	Vancomycin		846045		846045
Enterococcus faecium	Fluoroquinolones		193447		193447
Enterococcus faecium	Vancomycin		353515		353515
Other Enterococci	Fluoroquinolones		801		801
Other Enterococci	Vancomycin		3287		3287
Escherichia coli	Aminoglycosides		10524486	13012	10537498
Escherichia coli	Aminopenicillin		5162728	3582	5166310
Escherichia coli	Beta-lactam/Beta-lactamase inhibitors		4547346	3582	4550928
Escherichia coli	Carbapenem		8443688	224	8443912
Escherichia coli	Fluoroquinolones	946267	6505222	2398783	9850272
Escherichia coli	Third-generation cephalosporins	433884	2043110	2900403	5377397
Escherichia coli	Trimethoprim-Sulfamethoxazole		3459423	7164	3466587
Group A *Streptococcus*	Macrolide		10915	7933	18848
Group B *Streptococcus*	Fluoroquinolones		20050		20050
Group B *Streptococcus*	Macrolide		34507		34507
Group B *Streptococcus*	Penicillin		20424		20424
Haemophilus influenzae	Aminopenicillin		297932	3128	301060
Haemophilus influenzae	Third-generation cephalosporins		476427	2900	479327
Klebsiella pneumoniae	Aminoglycosides		3453036		3453036
Klebsiella pneumoniae	Beta-lactam/Beta-lactamase inhibitors		1556408		1556408
Klebsiella pneumoniae	Carbapenem	268169	873691	1182391	2324251
Klebsiella pneumoniae	Fluoroquinolones		2551051		2551051
Klebsiella pneumoniae	Third-generation cephalosporins	245676	851217	1059474	2156367
Klebsiella pneumoniae	Trimethoprim-Sulfamethoxazole		1225771		1225771
Morganella spp.	Fluoroquinolones		48697	3054	51751
Morganella spp.	Fourth-generation cephalosporins		23231	1527	24758
Morganella spp.	Third-generation cephalosporins		49780	4581	54361
Mycobacterium tuberculosis	Extensive drug resistance		15210		15210
Mycobacterium tuberculosis	Isoniazid mono-resistance		1980958	81360	2062318
Mycobacterium tuberculosis	Multi-drug resistance		14028		14028
Mycobacterium tuberculosis	Rifampicin mono-resistance		2846360	54169	2900529
Neisseria gonorrhoeae	Fluoroquinolones		27356		27356
Neisseria gonorrhoeae	Third-generation cephalosporins		49779		49779
Non-typhoidal *Salmonella*	Fluoroquinolones	6660	14		6674
Proteus spp.	Aminoglycosides		553876	3816	557692
Proteus spp.	Aminopenicillin		334346		334346
Proteus spp.	Fluoroquinolones		538122	7632	545754
Proteus spp.	Third-generation cephalosporins		656754	11448	668202
Proteus spp.	Trimethoprim-Sulfamethoxazole		171233		171233
Pseudomonas aeruginosa	Aminoglycosides		2801891		2801891
Pseudomonas aeruginosa	Anti-pseudomonal penicillin/Beta-lactamase inhibitors		2029407		2029407
Pseudomonas aeruginosa	Carbapenem		2311724		2311724
Pseudomonas aeruginosa	Fluoroquinolones		2331732		2331732
Pseudomonas aeruginosa	Fourth-generation cephalosporins		1138828		1138828
Pseudomonas aeruginosa	Third-generation cephalosporins		1315517		1315517
Salmonella Paratyphi	Fluoroquinolones	11123	6		11129
Salmonella Paratyphi	Multi-drug resistance	31383	12	237	31632
Salmonella Typhi	Fluoroquinolones	41718	162	1528	43408
Salmonella Typhi	Multi-drug resistance	113258	3608	6593	123459
Serratia spp.	Aminoglycosides		261873	1652	263525
Serratia spp.	Anti-pseudomonal penicillin/Beta-lactamase inhibitors		302571	1652	304223
Serratia spp.	Carbapenem		355742	3986	359728

Serratia spp.	Fluoroquinolones		322402	3304	325706
Serratia spp.	Fourth-generation cephalosporins		167538	1652	169190
Serratia spp.	Third-generation cephalosporins		419093	4956	424049
Shigella spp.	Fluoroquinolones	72148	13911	53514	139573
Staphylococcus aureus	Fluoroquinolones		4009367		4009367
Staphylococcus aureus	Macrolide		5963254		5963254
Staphylococcus aureus	Methicillin	178620	1893838	2471297	4543755
Staphylococcus aureus	Trimethoprim-Sulfamethoxazole		2645319		2645319
Staphylococcus aureus	Vancomycin		2859122		2859122
Streptococcus pneumoniae	Beta-lactam/Beta-lactamase inhibitors		5968		5968
Streptococcus pneumoniae	Carbapenem		298257		298257
Streptococcus pneumoniae	Fluoroquinolones		527610		527610
Streptococcus pneumoniae	Macrolide		760157	2687	762844
Streptococcus pneumoniae	Penicillin	60129	259584	314432	634145
Streptococcus pneumoniae	Third-generation cephalosporins		787948	2669	790617
Streptococcus pneumoniae	Trimethoprim-Sulfamethoxazole		31189	2684	33873
TOTAL		2409035	101202814	10695960	114307809

Table S8. Covariates for each core pathogen–drug combination (excluding 3GC-resistant *N. gonorrhoeae*)

Covariate name	MDR *S.* Typhi	FQ *S.* Typhi	MDR *S.* Paratyphi A	FQ *S.* Paratyphi A	FQ iNTS	FQ *Shigella* spp.	FQ *E. coli*	3GC *E. coli*	3GC *K. pneumoniae*	Carbapenems *K. pneumoniae*	Penicillin *S. penumoniae*	Methicillin *S. aureus*	Mono-Isoniazid TB	Mono-Rifampicin TB
Alcohol use[23]														▤
Antenatal care[1]	■													
Antibiotic usage proportion[3]				▤		■								
Antimalarial effectiveness[46]	▤													
BCG coverage[33]														▤
Childhood stunting[47]	▤	■												
Corruption Index[48]													■	▥
Diabetes[1]				■	■		■	■	■			■		▤
DPT coverage[33]	■													
DDD per 1000[3]							■	■	■	■				
Fraction of OOP health expenditure[49]							■	■			■			■
Handwashing[23]					■	■	■	■						
Healthcare Access and Quality Index[50]										■				
HIB coverage[33]					■									
HIV death rate (adults)[1]													■	
HIV incidence[1]				▥	■									
HIV prevalence per capita[1]									■					▥
Hospital beds per 1000 population[1]	▤		■	▥	■					■	■		▥	■
Improved sanitation[23]	■		■											
Improved water[23]				■										
J01C antibiotic consumption[3]	▤										■	■		
J01D antibiotic consumption[3]									■	■				
J01G antibiotic consumption[3]											■			
J01M antibiotic consumption[3]		■										■		
Lag-distributed income[12]		■							■	■			■	
Latitude					■	■	■	■		■				■
Mean temperature[1*]	■		■	■									▥	▥
Maternal education[51]											■		■	▥
Oral rehydration therapy[1*]					■	■								
Outdoor air pollution[52]		■	■	■	■									
Paratyphoid incidence[1]			■											
PCV3 coverage[33]											■			
Physicians per capita[1]			■				■	■	■			■	■	■
Pharmacists per capita[53]					■	■					■	■		
Pigs per capita[1*]							■	■						
Population density[1*]													■	▤
Proportion of underweight adults[1]														
Prop of pop in agricultural activities[23]							■	■						▥
Quality assessment (typhoid)	■	■	■	■										
Skilled birth attendant coverage[54]					■									
Smoking prevalence[23]														
Socio-demographic Index[12]														
Total antibiotic consumption[3]	■		■	■	■									
Total fertility rate[12]	▤	■	■											
TB prevalence[1]													■	▤
TB strain transmission risk[1]													■	■
Typhoid incidence[1]	■													

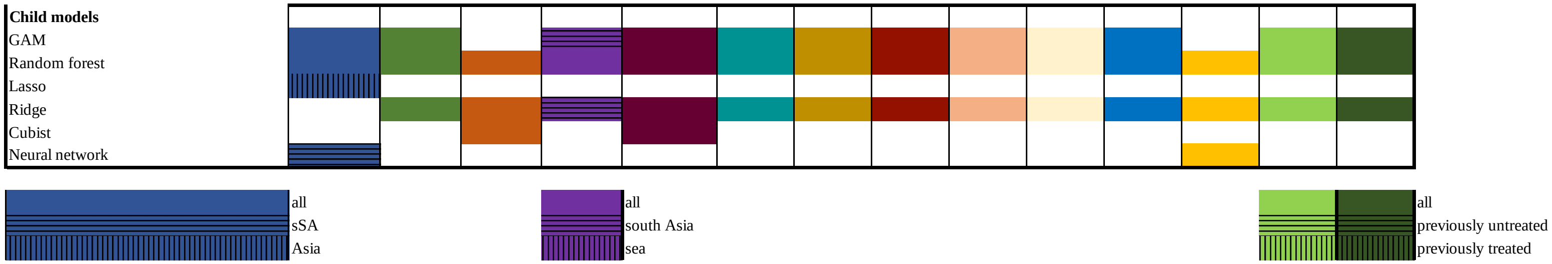

*See Appendix Section 7.4 for more details

Table S9. Summary of relative risk data

Pathogen	Antibiotic class	Literature	Microbiology	Total
Acinetobacter baumannii	Aminoglycosides		26547	26547
Acinetobacter baumannii	Anti-pseudomonal penicillin/Beta-lactamase inhibitors		8594	8594
Acinetobacter baumannii	Beta-lactam/Beta-lactamase inhibitors		10107	10107
Acinetobacter baumannii	Carbapenem		33071	33071
Acinetobacter baumannii	Fluoroquinolones		21091	21091
Acinetobacter baumannii	Fourth-generation cephalosporins		17670	17670
Acinetobacter baumannii	Third-generation cephalosporins		31653	31653
Citrobacter spp.	Aminoglycosides		27196	27196
Citrobacter spp.	Anti-pseudomonal penicillin/Beta-lactamase inhibitors		9695	9695
Citrobacter spp.	Carbapenem		18268	18268
Citrobacter spp.	Fluoroquinolones		21393	21393
Citrobacter spp.	Fourth-generation cephalosporins		9533	9533
Citrobacter spp.	Third-generation cephalosporins		22418	22418
Enterobacter spp.	Aminoglycosides		99118	99118
Enterobacter spp.	Anti-pseudomonal penicillin/Beta-lactamase inhibitors		40914	40914
Enterobacter spp.	Carbapenem		69258	69258
Enterobacter spp.	Fluoroquinolones		76075	76075
Enterobacter spp.	Fourth-generation cephalosporins		38593	38593
Enterobacter spp.	Trimethoprim-Sulfamethoxazole		44611	44611
Enterococcus faecalis	Fluoroquinolones		1948	1948
Enterococcus faecalis	Vancomycin		2832	2832
Enterococcus faecium	Fluoroquinolones		20642	20642
Enterococcus faecium	Vancomycin		37044	37044
Other Enterococci	Fluoroquinolones		670	670
Other Enterococci	Vancomycin	5948	8827	14775
Escherichia coli	Aminoglycosides		843033	843033
Escherichia coli	Aminopenicillin		372390	372390
Escherichia coli	Beta-lactam/Beta-lactamase inhibitors		439472	439472
Escherichia coli	Carbapenem	220	545090	545310
Escherichia coli	Fluoroquinolones	5734	646667	652401
Escherichia coli	Third-generation cephalosporins	15176	634823	649999
Escherichia coli	Trimethoprim-Sulfamethoxazole		394397	394397
Group A *Streptococcus*	Macrolide		413	413
Group B *Streptococcus*	Fluoroquinolones		135	135
Group B *Streptococcus*	Macrolide	432	208	640
Group B *Streptococcus*	Penicillin		128	128
Haemophilus influenzae	Aminopenicillin	1403	1577	2980
Haemophilus influenzae	Third-generation cephalosporins		553	553
Klebsiella pneumoniae	Aminoglycosides		293876	293876
Klebsiella pneumoniae	Beta-lactam/Beta-lactamase inhibitors		157604	157604
Klebsiella pneumoniae	Carbapenem	5019	204322	209341
Klebsiella pneumoniae	Fluoroquinolones		222853	222853
Klebsiella pneumoniae	Third-generation cephalosporins	1507	226729	228236
Klebsiella pneumoniae	Trimethoprim-Sulfamethoxazole		135376	135376
Morganella spp.	Fluoroquinolones		22376	22376
Morganella spp.	Fourth-generation cephalosporins		10417	10417
Morganella spp.	Third-generation cephalosporins		23186	23186
Mycobacterium tuberculosis	Extensive drug resistance		15210	15210
Mycobacterium tuberculosis	Multi-drug resistance		14028	14028
Neisseria gonorrhoeae	Fluoroquinolones		6	6
Neisseria gonorrhoeae	Third-generation cephalosporins		10	10
Non-typhoidal *Salmonella*	Fluoroquinolones		3059	3059
Proteus spp.	Aminoglycosides		171236	171236
Proteus spp.	Aminopenicillin		74260	74260
Proteus spp.	Fluoroquinolones		127450	127450
Proteus spp.	Third-generation cephalosporins		118679	118679
Proteus spp.	Trimethoprim-Sulfamethoxazole		77075	77075
Pseudomonas aeruginosa	Aminoglycosides		332015	332015
Pseudomonas aeruginosa	Anti-pseudomonal penicillin/Beta-lactamase inhibitors		138518	138518
Pseudomonas aeruginosa	Carbapenem	6141	180048	186189
Pseudomonas aeruginosa	Fluoroquinolones		218513	218513
Pseudomonas aeruginosa	Fourth-generation cephalosporins		129631	129631
Pseudomonas aeruginosa	Third-generation cephalosporins		132327	132327
Salmonella Paratyphi	Fluoroquinolones		66	66
Salmonella Typhi	Fluoroquinolones		795	795
Serratia spp.	Aminoglycosides		37402	37402
Serratia spp.	Anti-pseudomonal penicillin/Beta-lactamase inhibitors		11760	11760
Serratia spp.	Carbapenem		25054	25054
Serratia spp.	Fluoroquinolones		29148	29148

Serratia spp.	Fourth-generation cephalosporins		14622	14622
Serratia spp.	Third-generation cephalosporins		32521	32521
Shigella spp.	Fluoroquinolones		471	471
Staphylococcus aureus	Fluoroquinolones		292371	292371
Staphylococcus aureus	Macrolide		504053	504053
Staphylococcus aureus	Methicillin	25051	385688	410739
Staphylococcus aureus	Trimethoprim-Sulfamethoxazole		278366	278366
Staphylococcus aureus	Vancomycin		277781	277781
Streptococcus pneumoniae	Beta-lactam/Beta-lactamase inhibitors		2318	2318
Streptococcus pneumoniae	Carbapenem		4600	4600
Streptococcus pneumoniae	Fluoroquinolones	233	12940	13173
Streptococcus pneumoniae	Macrolide	871	20166	21037
Streptococcus pneumoniae	Penicillin	9172	44986	54158
Streptococcus pneumoniae	Third-generation cephalosporins	5100	37484	42584
Streptococcus pneumoniae	Trimethoprim-Sulfamethoxazole		9386	9386
TOTAL		82007	9627436	9709443

Table S10. Relative risk estimates for sterile sources of specimen across 88 pathogen–drug combinations

Pathogen	Drug	Sample size	Mean relative risk	Lower bound	Upper bound
Acinetobacter baumannii	Anti-pseudomonal penicillin/Beta-lactamase inhibitors	948	1.31	1.12	1.52
Acinetobacter baumannii	Beta-lactam/Beta-lactamase inhibitors	1555	1.27	1.11	1.44
Acinetobacter baumannii	Carbapenem	3232	1.42	1.27	1.58
Acinetobacter baumannii	Fourth-generation cephalosporins	1439	1.31	1.14	1.51
Acinetobacter baumannii	Third-generation cephalosporins	2055	1.35	1.13	1.62
Acinetobacter baumannii	Aminoglycosides	2066	1.1	0.97	1.25
Acinetobacter baumannii	Fluoroquinolones	3020	1.38	1.21	1.56
Citrobacter spp.	Aminoglycosides	4069	1.09	0.94	1.28
Citrobacter spp.	Anti-pseudomonal penicillin/Beta-lactamase inhibitors	3127	1.32	1.14	1.53
Citrobacter spp.	Carbapenem	3097	1.48	1.25	1.76
Citrobacter spp.	Fluoroquinolones	4387	1.36	1.18	1.57
Citrobacter spp.	Fourth-generation cephalosporins	2718	1.31	1.1	1.56
Citrobacter spp.	Third-generation cephalosporins	3984	1.38	1.16	1.64
Enterobacter spp.	Aminoglycosides	15211	1.19	1.06	1.34
Enterobacter spp.	Anti-pseudomonal penicillin/Beta-lactamase inhibitors	11857	1.23	1.13	1.34
Enterobacter spp.	Carbapenem	13299	1.53	1.4	1.67
Enterobacter spp.	Fluoroquinolones	17552	1.28	1.17	1.4
Enterobacter spp.	Fourth-generation cephalosporins	11482	1.31	1.18	1.45
Enterobacter spp.	Trimethoprim-Sulfamethoxazole	14798	1.09	0.98	1.21
Enterococcus faecalis	Fluoroquinolones	1126	1.43	1.24	1.64
Enterococcus faecalis	Vancomycin	36	1.7	1.39	2.07
Enterococcus faecium	Fluoroquinolones	4082	1.37	1.14	1.64
Enterococcus faecium	Vancomycin	9242	1.54	1.39	1.7
Other Enterococci	Fluoroquinolones	107	1.28	1.07	1.55
Other Enterococci	Vancomycin	7730	1.37	1.29	1.46
Escherichia coli	Aminoglycosides	164196	1.2	1.16	1.25
Escherichia coli	Aminopenicillin	157276	1.21	1.17	1.25
Escherichia coli	Beta-lactam/Beta-lactamase inhibitors	143458	1.15	1.11	1.18
Escherichia coli	Carbapenem	131382	1.7	1.5	1.93
Escherichia coli	Trimethoprim-Sulfamethoxazole	164240	1.14	1.11	1.18
Group A *Streptococcus*	Macrolide	130	1.07	0.89	1.29
Group B *Streptococcus*	Fluoroquinolones	44	1.26	1.04	1.53
Group B *Streptococcus*	Macrolide	465	1.18	0.99	1.41
Group B *Streptococcus*	Penicillin	15	1.29	1.06	1.57
Haemophilus influenzae	Aminopenicillin	1438	1.27	1.06	1.51
Haemophilus influenzae	Third-generation cephalosporins	308	1.48	1.23	1.79
Klebsiella pneumoniae	Aminoglycosides	51811	1.24	1.17	1.32
Klebsiella pneumoniae	Beta-lactam/Beta-lactamase inhibitors	46753	1.19	1.13	1.25
Klebsiella pneumoniae	Fluoroquinolones	53414	1.19	1.12	1.26
Klebsiella pneumoniae	Trimethoprim-Sulfamethoxazole	51737	1.12	1.06	1.19
Morganella spp.	Fluoroquinolones	3290	1.26	1.1	1.44
Morganella spp.	Fourth-generation cephalosporins	2352	1.23	1.02	1.49
Morganella spp.	Third-generation cephalosporins	3407	1.33	1.12	1.58
Proteus spp.	Aminoglycosides	21844	1.1	1.01	1.2
Proteus spp.	Aminopenicillin	20638	1.01	0.94	1.09
Proteus spp.	Fluoroquinolones	22141	1.13	1.05	1.21
Proteus spp.	Trimethoprim-Sulfamethoxazole	21838	1.06	0.98	1.14
Proteus spp.	Third-generation cephalosporins	18775	1.27	1.08	1.5
Pseudomonas aeruginosa	Aminoglycosides	39341	1.03	0.98	1.09
Pseudomonas aeruginosa	Anti-pseudomonal penicillin/Beta-lactamase inhibitors	36016	1.3	1.22	1.37
Pseudomonas aeruginosa	Carbapenem	41177	1.27	1.22	1.32
Pseudomonas aeruginosa	Fluoroquinolones	47417	1.19	1.15	1.23
Pseudomonas aeruginosa	Fourth-generation cephalosporins	34020	1.24	1.17	1.31
Pseudomonas aeruginosa	Third-generation cephalosporins	31041	1.35	1.15	1.59
Serratia spp.	Aminoglycosides	5250	1.05	0.93	1.19
Serratia spp.	Anti-pseudomonal penicillin/Beta-lactamase inhibitors	3003	1.17	1.01	1.35
Serratia spp.	Carbapenem	3639	1.39	1.2	1.63
Serratia spp.	Fluoroquinolones	5252	1.09	0.94	1.26
Serratia spp.	Fourth-generation cephalosporins	3928	1.17	0.99	1.38
Serratia spp.	Third-generation cephalosporins	5960	1.29	1.09	1.52
Staphylococcus aureus	Fluoroquinolones	37963	1.07	1.02	1.11
Staphylococcus aureus	Macrolide	53005	1.06	1.02	1.09
Staphylococcus aureus	Trimethoprim-Sulfamethoxazole	59632	1.17	1.09	1.25
Streptococcus pneumoniae	Beta-lactam/Beta-lactamase inhibitors	1419	1.14	0.95	1.37
Streptococcus pneumoniae	Carbapenem	1947	1.37	1.16	1.61
Streptococcus pneumoniae	Fluoroquinolones	6499	1.23	1.05	1.45
Streptococcus pneumoniae	Macrolide	7348	1.05	0.94	1.17
Streptococcus pneumoniae	Trimethoprim-Sulfamethoxazole	5413	1.14	1.01	1.28
Streptococcus pneumoniae	Third-generation cephalosporins	10457	1.33	1.13	1.57
Escherichia coli	Fluoroquinolones	171311	1.31	1.27	1.35
Escherichia coli	Third-generation cephalosporins	163801	1.37	1.17	1.61
Klebsiella pneumoniae	Carbapenem	41943	1.68	1.56	1.82
Klebsiella pneumoniae	Third-generation cephalosporins	52090	1.36	1.16	1.6
Mycobacterium tuberculosis	Extensive drug resistance	428524	2.59	2.46	2.72
Mycobacterium tuberculosis	Isoniazid mono-resistance	14537	1.19	0.84	1.67
Mycobacterium tuberculosis	Multidrug resistance	427342	2.5	1.17	4.74
Mycobacterium tuberculosis	Rifampicin mono-resistance	7161	1.39	1.06	1.77
Non-typhoidal *Salmonella*	Fluoroquinolones	42	1.23	1.01	1.5
Salmonella Paratyphi	Fluoroquinolones	24	1.24	1.02	1.52
Salmonella Paratyphi	Multidrug resistance	25	1.24	1.03	1.5
Salmonella Typhi	Fluoroquinolones	24	1.24	1.02	1.52
Salmonella Typhi	Multidrug resistance	25	1.24	1.03	1.5
Shigella spp.	Fluoroquinolones	24	1.24	1.02	1.52
Staphylococcus aureus	Methicillin	95696	1.43	1.2	1.7
Streptococcus pneumoniae	Penicillin	30849	1.27	1.18	1.36
Staphylococcus aureus	Vancomycin	53623	1.52	1.28	1.81

Sample size are the admission reported with known discharge disposition and antimicrobial susceptibility test.

Table S11: Decomposition of fatal antimicrobial resistance burden by GBD region, 2019

GBD region	All-cause death counts	All-cause death rate per 100 000	Fraction of all deaths that involve infection	Death counts involving infection	Death rate per 100 000 involving infection	Fraction of deaths involving infection that are associated with resistance	Death counts associated with resistance	Death rate per 100 000 associated with resistance	Fraction of deaths involving infection that are attributable to resistance	Death counts attributable to resistance	Death rate per 100 000 attributable to resistance
Central Asia	637,000 (587,000–697,000)	681.5 (628.0–744.7)	16.1% (11.9%–21.5%)	102,000 (74,200–140,000)	109.5 (79.3–150.1)	48.6% (46.7%–50.2%)	49,800 (35,300–69,500)	53.3 (37.7–74.3)	12.6% (11.1%–14.3%)	12,900 (8,870–18,200)	13.8 (9.5–19.5)
Central Europe	1,370,000 (1,230,000–1,520,000)	1201.3 (1074.6–1329.3)	11.5% (7.9%–16.1%)	157,000 (106,000–227,000)	137.8 (92.5–198.7)	49.1% (46.6%–51.5%)	77,600 (49,400–115,000)	68.0 (43.2–100.9)	12.0% (10.7%–13.4%)	19,000 (12,000–28,500)	16.6 (10.5–25.0)
Eastern Europe	2,730,000 (2,520,000–2,970,000)	1300.9 (1202.0–1415.4)	11.5% (8.1%–16.0%)	315,000 (217,000–434,000)	150.0 (103.5–206.6)	49.2% (46.7%–51.6%)	155,000 (103,000–222,000)	74.0 (48.8–105.6)	13.3% (11.7%–15.0%)	41,800 (27,600–59,900)	19.9 (13.1–28.5)
Australasia	205,000 (202,000–209,000)	706.6 (695.7–717.7)	12.7% (8.9%–17.6%)	26,200 (18,400–36,100)	90.0 (63.2–124.4)	31.0% (29.2%–32.8%)	8,140 (5,460–11,600)	28.0 (18.8–39.9)	7.2% (6.2%–8.2%)	1,880 (1,250–2,730)	6.5 (4.3–9.4)
High-income Asia Pacific	1,740,000 (1,730,000–1,760,000)	931.0 (921.9–940.3)	18.3% (14.2%–23.1%)	319,000 (248,000–405,000)	170.2 (132.5–216.0)	41.5% (39.6%–43.4%)	132,000 (99,700–173,000)	70.7 (53.2–92.3)	9.7% (7.9%–11.5%)	30,900 (21,700–43,300)	16.5 (11.6–23.1)
High-income North America	3,240,000 (3,210,000–3,260,000)	887.4 (881.1–893.8)	13.8% (10.0%–18.9%)	448,000 (323,000–612,000)	122.8 (88.7–167.9)	41.4% (39.5%–43.2%)	186,000 (129,000–261,000)	51.0 (35.4–71.5)	10.0% (8.6%–11.5%)	44,800 (30,300–63,900)	12.3 (8.3–17.5)
Southern Latin America	496,000 (485,000–507,000)	742.7 (726.9–760.0)	22.7% (18.1%–28.4%)	112,000 (89,800–141,000)	168.3 (134.5–210.8)	42.8% (40.9%–44.9%)	48,200 (36,800–62,300)	72.3 (55.1–93.4)	11.0% (9.5%–12.6%)	12,400 (9,280–16,500)	18.6 (13.9–24.7)
Western Europe	4,280,000 (4,240,000–4,320,000)	979.9 (970.7–989.4)	14.3% (10.7%–19.1%)	613,000 (457,000–816,000)	140.5 (104.7–186.9)	37.2% (34.9%–39.6%)	229,000 (162,000–318,000)	52.5 (37.0–73.0)	8.3% (7.3%–9.4%)	51,100 (35,100–72,400)	11.7 (8.0–16.6)
Andean Latin America	321,000 (270,000–381,000)	504.6 (423.9–599.2)	27.3% (22.0%–33.7%)	87,500 (64,700–116,000)	137.6 (101.7–182.1)	45.9% (43.3%–48.0%)	40,200 (28,900–54,300)	63.2 (45.4–85.4)	11.5% (10.1%–13.2%)	10,100 (7,050–13,900)	15.9 (11.1–21.9)
Caribbean	379,000 (335,000–427,000)	804.0 (709.5–905.2)	22.5% (17.5%–28.6%)	85,200 (63,900–112,000)	180.6 (135.5–237.8)	35.9% (32.9%–38.8%)	30,700 (21,500–42,700)	65.1 (45.5–90.5)	8.9% (7.7%–10.2%)	7,630 (5,200–10,900)	16.2 (11.0–23.2)
Central Latin America	1,430,000 (1,260,000–1,630,000)	573.2 (503.8–651.2)	19.9% (14.7%–26.5%)	285,000 (204,000–391,000)	113.9 (81.4–156.2)	44.3% (42.0%–46.3%)	127,000 (86,700–177,000)	50.6 (34.7–70.9)	11.4% (10.0%–13.0%)	32,600 (22,000–46,200)	13.0 (8.8–18.5)
Tropical Latin America	1,450,000 (1,410,000–1,490,000)	646.3 (629.1–664.3)	22.0% (17.2%–27.9%)	318,000 (249,000–404,000)	142.2 (111.5–180.6)	44.2% (42.3%–46.1%)	141,000 (105,000–186,000)	63.0 (47.1–83.1)	10.7% (9.6%–11.9%)	34,000 (24,800–46,000)	15.2 (11.1–20.6)
North Africa and Middle East	3,100,000 (2,820,000–3,420,000)	509.7 (462.6–561.1)	18.1% (13.2%–24.2%)	563,000 (402,000–774,000)	92.5 (66.0–127.1)	45.3% (42.6%–47.7%)	256,000 (174,000–362,000)	42.0 (28.7–59.5)	12.1% (10.7%–13.5%)	68,300 (45,600–99,000)	11.2 (7.5–16.3)
South Asia	11,900,000 (10,900,000–13,000,000)	661.3 (604.0–721.0)	31.8% (26.0%–39.2%)	3,800,000 (3,040,000–4,730,000)	210.6 (168.3–262.2)	36.4% (30.8%–41.4%)	1,390,000 (1,030,000–1,830,000)	76.8 (57.2–101.2)	10.2% (8.1%–12.7%)	389,000 (273,000–538,000)	21.5 (15.1–29.8)
East Asia	11,100,000 (9,730,000–12,500,000)	752.3 (661.0–848.3)	12.2% (8.2%–17.5%)	1,350,000 (900,000–2,000,000)	91.6 (61.2–135.8)	47.1% (44.6%–49.2%)	638,000 (404,000–973,000)	43.3 (27.4–66.1)	11.4% (9.9%–13.0%)	154,000 (96,000–235,000)	10.5 (6.5–16.0)
Oceania	97,200 (80,000–118,000)	732.4 (602.3–891.7)	33.1% (27.0%–40.4%)	32,200 (23,300–42,900)	242.6 (175.7–323.3)	29.5% (24.9%–33.0%)	9,500 (6,820–13,000)	71.6 (51.4–98.0)	7.0% (5.7%–8.3%)	2,260 (1,610–3,080)	17.0 (12.1–23.2)
Southeast Asia	4,390,000 (4,010,000–4,750,000)	651.3 (595.7–704.9)	25.8% (20.8%–32.0%)	1,130,000 (899,000–1,440,000)	168.2 (133.4–213.7)	32.4% (28.8%–35.7%)	369,000 (262,000–505,000)	54.8 (38.9–74.9)	8.5% (7.3%–9.9%)	97,000 (67,500–135,000)	14.4 (10.0–20.0)
Central Sub-Saharan Africa	872,000 (748,000–1,010,000)	662.8 (568.6–769.2)	48.0% (40.2%–56.1%)	418,000 (335,000–520,000)	317.7 (254.9–395.6)	27.0% (24.3%–30.1%)	113,000 (86,600–144,000)	86.0 (65.9–109.8)	6.5% (5.3%–8.1%)	27,200 (19,600–36,400)	20.7 (14.9–27.7)
Eastern Sub-Saharan Africa	2,590,000 (2,340,000–2,900,000)	628.3 (568.1–703.1)	50.7% (43.1%–58.8%)	1,310,000 (1,080,000–1,610,000)	318.9 (261.3–390.1)	27.9% (25.6%–30.1%)	366,000 (290,000–464,000)	89.0 (70.5–112.6)	6.7% (5.7%–7.9%)	88,200 (67,000–116,000)	21.4 (16.3–28.1)
Southern Sub-Saharan Africa	732,000 (688,000–787,000)	932.2 (875.3–1002.1)	45.6% (38.4%–52.7%)	334,000 (276,000–399,000)	425.4 (351.5–507.4)	18.6% (16.6%–20.9%)	62,400 (48,400–79,800)	79.4 (61.7–101.6)	4.6% (3.7%–5.7%)	15,300 (11,300–20,400)	19.4 (14.3–25.9)
Western Sub-Saharan Africa	3,460,000 (3,070,000–3,940,000)	757.8 (672.5–864.4)	53.5% (45.4%–62.0%)	1,850,000 (1,510,000–2,270,000)	405.5 (331.6–497.6)	28.3% (25.4%–30.8%)	524,000 (412,000–663,000)	114.8 (90.4–145.3)	6.7% (5.7%–7.7%)	125,000 (95,400–161,000)	27.3 (20.9–35.3)

Table S12. GATHER checklist

Item #	Checklist item	Reporting location:
**Objectives and funding**		
**1**	Define the indicator(s), populations (including age, sex, and geographic entities), and time period(s) for which estimates were made.	Main text methods section (overview)
**2**	List the funding sources for the work.	Main text funding statement and acknowledgements
**Data Inputs**		
For all data inputs from multiple sources that are synthesized as part of the study:		
**3**	Describe how the data were identified and how the data were accessed.	Main text methods section + supplementary appendix (sections 2, 4.1, 5.1, 6.1, 7.1, 8.1)
**4**	Specify the inclusion and exclusion criteria. Identify all ad-hoc exclusions.	Supplementary appendix (section 2)
**5**	Provide information on all included data sources and their main characteristics. For each data source used, report reference information or contact name/institution, population represented, data collection method, year(s) of data collection, sex and age range, diagnostic criteria or measurement method, and sample size, as relevant.	Some data source information in supplementary appendix (section 2); main characteristics of data, metadata, and/or NIDs available at http://ghdx.healthdata.org/record/ihme-data/global-bacterial-antimicrobial-resistance-burden-estimates-2019
**6**	Identify and describe any categories of input data that have potentially important biases (e.g., based on characteristics listed in item 5).	Main text limitations section + supplementary appendix (biases for input data in each modelling step identified in each section)
For data inputs that contribute to the analysis but were not synthesized as part of the study:		
**7**	Describe and give sources for any other data inputs.	GBD 2019 estimates (http://ghdx.healthdata.org/gbd-results-tool)
For all data inputs:		
**8**	Provide all data inputs in a file format from which data can be efficiently extracted (e.g., a spreadsheet rather than a PDF), including all relevant meta-data listed in item 5. For any data inputs that cannot be shared because of ethical or legal reasons, such as third-party ownership, provide a contact name or the name of the institution that retains the right to the data.	Data inputs and/or contact information available at http://ghdx.healthdata.org/record/ihme-data/global-bacterial-antimicrobial-resistance-burden-estimates-2019
**Data analysis**		
**9**	Provide a conceptual overview of the data analysis method. A diagram may be helpful.	Main text methods section + main text figure 1 (flowchart of methods)
**10**	Provide a detailed description of all steps of the analysis, including mathematical formulae. This description should cover, as relevant, data cleaning, data pre-processing, data adjustments and weighting of data sources, and mathematical or statistical model(s).	Supplementary appendix (sections 4–9)

**11**	Describe how candidate models were evaluated and how the final model(s) were selected.	Supplementary appendix (sections 4–8)
**12**	Provide the results of an evaluation of model performance, if done, as well as the results of any relevant sensitivity analysis.	Supplementary appendix (sections 4.7, 6.5, 7.6, 8.4)
**13**	Describe methods for calculating uncertainty of the estimates. State which sources of uncertainty were, and were not, accounted for in the uncertainty analysis.	Main text methods section (uncertainty analysis) + limitations section (scarcity of data) + supplementary appendix (sections 4–8)
**14**	State how analytic or statistical source code used to generate estimates can be accessed.	GitHub code found at: http://ghdx.healthdata.org/record/ihme-data/global-bacterial-antimicrobial-resistance-burden-estimates-2019
**Results and Discussion**		
**15**	Provide published estimates in a file format from which data can be efficiently extracted.	CSVs of tabulated estimates are available at: http://ghdx.healthdata.org/record/ihme-data/global-bacterial-antimicrobial-resistance-burden-estimates-2019
**16**	Report a quantitative measure of the uncertainty of the estimates (e.g. uncertainty intervals).	UIs provided for all estimates throughout the main text (summary, results, and discussion sections)
**17**	Interpret results in light of existing evidence. If updating a previous set of estimates, describe the reasons for changes in estimates.	Main text introduction and discussion sections
**18**	Discuss limitations of the estimates. Include a discussion of any modelling assumptions or data limitations that affect interpretation of the estimates.	Main text limitations section + supplementary appendix (sections 4–8; table S21)

Table S13. GBD location hierarchy with levels

Location	level
**Global**	**0**
**Central Europe, eastern Europe, and central Asia**	**1**
**Central Asia**	**2**
Armenia	3
Azerbaijan	3
Georgia	3
Kazakhstan	3
Kyrgyzstan	3
Mongolia	3
Tajikistan	3
Turkmenistan	3
Uzbekistan	3
**Central Europe**	**2**
Albania	3
Bosnia and Herzegovina	3
Bulgaria	3
Croatia	3
Czechia	3
Hungary	3
Montenegro	3
North Macedonia	3
Poland	3
Romania	3
Serbia	3
Slovakia	3
Slovenia	3
**Eastern Europe**	**2**
Belarus	3
Estonia	3
Latvia	3
Lithuania	3
Moldova	3
Russia	3
Ukraine	3
**High income**	**1**
**Australasia**	**2**
Australia	3
New Zealand	3

**High-income Asia Pacific**	**2**
Brunei	3
Japan	3
Singapore	3
South Korea	3
**High-income North America**	**2**
Canada	3
Greenland	3
USA	3
**Southern Latin America**	**2**
Argentina	3
Chile	3
Uruguay	3
**Western Europe**	**2**
Andorra	3
Austria	3
Belgium	3
Cyprus	3
Denmark	3
Finland	3
France	3
Germany	3
Greece	3
Iceland	3
Ireland	3
Israel	3
Italy	3
Luxembourg	3
Malta	3
Monaco	3
Netherlands	3
Norway	3
Portugal	3
San Marino	3
Spain	3
Sweden	3
Switzerland	3
UK	3
**Latin America and Caribbean**	**1**
**Andean Latin America**	**2**
Bolivia	3
Ecuador	3
Peru	3
**Caribbean**	**2**
Antigua and Barbuda	3
The Bahamas	3
Barbados	3

Belize	3
Bermuda	3
Cuba	3
Dominica	3
Dominican Republic	3
Grenada	3
Guyana	3
Haiti	3
Jamaica	3
Puerto Rico	3
Saint Kitts and Nevis	3
Saint Lucia	3
Saint Vincent and the Grenadines	3
Suriname	3
Trinidad and Tobago	3
Virgin Islands	3
**Central Latin America**	**2**
Colombia	3
Costa Rica	3
El Salvador	3
Guatemala	3
Honduras	3
Mexico	3
Nicaragua	3
Panama	3
Venezuela	3
**Tropical Latin America**	**2**
Brazil	3
Paraguay	3
**North Africa and Middle East**	**1**
**North Africa and Middle East**	**2**
Afghanistan	3
Algeria	3
Bahrain	3
Egypt	3
Iran	3
Iraq	3
Jordan	3
Kuwait	3
Lebanon	3
Libya	3
Morocco	3
Oman	3
Palestine	3
Qatar	3
Saudi Arabia	3
Sudan	3
Syria	3

Tunisia	3
Turkey	3
United Arab Emirates	3
Yemen	3
**South Asia**	**1**
**South Asia**	**2**
Bangladesh	3
Bhutan	3
India	3
Nepal	3
Pakistan	3
**Southeast Asia, east Asia, and Oceania**	**1**
**East Asia**	**2**
China	3
North Korea	3
Taiwan (province of China)	3
**Oceania**	**2**
American Samoa	3
Cook Islands	3
Fiji	3
Guam	3
Kiribati	3
Marshall Islands	3
Federated States of Micronesia	3
Nauru	3
Niue	3
Northern Mariana Islands	3
Palau	3
Papua New Guinea	3
Samoa	3
Solomon Islands	3
Tokelau	3
Tonga	3
Tuvalu	3
Vanuatu	3
**Southeast Asia**	**2**
Cambodia	3
Indonesia	3
Laos	3
Malaysia	3
Maldives	3
Mauritius	3
Myanmar	3
Philippines	3
Seychelles	3
Sri Lanka	3
Thailand	3
Timor-Leste	3

Vietnam	3
**Sub-Saharan Africa**	**1**
**Central sub-Saharan Africa**	**2**
Angola	3
Central African Republic	3
Congo (Brazzaville)	3
DR Congo	3
Equatorial Guinea	3
Gabon	3
Burundi	3
Comoros	3
Djibouti	3
Eritrea	3
Ethiopia	3
Kenya	3
Madagascar	3
Malawi	3
Mozambique	3
Rwanda	3
Somalia	3
South Sudan	3
Uganda	3
Tanzania	3
Zambia	3
**Southern sub-Saharan Africa**	**2**
Botswana	3
Eswatini	3
Lesotho	3
Namibia	3
South Africa	3
Zimbabwe	3
**Western sub-Saharan Africa**	**2**
Benin	3
Burkina Faso	3
Cape Verde	3
Cameroon	3
Chad	3
Côte d'Ivoire	3
The Gambia	3
Ghana	3
Guinea	3
Guinea-Bissau	3
Liberia	3
Mali	3
Mauritania	3
Niger	3
Nigeria	3
São Tomé and Príncipe	3

Senegal	3
Sierra Leone	3
Togo	3

Table S14. Deaths and DALYs (in counts and age-specific rates) associated with and attributable to bacterial antimicrobial resistance by pathogen–drug combination; by age group; 2019; central Europe, eastern Europe, and central Asia super-region

Pathogen	Antibiotic Class	Neonatal							
		Associated with resistance				Attributable to resistance			
		Deaths		DALYs		Deaths		DALYs	
		Counts	Rate per 100 000	Counts	Rate per 100 000	Counts	Rate per 100 000	Counts	Rate per 100 000
All pathogens	Resistance to one or more antibiotics	4540 (3240-6320)	1142.5 (816.3-1591.5)	404000 (288000-563000)	101677.2 (72609.5-141632.7)	1100 (772-1530)	277.2 (194.4-385.8)	97900 (68700-136000)	24661.7 (17295.1-34326.8)
Acinetobacter baumannii	Aminoglycosides	160 (99-241)	40.3 (24.8-60.8)	14200 (8770-21500)	3581.9 (2207.5-5407.9)	7 (0-17)	1.7 (0.0-4.2)	595 (1-1480)	149.9 (0.3-372.2)
Acinetobacter baumannii	Anti-pseudomonal penicillin/Beta-Lactamase inhibitors	206 (127-310)	51.8 (32.1-78.1)	18300 (11300-27600)	4606.3 (2853.8-6943.1)	7 (4-13)	1.8 (0.9-3.2)	647 (316-1140)	162.9 (79.6-288.1)
Acinetobacter baumannii	Beta Lactam/Beta-lactamase inhibitors	184 (114-277)	46.4 (28.7-69.6)	16400 (10100-24600)	4124.5 (2547.8-6194.4)	0 (0-0)	0.0 (0.0-0.1)	9 (3-20)	2.3 (0.6-4.9)
Acinetobacter baumannii	Carbapenem	171 (106-257)	43.1 (26.6-64.8)	15200 (9380-22900)	3830.6 (2362.0-5761.2)	28 (14-48)	6.9 (3.6-12.1)	2450 (1280-4260)	617.9 (321.5-1072.4)
Acinetobacter baumannii	Fluoroquinolones	193 (119-290)	48.7 (29.9-73.1)	17200 (10600-25800)	4330.3 (2660.5-6505.7)	25 (12-44)	6.2 (3.1-11.1)	2180 (1080-3920)	548.6 (273.0-987.6)
Acinetobacter baumannii	Fourth-generation cephalosporins	198 (123-299)	49.9 (31.0-75.3)	17600 (10900-26600)	4435.8 (2755.3-6698.4)	0 (0-0)	0.0 (0.0-0.1)	16 (6-33)	4.0 (1.5-8.4)
Acinetobacter baumannii	Third-generation cephalosporins	202 (125-304)	50.8 (31.4-76.6)	17900 (11100-27000)	4513.5 (2787.3-6807.9)	1 (0-1)	0.2 (0.1-0.3)	62 (23-123)	15.6 (5.7-30.9)
Acinetobacter baumannii	Resistance to one or more antibiotics	210 (130-316)	52.9 (32.8-79.6)	18700 (11600-28100)	4703.8 (2913.5-7081.6)	67 (39-106)	16.9 (9.9-26.6)	5960 (3500-9400)	1501.1 (881.2-2366.9)
Citrobacter spp.	Aminoglycosides	4 (2-7)	1.0 (0.5-1.8)	366 (193-646)	92.1 (48.6-162.6)	0 (0-1)	0.1 (0.0-0.2)	20 (0-55)	5.1 (0.0-13.9)
Citrobacter spp.	Anti-pseudomonal penicillin/Beta-Lactamase inhibitors	8 (4-15)	2.0 (1.0-3.8)	708 (345-1330)	178.3 (86.8-334.9)	1 (1-3)	0.4 (0.1-0.8)	133 (52-293)	33.5 (13.0-73.9)
Citrobacter spp.	Carbapenem	8 (4-13)	1.9 (1.0-3.3)	668 (357-1160)	168.2 (90.0-291.3)	2 (1-4)	0.5 (0.2-0.9)	164 (74-332)	41.3 (18.5-83.5)
Citrobacter spp.	Fluoroquinolones	11 (6-18)	2.7 (1.4-4.7)	952 (498-1650)	239.6 (125.3-414.3)	2 (1-3)	0.4 (0.2-0.8)	149 (59-300)	37.6 (14.9-75.4)
Citrobacter spp.	Fourth-generation cephalosporins	5 (3-10)	1.4 (0.7-2.4)	487 (252-851)	122.5 (63.5-214.3)	0 (0-1)	0.1 (0.0-0.2)	26 (10-54)	6.5 (2.5-13.7)
Citrobacter spp.	Third-generation cephalosporins	18 (9-31)	4.4 (2.3-7.8)	1570 (813-2750)	395.3 (204.7-692.4)	2 (1-4)	0.5 (0.2-1.0)	165 (61-362)	41.5 (15.2-91.2)
Citrobacter spp.	Resistance to one or more antibiotics	25 (13-43)	6.3 (3.3-10.8)	2240 (1160-3820)	564.1 (292.4-963.0)	7 (4-14)	1.9 (0.9-3.4)	657 (316-1200)	165.5 (79.7-302.4)
Enterobacter spp.	Aminoglycosides	43 (26-69)	10.9 (6.6-17.4)	3840 (2340-6140)	966.4 (590.3-1546.1)	3 (1-6)	0.7 (0.2-1.5)	247 (64-513)	62.3 (16.1-129.1)
Enterobacter spp.	Anti-pseudomonal penicillin/Beta-Lactamase inhibitors	140 (91-214)	35.4 (23.0-53.9)	12500 (8120-19000)	3145.7 (2044.1-4792.3)	19 (10-30)	4.7 (2.6-7.6)	1670 (904-2690)	420.4 (227.6-676.3)
Enterobacter spp.	Carbapenem	37 (23-57)	9.3 (5.9-14.4)	3290 (2080-5070)	829.5 (524.2-1277.7)	9 (5-15)	2.3 (1.3-3.8)	817 (459-1350)	205.7 (115.6-338.7)
Enterobacter spp.	Fluoroquinolones	48 (30-75)	12.0 (7.6-18.9)	4240 (2670-6680)	1066.5 (671.8-1682.5)	5 (2-9)	1.2 (0.6-2.2)	434 (212-789)	109.3 (53.5-198.7)
Enterobacter spp.	Fourth-generation cephalosporins	123 (80-184)	31.0 (20.0-46.4)	10900 (7080-16400)	2754.1 (1783.1-4132.7)	7 (3-12)	1.6 (0.8-3.0)	579 (269-1060)	145.7 (67.6-266.4)
Enterobacter spp.	Trimethoprim-Sulfamethoxazole	130 (83-202)	32.7 (20.9-50.8)	11600 (7360-17900)	2908.4 (1854.3-4513.5)	8 (0-17)	2.0 (0.0-4.3)	704 (0-1500)	177.2 (0.0-378.2)
Enterobacter spp.	Resistance to one or more antibiotics	230 (152-349)	57.8 (38.2-87.8)	20400 (13500-31000)	5143.9 (3400.5-7805.8)	50 (31-76)	12.6 (7.8-19.1)	4450 (2750-6740)	1120.6 (693.5-1698.2)
Enterococcus faecalis	Fluoroquinolones	93 (61-141)	23.3 (15.4-35.5)	8250 (5450-12600)	2078.3 (1371.0-3165.4)	23 (10-41)	5.8 (2.6-10.2)	2060 (924-3610)	518.6 (232.8-907.9)
Enterococcus faecalis	Vancomycin	8 (5-13)	2.0 (1.3-3.2)	717 (443-1130)	180.4 (111.6-285.4)	2 (1-5)	0.6 (0.3-1.2)	214 (91-413)	54.0 (22.9-103.9)
Enterococcus faecalis	Resistance to one or more antibiotics	96 (63-145)	24.1 (15.9-36.6)	8510 (5620-12900)	2143.5 (1415.6-3259.0)	26 (13-44)	6.4 (3.2-11.0)	2270 (1140-3880)	572.6 (286.1-978.2)
Enterococcus faecium	Fluoroquinolones	100 (65-159)	25.2 (16.4-40.0)	8920 (5780-14200)	2244.9 (1454.9-3564.7)	20 (6-38)	4.9 (1.5-9.5)	1740 (529-3340)	439.0 (133.1-841.7)
Enterococcus faecium	Vancomycin	22 (13-37)	5.5 (3.4-9.2)	1960 (1200-3250)	494.1 (301.9-818.8)	5 (2-10)	1.3 (0.6-2.4)	457 (213-865)	115.0 (53.7-217.7)
Enterococcus faecium	Resistance to one or more antibiotics	100 (65-159)	25.3 (16.4-40.0)	8950 (5800-14200)	2253.6 (1460.2-3571.3)	25 (12-44)	6.2 (3.0-11.2)	2200 (1080-3950)	554.0 (271.1-994.4)
Other enterococci	Fluoroquinolones	86 (49-143)	21.7 (12.3-35.9)	7680 (4340-12700)	1933.1 (1091.8-3190.4)	17 (2-33)	4.2 (0.6-8.4)	1470 (211-2980)	369.7 (53.0-749.2)
Other enterococci	Vancomycin	9 (5-15)	2.2 (1.3-3.7)	784 (442-1300)	197.5 (111.2-327.8)	2 (1-3)	0.4 (0.2-0.8)	135 (64-267)	34.1 (16.2-67.3)
Other enterococci	Resistance to one or more antibiotics	87 (49-143)	21.8 (12.3-36.0)	7700 (4340-12700)	1939.4 (1093.0-3198.8)	18 (5-35)	4.5 (1.2-8.9)	1600 (413-3130)	403.8 (104.0-787.7)
Escherichia coli	Aminoglycosides	220 (156-313)	55.4 (39.2-78.8)	19600 (13900-27900)	4937.8 (3494.0-7013.4)	13 (8-19)	3.2 (2.1-4.8)	1150 (744-1680)	288.6 (187.3-423.6)
Escherichia coli	Aminopenicillin	735 (527-1030)	185.0 (132.6-258.6)	65500 (46900-91500)	16484.2 (11807.6-23036.9)	9 (6-14)	2.4 (1.5-3.5)	836 (542-1240)	210.5 (136.4-312.6)
Escherichia coli	Beta Lactam/Beta-lactamase inhibitors	578 (413-811)	145.6 (104.0-204.2)	51500 (36800-72300)	12978.4 (9259.3-18201.5)	23 (15-33)	5.8 (3.8-8.3)	2040 (1350-2950)	514.4 (340.2-742.0)
Escherichia coli	Carbapenem	75 (49-111)	18.8 (12.3-27.9)	6670 (4360-9840)	1678.6 (1097.3-2478.5)	16 (8-27)	4.0 (2.1-6.7)	1430 (756-2360)	358.8 (190.4-594.1)
Escherichia coli	Fluoroquinolones	475 (337-671)	119.6 (84.8-169.0)	42300 (30000-59800)	10654.7 (7556.7-15058.9)	49 (33-69)	12.2 (8.3-17.4)	4320 (2940-6140)	1088.9 (740.4-1546.7)
Escherichia coli	Third-generation cephalosporins	471 (335-668)	118.7 (84.3-168.1)	42000 (29800-59600)	10574.6 (7508.5-14994.6)	64 (28-117)	16.2 (7.1-29.5)	5740 (2520-10400)	1445.0 (634.7-2626.9)
Escherichia coli	Trimethoprim-Sulfamethoxazole	545 (391-769)	137.3 (98.4-193.5)	48600 (34800-68500)	12238.1 (8763.0-17257.0)	31 (20-46)	7.8 (5.1-11.6)	2770 (1820-4090)	698.2 (457.7-1030.0)
Escherichia coli	Resistance to one or more antibiotics	803 (578-1120)	202.3 (145.5-282.8)	71600 (51500-100000)	18026.2 (12956.2-25200.3)	205 (137-300)	51.7 (34.4-75.6)	18300 (12200-26700)	4604.5 (3067.8-6726.8)
Group A Streptococcus	Macrolide	14 (8-23)	3.5 (2.1-5.7)	1240 (745-2010)	311.3 (187.7-507.1)	1 (0-4)	0.3 (0.0-1.1)	118 (0-384)	29.8 (0.0-96.8)
Group A Streptococcus	Resistance to one or more antibiotics	14 (8-23)	3.5 (2.1-5.7)	1240 (745-2010)	311.3 (187.7-507.1)	1 (0-4)	0.3 (0.0-1.1)	118 (0-384)	29.8 (0.0-96.8)
Group B Streptococcus	Fluoroquinolones	94 (61-138)	23.7 (15.4-34.8)	8380 (5440-12300)	2109.4 (1370.1-3096.4)	16 (3-37)	4.1 (0.7-9.2)	1440 (253-3250)	361.5 (63.8-817.2)
Group B Streptococcus	Macrolide	249 (169-359)	62.6 (42.6-90.5)	22100 (15100-32000)	5566.5 (3790.7-8049.6)	24 (0-67)	6.1 (0.0-16.7)	2170 (0-5910)	545.5 (0.0-1488.7)
Group B Streptococcus	Penicillin	19 (13-29)	4.9 (3.2-7.4)	1730 (1130-2600)	436.1 (283.3-654.8)	4 (1-8)	0.9 (0.2-1.9)	315 (61-682)	79.3 (15.3-171.8)
Group B Streptococcus	Resistance to one or more antibiotics	297 (205-420)	74.9 (51.5-105.8)	26500 (18200-37400)	6661.9 (4582.0-9418.2)	44 (7-92)	11.1 (1.8-23.2)	3920 (641-8190)	986.3 (161.3-2062.5)
Haemophilus influenzae	Aminopenicillin	23 (15-36)	5.9 (3.7-9.0)	2080 (1320-3190)	523.2 (332.5-802.4)	3 (0-7)	0.8 (0.1-1.8)	299 (25-643)	75.4 (6.4-161.9)
Haemophilus influenzae	Third-generation cephalosporins	16 (10-24)	4.1 (2.6-6.0)	1440 (924-2130)	363.8 (232.6-535.7)	5 (2-9)	1.2 (0.5-2.2)	423 (169-766)	106.5 (42.5-192.9)
Haemophilus influenzae	Resistance to one or more antibiotics	34 (23-51)	8.6 (5.7-12.9)	3040 (2010-4550)	765.0 (505.1-1145.5)	8 (3-14)	2.0 (0.9-3.5)	722 (307-1250)	181.9 (77.3-313.8)
Klebsiella pneumoniae	Aminoglycosides	413 (290-586)	104.0 (73.1-147.5)	36700 (25800-52100)	9250.5 (6500.0-13119.5)	31 (19-48)	7.9 (4.7-12.1)	2790 (1660-4260)	701.9 (418.3-1073.5)
Klebsiella pneumoniae	Beta Lactam/Beta-lactamase inhibitors	792 (555-1110)	199.5 (139.8-278.2)	70500 (49400-98300)	17741.1 (12431.8-24748.4)	10 (4-21)	2.6 (0.9-5.2)	913 (315-1840)	229.8 (79.4-463.4)
Klebsiella pneumoniae	Carbapenem	204 (126-329)	51.5 (31.7-83.0)	18200 (11200-29300)	4577.0 (2816.5-7375.6)	47 (27-76)	11.8 (6.7-19.1)	4170 (2380-6740)	1050.2 (599.1-1698.0)
Klebsiella pneumoniae	Fluoroquinolones	558 (394-782)	140.4 (99.1-196.8)	49600 (35000-69500)	12491.4 (8817.7-17504.8)	38 (23-58)	9.5 (5.7-14.7)	3360 (2010-5190)	845.7 (506.0-1307.8)
Klebsiella pneumoniae	Third-generation cephalosporins	778 (524-1120)	195.9 (131.9-282.6)	69200 (46600-99900)	17420.7 (11728.5-25142.2)	96 (35-178)	24.2 (8.8-44.9)	8540 (3120-15900)	2149.9 (785.7-3994.6)
Klebsiella pneumoniae	Trimethoprim-Sulfamethoxazole	780 (547-1100)	196.4 (137.8-278.0)	69400 (48700-98200)	17466.7 (12256.7-24726.1)	40 (19-67)	10.0 (4.8-16.9)	3520 (1680-5980)	886.7 (422.5-1504.6)
Klebsiella pneumoniae	Resistance to one or more antibiotics	923 (647-1290)	232.3 (162.9-325.4)	82100 (57600-115000)	20663.4 (14490.8-28939.6)	262 (164-385)	65.9 (41.2-97.0)	23300 (14600-34300)	5864.3 (3664.3-8625.1)
Morganella spp.	Fluoroquinolones	0 (0-0)	0.0 (0.0-0.0)	3 (1-9)	0.8 (0.2-2.3)	0 (0-0)	0.0 (0.0-0.0)	1 (0-2)	0.1 (0.0-0.4)
Morganella spp.	Fourth-generation cephalosporins	0 (0-0)	0.0 (0.0-0.0)	1 (0-2)	0.2 (0.1-0.6)	0 (0-0)	0.0 (0.0-0.0)	0 (0-1)	0.0 (0.0-0.1)
Morganella spp.	Third-generation cephalosporins	0 (0-0)	0.0 (0.0-0.0)	2 (1-7)	0.6 (0.2-1.8)	0 (0-0)	0.0 (0.0-0.0)	0 (0-1)	0.1 (0.0-0.3)
Morganella spp.	Resistance to one or more antibiotics	0 (0-0)	0.0 (0.0-0.0)	4 (1-13)	1.1 (0.3-3.3)	0 (0-0)	0.0 (0.0-0.0)	1 (0-3)	0.3 (0.1-0.8)
Mycobacterium tuberculosis	Extensive drug resistance in TB	--	--	0 (0-0)	0.0 (0.0-0.0)	--	--	0 (0-0)	0.0 (0.0-0.0)
Mycobacterium tuberculosis	Isoniazid mono-resistance	--	--	0 (0-0)	0.0 (0.0-0.0)	--	--	0 (0-0)	0.0 (0.0-0.0)
Mycobacterium tuberculosis	Multi-drug resistance excluding extensive drug resistance in TB	--	--	0 (0-0)	0.0 (0.0-0.0)	--	--	0 (0-0)	0.0 (0.0-0.0)
Mycobacterium tuberculosis	Rifampicin mono-resistance	--	--	0 (0-0)	0.0 (0.0-0.0)	--	--	0 (0-0)	0.0 (0.0-0.0)
Mycobacterium tuberculosis	Resistance to one or more antibiotics	--	--	0 (0-0)	0.0 (0.0-0.0)	--	--	0 (0-0)	0.0 (0.0-0.0)
Neisseria gonorrhoeae	Fluoroquinolones	--	--	--	--	--	--	--	--
Neisseria gonorrhoeae	Third-generation cephalosporins	--	--	--	--	--	--	--	--
Neisseria gonorrhoeae	Resistance to one or more antibiotics	--	--	--	--	--	--	--	--
Proteus spp.	Aminoglycosides	8 (5-12)	1.9 (1.2-2.9)	681 (429-1040)	171.4 (108.1-261.8)	0 (0-1)	0.1 (0.0-0.2)	35 (4-78)	8.8 (1.0-19.7)
Proteus spp.	Aminopenicillin	23 (14-36)	5.8 (3.6-9.2)	2070 (1270-3250)	520.3 (319.6-818.1)	0 (0-2)	0.1 (0.0-0.4)	43 (0-134)	10.7 (0.0-33.7)
Proteus spp.	Fluoroquinolones	8 (5-13)	2.1 (1.2-3.3)	731 (437-1180)	184.2 (110.0-296.5)	1 (0-1)	0.1 (0.1-0.3)	48 (18-98)	12.2 (4.6-24.8)
Proteus spp.	Third-generation cephalosporins	7 (4-11)	1.7 (1.0-2.9)	611 (344-1010)	153.8 (86.7-255.2)	1 (0-3)	0.4 (0.1-0.8)	132 (40-274)	33.1 (10.2-69.1)
Proteus spp.	Trimethoprim-Sulfamethoxazole	14 (8-22)	3.4 (2.1-5.5)	1220 (733-1950)	307.4 (184.5-490.2)	0 (0-1)	0.1 (0.0-0.3)	43 (0-102)	10.9 (0.0-25.6)

Pathogen	Antibiotic Class	Post Neonatal							
		Associated with resistance				Attributable to resistance			
		Deaths		DALYs		Deaths		DALYs	
		Counts	Rate per 100 000	Counts	Rate per 100 000	Counts	Rate per 100 000	Counts	Rate per 100 000
All pathogens	Resistance to one or more antibiotics	4460 (3580-5680)	92.2 (74.0-117.4)	396000 (317000-503000)	8179.6 (6564.9-10400.6)	1060 (809-1400)	22.0 (16.7-28.9)	94200 (71800-124000)	1948.6 (1485.4-2559.7)
Acinetobacter baumannii	Aminoglycosides	75 (48-112)	1.6 (1.0-2.3)	6640 (4280-9870)	137.3 (88.6-204.2)	3 (0-8)	0.1 (0.0-0.2)	277 (0-663)	5.7 (0.0-13.7)
Acinetobacter baumannii	Anti-pseudomonal penicillin/Beta-Lactamase inhibitors	97 (62-144)	2.0 (1.3-3.0)	8620 (5540-12700)	178.3 (114.6-262.4)	3 (2-6)	0.1 (0.0-0.1)	308 (152-531)	6.4 (3.1-11.0)
Acinetobacter baumannii	Beta Lactam/Beta-lactamase inhibitors	87 (55-127)	1.8 (1.1-2.6)	7700 (4910-11200)	159.3 (101.5-231.9)	0 (0-0)	0.0 (0.0-0.0)	4 (1-8)	0.1 (0.0-0.2)
Acinetobacter baumannii	Carbapenem	81 (51-119)	1.7 (1.1-2.5)	7150 (4530-10500)	147.8 (93.7-218.1)	13 (7-22)	0.3 (0.1-0.4)	1150 (622-1920)	23.8 (12.9-39.8)
Acinetobacter baumannii	Fluoroquinolones	92 (58-135)	1.9 (1.2-2.8)	8110 (5160-11900)	167.7 (106.7-247.0)	12 (6-19)	0.2 (0.1-0.4)	1030 (536-1710)	21.3 (11.1-35.5)
Acinetobacter baumannii	Fourth-generation cephalosporins	94 (60-138)	1.9 (1.2-2.9)	8310 (5320-12200)	171.8 (110.1-252.9)	0 (0-0)	0.0 (0.0-0.0)	8 (3-16)	0.2 (0.1-0.3)
Acinetobacter baumannii	Third-generation cephalosporins	95 (61-141)	2.0 (1.3-2.9)	8440 (5400-12500)	174.6 (111.7-258.3)	0 (0-1)	0.0 (0.0-0.0)	28 (10-56)	0.6 (0.2-1.2)
Acinetobacter baumannii	Resistance to one or more antibiotics	99 (64-146)	2.1 (1.3-3.0)	8800 (5640-12900)	181.9 (116.5-267.4)	32 (20-50)	0.7 (0.4-1.0)	2810 (1730-4420)	58.0 (35.7-91.4)
Citrobacter spp.	Aminoglycosides	3 (2-6)	0.1 (0.0-0.1)	295 (147-520)	6.1 (3.0-10.8)	0 (0-1)	0.0 (0.0-0.0)	16 (0-46)	0.3 (0.0-1.0)
Citrobacter spp.	Anti-pseudomonal penicillin/Beta-Lactamase inhibitors	7 (3-13)	0.1 (0.1-0.3)	597 (286-1130)	12.4 (5.9-23.3)	1 (0-3)	0.0 (0.0-0.1)	114 (42-245)	2.4 (0.9-5.1)
Citrobacter spp.	Carbapenem	6 (3-11)	0.1 (0.1-0.2)	544 (276-955)	11.2 (5.7-19.8)	2 (1-3)	0.0 (0.0-0.1)	135 (57-264)	2.8 (1.2-5.5)
Citrobacter spp.	Fluoroquinolones	9 (4-15)	0.2 (0.1-0.3)	753 (384-1340)	15.6 (7.9-27.7)	1 (1-3)	0.0 (0.0-0.1)	118 (45-242)	2.4 (0.9-5.0)
Citrobacter spp.	Fourth-generation cephalosporins	5 (2-9)	0.1 (0.0-0.2)	411 (197-760)	8.5 (4.1-15.7)	0 (0-1)	0.0 (0.0-0.0)	22 (8-47)	0.5 (0.2-1.0)
Citrobacter spp.	Third-generation cephalosporins	15 (7-28)	0.3 (0.2-0.6)	1310 (643-2470)	27.2 (13.3-51.1)	2 (1-3)	0.0 (0.0-0.1)	139 (47-304)	2.9 (1.0-6.3)
Citrobacter spp.	Resistance to one or more antibiotics	21 (11-38)	0.4 (0.2-0.8)	1850 (936-3350)	38.3 (19.3-69.2)	6 (3-12)	0.1 (0.1-0.2)	544 (260-1020)	11.3 (5.4-21.2)
Enterobacter spp.	Aminoglycosides	15 (10-22)	0.3 (0.2-0.5)	1360 (913-1990)	28.1 (18.9-41.1)	1 (0-2)	0.0 (0.0-0.0)	88 (24-171)	1.8 (0.5-3.5)
Enterobacter spp.	Anti-pseudomonal penicillin/Beta-Lactamase inhibitors	51 (36-71)	1.1 (0.8-1.5)	4540 (3220-6330)	93.8 (66.6-131.0)	7 (4-11)	0.1 (0.1-0.2)	610 (353-944)	12.6 (7.3-19.5)
Enterobacter spp.	Carbapenem	14 (10-20)	0.3 (0.2-0.4)	1210 (854-1750)	25.0 (17.7-36.3)	3 (2-5)	0.1 (0.0-0.1)	301 (185-460)	6.2 (3.8-9.5)
Enterobacter spp.	Fluoroquinolones	17 (11-25)	0.4 (0.2-0.5)	1540 (1010-2200)	31.8 (21.0-45.5)	2 (1-3)	0.0 (0.0-0.1)	159 (81-277)	3.3 (1.7-5.7)
Enterobacter spp.	Fourth-generation cephalosporins	45 (32-64)	0.9 (0.7-1.3)	4000 (2820-5640)	82.6 (58.3-116.5)	2 (1-4)	0.0 (0.0-0.1)	211 (106-367)	4.4 (2.2-7.6)
Enterobacter spp.	Trimethoprim-Sulfamethoxazole	46 (32-66)	1.0 (0.7-1.4)	4090 (2830-5820)	84.7 (58.6-120.4)	3 (0-6)	0.1 (0.0-0.1)	249 (0-535)	5.1 (0.0-11.1)
Enterobacter spp.	Resistance to one or more antibiotics	84 (60-115)	1.7 (1.2-2.4)	7400 (5300-10200)	153.0 (109.6-210.4)	18 (13-26)	0.4 (0.3-0.5)	1620 (1110-2330)	33.5 (23.0-48.1)
Enterococcus faecalis	Fluoroquinolones	21 (12-33)	0.4 (0.3-0.7)	1850 (1100-2990)	38.2 (22.8-61.8)	5 (2-9)	0.1 (0.0-0.2)	455 (202-843)	9.4 (4.2-17.4)
Enterococcus faecalis	Vancomycin	2 (1-3)	0.0 (0.0-0.1)	154 (90-259)	3.2 (1.9-5.4)	1 (0-1)	0.0 (0.0-0.0)	46 (20-93)	0.9 (0.4-1.9)
Enterococcus faecalis	Resistance to one or more antibiotics	21 (13-34)	0.4 (0.3-0.7)	1900 (1140-3070)	39.3 (23.5-63.4)	6 (3-10)	0.1 (0.1-0.2)	501 (248-899)	10.4 (5.1-18.6)
Enterococcus faecium	Fluoroquinolones	42 (25-69)	0.9 (0.5-1.4)	3760 (2210-6150)	77.7 (45.7-127.2)	8 (2-17)	0.2 (0.1-0.4)	733 (219-1540)	15.2 (4.5-31.8)
Enterococcus faecium	Vancomycin	10 (5-16)	0.2 (0.1-0.3)	856 (475-1450)	17.7 (9.8-30.0)	2 (1-4)	0.0 (0.0-0.1)	199 (90-391)	4.1 (1.9-8.1)
Enterococcus faecium	Resistance to one or more antibiotics	43 (25-69)	0.9 (0.5-1.4)	3770 (2220-6160)	78.0 (45.9-127.4)	11 (5-20)	0.2 (0.1-0.4)	932 (413-1810)	19.3 (8.5-37.4)
Other enterococci	Fluoroquinolones	24 (12-43)	0.5 (0.3-0.9)	2140 (1100-3780)	44.3 (22.7-78.2)	5 (1-10)	0.1 (0.0-0.2)	408 (60-877)	8.4 (1.2-18.1)
Other enterococci	Vancomycin	3 (1-5)	0.1 (0.0-0.1)	236 (119-425)	4.9 (2.5-8.8)	0 (0-1)	0.0 (0.0-0.0)	41 (17-85)	0.8 (0.4-1.8)
Other enterococci	Resistance to one or more antibiotics	24 (12-43)	0.5 (0.3-0.9)	2150 (1100-3790)	44.4 (22.8-78.5)	5 (1-10)	0.1 (0.0-0.2)	449 (107-922)	9.3 (2.2-19.1)
Escherichia coli	Aminoglycosides	107 (82-140)	2.2 (1.7-2.9)	9640 (7370-12600)	199.4 (152.3-261.2)	6 (4-9)	0.1 (0.1-0.2)	560 (390-783)	11.6 (8.1-16.2)
Escherichia coli	Aminopenicillin	355 (275-464)	7.4 (5.7-9.6)	32100 (24800-41800)	663.3 (511.9-864.5)	5 (3-7)	0.1 (0.1-0.1)	413 (284-592)	8.5 (5.9-12.2)
Escherichia coli	Beta Lactam/Beta-lactamase inhibitors	277 (214-363)	5.7 (4.4-7.5)	25000 (19400-32700)	517.4 (400.6-675.6)	11 (8-15)	0.2 (0.2-0.3)	972 (702-1330)	20.1 (14.5-27.6)
Escherichia coli	Carbapenem	39 (26-54)	0.8 (0.5-1.1)	3500 (2390-4890)	72.3 (49.4-101.1)	8 (5-13)	0.2 (0.1-0.3)	738 (404-1190)	15.3 (8.4-24.6)
Escherichia coli	Fluoroquinolones	235 (183-311)	4.9 (3.8-6.4)	21100 (16500-28000)	437.3 (341.3-578.6)	24 (18-33)	0.5 (0.4-0.7)	2160 (1580-2930)	44.7 (32.8-60.6)
Escherichia coli	Third-generation cephalosporins	230 (172-304)	4.8 (3.6-6.3)	20700 (15500-27500)	428.9 (320.3-569.6)	31 (14-53)	0.6 (0.3-1.1)	2790 (1260-4770)	57.7 (26.0-98.7)
Escherichia coli	Trimethoprim-Sulfamethoxazole	264 (205-350)	5.5 (4.2-7.2)	23800 (18400-31400)	492.9 (380.9-649.8)	15 (10-21)	0.3 (0.2-0.4)	1340 (921-1870)	27.7 (19.0-38.7)
Escherichia coli	Resistance to one or more antibiotics	389 (303-507)	8.0 (6.3-10.5)	35100 (27300-45800)	725.7 (563.8-946.1)	100 (70-138)	2.1 (1.4-2.9)	8980 (6320-12300)	185.6 (130.7-254.9)
Group A Streptococcus	Macrolide	12 (7-19)	0.2 (0.2-0.4)	1080 (669-1680)	22.2 (13.8-34.8)	1 (0-4)	0.0 (0.0-0.1)	103 (0-335)	2.1 (0.0-6.9)
Group A Streptococcus	Resistance to one or more antibiotics	12 (7-19)	0.2 (0.2-0.4)	1080 (669-1680)	22.2 (13.8-34.8)	1 (0-4)	0.0 (0.0-0.1)	103 (0-335)	2.1 (0.0-6.9)
Group B Streptococcus	Fluoroquinolones	27 (20-38)	0.6 (0.4-0.8)	2430 (1770-3360)	50.3 (36.6-69.5)	5 (1-10)	0.1 (0.0-0.2)	413 (69-928)	8.6 (1.4-19.2)
Group B Streptococcus	Macrolide	82 (60-109)	1.7 (1.2-2.2)	7280 (5280-9620)	150.5 (109.3-199.0)	8 (0-21)	0.2 (0.0-0.4)	720 (0-1890)	14.9 (0.0-39.1)
Group B Streptococcus	Penicillin	6 (4-8)	0.1 (0.1-0.2)	503 (347-706)	10.4 (7.2-14.6)	1 (0-2)	0.0 (0.0-0.0)	93 (18-201)	1.9 (0.4-4.1)
Group B Streptococcus	Resistance to one or more antibiotics	96 (72-126)	2.0 (1.5-2.6)	8510 (6410-11200)	176.0 (132.5-231.1)	14 (2-28)	0.3 (0.0-0.6)	1230 (176-2490)	25.4 (3.6-51.4)
Haemophilus influenzae	Aminopenicillin	42 (30-58)	0.9 (0.6-1.2)	3710 (2660-5140)	76.7 (54.9-106.2)	6 (0-13)	0.1 (0.0-0.3)	533 (40-1120)	11.0 (0.8-23.2)
Haemophilus influenzae	Third-generation cephalosporins	31 (22-41)	0.6 (0.5-0.8)	2710 (1980-3610)	56.0 (40.9-74.6)	9 (4-16)	0.2 (0.1-0.3)	793 (335-1370)	16.4 (6.9-28.4)
Haemophilus influenzae	Resistance to one or more antibiotics	63 (48-82)	1.3 (1.0-1.7)	5530 (4220-7280)	114.4 (87.3-150.5)	15 (7-24)	0.3 (0.1-0.5)	1330 (629-2160)	27.4 (13.0-44.8)
Klebsiella pneumoniae	Aminoglycosides	292 (225-386)	6.0 (4.7-8.0)	25800 (19900-34100)	534.1 (412.1-705.9)	22 (14-32)	0.5 (0.3-0.7)	1960 (1280-2840)	40.6 (26.4-58.6)
Klebsiella pneumoniae	Beta Lactam/Beta-lactamase inhibitors	563 (439-721)	11.7 (9.1-14.9)	49900 (38900-63800)	1031.0 (804.2-1319.2)	7 (2-15)	0.2 (0.0-0.3)	644 (213-1290)	13.3 (4.4-26.6)
Klebsiella pneumoniae	Carbapenem	142 (89-229)	2.9 (1.8-4.7)	12600 (7890-20300)	260.3 (163.2-419.0)	33 (19-53)	0.7 (0.4-1.1)	2890 (1700-4670)	59.7 (35.2-96.6)
Klebsiella pneumoniae	Fluoroquinolones	395 (309-508)	8.2 (6.4-10.5)	34900 (27300-45000)	722.3 (565.5-930.1)	27 (17-39)	0.6 (0.3-0.8)	2370 (1490-3440)	48.9 (30.7-71.2)
Klebsiella pneumoniae	Third-generation cephalosporins	555 (412-736)	11.5 (8.5-15.2)	49100 (36400-65100)	1015.9 (753.6-1345.9)	69 (27-127)	1.4 (0.6-2.6)	6130 (2390-11300)	126.7 (49.4-232.8)
Klebsiella pneumoniae	Trimethoprim-Sulfamethoxazole	557 (435-717)	11.5 (9.0-14.8)	49300 (38500-63400)	1018.5 (796.4-1311.2)	28 (14-45)	0.6 (0.3-0.9)	2510 (1210-3980)	51.8 (25.1-82.2)
Klebsiella pneumoniae	Resistance to one or more antibiotics	658 (518-845)	13.6 (10.7-17.5)	58300 (45900-74800)	1204.7 (948.5-1546.1)	186 (127-268)	3.9 (2.6-5.5)	16500 (11200-23700)	341.2 (232.5-489.9)
Morganella spp.	Fluoroquinolones	0 (0-0)	0.0 (0.0-0.0)	7 (3-16)	0.2 (0.1-0.3)	0 (0-0)	0.0 (0.0-0.0)	1 (0-3)	0.0 (0.0-0.1)
Morganella spp.	Fourth-generation cephalosporins	0 (0-0)	0.0 (0.0-0.0)	2 (1-4)	0.0 (0.0-0.1)	0 (0-0)	0.0 (0.0-0.0)	0 (0-1)	0.0 (0.0-0.0)
Morganella spp.	Third-generation cephalosporins	0 (0-0)	0.0 (0.0-0.0)	7 (3-15)	0.1 (0.1-0.3)	0 (0-0)	0.0 (0.0-0.0)	1 (0-3)	0.0 (0.0-0.1)
Morganella spp.	Resistance to one or more antibiotics	0 (0-0)	0.0 (0.0-0.0)	11 (5-24)	0.2 (0.1-0.5)	0 (0-0)	0.0 (0.0-0.0)	3 (1-7)	0.1 (0.0-0.1)
Mycobacterium tuberculosis	Extensive drug resistance in TB	26 (14-43)	0.5 (0.3-0.9)	2340 (1230-3850)	48.4 (25.3-79.6)	16 (8-27)	0.3 (0.2-0.6)	1430 (747-2360)	29.6 (15.5-48.8)
Mycobacterium tuberculosis	Isoniazid mono-resistance	21 (12-33)	0.4 (0.3-0.7)	1850 (1090-2910)	38.3 (22.4-60.2)	3 (0-11)	0.1 (0.0-0.2)	288 (0-1010)	6.0 (0.0-20.9)
Mycobacterium tuberculosis	Multi-drug resistance excluding extensive drug resistance in TB	58 (31-92)	1.2 (0.6-1.9)	5120 (2770-8180)	105.8 (57.4-169.1)	33 (5-70)	0.7 (0.1-1.4)	2930 (455-6140)	60.7 (9.4-127.0)
Mycobacterium tuberculosis	Rifampicin mono-resistance	13 (8-20)	0.3 (0.2-0.4)	1160 (695-1800)	23.9 (14.4-37.2)	4 (1-8)	0.1 (0.0-0.2)	322 (50-701)	6.7 (1.0-14.5)
Mycobacterium tuberculosis	Resistance to one or more antibiotics	118 (82-166)	2.4 (1.7-3.4)	10500 (7310-14700)	216.4 (151.2-304.3)	56 (21-101)	1.2 (0.4-2.1)	4980 (1870-8900)	102.9 (38.7-184.0)
Neisseria gonorrhoeae	Fluoroquinolones	--	--	--	--	--	--	--	--
Neisseria gonorrhoeae	Third-generation cephalosporins	--	--	--	--	--	--	--	--
Neisseria gonorrhoeae	Resistance to one or more antibiotics	--	--	--	--	--	--	--	--
Proteus spp.	Aminoglycosides	4 (2-6)	0.1 (0.0-0.1)	343 (208-546)	7.1 (4.3-11.3)	0 (0-0)	0.0 (0.0-0.0)	18 (2-40)	0.4 (0.0-0.8)
Proteus spp.	Aminopenicillin	12 (7-18)	0.2 (0.1-0.4)	1050 (633-1660)	21.6 (13.1-34.4)	0 (0-1)	0.0 (0.0-0.0)	22 (0-70)	0.4 (0.0-1.4)
Proteus spp.	Fluoroquinolones	4 (2-7)	0.1 (0.1-0.1)	371 (220-592)	7.7 (4.6-12.2)	0 (0-1)	0.0 (0.0-0.0)	24 (9-51)	0.5 (0.2-1.1)
Proteus spp.	Third-generation cephalosporins	3 (2-5)	0.1 (0.0-0.1)	302 (178-486)	6.2 (3.7-10.0)	1 (0-1)	0.0 (0.0-0.0)	65 (21-129)	1.3 (0.4-2.7)
Proteus spp.	Trimethoprim-Sulfamethoxazole	7 (4-11)	0.1 (0.1-0.2)	608 (365-971)	12.6 (7.6-20.1)	0 (0-1)	0.0 (0.0-0.0)	22 (0-49)	0.4 (0.0-1.0)

Pathogen	Antibiotic Class	1 to 4							
		Associated with resistance				Attributable to resistance			
		Deaths		DALYs		Deaths		DALYs	
		Counts	Rate per 100 000	Counts	Rate per 100 000	Counts	Rate per 100 000	Counts	Rate per 100 000
All pathogens	Resistance to one or more antibiotics	1840 (1480-2320)	8.3 (6.6-10.4)	162000 (130000-204000)	725.1 (582.6-913.9)	443 (337-572)	2.0 (1.5-2.6)	38600 (29500-49800)	173.0 (131.9-223.0)
Acinetobacter baumannii	Aminoglycosides	27 (16-43)	0.1 (0.1-0.2)	2340 (1410-3690)	10.5 (6.3-16.5)	1 (0-3)	0.0 (0.0-0.0)	98 (1-241)	0.4 (0.0-1.1)
Acinetobacter baumannii	Anti-pseudomonal penicillin/Beta-Lactamase inhibitors	35 (21-54)	0.2 (0.1-0.2)	2990 (1820-4700)	13.4 (8.2-21.1)	1 (1-2)	0.0 (0.0-0.0)	103 (50-182)	0.5 (0.2-0.8)
Acinetobacter baumannii	Beta Lactam/Beta-lactamase inhibitors	31 (19-48)	0.1 (0.1-0.2)	2670 (1630-4160)	12.0 (7.3-18.6)	0 (0-0)	0.0 (0.0-0.0)	2 (0-4)	0.0 (0.0-0.0)
Acinetobacter baumannii	Carbapenem	29 (18-46)	0.1 (0.1-0.2)	2500 (1540-3940)	11.2 (6.9-17.6)	5 (2-8)	0.0 (0.0-0.0)	403 (213-698)	1.8 (1.0-3.1)
Acinetobacter baumannii	Fluoroquinolones	33 (20-52)	0.1 (0.1-0.2)	2810 (1710-4470)	12.6 (7.7-20.0)	4 (2-7)	0.0 (0.0-0.0)	355 (181-608)	1.6 (0.8-2.7)
Acinetobacter baumannii	Fourth-generation cephalosporins	33 (20-52)	0.1 (0.1-0.2)	2880 (1750-4520)	12.9 (7.8-20.2)	0 (0-0)	0.0 (0.0-0.0)	3 (1-6)	0.0 (0.0-0.0)
Acinetobacter baumannii	Third-generation cephalosporins	34 (21-53)	0.2 (0.1-0.2)	2940 (1790-4610)	13.1 (8.0-20.6)	0 (0-0)	0.0 (0.0-0.0)	11 (4-22)	0.0 (0.0-0.1)
Acinetobacter baumannii	Resistance to one or more antibiotics	35 (22-56)	0.2 (0.1-0.2)	3060 (1870-4810)	13.7 (8.4-21.6)	11 (7-18)	0.1 (0.0-0.1)	975 (570-1560)	4.4 (2.6-7.0)
Citrobacter spp.	Aminoglycosides	1 (0-2)	0.0 (0.0-0.0)	81 (37-153)	0.4 (0.2-0.7)	0 (0-0)	0.0 (0.0-0.0)	5 (0-13)	0.0 (0.0-0.1)
Citrobacter spp.	Anti-pseudomonal penicillin/Beta-Lactamase inhibitors	2 (1-5)	0.0 (0.0-0.0)	209 (82-444)	0.9 (0.4-2.0)	0 (0-1)	0.0 (0.0-0.0)	42 (14-103)	0.2 (0.1-0.5)
Citrobacter spp.	Carbapenem	2 (1-3)	0.0 (0.0-0.0)	148 (66-280)	0.7 (0.3-1.3)	0 (0-1)	0.0 (0.0-0.0)	37 (14-80)	0.2 (0.1-0.4)
Citrobacter spp.	Fluoroquinolones	3 (1-5)	0.0 (0.0-0.0)	224 (100-437)	1.0 (0.4-2.0)	0 (0-1)	0.0 (0.0-0.0)	36 (12-77)	0.2 (0.1-0.3)
Citrobacter spp.	Fourth-generation cephalosporins	1 (1-3)	0.0 (0.0-0.0)	117 (49-233)	0.5 (0.2-1.0)	0 (0-0)	0.0 (0.0-0.0)	7 (2-15)	0.0 (0.0-0.1)
Citrobacter spp.	Third-generation cephalosporins	5 (2-10)	0.0 (0.0-0.0)	413 (169-846)	1.8 (0.8-3.8)	0 (0-1)	0.0 (0.0-0.0)	43 (13-102)	0.2 (0.1-0.5)
Citrobacter spp.	Resistance to one or more antibiotics	7 (3-13)	0.0 (0.0-0.1)	578 (248-1160)	2.6 (1.1-5.2)	2 (1-4)	0.0 (0.0-0.0)	169 (68-350)	0.8 (0.3-1.6)
Enterobacter spp.	Aminoglycosides	6 (4-9)	0.0 (0.0-0.0)	520 (353-753)	2.3 (1.6-3.4)	0 (0-1)	0.0 (0.0-0.0)	34 (9-66)	0.2 (0.0-0.3)
Enterobacter spp.	Anti-pseudomonal penicillin/Beta-Lactamase inhibitors	21 (14-29)	0.1 (0.1-0.1)	1790 (1250-2550)	8.0 (5.6-11.4)	3 (2-4)	0.0 (0.0-0.0)	243 (136-379)	1.1 (0.6-1.7)
Enterobacter spp.	Carbapenem	5 (4-8)	0.0 (0.0-0.0)	475 (328-678)	2.1 (1.5-3.0)	1 (1-2)	0.0 (0.0-0.0)	118 (72-181)	0.5 (0.3-0.8)
Enterobacter spp.	Fluoroquinolones	7 (5-10)	0.0 (0.0-0.0)	610 (398-897)	2.7 (1.8-4.0)	1 (0-1)	0.0 (0.0-0.0)	63 (31-110)	0.3 (0.1-0.5)
Enterobacter spp.	Fourth-generation cephalosporins	18 (13-26)	0.1 (0.1-0.1)	1590 (1110-2240)	7.1 (5.0-10.0)	1 (0-2)	0.0 (0.0-0.0)	85 (43-149)	0.4 (0.2-0.7)
Enterobacter spp.	Trimethoprim-Sulfamethoxazole	18 (13-26)	0.1 (0.1-0.1)	1580 (1110-2240)	7.1 (5.0-10.0)	1 (0-2)	0.0 (0.0-0.0)	96 (0-206)	0.4 (0.0-0.9)
Enterobacter spp.	Resistance to one or more antibiotics	33 (23-47)	0.1 (0.1-0.2)	2910 (2050-4090)	13.0 (9.2-18.3)	7 (5-11)	0.0 (0.0-0.0)	638 (428-924)	2.9 (1.9-4.1)
Enterococcus faecalis	Fluoroquinolones	10 (6-17)	0.0 (0.0-0.1)	992 (591-1610)	4.4 (2.6-7.2)	3 (1-5)	0.0 (0.0-0.0)	231 (104-426)	1.0 (0.5-1.9)
Enterococcus faecalis	Vancomycin	1 (0-2)	0.0 (0.0-0.0)	83 (48-142)	0.4 (0.2-0.6)	0 (0-1)	0.0 (0.0-0.0)	25 (11-48)	0.1 (0.0-0.2)
Enterococcus faecalis	Resistance to one or more antibiotics	11 (6-18)	0.0 (0.0-0.1)	1020 (607-1640)	4.6 (2.7-7.4)	3 (1-5)	0.0 (0.0-0.0)	256 (122-461)	1.1 (0.5-2.1)
Enterococcus faecium	Fluoroquinolones	19 (10-34)	0.1 (0.0-0.2)	1660 (842-2930)	7.4 (3.8-13.1)	4 (1-8)	0.0 (0.0-0.0)	323 (89-726)	1.4 (0.4-3.3)
Enterococcus faecium	Vancomycin	4 (2-8)	0.0 (0.0-0.0)	377 (188-688)	1.7 (0.8-3.1)	1 (0-2)	0.0 (0.0-0.0)	88 (35-180)	0.4 (0.2-0.8)
Enterococcus faecium	Resistance to one or more antibiotics	19 (10-34)	0.1 (0.0-0.2)	1660 (844-2930)	7.4 (3.8-13.1)	5 (2-10)	0.0 (0.0-0.0)	411 (169-864)	1.8 (0.8-3.9)
Other enterococci	Fluoroquinolones	9 (4-18)	0.0 (0.0-0.1)	839 (432-1590)	3.8 (1.9-7.1)	2 (0-4)	0.0 (0.0-0.0)	155 (25-346)	0.7 (0.1-1.5)
Other enterococci	Vancomycin	1 (0-2)	0.0 (0.0-0.0)	102 (50-199)	0.5 (0.2-0.9)	0 (0-0)	0.0 (0.0-0.0)	19 (8-40)	0.1 (0.0-0.2)
Other enterococci	Resistance to one or more antibiotics	9 (4-18)	0.0 (0.0-0.1)	843 (435-1600)	3.8 (1.9-7.2)	2 (0-4)	0.0 (0.0-0.0)	173 (47-372)	0.8 (0.2-1.7)
Escherichia coli	Aminoglycosides	48 (36-64)	0.2 (0.2-0.3)	4530 (3460-5980)	20.3 (15.5-26.8)	3 (2-4)	0.0 (0.0-0.0)	261 (180-360)	1.2 (0.8-1.6)
Escherichia coli	Aminopenicillin	160 (122-211)	0.7 (0.5-0.9)	15100 (11600-19600)	67.7 (51.8-87.9)	2 (1-3)	0.0 (0.0-0.0)	192 (132-272)	0.9 (0.6-1.2)
Escherichia coli	Beta Lactam/Beta-lactamase inhibitors	125 (96-164)	0.6 (0.4-0.7)	11800 (8980-15400)	52.9 (40.2-69.0)	5 (3-7)	0.0 (0.0-0.0)	458 (325-626)	2.1 (1.5-2.8)
Escherichia coli	Carbapenem	18 (12-24)	0.1 (0.1-0.1)	1650 (1140-2270)	7.4 (5.1-10.2)	4 (2-6)	0.0 (0.0-0.0)	343 (199-542)	1.5 (0.9-2.4)
Escherichia coli	Fluoroquinolones	106 (81-138)	0.5 (0.4-0.6)	9910 (7610-12900)	44.4 (34.1-57.9)	11 (8-15)	0.0 (0.0-0.1)	1000 (736-1350)	4.5 (3.3-6.0)
Escherichia coli	Third-generation cephalosporins	104 (78-136)	0.5 (0.3-0.6)	9800 (7360-12900)	43.9 (33.0-58.0)	14 (6-25)	0.1 (0.0-0.1)	1320 (627-2260)	5.9 (2.8-10.1)
Escherichia coli	Trimethoprim-Sulfamethoxazole	120 (92-158)	0.5 (0.4-0.7)	11200 (8590-14700)	50.3 (38.5-65.9)	7 (5-9)	0.0 (0.0-0.0)	612 (412-864)	2.7 (1.8-3.9)
Escherichia coli	Resistance to one or more antibiotics	176 (136-232)	0.8 (0.6-1.0)	16600 (12800-21700)	74.3 (57.3-97.3)	45 (32-63)	0.2 (0.1-0.3)	4190 (3000-5780)	18.8 (13.4-25.9)
Group A Streptococcus	Macrolide	4 (2-8)	0.0 (0.0-0.0)	428 (223-751)	1.9 (1.0-3.4)	0 (0-1)	0.0 (0.0-0.0)	41 (0-134)	0.2 (0.0-0.6)
Group A Streptococcus	Resistance to one or more antibiotics	4 (2-8)	0.0 (0.0-0.0)	428 (223-751)	1.9 (1.0-3.4)	0 (0-1)	0.0 (0.0-0.0)	41 (0-134)	0.2 (0.0-0.6)
Group B Streptococcus	Fluoroquinolones	11 (8-16)	0.1 (0.0-0.1)	991 (710-1390)	4.4 (3.2-6.2)	2 (0-4)	0.0 (0.0-0.0)	167 (28-370)	0.7 (0.1-1.7)
Group B Streptococcus	Macrolide	35 (25-47)	0.2 (0.1-0.2)	3040 (2210-4060)	13.6 (9.9-18.2)	3 (0-9)	0.0 (0.0-0.0)	301 (0-793)	1.3 (0.0-3.6)
Group B Streptococcus	Penicillin	2 (2-3)	0.0 (0.0-0.0)	204 (140-283)	0.9 (0.6-1.3)	0 (0-1)	0.0 (0.0-0.0)	37 (8-81)	0.2 (0.0-0.4)
Group B Streptococcus	Resistance to one or more antibiotics	40 (30-54)	0.2 (0.1-0.2)	3540 (2630-4720)	15.9 (11.8-21.2)	6 (1-12)	0.0 (0.0-0.1)	505 (75-1020)	2.3 (0.3-4.6)
Haemophilus influenzae	Aminopenicillin	18 (13-24)	0.1 (0.1-0.1)	1530 (1120-2100)	6.8 (5.0-9.4)	3 (0-5)	0.0 (0.0-0.0)	221 (19-468)	1.0 (0.1-2.1)
Haemophilus influenzae	Third-generation cephalosporins	12 (9-16)	0.1 (0.0-0.1)	1070 (791-1410)	4.8 (3.5-6.3)	4 (2-6)	0.0 (0.0-0.0)	312 (134-533)	1.4 (0.6-2.4)
Haemophilus influenzae	Resistance to one or more antibiotics	26 (20-34)	0.1 (0.1-0.2)	2240 (1720-2930)	10.0 (7.7-13.1)	6 (3-10)	0.0 (0.0-0.0)	533 (250-864)	2.4 (1.1-3.9)
Klebsiella pneumoniae	Aminoglycosides	121 (92-162)	0.5 (0.4-0.7)	10500 (7970-14000)	46.9 (35.7-62.8)	9 (6-13)	0.0 (0.0-0.1)	795 (506-1170)	3.6 (2.3-5.2)
Klebsiella pneumoniae	Beta Lactam/Beta-lactamase inhibitors	232 (180-303)	1.0 (0.8-1.4)	20100 (15600-26200)	89.9 (70.0-117.4)	3 (1-6)	0.0 (0.0-0.0)	262 (91-508)	1.2 (0.4-2.3)
Klebsiella pneumoniae	Carbapenem	58 (36-93)	0.3 (0.2-0.4)	5050 (3150-8090)	22.6 (14.1-36.2)	13 (8-22)	0.1 (0.0-0.1)	1150 (682-1880)	5.2 (3.1-8.4)
Klebsiella pneumoniae	Fluoroquinolones	163 (126-215)	0.7 (0.6-1.0)	14100 (11000-18600)	63.1 (49.2-83.5)	11 (7-16)	0.0 (0.0-0.1)	959 (600-1410)	4.3 (2.7-6.3)
Klebsiella pneumoniae	Third-generation cephalosporins	227 (167-299)	1.0 (0.7-1.3)	19700 (14500-25900)	88.2 (65.0-116.1)	28 (11-52)	0.1 (0.0-0.2)	2440 (936-4490)	10.9 (4.2-20.1)
Klebsiella pneumoniae	Trimethoprim-Sulfamethoxazole	227 (179-297)	1.0 (0.8-1.3)	19700 (15500-25700)	88.2 (69.4-114.9)	12 (6-18)	0.1 (0.0-0.1)	993 (502-1590)	4.4 (2.3-7.1)
Klebsiella pneumoniae	Resistance to one or more antibiotics	269 (212-351)	1.2 (0.9-1.6)	23300 (18400-30400)	104.4 (82.5-136.0)	76 (52-110)	0.3 (0.2-0.5)	6610 (4490-9490)	29.6 (20.1-42.5)
Morganella spp.	Fluoroquinolones	0 (0-0)	0.0 (0.0-0.0)	9 (5-16)	0.0 (0.0-0.1)	0 (0-0)	0.0 (0.0-0.0)	1 (1-3)	0.0 (0.0-0.0)
Morganella spp.	Fourth-generation cephalosporins	0 (0-0)	0.0 (0.0-0.0)	2 (1-4)	0.0 (0.0-0.0)	0 (0-0)	0.0 (0.0-0.0)	0 (0-1)	0.0 (0.0-0.0)
Morganella spp.	Third-generation cephalosporins	0 (0-0)	0.0 (0.0-0.0)	8 (4-15)	0.0 (0.0-0.1)	0 (0-0)	0.0 (0.0-0.0)	2 (1-3)	0.0 (0.0-0.0)
Morganella spp.	Resistance to one or more antibiotics	0 (0-0)	0.0 (0.0-0.0)	14 (7-25)	0.1 (0.0-0.1)	0 (0-0)	0.0 (0.0-0.0)	3 (2-7)	0.0 (0.0-0.0)
Mycobacterium tuberculosis	Extensive drug resistance in TB	14 (8-24)	0.1 (0.0-0.1)	1270 (683-2090)	5.7 (3.1-9.4)	9 (5-14)	0.0 (0.0-0.1)	740 (400-1210)	3.3 (1.8-5.4)
Mycobacterium tuberculosis	Isoniazid mono-resistance	11 (7-17)	0.1 (0.0-0.1)	1090 (665-1610)	4.9 (3.0-7.2)	2 (0-6)	0.0 (0.0-0.0)	156 (0-518)	0.7 (0.0-2.3)
Mycobacterium tuberculosis	Multi-drug resistance excluding extensive drug resistance in TB	31 (18-49)	0.1 (0.1-0.2)	2880 (1660-4520)	12.9 (7.4-20.2)	18 (3-38)	0.1 (0.0-0.2)	1510 (219-3200)	6.8 (1.0-14.3)
Mycobacterium tuberculosis	Rifampicin mono-resistance	7 (4-10)	0.0 (0.0-0.0)	658 (398-956)	2.9 (1.8-4.3)	2 (0-4)	0.0 (0.0-0.0)	173 (36-355)	0.8 (0.2-1.6)
Mycobacterium tuberculosis	Resistance to one or more antibiotics	63 (45-88)	0.3 (0.2-0.4)	5900 (4220-8080)	26.4 (18.9-36.2)	30 (12-53)	0.1 (0.1-0.2)	2580 (991-4560)	11.6 (4.4-20.4)
Neisseria gonorrhoeae	Fluoroquinolones	--	--	--	--	--	--	--	--
Neisseria gonorrhoeae	Third-generation cephalosporins	--	--	--	--	--	--	--	--
Neisseria gonorrhoeae	Resistance to one or more antibiotics	--	--	--	--	--	--	--	--
Proteus spp.	Aminoglycosides	2 (1-3)	0.0 (0.0-0.0)	189 (116-299)	0.8 (0.5-1.3)	0 (0-0)	0.0 (0.0-0.0)	10 (2-21)	0.0 (0.0-0.1)
Proteus spp.	Aminopenicillin	6 (4-10)	0.0 (0.0-0.0)	567 (349-902)	2.5 (1.6-4.0)	0 (0-0)	0.0 (0.0-0.0)	12 (0-36)	0.1 (0.0-0.2)
Proteus spp.	Fluoroquinolones	2 (1-3)	0.0 (0.0-0.0)	199 (123-314)	0.9 (0.5-1.4)	0 (0-0)	0.0 (0.0-0.0)	12 (5-27)	0.1 (0.0-0.1)
Proteus spp.	Third-generation cephalosporins	2 (1-3)	0.0 (0.0-0.0)	161 (98-252)	0.7 (0.4-1.1)	0 (0-1)	0.0 (0.0-0.0)	35 (11-69)	0.2 (0.1-0.3)
Proteus spp.	Trimethoprim-Sulfamethoxazole	3 (2-6)	0.0 (0.0-0.0)	329 (203-520)	1.5 (0.9-2.3)	0 (0-0)	0.0 (0.0-0.0)	12 (1-27)	0.1 (0.0-0.1)

Pathogen	Antibiotic Class	5 and over							
		Associated with resistance				Attributable to resistance			
		Deaths		DALYs		Deaths		DALYs	
		Counts	Rate per 100 000	Counts	Rate per 100 000	Counts	Rate per 100 000	Counts	Rate per 100 000
All pathogens	Resistance to one or more antibiotics	272000 (180000-389000)	69.7 (46.3-99.8)	6670000 (4540000-9420000)	1709.6 (1163.5-2414.3)	71100 (46800-103000)	18.2 (12.0-26.3)	1760000 (1180000-2510000)	451.2 (303.4-643.2)
Acinetobacter baumannii	Aminoglycosides	11800 (6300-20000)	3.0 (1.6-5.1)	262000 (142000-448000)	67.1 (36.3-114.7)	494 (0-1330)	0.1 (0.0-0.3)	11000 (52-29500)	2.8 (0.0-7.6)
Acinetobacter baumannii	Anti-pseudomonal penicillin/Beta-Lactamase inhibitors	14400 (7860-24000)	3.7 (2.0-6.1)	321000 (175000-535000)	82.2 (44.8-137.2)	415 (184-782)	0.1 (0.0-0.2)	9260 (4060-17400)	2.4 (1.0-4.5)
Acinetobacter baumannii	Beta Lactam/Beta-lactamase inhibitors	13000 (7100-21700)	3.3 (1.8-5.6)	288000 (157000-482000)	73.9 (40.2-123.6)	40 (11-84)	0.0 (0.0-0.0)	746 (197-1540)	0.2 (0.1-0.4)
Acinetobacter baumannii	Carbapenem	12500 (6860-20800)	3.2 (1.8-5.3)	277000 (150000-465000)	71.0 (38.5-119.1)	2020 (980-3700)	0.5 (0.3-0.9)	44800 (21400-82100)	11.5 (5.5-21.0)
Acinetobacter baumannii	Fluoroquinolones	13900 (7530-23100)	3.6 (1.9-5.9)	307000 (167000-515000)	78.7 (42.8-131.9)	1740 (789-3180)	0.4 (0.2-0.8)	38500 (17400-70500)	9.9 (4.4-18.1)
Acinetobacter baumannii	Fourth-generation cephalosporins	14100 (7670-23600)	3.6 (2.0-6.1)	313000 (170000-524000)	80.3 (43.5-134.4)	37 (15-74)	0.0 (0.0-0.0)	751 (302-1500)	0.2 (0.1-0.4)
Acinetobacter baumannii	Third-generation cephalosporins	14400 (7850-24000)	3.7 (2.0-6.2)	319000 (174000-532000)	81.8 (44.5-136.3)	88 (31-179)	0.0 (0.0-0.0)	1820 (642-3720)	0.5 (0.2-1.0)
Acinetobacter baumannii	Resistance to one or more antibiotics	15300 (8360-25400)	3.9 (2.1-6.5)	337000 (184000-561000)	86.3 (47.1-143.7)	4840 (2510-8390)	1.2 (0.6-2.2)	107000 (55900-186000)	27.4 (14.3-47.6)
Citrobacter spp.	Aminoglycosides	138 (76-232)	0.0 (0.0-0.1)	3470 (1900-5790)	0.9 (0.5-1.5)	8 (0-22)	0.0 (0.0-0.0)	207 (0-530)	0.1 (0.0-0.1)
Citrobacter spp.	Anti-pseudomonal penicillin/Beta-Lactamase inhibitors	565 (306-1040)	0.1 (0.1-0.3)	13600 (7290-24900)	3.5 (1.9-6.4)	124 (55-252)	0.0 (0.0-0.1)	2970 (1320-5940)	0.8 (0.3-1.5)
Citrobacter spp.	Carbapenem	237 (130-404)	0.1 (0.0-0.1)	6040 (3340-10100)	1.5 (0.9-2.6)	61 (29-113)	0.0 (0.0-0.0)	1550 (723-2860)	0.4 (0.2-0.7)
Citrobacter spp.	Fluoroquinolones	437 (234-750)	0.1 (0.1-0.2)	10900 (5880-18600)	2.8 (1.5-4.8)	72 (28-142)	0.0 (0.0-0.0)	1790 (698-3510)	0.5 (0.2-0.9)
Citrobacter spp.	Fourth-generation cephalosporins	228 (119-399)	0.1 (0.0-0.1)	5660 (2930-9820)	1.5 (0.8-2.5)	13 (5-27)	0.0 (0.0-0.0)	330 (132-671)	0.1 (0.0-0.2)
Citrobacter spp.	Third-generation cephalosporins	893 (465-1610)	0.2 (0.1-0.4)	21900 (11400-38900)	5.6 (2.9-10.0)	85 (31-187)	0.0 (0.0-0.0)	2100 (768-4550)	0.5 (0.2-1.2)
Citrobacter spp.	Resistance to one or more antibiotics	1260 (671-2210)	0.3 (0.2-0.6)	30900 (16400-54000)	7.9 (4.2-13.9)	364 (177-679)	0.1 (0.0-0.2)	8950 (4410-16600)	2.3 (1.1-4.2)
Enterobacter spp.	Aminoglycosides	1370 (873-2030)	0.4 (0.2-0.5)	32600 (20400-49500)	8.3 (5.2-12.7)	89 (26-176)	0.0 (0.0-0.0)	2130 (623-4240)	0.5 (0.2-1.1)
Enterobacter spp.	Anti-pseudomonal penicillin/Beta-Lactamase inhibitors	6270 (4020-9170)	1.6 (1.0-2.3)	151000 (96200-222000)	38.7 (24.7-57.0)	893 (484-1440)	0.2 (0.1-0.4)	21500 (11600-34700)	5.5 (3.0-8.9)
Enterobacter spp.	Carbapenem	1410 (891-2120)	0.4 (0.2-0.5)	34000 (21100-51000)	8.7 (5.4-13.1)	364 (211-579)	0.1 (0.1-0.1)	8750 (5030-14100)	2.2 (1.3-3.6)
Enterobacter spp.	Fluoroquinolones	1830 (1140-2790)	0.5 (0.3-0.7)	43300 (26400-66900)	11.1 (6.8-17.2)	197 (100-345)	0.1 (0.0-0.1)	4630 (2310-8180)	1.2 (0.6-2.1)
Enterobacter spp.	Fourth-generation cephalosporins	5500 (3420-8220)	1.4 (0.9-2.1)	132000 (80100-198000)	33.9 (20.5-50.8)	274 (123-495)	0.1 (0.0-0.1)	6690 (3030-12100)	1.7 (0.8-3.1)
Enterobacter spp.	Trimethoprim-Sulfamethoxazole	4300 (2690-6380)	1.1 (0.7-1.6)	104000 (63900-155000)	26.7 (16.4-39.8)	254 (0-545)	0.1 (0.0-0.1)	6170 (0-13400)	1.6 (0.0-3.4)
Enterobacter spp.	Resistance to one or more antibiotics	9190 (5810-13300)	2.4 (1.5-3.4)	222000 (137000-324000)	57.0 (35.2-83.2)	2070 (1240-3100)	0.5 (0.3-0.8)	49900 (29100-75500)	12.8 (7.5-19.4)
Enterococcus faecalis	Fluoroquinolones	10200 (5880-15900)	2.6 (1.5-4.1)	260000 (152000-400000)	66.6 (39.0-102.4)	2540 (1140-4620)	0.7 (0.3-1.2)	64500 (29300-114000)	16.5 (7.5-29.2)
Enterococcus faecalis	Vancomycin	739 (417-1200)	0.2 (0.1-0.3)	19200 (10900-30700)	4.9 (2.8-7.9)	211 (86-420)	0.1 (0.0-0.1)	5510 (2270-11000)	1.4 (0.6-2.8)
Enterococcus faecalis	Resistance to one or more antibiotics	10400 (6010-16200)	2.7 (1.5-4.2)	265000 (155000-409000)	68.0 (39.7-104.8)	2750 (1320-4940)	0.7 (0.3-1.3)	70000 (34100-122000)	18.0 (8.7-31.2)
Enterococcus faecium	Fluoroquinolones	18900 (11500-28700)	4.8 (2.9-7.3)	469000 (280000-720000)	120.3 (71.6-184.6)	3650 (1060-7080)	0.9 (0.3-1.8)	90900 (26500-178000)	23.3 (6.8-45.6)
Enterococcus faecium	Vancomycin	4620 (2780-7160)	1.2 (0.7-1.8)	113000 (66600-176000)	29.0 (17.1-45.2)	1060 (492-1920)	0.3 (0.1-0.5)	26100 (11800-47300)	6.7 (3.0-12.1)
Enterococcus faecium	Resistance to one or more antibiotics	18900 (11600-28700)	4.9 (3.0-7.4)	471000 (281000-724000)	120.7 (72.0-185.5)	4710 (2290-8180)	1.2 (0.6-2.1)	117000 (56400-204000)	30.0 (14.5-52.2)
Other enterococci	Fluoroquinolones	4670 (2730-7330)	1.2 (0.7-1.9)	98400 (57700-156000)	25.2 (14.8-40.0)	883 (123-1760)	0.2 (0.0-0.5)	18500 (2650-37200)	4.8 (0.7-9.5)
Other enterococci	Vancomycin	708 (405-1160)	0.2 (0.1-0.3)	14600 (8350-23800)	3.8 (2.1-6.1)	124 (59-240)	0.0 (0.0-0.1)	2570 (1250-5120)	0.7 (0.3-1.3)
Other enterococci	Resistance to one or more antibiotics	4700 (2740-7430)	1.2 (0.7-1.9)	99100 (58200-156000)	25.4 (14.9-40.1)	1010 (311-1890)	0.3 (0.1-0.5)	21100 (6500-39900)	5.4 (1.7-10.2)
Escherichia coli	Aminoglycosides	17900 (11000-26500)	4.6 (2.8-6.8)	408000 (253000-605000)	104.5 (64.7-155.0)	1060 (599-1650)	0.3 (0.2-0.4)	24100 (13700-37900)	6.2 (3.5-9.7)
Escherichia coli	Aminopenicillin	63100 (40100-94000)	16.2 (10.3-24.1)	1430000 (906000-2100000)	365.8 (232.3-537.9)	951 (563-1500)	0.2 (0.1-0.4)	20800 (12400-32800)	5.3 (3.2-8.4)
Escherichia coli	Beta Lactam/Beta-lactamase inhibitors	48900 (30900-72800)	12.5 (7.9-18.7)	1110000 (705000-1640000)	284.6 (180.7-421.2)	2090 (1260-3210)	0.5 (0.3-0.8)	46400 (27900-70900)	11.9 (7.2-18.2)
Escherichia coli	Carbapenem	5660 (3190-9200)	1.5 (0.8-2.4)	131000 (75400-214000)	33.7 (19.3-54.8)	1230 (619-2150)	0.3 (0.2-0.6)	28500 (14400-49400)	7.3 (3.7-12.7)
Escherichia coli	Fluoroquinolones	40000 (25200-59400)	10.2 (6.5-15.2)	905000 (569000-1340000)	231.9 (145.9-342.7)	4160 (2500-6430)	1.1 (0.6-1.6)	93500 (56400-143000)	24.0 (14.4-36.6)
Escherichia coli	Third-generation cephalosporins	40100 (25300-60300)	10.3 (6.5-15.5)	918000 (582000-1360000)	235.2 (149.3-349.4)	5690 (2400-10800)	1.5 (0.6-2.8)	130000 (55200-245000)	33.3 (14.1-62.8)
Escherichia coli	Trimethoprim-Sulfamethoxazole	44700 (28500-66700)	11.5 (7.3-17.1)	1020000 (650000-1490000)	260.7 (166.6-382.6)	2600 (1530-3990)	0.7 (0.4-1.0)	58500 (34500-90100)	15.0 (8.8-23.1)
Escherichia coli	Resistance to one or more antibiotics	70400 (44800-105000)	18.0 (11.5-26.8)	1590000 (1010000-2340000)	407.4 (259.3-598.5)	17800 (10800-28200)	4.6 (2.8-7.2)	402000 (243000-626000)	103.0 (62.4-160.4)
Group A Streptococcus	Macrolide	809 (368-1620)	0.2 (0.1-0.4)	20900 (10600-38800)	5.4 (2.7-9.9)	78 (0-308)	0.0 (0.0-0.1)	2000 (0-7200)	0.5 (0.0-1.8)
Group A Streptococcus	Resistance to one or more antibiotics	809 (368-1620)	0.2 (0.1-0.4)	20900 (10600-38800)	5.4 (2.7-9.9)	78 (0-308)	0.0 (0.0-0.1)	2000 (0-7200)	0.5 (0.0-1.8)
Group B Streptococcus	Fluoroquinolones	699 (432-1100)	0.2 (0.1-0.3)	18800 (11900-28700)	4.8 (3.1-7.4)	114 (18-272)	0.0 (0.0-0.1)	3050 (505-7250)	0.8 (0.1-1.9)
Group B Streptococcus	Macrolide	4750 (3010-7210)	1.2 (0.8-1.8)	121000 (78700-176000)	30.9 (20.2-45.1)	485 (0-1380)	0.1 (0.0-0.4)	12300 (0-34000)	3.1 (0.0-8.7)
Group B Streptococcus	Penicillin	152 (95-236)	0.0 (0.0-0.1)	4190 (2660-6330)	1.1 (0.7-1.6)	32 (6-70)	0.0 (0.0-0.0)	847 (164-1880)	0.2 (0.0-0.5)
Group B Streptococcus	Resistance to one or more antibiotics	5010 (3210-7580)	1.3 (0.8-1.9)	128000 (84100-187000)	32.8 (21.5-47.9)	630 (0-1560)	0.2 (0.0-0.4)	16200 (0-39300)	4.1 (0.0-10.1)
Haemophilus influenzae	Aminopenicillin	213 (153-294)	0.1 (0.0-0.1)	5750 (4280-7710)	1.5 (1.1-2.0)	32 (2-65)	0.0 (0.0-0.0)	857 (84-1710)	0.2 (0.0-0.4)
Haemophilus influenzae	Third-generation cephalosporins	148 (107-200)	0.0 (0.0-0.1)	3980 (2960-5190)	1.0 (0.8-1.3)	44 (18-75)	0.0 (0.0-0.0)	1170 (509-1970)	0.3 (0.1-0.5)
Haemophilus influenzae	Resistance to one or more antibiotics	319 (240-426)	0.1 (0.1-0.1)	8590 (6710-11100)	2.2 (1.7-2.8)	76 (36-128)	0.0 (0.0-0.0)	2030 (999-3310)	0.5 (0.3-0.8)
Klebsiella pneumoniae	Aminoglycosides	23100 (15100-34200)	5.9 (3.9-8.8)	532000 (347000-778000)	136.2 (88.9-199.5)	1730 (989-2860)	0.4 (0.3-0.7)	39900 (22700-65500)	10.2 (5.8-16.8)
Klebsiella pneumoniae	Beta Lactam/Beta-lactamase inhibitors	35800 (23400-52100)	9.2 (6.0-13.4)	828000 (541000-1200000)	212.2 (138.6-308.8)	430 (215-741)	0.1 (0.1-0.2)	9880 (4870-17200)	2.5 (1.2-4.4)
Klebsiella pneumoniae	Carbapenem	16800 (10700-24600)	4.3 (2.7-6.3)	386000 (245000-566000)	98.8 (62.8-145.0)	3710 (2210-5740)	1.0 (0.6-1.5)	85200 (50700-132000)	21.8 (13.0-33.8)
Klebsiella pneumoniae	Fluoroquinolones	29500 (19300-43200)	7.6 (4.9-11.1)	679000 (442000-991000)	174.0 (113.4-253.9)	1990 (1130-3150)	0.5 (0.3-0.8)	45900 (26400-72700)	11.8 (6.8-18.6)
Klebsiella pneumoniae	Third-generation cephalosporins	33600 (22200-49000)	8.6 (5.7-12.6)	778000 (513000-1130000)	199.3 (131.5-289.7)	2790 (957-5570)	0.7 (0.2-1.4)	65200 (22500-128000)	16.7 (5.8-32.9)
Klebsiella pneumoniae	Trimethoprim-Sulfamethoxazole	31800 (20900-46400)	8.2 (5.3-11.9)	742000 (487000-1070000)	190.2 (124.9-274.5)	1460 (692-2620)	0.4 (0.2-0.7)	34200 (16200-60800)	8.8 (4.1-15.6)
Klebsiella pneumoniae	Resistance to one or more antibiotics	39100 (25600-57100)	10.0 (6.6-14.6)	905000 (591000-1320000)	232.0 (151.5-338.4)	12100 (7670-18300)	3.1 (2.0-4.7)	280000 (177000-419000)	71.8 (45.3-107.5)
Morganella spp.	Fluoroquinolones	72 (42-115)	0.0 (0.0-0.0)	1460 (886-2280)	0.4 (0.2-0.6)	11 (4-22)	0.0 (0.0-0.0)	231 (90-441)	0.1 (0.0-0.1)
Morganella spp.	Fourth-generation cephalosporins	18 (10-29)	0.0 (0.0-0.0)	365 (224-576)	0.1 (0.1-0.1)	4 (2-8)	0.0 (0.0-0.0)	83 (39-160)	0.0 (0.0-0.0)
Morganella spp.	Third-generation cephalosporins	112 (67-182)	0.0 (0.0-0.0)	2140 (1330-3370)	0.5 (0.3-0.9)	24 (9-47)	0.0 (0.0-0.0)	445 (180-881)	0.1 (0.0-0.2)
Morganella spp.	Resistance to one or more antibiotics	147 (88-232)	0.0 (0.0-0.1)	2880 (1810-4500)	0.7 (0.5-1.2)	39 (19-71)	0.0 (0.0-0.0)	759 (381-1370)	0.2 (0.1-0.4)
Mycobacterium tuberculosis	Extensive drug resistance in TB	2590 (1470-3920)	0.7 (0.4-1.0)	106000 (60200-160000)	27.1 (15.4-40.9)	1590 (898-2400)	0.4 (0.2-0.6)	61200 (34900-92300)	15.7 (8.9-23.7)
Mycobacterium tuberculosis	Isoniazid mono-resistance	1910 (1210-2750)	0.5 (0.3-0.7)	85500 (55000-123000)	21.9 (14.1-31.5)	297 (0-987)	0.1 (0.0-0.3)	12000 (0-39200)	3.1 (0.0-10.0)
Mycobacterium tuberculosis	Multi-drug resistance excluding extensive drug resistance in TB	5670 (3330-8210)	1.5 (0.9-2.1)	240000 (141000-354000)	61.5 (36.1-90.7)	3250 (487-6350)	0.8 (0.1-1.6)	125000 (17700-246000)	32.0 (4.5-63.0)
Mycobacterium tuberculosis	Rifampicin mono-resistance	1040 (665-1450)	0.3 (0.2-0.4)	46100 (29800-64200)	11.8 (7.6-16.5)	289 (45-584)	0.1 (0.0-0.1)	12000 (2350-23800)	3.1 (0.6-6.1)
Mycobacterium tuberculosis	Resistance to one or more antibiotics	11200 (8670-14100)	2.9 (2.2-3.6)	477000 (372000-597000)	122.3 (95.3-153.0)	5420 (2120-9040)	1.4 (0.5-2.3)	210000 (82900-348000)	53.8 (21.2-89.1)
Neisseria gonorrhoeae	Fluoroquinolones	--	--	1730 (951-2930)	0.4 (0.2-0.7)	--	--	166 (40-355)	0.0 (0.0-0.1)
Neisseria gonorrhoeae	Third-generation cephalosporins	--	--	29 (12-61)	0.0 (0.0-0.0)	--	--	7 (2-16)	0.0 (0.0-0.0)
Neisseria gonorrhoeae	Resistance to one or more antibiotics	--	--	1740 (954-2940)	0.4 (0.2-0.8)	--	--	173 (47-367)	0.0 (0.0-0.1)
Proteus spp.	Aminoglycosides	1980 (1260-2870)	0.5 (0.3-0.7)	43000 (27400-63000)	11.0 (7.0-16.2)	107 (11-242)	0.0 (0.0-0.1)	2320 (262-5180)	0.6 (0.1-1.3)
Proteus spp.	Aminopenicillin	5780 (3730-8380)	1.5 (1.0-2.1)	124000 (78700-180000)	31.7 (20.2-46.1)	133 (0-420)	0.0 (0.0-0.1)	2830 (0-8800)	0.7 (0.0-2.3)
Proteus spp.	Fluoroquinolones	1910 (1210-2840)	0.5 (0.3-0.7)	40600 (25500-60100)	10.4 (6.5-15.4)	131 (51-256)	0.0 (0.0-0.1)	2750 (1070-5440)	0.7 (0.3-1.4)
Proteus spp.	Third-generation cephalosporins	1270 (773-1910)	0.3 (0.2-0.5)	27100 (16600-41000)	6.9 (4.2-10.5)	286 (93-563)	0.1 (0.0-0.1)	6100 (2010-12100)	1.6 (0.5-3.1)
Proteus spp.	Trimethoprim-Sulfamethoxazole	2990 (1900-4360)	0.8 (0.5-1.1)	64700 (41000-94900)	16.6 (10.5-24.3)	105 (0-244)	0.0 (0.0-0.1)	2280 (6-5240)	0.6 (0.0-1.3)

Proteus spp.	Resistance to one or more antibiotics	25 (15-39)	6.3 (3.8-9.8)	2220 (1360-3480)	558.7 (341.8-875.9)	3 (2-6)	0.9 (0.4-1.6)	301 (153-552)	75.8 (38.6-139.0)
Pseudomonas aeruginosa	Aminoglycosides	176 (121-249)	44.4 (30.5-62.8)	15700 (10800-22200)	3949.4 (2717.4-5587.3)	4 (0-11)	1.1 (0.0-2.7)	391 (0-946)	98.5 (0.0-238.3)
Pseudomonas aeruginosa	Anti-pseudomonal penicillin/Beta-Lactamase inhibitors	278 (192-390)	69.9 (48.3-98.3)	24700 (17100-34700)	6219.9 (4295.3-8741.4)	21 (12-32)	5.2 (3.1-8.2)	1840 (1080-2880)	462.9 (273.0-725.3)
Pseudomonas aeruginosa	Carbapenem	294 (204-411)	74.1 (51.2-103.5)	26200 (18100-36600)	6589.2 (4558.8-9220.2)	51 (32-78)	12.8 (8.0-19.7)	4530 (2840-6960)	1139.5 (715.9-1752.6)
Pseudomonas aeruginosa	Fluoroquinolones	302 (212-423)	76.0 (53.3-106.4)	26900 (18800-37600)	6764.6 (4742.5-9473.7)	32 (20-50)	8.2 (5.1-12.5)	2890 (1810-4410)	726.5 (456.9-1110.1)
Pseudomonas aeruginosa	Fourth-generation cephalosporins	266 (187-373)	67.0 (47.2-94.0)	23700 (16700-33300)	5961.7 (4198.3-8372.2)	6 (3-10)	1.5 (0.8-2.5)	532 (288-892)	134.0 (72.6-224.5)
Pseudomonas aeruginosa	Third-generation cephalosporins	356 (239-492)	89.6 (60.3-123.9)	31700 (21300-43800)	7969.8 (5362.7-11023.4)	17 (7-29)	4.2 (1.8-7.4)	1500 (621-2620)	377.7 (156.4-658.5)
Pseudomonas aeruginosa	Resistance to one or more antibiotics	509 (352-707)	128.1 (88.6-177.9)	45300 (31300-62900)	11398.8 (7880.3-15833.4)	131 (83-199)	33.0 (20.9-50.2)	11700 (7380-17700)	2939.2 (1858.6-4467.3)
Salmonella Paratyphi	Fluoroquinolones	--	--	0 (0-0)	0.0 (0.0-0.0)	--	--	0 (0-0)	0.0 (0.0-0.0)
Salmonella Paratyphi	Multi-drug resistance in Salmonella Typhi and Paratyphi	--	--	0 (0-0)	0.0 (0.0-0.0)	--	--	0 (0-0)	0.0 (0.0-0.0)
Salmonella Paratyphi	Resistance to one or more antibiotics	--	--	0 (0-0)	0.0 (0.0-0.0)	--	--	0 (0-0)	0.0 (0.0-0.0)
Salmonella Typhi	Fluoroquinolones	32 (14-60)	8.0 (3.6-15.2)	2810 (1280-5350)	708.6 (321.7-1346.9)	6 (1-16)	1.6 (0.2-4.1)	571 (86-1440)	143.7 (21.8-362.2)
Salmonella Typhi	Multi-drug resistance in Salmonella Typhi and Paratyphi	9 (4-18)	2.2 (1.0-4.5)	785 (348-1600)	197.7 (87.6-402.5)	1 (0-3)	0.3 (0.0-0.8)	93 (0-287)	23.3 (0.0-72.2)
Salmonella Typhi	Resistance to one or more antibiotics	38 (18-71)	9.6 (4.6-17.9)	3390 (1630-6340)	852.4 (411.2-1595.6)	7 (2-18)	1.9 (0.4-4.5)	663 (144-1570)	167.0 (36.2-395.7)
Non-typhoidal *Salmonella*	Fluoroquinolones	1 (1-2)	0.3 (0.2-0.6)	113 (57-200)	28.6 (14.4-50.4)	0 (0-1)	0.1 (0.0-0.2)	24 (4-56)	5.9 (0.9-14.1)
Non-typhoidal *Salmonella*	Resistance to one or more antibiotics	1 (1-2)	0.3 (0.2-0.6)	113 (57-200)	28.6 (14.4-50.4)	0 (0-1)	0.1 (0.0-0.2)	24 (4-56)	5.9 (0.9-14.1)
Serratia spp.	Aminoglycosides	20 (12-31)	5.0 (2.9-7.8)	1750 (1040-2750)	440.8 (262.0-691.3)	1 (0-3)	0.3 (0.0-0.8)	104 (0-276)	26.1 (0.0-69.5)
Serratia spp.	Anti-pseudomonal penicillin/Beta-Lactamase inhibitors	18 (11-29)	4.6 (2.7-7.4)	1610 (968-2620)	405.5 (243.8-659.8)	3 (2-6)	0.9 (0.4-1.6)	307 (151-558)	77.2 (37.9-140.5)
Serratia spp.	Carbapenem	11 (6-17)	2.7 (1.6-4.2)	939 (568-1470)	236.5 (143.0-370.2)	3 (1-5)	0.7 (0.4-1.2)	247 (124-440)	62.2 (31.2-110.8)
Serratia spp.	Fluoroquinolones	9 (5-15)	2.2 (1.2-3.7)	768 (428-1290)	193.4 (107.8-326.0)	1 (0-2)	0.2 (0.0-0.5)	82 (16-190)	20.5 (4.2-47.9)
Serratia spp.	Fourth-generation cephalosporins	32 (19-52)	8.0 (4.8-13.0)	2810 (1700-4590)	707.4 (428.3-1155.8)	3 (1-6)	0.8 (0.3-1.6)	285 (112-570)	71.7 (28.3-143.6)
Serratia spp.	Third-generation cephalosporins	34 (20-55)	8.6 (5.1-14.0)	3040 (1820-4930)	765.1 (457.6-1241.1)	2 (1-4)	0.5 (0.1-1.0)	160 (51-357)	40.3 (12.9-89.8)
Serratia spp.	Resistance to one or more antibiotics	52 (32-85)	13.2 (8.1-21.3)	4670 (2860-7520)	1175.4 (720.8-1892.2)	13 (7-23)	3.4 (1.8-5.9)	1180 (649-2080)	298.1 (163.5-524.4)
Shigella spp.	Fluoroquinolones	2 (1-5)	0.5 (0.1-1.3)	179 (55-476)	45.0 (13.7-119.8)	0 (0-1)	0.1 (0.0-0.3)	37 (4-121)	9.2 (1.0-30.4)
Shigella spp.	Resistance to one or more antibiotics	2 (1-5)	0.5 (0.1-1.3)	179 (55-476)	45.0 (13.7-119.8)	0 (0-1)	0.1 (0.0-0.3)	37 (4-121)	9.2 (1.0-30.4)
Staphylococcus aureus	Fluoroquinolones	291 (202-407)	73.2 (50.8-102.5)	25900 (17900-36300)	6516.9 (4513.7-9140.1)	13 (6-23)	3.4 (1.4-5.9)	1180 (490-2080)	298.2 (123.4-524.0)
Staphylococcus aureus	Macrolide	438 (313-606)	110.3 (78.9-152.5)	39000 (27900-53900)	9813.2 (7013.5-13569.9)	18 (7-32)	4.5 (1.7-8.1)	1580 (613-2850)	398.6 (154.4-716.7)
Staphylococcus aureus	Methicillin	334 (229-475)	84.1 (57.6-119.5)	29700 (20400-42200)	7486.9 (5129.4-10633.8)	86 (37-152)	21.6 (9.3-38.4)	7640 (3300-13600)	1923.0 (829.8-3416.5)
Staphylococcus aureus	Trimethoprim-Sulfamethoxazole	97 (66-134)	24.3 (16.7-33.7)	8590 (5910-11900)	2163.2 (1488.7-2992.9)	9 (4-14)	2.2 (1.1-3.6)	793 (387-1260)	199.7 (97.4-317.0)
Staphylococcus aureus	Vancomycin	9 (6-12)	2.1 (1.5-3.1)	757 (519-1100)	190.7 (130.6-275.9)	3 (1-5)	0.6 (0.3-1.2)	227 (113-409)	57.3 (28.5-102.9)
Staphylococcus aureus	Resistance to one or more antibiotics	591 (419-818)	148.9 (105.5-205.8)	52600 (37300-72800)	13248.5 (9380.6-18318.4)	128 (74-208)	32.3 (18.6-52.3)	11400 (6580-18500)	2876.9 (1657.1-4651.4)
Streptococcus pneumoniae	Beta Lactam/Beta-lactamase inhibitors	91 (61-131)	22.9 (15.3-33.0)	8070 (5410-11700)	2032.8 (1361.1-2938.2)	1 (0-4)	0.4 (0.0-1.0)	131 (2-362)	32.9 (0.6-91.3)
Streptococcus pneumoniae	Carbapenem	179 (121-262)	45.1 (30.5-65.8)	15900 (10800-23300)	4012.2 (2714.3-5858.0)	40 (18-71)	10.0 (4.5-17.8)	3550 (1580-6300)	892.8 (397.4-1586.5)
Streptococcus pneumoniae	Fluoroquinolones	20 (13-30)	5.0 (3.3-7.4)	1780 (1180-2630)	448.6 (296.8-661.4)	2 (1-5)	0.6 (0.1-1.3)	208 (49-453)	52.3 (12.4-113.9)
Streptococcus pneumoniae	Macrolide	182 (124-263)	45.9 (31.1-66.3)	16200 (11000-23400)	4085.8 (2767.7-5893.9)	7 (0-18)	1.8 (0.0-4.6)	619 (0-1640)	155.9 (0.0-412.9)
Streptococcus pneumoniae	Penicillin	184 (122-266)	46.3 (30.6-67.0)	16300 (10800-23700)	4114.8 (2720.7-5965.0)	9 (4-16)	2.2 (1.1-4.0)	784 (373-1400)	197.4 (94.0-351.5)
Streptococcus pneumoniae	Third-generation cephalosporins	78 (52-114)	19.5 (13.1-28.8)	6910 (4620-10200)	1739.0 (1164.1-2563.7)	3 (1-6)	0.7 (0.3-1.4)	250 (98-500)	62.9 (24.7-125.8)
Streptococcus pneumoniae	Trimethoprim-Sulfamethoxazole	430 (305-604)	108.2 (76.9-152.0)	38200 (27200-53700)	9629.0 (6839.9-13524.9)	41 (6-85)	10.2 (1.4-21.3)	3620 (494-7530)	910.8 (124.5-1895.8)
Streptococcus pneumoniae	Resistance to one or more antibiotics	500 (355-700)	125.8 (89.3-176.2)	44500 (31600-62300)	11192.5 (7948.3-15679.5)	103 (61-157)	25.9 (15.4-39.4)	9150 (5430-13900)	2305.0 (1368.4-3506.7)

Proteus spp.	Resistance to one or more antibiotics	12 (7-20)	0.3 (0.2-0.4)	1120 (673-1760)	23.2 (13.9-36.4)	2 (1-3)	0.0 (0.0-0.1)	151 (72-265)	3.1 (1.5-5.5)
Pseudomonas aeruginosa	Aminoglycosides	111 (84-143)	2.3 (1.7-3.0)	9780 (7470-12700)	202.3 (154.5-262.3)	3 (0-6)	0.1 (0.0-0.1)	244 (0-565)	5.0 (0.0-11.7)
Pseudomonas aeruginosa	Anti-pseudomonal penicillin/Beta-Lactamase inhibitors	174 (135-221)	3.6 (2.8-4.6)	15400 (12000-19600)	318.4 (247.2-405.3)	13 (8-19)	0.3 (0.2-0.4)	1140 (730-1720)	23.5 (15.1-35.6)
Pseudomonas aeruginosa	Carbapenem	185 (144-236)	3.8 (3.0-4.9)	16400 (12800-20800)	338.8 (263.9-431.2)	32 (22-46)	0.7 (0.5-0.9)	2840 (1940-4060)	58.7 (40.1-83.9)
Pseudomonas aeruginosa	Fluoroquinolones	189 (149-243)	3.9 (3.1-5.0)	16700 (13200-21500)	345.5 (272.1-445.5)	20 (14-28)	0.4 (0.3-0.6)	1790 (1240-2530)	37.1 (25.5-52.2)
Pseudomonas aeruginosa	Fourth-generation cephalosporins	167 (130-218)	3.4 (2.7-4.5)	14700 (11500-19300)	304.8 (237.4-398.2)	4 (2-6)	0.1 (0.0-0.1)	332 (179-558)	6.9 (3.7-11.5)
Pseudomonas aeruginosa	Third-generation cephalosporins	226 (173-291)	4.7 (3.6-6.0)	20000 (15300-25700)	414.1 (315.7-531.8)	11 (5-19)	0.2 (0.1-0.4)	977 (433-1650)	20.2 (9.0-34.2)
Pseudomonas aeruginosa	Resistance to one or more antibiotics	320 (252-405)	6.6 (5.2-8.4)	28300 (22300-35900)	586.1 (461.0-741.4)	83 (57-118)	1.7 (1.2-2.4)	7330 (5040-10500)	151.5 (104.3-216.6)
Salmonella Paratyphi	Fluoroquinolones	0 (0-0)	0.0 (0.0-0.0)	1 (0-3)	0.0 (0.0-0.1)	0 (0-0)	0.0 (0.0-0.0)	0 (0-1)	0.0 (0.0-0.0)
Salmonella Paratyphi	Multi-drug resistance in Salmonella Typhi and Paratyphi	0 (0-0)	0.0 (0.0-0.0)	0 (0-0)	0.0 (0.0-0.0)	0 (0-0)	0.0 (0.0-0.0)	0 (0-0)	0.0 (0.0-0.0)
Salmonella Paratyphi	Resistance to one or more antibiotics	0 (0-0)	0.0 (0.0-0.0)	1 (1-3)	0.0 (0.0-0.1)	0 (0-0)	0.0 (0.0-0.0)	0 (0-1)	0.0 (0.0-0.0)
Salmonella Typhi	Fluoroquinolones	12 (6-24)	0.3 (0.1-0.5)	1100 (500-2120)	22.7 (10.3-43.8)	3 (0-6)	0.1 (0.0-0.1)	221 (35-530)	4.6 (0.7-11.0)
Salmonella Typhi	Multi-drug resistance in Salmonella Typhi and Paratyphi	4 (2-7)	0.1 (0.0-0.2)	324 (144-642)	6.7 (3.0-13.3)	0 (0-1)	0.0 (0.0-0.0)	38 (0-118)	0.8 (0.0-2.4)
Salmonella Typhi	Resistance to one or more antibiotics	15 (7-28)	0.3 (0.2-0.6)	1330 (646-2510)	27.6 (13.4-51.8)	3 (1-7)	0.1 (0.0-0.1)	259 (62-594)	5.4 (1.3-12.3)
Non-typhoidal *Salmonella*	Fluoroquinolones	1 (1-2)	0.0 (0.0-0.0)	120 (49-248)	2.5 (1.0-5.1)	0 (0-1)	0.0 (0.0-0.0)	22 (3-56)	0.5 (0.1-1.2)
Non-typhoidal *Salmonella*	Resistance to one or more antibiotics	1 (1-2)	0.0 (0.0-0.0)	120 (49-248)	2.5 (1.0-5.1)	0 (0-1)	0.0 (0.0-0.0)	22 (3-56)	0.5 (0.1-1.2)
Serratia spp.	Aminoglycosides	5 (3-9)	0.1 (0.1-0.2)	468 (272-774)	9.7 (5.6-16.0)	0 (0-1)	0.0 (0.0-0.0)	28 (0-78)	0.6 (0.0-1.6)
Serratia spp.	Anti-pseudomonal penicillin/Beta-Lactamase inhibitors	5 (3-8)	0.1 (0.1-0.2)	419 (240-712)	8.7 (5.0-14.7)	1 (0-2)	0.0 (0.0-0.0)	79 (39-147)	1.6 (0.8-3.0)
Serratia spp.	Carbapenem	3 (2-5)	0.1 (0.0-0.1)	246 (141-404)	5.1 (2.9-8.3)	1 (0-1)	0.0 (0.0-0.0)	65 (32-116)	1.3 (0.7-2.4)
Serratia spp.	Fluoroquinolones	2 (1-4)	0.0 (0.0-0.1)	188 (106-313)	3.9 (2.2-6.5)	0 (0-1)	0.0 (0.0-0.0)	20 (4-47)	0.4 (0.1-1.0)
Serratia spp.	Fourth-generation cephalosporins	9 (5-15)	0.2 (0.1-0.3)	778 (455-1350)	16.1 (9.4-28.0)	1 (0-2)	0.0 (0.0-0.0)	81 (32-165)	1.7 (0.7-3.4)
Serratia spp.	Third-generation cephalosporins	9 (5-15)	0.2 (0.1-0.3)	801 (455-1330)	16.6 (9.4-27.5)	0 (0-1)	0.0 (0.0-0.0)	40 (13-87)	0.8 (0.3-1.8)
Serratia spp.	Resistance to one or more antibiotics	14 (8-23)	0.3 (0.2-0.5)	1240 (732-2040)	25.6 (15.1-42.2)	4 (2-6)	0.1 (0.0-0.1)	313 (163-545)	6.5 (3.4-11.3)
Shigella spp.	Fluoroquinolones	15 (4-38)	0.3 (0.1-0.8)	1420 (392-3620)	29.4 (8.1-74.8)	3 (0-10)	0.1 (0.0-0.2)	286 (30-943)	5.9 (0.6-19.5)
Shigella spp.	Resistance to one or more antibiotics	15 (4-38)	0.3 (0.1-0.8)	1420 (392-3620)	29.4 (8.1-74.8)	3 (0-10)	0.1 (0.0-0.2)	286 (30-943)	5.9 (0.6-19.5)
Staphylococcus aureus	Fluoroquinolones	319 (247-418)	6.6 (5.1-8.6)	28300 (21800-37100)	584.7 (451.6-766.2)	15 (6-24)	0.3 (0.1-0.5)	1290 (553-2160)	26.6 (11.4-44.7)
Staphylococcus aureus	Macrolide	491 (386-625)	10.1 (8.0-12.9)	43400 (34200-55300)	898.1 (706.3-1143.7)	20 (8-34)	0.4 (0.2-0.7)	1760 (746-3040)	36.3 (15.4-62.9)
Staphylococcus aureus	Methicillin	372 (285-488)	7.7 (5.9-10.1)	33000 (25200-43300)	681.7 (521.9-894.7)	96 (42-162)	2.0 (0.9-3.3)	8460 (3680-14300)	174.9 (76.1-296.3)
Staphylococcus aureus	Trimethoprim-Sulfamethoxazole	112 (87-146)	2.3 (1.8-3.0)	9910 (7690-12900)	205.0 (159.1-267.6)	10 (5-16)	0.2 (0.1-0.3)	914 (461-1410)	18.9 (9.5-29.2)
Staphylococcus aureus	Vancomycin	9 (7-12)	0.2 (0.1-0.3)	819 (609-1100)	16.9 (12.6-22.7)	3 (1-5)	0.1 (0.0-0.1)	245 (128-413)	5.1 (2.6-8.5)
Staphylococcus aureus	Resistance to one or more antibiotics	657 (521-839)	13.6 (10.8-17.4)	58200 (46100-74300)	1202.7 (954.0-1536.1)	143 (82-221)	3.0 (1.7-4.6)	12700 (7290-19600)	261.8 (150.8-404.5)
Streptococcus pneumoniae	Beta Lactam/Beta-lactamase inhibitors	343 (245-472)	7.1 (5.1-9.8)	30300 (21700-41700)	627.4 (449.2-862.5)	5 (0-15)	0.1 (0.0-0.3)	472 (8-1300)	9.8 (0.2-26.8)
Streptococcus pneumoniae	Carbapenem	684 (499-916)	14.1 (10.3-18.9)	60500 (44100-81100)	1251.1 (913.0-1676.4)	152 (70-261)	3.1 (1.4-5.4)	13500 (6200-23100)	278.4 (128.2-476.9)
Streptococcus pneumoniae	Fluoroquinolones	71 (53-97)	1.5 (1.1-2.0)	6300 (4650-8550)	130.2 (96.2-176.7)	8 (2-17)	0.2 (0.0-0.4)	725 (173-1510)	15.0 (3.6-31.3)
Streptococcus pneumoniae	Macrolide	679 (515-909)	14.0 (10.6-18.8)	60100 (45500-80400)	1242.7 (941.2-1662.5)	26 (0-66)	0.5 (0.0-1.4)	2290 (0-5820)	47.3 (0.0-120.4)
Streptococcus pneumoniae	Penicillin	684 (493-921)	14.1 (10.2-19.0)	60500 (43600-81500)	1250.7 (901.7-1684.3)	32 (16-55)	0.7 (0.3-1.1)	2810 (1410-4830)	58.1 (29.2-99.8)
Streptococcus pneumoniae	Third-generation cephalosporins	275 (199-373)	5.7 (4.1-7.7)	24300 (17600-33000)	502.3 (364.1-682.8)	8 (3-16)	0.2 (0.1-0.3)	752 (299-1440)	15.5 (6.2-29.8)
Streptococcus pneumoniae	Trimethoprim-Sulfamethoxazole	1540 (1220-1980)	31.8 (25.3-41.0)	136000 (108000-175000)	2812.5 (2236.4-3624.2)	144 (19-288)	3.0 (0.4-6.0)	12700 (1700-25400)	263.6 (35.1-526.0)
Streptococcus pneumoniae	Resistance to one or more antibiotics	1800 (1440-2280)	37.2 (29.7-47.1)	159000 (127000-201000)	3287.9 (2631.4-4163.1)	376 (250-547)	7.8 (5.2-11.3)	33300 (22100-48400)	687.7 (457.2-1000.8)

Proteus spp.	Resistance to one or more antibiotics	6 (4-10)	0.0 (0.0-0.0)	608 (377-958)	2.7 (1.7-4.3)	1 (0-2)	0.0 (0.0-0.0)	80 (39-139)	0.4 (0.2-0.6)
Pseudomonas aeruginosa	Aminoglycosides	45 (34-59)	0.2 (0.2-0.3)	3890 (2980-5090)	17.4 (13.4-22.8)	1 (0-3)	0.0 (0.0-0.0)	99 (2-228)	0.4 (0.0-1.0)
Pseudomonas aeruginosa	Anti-pseudomonal penicillin/Beta-Lactamase inhibitors	70 (54-91)	0.3 (0.2-0.4)	6100 (4700-7910)	27.3 (21.1-35.4)	5 (3-8)	0.0 (0.0-0.0)	450 (281-680)	2.0 (1.3-3.0)
Pseudomonas aeruginosa	Carbapenem	75 (59-98)	0.3 (0.3-0.4)	6530 (5100-8490)	29.2 (22.8-38.0)	13 (9-19)	0.1 (0.0-0.1)	1130 (770-1610)	5.1 (3.4-7.2)
Pseudomonas aeruginosa	Fluoroquinolones	77 (60-99)	0.3 (0.3-0.4)	6680 (5230-8610)	29.9 (23.4-38.5)	8 (6-12)	0.0 (0.0-0.1)	721 (499-1010)	3.2 (2.2-4.5)
Pseudomonas aeruginosa	Fourth-generation cephalosporins	68 (53-88)	0.3 (0.2-0.4)	5880 (4600-7630)	26.3 (20.6-34.2)	2 (1-3)	0.0 (0.0-0.0)	133 (73-221)	0.6 (0.3-1.0)
Pseudomonas aeruginosa	Third-generation cephalosporins	92 (71-117)	0.4 (0.3-0.5)	7980 (6150-10100)	35.7 (27.5-45.4)	5 (2-8)	0.0 (0.0-0.0)	390 (176-660)	1.7 (0.8-3.0)
Pseudomonas aeruginosa	Resistance to one or more antibiotics	130 (103-166)	0.6 (0.5-0.7)	11300 (8910-14300)	50.6 (39.9-64.2)	34 (23-49)	0.2 (0.1-0.2)	2920 (2040-4210)	13.1 (9.1-18.9)
Salmonella Paratyphi	Fluoroquinolones	0 (0-0)	0.0 (0.0-0.0)	4 (1-9)	0.0 (0.0-0.0)	0 (0-0)	0.0 (0.0-0.0)	1 (0-2)	0.0 (0.0-0.0)
Salmonella Paratyphi	Multi-drug resistance in Salmonella Typhi and Paratyphi	0 (0-0)	0.0 (0.0-0.0)	0 (0-0)	0.0 (0.0-0.0)	0 (0-0)	0.0 (0.0-0.0)	0 (0-0)	0.0 (0.0-0.0)
Salmonella Paratyphi	Resistance to one or more antibiotics	0 (0-0)	0.0 (0.0-0.0)	4 (2-9)	0.0 (0.0-0.0)	0 (0-0)	0.0 (0.0-0.0)	1 (0-2)	0.0 (0.0-0.0)
Salmonella Typhi	Fluoroquinolones	4 (1-8)	0.0 (0.0-0.0)	325 (119-717)	1.5 (0.5-3.2)	1 (0-2)	0.0 (0.0-0.0)	66 (9-167)	0.3 (0.0-0.7)
Salmonella Typhi	Multi-drug resistance in Salmonella Typhi and Paratyphi	1 (0-3)	0.0 (0.0-0.0)	102 (38-228)	0.5 (0.2-1.0)	0 (0-0)	0.0 (0.0-0.0)	12 (0-40)	0.1 (0.0-0.2)
Salmonella Typhi	Resistance to one or more antibiotics	5 (2-10)	0.0 (0.0-0.0)	401 (159-852)	1.8 (0.7-3.8)	1 (0-2)	0.0 (0.0-0.0)	78 (17-194)	0.3 (0.1-0.9)
Non-typhoidal *Salmonella*	Fluoroquinolones	0 (0-1)	0.0 (0.0-0.0)	91 (15-293)	0.4 (0.1-1.3)	0 (0-0)	0.0 (0.0-0.0)	12 (2-37)	0.1 (0.0-0.2)
Non-typhoidal *Salmonella*	Resistance to one or more antibiotics	0 (0-1)	0.0 (0.0-0.0)	91 (15-293)	0.4 (0.1-1.3)	0 (0-0)	0.0 (0.0-0.0)	12 (2-37)	0.1 (0.0-0.2)
Serratia spp.	Aminoglycosides	2 (1-3)	0.0 (0.0-0.0)	170 (85-300)	0.8 (0.4-1.3)	0 (0-0)	0.0 (0.0-0.0)	10 (0-28)	0.0 (0.0-0.1)
Serratia spp.	Anti-pseudomonal penicillin/Beta-Lactamase inhibitors	2 (1-3)	0.0 (0.0-0.0)	146 (73-262)	0.7 (0.3-1.2)	0 (0-1)	0.0 (0.0-0.0)	27 (12-53)	0.1 (0.1-0.2)
Serratia spp.	Carbapenem	1 (0-2)	0.0 (0.0-0.0)	86 (44-153)	0.4 (0.2-0.7)	0 (0-1)	0.0 (0.0-0.0)	23 (10-44)	0.1 (0.0-0.2)
Serratia spp.	Fluoroquinolones	1 (0-1)	0.0 (0.0-0.0)	58 (28-101)	0.3 (0.1-0.5)	0 (0-0)	0.0 (0.0-0.0)	6 (1-15)	0.0 (0.0-0.1)
Serratia spp.	Fourth-generation cephalosporins	3 (2-6)	0.0 (0.0-0.0)	268 (137-487)	1.2 (0.6-2.2)	0 (0-1)	0.0 (0.0-0.0)	27 (10-59)	0.1 (0.0-0.3)
Serratia spp.	Third-generation cephalosporins	3 (2-6)	0.0 (0.0-0.0)	275 (134-499)	1.2 (0.6-2.2)	0 (0-0)	0.0 (0.0-0.0)	13 (4-31)	0.1 (0.0-0.1)
Serratia spp.	Resistance to one or more antibiotics	5 (2-9)	0.0 (0.0-0.0)	424 (218-749)	1.9 (1.0-3.4)	1 (1-2)	0.0 (0.0-0.0)	107 (50-200)	0.5 (0.2-0.9)
Shigella spp.	Fluoroquinolones	7 (2-18)	0.0 (0.0-0.1)	860 (238-2070)	3.9 (1.1-9.3)	1 (0-5)	0.0 (0.0-0.0)	156 (24-467)	0.7 (0.1-2.1)
Shigella spp.	Resistance to one or more antibiotics	7 (2-18)	0.0 (0.0-0.1)	860 (238-2070)	3.9 (1.1-9.3)	1 (0-5)	0.0 (0.0-0.0)	156 (24-467)	0.7 (0.1-2.1)
Staphylococcus aureus	Fluoroquinolones	132 (103-170)	0.6 (0.5-0.8)	11500 (8930-14800)	51.5 (40.0-66.2)	6 (3-10)	0.0 (0.0-0.0)	523 (231-868)	2.3 (1.0-3.9)
Staphylococcus aureus	Macrolide	201 (159-257)	0.9 (0.7-1.2)	17400 (13800-22200)	78.1 (61.7-99.6)	8 (3-14)	0.0 (0.0-0.1)	706 (305-1220)	3.2 (1.4-5.5)
Staphylococcus aureus	Methicillin	154 (118-200)	0.7 (0.5-0.9)	13400 (10200-17400)	59.9 (45.6-77.9)	40 (17-66)	0.2 (0.1-0.3)	3420 (1490-5700)	15.3 (6.7-25.5)
Staphylococcus aureus	Trimethoprim-Sulfamethoxazole	46 (36-58)	0.2 (0.2-0.3)	3980 (3120-5000)	17.8 (14.0-22.4)	4 (2-6)	0.0 (0.0-0.0)	366 (186-555)	1.6 (0.8-2.5)
Staphylococcus aureus	Vancomycin	4 (3-5)	0.0 (0.0-0.0)	336 (253-450)	1.5 (1.1-2.0)	1 (1-2)	0.0 (0.0-0.0)	101 (53-170)	0.5 (0.2-0.8)
Staphylococcus aureus	Resistance to one or more antibiotics	271 (214-344)	1.2 (1.0-1.5)	23500 (18600-29900)	105.2 (83.3-133.7)	59 (35-89)	0.3 (0.2-0.4)	5120 (3040-7690)	22.9 (13.6-34.4)
Streptococcus pneumoniae	Beta Lactam/Beta-lactamase inhibitors	138 (99-186)	0.6 (0.4-0.8)	11900 (8560-16100)	53.4 (38.3-72.0)	2 (0-6)	0.0 (0.0-0.0)	184 (4-514)	0.8 (0.0-2.3)
Streptococcus pneumoniae	Carbapenem	276 (206-359)	1.2 (0.9-1.6)	23900 (17900-31000)	106.9 (80.2-139.0)	61 (28-105)	0.3 (0.1-0.5)	5310 (2430-9090)	23.8 (10.9-40.7)
Streptococcus pneumoniae	Fluoroquinolones	29 (21-38)	0.1 (0.1-0.2)	2470 (1830-3260)	11.1 (8.2-14.6)	3 (1-7)	0.0 (0.0-0.0)	283 (68-576)	1.3 (0.3-2.6)
Streptococcus pneumoniae	Macrolide	275 (212-357)	1.2 (0.9-1.6)	23800 (18300-30900)	106.5 (82.0-138.4)	10 (0-27)	0.0 (0.0-0.1)	900 (0-2310)	4.0 (0.0-10.3)
Streptococcus pneumoniae	Penicillin	275 (203-362)	1.2 (0.9-1.6)	23800 (17600-31300)	106.5 (78.8-140.2)	13 (6-22)	0.1 (0.0-0.1)	1100 (550-1900)	4.9 (2.5-8.5)
Streptococcus pneumoniae	Third-generation cephalosporins	109 (81-146)	0.5 (0.4-0.7)	9410 (7000-12600)	42.1 (31.4-56.3)	3 (1-6)	0.0 (0.0-0.0)	275 (113-532)	1.2 (0.5-2.4)
Streptococcus pneumoniae	Trimethoprim-Sulfamethoxazole	620 (493-778)	2.8 (2.2-3.5)	53600 (42600-67200)	240.3 (190.7-301.1)	58 (8-115)	0.3 (0.0-0.5)	5010 (664-9950)	22.5 (3.0-44.6)
Streptococcus pneumoniae	Resistance to one or more antibiotics	724 (583-902)	3.2 (2.6-4.0)	62600 (50500-77900)	280.4 (226.2-349.0)	151 (100-216)	0.7 (0.4-1.0)	13100 (8620-18600)	58.5 (38.6-83.4)

Proteus spp.	Resistance to one or more antibiotics	6230 (3990-9070)	1.6 (1.0-2.3)	133000 (84700-194000)	34.1 (21.7-49.8)	762 (404-1290)	0.2 (0.1-0.3)	16300 (8620-27300)	4.2 (2.2-7.0)
Pseudomonas aeruginosa	Aminoglycosides	11600 (7510-17100)	3.0 (1.9-4.4)	270000 (175000-397000)	69.1 (44.8-101.8)	279 (0-717)	0.1 (0.0-0.2)	6570 (23-16600)	1.7 (0.0-4.3)
Pseudomonas aeruginosa	Anti-pseudomonal penicillin/Beta-Lactamase inhibitors	15700 (10300-22800)	4.0 (2.6-5.8)	367000 (241000-530000)	94.0 (61.8-135.9)	824 (477-1350)	0.2 (0.1-0.3)	19300 (11100-31300)	4.9 (2.8-8.0)
Pseudomonas aeruginosa	Carbapenem	17700 (11500-25900)	4.5 (2.9-6.6)	411000 (268000-598000)	105.4 (68.6-153.3)	2980 (1800-4630)	0.8 (0.5-1.2)	69400 (42100-108000)	17.8 (10.8-27.6)
Pseudomonas aeruginosa	Fluoroquinolones	16600 (10900-24400)	4.2 (2.8-6.2)	386000 (253000-568000)	99.0 (64.7-145.7)	1730 (1030-2750)	0.4 (0.3-0.7)	40600 (24200-64300)	10.4 (6.2-16.5)
Pseudomonas aeruginosa	Fourth-generation cephalosporins	14100 (9230-20600)	3.6 (2.4-5.3)	330000 (216000-478000)	84.7 (55.4-122.6)	210 (115-339)	0.1 (0.0-0.1)	4930 (2750-7910)	1.3 (0.7-2.0)
Pseudomonas aeruginosa	Third-generation cephalosporins	16200 (10400-23500)	4.1 (2.7-6.0)	382000 (247000-553000)	97.9 (63.2-141.7)	406 (166-740)	0.1 (0.0-0.2)	9930 (4100-18200)	2.5 (1.1-4.7)
Pseudomonas aeruginosa	Resistance to one or more antibiotics	25200 (16600-36600)	6.5 (4.2-9.4)	590000 (387000-852000)	151.2 (99.2-218.4)	6430 (3890-10100)	1.6 (1.0-2.6)	151000 (91100-235000)	38.6 (23.4-60.3)
Salmonella Paratyphi	Fluoroquinolones	7 (1-13)	0.0 (0.0-0.0)	141 (20-247)	0.0 (0.0-0.1)	1 (0-3)	0.0 (0.0-0.0)	28 (2-63)	0.0 (0.0-0.0)
Salmonella Paratyphi	Multi-drug resistance in Salmonella Typhi and Paratyphi	0 (0-0)	0.0 (0.0-0.0)	1 (0-2)	0.0 (0.0-0.0)	0 (0-0)	0.0 (0.0-0.0)	0 (0-0)	0.0 (0.0-0.0)
Salmonella Paratyphi	Resistance to one or more antibiotics	7 (1-13)	0.0 (0.0-0.0)	141 (21-248)	0.0 (0.0-0.1)	1 (0-3)	0.0 (0.0-0.0)	28 (2-63)	0.0 (0.0-0.0)
Salmonella Typhi	Fluoroquinolones	351 (117-835)	0.1 (0.0-0.2)	12000 (4100-28200)	3.1 (1.1-7.2)	71 (8-203)	0.0 (0.0-0.1)	2430 (295-6840)	0.6 (0.1-1.8)
Salmonella Typhi	Multi-drug resistance in Salmonella Typhi and Paratyphi	124 (42-303)	0.0 (0.0-0.1)	4240 (1400-10100)	1.1 (0.4-2.6)	15 (0-54)	0.0 (0.0-0.0)	506 (0-1830)	0.1 (0.0-0.5)
Salmonella Typhi	Resistance to one or more antibiotics	446 (164-1010)	0.1 (0.0-0.3)	15200 (5510-33700)	3.9 (1.4-8.6)	86 (16-230)	0.0 (0.0-0.1)	2940 (534-7670)	0.8 (0.1-2.0)
Non-typhoidal *Salmonella*	Fluoroquinolones	43 (18-90)	0.0 (0.0-0.0)	1490 (529-3420)	0.4 (0.1-0.9)	9 (1-24)	0.0 (0.0-0.0)	265 (43-678)	0.1 (0.0-0.2)
Non-typhoidal *Salmonella*	Resistance to one or more antibiotics	43 (18-90)	0.0 (0.0-0.0)	1490 (529-3420)	0.4 (0.1-0.9)	9 (1-24)	0.0 (0.0-0.0)	265 (43-678)	0.1 (0.0-0.2)
Serratia spp.	Aminoglycosides	961 (556-1470)	0.2 (0.1-0.4)	24800 (14200-38000)	6.4 (3.6-9.8)	57 (0-151)	0.0 (0.0-0.0)	1480 (0-3940)	0.4 (0.0-1.0)
Serratia spp.	Anti-pseudomonal penicillin/Beta-Lactamase inhibitors	746 (420-1180)	0.2 (0.1-0.3)	19400 (10800-30600)	5.0 (2.8-7.8)	134 (64-243)	0.0 (0.0-0.1)	3470 (1670-6310)	0.9 (0.4-1.6)
Serratia spp.	Carbapenem	466 (273-735)	0.1 (0.1-0.2)	12000 (6910-19100)	3.1 (1.8-4.9)	122 (61-219)	0.0 (0.0-0.1)	3140 (1540-5620)	0.8 (0.4-1.4)
Serratia spp.	Fluoroquinolones	193 (110-303)	0.0 (0.0-0.1)	5060 (2860-8020)	1.3 (0.7-2.1)	21 (4-47)	0.0 (0.0-0.0)	564 (104-1240)	0.1 (0.0-0.3)
Serratia spp.	Fourth-generation cephalosporins	1490 (883-2370)	0.4 (0.2-0.6)	37900 (22200-60900)	9.7 (5.7-15.6)	159 (67-308)	0.0 (0.0-0.1)	3970 (1640-7730)	1.0 (0.4-2.0)
Serratia spp.	Third-generation cephalosporins	1410 (820-2240)	0.4 (0.2-0.6)	36200 (20500-57100)	9.3 (5.3-14.6)	57 (19-117)	0.0 (0.0-0.0)	1470 (502-3060)	0.4 (0.1-0.8)
Serratia spp.	Resistance to one or more antibiotics	2180 (1280-3380)	0.6 (0.3-0.9)	55900 (32600-87200)	14.3 (8.3-22.3)	550 (296-915)	0.1 (0.1-0.2)	14100 (7610-23900)	3.6 (2.0-6.1)
Shigella spp.	Fluoroquinolones	10 (3-25)	0.0 (0.0-0.0)	2220 (803-5070)	0.6 (0.2-1.3)	2 (0-6)	0.0 (0.0-0.0)	285 (0-892)	0.1 (0.0-0.2)
Shigella spp.	Resistance to one or more antibiotics	10 (3-25)	0.0 (0.0-0.0)	2220 (803-5070)	0.6 (0.2-1.3)	2 (0-6)	0.0 (0.0-0.0)	285 (0-892)	0.1 (0.0-0.2)
Staphylococcus aureus	Fluoroquinolones	15700 (10400-22900)	4.0 (2.7-5.9)	388000 (255000-563000)	99.4 (65.5-144.3)	768 (318-1370)	0.2 (0.1-0.4)	18900 (7860-33700)	4.8 (2.0-8.6)
Staphylococcus aureus	Macrolide	19900 (13500-28200)	5.1 (3.5-7.2)	474000 (321000-675000)	121.5 (82.3-173.0)	824 (313-1540)	0.2 (0.1-0.4)	19500 (7460-36800)	5.0 (1.9-9.4)
Staphylococcus aureus	Methicillin	20300 (13500-29400)	5.2 (3.5-7.5)	489000 (326000-717000)	125.4 (83.6-183.8)	5430 (2300-9470)	1.4 (0.6-2.4)	130000 (55800-227000)	33.4 (14.3-58.2)
Staphylococcus aureus	Trimethoprim-Sulfamethoxazole	2340 (1580-3360)	0.6 (0.4-0.9)	60800 (40800-87000)	15.6 (10.5-22.3)	219 (106-363)	0.1 (0.0-0.1)	5660 (2710-9360)	1.4 (0.7-2.4)
Staphylococcus aureus	Vancomycin	563 (363-835)	0.1 (0.1-0.2)	13900 (8950-20600)	3.6 (2.3-5.3)	180 (88-337)	0.0 (0.0-0.1)	4420 (2170-8220)	1.1 (0.6-2.1)
Staphylococcus aureus	Resistance to one or more antibiotics	31200 (21000-44100)	8.0 (5.4-11.3)	752000 (505000-1070000)	192.8 (129.5-273.5)	7420 (3890-12500)	1.9 (1.0-3.2)	179000 (94800-298000)	45.9 (24.3-76.4)
Streptococcus pneumoniae	Beta Lactam/Beta-lactamase inhibitors	2200 (1560-3080)	0.6 (0.4-0.8)	62700 (45400-85400)	16.1 (11.6-21.9)	29 (1-71)	0.0 (0.0-0.0)	841 (32-2090)	0.2 (0.0-0.5)
Streptococcus pneumoniae	Carbapenem	4990 (3560-6940)	1.3 (0.9-1.8)	137000 (101000-185000)	35.0 (25.9-47.5)	1180 (560-2030)	0.3 (0.1-0.5)	32200 (15300-54400)	8.3 (3.9-13.9)
Streptococcus pneumoniae	Fluoroquinolones	982 (688-1400)	0.3 (0.2-0.4)	26300 (18900-36900)	6.8 (4.8-9.5)	127 (31-268)	0.0 (0.0-0.1)	3330 (819-6930)	0.9 (0.2-1.8)
Streptococcus pneumoniae	Macrolide	5920 (4310-8120)	1.5 (1.1-2.1)	160000 (119000-215000)	41.0 (30.6-55.1)	243 (0-635)	0.1 (0.0-0.2)	6400 (0-16600)	1.6 (0.0-4.3)
Streptococcus pneumoniae	Penicillin	7240 (5140-10200)	1.9 (1.3-2.6)	200000 (146000-275000)	51.3 (37.5-70.6)	438 (217-766)	0.1 (0.1-0.2)	12000 (5980-20900)	3.1 (1.5-5.4)
Streptococcus pneumoniae	Third-generation cephalosporins	4220 (2950-5860)	1.1 (0.8-1.5)	118000 (84200-161000)	30.2 (21.6-41.3)	342 (131-668)	0.1 (0.0-0.2)	9680 (3840-18600)	2.5 (1.0-4.8)
Streptococcus pneumoniae	Trimethoprim-Sulfamethoxazole	16000 (11900-21300)	4.1 (3.1-5.5)	462000 (358000-601000)	118.5 (91.8-154.1)	1580 (220-3250)	0.4 (0.1-0.8)	45500 (6630-92700)	11.7 (1.7-23.8)
Streptococcus pneumoniae	Resistance to one or more antibiotics	19900 (14900-26400)	5.1 (3.8-6.8)	561000 (432000-721000)	143.7 (110.8-184.9)	3950 (2440-5820)	1.0 (0.6-1.5)	110000 (68500-159000)	28.2 (17.6-40.8)

Table S15. Deaths and DALYs (in counts and age-specific rates) associated with and attributable to bacterial antimicrobial resistance by pathogen–drug combination, by age group, 2019, high-income super-region

Pathogen	Antibiotic class	Neonatal								Post Neonatal								1 to 4								5 and over							
		Associated with resistance				Attributable to resistance				Associated with resistance				Attributable to resistance				Associated with resistance				Attributable to resistance				Associated with resistance				Attributable to resistance			
		Deaths		DALYs		Deaths		DALYs		Deaths		DALYs		Deaths		DALYs		Deaths		DALYs		Deaths		DALYs		Deaths		DALYs		Deaths		DALYs	
		Counts	Rate per 100 000	Counts	Rate per 100 000	Counts	Rate per 100 000	Counts	Rate per 100 000	Counts	Rate per 100 000	Counts	Rate per 100 000	Counts	Rate per 100 000	Counts	Rate per 100 000	Counts	Rate per 100 000	Counts	Rate per 100 000	Counts	Rate per 100 000	Counts	Rate per 100 000	Counts	Rate per 100 000	Counts	Rate per 100 000	Counts	Rate per 100 000	Counts	Rate per 100 000
All pathogens	Resistance to one or more antibiotics	1910 (1200-2890)	223.5 (140.7-338.4)	170000 (107000-257000)	19894.9 (12534.5-30106.6)	458 (285-706)	53.5 (33.3-82.6)	40700 (25300-63000)	4763.7 (2963.1-7362.3)	1310 (891-1850)	12.8 (8.7-18.0)	118000 (80800-167000)	1149.8 (785.6-1627.9)	305 (206-445)	3.0 (2.0-4.3)	27300 (18400-39800)	265.7 (178.7-386.6)	567 (371-829)	1.2 (0.8-1.8)	52700 (35600-75700)	115.1 (77.8-165.3)	130 (85-196)	0.3 (0.2-0.4)	11700 (7810-17500)	25.6 (17.0-38.2)	600000 (432000-819000)	58.4 (42.0-79.7)	9920000 (6830000-13900000)	966.0 (665.4-1354.5)	140000 (98100-196000)	13.6 (9.6-19.1)	2330000 (1590000-3320000)	226.9 (154.4-322.9)
Acinetobacter baumannii	Aminoglycosides	74 (46-116)	8.7 (5.3-13.6)	6620 (4040-10300)	774.4 (473.0-1204.7)	3 (0-8)	0.4 (0.0-0.9)	285 (0-694)	33.4 (0.0-81.1)	33 (18-54)	0.3 (0.2-0.5)	2910 (1630-4770)	28.3 (15.9-46.3)	1 (0-4)	0.0 (0.0-0.0)	126 (0-317)	1.2 (0.0-3.1)	12 (6-22)	0.0 (0.0-0.0)	1070 (524-1940)	2.3 (1.1-4.2)	1 (0-1)	0.0 (0.0-0.0)	48 (1-126)	0.1 (0.0-0.3)	14100 (8210-23300)	1.4 (0.8-2.3)	259000 (149000-419000)	25.2 (14.5-40.8)	644 (0-1660)	0.1 (0.0-0.2)	11800 (109-30500)	1.1 (0.0-3.0)
Acinetobacter baumannii	Anti-pseudomonal penicillin/Beta-Lactamase inhibitors	114 (69-175)	13.3 (8.1-20.5)	10100 (6180-15600)	1183.7 (722.2-1821.2)	3 (2-6)	0.4 (0.2-0.7)	298 (143-542)	34.9 (16.7-63.4)	50 (28-78)	0.5 (0.3-0.8)	4400 (2500-6920)	42.8 (24.3-67.3)	1 (1-3)	0.0 (0.0-0.0)	125 (59-224)	1.2 (0.6-2.2)	19 (9-34)	0.0 (0.0-0.1)	1660 (796-2970)	3.6 (1.7-6.5)	1 (0-1)	0.0 (0.0-0.0)	61 (25-113)	0.1 (0.1-0.2)	22600 (13000-36000)	2.2 (1.3-3.5)	411000 (237000-661000)	40.0 (23.1-64.3)	1090 (503-1930)	0.1 (0.0-0.2)	18700 (8750-33200)	1.8 (0.9-3.2)
Acinetobacter baumannii	Beta Lactam/Beta-lactamase inhibitors	96 (59-151)	11.2 (6.9-17.7)	8550 (5210-13500)	999.8 (609.1-1574.3)	1 (0-2)	0.1 (0.0-0.2)	73 (20-151)	8.5 (2.3-17.7)	44 (25-70)	0.4 (0.2-0.7)	3890 (2210-6210)	37.8 (21.5-60.4)	0 (0-1)	0.0 (0.0-0.0)	37 (10-79)	0.4 (0.1-0.8)	18 (9-32)	0.0 (0.0-0.1)	1580 (778-2820)	3.4 (1.7-6.1)	0 (0-1)	0.0 (0.0-0.0)	22 (5-48)	0.0 (0.0-0.1)	25200 (14800-41000)	2.5 (1.4-4.0)	437000 (254000-699000)	42.6 (24.8-68.0)	501 (138-1020)	0.0 (0.0-0.1)	7900 (2210-16100)	0.8 (0.2-1.6)
Acinetobacter baumannii	Carbapenem	107 (65-166)	12.6 (7.6-19.4)	9540 (5750-14800)	1115.6 (672.9-1727.8)	18 (9-31)	2.1 (1.1-3.7)	1630 (824-2790)	190.9 (96.4-326.1)	47 (26-75)	0.5 (0.3-0.7)	4150 (2310-6620)	40.3 (22.5-64.3)	8 (4-14)	0.1 (0.0-0.1)	712 (351-1230)	6.9 (3.4-12.0)	18 (9-31)	0.0 (0.0-0.0)	1520 (745-2700)	3.3 (1.6-5.9)	3 (1-6)	0.0 (0.0-0.0)	263 (114-512)	0.6 (0.2-1.1)	19500 (11600-31100)	1.9 (1.1-3.0)	361000 (210000-577000)	35.2 (20.5-56.2)	3450 (1790-6130)	0.3 (0.2-0.6)	63200 (32600-113000)	6.2 (3.2-11.0)
Acinetobacter baumannii	Fluoroquinolones	105 (64-162)	12.3 (7.5-18.9)	9310 (5700-14400)	1089.3 (666.3-1683.2)	13 (7-23)	1.5 (0.8-2.7)	1160 (591-2030)	136.2 (69.1-237.7)	46 (26-72)	0.4 (0.2-0.7)	4030 (2270-6410)	39.2 (22.1-62.3)	6 (3-10)	0.1 (0.0-0.1)	505 (239-865)	4.9 (2.3-8.4)	17 (8-30)	0.0 (0.0-0.1)	1460 (712-2610)	3.2 (1.6-5.7)	2 (1-4)	0.0 (0.0-0.0)	183 (76-340)	0.4 (0.2-0.7)	18400 (10700-29400)	1.8 (1.0-2.9)	343000 (197000-545000)	33.4 (19.2-53.1)	2330 (1120-3970)	0.2 (0.1-0.4)	43200 (21000-74400)	4.2 (2.0-7.2)
Acinetobacter baumannii	Fourth-generation cephalosporins	112 (68-176)	13.1 (7.9-20.6)	9960 (6040-15700)	1164.9 (706.4-1831.0)	1 (0-2)	0.1 (0.0-0.2)	70 (28-146)	8.2 (3.3-17.0)	49 (28-79)	0.5 (0.3-0.8)	4320 (2440-6950)	42.0 (23.8-67.6)	0 (0-1)	0.0 (0.0-0.0)	30 (12-61)	0.3 (0.1-0.6)	19 (9-33)	0.0 (0.0-0.1)	1620 (786-2900)	3.5 (1.7-6.3)	0 (0-0)	0.0 (0.0-0.0)	14 (5-29)	0.0 (0.0-0.1)	21800 (12500-34900)	2.1 (1.2-3.4)	399000 (228000-646000)	38.8 (22.2-62.9)	257 (99-526)	0.0 (0.0-0.1)	4430 (1770-9030)	0.4 (0.2-0.9)
Acinetobacter baumannii	Third-generation cephalosporins	142 (85-220)	16.6 (9.9-25.8)	12600 (7550-19600)	1474.2 (883.1-2290.4)	8 (3-15)	0.9 (0.4-1.8)	721 (290-1350)	84.3 (33.9-157.9)	62 (35-99)	0.6 (0.3-1.0)	5460 (3060-8770)	53.1 (29.7-85.2)	3 (1-6)	0.0 (0.0-0.1)	304 (116-565)	3.0 (1.1-5.5)	25 (12-44)	0.1 (0.0-0.1)	2170 (1060-3860)	4.7 (2.3-8.4)	2 (1-3)	0.0 (0.0-0.0)	144 (51-282)	0.3 (0.1-0.6)	31300 (18000-50800)	3.0 (1.8-4.9)	561000 (321000-895000)	54.7 (31.3-87.1)	2440 (1020-4580)	0.2 (0.1-0.4)	42300 (17500-79700)	4.1 (1.7-7.8)
Acinetobacter baumannii	Resistance to one or more antibiotics	155 (93-240)	18.2 (10.9-28.1)	13800 (8280-21400)	1614.4 (968.3-2499.4)	48 (27-78)	5.6 (3.2-9.1)	4250 (2420-6920)	496.5 (282.6-809.4)	68 (38-108)	0.7 (0.4-1.1)	5990 (3350-9580)	58.3 (32.6-93.1)	21 (11-34)	0.2 (0.1-0.3)	1840 (983-3030)	17.9 (9.6-29.4)	28 (14-50)	0.1 (0.0-0.1)	2430 (1180-4330)	5.3 (2.6-9.5)	8 (4-15)	0.0 (0.0-0.0)	733 (340-1340)	1.6 (0.7-2.9)	36100 (21000-58300)	3.5 (2.0-5.7)	643000 (372000-1030000)	62.6 (36.3-99.9)	10700 (5890-17500)	1.0 (0.6-1.7)	192000 (104000-316000)	18.6 (10.1-30.8)
Citrobacter spp.	Aminoglycosides	1 (1-2)	0.1 (0.1-0.2)	93 (50-161)	10.9 (5.9-18.8)	0 (0-0)	0.0 (0.0-0.0)	6 (0-15)	0.7 (0.0-1.7)	1 (1-2)	0.0 (0.0-0.0)	106 (55-184)	1.0 (0.5-1.8)	0 (0-0)	0.0 (0.0-0.0)	6 (0-17)	0.1 (0.0-0.2)	0 (0-1)	0.0 (0.0-0.0)	39 (17-72)	0.1 (0.0-0.2)	0 (0-0)	0.0 (0.0-0.0)	2 (0-6)	0.0 (0.0-0.0)	192 (126-281)	0.0 (0.0-0.0)	3690 (2340-5470)	0.4 (0.2-0.5)	12 (0-29)	0.0 (0.0-0.0)	231 (0-566)	0.0 (0.0-0.1)
Citrobacter spp.	Anti-pseudomonal penicillin/Beta-Lactamase inhibitors	5 (3-9)	0.6 (0.3-1.1)	457 (244-819)	53.5 (28.5-95.7)	1 (0-2)	0.1 (0.1-0.3)	103 (43-207)	12.1 (5.1-24.2)	6 (3-11)	0.1 (0.0-0.1)	548 (280-942)	5.3 (2.7-9.2)	1 (1-3)	0.0 (0.0-0.0)	124 (54-243)	1.2 (0.5-2.4)	2 (1-5)	0.0 (0.0-0.0)	213 (94-397)	0.5 (0.2-0.9)	1 (0-1)	0.0 (0.0-0.0)	49 (19-100)	0.1 (0.0-0.2)	1230 (805-1830)	0.1 (0.1-0.2)	22100 (14000-33500)	2.1 (1.4-3.3)	290 (150-499)	0.0 (0.0-0.0)	5150 (2630-9040)	0.5 (0.3-0.9)
Citrobacter spp.	Carbapenem	1 (1-2)	0.2 (0.1-0.3)	117 (63-203)	13.7 (7.4-23.7)	0 (0-1)	0.0 (0.0-0.1)	30 (13-59)	3.5 (1.6-6.9)	2 (1-3)	0.0 (0.0-0.0)	137 (69-236)	1.3 (0.7-2.3)	0 (0-1)	0.0 (0.0-0.0)	35 (15-69)	0.3 (0.1-0.7)	1 (0-1)	0.0 (0.0-0.0)	47 (20-90)	0.1 (0.0-0.2)	0 (0-0)	0.0 (0.0-0.0)	12 (5-26)	0.0 (0.0-0.1)	241 (154-363)	0.0 (0.0-0.0)	4430 (2740-6830)	0.4 (0.3-0.7)	64 (34-109)	0.0 (0.0-0.0)	1170 (613-2020)	0.1 (0.1-0.2)
Citrobacter spp.	Fluoroquinolones	4 (2-7)	0.5 (0.3-0.8)	351 (194-584)	41.0 (22.7-68.3)	1 (0-1)	0.1 (0.0-0.1)	58 (23-112)	6.8 (2.6-13.1)	5 (2-8)	0.0 (0.0-0.1)	408 (217-704)	4.0 (2.1-6.8)	1 (0-2)	0.0 (0.0-0.0)	68 (26-133)	0.7 (0.3-1.3)	2 (1-3)	0.0 (0.0-0.0)	138 (62-258)	0.3 (0.1-0.6)	0 (0-1)	0.0 (0.0-0.0)	23 (8-47)	0.0 (0.0-0.1)	666 (433-974)	0.1 (0.0-0.1)	12600 (8020-18600)	1.2 (0.8-1.8)	108 (48-196)	0.0 (0.0-0.0)	2040 (918-3730)	0.2 (0.1-0.4)
Citrobacter spp.	Fourth-generation cephalosporins	2 (1-3)	0.2 (0.1-0.4)	176 (95-309)	20.5 (11.1-36.1)	0 (0-0)	0.0 (0.0-0.0)	12 (5-24)	1.4 (0.6-2.9)	2 (1-4)	0.0 (0.0-0.0)	197 (99-342)	1.9 (1.0-3.3)	0 (0-0)	0.0 (0.0-0.0)	13 (5-26)	0.1 (0.0-0.3)	1 (0-1)	0.0 (0.0-0.0)	67 (29-126)	0.1 (0.1-0.3)	0 (0-0)	0.0 (0.0-0.0)	4 (1-9)	0.0 (0.0-0.0)	312 (198-462)	0.0 (0.0-0.0)	5750 (3510-8670)	0.6 (0.3-0.8)	18 (8-34)	0.0 (0.0-0.0)	339 (154-627)	0.0 (0.0-0.1)
Citrobacter spp.	Third-generation cephalosporins	8 (4-14)	1.0 (0.5-1.7)	725 (378-1280)	84.8 (44.2-149.4)	1 (0-2)	0.1 (0.0-0.2)	68 (24-144)	8.0 (2.9-16.8)	10 (5-17)	0.1 (0.0-0.2)	847 (430-1470)	8.2 (4.2-14.3)	1 (0-2)	0.0 (0.0-0.0)	79 (29-164)	0.8 (0.3-1.6)	4 (2-7)	0.0 (0.0-0.0)	314 (138-596)	0.7 (0.3-1.3)	0 (0-1)	0.0 (0.0-0.0)	29 (9-63)	0.1 (0.0-0.1)	1690 (1090-2520)	0.2 (0.1-0.2)	31000 (19300-46600)	3.0 (1.9-4.5)	151 (63-275)	0.0 (0.0-0.0)	2820 (1160-5180)	0.3 (0.1-0.5)
Citrobacter spp.	Resistance to one or more antibiotics	11 (6-19)	1.3 (0.7-2.2)	961 (519-1670)	112.4 (60.7-195.8)	3 (2-6)	0.4 (0.2-0.7)	277 (135-508)	32.4 (15.8-59.5)	13 (7-22)	0.1 (0.1-0.2)	1130 (585-1940)	11.0 (5.7-18.9)	4 (2-7)	0.0 (0.0-0.1)	325 (154-599)	3.2 (1.5-5.8)	5 (2-9)	0.0 (0.0-0.0)	416 (182-775)	0.9 (0.4-1.7)	1 (1-3)	0.0 (0.0-0.0)	119 (48-239)	0.3 (0.1-0.5)	2240 (1470-3260)	0.2 (0.1-0.3)	41000 (26100-60400)	4.0 (2.5-5.9)	643 (372-1030)	0.1 (0.0-0.1)	11800 (6650-19000)	1.1 (0.6-1.8)
Enterobacter spp.	Aminoglycosides	14 (8-22)	1.6 (1.0-2.5)	1210 (727-1940)	141.0 (85.0-226.4)	1 (0-2)	0.1 (0.0-0.2)	82 (23-168)	9.6 (2.7-19.7)	4 (2-6)	0.0 (0.0-0.1)	328 (196-520)	3.2 (1.9-5.1)	0 (0-1)	0.0 (0.0-0.0)	22 (7-46)	0.2 (0.1-0.4)	1 (1-2)	0.0 (0.0-0.0)	131 (74-216)	0.3 (0.2-0.5)	0 (0-0)	0.0 (0.0-0.0)	9 (3-19)	0.0 (0.0-0.0)	1440 (970-2090)	0.1 (0.1-0.2)	27000 (17500-39700)	2.6 (1.7-3.9)	103 (29-201)	0.0 (0.0-0.0)	1910 (565-3770)	0.2 (0.1-0.4)
Enterobacter spp.	Anti-pseudomonal penicillin/Beta-Lactamase inhibitors	77 (47-122)	9.0 (5.5-14.3)	6840 (4200-10900)	799.8 (491.4-1269.5)	10 (5-17)	1.2 (0.6-2.0)	894 (475-1540)	104.5 (55.6-179.6)	22 (13-34)	0.2 (0.1-0.3)	1970 (1180-3050)	19.1 (11.5-29.7)	3 (1-5)	0.0 (0.0-0.0)	258 (130-433)	2.5 (1.3-4.2)	9 (5-15)	0.0 (0.0-0.0)	827 (479-1330)	1.8 (1.0-2.9)	1 (1-2)	0.0 (0.0-0.0)	110 (56-192)	0.2 (0.1-0.4)	10800 (7400-14900)	1.0 (0.7-1.4)	189000 (125000-268000)	18.4 (12.2-26.1)	1470 (842-2250)	0.1 (0.1-0.2)	25700 (14300-40300)	2.5 (1.4-3.9)
Enterobacter spp.	Carbapenem	33 (20-51)	3.8 (2.4-6.0)	2920 (1790-4560)	341.7 (209.2-533.5)	9 (5-15)	1.0 (0.6-1.7)	789 (433-1320)	92.3 (50.7-154.6)	9 (6-14)	0.1 (0.1-0.1)	833 (501-1270)	8.1 (4.9-12.4)	3 (1-4)	0.0 (0.0-0.0)	226 (124-369)	2.2 (1.2-3.6)	4 (2-6)	0.0 (0.0-0.0)	337 (195-536)	0.7 (0.4-1.2)	1 (1-2)	0.0 (0.0-0.0)	92 (49-156)	0.2 (0.1-0.3)	4060 (2820-5710)	0.4 (0.3-0.6)	72000 (48200-103000)	7.0 (4.7-10.1)	1120 (693-1680)	0.1 (0.1-0.2)	19700 (11800-30100)	1.9 (1.2-2.9)
Enterobacter spp.	Fluoroquinolones	28 (18-45)	3.3 (2.1-5.3)	2520 (1560-4000)	294.6 (182.8-467.4)	3 (2-6)	0.4 (0.2-0.7)	278 (144-509)	32.5 (16.8-59.5)	8 (5-12)	0.1 (0.0-0.1)	706 (426-1110)	6.9 (4.1-10.7)	1 (0-2)	0.0 (0.0-0.0)	78 (39-139)	0.8 (0.4-1.4)	3 (2-5)	0.0 (0.0-0.0)	282 (163-461)	0.6 (0.4-1.0)	0 (0-1)	0.0 (0.0-0.0)	31 (15-57)	0.1 (0.0-0.1)	3340 (2290-4710)	0.3 (0.2-0.5)	60400 (40000-87000)	5.9 (3.9-8.5)	376 (209-636)	0.0 (0.0-0.1)	6760 (3640-11600)	0.7 (0.4-1.1)
Enterobacter spp.	Fourth-generation cephalosporins	48 (30-75)	5.7 (3.5-8.8)	4300 (2660-6700)	503.1 (311.3-783.5)	2 (1-3)	0.2 (0.1-0.4)	164 (73-309)	19.1 (8.5-36.1)	14 (8-21)	0.1 (0.1-0.2)	1210 (727-1890)	11.8 (7.1-18.3)	1 (0-1)	0.0 (0.0-0.0)	47 (21-86)	0.5 (0.2-0.8)	5 (3-9)	0.0 (0.0-0.0)	484 (284-781)	1.1 (0.6-1.7)	0 (0-0)	0.0 (0.0-0.0)	18 (8-34)	0.0 (0.0-0.1)	5630 (3850-7840)	0.5 (0.4-0.8)	101000 (66200-144000)	9.8 (6.4-14.0)	209 (101-360)	0.0 (0.0-0.0)	3680 (1750-6350)	0.4 (0.2-0.6)
Enterobacter spp.	Trimethoprim-Sulfamethoxazole	37 (22-59)	4.3 (2.6-6.9)	3260 (1980-5220)	381.8 (231.2-610.9)	2 (0-5)	0.3 (0.0-0.6)	194 (0-428)	22.7 (0.0-50.0)	10 (6-16)	0.1 (0.1-0.2)	925 (545-1420)	9.0 (5.3-13.8)	1 (0-1)	0.0 (0.0-0.0)	55 (0-119)	0.5 (0.0-1.2)	4 (2-7)	0.0 (0.0-0.0)	384 (222-611)	0.8 (0.5-1.3)	0 (0-1)	0.0 (0.0-0.0)	23 (0-50)	0.1 (0.0-0.1)	4660 (3190-6530)	0.5 (0.3-0.6)	83200 (54600-119000)	8.1 (5.3-11.6)	286 (0-590)	0.0 (0.0-0.0)	5060 (0-10600)	0.5 (0.0-1.0)
Enterobacter spp.	Resistance to one or more antibiotics	112 (70-174)	13.2 (8.2-20.4)	10000 (6210-15500)	1169.5 (726.7-1812.3)	27 (16-42)	3.2 (1.9-5.0)	2400 (1470-3770)	280.6 (171.4-440.8)	32 (19-49)	0.3 (0.2-0.5)	2870 (1730-4370)	27.9 (16.8-42.5)	8 (5-12)	0.1 (0.0-0.1)	686 (401-1060)	6.7 (3.9-10.3)	14 (8-22)	0.0 (0.0-0.0)	1190 (700-1920)	2.6 (1.5-4.2)	3 (2-5)	0.0 (0.0-0.0)	283 (159-461)	0.6 (0.3-1.0)	15100 (10500-21000)	1.5 (1.0-2.0)	267000 (178000-376000)	26.0 (17.3-36.6)	3560 (2350-5080)	0.3 (0.2-0.5)	62900 (40500-91700)	6.1 (3.9-8.9)
Enterococcus faecalis	Fluoroquinolones	41 (26-62)	4.7 (3.0-7.2)	3610 (2290-5510)	421.7 (267.7-644.6)	10 (5-18)	1.2 (0.5-2.1)	898 (412-1630)	105.1 (48.2-190.3)	16 (9-28)	0.2 (0.1-0.3)	1410 (770-2470)	13.7 (7.5-24.0)	4 (2-8)	0.0 (0.0-0.1)	348 (145-674)	3.4 (1.4-6.6)	6 (4-11)	0.0 (0.0-0.0)	609 (345-1020)	1.3 (0.8-2.2)	2 (1-3)	0.0 (0.0-0.0)	143 (61-266)	0.3 (0.1-0.6)	11600 (7110-18100)	1.1 (0.7-1.8)	211000 (131000-319000)	20.6 (12.7-31.1)	2890 (1340-5250)	0.3 (0.1-0.5)	52300 (24500-93900)	5.1 (2.4-9.1)
Enterococcus faecalis	Vancomycin	6 (3-9)	0.6 (0.4-1.1)	492 (296-818)	57.5 (34.6-95.6)	2 (1-4)	0.2 (0.1-0.4)	165 (70-314)	19.3 (8.2-36.7)	2 (1-4)	0.0 (0.0-0.0)	203 (105-368)	2.0 (1.0-3.6)	1 (0-2)	0.0 (0.0-0.0)	68 (28-146)	0.7 (0.3-1.4)	1 (0-2)	0.0 (0.0-0.0)	83 (46-146)	0.2 (0.1-0.3)	0 (0-1)	0.0 (0.0-0.0)	28 (13-57)	0.1 (0.0-0.1)	1360 (794-2220)	0.1 (0.1-0.2)	26000 (15300-42000)	2.5 (1.5-4.1)	461 (201-905)	0.0 (0.0-0.1)	8810 (3900-17100)	0.9 (0.4-1.7)
Enterococcus faecalis	Resistance to one or more antibiotics	44 (28-67)	5.1 (3.2-7.8)	3870 (2450-5920)	452.9 (286.5-692.9)	12 (6-21)	1.4 (0.7-2.5)	1060 (543-1870)	124.3 (63.5-218.3)	17 (9-30)	0.2 (0.1-0.3)	1520 (833-2690)	14.8 (8.1-26.1)	5 (2-9)	0.0 (0.0-0.1)	416 (190-789)	4.0 (1.9-7.7)	7 (4-12)	0.0 (0.0-0.0)	656 (372-1100)	1.4 (0.8-2.4)	2 (1-3)	0.0 (0.0-0.0)	172 (80-307)	0.4 (0.2-0.7)	12300 (7560-19400)	1.2 (0.7-1.9)	225000 (139000-343000)	21.9 (13.6-33.4)	3350 (1670-5890)	0.3 (0.2-0.6)	61100 (30700-106000)	6.0 (3.0-10.3)
Enterococcus faecium	Fluoroquinolones	97 (62-151)	11.4 (7.3-17.7)	8640 (5530-13500)	1010.6 (646.1-1574.1)	16 (5-31)	1.9 (0.6-3.6)	1410 (436-2730)	164.9 (50.9-319.2)	65 (35-110)	0.6 (0.3-1.1)	5770 (3090-9750)	56.1 (30.0-94.8)	11 (3-23)	0.1 (0.0-0.2)	939 (261-2010)	9.1 (2.5-19.5)	26 (13-46)	0.1 (0.0-0.1)	2220 (1090-3940)	4.8 (2.4-8.6)	4 (1-9)	0.0 (0.0-0.0)	370 (100-815)	0.8 (0.2-1.8)	45500 (28700-67300)	4.4 (2.8-6.6)	857000 (536000-1270000)	83.4 (52.2-123.8)	8030 (2330-15300)	0.8 (0.2-1.5)	149000 (43200-284000)	14.5 (4.2-27.6)
Enterococcus faecium	Vancomycin	56 (35-90)	6.6 (4.1-10.5)	5010 (3150-7960)	586.1 (368.3-931.2)	13 (6-24)	1.6 (0.8-2.8)	1190 (573-2110)	139.6 (67.1-247.2)	38 (21-64)	0.4 (0.2-0.6)	3370 (1820-5720)	32.8 (17.7-55.7)	9 (4-17)	0.1 (0.0-0.2)	803 (360-1510)	7.8 (3.5-14.7)	14 (7-24)	0.0 (0.0-0.1)	1180 (591-2090)	2.6 (1.3-4.6)	3 (1-6)	0.0 (0.0-0.0)	282 (119-532)	0.6 (0.3-1.2)	19200 (12100-28500)	1.9 (1.2-2.8)	391000 (243000-581000)	38.1 (23.6-56.6)	4560 (2330-7660)	0.4 (0.2-0.7)	93200 (47800-158000)	9.1 (4.7-15.3)
Enterococcus faecium	Resistance to one or more antibiotics	99 (63-154)	11.6 (7.4-18.0)	8790 (5610-13700)	1028.3 (655.7-1602.7)	29 (17-48)	3.4 (2.0-5.7)	2600 (1520-4310)	304.5 (178.1-503.8)	66 (36-112)	0.6 (0.3-1.1)	5870 (3170-9960)	57.1 (30.8-96.8)	20 (10-36)	0.2 (0.1-0.3)	1740 (882-3160)	16.9 (8.6-30.7)	26 (13-46)	0.1 (0.0-0.1)	2250 (1100-3980)	4.9 (2.4-8.7)	8 (4-14)	0.0 (0.0-0.0)	652 (314-1250)	1.4 (0.7-2.7)	45900 (29000-68100)	4.5 (2.8-6.6)	867000 (544000-1290000)	84.4 (52.9-125.4)	12600 (6960-20500)	1.2 (0.7-2.0)	242000 (136000-395000)	23.5 (13.2-38.4)
Other enterococci	Fluoroquinolones	29 (17-48)	3.3 (1.9-5.6)	2530 (1470-4230)	296.5 (171.6-495.1)	5 (1-11)	0.6 (0.1-1.2)	427 (63-943)	49.9 (7.4-110.3)	10 (5-18)	0.1 (0.1-0.2)	913 (467-1600)	8.9 (4.5-15.5)	2 (0-4)	0.0 (0.0-0.0)	154 (24-327)	1.5 (0.2-3.2)	4 (2-7)	0.0 (0.0-0.0)	332 (158-616)	0.7 (0.3-1.3)	1 (0-1)	0.0 (0.0-0.0)	55 (9-120)	0.1 (0.0-0.3)	4010 (2710-5570)	0.4 (0.3-0.5)	61600 (40600-88200)	6.0 (4.0-8.6)	701 (107-1370)	0.1 (0.0-0.1)	10700 (1720-20900)	1.0 (0.2-2.0)
Other enterococci	Vancomycin	16 (9-27)	1.9 (1.1-3.1)	1460 (825-2390)	170.2 (96.4-279.6)	3 (2-6)	0.4 (0.2-0.7)	297 (145-531)	34.8 (17.0-62.1)	6 (3-10)	0.1 (0.0-0.1)	524 (279-921)	5.1 (2.7-9.0)	1 (1-2)	0.0 (0.0-0.0)	107 (51-204)	1.0 (0.5-2.0)	2 (1-4)	0.0 (0.0-0.0)	198 (98-380)	0.4 (0.2-0.8)	0 (0-1)	0.0 (0.0-0.0)	43 (19-85)	0.1 (0.0-0.2)	2280 (1600-3160)	0.2 (0.2-0.3)	36400 (24500-52000)	3.5 (2.4-5.1)	502 (303-797)	0.0 (0.0-0.1)	8090 (4820-13300)	0.8 (0.5-1.3)
Other enterococci	Resistance to one or more antibiotics	33 (19-54)	3.9 (2.3-6.4)	2970 (1730-4840)	347.9 (201.8-565.7)	8 (4-15)	1.0 (0.5-1.7)	724 (354-1330)	84.7 (41.4-155.1)	12 (6-21)	0.1 (0.1-0.2)	1070 (572-1870)	10.4 (5.6-18.2)	3 (1-6)	0.0 (0.0-0.1)	261 (117-493)	2.5 (1.1-4.8)	4 (2-9)	0.0 (0.0-0.0)	404 (198-759)	0.9 (0.4-1.7)	1 (0-2)	0.0 (0.0-0.0)	98 (43-200)	0.2 (0.1-0.4)	4970 (3460-6880)	0.5 (0.3-0.7)	77200 (51800-110000)	7.5 (5.0-10.7)	1200 (646-1890)	0.1 (0.1-0.2)	18700 (10300-29400)	1.8 (1.0-2.9)
Escherichia coli	Aminoglycosides	53 (33-81)	6.2 (3.8-9.5)	4700 (2920-7250)	550.2 (341.9-848.0)	3 (2-6)	0.4 (0.2-0.7)	309 (179-497)	36.2 (21.0-58.1)	29 (18-44)	0.3 (0.2-0.4)	2760 (1770-4140)	26.8 (17.2-40.3)	2 (1-3)	0.0 (0.0-0.0)	179 (108-277)	1.7 (1.1-2.7)	13 (8-19)	0.0 (0.0-0.0)	1350 (902-1960)	3.0 (2.0-4.3)	1 (0-1)	0.0 (0.0-0.0)	86 (55-129)	0.2 (0.1-0.3)	26400 (17800-37300)	2.6 (1.7-3.6)	421000 (278000-600000)	40.9 (27.1-58.4)	1710 (1080-2510)	0.2 (0.1-0.2)	27100 (16900-40300)	2.6 (1.6-3.9)
Escherichia coli	Aminopenicillin	298 (188-453)	34.9 (21.9-53.0)	26600 (16700-40500)	3115.7 (1955.8-4742.1)	8 (4-12)	0.9 (0.5-1.4)	687 (396-1070)	80.4 (46.3-125.3)	155 (100-235)	1.5 (1.0-2.3)	14900 (9850-22100)	144.9 (95.7-214.8)	4 (2-6)	0.0 (0.0-0.1)	373 (232-566)	3.6 (2.3-5.5)	69 (45-103)	0.2 (0.1-0.2)	7330 (4920-10600)	16.0 (10.7-23.1)	2 (1-3)	0.0 (0.0-0.0)	184 (118-278)	0.4 (0.3-0.6)	129000 (89100-181000)	12.6 (8.7-17.7)	2070000 (1390000-2950000)	201.3 (135.4-286.8)	3460 (2250-5110)	0.3 (0.2-0.5)	54400 (34600-80700)	5.3 (3.4-7.9)
Escherichia coli	Beta Lactam/Beta-lactamase inhibitors	223 (140-343)	26.1 (16.3-40.1)	19900 (12500-30600)	2333.1 (1462.5-3577.3)	17 (10-27)	2.0 (1.2-3.1)	1540 (932-2400)	180.0 (109.0-281.0)	117 (75-177)	1.1 (0.7-1.7)	11200 (7420-16800)	109.3 (72.2-163.2)	9 (6-13)	0.1 (0.1-0.1)	854 (548-1260)	8.3 (5.3-12.2)	52 (34-78)	0.1 (0.1-0.2)	5500 (3700-8010)	12.0 (8.1-17.5)	4 (2-6)	0.0 (0.0-0.0)	413 (271-608)	0.9 (0.6-1.3)	95000 (65800-132000)	9.2 (6.4-12.9)	1530000 (1040000-2160000)	148.8 (101.5-210.0)	7180 (4800-10300)	0.7 (0.5-1.0)	115000 (76100-168000)	11.2 (7.4-16.3)
Escherichia coli	Carbapenem	8 (5-12)	0.9 (0.5-1.4)	671 (419-1050)	78.5 (49.1-122.3)	2 (1-4)	0.3 (0.1-0.4)	191 (101-331)	22.3 (11.8-38.7)	4 (2-6)	0.0 (0.0-0.1)	375 (242-567)	3.6 (2.4-5.5)	1 (1-2)	0.0 (0.0-0.0)	105 (58-175)	1.0 (0.6-1.7)	2 (1-3)	0.0 (0.0-0.0)	188 (124-280)	0.4 (0.3-0.6)	1 (0-1)	0.0 (0.0-0.0)	51 (30-83)	0.1 (0.1-0.2)	3250 (2200-4620)	0.3 (0.2-0.5)	52800 (34900-75900)	5.1 (3.4-7.4)	938 (520-1510)	0.1 (0.1-0.1)	15100 (8370-24300)	1.5 (0.8-2.4)
Escherichia coli	Fluoroquinolones	147 (91-223)	17.2 (10.7-26.1)	13100 (8170-19900)	1535.9 (955.4-2330.8)	17 (10-27)	2.0 (1.2-3.1)	1500 (894-2370)	175.7 (104.6-277.2)	80 (51-122)	0.8 (0.5-1.2)	7640 (4970-11500)	74.3 (48.3-111.6)	9 (6-14)	0.1 (0.1-0.1)	871 (546-1330)	8.5 (5.3-12.9)	36 (23-53)	0.1 (0.0-0.1)	3770 (2520-5450)	8.2 (5.5-11.9)	4 (3-6)	0.0 (0.0-0.0)	420 (273-618)	0.9 (0.6-1.3)	71200 (49200-99900)	6.9 (4.8-9.7)	1130000 (768000-1610000)	110.1 (74.8-156.4)	8310 (5570-12100)	0.8 (0.5-1.2)	132000 (85800-195000)	12.8 (8.4-19.0)
Escherichia coli	Third-generation cephalosporins	102 (63-159)	12.0 (7.3-18.6)	9140 (5620-14200)	1069.2 (656.9-1659.8)	15 (6-31)	1.8 (0.7-3.6)	1350 (540-2750)	158.4 (63.1-322.0)	55 (35-83)	0.5 (0.3-0.8)	5210 (3370-7860)	50.7 (32.8-76.5)	8 (3-16)	0.1 (0.0-0.2)	762 (338-1460)	7.4 (3.3-14.2)	24 (15-37)	0.1 (0.0-0.1)	2540 (1700-3770)	5.5 (3.7-8.2)	4 (1-7)	0.0 (0.0-0.0)	366 (167-668)	0.8 (0.4-1.5)	45800 (31100-65200)	4.5 (3.0-6.3)	729000 (486000-1050000)	71.0 (47.3-102.1)	6610 (2810-12300)	0.6 (0.3-1.2)	105000 (45300-196000)	10.2 (4.4-19.1)
Escherichia coli	Trimethoprim-Sulfamethoxazole	168 (106-256)	19.6 (12.4-29.9)	15000 (9480-22900)	1753.9 (1108.3-2673.8)	11 (6-17)	1.3 (0.8-2.0)	961 (572-1520)	112.4 (66.9-177.6)	86 (55-129)	0.8 (0.5-1.3)	8290 (5440-12400)	80.6 (52.8-120.3)	5 (3-9)	0.1 (0.0-0.1)	505 (305-775)	4.9 (3.0-7.5)	39 (25-58)	0.1 (0.1-0.1)	4120 (2760-6030)	9.0 (6.0-13.2)	2 (2-4)	0.0 (0.0-0.0)	238 (139-377)	0.5 (0.3-0.8)	74000 (50700-104000)	7.2 (4.9-10.1)	1180000 (792000-1670000)	114.5 (77.1-162.3)	4700 (3040-6880)	0.5 (0.3-0.7)	74000 (47200-110000)	7.2 (4.6-10.7)
Escherichia coli	Resistance to one or more antibiotics	329 (207-498)	38.4 (24.3-58.2)	29400 (18500-44600)	3434.6 (2161.8-5217.4)	73 (44-115)	8.6 (5.2-13.4)	6540 (3970-10200)	765.3 (464.8-1198.5)	172 (111-260)	1.7 (1.1-2.5)	16500 (10800-24600)	160.9 (105.5-239.0)	39 (25-60)	0.4 (0.2-0.6)	3650 (2340-5590)	35.5 (22.7-54.3)	77 (50-114)	0.2 (0.1-0.2)	8180 (5480-11800)	17.9 (12.0-25.8)	17 (11-27)	0.0 (0.0-0.1)	1760 (1170-2600)	3.8 (2.6-5.7)	146000 (101000-206000)	14.3 (9.9-20.0)	2330000 (1580000-3310000)	227.3 (153.6-322.4)	32900 (22000-47200)	3.2 (2.1-4.6)	523000 (341000-758000)	50.9 (33.2-73.8)
Group A *Streptococcus*	Macrolide	18 (11-29)	2.1 (1.3-3.4)	1580 (966-2580)	185.1 (113.0-301.6)	2 (0-6)	0.2 (0.0-0.7)	149 (0-496)	17.5 (0.0-58.0)	21 (12-33)	0.2 (0.1-0.3)	1900 (1140-2970)	18.5 (11.1-28.9)	2 (0-6)	0.0 (0.0-0.1)	179 (0-580)	1.7 (0.0-5.6)	9 (5-17)	0.0 (0.0-0.0)	1060 (613-1760)	2.3 (1.3-3.8)	1 (0-3)	0.0 (0.0-0.0)	99 (0-303)	0.2 (0.0-0.7)	3710 (1890-6900)	0.4 (0.2-0.7)	73800 (40700-133000)	7.2 (4.0-13.0)	355 (0-1300)	0.0 (0.0-0.1)	7010 (0-24400)	0.7 (0.0-2.4)
Group A *Streptococcus*	Resistance to one or more antibiotics	18 (11-29)	2.1 (1.3-3.4)	1580 (966-2580)	185.1 (113.0-301.6)	2 (0-6)	0.2 (0.0-0.7)	149 (0-496)	17.5 (0.0-58.0)	21 (12-33)	0.2 (0.1-0.3)	1900 (1140-2970)	18.5 (11.1-28.9)	2 (0-6)	0.0 (0.0-0.1)	179 (0-580)	1.7 (0.0-5.6)	9 (5-17)	0.0 (0.0-0.0)	1060 (613-1760)	2.3 (1.3-3.8)	1 (0-3)	0.0 (0.0-0.0)	99 (0-303)	0.2 (0.0-0.7)	3710 (1890-6900)	0.4 (0.2-0.7)	73800 (40700-133000)	7.2 (4.0-13.0)	355 (0-1300)	0.0 (0.0-0.1)	7010 (0-24400)	0.7 (0.0-2.4)
Group B *Streptococcus*	Fluoroquinolones	27 (15-44)	3.2 (1.8-5.2)	2410 (1360-3960)	281.4 (158.7-462.7)	5 (1-11)	0.6 (0.1-1.3)	434 (63-989)	50.7 (7.4-115.7)	13 (8-19)	0.1 (0.1-0.2)	1140 (723-1680)	11.1 (7.0-16.3)	2 (0-5)	0.0 (0.0-0.0)	201 (31-453)	2.0 (0.3-4.4)	6 (3-9)	0.0 (0.0-0.0)	521 (321-813)	1.1 (0.7-1.8)	1 (0-2)	0.0 (0.0-0.0)	89 (15-203)	0.2 (0.0-0.4)	3840 (2650-5570)	0.4 (0.3-0.5)	64100 (42500-94400)	6.2 (4.1-9.2)	668 (118-1470)	0.1 (0.0-0.1)	11000 (1950-25100)	1.1 (0.2-2.4)
Group B *Streptococcus*	Macrolide	113 (68-177)	13.2 (7.9-20.7)	10000 (6050-15700)	1170.6 (707.2-1841.2)	11 (0-33)	1.3 (0.0-3.9)	1020 (0-2940)	119.1 (0.0-343.5)	56 (36-83)	0.5 (0.4-0.8)	4990 (3210-7330)	48.5 (31.2-71.3)	6 (0-16)	0.1 (0.0-0.2)	507 (0-1430)	4.9 (0.0-13.9)	25 (15-40)	0.1 (0.0-0.1)	2250 (1360-3540)	4.9 (3.0-7.7)	3 (0-7)	0.0 (0.0-0.0)	227 (0-646)	0.5 (0.0-1.4)	13900 (9280-20100)	1.4 (0.9-2.0)	267000 (171000-398000)	26.0 (16.6-38.8)	1390 (0-3940)	0.1 (0.0-0.4)	26800 (0-74800)	2.6 (0.0-7.3)
Group B *Streptococcus*	Penicillin	2 (1-3)	0.2 (0.1-0.4)	178 (105-286)	20.9 (12.3-33.4)	0 (0-1)	0.1 (0.0-0.1)	42 (8-100)	4.9 (1.0-11.7)	1 (1-2)	0.0 (0.0-0.0)	90 (55-138)	0.9 (0.5-1.3)	0 (0-1)	0.0 (0.0-0.0)	21 (4-47)	0.2 (0.0-0.5)	0 (0-1)	0.0 (0.0-0.0)	43 (25-68)	0.1 (0.1-0.1)	0 (0-0)	0.0 (0.0-0.0)	10 (2-22)	0.0 (0.0-0.0)	299 (194-450)	0.0 (0.0-0.0)	5290 (3350-8000)	0.5 (0.3-0.8)	67 (14-146)	0.0 (0.0-0.0)	1190 (236-2620)	0.1 (0.0-0.3)
Group B *Streptococcus*	Resistance to one or more antibiotics	129 (78-203)	15.0 (9.1-23.8)	11400 (6900-18100)	1338.4 (807.4-2116.0)	17 (0-39)	2.0 (0.0-4.6)	1490 (0-3500)	174.8 (0.0-409.7)	63 (42-93)	0.6 (0.4-0.9)	5620 (3680-8250)	54.6 (35.8-80.2)	8 (0-19)	0.1 (0.0-0.2)	729 (0-1700)	7.1 (0.0-16.6)	28 (17-45)	0.1 (0.0-0.1)	2520 (1550-3960)	5.5 (3.4-8.6)	4 (0-9)	0.0 (0.0-0.0)	326 (0-772)	0.7 (0.0-1.7)	15900 (10700-22700)	1.5 (1.0-2.2)	299000 (196000-438000)	29.1 (19.1-42.6)	2120 (100-4760)	0.2 (0.0-0.5)	39000 (527-88600)	3.8 (0.1-8.6)
Haemophilus influenzae	Aminopenicillin	16 (8-31)	1.9 (0.9-3.6)	1460 (688-2730)	171.0 (80.5-319.3)	3 (0-7)	0.3 (0.0-0.8)	250 (16-580)	29.2 (1.9-67.8)	20 (14-27)	0.2 (0.1-0.3)	1760 (1280-2390)	17.1 (12.4-23.2)	3 (0-7)	0.0 (0.0-0.1)	301 (29-610)	2.9 (0.3-5.9)	10 (7-14)	0.0 (0.0-0.0)	930 (694-1250)	2.0 (1.5-2.7)	2 (0-3)	0.0 (0.0-0.0)	157 (22-303)	0.3 (0.0-0.7)	3740 (3030-4570)	0.4 (0.3-0.4)	51800 (41600-65100)	5.0 (4.1-6.3)	648 (57-1210)	0.1 (0.0-0.1)	8920 (822-17000)	0.9 (0.1-1.7)
Haemophilus influenzae	Third-generation cephalosporins	2 (1-3)	0.2 (0.1-0.4)	145 (66-278)	17.0 (7.7-32.5)	0 (0-1)	0.1 (0.0-0.1)	44 (14-99)	5.1 (1.7-11.5)	2 (1-3)	0.0 (0.0-0.0)	171 (120-238)	1.7 (1.2-2.3)	1 (0-1)	0.0 (0.0-0.0)	51 (24-89)	0.5 (0.2-0.9)	1 (1-1)	0.0 (0.0-0.0)	87 (63-120)	0.2 (0.1-0.3)	0 (0-0)	0.0 (0.0-0.0)	26 (13-45)	0.1 (0.0-0.1)	310 (236-402)	0.0 (0.0-0.0)	4490 (3380-6000)	0.4 (0.3-0.6)	94 (46-155)	0.0 (0.0-0.0)	1350 (658-2270)	0.1 (0.1-0.2)
Haemophilus influenzae	Resistance to one or more antibiotics	17 (8-31)	2.0 (0.9-3.7)	1500 (708-2810)	175.8 (82.8-328.1)	3 (1-7)	0.4 (0.1-0.8)	293 (54-645)	34.3 (6.3-75.4)	20 (15-27)	0.2 (0.1-0.3)	1810 (1330-2450)	17.6 (12.9-23.8)	4 (1-8)	0.0 (0.0-0.1)	352 (82-666)	3.4 (0.8-6.5)	10 (8-14)	0.0 (0.0-0.0)	955 (717-1270)	2.1 (1.6-2.8)	2 (0-4)	0.0 (0.0-0.0)	183 (49-342)	0.4 (0.1-0.7)	3830 (3120-4680)	0.4 (0.3-0.5)	53100 (42800-66800)	5.2 (4.2-6.5)	742 (155-1310)	0.1 (0.0-0.1)	10300 (2320-18500)	1.0 (0.2-1.8)
Klebsiella pneumoniae	Aminoglycosides	97 (61-150)	11.3 (7.1-17.6)	8600 (5400-13300)	1005.6 (631.4-1560.7)	8 (4-14)	0.9 (0.5-1.6)	703 (394-1200)	82.2 (46.1-140.5)	54 (36-79)	0.5 (0.4-0.8)	4770 (3220-7040)	46.4 (31.3-68.4)	4 (3-7)	0.0 (0.0-0.1)	393 (233-614)	3.8 (2.3-6.0)	21 (14-32)	0.0 (0.0-0.1)	1850 (1200-2790)	4.0 (2.6-6.1)	2 (1-3)	0.0 (0.0-0.0)	154 (90-255)	0.3 (0.2-0.6)	16500 (11600-22800)	1.6 (1.1-2.2)	268000 (184000-376000)	26.1 (18.0-36.6)	1380 (838-2160)	0.1 (0.1-0.2)	22500 (13400-35600)	2.2 (1.3-3.5)
Klebsiella pneumoniae	Beta Lactam/Beta-lactamase inhibitors	202 (126-306)	23.7 (14.7-35.8)	18000 (11200-27200)	2105.6 (1310.0-3181.2)	7 (4-12)	0.8 (0.4-1.4)	616 (330-1030)	72.0 (38.6-120.9)	116 (78-165)	1.1 (0.8-1.6)	10300 (6910-14600)	100.0 (67.2-142.1)	4 (2-7)	0.0 (0.0-0.1)	362 (200-577)	3.5 (1.9-5.6)	47 (31-71)	0.1 (0.1-0.2)	4140 (2710-6180)	9.0 (5.9-13.5)	2 (1-3)	0.0 (0.0-0.0)	161 (87-266)	0.4 (0.2-0.6)	38900 (27500-53600)	3.8 (2.7-5.2)	632000 (438000-889000)	61.6 (42.7-86.5)	1720 (1000-2680)	0.2 (0.1-0.3)	27800 (15900-44300)	2.7 (1.5-4.3)
Klebsiella pneumoniae	Carbapenem	50 (29-79)	5.8 (3.4-9.3)	4420 (2580-7050)	517.5 (301.9-824.1)	12 (7-19)	1.4 (0.8-2.3)	1050 (583-1730)	123.4 (68.2-202.3)	28 (18-43)	0.3 (0.2-0.4)	2460 (1580-3800)	23.9 (15.4-36.9)	7 (4-11)	0.1 (0.0-0.1)	591 (353-944)	5.7 (3.4-9.2)	10 (6-16)	0.0 (0.0-0.0)	879 (554-1390)	1.9 (1.2-3.0)	3 (1-4)	0.0 (0.0-0.0)	217 (125-355)	0.5 (0.3-0.8)	7140 (4770-10700)	0.7 (0.5-1.0)	117000 (76600-179000)	11.4 (7.5-17.4)	1850 (1120-2870)	0.2 (0.1-0.3)	30200 (18200-47300)	2.9 (1.8-4.6)
Klebsiella pneumoniae	Fluoroquinolones	146 (92-223)	17.1 (10.8-26.0)	13000 (8190-19800)	1518.7 (957.4-2315.5)	11 (6-18)	1.3 (0.7-2.1)	970 (528-1600)	113.4 (61.7-186.7)	82 (56-117)	0.8 (0.5-1.1)	7290 (4940-10400)	70.9 (48.0-100.7)	6 (4-10)	0.1 (0.0-0.1)	547 (315-865)	5.3 (3.1-8.4)	32 (21-49)	0.1 (0.0-0.1)	2810 (1840-4220)	6.1 (4.0-9.2)	2 (1-4)	0.0 (0.0-0.0)	214 (120-353)	0.5 (0.3-0.8)	24700 (17800-34000)	2.4 (1.7-3.3)	405000 (282000-572000)	39.5 (27.4-55.7)	1880 (1120-2900)	0.2 (0.1-0.3)	31000 (18400-48400)	3.0 (1.8-4.7)
Klebsiella pneumoniae	Third-generation cephalosporins	176 (110-267)	20.6 (12.8-31.2)	15600 (9750-23700)	1827.3 (1140.7-2774.7)	21 (8-43)	2.5 (0.9-5.1)	1900 (676-3870)	221.7 (79.1-452.1)	100 (67-143)	1.0 (0.7-1.4)	8840 (5970-12700)	86.0 (58.1-123.2)	12 (4-24)	0.1 (0.0-0.2)	1090 (392-2130)	10.6 (3.8-20.7)	39 (25-59)	0.1 (0.1-0.1)	3400 (2230-5160)	7.4 (4.9-11.3)	5 (2-10)	0.0 (0.0-0.0)	425 (154-870)	0.9 (0.3-1.9)	29600 (21100-41700)	2.9 (2.1-4.1)	484000 (334000-697000)	47.1 (32.5-67.8)	3760 (1410-7280)	0.4 (0.1-0.7)	61600 (22500-120000)	6.0 (2.2-11.6)
Klebsiella pneumoniae	Trimethoprim-Sulfamethoxazole	155 (97-236)	18.1 (11.4-27.6)	13800 (8640-21000)	1611.6 (1010.9-2454.6)	9 (4-15)	1.0 (0.5-1.8)	764 (346-1370)	89.3 (40.4-160.4)	88 (60-127)	0.9 (0.6-1.2)	7840 (5300-11200)	76.2 (51.5-109.3)	5 (2-9)	0.0 (0.0-0.1)	439 (210-793)	4.3 (2.0-7.7)	36 (24-54)	0.1 (0.1-0.1)	3170 (2090-4690)	6.9 (4.6-10.3)	2 (1-4)	0.0 (0.0-0.0)	181 (86-335)	0.4 (0.2-0.7)	28900 (20600-40000)	2.8 (2.0-3.9)	466000 (322000-656000)	45.3 (31.4-63.8)	1700 (830-2940)	0.2 (0.1-0.3)	27300 (13400-47300)	2.7 (1.3-4.6)
Klebsiella pneumoniae	Resistance to one or more antibiotics	256 (160-386)	30.0 (18.8-45.1)	22800 (14300-34300)	2663.6 (1668.2-4012.7)	68 (40-107)	7.9 (4.7-12.6)	6000 (3560-9540)	702.0 (416.4-1116.2)	147 (100-209)	1.4 (1.0-2.0)	13000 (8880-18500)	126.8 (86.3-180.1)	39 (24-59)	0.4 (0.2-0.6)	3420 (2170-5220)	33.2 (21.1-50.8)	61 (39-91)	0.1 (0.1-0.2)	5290 (3450-7910)	11.6 (7.5-17.3)	15 (9-25)	0.0 (0.0-0.1)	1350 (833-2190)	3.0 (1.8-4.8)	49600 (35400-68600)	4.8 (3.4-6.7)	805000 (561000-1140000)	78.4 (54.6-111.1)	12300 (7980-18100)	1.2 (0.8-1.8)	200000 (129000-302000)	19.5 (12.6-29.4)
Morganella spp.	Fluoroquinolones	0 (0-0)	0.0 (0.0-0.0)	1 (1-3)	0.2 (0.1-0.4)	0 (0-0)	0.0 (0.0-0.0)	0 (0-1)	0.0 (0.0-0.1)	0 (0-0)	0.0 (0.0-0.0)	4 (2-8)	0.0 (0.0-0.1)	0 (0-0)	0.0 (0.0-0.0)	1 (0-2)	0.0 (0.0-0.0)	0 (0-0)	0.0 (0.0-0.0)	3 (2-7)	0.0 (0.0-0.0)	0 (0-0)	0.0 (0.0-0.0)	1 (0-1)	0.0 (0.0-0.0)	167 (121-231)	0.0 (0.0-0.0)	2160 (1500-3110)	0.2 (0.1-0.3)	28 (11-51)	0.0 (0.0-0.0)	354 (144-662)	0.0 (0.0-0.1)
Morganella spp.	Fourth-generation cephalosporins	0 (0-0)	0.0 (0.0-0.0)	0 (0-1)	0.0 (0.0-0.1)	0 (0-0)	0.0 (0.0-0.0)	0 (0-0)	0.0 (0.0-0.0)	0 (0-0)	0.0 (0.0-0.0)	1 (0-1)	0.0 (0.0-0.0)	0 (0-0)	0.0 (0.0-0.0)	0 (0-0)	0.0 (0.0-0.0)	0 (0-0)	0.0 (0.0-0.0)	1 (0-1)	0.0 (0.0-0.0)	0 (0-0)	0.0 (0.0-0.0)	0 (0-0)	0.0 (0.0-0.0)	31 (22-45)	0.0 (0.0-0.0)	397 (273-593)	0.0 (0.0-0.1)	7 (4-13)	0.0 (0.0-0.0)	93 (47-172)	0.0 (0.0-0.0)
Morganella spp.	Third-generation cephalosporins	0 (0-0)	0.0 (0.0-0.0)	2 (1-4)	0.2 (0.1-0.5)	0 (0-0)	0.0 (0.0-0.0)	0 (0-1)	0.0 (0.0-0.1)	0 (0-0)	0.0 (0.0-0.0)	5 (2-11)	0.0 (0.0-0.1)	0 (0-0)	0.0 (0.0-0.0)	1 (0-3)	0.0 (0.0-0.0)	0 (0-0)	0.0 (0.0-0.0)	4 (2-9)	0.0 (0.0-0.0)	0 (0-0)	0.0 (0.0-0.0)	1 (0-2)	0.0 (0.0-0.0)	201 (145-283)	0.0 (0.0-0.0)	2630 (1830-3830)	0.3 (0.2-0.4)	42 (17-80)	0.0 (0.0-0.0)	547 (229-1090)	0.1 (0.0-0.1)
Morganella spp.	Resistance to one or more antibiotics	0 (0-0)	0.0 (0.0-0.0)	3 (1-6)	0.3 (0.1-0.7)	0 (0-0)	0.0 (0.0-0.0)	1 (0-2)	0.1 (0.0-0.2)	0 (0-0)	0.0 (0.0-0.0)	7 (3-15)	0.1 (0.0-0.2)	0 (0-0)	0.0 (0.0-0.0)	2 (1-4)	0.0 (0.0-0.0)	0 (0-0)	0.0 (0.0-0.0)	6 (3-13)	0.0 (0.0-0.0)	0 (0-0)	0.0 (0.0-0.0)	2 (1-4)	0.0 (0.0-0.0)	295 (219-411)	0.0 (0.0-0.0)	3850 (2740-5490)	0.4 (0.3-0.5)	77 (42-128)	0.0 (0.0-0.0)	995 (530-1700)	0.1 (0.1-0.2)
Mycobacterium tuberculosis	Extensive drug resistance in TB	--	--	0 (0-0)	0.0 (0.0-0.0)	--	--	0 (0-0)	0.0 (0.0-0.0)	0 (0-0)	0.0 (0.0-0.0)	6 (2-15)	0.1 (0.0-0.1)	0 (0-0)	0.0 (0.0-0.0)	3 (1-9)	0.0 (0.0-0.1)	0 (0-0)	0.0 (0.0-0.0)	8 (3-20)	0.0 (0.0-0.0)	0 (0-0)	0.0 (0.0-0.0)	4 (2-11)	0.0 (0.0-0.0)	101 (40-217)	0.0 (0.0-0.0)	1750 (699-3710)	0.2 (0.1-0.4)	62 (24-134)	0.0 (0.0-0.0)	1020 (406-2130)	0.1 (0.0-0.2)
Mycobacterium tuberculosis	Isoniazid mono-resistance	--	--	0 (0-0)	0.0 (0.0-0.0)	--	--	0 (0-0)	0.0 (0.0-0.0)	0 (0-1)	0.0 (0.0-0.0)	36 (25-52)	0.4 (0.2-0.5)	0 (0-0)	0.0 (0.0-0.0)	6 (0-18)	0.1 (0.0-0.2)	1 (0-1)	0.0 (0.0-0.0)	51 (37-70)	0.1 (0.1-0.2)	0 (0-0)	0.0 (0.0-0.0)	7 (0-24)	0.0 (0.0-0.1)	822 (536-1170)	0.1 (0.1-0.1)	14800 (10400-20000)	1.4 (1.0-1.9)	130 (0-440)	0.0 (0.0-0.0)	2130 (0-7030)	0.2 (0.0-0.7)
Mycobacterium tuberculosis	Multi-drug resistance excluding extensive drug resistance in TB	--	--	0 (0-0)	0.0 (0.0-0.0)	--	--	0 (0-0)	0.0 (0.0-0.0)	0 (0-1)	0.0 (0.0-0.0)	22 (7-56)	0.2 (0.1-0.5)	0 (0-0)	0.0 (0.0-0.0)	12 (1-39)	0.1 (0.0-0.4)	0 (0-1)	0.0 (0.0-0.0)	31 (11-77)	0.1 (0.0-0.2)	0 (0-1)	0.0 (0.0-0.0)	16 (2-52)	0.0 (0.0-0.1)	384 (152-819)	0.0 (0.0-0.1)	6960 (2800-14600)	0.7 (0.3-1.4)	228 (21-604)	0.0 (0.0-0.1)	3730 (362-9990)	0.4 (0.0-1.0)
Mycobacterium tuberculosis	Rifampicin mono-resistance	--	--	0 (0-0)	0.0 (0.0-0.0)	--	--	0 (0-0)	0.0 (0.0-0.0)	0 (0-0)	0.0 (0.0-0.0)	5 (4-8)	0.1 (0.0-0.1)	0 (0-0)	0.0 (0.0-0.0)	2 (0-3)	0.0 (0.0-0.0)	0 (0-0)	0.0 (0.0-0.0)	7 (5-9)	0.0 (0.0-0.0)	0 (0-0)	0.0 (0.0-0.0)	2 (0-4)	0.0 (0.0-0.0)	89 (64-119)	0.0 (0.0-0.0)	1670 (1250-2180)	0.2 (0.1-0.2)	25 (4-49)	0.0 (0.0-0.0)	441 (88-861)	0.0 (0.0-0.1)
Mycobacterium tuberculosis	Resistance to one or more antibiotics	--	--	0 (0-0)	0.0 (0.0-0.0)	--	--	0 (0-0)	0.0 (0.0-0.0)	1 (0-1)	0.0 (0.0-0.0)	69 (46-111)	0.7 (0.4-1.1)	0 (0-1)	0.0 (0.0-0.0)	23 (5-55)	0.2 (0.1-0.5)	1 (1-2)	0.0 (0.0-0.0)	96 (67-153)	0.2 (0.1-0.3)	0 (0-1)	0.0 (0.0-0.0)	30 (7-73)	0.1 (0.0-0.2)	1400 (969-1960)	0.1 (0.1-0.2)	25200 (18800-34700)	2.5 (1.8-3.4)	445 (112-935)	0.0 (0.0-0.1)	7310 (1930-15700)	0.7 (0.2-1.5)
Neisseria gonorrhoeae	Fluoroquinolones	--	--	--	--	--	--	--	--	--	--	--	--	--	--	--	--	--	--	--	--	--	--	--	--	--	--	1550 (933-2460)	0.2 (0.1-0.2)	--	--	145 (39-290)	0.0 (0.0-0.0)
Neisseria gonorrhoeae	Third-generation cephalosporins	--	--	--	--	--	--	--	--	--	--	--	--	--	--	--	--	--	--	--	--	--	--	--	--	--	--	209 (123-341)	0.0 (0.0-0.0)	--	--	41 (15-82)	0.0 (0.0-0.0)
Neisseria gonorrhoeae	Resistance to one or more antibiotics	--	--	--	--	--	--	--	--	--	--	--	--	--	--	--	--	--	--	--	--	--	--	--	--	--	--	1610 (983-2550)	0.2 (0.1-0.2)	--	--	186 (74-341)	0.0 (0.0-0.0)
Proteus spp.	Aminoglycosides	2 (1-3)	0.2 (0.1-0.4)	178 (109-287)	20.8 (12.8-33.6)	0 (0-0)	0.0 (0.0-0.0)	9 (1-20)	1.0 (0.1-2.3)	2 (1-3)	0.0 (0.0-0.0)	137 (79-224)	1.3 (0.8-2.2)	0 (0-0)	0.0 (0.0-0.0)	7 (1-15)	0.1 (0.0-0.1)	1 (0-1)	0.0 (0.0-0.0)	59 (34-94)	0.1 (0.1-0.2)	0 (0-0)	0.0 (0.0-0.0)	3 (1-6)	0.0 (0.0-0.0)	1420 (996-1950)	0.1 (0.1-0.2)	23000 (15700-32000)	2.2 (1.5-3.1)	71 (8-155)	0.0 (0.0-0.0)	1130 (137-2470)	0.1 (0.0-0.2)
Proteus spp.	Aminopenicillin	13 (8-20)	1.5 (0.9-2.4)	1160 (704-1820)	135.2 (82.4-212.3)	0 (0-1)	0.0 (0.0-0.1)	26 (0-84)	3.1 (0.0-9.8)	9 (5-16)	0.1 (0.1-0.2)	847 (491-1400)	8.2 (4.8-13.6)	0 (0-1)	0.0 (0.0-0.0)	19 (0-61)	0.2 (0.0-0.6)	4 (2-7)	0.0 (0.0-0.0)	396 (232-631)	0.9 (0.5-1.4)	0 (0-0)	0.0 (0.0-0.0)	9 (0-28)	0.0 (0.0-0.1)	10700 (7510-14700)	1.0 (0.7-1.4)	163000 (112000-227000)	15.8 (10.9-22.1)	245 (0-746)	0.0 (0.0-0.1)	3700 (0-11300)	0.4 (0.0-1.1)
Proteus spp.	Fluoroquinolones	7 (4-11)	0.8 (0.5-1.3)	623 (381-988)	72.9 (44.6-115.5)	1 (0-1)	0.1 (0.0-0.1)	50 (19-98)	5.8 (2.3-11.4)	5 (3-9)	0.1 (0.0-0.1)	484 (278-798)	4.7 (2.7-7.8)	0 (0-1)	0.0 (0.0-0.0)	38 (15-81)	0.4 (0.1-0.8)	2 (1-4)	0.0 (0.0-0.0)	207 (119-329)	0.5 (0.3-0.7)	0 (0-0)	0.0 (0.0-0.0)	15 (6-32)	0.0 (0.0-0.1)	4820 (3360-6660)	0.5 (0.3-0.6)	77600 (52900-109000)	7.6 (5.1-10.6)	382 (164-714)	0.0 (0.0-0.1)	6100 (2600-11500)	0.6 (0.3-1.1)
Proteus spp.	Third-generation cephalosporins	4 (2-6)	0.4 (0.2-0.7)	315 (187-514)	36.8 (21.8-60.1)	1 (0-2)	0.1 (0.0-0.2)	68 (21-145)	7.9 (2.5-17.0)	3 (2-4)	0.0 (0.0-0.0)	245 (139-403)	2.4 (1.3-3.9)	1 (0-1)	0.0 (0.0-0.0)	52 (15-111)	0.5 (0.2-1.1)	1 (1-2)	0.0 (0.0-0.0)	112 (64-184)	0.2 (0.1-0.4)	0 (0-1)	0.0 (0.0-0.0)	24 (8-49)	0.1 (0.0-0.1)	2950 (2010-4260)	0.3 (0.2-0.4)	45200 (30000-65900)	4.4 (2.9-6.4)	642 (213-1230)	0.1 (0.0-0.1)	9770 (3250-18800)	1.0 (0.3-1.8)
Proteus spp.	Trimethoprim-Sulfamethoxazole	8 (5-12)	0.9 (0.6-1.5)	705 (427-1110)	82.4 (50.0-130.2)	0 (0-1)	0.0 (0.0-0.1)	27 (0-63)	3.2 (0.0-7.4)	6 (3-10)	0.1 (0.0-0.1)	535 (308-883)	5.2 (3.0-8.6)	0 (0-1)	0.0 (0.0-0.0)	20 (0-48)	0.2 (0.0-0.5)	3 (1-4)	0.0 (0.0-0.0)	247 (143-397)	0.5 (0.3-0.9)	0 (0-0)	0.0 (0.0-0.0)	10 (0-23)	0.0 (0.0-0.0)	6850 (4800-9440)	0.7 (0.5-0.9)	104000 (72000-146000)	10.2 (7.0-14.2)	272 (0-607)	0.0 (0.0-0.1)	4110 (5-9260)	0.4 (0.0-0.9)
Proteus spp.	Resistance to one or more antibiotics	16 (10-25)	1.8 (1.1-2.9)	1400 (857-2200)	163.6 (100.2-256.9)	2 (1-4)	0.2 (0.1-0.4)	179 (91-328)	21.0 (10.6-38.4)	12 (7-19)	0.1 (0.1-0.2)	1050 (608-1720)	10.2 (5.9-16.7)	2 (1-3)	0.0 (0.0-0.0)	136 (64-249)	1.3 (0.6-2.4)	5 (3-8)	0.0 (0.0-0.0)	482 (283-769)	1.1 (0.6-1.7)	1 (0-1)	0.0 (0.0-0.0)	61 (29-111)	0.1 (0.1-0.2)	12800 (9030-17600)	1.2 (0.9-1.7)	196000 (135000-273000)	19.1 (13.2-26.6)	1610 (896-2590)	0.2 (0.1-0.3)	24800 (13700-40200)	2.4 (1.3-3.9)
Pseudomonas aeruginosa	Aminoglycosides	49 (29-76)	5.7 (3.4-8.9)	4340 (2570-6790)	508.1 (300.7-794.0)	1 (0-3)	0.1 (0.0-0.4)	109 (0-281)	12.8 (0.0-32.9)	21 (14-32)	0.2 (0.1-0.3)	1890 (1250-2800)	18.4 (12.1-27.2)	1 (0-1)	0.0 (0.0-0.0)	48 (0-121)	0.5 (0.0-1.2)	9 (5-13)	0.0 (0.0-0.0)	765 (493-1160)	1.7 (1.1-2.5)	0 (0-1)	0.0 (0.0-0.0)	21 (1-51)	0.0 (0.0-0.1)	9710 (7010-13300)	0.9 (0.7-1.3)	160000 (111000-224000)	15.6 (10.8-21.8)	252 (0-615)	0.0 (0.0-0.1)	4190 (51-10200)	0.4 (0.0-1.0)
Pseudomonas aeruginosa	Anti-pseudomonal penicillin/Beta-Lactamase inhibitors	91 (55-141)	10.6 (6.4-16.5)	8070 (4890-12500)	944.2 (571.9-1464.9)	7 (4-13)	0.8 (0.5-1.5)	641 (353-1120)	74.9 (41.3-130.7)	41 (27-61)	0.4 (0.3-0.6)	3650 (2370-5390)	35.4 (23.1-52.4)	3 (2-6)	0.0 (0.0-0.1)	300 (176-501)	2.9 (1.7-4.9)	17 (11-27)	0.0 (0.0-0.1)	1540 (983-2340)	3.4 (2.1-5.1)	2 (1-3)	0.0 (0.0-0.0)	132 (76-220)	0.3 (0.2-0.5)	22100 (16100-30200)	2.2 (1.6-2.9)	357000 (248000-500000)	34.8 (24.2-48.7)	2120 (1370-3240)	0.2 (0.1-0.3)	33500 (21000-51800)	3.3 (2.0-5.0)
Pseudomonas aeruginosa	Carbapenem	111 (67-174)	13.0 (7.8-20.4)	9850 (5920-15500)	1152.3 (692.9-1813.9)	19 (11-32)	2.3 (1.3-3.7)	1730 (985-2850)	202.9 (115.3-333.4)	49 (32-72)	0.5 (0.3-0.7)	4380 (2870-6410)	42.5 (27.9-62.3)	9 (5-14)	0.1 (0.1-0.1)	773 (476-1200)	7.5 (4.6-11.7)	21 (13-32)	0.0 (0.0-0.1)	1820 (1150-2780)	4.0 (2.5-6.1)	4 (2-6)	0.0 (0.0-0.0)	322 (194-517)	0.7 (0.4-1.1)	24300 (17500-33200)	2.4 (1.7-3.2)	402000 (277000-561000)	39.1 (27.0-54.7)	4370 (2870-6450)	0.4 (0.3-0.6)	72100 (45700-108000)	7.0 (4.5-10.5)
Pseudomonas aeruginosa	Fluoroquinolones	114 (70-180)	13.4 (8.2-21.0)	10200 (6250-16000)	1190.1 (730.8-1872.1)	13 (7-21)	1.5 (0.8-2.5)	1130 (647-1870)	132.6 (75.6-218.7)	51 (33-74)	0.5 (0.3-0.7)	4510 (2960-6560)	43.9 (28.7-63.8)	6 (3-9)	0.1 (0.0-0.1)	506 (308-768)	4.9 (3.0-7.5)	21 (13-32)	0.0 (0.0-0.1)	1870 (1190-2820)	4.1 (2.6-6.2)	2 (1-4)	0.0 (0.0-0.0)	215 (131-336)	0.5 (0.3-0.7)	24500 (17500-33500)	2.4 (1.7-3.3)	411000 (284000-578000)	40.0 (27.7-56.2)	2750 (1790-3980)	0.3 (0.2-0.4)	46500 (29300-68500)	4.5 (2.9-6.7)
Pseudomonas aeruginosa	Fourth-generation cephalosporins	85 (51-132)	10.0 (6.0-15.4)	7580 (4570-11700)	885.9 (534.3-1371.0)	2 (1-3)	0.2 (0.1-0.4)	172 (89-298)	20.1 (10.5-34.9)	38 (25-56)	0.4 (0.2-0.5)	3380 (2200-4960)	32.9 (21.4-48.2)	1 (0-1)	0.0 (0.0-0.0)	77 (43-127)	0.8 (0.4-1.2)	16 (10-24)	0.0 (0.0-0.1)	1400 (896-2110)	3.1 (2.0-4.6)	0 (0-1)	0.0 (0.0-0.0)	32 (18-54)	0.1 (0.0-0.1)	19100 (13800-26100)	1.9 (1.3-2.5)	315000 (216000-441000)	30.6 (21.0-42.9)	428 (260-668)	0.0 (0.0-0.1)	7210 (4240-11300)	0.7 (0.4-1.1)
Pseudomonas aeruginosa	Third-generation cephalosporins	100 (59-160)	11.7 (6.9-18.7)	8910 (5250-14300)	1041.9 (613.8-1667.1)	4 (2-9)	0.5 (0.2-1.0)	396 (148-779)	46.3 (17.3-91.2)	45 (28-67)	0.4 (0.3-0.7)	3990 (2530-5970)	38.8 (24.6-58.1)	2 (1-4)	0.0 (0.0-0.0)	178 (71-338)	1.7 (0.7-3.3)	19 (12-29)	0.0 (0.0-0.1)	1660 (1020-2550)	3.6 (2.2-5.6)	1 (0-2)	0.0 (0.0-0.0)	74 (30-142)	0.2 (0.1-0.3)	22300 (15400-31100)	2.2 (1.5-3.0)	373000 (248000-532000)	36.3 (24.2-51.8)	934 (387-1700)	0.1 (0.0-0.2)	16400 (6780-30000)	1.6 (0.7-2.9)
Pseudomonas aeruginosa	Resistance to one or more antibiotics	192 (116-299)	22.4 (13.6-34.9)	17100 (10300-26600)	1996.6 (1210.4-3110.1)	47 (27-79)	5.5 (3.1-9.2)	4190 (2390-7010)	489.7 (279.2-819.6)	86 (57-127)	0.8 (0.6-1.2)	7660 (5030-11200)	74.5 (48.9-109.2)	21 (13-34)	0.2 (0.1-0.3)	1880 (1160-2990)	18.3 (11.3-29.1)	36 (23-55)	0.1 (0.0-0.1)	3230 (2040-4850)	7.0 (4.5-10.6)	9 (5-15)	0.0 (0.0-0.0)	796 (474-1290)	1.7 (1.0-2.8)	44300 (31800-60100)	4.3 (3.1-5.9)	733000 (503000-1020000)	71.3 (48.9-99.4)	10900 (7140-16000)	1.1 (0.7-1.6)	180000 (115000-271000)	17.5 (11.2-26.3)
Salmonella Paratyphi	Fluoroquinolones	--	--	0 (0-0)	0.0 (0.0-0.0)	--	--	0 (0-0)	0.0 (0.0-0.0)	0 (0-0)	0.0 (0.0-0.0)	5 (3-6)	0.0 (0.0-0.1)	0 (0-0)	0.0 (0.0-0.0)	1 (0-2)	0.0 (0.0-0.0)	0 (0-0)	0.0 (0.0-0.0)	8 (5-11)	0.0 (0.0-0.0)	0 (0-0)	0.0 (0.0-0.0)	2 (0-3)	0.0 (0.0-0.0)	4 (2-6)	0.0 (0.0-0.0)	82 (41-99)	0.0 (0.0-0.0)	1 (0-2)	0.0 (0.0-0.0)	16 (3-29)	0.0 (0.0-0.0)
Salmonella Paratyphi	Multi-drug resistance in Salmonella Typhi and Paratyphi	--	--	0 (0-0)	0.0 (0.0-0.0)	--	--	0 (0-0)	0.0 (0.0-0.0)	0 (0-0)	0.0 (0.0-0.0)	0 (0-0)	0.0 (0.0-0.0)	0 (0-0)	0.0 (0.0-0.0)	0 (0-0)	0.0 (0.0-0.0)	0 (0-0)	0.0 (0.0-0.0)	0 (0-0)	0.0 (0.0-0.0)	0 (0-0)	0.0 (0.0-0.0)	0 (0-0)	0.0 (0.0-0.0)	0 (0-0)	0.0 (0.0-0.0)	1 (0-1)	0.0 (0.0-0.0)	0 (0-0)	0.0 (0.0-0.0)	0 (0-0)	0.0 (0.0-0.0)
Salmonella Paratyphi	Resistance to one or more antibiotics	--	--	0 (0-0)	0.0 (0.0-0.0)	--	--	0 (0-0)	0.0 (0.0-0.0)	0 (0-0)	0.0 (0.0-0.0)	5 (3-6)	0.0 (0.0-0.1)	0 (0-0)	0.0 (0.0-0.0)	1 (0-2)	0.0 (0.0-0.0)	0 (0-0)	0.0 (0.0-0.0)	8 (5-11)	0.0 (0.0-0.0)	0 (0-0)	0.0 (0.0-0.0)	2 (0-3)	0.0 (0.0-0.0)	4 (2-6)	0.0 (0.0-0.0)	82 (41-99)	0.0 (0.0-0.0)	1 (0-2)	0.0 (0.0-0.0)	16 (3-29)	0.0 (0.0-0.0)
Salmonella Typhi	Fluoroquinolones	6 (2-13)	0.7 (0.2-1.5)	519 (180-1150)	60.8 (21.1-134.8)	1 (0-3)	0.1 (0.0-0.4)	107 (12-292)	12.5 (1.4-34.2)	3 (1-7)	0.0 (0.0-0.1)	272 (94-585)	2.6 (0.9-5.7)	1 (0-2)	0.0 (0.0-0.0)	55 (7-151)	0.5 (0.1-1.5)	1 (0-2)	0.0 (0.0-0.0)	93 (31-208)	0.2 (0.1-0.5)	0 (0-1)	0.0 (0.0-0.0)	19 (3-51)	0.0 (0.0-0.1)	165 (66-331)	0.0 (0.0-0.0)	4460 (1770-9120)	0.4 (0.2-0.9)	34 (5-82)	0.0 (0.0-0.0)	902 (123-2280)	0.1 (0.0-0.2)
Salmonella Typhi	Multi-drug resistance in Salmonella Typhi and Paratyphi	1 (0-2)	0.1 (0.0-0.3)	90 (36-191)	10.5 (4.3-22.3)	0 (0-0)	0.0 (0.0-0.0)	11 (0-34)	1.3 (0.0-4.0)	1 (0-1)	0.0 (0.0-0.0)	49 (19-105)	0.5 (0.2-1.0)	0 (0-0)	0.0 (0.0-0.0)	6 (0-19)	0.1 (0.0-0.2)	0 (0-1)	0.0 (0.0-0.0)	19 (7-45)	0.0 (0.0-0.1)	0 (0-0)	0.0 (0.0-0.0)	2 (0-8)	0.0 (0.0-0.0)	41 (18-81)	0.0 (0.0-0.0)	1050 (455-2080)	0.1 (0.0-0.2)	5 (0-16)	0.0 (0.0-0.0)	128 (0-403)	0.0 (0.0-0.0)
Salmonella Typhi	Resistance to one or more antibiotics	7 (3-14)	0.8 (0.3-1.7)	597 (231-1280)	69.8 (27.1-149.5)	1 (0-3)	0.2 (0.0-0.4)	118 (20-307)	13.8 (2.3-36.0)	4 (1-7)	0.0 (0.0-0.1)	314 (117-656)	3.1 (1.1-6.4)	1 (0-2)	0.0 (0.0-0.0)	62 (11-159)	0.6 (0.1-1.5)	1 (0-3)	0.0 (0.0-0.0)	110 (39-233)	0.2 (0.1-0.5)	0 (0-1)	0.0 (0.0-0.0)	21 (4-54)	0.0 (0.0-0.1)	200 (85-400)	0.0 (0.0-0.0)	5340 (2240-10700)	0.5 (0.2-1.0)	39 (8-89)	0.0 (0.0-0.0)	1030 (221-2410)	0.1 (0.0-0.2)
Non-typhoidal *Salmonella*	Fluoroquinolones	2 (1-3)	0.2 (0.1-0.3)	168 (91-280)	19.6 (10.6-32.7)	0 (0-1)	0.0 (0.0-0.1)	30 (6-68)	3.5 (0.7-8.0)	1 (1-2)	0.0 (0.0-0.0)	527 (151-1200)	5.1 (1.5-11.7)	0 (0-1)	0.0 (0.0-0.0)	48 (2-126)	0.5 (0.0-1.2)	1 (0-1)	0.0 (0.0-0.0)	625 (28-2130)	1.4 (0.1-4.6)	0 (0-0)	0.0 (0.0-0.0)	46 (0-211)	0.1 (0.0-0.5)	172 (83-320)	0.0 (0.0-0.0)	6550 (1630-16500)	0.6 (0.2-1.6)	36 (5-86)	0.0 (0.0-0.0)	858 (122-2320)	0.1 (0.0-0.2)
Non-typhoidal *Salmonella*	Resistance to one or more antibiotics	2 (1-3)	0.2 (0.1-0.3)	168 (91-280)	19.6 (10.6-32.7)	0 (0-1)	0.0 (0.0-0.1)	30 (6-68)	3.5 (0.7-8.0)	1 (1-2)	0.0 (0.0-0.0)	527 (151-1200)	5.1 (1.5-11.7)	0 (0-1)	0.0 (0.0-0.0)	48 (2-126)	0.5 (0.0-1.2)	1 (0-1)	0.0 (0.0-0.0)	625 (28-2130)	1.4 (0.1-4.6)	0 (0-0)	0.0 (0.0-0.0)	46 (0-211)	0.1 (0.0-0.5)	172 (83-320)	0.0 (0.0-0.0)	6550 (1630-16500)	0.6 (0.2-1.6)	36 (5-86)	0.0 (0.0-0.0)	858 (122-2320)	0.1 (0.0-0.2)
Serratia spp.	Aminoglycosides	8 (4-12)	0.9 (0.5-1.4)	671 (398-1090)	78.4 (46.6-128.0)	0 (0-1)	0.1 (0.0-0.1)	39 (0-108)	4.5 (0.0-12.6)	3 (1-4)	0.0 (0.0-0.0)	225 (126-366)	2.2 (1.2-3.6)	0 (0-0)	0.0 (0.0-0.0)	13 (0-37)	0.1 (0.0-0.4)	1 (0-1)	0.0 (0.0-0.0)	68 (34-120)	0.1 (0.1-0.3)	0 (0-0)	0.0 (0.0-0.0)	4 (0-12)	0.0 (0.0-0.0)	458 (273-701)	0.0 (0.0-0.1)	9600 (5700-14800)	0.9 (0.6-1.4)	27 (0-73)	0.0 (0.0-0.0)	565 (0-1520)	0.1 (0.0-0.1)
Serratia spp.	Anti-pseudomonal penicillin/Beta-Lactamase inhibitors	11 (7-19)	1.3 (0.8-2.2)	1020 (604-1700)	119.4 (70.6-198.2)	2 (1-4)	0.3 (0.1-0.5)	205 (96-387)	23.9 (11.3-45.3)	4 (2-7)	0.0 (0.0-0.1)	358 (203-594)	3.5 (2.0-5.8)	1 (0-2)	0.0 (0.0-0.0)	72 (35-135)	0.7 (0.3-1.3)	1 (1-2)	0.0 (0.0-0.0)	117 (57-212)	0.3 (0.1-0.5)	0 (0-1)	0.0 (0.0-0.0)	23 (10-47)	0.1 (0.0-0.1)	961 (565-1520)	0.1 (0.1-0.1)	19200 (11400-30600)	1.9 (1.1-3.0)	195 (93-358)	0.0 (0.0-0.0)	3890 (1860-7140)	0.4 (0.2-0.7)
Serratia spp.	Carbapenem	4 (2-6)	0.5 (0.3-0.7)	360 (219-568)	42.1 (25.7-66.4)	1 (1-2)	0.1 (0.1-0.2)	95 (48-173)	11.1 (5.6-20.3)	1 (1-2)	0.0 (0.0-0.0)	133 (73-216)	1.3 (0.7-2.1)	0 (0-1)	0.0 (0.0-0.0)	35 (17-65)	0.3 (0.2-0.6)	1 (0-1)	0.0 (0.0-0.0)	47 (23-86)	0.1 (0.0-0.2)	0 (0-0)	0.0 (0.0-0.0)	13 (5-25)	0.0 (0.0-0.1)	456 (271-704)	0.0 (0.0-0.1)	9020 (5350-14100)	0.9 (0.5-1.4)	124 (64-222)	0.0 (0.0-0.0)	2440 (1260-4400)	0.2 (0.1-0.4)
Serratia spp.	Fluoroquinolones	7 (4-12)	0.9 (0.5-1.5)	666 (400-1110)	77.9 (46.7-129.5)	1 (0-2)	0.1 (0.0-0.2)	74 (13-167)	8.6 (1.5-19.6)	3 (1-4)	0.0 (0.0-0.0)	234 (133-387)	2.3 (1.3-3.8)	0 (0-1)	0.0 (0.0-0.0)	26 (5-61)	0.3 (0.1-0.6)	1 (0-2)	0.0 (0.0-0.0)	79 (39-138)	0.2 (0.1-0.3)	0 (0-0)	0.0 (0.0-0.0)	9 (2-22)	0.0 (0.0-0.0)	660 (393-1030)	0.1 (0.0-0.1)	13500 (8010-21100)	1.3 (0.8-2.1)	75 (13-166)	0.0 (0.0-0.0)	1550 (303-3500)	0.2 (0.0-0.3)
Serratia spp.	Fourth-generation cephalosporins	9 (5-15)	1.1 (0.6-1.7)	815 (473-1290)	95.3 (55.3-151.3)	1 (0-2)	0.1 (0.0-0.2)	70 (28-136)	8.2 (3.3-16.0)	3 (2-5)	0.0 (0.0-0.0)	267 (148-451)	2.6 (1.4-4.4)	0 (0-1)	0.0 (0.0-0.0)	23 (9-46)	0.2 (0.1-0.4)	1 (0-2)	0.0 (0.0-0.0)	75 (36-135)	0.2 (0.1-0.3)	0 (0-0)	0.0 (0.0-0.0)	6 (2-14)	0.0 (0.0-0.0)	427 (257-674)	0.0 (0.0-0.1)	8850 (5210-14000)	0.9 (0.5-1.4)	34 (14-66)	0.0 (0.0-0.0)	705 (298-1360)	0.1 (0.0-0.1)
Serratia spp.	Third-generation cephalosporins	19 (11-29)	2.2 (1.3-3.4)	1670 (1010-2620)	195.2 (117.9-306.0)	1 (0-3)	0.1 (0.1-0.3)	112 (39-228)	13.1 (4.6-26.7)	7 (4-11)	0.1 (0.0-0.1)	592 (346-957)	5.8 (3.4-9.3)	0 (0-1)	0.0 (0.0-0.0)	42 (14-86)	0.4 (0.1-0.8)	2 (1-4)	0.0 (0.0-0.0)	191 (95-346)	0.4 (0.2-0.8)	0 (0-0)	0.0 (0.0-0.0)	14 (5-31)	0.0 (0.0-0.1)	1640 (986-2550)	0.2 (0.1-0.2)	32700 (19700-50900)	3.2 (1.9-5.0)	149 (55-294)	0.0 (0.0-0.0)	2920 (1090-5750)	0.3 (0.1-0.6)
Serratia spp.	Resistance to one or more antibiotics	26 (16-40)	3.1 (1.9-4.7)	2320 (1440-3590)	271.4 (168.5-419.3)	7 (4-12)	0.8 (0.4-1.4)	594 (325-1040)	69.5 (38.0-121.3)	9 (5-15)	0.1 (0.1-0.1)	821 (471-1320)	8.0 (4.6-12.8)	2 (1-4)	0.0 (0.0-0.0)	211 (109-366)	2.0 (1.1-3.6)	3 (2-5)	0.0 (0.0-0.0)	273 (136-472)	0.6 (0.3-1.0)	1 (0-2)	0.0 (0.0-0.0)	70 (32-134)	0.2 (0.1-0.3)	2340 (1430-3590)	0.2 (0.1-0.3)	47000 (28300-71800)	4.6 (2.8-7.0)	603 (326-1030)	0.1 (0.0-0.1)	12100 (6510-20300)	1.2 (0.6-2.0)
Shigella spp.	Fluoroquinolones	0 (0-1)	0.0 (0.0-0.1)	27 (9-62)	3.2 (1.1-7.3)	0 (0-0)	0.0 (0.0-0.0)	5 (1-15)	0.6 (0.1-1.7)	1 (0-2)	0.0 (0.0-0.0)	138 (42-309)	1.3 (0.4-3.0)	0 (0-0)	0.0 (0.0-0.0)	21 (3-59)	0.2 (0.0-0.6)	0 (0-1)	0.0 (0.0-0.0)	228 (62-608)	0.5 (0.1-1.3)	0 (0-0)	0.0 (0.0-0.0)	30 (0-90)	0.1 (0.0-0.2)	115 (43-252)	0.0 (0.0-0.0)	3410 (1330-7490)	0.3 (0.1-0.7)	24 (3-63)	0.0 (0.0-0.0)	517 (82-1360)	0.1 (0.0-0.1)
Shigella spp.	Resistance to one or more antibiotics	0 (0-1)	0.0 (0.0-0.1)	27 (9-62)	3.2 (1.1-7.3)	0 (0-0)	0.0 (0.0-0.0)	5 (1-15)	0.6 (0.1-1.7)	1 (0-2)	0.0 (0.0-0.0)	138 (42-309)	1.3 (0.4-3.0)	0 (0-0)	0.0 (0.0-0.0)	21 (3-59)	0.2 (0.0-0.6)	0 (0-1)	0.0 (0.0-0.0)	228 (62-608)	0.5 (0.1-1.3)	0 (0-0)	0.0 (0.0-0.0)	30 (0-90)	0.1 (0.0-0.2)	115 (43-252)	0.0 (0.0-0.0)	3410 (1330-7490)	0.3 (0.1-0.7)	24 (3-63)	0.0 (0.0-0.0)	517 (82-1360)	0.1 (0.0-0.1)
Staphylococcus aureus	Fluoroquinolones	153 (92-240)	17.9 (10.7-28.1)	13600 (8150-21400)	1595.2 (952.7-2500.0)	7 (3-14)	0.8 (0.3-1.6)	631 (248-1200)	73.8 (29.0-140.6)	146 (97-210)	1.4 (0.9-2.0)	13000 (8590-18700)	126.3 (83.5-181.5)	7 (3-12)	0.1 (0.0-0.1)	598 (258-1050)	5.8 (2.5-10.2)	65 (41-96)	0.1 (0.1-0.2)	5810 (3730-8610)	12.7 (8.2-18.8)	3 (1-5)	0.0 (0.0-0.0)	265 (114-478)	0.6 (0.2-1.0)	84100 (62200-113000)	8.2 (6.1-11.0)	1340000 (949000-1860000)	130.8 (92.4-180.8)	3890 (1700-6660)	0.4 (0.2-0.6)	61900 (27300-108000)	6.0 (2.7-10.5)
Staphylococcus aureus	Macrolide	230 (138-357)	26.9 (16.1-41.8)	20500 (12200-31800)	2394.5 (1431.8-3715.2)	9 (3-18)	1.1 (0.4-2.1)	815 (280-1570)	95.3 (32.8-184.1)	210 (139-299)	2.0 (1.4-2.9)	18700 (12400-26500)	181.8 (120.4-257.9)	8 (3-15)	0.1 (0.0-0.1)	743 (276-1350)	7.2 (2.7-13.1)	90 (58-132)	0.2 (0.1-0.3)	8120 (5330-11900)	17.7 (11.6-26.0)	4 (1-7)	0.0 (0.0-0.0)	323 (125-607)	0.7 (0.3-1.3)	110000 (81800-149000)	10.8 (8.0-14.5)	1790000 (1260000-2480000)	173.9 (123.1-241.4)	4400 (1740-7880)	0.4 (0.2-0.8)	71300 (27400-130000)	6.9 (2.7-12.7)
Staphylococcus aureus	Methicillin	231 (139-362)	27.0 (16.3-42.3)	20600 (12400-32200)	2405.1 (1447.9-3762.8)	62 (25-116)	7.3 (3.0-13.6)	5520 (2250-10400)	645.5 (262.9-1210.5)	208 (139-297)	2.0 (1.3-2.9)	18400 (12300-26500)	179.2 (119.8-257.2)	55 (24-96)	0.5 (0.2-0.9)	4920 (2100-8540)	47.8 (20.4-83.0)	87 (56-131)	0.2 (0.1-0.3)	7820 (5150-11600)	17.1 (11.2-25.3)	23 (10-42)	0.1 (0.0-0.1)	2050 (892-3670)	4.5 (1.9-8.0)	103000 (75500-138000)	10.0 (7.3-13.4)	1660000 (1180000-2290000)	161.2 (115.0-223.1)	27200 (12100-46300)	2.6 (1.2-4.5)	437000 (190000-754000)	42.5 (18.5-73.5)
Staphylococcus aureus	Trimethoprim-Sulfamethoxazole	16 (10-26)	1.9 (1.1-3.0)	1460 (858-2300)	170.5 (100.3-269.1)	1 (1-2)	0.2 (0.1-0.3)	124 (57-222)	14.5 (6.7-25.9)	15 (10-21)	0.1 (0.1-0.2)	1300 (869-1840)	12.7 (8.5-17.9)	1 (1-2)	0.0 (0.0-0.0)	111 (55-187)	1.1 (0.5-1.8)	6 (4-9)	0.0 (0.0-0.0)	554 (357-818)	1.2 (0.8-1.8)	1 (0-1)	0.0 (0.0-0.0)	47 (23-81)	0.1 (0.1-0.2)	6930 (5080-9410)	0.7 (0.5-0.9)	116000 (80300-164000)	11.3 (7.8-15.9)	628 (311-1000)	0.1 (0.0-0.1)	10300 (5010-16900)	1.0 (0.5-1.6)
Staphylococcus aureus	Vancomycin	3 (2-5)	0.4 (0.2-0.6)	294 (173-470)	34.4 (20.2-54.9)	1 (1-2)	0.1 (0.1-0.3)	102 (45-205)	11.9 (5.3-24.0)	3 (2-4)	0.0 (0.0-0.0)	258 (169-377)	2.5 (1.6-3.7)	1 (0-2)	0.0 (0.0-0.0)	93 (44-173)	0.9 (0.4-1.7)	1 (1-2)	0.0 (0.0-0.0)	109 (70-165)	0.2 (0.2-0.4)	0 (0-1)	0.0 (0.0-0.0)	41 (20-76)	0.1 (0.0-0.2)	1380 (986-1930)	0.1 (0.1-0.2)	22400 (15200-32600)	2.2 (1.5-3.2)	583 (289-1010)	0.1 (0.0-0.1)	9240 (4480-16700)	0.9 (0.4-1.6)
Staphylococcus aureus	Resistance to one or more antibiotics	321 (194-495)	37.6 (22.7-57.8)	28600 (17300-44000)	3340.7 (2021.1-5143.6)	81 (40-144)	9.5 (4.7-16.8)	7190 (3600-12800)	841.0 (420.7-1495.5)	292 (196-418)	2.8 (1.9-4.1)	25900 (17400-37100)	252.0 (169.2-360.5)	73 (38-120)	0.7 (0.4-1.2)	6460 (3380-10600)	62.8 (32.8-103.3)	125 (80-185)	0.3 (0.2-0.4)	11200 (7360-16400)	24.5 (16.1-35.8)	31 (16-53)	0.1 (0.0-0.1)	2730 (1420-4620)	6.0 (3.1-10.1)	152000 (113000-203000)	14.8 (11.0-19.7)	2450000 (1740000-3390000)	238.7 (169.2-330.1)	36700 (20400-57400)	3.6 (2.0-5.6)	589000 (325000-948000)	57.4 (31.7-92.3)
Streptococcus pneumoniae	Beta Lactam/Beta-lactamase inhibitors	23 (13-37)	2.6 (1.5-4.3)	2010 (1140-3300)	234.9 (133.2-386.5)	0 (0-1)	0.0 (0.0-0.1)	38 (1-101)	4.4 (0.1-11.8)	43 (30-59)	0.4 (0.3-0.6)	3790 (2640-5250)	36.9 (25.7-51.1)	1 (0-2)	0.0 (0.0-0.0)	70 (2-173)	0.7 (0.0-1.7)	17 (12-24)	0.0 (0.0-0.1)	1540 (1110-2140)	3.4 (2.4-4.7)	0 (0-1)	0.0 (0.0-0.0)	26 (1-67)	0.1 (0.0-0.1)	5630 (4390-7310)	0.5 (0.4-0.7)	92600 (70000-125000)	9.0 (6.8-12.2)	91 (3-212)	0.0 (0.0-0.0)	1450 (66-3480)	0.1 (0.0-0.3)
Streptococcus pneumoniae	Carbapenem	49 (29-81)	5.8 (3.4-9.5)	4390 (2560-7190)	513.5 (299.1-841.1)	12 (5-22)	1.3 (0.6-2.6)	1030 (438-1960)	119.9 (51.3-228.8)	93 (69-127)	0.9 (0.7-1.2)	8260 (6120-11200)	80.3 (59.5-109.3)	22 (10-38)	0.2 (0.1-0.4)	1940 (910-3350)	18.8 (8.9-32.6)	40 (29-56)	0.1 (0.1-0.1)	3600 (2630-4950)	7.9 (5.7-10.8)	10 (5-17)	0.0 (0.0-0.0)	851 (426-1440)	1.9 (0.9-3.2)	14600 (11500-18400)	1.4 (1.1-1.8)	235000 (180000-310000)	22.9 (17.5-30.2)	3520 (1710-5900)	0.3 (0.2-0.6)	56500 (27200-95200)	5.5 (2.7-9.3)
Streptococcus pneumoniae	Fluoroquinolones	17 (10-27)	1.9 (1.1-3.2)	1480 (865-2420)	172.8 (101.2-283.1)	2 (1-5)	0.3 (0.1-0.6)	218 (46-487)	25.6 (5.4-56.9)	28 (20-40)	0.3 (0.2-0.4)	2510 (1740-3510)	24.4 (16.9-34.2)	4 (1-9)	0.0 (0.0-0.1)	362 (80-759)	3.5 (0.8-7.4)	14 (10-21)	0.0 (0.0-0.0)	1280 (901-1830)	2.8 (2.0-4.0)	2 (0-5)	0.0 (0.0-0.0)	186 (42-389)	0.4 (0.1-0.9)	6450 (4900-8460)	0.6 (0.5-0.8)	98600 (72300-134000)	9.6 (7.0-13.0)	986 (223-1910)	0.1 (0.0-0.2)	14900 (3310-29300)	1.4 (0.3-2.9)
Streptococcus pneumoniae	Macrolide	87 (51-141)	10.2 (5.9-16.5)	7740 (4500-12500)	905.3 (525.7-1464.5)	4 (0-11)	0.5 (0.0-1.3)	342 (0-1020)	40.0 (0.0-119.1)	172 (129-229)	1.7 (1.3-2.2)	15300 (11400-20300)	148.5 (111.2-197.3)	8 (0-21)	0.1 (0.0-0.2)	680 (0-1830)	6.6 (0.0-17.8)	80 (60-108)	0.2 (0.1-0.2)	7090 (5420-9580)	15.5 (11.8-20.9)	4 (0-10)	0.0 (0.0-0.0)	309 (0-836)	0.7 (0.0-1.8)	34300 (28000-41500)	3.3 (2.7-4.0)	506000 (405000-635000)	49.3 (39.5-61.8)	1610 (0-4230)	0.2 (0.0-0.4)	23200 (0-60800)	2.3 (0.0-5.9)
Streptococcus pneumoniae	Penicillin	62 (35-102)	7.3 (4.1-12.0)	5520 (3090-9150)	645.1 (361.9-[illegible]	5 (2-9)	0.5 (0.2-1.0)	405 (186-768)	47.3 (21.7-89.8)	112 (83-150)	1.1 (0.8-1.5)	9940 (7370-13300)	96.6 (71.6-129.6)	8 (4-13)	0.1 (0.0-0.1)	710 (385-1150)	6.9 (3.7-11.2)	50 (36-68)	0.1 (0.1-0.1)	4440 (3240-6040)	9.7 (7.1-13.2)	4 (2-6)	0.0 (0.0-0.0)	319 (174-539)	0.7 (0.4-1.2)	17100 (13400-[illegible]	1.7 (1.3-2.1)	286000 (216000-[illegible]	27.8 (21.0-36.0)	1230 (706-1950)	0.1 (0.1-0.2)	20700 (11800-[illegible]	2.0 (1.1-3.2)
Streptococcus pneumoniae	Third-generation cephalosporins	[illegible]	[illegible]	[illegible]	[illegible]	[illegible]	[illegible]	[illegible]	[illegible]	[illegible]	[illegible]	[illegible]	[illegible]	[illegible]	[illegible]	[illegible]	[illegible]	[illegible]	[illegible]	[illegible]	[illegible]	[illegible]	[illegible]	[illegible]	[illegible]	[illegible]	[illegible]	[illegible]	[illegible]	[illegible]	[illegible]	[illegible]	[illegible]
Streptococcus pneumoniae	Trimethoprim-Sulfamethoxazole	[illegible]	[illegible]	[illegible]	[illegible]	[illegible]	[illegible]	[illegible]	[illegible]	[illegible]	[illegible]	[illegible]	[illegible]	[illegible]	[illegible]	[illegible]	[illegible]	[illegible]	[illegible]	[illegible]	[illegible]	[illegible]	[illegible]	[illegible]	[illegible]	[illegible]	[illegible]	[illegible]	[illegible]	[illegible]	[illegible]	[illegible]	[illegible]
Streptococcus pneumoniae	Resistance to one or more antibiotics	145 (84-232)	16.9 (9.9-27.2)	12900 (7500-20700)	1506.9 (877.7-2416.4)	30 (16-50)	3.5 (1.8-5.9)	2630 (1400-4460)	307.7 (163.3-521.9)	274 (207-362)	2.7 (2.0-3.5)	24400 (18400-32100)	237.0 (178.4-312.1)	55 (35-80)	0.5 (0.3-0.8)	4880 (3160-7090)	47.5 (30.7-68.9)	124 (95-166)	0.3 (0.2-0.4)	11100 (8490-14700)	24.2 (18.5-32.2)	25 (16-36)	0.1 (0.0-0.1)	2160 (1400-3130)	4.7 (3.1-6.8)	50100 (41300-60800)	4.9 (4.0-5.9)	762000 (609000-960000)	74.2 (59.3-93.4)	9310 (6160-12900)	0.9 (0.6-1.3)	146000 (96600-206000)	14.2 (9.4-20.1)

Table S16. Deaths and DALYs (in counts and age-specific rates) associated with and attributable to bacterial antimicrobial resistance by pathogen–drug combination, by age group, 2019, Latin America and the Caribbean

Pathogen	Antibiotic Class	Neonatal							
		Associated with resistance				Attributable to resistance			
		Deaths		DALYs		Deaths		DALYs	
		Counts	Rate per 100 000	Counts	Rate per 100 000	Counts	Rate per 100 000	Counts	Rate per 100 000
All pathogens	Resistance to one or more antibiotics	15800 (10800-22100)	2142.1 (1462.3-2985.4)	1410000 (962000-1970000)	190601.1 (130097.0-265681.3)	3890 (2640-5410)	525.9 (356.9-731.4)	346000 (235000-481000)	46783.3 (31741.9-65049.1)
Acinetobacter baumannii	Aminoglycosides	764 (505-1090)	103.2 (68.3-147.8)	67900 (45000-97200)	9182.0 (6078.7-13144.6)	32 (0-74)	4.3 (0.0-10.0)	2840 (6-6620)	384.2 (0.8-894.3)
Acinetobacter baumannii	Anti-pseudomonal penicillin/Beta-Lactamase inhibitors	973 (645-1380)	131.5 (87.2-186.6)	86500 (57400-123000)	11698.3 (7754.6-16591.0)	21 (10-36)	2.8 (1.4-4.9)	1850 (928-3200)	250.0 (125.4-432.0)
Acinetobacter baumannii	Beta Lactam/Beta-lactamase inhibitors	883 (589-1240)	119.4 (79.7-167.9)	78500 (52400-111000)	10617.1 (7089.5-14938.5)	2 (0-4)	0.2 (0.1-0.6)	154 (38-366)	20.8 (5.1-49.5)
Acinetobacter baumannii	Carbapenem	915 (611-1290)	123.6 (82.6-174.3)	81400 (54400-115000)	10997.3 (7348.5-15499.7)	153 (85-250)	20.6 (11.5-33.8)	13600 (7540-22200)	1835.1 (1019.9-3003.3)
Acinetobacter baumannii	Fluoroquinolones	944 (629-1340)	127.6 (85.0-180.5)	83900 (55900-119000)	11347.6 (7561.1-16053.0)	118 (63-193)	15.9 (8.5-26.0)	10500 (5620-17100)	1417.9 (759.7-2314.9)
Acinetobacter baumannii	Fourth-generation cephalosporins	990 (657-1410)	133.8 (88.8-190.1)	88100 (58400-125000)	11904.6 (7898.8-16911.4)	5 (2-11)	0.7 (0.2-1.4)	439 (151-934)	59.3 (20.5-126.3)
Acinetobacter baumannii	Third-generation cephalosporins	1000 (672-1420)	135.6 (90.8-192.5)	89200 (59800-127000)	12064.0 (8082.4-17112.8)	13 (5-23)	1.7 (0.7-3.1)	1130 (431-2050)	152.3 (58.2-277.6)
Acinetobacter baumannii	Resistance to one or more antibiotics	1080 (718-1530)	145.8 (97.1-206.2)	96000 (63900-136000)	12972.1 (8634.7-18340.4)	343 (218-509)	46.3 (29.5-68.8)	30500 (19400-45300)	4119.6 (2621.7-6118.4)
Citrobacter spp.	Aminoglycosides	19 (10-31)	2.5 (1.4-4.1)	1650 (933-2730)	222.9 (126.1-368.9)	1 (0-3)	0.2 (0.0-0.4)	100 (0-251)	13.5 (0.0-33.9)
Citrobacter spp.	Anti-pseudomonal penicillin/Beta-Lactamase inhibitors	31 (17-51)	4.1 (2.3-6.9)	2730 (1550-4550)	368.4 (209.0-614.4)	6 (3-12)	0.9 (0.4-1.6)	567 (267-1060)	76.7 (36.0-143.0)
Citrobacter spp.	Carbapenem	14 (8-23)	1.9 (1.1-3.1)	1240 (724-2060)	167.8 (97.9-278.2)	3 (2-7)	0.5 (0.2-0.9)	310 (148-583)	41.9 (20.1-78.8)
Citrobacter spp.	Fluoroquinolones	35 (20-57)	4.7 (2.7-7.7)	3100 (1800-5060)	419.6 (243.0-683.5)	5 (2-10)	0.7 (0.3-1.4)	480 (194-906)	64.8 (26.3-122.4)
Citrobacter spp.	Fourth-generation cephalosporins	39 (23-66)	5.3 (3.1-8.9)	3470 (2010-5870)	468.6 (271.9-793.2)	4 (2-8)	0.5 (0.2-1.0)	351 (137-691)	47.4 (18.6-93.3)
Citrobacter spp.	Third-generation cephalosporins	75 (44-121)	10.1 (6.0-16.3)	6630 (3930-10700)	896.6 (531.3-1449.7)	8 (3-16)	1.1 (0.4-2.2)	744 (290-1470)	100.6 (39.1-198.2)
Citrobacter spp.	Resistance to one or more antibiotics	101 (60-163)	13.7 (8.1-22.0)	9000 (5340-14500)	1216.2 (722.0-1959.7)	29 (15-49)	3.9 (2.0-6.7)	2550 (1300-4390)	345.0 (175.3-593.5)
Enterobacter spp.	Aminoglycosides	192 (123-284)	26.0 (16.7-38.4)	17100 (11000-25200)	2309.3 (1482.7-3412.9)	12 (3-24)	1.6 (0.5-3.2)	1090 (298-2100)	146.8 (40.4-284.0)
Enterobacter spp.	Anti-pseudomonal penicillin/Beta-Lactamase inhibitors	423 (281-609)	57.2 (38.0-82.3)	37600 (25000-54100)	5084.6 (3381.6-7318.8)	47 (26-74)	6.4 (3.6-10.0)	4200 (2350-6580)	568.1 (317.2-889.2)
Enterobacter spp.	Carbapenem	225 (141-325)	30.4 (19.1-44.0)	20000 (12600-28900)	2702.4 (1700.9-3908.8)	55 (32-86)	7.4 (4.3-11.7)	4900 (2810-7680)	661.8 (380.1-1038.7)
Enterobacter spp.	Fluoroquinolones	342 (222-503)	46.2 (30.0-68.0)	30400 (19800-44700)	4109.7 (2671.0-6047.8)	40 (21-68)	5.4 (2.8-9.1)	3530 (1840-6010)	477.2 (248.9-812.6)
Enterobacter spp.	Fourth-generation cephalosporins	581 (390-840)	78.5 (52.7-113.5)	51700 (34700-74700)	6983.4 (4690.2-10096.4)	39 (19-69)	5.2 (2.5-9.3)	3450 (1670-6100)	466.0 (225.1-824.0)
Enterobacter spp.	Trimethoprim-Sulfamethoxazole	548 (352-809)	74.1 (47.6-109.3)	48700 (31300-71900)	6588.9 (4230.1-9722.5)	33 (0-70)	4.4 (0.0-9.4)	2920 (0-6200)	394.1 (0.0-837.5)
Enterobacter spp.	Resistance to one or more antibiotics	983 (652-1410)	132.9 (88.2-190.5)	87400 (58000-125000)	11816.0 (7840.2-16939.5)	226 (142-333)	30.5 (19.3-45.0)	20100 (12700-29600)	2713.9 (1711.7-4000.0)
Enterococcus faecalis	Fluoroquinolones	302 (206-427)	40.8 (27.8-57.7)	26800 (18300-38000)	3627.9 (2476.7-5132.5)	74 (34-126)	10.0 (4.6-17.1)	6550 (3030-11200)	885.7 (409.0-1518.8)
Enterococcus faecalis	Vancomycin	66 (44-100)	9.0 (5.9-13.5)	5910 (3910-8910)	798.8 (528.6-1204.7)	22 (10-39)	2.9 (1.3-5.2)	1940 (862-3430)	261.9 (116.6-464.3)
Enterococcus faecalis	Resistance to one or more antibiotics	336 (231-474)	45.4 (31.2-64.1)	29900 (20600-42200)	4038.8 (2780.3-5706.3)	95 (52-155)	12.9 (7.1-20.9)	8490 (4670-13800)	1147.6 (631.3-1862.6)
Enterococcus faecium	Fluoroquinolones	398 (274-571)	53.7 (37.0-77.2)	35400 (24400-50900)	4783.4 (3293.9-6874.5)	65 (20-120)	8.8 (2.7-16.3)	5800 (1810-10700)	784.6 (244.0-1445.3)
Enterococcus faecium	Vancomycin	236 (161-338)	31.9 (21.8-45.7)	21000 (14300-30100)	2838.3 (1935.0-4068.5)	59 (33-96)	8.0 (4.5-13.0)	5280 (2940-8560)	713.7 (397.0-1157.7)
Enterococcus faecium	Resistance to one or more antibiotics	420 (289-599)	56.7 (39.0-80.9)	37400 (25700-53300)	5050.1 (3473.6-7200.6)	125 (77-186)	16.8 (10.5-25.2)	11100 (6890-16600)	1498.2 (931.6-2240.4)
Other enterococci	Fluoroquinolones	454 (284-709)	61.4 (38.4-95.9)	40400 (25300-63000)	5456.5 (3416.2-8522.4)	83 (12-170)	11.2 (1.7-23.0)	7390 (1110-15100)	999.3 (149.9-2041.4)
Other enterococci	Vancomycin	92 (57-140)	12.4 (7.7-19.0)	8160 (5040-12500)	1102.4 (681.5-1687.1)	15 (8-29)	2.1 (1.0-4.0)	1360 (673-2610)	184.5 (90.9-353.5)
Other enterococci	Resistance to one or more antibiotics	455 (284-711)	61.5 (38.5-96.1)	40500 (25300-63200)	5468.1 (3419.3-8543.8)	99 (35-184)	13.3 (4.8-24.9)	8760 (3130-16300)	1183.8 (423.7-2209.6)
Escherichia coli	Aminoglycosides	406 (274-583)	54.8 (37.0-78.8)	36100 (24400-51900)	4883.5 (3296.0-7021.1)	25 (15-37)	3.3 (2.1-4.9)	2180 (1350-3260)	295.2 (183.1-440.8)
Escherichia coli	Aminopenicillin	2040 (1360-2940)	276.4 (184.2-398.0)	182000 (121000-263000)	24614.9 (16409.0-35494.8)	31 (19-47)	4.2 (2.6-6.4)	2770 (1690-4220)	374.5 (229.0-569.8)
Escherichia coli	Beta Lactam/Beta-lactamase inhibitors	1600 (1070-2310)	216.3 (144.9-311.9)	142000 (95500-205000)	19262.9 (12903.2-27774.7)	74 (47-111)	10.0 (6.3-15.0)	6600 (4170-9870)	892.2 (563.7-1334.1)
Escherichia coli	Carbapenem	125 (79-185)	16.9 (10.7-25.0)	11100 (7050-16500)	1503.3 (953.1-2230.6)	27 (15-45)	3.7 (2.0-6.1)	2440 (1290-4030)	329.5 (174.8-545.1)
Escherichia coli	Fluoroquinolones	1300 (876-1860)	175.8 (118.5-251.8)	116000 (78100-166000)	15657.7 (10553.1-22439.4)	136 (87-202)	18.4 (11.8-27.4)	12100 (7740-18000)	1638.6 (1046.6-2436.1)
Escherichia coli	Third-generation cephalosporins	1110 (737-1610)	150.2 (99.6-217.4)	99000 (65600-143000)	13380.6 (8871.2-19350.4)	152 (66-290)	20.6 (8.9-39.2)	13600 (5880-25800)	1833.8 (795.0-3488.3)
Escherichia coli	Trimethoprim-Sulfamethoxazole	1560 (1050-2260)	210.8 (142.1-305.7)	139000 (93600-201000)	18775.7 (12650.5-27224.4)	93 (59-141)	12.6 (8.0-19.0)	8310 (5240-12500)	1123.8 (708.1-1692.0)
Escherichia coli	Resistance to one or more antibiotics	2210 (1470-3180)	298.2 (199.2-430.1)	196000 (131000-284000)	26558.0 (17757.5-38324.4)	539 (344-814)	72.9 (46.5-110.0)	48000 (30600-72500)	6487.7 (4139.7-9794.5)
Group A *Streptococcus*	Macrolide	86 (57-129)	11.6 (7.7-17.4)	7650 (5080-11500)	1034.6 (686.6-1548.3)	8 (0-27)	1.1 (0.0-3.6)	726 (0-2400)	98.1 (0.0-323.9)
Group A *Streptococcus*	Resistance to one or more antibiotics	86 (57-129)	11.6 (7.7-17.4)	7650 (5080-11500)	1034.6 (686.6-1548.3)	8 (0-27)	1.1 (0.0-3.6)	726 (0-2400)	98.1 (0.0-323.9)
Group B *Streptococcus*	Fluoroquinolones	259 (165-389)	35.0 (22.3-52.6)	23100 (14700-34600)	3117.9 (1981.7-4682.2)	45 (7-109)	6.1 (1.0-14.7)	4040 (641-9650)	545.7 (86.7-1304.4)
Group B Streptococcus	Macrolide	727 (463-1090)	98.3 (62.6-147.3)	64700 (41200-97000)	8743.6 (5568.3-13108.2)	73 (0-203)	9.9 (0.0-27.4)	6500 (0-18000)	878.1 (0.0-2433.4)
Group B Streptococcus	Penicillin	28 (17-42)	3.8 (2.3-5.7)	2470 (1520-3740)	334.4 (205.0-504.9)	6 (1-13)	0.8 (0.2-1.7)	510 (102-1150)	69.0 (13.8-155.5)
Group B Streptococcus	Resistance to one or more antibiotics	868 (562-1280)	117.4 (76.0-173.0)	77300 (50100-114000)	10447.4 (6766.3-15398.6)	124 (13-272)	16.8 (1.7-36.7)	11000 (1160-24200)	1492.8 (157.4-3265.8)
Haemophilus influenzae	Aminopenicillin	98 (58-150)	13.2 (7.8-20.2)	8690 (5170-13300)	1175.3 (698.6-1802.1)	16 (1-34)	2.1 (0.2-4.7)	1390 (102-3070)	187.5 (13.8-414.9)
Haemophilus influenzae	Third-generation cephalosporins	29 (17-46)	3.9 (2.3-6.2)	2590 (1510-4080)	350.2 (204.5-551.5)	8 (3-17)	1.1 (0.5-2.3)	755 (310-1510)	102.0 (41.9-203.8)
Haemophilus influenzae	Resistance to one or more antibiotics	113 (69-170)	15.2 (9.3-23.0)	10000 (6140-15100)	1356.7 (829.7-2046.3)	24 (8-46)	3.3 (1.0-6.2)	2140 (686-4070)	289.5 (92.7-549.9)
Klebsiella pneumoniae	Aminoglycosides	1500 (1030-2080)	202.4 (138.8-280.9)	133000 (91300-185000)	17998.5 (12342.3-24988.5)	115 (68-175)	15.6 (9.2-23.7)	10200 (6030-15600)	1384.4 (815.6-2106.6)
Klebsiella pneumoniae	Beta Lactam/Beta-lactamase inhibitors	2770 (1900-3800)	374.9 (256.8-513.3)	247000 (169000-338000)	33343.8 (22838.2-45659.0)	70 (37-115)	9.5 (5.0-15.6)	6240 (3270-10200)	843.2 (441.8-1385.4)
Klebsiella pneumoniae	Carbapenem	704 (437-1050)	95.2 (59.0-141.6)	62600 (38800-93100)	8461.4 (5245.8-12591.5)	165 (96-259)	22.3 (13.0-35.1)	14600 (8550-23000)	1978.5 (1156.1-3115.8)
Klebsiella pneumoniae	Fluoroquinolones	1870 (1290-2570)	253.0 (173.8-347.7)	166000 (114000-229000)	22502.2 (15454.4-30923.2)	130 (76-200)	17.6 (10.2-27.1)	11600 (6740-17800)	1564.9 (911.4-2406.8)
Klebsiella pneumoniae	Third-generation cephalosporins	2320 (1560-3280)	313.6 (211.2-442.9)	206000 (139000-291000)	27891.5 (18782.1-39392.3)	275 (100-521)	37.2 (13.5-70.5)	24400 (8890-46300)	3304.0 (1201.3-6263.7)
Klebsiella pneumoniae	Trimethoprim-Sulfamethoxazole	2320 (1590-3190)	313.3 (215.4-431.2)	206000 (142000-284000)	27864.1 (19151.5-38349.5)	115 (56-196)	15.5 (7.6-26.5)	10200 (4970-17400)	1378.5 (672.4-2356.9)
Klebsiella pneumoniae	Resistance to one or more antibiotics	3170 (2180-4360)	428.3 (295.0-588.8)	282000 (194000-387000)	38090.6 (26236.7-52370.5)	870 (549-1270)	117.5 (74.3-171.6)	77300 (48800-113000)	10453.5 (6602.9-15264.1)
Morganella spp.	Fluoroquinolones	0 (0-1)	0.0 (0.0-0.1)	20 (6-54)	2.7 (0.8-7.3)	0 (0-0)	0.0 (0.0-0.0)	4 (1-11)	0.5 (0.1-1.4)
Morganella spp.	Fourth-generation cephalosporins	0 (0-0)	0.0 (0.0-0.0)	6 (2-17)	0.8 (0.3-2.3)	0 (0-0)	0.0 (0.0-0.0)	1 (0-3)	0.1 (0.0-0.5)
Morganella spp.	Third-generation cephalosporins	0 (0-0)	0.0 (0.0-0.0)	6 (2-16)	0.8 (0.2-2.2)	0 (0-0)	0.0 (0.0-0.0)	1 (0-2)	0.1 (0.0-0.3)
Morganella spp.	Resistance to one or more antibiotics	0 (0-1)	0.0 (0.0-0.1)	23 (7-63)	3.2 (0.9-8.5)	0 (0-0)	0.0 (0.0-0.0)	5 (1-15)	0.7 (0.2-2.0)
Mycobacterium tuberculosis	Extensive drug resistance in TB	--	--	0 (0-0)	0.0 (0.0-0.0)	--	--	0 (0-0)	0.0 (0.0-0.0)
Mycobacterium tuberculosis	Isoniazid mono-resistance	--	--	0 (0-0)	0.0 (0.0-0.0)	--	--	0 (0-0)	0.0 (0.0-0.0)
Mycobacterium tuberculosis	Multi-drug resistance excluding extensive drug resistance in TB	--	--	0 (0-0)	0.0 (0.0-0.0)	--	--	0 (0-0)	0.0 (0.0-0.0)
Mycobacterium tuberculosis	Rifampicin mono-resistance	--	--	0 (0-0)	0.0 (0.0-0.0)	--	--	0 (0-0)	0.0 (0.0-0.0)
Mycobacterium tuberculosis	Resistance to one or more antibiotics	--	--	0 (0-0)	0.0 (0.0-0.0)	--	--	0 (0-0)	0.0 (0.0-0.0)
Neisseria gonorrhoeae	Fluoroquinolones	--	--	--	--	--	--	--	--
Neisseria gonorrhoeae	Third-generation cephalosporins	--	--	--	--	--	--	--	--
Neisseria gonorrhoeae	Resistance to one or more antibiotics	--	--	--	--	--	--	--	--
Proteus spp.	Aminoglycosides	8 (5-11)	1.0 (0.7-1.5)	673 (439-1000)	91.0 (59.4-135.8)	0 (0-1)	0.1 (0.0-0.1)	38 (4-83)	5.1 (0.6-11.2)
Proteus spp.	Aminopenicillin	99 (66-145)	13.4 (8.9-19.6)	8820 (5860-12900)	1191.9 (791.9-1745.0)	2 (0-7)	0.3 (0.0-1.0)	207 (0-627)	28.0 (0.0-84.7)
Proteus spp.	Fluoroquinolones	39 (26-58)	5.3 (3.5-7.9)	3480 (2280-5180)	469.8 (308.8-700.1)	3 (1-6)	0.4 (0.1-0.8)	257 (98-502)	34.7 (13.2-67.8)
Proteus spp.	Third-generation cephalosporins	23 (14-35)	3.1 (1.9-4.7)	2060 (1270-3110)	278.3 (171.9-420.1)	5 (2-11)	0.7 (0.2-1.5)	478 (152-964)	64.6 (20.5-130.3)
Proteus spp.	Trimethoprim-Sulfamethoxazole	53 (35-78)	7.2 (4.8-10.6)	4750 (3150-6960)	642.6 (425.5-941.4)	2 (0-5)	0.3 (0.0-0.6)	184 (0-406)	24.8 (0.0-54.9)
Proteus spp.	Resistance to one or more antibiotics	106 (70-156)	14.3 (9.5-21.1)	9440 (6270-13900)	1276.1 (847.9-1874.2)	13 (7-23)	1.8 (0.9-3.1)	1160 (594-2020)	157.2 (80.3-273.3)
Pseudomonas aeruginosa	Aminoglycosides	514 (341-727)	69.5 (46.1-98.2)	45800 (30300-64700)	6185.1 (4102.7-8746.3)	13 (0-32)	1.8 (0.0-4.4)	1170 (0-2890)	158.8 (0.0-390.7)
Pseudomonas aeruginosa	Anti-pseudomonal penicillin/Beta-Lactamase inhibitors	833 (558-1190)	112.6 (75.5-161.2)	74100 (49700-106000)	10015.6 (6712.8-14346.0)	45 (27-72)	6.1 (3.6-9.7)	4030 (2380-6370)	544.2 (322.2-861.2)
Pseudomonas aeruginosa	Carbapenem	1210 (811-1710)	163.7 (109.6-230.9)	108000 (72100-152000)	14566.5 (9753.3-20549.4)	218 (139-333)	29.4 (18.8-45.0)	19400 (12400-29600)	2617.7 (1670.4-4000.4)
Pseudomonas aeruginosa	Fluoroquinolones	914 (611-1300)	123.6 (82.6-176.0)	81400 (54400-116000)	10999.0 (7349.7-15659.3)	96 (59-146)	13.0 (8.0-19.8)	8560 (5300-13000)	1157.6 (716.3-1761.5)
Pseudomonas aeruginosa	Fourth-generation cephalosporins	726 (483-1030)	98.1 (65.3-139.4)	64600 (43000-91700)	8727.4 (5810.1-12401.5)	11 (6-17)	1.4 (0.8-2.3)	935 (520-1530)	126.3 (70.3-206.8)
Pseudomonas aeruginosa	Third-generation cephalosporins	810 (534-1160)	109.4 (72.2-156.5)	72000 (47500-103000)	9737.3 (6426.4-13924.4)	23 (9-43)	3.1 (1.3-5.8)	2050 (838-3820)	277.7 (113.3-516.2)
Pseudomonas aeruginosa	Resistance to one or more antibiotics	1660 (1100-2340)	224.0 (149.2-315.9)	147000 (98200-208000)	19929.4 (13271.2-28106.3)	406 (261-613)	54.9 (35.3-82.8)	36100 (23300-54500)	4882.3 (3146.1-7371.6)
Salmonella Paratyphi	Fluoroquinolones	--	--	0 (0-0)	0.0 (0.0-0.0)	--	--	0 (0-0)	0.0 (0.0-0.0)
Salmonella Paratyphi	Multi-drug resistance in Salmonella Typhi and Paratyphi	--	--	0 (0-0)	0.0 (0.0-0.0)	--	--	0 (0-0)	0.0 (0.0-0.0)
Salmonella Paratyphi	Resistance to one or more antibiotics	--	--	0 (0-0)	0.0 (0.0-0.0)	--	--	0 (0-0)	0.0 (0.0-0.0)
Salmonella Typhi	Fluoroquinolones	191 (89-356)	25.8 (12.1-48.2)	17000 (7940-31700)	2296.7 (1073.0-4283.3)	39 (5-90)	5.3 (0.6-12.1)	3490 (409-7960)	471.7 (55.3-1075.9)
Salmonella Typhi	Multi-drug resistance in Salmonella Typhi and Paratyphi	36 (17-69)	4.9 (2.3-9.3)	3230 (1490-6100)	436.2 (201.0-824.5)	5 (0-14)	0.6 (0.0-1.8)	406 (0-1210)	54.9 (0.0-163.6)
Salmonella Typhi	Resistance to one or more antibiotics	222 (110-407)	30.0 (14.9-55.0)	19700 (9790-36200)	2664.3 (1323.0-4893.5)	44 (8-97)	5.9 (1.1-13.1)	3900 (715-8650)	526.6 (96.7-1168.7)
Non-typhoidal *Salmonella*	Fluoroquinolones	17 (8-33)	2.3 (1.1-4.4)	1550 (752-2920)	209.9 (101.7-395.0)	4 (0-9)	0.5 (0.1-1.2)	323 (44-818)	43.7 (6.0-110.6)
Non-typhoidal *Salmonella*	Resistance to one or more antibiotics	17 (8-33)	2.3 (1.1-4.4)	1550 (752-2920)	209.9 (101.7-395.0)	4 (0-9)	0.5 (0.1-1.2)	323 (44-818)	43.7 (6.0-110.6)
Serratia spp.	Aminoglycosides	82 (51-124)	11.1 (6.9-16.8)	7300 (4560-11100)	987.1 (617.1-1493.8)	5 (0-13)	0.6 (0.0-1.7)	427 (0-1120)	57.7 (0.0-151.1)
Serratia spp.	Anti-pseudomonal penicillin/Beta-Lactamase inhibitors	75 (47-114)	10.1 (6.4-15.4)	6670 (4190-10200)	902.0 (565.8-1372.5)	14 (7-24)	1.9 (0.9-3.2)	1240 (615-2130)	167.2 (83.2-288.3)
Serratia spp.	Carbapenem	88 (56-131)	11.9 (7.5-17.7)	7830 (4960-11700)	1059.0 (670.7-1575.8)	24 (13-40)	3.2 (1.7-5.4)	2120 (1110-3560)	286.4 (150.4-481.7)
Serratia spp.	Fluoroquinolones	56 (35-87)	7.6 (4.7-11.8)	5020 (3110-7770)	679.3 (420.3-1049.9)	6 (1-13)	0.8 (0.2-1.7)	524 (113-1120)	70.9 (15.3-150.9)
Serratia spp.	Fourth-generation cephalosporins	133 (82-201)	18.0 (11.1-27.2)	11800 (7320-17900)	1598.8 (989.8-2417.7)	12 (5-23)	1.6 (0.7-3.1)	1080 (448-2050)	145.8 (60.6-277.3)

Pathogen	Antibiotic Class	Post Neonatal							
		Associated with resistance				Attributable to resistance			
		Deaths		DALYs		Deaths		DALYs	
		Counts	Rate per 100 000	Counts	Rate per 100 000	Counts	Rate per 100 000	Counts	Rate per 100 000
All pathogens	Resistance to one or more antibiotics	11500 (8210-15600)	129.6 (92.6-176.4)	1020000 (727000-1390000)	11482.5 (8201.9-15631.6)	2700 (1880-3690)	30.5 (21.2-41.6)	239000 (166000-327000)	2697.8 (1874.4-3685.1)
Acinetobacter baumannii	Aminoglycosides	342 (201-528)	3.9 (2.3-6.0)	30300 (17800-46700)	341.6 (200.7-526.8)	14 (0-35)	0.2 (0.0-0.4)	1260 (1-3130)	14.2 (0.0-35.3)
Acinetobacter baumannii	Anti-pseudomonal penicillin/Beta-Lactamase inhibitors	432 (254-660)	4.9 (2.9-7.4)	38200 (22400-58400)	431.2 (253.1-658.5)	8 (4-14)	0.1 (0.0-0.2)	701 (317-1280)	7.9 (3.6-14.4)
Acinetobacter baumannii	Beta Lactam/Beta-lactamase inhibitors	398 (236-608)	4.5 (2.7-6.9)	35200 (20900-53800)	397.3 (235.5-606.8)	1 (0-2)	0.0 (0.0-0.0)	76 (17-170)	0.9 (0.2-1.9)
Acinetobacter baumannii	Carbapenem	412 (242-628)	4.6 (2.7-7.1)	36400 (21400-55500)	410.5 (241.5-626.3)	68 (35-117)	0.8 (0.4-1.3)	6040 (3100-10400)	68.1 (35.0-116.7)
Acinetobacter baumannii	Fluoroquinolones	424 (252-643)	4.8 (2.8-7.3)	37500 (22200-56900)	422.8 (250.9-641.8)	53 (26-91)	0.6 (0.3-1.0)	4680 (2320-8040)	52.7 (26.2-90.7)
Acinetobacter baumannii	Fourth-generation cephalosporins	440 (259-671)	5.0 (2.9-7.6)	38900 (22900-59300)	438.9 (258.4-668.9)	2 (1-5)	0.0 (0.0-0.1)	186 (62-417)	2.1 (0.7-4.7)
Acinetobacter baumannii	Third-generation cephalosporins	447 (266-683)	5.0 (3.0-7.7)	39500 (23600-60400)	445.4 (265.7-681.6)	6 (2-11)	0.1 (0.0-0.1)	509 (197-945)	5.7 (2.2-10.7)
Acinetobacter baumannii	Resistance to one or more antibiotics	479 (284-731)	5.4 (3.2-8.2)	42300 (25100-64700)	477.5 (283.2-729.5)	152 (87-241)	1.7 (1.0-2.7)	13400 (7700-21300)	151.6 (86.9-240.6)
Citrobacter spp.	Aminoglycosides	12 (6-20)	0.1 (0.1-0.2)	1030 (553-1790)	11.6 (6.2-20.2)	1 (0-2)	0.0 (0.0-0.0)	64 (0-176)	0.7 (0.0-2.0)
Citrobacter spp.	Anti-pseudomonal penicillin/Beta-Lactamase inhibitors	20 (11-33)	0.2 (0.1-0.4)	1730 (931-2950)	19.5 (10.5-33.3)	4 (2-8)	0.0 (0.0-0.1)	378 (161-721)	4.3 (1.8-8.1)
Citrobacter spp.	Carbapenem	10 (5-18)	0.1 (0.1-0.2)	916 (475-1570)	10.3 (5.4-17.7)	3 (1-5)	0.0 (0.0-0.1)	233 (102-463)	2.6 (1.1-5.2)
Citrobacter spp.	Fluoroquinolones	24 (13-42)	0.3 (0.1-0.5)	2150 (1160-3680)	24.3 (13.0-41.5)	4 (2-8)	0.0 (0.0-0.1)	342 (138-708)	3.9 (1.6-8.0)
Citrobacter spp.	Fourth-generation cephalosporins	23 (12-40)	0.3 (0.1-0.5)	2080 (1060-3580)	23.4 (11.9-40.4)	2 (1-5)	0.0 (0.0-0.1)	212 (79-430)	2.4 (0.9-4.9)
Citrobacter spp.	Third-generation cephalosporins	57 (30-98)	0.6 (0.3-1.1)	5020 (2670-8630)	56.6 (30.1-97.4)	7 (3-15)	0.1 (0.0-0.2)	653 (247-1330)	7.4 (2.8-15.0)
Citrobacter spp.	Resistance to one or more antibiotics	75 (41-128)	0.8 (0.5-1.4)	6620 (3590-11300)	74.6 (40.5-128.0)	21 (11-39)	0.2 (0.1-0.4)	1880 (935-3490)	21.2 (10.5-39.4)
Enterobacter spp.	Aminoglycosides	54 (35-80)	0.6 (0.4-0.9)	4760 (3060-7090)	53.7 (34.5-80.0)	4 (1-7)	0.0 (0.0-0.1)	311 (85-615)	3.5 (1.0-6.9)
Enterobacter spp.	Anti-pseudomonal penicillin/Beta-Lactamase inhibitors	120 (76-178)	1.4 (0.9-2.0)	10600 (6750-15800)	120.1 (76.2-177.9)	13 (7-22)	0.2 (0.1-0.2)	1190 (628-1950)	13.5 (7.1-22.0)
Enterobacter spp.	Carbapenem	63 (40-94)	0.7 (0.4-1.1)	5570 (3500-8340)	62.9 (39.5-94.0)	15 (9-25)	0.2 (0.1-0.3)	1370 (756-2220)	15.5 (8.5-25.0)
Enterobacter spp.	Fluoroquinolones	80 (51-120)	0.9 (0.6-1.3)	7070 (4540-10600)	79.8 (51.2-119.4)	9 (5-16)	0.1 (0.1-0.2)	806 (419-1390)	9.1 (4.7-15.7)
Enterobacter spp.	Fourth-generation cephalosporins	157 (103-234)	1.8 (1.2-2.6)	13900 (9170-20800)	157.1 (103.4-234.2)	10 (5-18)	0.1 (0.1-0.2)	893 (420-1620)	10.1 (4.7-18.3)
Enterobacter spp.	Trimethoprim-Sulfamethoxazole	159 (101-237)	1.8 (1.1-2.7)	14000 (8970-21000)	158.4 (101.1-236.6)	10 (0-21)	0.1 (0.0-0.2)	864 (0-1840)	9.7 (0.0-20.7)
Enterobacter spp.	Resistance to one or more antibiotics	271 (177-399)	3.1 (2.0-4.5)	24000 (15700-35400)	271.0 (177.1-398.8)	61 (39-92)	0.7 (0.4-1.0)	5440 (3460-8190)	61.3 (39.0-92.3)
Enterococcus faecalis	Fluoroquinolones	80 (50-122)	0.9 (0.6-1.4)	7100 (4400-10900)	80.1 (49.7-122.7)	19 (9-35)	0.2 (0.1-0.4)	1710 (777-3130)	19.3 (8.8-35.3)
Enterococcus faecalis	Vancomycin	22 (14-36)	0.3 (0.2-0.4)	1970 (1220-3150)	22.2 (13.7-35.5)	7 (3-14)	0.1 (0.0-0.2)	652 (300-1240)	7.4 (3.4-14.0)
Enterococcus faecalis	Resistance to one or more antibiotics	92 (57-143)	1.0 (0.6-1.6)	8150 (5070-12700)	91.9 (57.1-143.6)	27 (14-46)	0.3 (0.2-0.5)	2360 (1260-4060)	26.6 (14.2-45.8)
Enterococcus faecium	Fluoroquinolones	191 (109-306)	2.2 (1.2-3.5)	16900 (9660-27100)	190.6 (109.0-306.1)	30 (9-60)	0.3 (0.1-0.7)	2620 (775-5270)	29.5 (8.7-59.4)
Enterococcus faecium	Vancomycin	134 (80-213)	1.5 (0.9-2.4)	11800 (7090-18900)	133.5 (79.9-213.0)	34 (18-60)	0.4 (0.2-0.7)	3020 (1580-5300)	34.0 (17.9-59.8)
Enterococcus faecium	Resistance to one or more antibiotics	205 (119-325)	2.3 (1.3-3.7)	18100 (10500-28800)	204.7 (118.8-324.6)	64 (36-107)	0.7 (0.4-1.2)	5630 (3160-9440)	63.5 (35.7-106.4)
Other enterococci	Fluoroquinolones	150 (84-252)	1.7 (0.9-2.8)	13300 (7460-22300)	149.9 (84.1-251.4)	27 (4-56)	0.3 (0.0-0.6)	2420 (332-4940)	27.3 (3.7-55.7)
Other enterococci	Vancomycin	34 (18-58)	0.4 (0.2-0.7)	2980 (1640-5170)	33.6 (18.5-58.3)	6 (2-11)	0.1 (0.0-0.1)	497 (219-1010)	5.6 (2.5-11.4)
Other enterococci	Resistance to one or more antibiotics	150 (84-253)	1.7 (0.9-2.8)	13300 (7460-22400)	150.2 (84.1-252.1)	33 (10-62)	0.4 (0.1-0.7)	2910 (923-5500)	32.9 (10.4-62.1)
Escherichia coli	Aminoglycosides	180 (126-248)	2.0 (1.4-2.8)	16100 (11200-22100)	181.0 (126.3-248.9)	11 (7-16)	0.1 (0.1-0.2)	979 (638-1410)	11.0 (7.2-15.9)
Escherichia coli	Aminopenicillin	1030 (732-1390)	11.6 (8.3-15.7)	91500 (65100-124000)	1031.9 (734.0-1395.8)	18 (11-25)	0.2 (0.1-0.3)	1580 (1020-2250)	17.9 (11.5-25.4)
Escherichia coli	Beta Lactam/Beta-lactamase inhibitors	781 (551-1070)	8.8 (6.2-12.0)	69500 (49000-94800)	784.3 (552.2-1069.6)	39 (27-54)	0.4 (0.3-0.6)	3450 (2390-4830)	39.0 (27.0-54.5)
Escherichia coli	Carbapenem	60 (39-88)	0.7 (0.4-1.0)	5370 (3500-7810)	60.6 (39.5-88.0)	13 (7-22)	0.2 (0.1-0.2)	1190 (650-1970)	13.4 (7.3-22.2)
Escherichia coli	Fluoroquinolones	628 (433-866)	7.1 (4.9-9.8)	55900 (38500-77100)	630.3 (434.3-869.1)	66 (44-94)	0.7 (0.5-1.1)	5860 (3930-8380)	66.0 (44.3-94.6)
Escherichia coli	Third-generation cephalosporins	524 (356-733)	5.9 (4.0-8.3)	46600 (31600-65400)	526.0 (356.7-737.8)	72 (32-133)	0.8 (0.4-1.5)	6410 (2840-11800)	72.2 (32.0-133.0)
Escherichia coli	Trimethoprim-Sulfamethoxazole	791 (561-1080)	8.9 (6.3-12.2)	70400 (50000-95800)	794.0 (563.5-1080.5)	49 (32-68)	0.5 (0.4-0.8)	4310 (2860-6070)	48.6 (32.3-68.5)
Escherichia coli	Resistance to one or more antibiotics	1110 (792-1510)	12.5 (8.9-17.0)	99000 (70500-134000)	1116.9 (794.8-1512.7)	267 (181-380)	3.0 (2.0-4.3)	23800 (16100-33800)	268.1 (181.2-381.5)
Group A *Streptococcus*	Macrolide	100 (63-151)	1.1 (0.7-1.7)	8890 (5610-13400)	100.2 (63.3-151.2)	9 (0-31)	0.1 (0.0-0.4)	843 (0-2770)	9.5 (0.0-31.2)
Group A *Streptococcus*	Resistance to one or more antibiotics	100 (63-151)	1.1 (0.7-1.7)	8890 (5610-13400)	100.2 (63.3-151.2)	9 (0-31)	0.1 (0.0-0.4)	843 (0-2770)	9.5 (0.0-31.2)
Group B *Streptococcus*	Fluoroquinolones	65 (43-92)	0.7 (0.5-1.0)	5730 (3770-8180)	64.6 (42.6-92.3)	11 (2-25)	0.1 (0.0-0.3)	990 (151-2210)	11.2 (1.7-24.9)
Group B Streptococcus	Macrolide	224 (150-314)	2.5 (1.7-3.5)	19800 (13300-27800)	223.8 (149.7-314.0)	23 (0-62)	0.3 (0.0-0.7)	2020 (0-5480)	22.8 (0.0-61.8)
Group B Streptococcus	Penicillin	7 (5-11)	0.1 (0.1-0.1)	655 (433-933)	7.4 (4.9-10.5)	2 (0-3)	0.0 (0.0-0.0)	140 (31-298)	1.6 (0.3-3.4)
Group B Streptococcus	Resistance to one or more antibiotics	258 (177-357)	2.9 (2.0-4.0)	22900 (15600-31600)	257.7 (176.3-356.5)	36 (2-77)	0.4 (0.0-0.9)	3150 (209-6830)	35.5 (2.4-77.0)
Haemophilus influenzae	Aminopenicillin	140 (99-196)	1.6 (1.1-2.2)	12400 (8740-17400)	140.3 (98.6-195.8)	23 (2-47)	0.3 (0.0-0.5)	2010 (157-4190)	22.6 (1.8-47.3)
Haemophilus influenzae	Third-generation cephalosporins	39 (27-55)	0.4 (0.3-0.6)	3430 (2370-4850)	38.6 (26.8-54.7)	11 (5-21)	0.1 (0.1-0.2)	997 (458-1810)	11.2 (5.2-20.5)
Haemophilus influenzae	Resistance to one or more antibiotics	160 (112-219)	1.8 (1.3-2.5)	14200 (9910-19400)	159.6 (111.7-218.7)	34 (12-63)	0.4 (0.1-0.7)	3000 (1030-5580)	33.9 (11.6-63.0)
Klebsiella pneumoniae	Aminoglycosides	862 (601-1200)	9.7 (6.8-13.5)	76300 (53200-106000)	860.4 (599.6-1194.7)	67 (41-98)	0.8 (0.5-1.1)	5910 (3610-8710)	66.7 (40.7-98.2)
Klebsiella pneumoniae	Beta Lactam/Beta-lactamase inhibitors	1640 (1160-2240)	18.5 (13.1-25.3)	145000 (103000-199000)	1632.6 (1160.3-2239.3)	42 (22-67)	0.5 (0.3-0.8)	3700 (1970-5930)	41.8 (22.3-66.9)
Klebsiella pneumoniae	Carbapenem	492 (325-712)	5.5 (3.7-8.0)	43500 (28800-63000)	490.7 (324.5-710.4)	113 (69-174)	1.3 (0.8-2.0)	10000 (6140-15400)	113.2 (69.3-173.4)
Klebsiella pneumoniae	Fluoroquinolones	1120 (792-1520)	12.6 (8.9-17.2)	99100 (70100-135000)	1117.6 (790.5-1521.5)	79 (46-121)	0.9 (0.5-1.4)	7000 (4120-10700)	79.0 (46.5-121.0)
Klebsiella pneumoniae	Third-generation cephalosporins	1360 (908-1920)	15.3 (10.2-21.7)	120000 (80300-170000)	1356.8 (905.6-1917.0)	154 (55-294)	1.7 (0.6-3.3)	13600 (4910-26000)	153.3 (55.4-293.6)
Klebsiella pneumoniae	Trimethoprim-Sulfamethoxazole	1320 (934-1840)	14.9 (10.5-20.8)	117000 (82600-163000)	1318.8 (931.3-1836.0)	65 (33-109)	0.7 (0.4-1.2)	5780 (2890-9630)	65.2 (32.5-108.6)
Klebsiella pneumoniae	Resistance to one or more antibiotics	1860 (1310-2570)	21.0 (14.8-29.0)	165000 (116000-227000)	1859.9 (1305.8-2562.3)	520 (337-765)	5.9 (3.8-8.6)	46000 (29800-67700)	519.1 (336.0-763.3)
Morganella spp.	Fluoroquinolones	1 (1-2)	0.0 (0.0-0.0)	93 (51-161)	1.0 (0.6-1.8)	0 (0-0)	0.0 (0.0-0.0)	16 (6-34)	0.2 (0.1-0.4)
Morganella spp.	Fourth-generation cephalosporins	0 (0-1)	0.0 (0.0-0.0)	34 (19-61)	0.4 (0.2-0.7)	0 (0-0)	0.0 (0.0-0.0)	6 (2-14)	0.1 (0.0-0.2)
Morganella spp.	Third-generation cephalosporins	0 (0-1)	0.0 (0.0-0.0)	38 (21-65)	0.4 (0.2-0.7)	0 (0-0)	0.0 (0.0-0.0)	5 (2-10)	0.1 (0.0-0.1)
Morganella spp.	Resistance to one or more antibiotics	1 (1-2)	0.0 (0.0-0.0)	117 (64-198)	1.3 (0.7-2.2)	0 (0-1)	0.0 (0.0-0.0)	27 (13-50)	0.3 (0.1-0.6)
Mycobacterium tuberculosis	Extensive drug resistance in TB	2 (1-5)	0.0 (0.0-0.1)	197 (73-421)	2.2 (0.8-4.8)	1 (1-3)	0.0 (0.0-0.0)	120 (45-259)	1.4 (0.5-2.9)
Mycobacterium tuberculosis	Isoniazid mono-resistance	7 (4-11)	0.1 (0.0-0.1)	615 (365-939)	6.9 (4.1-10.6)	1 (0-4)	0.0 (0.0-0.0)	97 (0-341)	1.1 (0.0-3.8)
Mycobacterium tuberculosis	Multi-drug resistance excluding extensive drug resistance in TB	13 (5-30)	0.2 (0.1-0.3)	1190 (442-2620)	13.4 (5.0-29.5)	8 (1-22)	0.1 (0.0-0.3)	703 (67-1980)	7.9 (0.8-22.3)
Mycobacterium tuberculosis	Rifampicin mono-resistance	2 (1-2)	0.0 (0.0-0.0)	141 (88-210)	1.6 (1.0-2.4)	0 (0-1)	0.0 (0.0-0.0)	39 (7-84)	0.4 (0.1-0.9)
Mycobacterium tuberculosis	Resistance to one or more antibiotics	24 (14-43)	0.3 (0.2-0.5)	2140 (1210-3830)	24.2 (13.6-43.2)	11 (2-27)	0.1 (0.0-0.3)	960 (216-2410)	10.8 (2.4-27.1)
Neisseria gonorrhoeae	Fluoroquinolones	--	--	--	--	--	--	--	--
Neisseria gonorrhoeae	Third-generation cephalosporins	--	--	--	--	--	--	--	--
Neisseria gonorrhoeae	Resistance to one or more antibiotics	--	--	--	--	--	--	--	--
Proteus spp.	Aminoglycosides	4 (3-7)	0.1 (0.0-0.1)	394 (253-585)	4.4 (2.9-6.6)	0 (0-1)	0.0 (0.0-0.0)	23 (2-50)	0.3 (0.0-0.6)
Proteus spp.	Aminopenicillin	67 (44-99)	0.8 (0.5-1.1)	5970 (3880-8830)	67.3 (43.8-99.6)	2 (0-5)	0.0 (0.0-0.1)	141 (0-423)	1.6 (0.0-4.8)
Proteus spp.	Fluoroquinolones	27 (17-39)	0.3 (0.2-0.4)	2350 (1520-3440)	26.6 (17.1-38.8)	2 (1-4)	0.0 (0.0-0.0)	175 (67-360)	2.0 (0.8-4.1)
Proteus spp.	Third-generation cephalosporins	16 (9-24)	0.2 (0.1-0.3)	1390 (839-2170)	15.6 (9.5-24.4)	4 (1-7)	0.0 (0.0-0.1)	321 (97-631)	3.6 (1.1-7.1)
Proteus spp.	Trimethoprim-Sulfamethoxazole	34 (22-52)	0.4 (0.2-0.6)	3040 (1940-4570)	34.2 (21.9-51.5)	1 (0-3)	0.0 (0.0-0.0)	117 (0-253)	1.3 (0.0-2.8)
Proteus spp.	Resistance to one or more antibiotics	71 (46-105)	0.8 (0.5-1.2)	6320 (4050-9360)	71.3 (45.7-105.6)	9 (4-15)	0.1 (0.0-0.2)	776 (375-1350)	8.8 (4.2-15.2)
Pseudomonas aeruginosa	Aminoglycosides	219 (151-301)	2.5 (1.7-3.4)	19400 (13400-26700)	218.7 (150.8-301.0)	5 (0-14)	0.1 (0.0-0.2)	487 (0-1220)	5.5 (0.0-13.8)
Pseudomonas aeruginosa	Anti-pseudomonal penicillin/Beta-Lactamase inhibitors	382 (266-532)	4.3 (3.0-6.0)	33800 (23600-47100)	381.2 (265.6-531.3)	20 (12-32)	0.2 (0.1-0.4)	1750 (1040-2830)	19.7 (11.7-32.0)
Pseudomonas aeruginosa	Carbapenem	568 (394-799)	6.4 (4.4-9.0)	50200 (34900-70700)	566.6 (393.7-797.5)	100 (66-150)	1.1 (0.7-1.7)	8890 (5850-13300)	100.2 (66.0-149.9)
Pseudomonas aeruginosa	Fluoroquinolones	451 (311-627)	5.1 (3.5-7.1)	39900 (27500-55500)	450.2 (310.4-626.5)	47 (31-70)	0.5 (0.3-0.8)	4210 (2740-6200)	47.5 (30.9-70.0)
Pseudomonas aeruginosa	Fourth-generation cephalosporins	330 (227-462)	3.7 (2.6-5.2)	29200 (20100-40900)	329.8 (226.5-461.3)	5 (3-8)	0.1 (0.0-0.1)	414 (234-687)	4.7 (2.6-7.7)
Pseudomonas aeruginosa	Third-generation cephalosporins	360 (246-512)	4.1 (2.8-5.8)	31900 (21800-45300)	359.3 (245.9-510.7)	10 (4-19)	0.1 (0.0-0.2)	868 (358-1640)	9.8 (4.0-18.5)
Pseudomonas aeruginosa	Resistance to one or more antibiotics	770 (533-1080)	8.7 (6.0-12.1)	68100 (47200-95300)	768.5 (532.2-1074.7)	188 (123-283)	2.1 (1.4-3.2)	16600 (10900-25000)	187.4 (122.8-282.4)
Salmonella Paratyphi	Fluoroquinolones	0 (0-0)	0.0 (0.0-0.0)	6 (3-15)	0.1 (0.0-0.2)	0 (0-0)	0.0 (0.0-0.0)	1 (0-4)	0.0 (0.0-0.0)
Salmonella Paratyphi	Multi-drug resistance in Salmonella Typhi and Paratyphi	0 (0-0)	0.0 (0.0-0.0)	0 (0-0)	0.0 (0.0-0.0)	0 (0-0)	0.0 (0.0-0.0)	0 (0-0)	0.0 (0.0-0.0)
Salmonella Paratyphi	Resistance to one or more antibiotics	0 (0-0)	0.0 (0.0-0.0)	6 (3-15)	0.1 (0.0-0.2)	0 (0-0)	0.0 (0.0-0.0)	1 (0-4)	0.0 (0.0-0.0)
Salmonella Typhi	Fluoroquinolones	74 (32-141)	0.8 (0.4-1.6)	6560 (2860-12500)	74.0 (32.2-140.7)	15 (2-37)	0.2 (0.0-0.4)	1340 (162-3280)	15.1 (1.8-37.0)
Salmonella Typhi	Multi-drug resistance in Salmonella Typhi and Paratyphi	13 (5-27)	0.1 (0.1-0.3)	1140 (484-2350)	12.8 (5.5-26.5)	2 (0-5)	0.0 (0.0-0.1)	141 (0-410)	1.6 (0.0-4.6)
Salmonella Typhi	Resistance to one or more antibiotics	85 (38-155)	1.0 (0.4-1.7)	7510 (3370-13700)	84.8 (38.0-154.2)	17 (3-39)	0.2 (0.0-0.4)	1480 (305-3430)	16.7 (3.4-38.7)
Non-typhoidal *Salmonella*	Fluoroquinolones	19 (7-43)	0.2 (0.1-0.5)	1850 (685-4250)	20.9 (7.7-47.9)	4 (0-10)	0.0 (0.0-0.1)	365 (50-923)	4.1 (0.6-10.4)
Non-typhoidal *Salmonella*	Resistance to one or more antibiotics	19 (7-43)	0.2 (0.1-0.5)	1850 (685-4250)	20.9 (7.7-47.9)	4 (0-10)	0.0 (0.0-0.1)	365 (50-923)	4.1 (0.6-10.4)
Serratia spp.	Aminoglycosides	22 (13-35)	0.2 (0.1-0.4)	1940 (1150-3120)	21.9 (13.0-35.2)	1 (0-4)	0.0 (0.0-0.0)	114 (0-317)	1.3 (0.0-3.6)
Serratia spp.	Anti-pseudomonal penicillin/Beta-Lactamase inhibitors	22 (13-35)	0.2 (0.1-0.4)	1950 (1160-3110)	22.0 (13.1-35.1)	4 (2-7)	0.0 (0.0-0.1)	353 (170-654)	4.0 (1.9-7.4)
Serratia spp.	Carbapenem	27 (16-43)	0.3 (0.2-0.5)	2390 (1410-3820)	27.0 (15.9-43.1)	7 (4-13)	0.1 (0.0-0.1)	649 (327-1150)	7.3 (3.7-13.0)
Serratia spp.	Fluoroquinolones	15 (9-25)	0.2 (0.1-0.3)	1350 (785-2210)	15.2 (8.9-24.9)	2 (0-4)	0.0 (0.0-0.0)	140 (31-311)	1.6 (0.3-3.5)
Serratia spp.	Fourth-generation cephalosporins	36 (22-59)	0.4 (0.2-0.7)	3230 (1920-5180)	36.4 (21.6-58.4)	3 (1-6)	0.0 (0.0-0.1)	276 (111-538)	3.1 (1.2-6.1)

Pathogen	Antibiotic Class	1 to 4							
		Associated with resistance				Attributable to resistance			
		Deaths		DALYs		Deaths		DALYs	
		Counts	Rate per 100 000	Counts	Rate per 100 000	Counts	Rate per 100 000	Counts	Rate per 100 000
All pathogens	Resistance to one or more antibiotics	4220 (3060-5780)	11.0 (7.9-15.0)	369000 (267000-504000)	958.4 (693.5-1308.9)	990 (700-1350)	2.6 (1.8-3.5)	86100 (61000-117000)	223.7 (158.5-305.1)
Acinetobacter baumannii	Aminoglycosides	92 (49-155)	0.2 (0.1-0.4)	7920 (4210-13400)	20.6 (10.9-34.7)	4 (0-10)	0.0 (0.0-0.0)	336 (2-894)	0.9 (0.0-2.3)
Acinetobacter baumannii	Anti-pseudomonal penicillin/Beta-Lactamase inhibitors	120 (65-201)	0.3 (0.2-0.5)	10400 (5590-17300)	27.0 (14.5-45.1)	4 (2-7)	0.0 (0.0-0.0)	303 (137-579)	0.8 (0.4-1.5)
Acinetobacter baumannii	Beta Lactam/Beta-lactamase inhibitors	108 (58-182)	0.3 (0.2-0.5)	9340 (5020-15700)	24.3 (13.1-40.7)	0 (0-1)	0.0 (0.0-0.0)	18 (4-49)	0.0 (0.0-0.1)
Acinetobacter baumannii	Carbapenem	108 (57-180)	0.3 (0.1-0.5)	9300 (4940-15500)	24.2 (12.8-40.4)	18 (8-33)	0.0 (0.0-0.1)	1550 (725-2860)	4.0 (1.9-7.4)
Acinetobacter baumannii	Fluoroquinolones	113 (61-188)	0.3 (0.2-0.5)	9770 (5240-16200)	25.4 (13.6-42.2)	14 (6-25)	0.0 (0.0-0.1)	1220 (541-2180)	3.2 (1.4-5.7)
Acinetobacter baumannii	Fourth-generation cephalosporins	122 (66-206)	0.3 (0.2-0.5)	10500 (5710-17800)	27.3 (14.9-46.2)	1 (0-2)	0.0 (0.0-0.0)	58 (16-140)	0.2 (0.0-0.4)
Acinetobacter baumannii	Third-generation cephalosporins	122 (66-206)	0.3 (0.2-0.5)	10600 (5670-17700)	27.4 (14.7-46.0)	2 (1-3)	0.0 (0.0-0.0)	130 (45-271)	0.3 (0.1-0.7)
Acinetobacter baumannii	Resistance to one or more antibiotics	133 (72-222)	0.3 (0.2-0.6)	11400 (6180-19100)	29.8 (16.1-49.8)	42 (22-71)	0.1 (0.1-0.2)	3620 (1880-6100)	9.4 (4.9-15.9)
Citrobacter spp.	Aminoglycosides	4 (2-7)	0.0 (0.0-0.0)	329 (153-606)	0.9 (0.4-1.6)	0 (0-1)	0.0 (0.0-0.0)	20 (0-55)	0.1 (0.0-0.1)
Citrobacter spp.	Anti-pseudomonal penicillin/Beta-Lactamase inhibitors	7 (3-12)	0.0 (0.0-0.0)	573 (280-1060)	1.5 (0.7-2.8)	1 (1-3)	0.0 (0.0-0.0)	119 (47-240)	0.3 (0.1-0.6)
Citrobacter spp.	Carbapenem	3 (1-5)	0.0 (0.0-0.0)	259 (123-470)	0.7 (0.3-1.2)	1 (0-2)	0.0 (0.0-0.0)	65 (26-134)	0.2 (0.1-0.3)
Citrobacter spp.	Fluoroquinolones	7 (4-14)	0.0 (0.0-0.0)	633 (309-1180)	1.6 (0.8-3.1)	1 (0-2)	0.0 (0.0-0.0)	98 (36-207)	0.3 (0.1-0.5)
Citrobacter spp.	Fourth-generation cephalosporins	8 (4-15)	0.0 (0.0-0.0)	703 (327-1280)	1.8 (0.8-3.3)	1 (0-2)	0.0 (0.0-0.0)	70 (23-152)	0.2 (0.1-0.4)
Citrobacter spp.	Third-generation cephalosporins	16 (8-29)	0.0 (0.0-0.1)	1370 (652-2490)	3.6 (1.7-6.5)	2 (1-4)	0.0 (0.0-0.0)	152 (52-320)	0.4 (0.1-0.8)
Citrobacter spp.	Resistance to one or more antibiotics	21 (10-39)	0.1 (0.0-0.1)	1840 (879-3380)	4.8 (2.3-8.8)	6 (3-12)	0.0 (0.0-0.0)	524 (229-1010)	1.4 (0.6-2.6)
Enterobacter spp.	Aminoglycosides	19 (12-30)	0.0 (0.0-0.1)	1660 (1040-2560)	4.3 (2.7-6.7)	1 (0-2)	0.0 (0.0-0.0)	105 (30-207)	0.3 (0.1-0.5)
Enterobacter spp.	Anti-pseudomonal penicillin/Beta-Lactamase inhibitors	43 (27-64)	0.1 (0.1-0.2)	3700 (2340-5540)	9.6 (6.1-14.4)	5 (2-8)	0.0 (0.0-0.0)	404 (212-662)	1.1 (0.6-1.7)
Enterobacter spp.	Carbapenem	23 (15-35)	0.1 (0.0-0.1)	2030 (1260-3040)	5.3 (3.3-7.9)	6 (3-9)	0.0 (0.0-0.0)	493 (266-794)	1.3 (0.7-2.1)
Enterobacter spp.	Fluoroquinolones	36 (22-55)	0.1 (0.1-0.1)	3120 (1920-4770)	8.1 (5.0-12.4)	4 (2-7)	0.0 (0.0-0.0)	359 (178-616)	0.9 (0.5-1.6)
Enterobacter spp.	Fourth-generation cephalosporins	59 (38-89)	0.2 (0.1-0.2)	5110 (3260-7700)	13.3 (8.5-20.0)	4 (2-7)	0.0 (0.0-0.0)	335 (161-598)	0.9 (0.4-1.6)
Enterobacter spp.	Trimethoprim-Sulfamethoxazole	52 (33-81)	0.1 (0.1-0.2)	4530 (2890-7030)	11.8 (7.5-18.3)	3 (0-6)	0.0 (0.0-0.0)	264 (0-561)	0.7 (0.0-1.5)
Enterobacter spp.	Resistance to one or more antibiotics	97 (63-148)	0.3 (0.2-0.4)	8410 (5410-12800)	21.9 (14.1-33.3)	23 (14-35)	0.1 (0.0-0.1)	1960 (1210-3000)	5.1 (3.2-7.8)
Enterococcus faecalis	Fluoroquinolones	31 (18-51)	0.1 (0.0-0.1)	2740 (1590-4480)	7.1 (4.1-11.7)	8 (3-14)	0.0 (0.0-0.0)	657 (296-1230)	1.7 (0.8-3.2)
Enterococcus faecalis	Vancomycin	6 (4-11)	0.0 (0.0-0.0)	550 (329-945)	1.4 (0.9-2.5)	2 (1-4)	0.0 (0.0-0.0)	179 (81-356)	0.5 (0.2-0.9)
Enterococcus faecalis	Resistance to one or more antibiotics	34 (20-56)	0.1 (0.1-0.1)	3020 (1750-4930)	7.9 (4.5-12.8)	10 (5-17)	0.0 (0.0-0.0)	837 (421-1500)	2.2 (1.1-3.9)
Enterococcus faecium	Fluoroquinolones	66 (34-121)	0.2 (0.1-0.3)	5700 (2960-10400)	14.8 (7.7-27.1)	11 (3-25)	0.0 (0.0-0.1)	984 (271-2180)	2.6 (0.7-5.7)
Enterococcus faecium	Vancomycin	33 (17-59)	0.1 (0.0-0.2)	2860 (1490-5110)	7.4 (3.9-13.3)	8 (4-15)	0.0 (0.0-0.0)	712 (329-1320)	1.9 (0.9-3.4)
Enterococcus faecium	Resistance to one or more antibiotics	69 (36-126)	0.2 (0.1-0.3)	5940 (3090-10900)	15.4 (8.0-28.3)	20 (10-38)	0.1 (0.0-0.1)	1700 (826-3260)	4.4 (2.1-8.5)
Other enterococci	Fluoroquinolones	35 (18-63)	0.1 (0.0-0.2)	3140 (1660-5600)	8.2 (4.3-14.6)	6 (1-14)	0.0 (0.0-0.0)	560 (94-1200)	1.5 (0.2-3.1)
Other enterococci	Vancomycin	7 (4-14)	0.0 (0.0-0.0)	667 (350-1200)	1.7 (0.9-3.1)	1 (1-3)	0.0 (0.0-0.0)	115 (50-246)	0.3 (0.1-0.6)
Other enterococci	Resistance to one or more antibiotics	35 (18-63)	0.1 (0.0-0.2)	3140 (1660-5630)	8.2 (4.3-14.6)	8 (2-15)	0.0 (0.0-0.0)	676 (235-1360)	1.8 (0.6-3.5)
Escherichia coli	Aminoglycosides	90 (61-123)	0.2 (0.2-0.3)	7940 (5510-10800)	20.6 (14.3-28.2)	5 (3-8)	0.0 (0.0-0.0)	477 (310-696)	1.2 (0.8-1.8)
Escherichia coli	Aminopenicillin	441 (318-596)	1.1 (0.8-1.5)	39400 (28400-52900)	102.3 (73.8-137.6)	7 (4-9)	0.0 (0.0-0.0)	595 (391-837)	1.5 (1.0-2.2)
Escherichia coli	Beta Lactam/Beta-lactamase inhibitors	337 (242-457)	0.9 (0.6-1.2)	30200 (21600-41000)	78.4 (56.2-106.5)	15 (10-21)	0.0 (0.0-0.1)	1320 (910-1830)	3.4 (2.4-4.8)
Escherichia coli	Carbapenem	31 (20-47)	0.1 (0.1-0.1)	2770 (1790-4070)	7.2 (4.7-10.6)	7 (4-11)	0.0 (0.0-0.0)	597 (311-985)	1.6 (0.8-2.6)
Escherichia coli	Fluoroquinolones	284 (201-387)	0.7 (0.5-1.0)	25300 (18200-34800)	65.9 (47.2-90.5)	30 (20-42)	0.1 (0.1-0.1)	2630 (1830-3690)	6.8 (4.8-9.6)
Escherichia coli	Third-generation cephalosporins	251 (175-346)	0.7 (0.5-0.9)	22400 (15600-31100)	58.2 (40.7-80.8)	34 (15-62)	0.1 (0.0-0.2)	3050 (1370-5580)	7.9 (3.6-14.5)
Escherichia coli	Trimethoprim-Sulfamethoxazole	334 (241-453)	0.9 (0.6-1.2)	29800 (21500-40200)	77.5 (55.9-104.6)	20 (13-28)	0.1 (0.0-0.1)	1730 (1130-2440)	4.5 (2.9-6.3)
Escherichia coli	Resistance to one or more antibiotics	473 (341-642)	1.2 (0.9-1.7)	42200 (30400-57300)	109.8 (79.0-149.0)	117 (80-170)	0.3 (0.2-0.4)	10400 (7120-15000)	27.0 (18.5-38.9)
Group A *Streptococcus*	Macrolide	19 (10-32)	0.0 (0.0-0.1)	1810 (1020-2960)	4.7 (2.7-7.7)	2 (0-6)	0.0 (0.0-0.0)	171 (0-558)	0.4 (0.0-1.5)
Group A *Streptococcus*	Resistance to one or more antibiotics	19 (10-32)	0.0 (0.0-0.1)	1810 (1020-2960)	4.7 (2.7-7.7)	2 (0-6)	0.0 (0.0-0.0)	171 (0-558)	0.4 (0.0-1.5)
Group B *Streptococcus*	Fluoroquinolones	33 (22-47)	0.1 (0.1-0.1)	2850 (1880-4090)	7.4 (4.9-10.6)	6 (1-13)	0.0 (0.0-0.0)	498 (81-1110)	1.3 (0.2-2.9)
Group B Streptococcus	Macrolide	77 (51-108)	0.2 (0.1-0.3)	6760 (4470-9420)	17.6 (11.6-24.5)	8 (0-21)	0.0 (0.0-0.1)	672 (0-1810)	1.7 (0.0-4.7)
Group B Streptococcus	Penicillin	3 (2-4)	0.0 (0.0-0.0)	266 (170-385)	0.7 (0.4-1.0)	1 (0-1)	0.0 (0.0-0.0)	54 (11-117)	0.1 (0.0-0.3)
Group B Streptococcus	Resistance to one or more antibiotics	96 (65-133)	0.2 (0.2-0.3)	8360 (5690-11600)	21.7 (14.8-30.0)	14 (2-29)	0.0 (0.0-0.1)	1220 (186-2540)	3.2 (0.5-6.6)
Haemophilus influenzae	Aminopenicillin	65 (47-90)	0.2 (0.1-0.2)	5670 (4060-7790)	14.8 (10.5-20.2)	10 (1-21)	0.0 (0.0-0.1)	896 (72-1860)	2.3 (0.2-4.8)
Haemophilus influenzae	Third-generation cephalosporins	20 (14-28)	0.1 (0.0-0.1)	1760 (1220-2460)	4.6 (3.2-6.4)	6 (3-10)	0.0 (0.0-0.0)	510 (230-903)	1.3 (0.6-2.3)
Haemophilus influenzae	Resistance to one or more antibiotics	75 (55-102)	0.2 (0.1-0.3)	6560 (4740-8870)	17.1 (12.3-23.0)	16 (6-30)	0.0 (0.0-0.1)	1410 (510-2570)	3.7 (1.3-6.7)
Klebsiella pneumoniae	Aminoglycosides	289 (199-405)	0.8 (0.5-1.1)	25000 (17200-35000)	65.0 (44.7-90.9)	22 (13-33)	0.1 (0.0-0.1)	1910 (1140-2860)	5.0 (3.0-7.4)
Klebsiella pneumoniae	Beta Lactam/Beta-lactamase inhibitors	562 (393-783)	1.5 (1.0-2.0)	48600 (34000-67700)	126.3 (88.4-175.9)	14 (7-23)	0.0 (0.0-0.1)	1210 (620-2010)	3.1 (1.6-5.2)
Klebsiella pneumoniae	Carbapenem	125 (79-194)	0.3 (0.2-0.5)	10800 (6830-16800)	28.2 (17.8-43.6)	30 (18-48)	0.1 (0.0-0.1)	2560 (1520-4110)	6.6 (3.9-10.7)
Klebsiella pneumoniae	Fluoroquinolones	375 (260-528)	1.0 (0.7-1.4)	32400 (22500-45600)	84.3 (58.5-118.6)	26 (15-40)	0.1 (0.0-0.1)	2250 (1350-3450)	5.9 (3.5-9.0)
Klebsiella pneumoniae	Third-generation cephalosporins	475 (321-677)	1.2 (0.8-1.8)	41000 (27800-58400)	106.7 (72.3-151.8)	59 (22-113)	0.2 (0.1-0.3)	5070 (1860-9740)	13.2 (4.8-25.3)
Klebsiella pneumoniae	Trimethoprim-Sulfamethoxazole	470 (330-662)	1.2 (0.9-1.7)	40600 (28600-57100)	105.6 (74.3-148.5)	23 (12-40)	0.1 (0.0-0.1)	2010 (994-3420)	5.2 (2.6-8.9)
Klebsiella pneumoniae	Resistance to one or more antibiotics	636 (445-890)	1.7 (1.2-2.3)	55000 (38600-77000)	143.0 (100.3-200.1)	174 (110-256)	0.5 (0.3-0.7)	15000 (9520-22100)	39.0 (24.8-57.5)
Morganella spp.	Fluoroquinolones	1 (0-1)	0.0 (0.0-0.0)	51 (28-89)	0.1 (0.1-0.2)	0 (0-0)	0.0 (0.0-0.0)	9 (3-18)	0.0 (0.0-0.0)
Morganella spp.	Fourth-generation cephalosporins	0 (0-0)	0.0 (0.0-0.0)	15 (8-28)	0.0 (0.0-0.1)	0 (0-0)	0.0 (0.0-0.0)	3 (1-6)	0.0 (0.0-0.0)
Morganella spp.	Third-generation cephalosporins	0 (0-0)	0.0 (0.0-0.0)	16 (9-28)	0.0 (0.0-0.1)	0 (0-0)	0.0 (0.0-0.0)	2 (1-4)	0.0 (0.0-0.0)
Morganella spp.	Resistance to one or more antibiotics	1 (0-1)	0.0 (0.0-0.0)	61 (33-103)	0.2 (0.1-0.3)	0 (0-0)	0.0 (0.0-0.0)	13 (6-25)	0.0 (0.0-0.1)
Mycobacterium tuberculosis	Extensive drug resistance in TB	2 (1-4)	0.0 (0.0-0.0)	162 (61-335)	0.4 (0.2-0.9)	1 (0-2)	0.0 (0.0-0.0)	94 (36-198)	0.2 (0.1-0.5)
Mycobacterium tuberculosis	Isoniazid mono-resistance	6 (3-9)	0.0 (0.0-0.0)	544 (341-822)	1.4 (0.9-2.1)	1 (0-3)	0.0 (0.0-0.0)	79 (0-284)	0.2 (0.0-0.7)
Mycobacterium tuberculosis	Multi-drug resistance excluding extensive drug resistance in TB	11 (4-23)	0.0 (0.0-0.1)	1010 (399-2150)	2.6 (1.0-5.6)	6 (1-17)	0.0 (0.0-0.0)	552 (53-1490)	1.4 (0.1-3.9)
Mycobacterium tuberculosis	Rifampicin mono-resistance	2 (1-2)	0.0 (0.0-0.0)	151 (98-217)	0.4 (0.3-0.6)	0 (0-1)	0.0 (0.0-0.0)	40 (8-82)	0.1 (0.0-0.2)
Mycobacterium tuberculosis	Resistance to one or more antibiotics	20 (11-34)	0.1 (0.0-0.1)	1870 (1090-3150)	4.9 (2.8-8.2)	9 (2-21)	0.0 (0.0-0.1)	766 (177-1830)	2.0 (0.5-4.8)
Neisseria gonorrhoeae	Fluoroquinolones	--	--	--	--	--	--	--	--
Neisseria gonorrhoeae	Third-generation cephalosporins	--	--	--	--	--	--	--	--
Neisseria gonorrhoeae	Resistance to one or more antibiotics	--	--	--	--	--	--	--	--
Proteus spp.	Aminoglycosides	2 (1-3)	0.0 (0.0-0.0)	164 (97-268)	0.4 (0.3-0.7)	0 (0-0)	0.0 (0.0-0.0)	9 (1-22)	0.0 (0.0-0.1)
Proteus spp.	Aminopenicillin	23 (13-36)	0.1 (0.0-0.1)	2040 (1220-3230)	5.3 (3.2-8.4)	1 (0-2)	0.0 (0.0-0.0)	48 (0-149)	0.1 (0.0-0.4)
Proteus spp.	Fluoroquinolones	9 (5-13)	0.0 (0.0-0.0)	761 (458-1190)	2.0 (1.2-3.1)	1 (0-1)	0.0 (0.0-0.0)	54 (20-118)	0.1 (0.1-0.3)
Proteus spp.	Third-generation cephalosporins	5 (3-9)	0.0 (0.0-0.0)	488 (284-785)	1.3 (0.7-2.0)	1 (0-3)	0.0 (0.0-0.0)	113 (39-231)	0.3 (0.1-0.6)
Proteus spp.	Trimethoprim-Sulfamethoxazole	13 (7-20)	0.0 (0.0-0.1)	1120 (668-1780)	2.9 (1.7-4.6)	0 (0-1)	0.0 (0.0-0.0)	44 (1-103)	0.1 (0.0-0.3)
Proteus spp.	Resistance to one or more antibiotics	24 (14-39)	0.1 (0.0-0.1)	2200 (1320-3460)	5.7 (3.4-9.0)	3 (1-5)	0.0 (0.0-0.0)	268 (128-482)	0.7 (0.3-1.3)
Pseudomonas aeruginosa	Aminoglycosides	79 (54-111)	0.2 (0.1-0.3)	6820 (4690-9610)	17.7 (12.2-25.0)	2 (0-5)	0.0 (0.0-0.0)	177 (1-436)	0.5 (0.0-1.1)
Pseudomonas aeruginosa	Anti-pseudomonal penicillin/Beta-Lactamase inhibitors	135 (95-191)	0.4 (0.2-0.5)	11700 (8230-16500)	30.5 (21.4-42.8)	7 (4-11)	0.0 (0.0-0.0)	608 (375-962)	1.6 (1.0-2.5)
Pseudomonas aeruginosa	Carbapenem	201 (142-279)	0.5 (0.4-0.7)	17500 (12300-24100)	45.4 (32.1-62.8)	36 (24-53)	0.1 (0.1-0.1)	3120 (2090-4620)	8.1 (5.4-12.0)
Pseudomonas aeruginosa	Fluoroquinolones	150 (104-208)	0.4 (0.3-0.5)	13000 (9070-18000)	33.9 (23.6-46.8)	16 (10-23)	0.0 (0.0-0.1)	1360 (892-2020)	3.5 (2.3-5.3)
Pseudomonas aeruginosa	Fourth-generation cephalosporins	117 (81-164)	0.3 (0.2-0.4)	10100 (7010-14200)	26.3 (18.2-36.9)	2 (1-3)	0.0 (0.0-0.0)	139 (78-223)	0.4 (0.2-0.6)
Pseudomonas aeruginosa	Third-generation cephalosporins	127 (86-180)	0.3 (0.2-0.5)	11000 (7510-15500)	28.5 (19.5-40.4)	3 (1-6)	0.0 (0.0-0.0)	300 (124-560)	0.8 (0.3-1.5)
Pseudomonas aeruginosa	Resistance to one or more antibiotics	268 (188-374)	0.7 (0.5-1.0)	23300 (16300-32300)	60.5 (42.5-84.0)	66 (44-98)	0.2 (0.1-0.3)	5700 (3790-8520)	14.8 (9.9-22.1)
Salmonella Paratyphi	Fluoroquinolones	0 (0-1)	0.0 (0.0-0.0)	29 (10-67)	0.1 (0.0-0.2)	0 (0-0)	0.0 (0.0-0.0)	6 (1-17)	0.0 (0.0-0.0)
Salmonella Paratyphi	Multi-drug resistance in Salmonella Typhi and Paratyphi	0 (0-0)	0.0 (0.0-0.0)	1 (0-2)	0.0 (0.0-0.0)	0 (0-0)	0.0 (0.0-0.0)	0 (0-0)	0.0 (0.0-0.0)
Salmonella Paratyphi	Resistance to one or more antibiotics	0 (0-1)	0.0 (0.0-0.0)	29 (10-68)	0.1 (0.0-0.2)	0 (0-0)	0.0 (0.0-0.0)	6 (1-17)	0.0 (0.0-0.0)
Salmonella Typhi	Fluoroquinolones	19 (8-39)	0.0 (0.0-0.1)	1650 (693-3370)	4.3 (1.8-8.8)	4 (1-10)	0.0 (0.0-0.0)	335 (45-824)	0.9 (0.1-2.1)
Salmonella Typhi	Multi-drug resistance in Salmonella Typhi and Paratyphi	4 (1-8)	0.0 (0.0-0.0)	313 (122-675)	0.8 (0.3-1.8)	0 (0-1)	0.0 (0.0-0.0)	39 (0-125)	0.1 (0.0-0.3)
Salmonella Typhi	Resistance to one or more antibiotics	22 (10-42)	0.1 (0.0-0.1)	1910 (856-3660)	5.0 (2.2-9.5)	4 (1-10)	0.0 (0.0-0.0)	374 (77-866)	1.0 (0.2-2.3)
Non-typhoidal *Salmonella*	Fluoroquinolones	5 (2-13)	0.0 (0.0-0.0)	910 (151-3080)	2.4 (0.4-8.0)	1 (0-3)	0.0 (0.0-0.0)	124 (15-394)	0.3 (0.0-1.0)
Non-typhoidal *Salmonella*	Resistance to one or more antibiotics	5 (2-13)	0.0 (0.0-0.0)	910 (151-3080)	2.4 (0.4-8.0)	1 (0-3)	0.0 (0.0-0.0)	124 (15-394)	0.3 (0.0-1.0)
Serratia spp.	Aminoglycosides	6 (3-10)	0.0 (0.0-0.0)	498 (250-869)	1.3 (0.7-2.3)	0 (0-1)	0.0 (0.0-0.0)	29 (0-85)	0.1 (0.0-0.2)
Serratia spp.	Anti-pseudomonal penicillin/Beta-Lactamase inhibitors	5 (3-9)	0.0 (0.0-0.0)	446 (229-784)	1.2 (0.6-2.0)	1 (0-2)	0.0 (0.0-0.0)	82 (36-164)	0.2 (0.1-0.4)
Serratia spp.	Carbapenem	6 (3-11)	0.0 (0.0-0.0)	532 (274-916)	1.4 (0.7-2.4)	2 (1-3)	0.0 (0.0-0.0)	144 (65-277)	0.4 (0.2-0.7)
Serratia spp.	Fluoroquinolones	4 (2-7)	0.0 (0.0-0.0)	339 (177-606)	0.9 (0.5-1.6)	0 (0-1)	0.0 (0.0-0.0)	35 (8-83)	0.1 (0.0-0.2)
Serratia spp.	Fourth-generation cephalosporins	9 (5-16)	0.0 (0.0-0.0)	805 (420-1410)	2.1 (1.1-3.7)	1 (0-2)	0.0 (0.0-0.0)	74 (28-159)	0.2 (0.1-0.4)

Pathogen	Antibiotic Class	5 and over							
		Associated with resistance				Attributable to resistance			
		Deaths		DALYs		Deaths		DALYs	
		Counts	Rate per 100 000	Counts	Rate per 100 000	Counts	Rate per 100 000	Counts	Rate per 100 000
All pathogens	Resistance to one or more antibiotics	307000 (220000-413000)	57.2 (41.1-77.0)	6850000 (4780000-9420000)	1276.9 (891.6-1757.3)	76700 (54500-107000)	14.3 (10.2-19.9)	1710000 (1190000-2440000)	319.5 (221.0-454.5)
Acinetobacter baumannii	Aminoglycosides	19000 (10600-30200)	3.5 (2.0-5.6)	420000 (230000-665000)	78.2 (42.9-123.9)	791 (0-2030)	0.1 (0.0-0.4)	17600 (111-45200)	3.3 (0.0-8.4)
Acinetobacter baumannii	Anti-pseudomonal penicillin/Beta-Lactamase inhibitors	23400 (13500-36600)	4.4 (2.5-6.8)	517000 (289000-830000)	96.4 (53.9-154.8)	294 (133-545)	0.1 (0.0-0.1)	6680 (3000-12300)	1.2 (0.6-2.3)
Acinetobacter baumannii	Beta Lactam/Beta-lactamase inhibitors	21800 (12500-33900)	4.1 (2.3-6.3)	480000 (270000-753000)	89.5 (50.3-140.3)	44 (10-98)	0.0 (0.0-0.0)	947 (218-2150)	0.2 (0.0-0.4)
Acinetobacter baumannii	Carbapenem	23100 (13300-36100)	4.3 (2.5-6.7)	508000 (285000-798000)	94.7 (53.2-148.8)	3830 (1930-6830)	0.7 (0.4-1.3)	84400 (41400-153000)	15.7 (7.7-28.5)
Acinetobacter baumannii	Fluoroquinolones	23300 (13400-36400)	4.4 (2.5-6.8)	514000 (292000-806000)	95.9 (54.5-150.4)	2900 (1380-4970)	0.5 (0.3-0.9)	63700 (29700-112000)	11.9 (5.5-20.9)
Acinetobacter baumannii	Fourth-generation cephalosporins	23900 (13700-37300)	4.5 (2.5-6.9)	527000 (295000-830000)	98.3 (55.1-154.7)	113 (44-242)	0.0 (0.0-0.0)	2440 (927-5340)	0.5 (0.2-1.0)
Acinetobacter baumannii	Third-generation cephalosporins	24400 (14000-38200)	4.6 (2.6-7.1)	539000 (304000-860000)	100.5 (56.7-160.4)	320 (115-617)	0.1 (0.0-0.1)	7020 (2550-13900)	1.3 (0.5-2.6)
Acinetobacter baumannii	Resistance to one or more antibiotics	26000 (15000-40500)	4.9 (2.8-7.5)	574000 (324000-905000)	107.1 (60.5-168.7)	8290 (4560-13500)	1.5 (0.8-2.5)	183000 (97700-300000)	34.1 (18.2-55.9)
Citrobacter spp.	Aminoglycosides	149 (95-223)	0.0 (0.0-0.0)	3530 (2200-5350)	0.7 (0.4-1.0)	9 (0-24)	0.0 (0.0-0.0)	222 (0-551)	0.0 (0.0-0.1)
Citrobacter spp.	Anti-pseudomonal penicillin/Beta-Lactamase inhibitors	303 (189-457)	0.1 (0.0-0.1)	7130 (4340-10900)	1.3 (0.8-2.0)	67 (33-116)	0.0 (0.0-0.0)	1590 (769-2790)	0.3 (0.1-0.5)
Citrobacter spp.	Carbapenem	177 (113-266)	0.0 (0.0-0.0)	4120 (2510-6310)	0.8 (0.5-1.2)	46 (24-79)	0.0 (0.0-0.0)	1060 (537-1860)	0.2 (0.1-0.3)
Citrobacter spp.	Fluoroquinolones	375 (236-560)	0.1 (0.0-0.1)	8790 (5300-13400)	1.6 (1.0-2.5)	61 (26-109)	0.0 (0.0-0.0)	1420 (622-2590)	0.3 (0.1-0.5)
Citrobacter spp.	Fourth-generation cephalosporins	337 (208-514)	0.1 (0.0-0.1)	8000 (4790-12400)	1.5 (0.9-2.3)	34 (15-63)	0.0 (0.0-0.0)	810 (351-1520)	0.2 (0.1-0.3)
Citrobacter spp.	Third-generation cephalosporins	904 (575-1360)	0.2 (0.1-0.3)	21200 (13100-32700)	4.0 (2.4-6.1)	121 (53-223)	0.0 (0.0-0.0)	2830 (1190-5270)	0.5 (0.2-1.0)
Citrobacter spp.	Resistance to one or more antibiotics	1180 (763-1760)	0.2 (0.1-0.3)	27800 (17200-42000)	5.2 (3.2-7.8)	338 (191-550)	0.1 (0.0-0.1)	7930 (4400-13000)	1.5 (0.8-2.4)
Enterobacter spp.	Aminoglycosides	2100 (1390-3090)	0.4 (0.3-0.6)	48700 (31400-73500)	9.1 (5.9-13.7)	141 (42-278)	0.0 (0.0-0.1)	3280 (972-6560)	0.6 (0.2-1.2)
Enterobacter spp.	Anti-pseudomonal penicillin/Beta-Lactamase inhibitors	5080 (3340-7250)	0.9 (0.6-1.4)	118000 (75000-173000)	22.0 (14.0-32.3)	597 (325-955)	0.1 (0.1-0.2)	13900 (7490-22300)	2.6 (1.4-4.2)
Enterobacter spp.	Carbapenem	2520 (1650-3690)	0.5 (0.3-0.7)	58100 (36800-86700)	10.8 (6.9-16.2)	634 (370-995)	0.1 (0.1-0.2)	14600 (8280-23200)	2.7 (1.5-4.3)
Enterobacter spp.	Fluoroquinolones	2680 (1680-3900)	0.5 (0.3-0.7)	63500 (39000-96100)	11.8 (7.3-17.9)	302 (158-518)	0.1 (0.0-0.1)	7150 (3670-12500)	1.3 (0.7-2.3)
Enterobacter spp.	Fourth-generation cephalosporins	5900 (4000-8360)	1.1 (0.7-1.6)	138000 (88700-200000)	25.6 (16.5-37.2)	358 (171-632)	0.1 (0.0-0.1)	8380 (4000-14900)	1.6 (0.7-2.8)
Enterobacter spp.	Trimethoprim-Sulfamethoxazole	6160 (4080-8890)	1.1 (0.8-1.7)	140000 (90400-206000)	26.1 (16.9-38.4)	388 (0-820)	0.1 (0.0-0.2)	8780 (0-18900)	1.6 (0.0-3.5)
Enterobacter spp.	Resistance to one or more antibiotics	10800 (7240-15200)	2.0 (1.3-2.8)	249000 (161000-362000)	46.5 (30.1-67.5)	2420 (1510-3540)	0.5 (0.3-0.7)	56100 (34400-83900)	10.5 (6.4-15.6)
Enterococcus faecalis	Fluoroquinolones	6010 (3720-9170)	1.1 (0.7-1.7)	152000 (93800-230000)	28.4 (17.5-42.9)	1460 (650-2670)	0.3 (0.1-0.5)	36800 (16700-66400)	6.9 (3.1-12.4)
Enterococcus faecalis	Vancomycin	1520 (933-2450)	0.3 (0.2-0.5)	37400 (22900-57400)	7.0 (4.3-10.7)	504 (226-945)	0.1 (0.0-0.2)	12300 (5610-22400)	2.3 (1.0-4.2)
Enterococcus faecalis	Resistance to one or more antibiotics	6820 (4240-10300)	1.3 (0.8-1.9)	172000 (106000-256000)	32.1 (19.9-47.7)	1960 (997-3360)	0.4 (0.2-0.6)	49100 (25500-81800)	9.2 (4.7-15.2)
Enterococcus faecium	Fluoroquinolones	14900 (9340-22300)	2.8 (1.7-4.2)	361000 (221000-548000)	67.3 (41.2-102.1)	2400 (727-4570)	0.4 (0.1-0.9)	58400 (17500-112000)	10.9 (3.3-20.9)
Enterococcus faecium	Vancomycin	9350 (5940-13900)	1.7 (1.1-2.6)	223000 (138000-337000)	41.6 (25.7-62.8)	2370 (1260-3900)	0.4 (0.2-0.7)	56500 (29400-94200)	10.5 (5.5-17.6)
Enterococcus faecium	Resistance to one or more antibiotics	15900 (10100-23500)	3.0 (1.9-4.4)	383000 (236000-580000)	71.5 (44.0-108.1)	4780 (2870-7650)	0.9 (0.5-1.4)	115000 (66800-188000)	21.4 (12.5-35.0)
Other enterococci	Fluoroquinolones	7160 (5070-9960)	1.3 (0.9-1.9)	138000 (95300-197000)	25.8 (17.8-36.8)	1300 (197-2420)	0.2 (0.0-0.5)	24800 (4200-46700)	4.6 (0.8-8.7)
Other enterococci	Vancomycin	1700 (1150-2340)	0.3 (0.2-0.4)	32500 (21800-46200)	6.1 (4.1-8.6)	282 (154-523)	0.1 (0.0-0.1)	5490 (2980-10400)	1.0 (0.6-1.9)
Other enterococci	Resistance to one or more antibiotics	7180 (5110-9960)	1.3 (1.0-1.9)	139000 (95600-198000)	25.9 (17.8-36.8)	1580 (622-2700)	0.3 (0.1-0.5)	30300 (11900-52100)	5.6 (2.2-9.7)
Escherichia coli	Aminoglycosides	8560 (6060-11700)	1.6 (1.1-2.2)	186000 (128000-257000)	34.7 (23.9-48.0)	526 (339-762)	0.1 (0.1-0.1)	11400 (7230-16700)	2.1 (1.3-3.1)
Escherichia coli	Aminopenicillin	55600 (39800-75000)	10.4 (7.4-14.0)	1200000 (845000-1660000)	223.9 (157.6-309.8)	943 (599-1360)	0.2 (0.1-0.3)	19900 (12500-28900)	3.7 (2.3-5.4)
Escherichia coli	Beta Lactam/Beta-lactamase inhibitors	43100 (30900-58400)	8.0 (5.8-10.9)	931000 (653000-1280000)	173.6 (121.7-239.4)	2160 (1500-2990)	0.4 (0.3-0.6)	45500 (31100-64500)	8.5 (5.8-12.0)
Escherichia coli	Carbapenem	2550 (1760-3620)	0.5 (0.3-0.7)	55300 (37400-79600)	10.3 (7.0-14.8)	580 (326-924)	0.1 (0.1-0.2)	12500 (6940-20400)	2.3 (1.3-3.8)
Escherichia coli	Fluoroquinolones	34200 (24300-46900)	6.4 (4.5-8.7)	748000 (518000-1050000)	139.5 (96.7-195.0)	3630 (2430-5130)	0.7 (0.5-1.0)	79100 (52100-113000)	14.7 (9.7-21.1)
Escherichia coli	Third-generation cephalosporins	28400 (19900-39700)	5.3 (3.7-7.4)	629000 (431000-891000)	117.3 (80.3-166.2)	4000 (1750-7280)	0.7 (0.3-1.4)	88700 (39200-162000)	16.5 (7.3-30.2)
Escherichia coli	Trimethoprim-Sulfamethoxazole	42900 (31100-58000)	8.0 (5.8-10.8)	923000 (654000-1270000)	172.0 (121.9-236.3)	2660 (1790-3770)	0.5 (0.3-0.7)	56200 (36900-80200)	10.5 (6.9-15.0)
Escherichia coli	Resistance to one or more antibiotics	60600 (43600-82000)	11.3 (8.1-15.3)	1310000 (923000-1810000)	243.9 (172.0-337.0)	14500 (9990-20900)	2.7 (1.9-3.9)	313000 (211000-459000)	58.4 (39.4-85.7)
Group A *Streptococcus*	Macrolide	1680 (896-3050)	0.3 (0.2-0.6)	40600 (23100-72700)	7.6 (4.3-13.6)	161 (0-586)	0.0 (0.0-0.1)	3870 (0-13300)	0.7 (0.0-2.5)
Group A *Streptococcus*	Resistance to one or more antibiotics	1680 (896-3050)	0.3 (0.2-0.6)	40600 (23100-72700)	7.6 (4.3-13.6)	161 (0-586)	0.0 (0.0-0.1)	3870 (0-13300)	0.7 (0.0-2.5)
Group B *Streptococcus*	Fluoroquinolones	848 (563-1260)	0.2 (0.1-0.2)	20800 (13900-30800)	3.9 (2.6-5.7)	143 (24-342)	0.0 (0.0-0.1)	3490 (592-8180)	0.7 (0.1-1.5)
Group B Streptococcus	Macrolide	4390 (2980-6260)	0.8 (0.6-1.2)	106000 (71200-154000)	19.7 (13.3-28.6)	451 (0-1250)	0.1 (0.0-0.2)	10800 (0-30500)	2.0 (0.0-5.7)
Group B Streptococcus	Penicillin	129 (84-190)	0.0 (0.0-0.0)	3090 (1970-4620)	0.6 (0.4-0.9)	29 (6-63)	0.0 (0.0-0.0)	685 (149-1520)	0.1 (0.0-0.3)
Group B Streptococcus	Resistance to one or more antibiotics	4790 (3320-6890)	0.9 (0.6-1.3)	116000 (78500-167000)	21.6 (14.6-31.2)	623 (0-1420)	0.1 (0.0-0.3)	15000 (0-34800)	2.8 (0.0-6.5)
Haemophilus influenzae	Aminopenicillin	813 (618-1060)	0.2 (0.1-0.2)	18400 (13900-23900)	3.4 (2.6-4.5)	134 (11-268)	0.0 (0.0-0.0)	3000 (269-5960)	0.6 (0.1-1.1)
Haemophilus influenzae	Third-generation cephalosporins	199 (152-265)	0.0 (0.0-0.0)	4550 (3460-6030)	0.8 (0.6-1.1)	58 (27-100)	0.0 (0.0-0.0)	1330 (643-2270)	0.2 (0.1-0.4)
Haemophilus influenzae	Resistance to one or more antibiotics	918 (709-1170)	0.2 (0.1-0.2)	20800 (15900-26700)	3.9 (3.0-5.0)	192 (67-335)	0.0 (0.0-0.1)	4330 (1590-7520)	0.8 (0.3-1.4)
Klebsiella pneumoniae	Aminoglycosides	19100 (13500-27100)	3.6 (2.5-5.0)	410000 (281000-585000)	76.5 (52.5-109.1)	1510 (923-2330)	0.3 (0.2-0.4)	32400 (19400-50700)	6.0 (3.6-9.5)
Klebsiella pneumoniae	Beta Lactam/Beta-lactamase inhibitors	36400 (25800-49700)	6.8 (4.8-9.3)	787000 (546000-1100000)	146.8 (101.8-205.2)	922 (481-1550)	0.2 (0.1-0.3)	20100 (10400-33800)	3.7 (1.9-6.3)
Klebsiella pneumoniae	Carbapenem	11900 (8260-17400)	2.2 (1.5-3.2)	253000 (172000-368000)	47.1 (32.1-68.6)	2750 (1700-4120)	0.5 (0.3-0.8)	58100 (35200-88000)	10.8 (6.6-16.4)
Klebsiella pneumoniae	Fluoroquinolones	24800 (17600-34200)	4.6 (3.3-6.4)	533000 (368000-739000)	99.3 (68.6-137.8)	1780 (1070-2740)	0.3 (0.2-0.5)	38500 (22900-60100)	7.2 (4.3-11.2)
Klebsiella pneumoniae	Third-generation cephalosporins	31000 (20800-44300)	5.8 (3.9-8.3)	670000 (446000-969000)	124.9 (83.2-180.6)	3510 (1270-6800)	0.7 (0.2-1.3)	76500 (27100-149000)	14.3 (5.1-27.8)
Klebsiella pneumoniae	Trimethoprim-Sulfamethoxazole	29300 (20800-40500)	5.5 (3.9-7.6)	633000 (436000-888000)	118.0 (81.3-165.5)	1480 (732-2520)	0.3 (0.1-0.5)	31800 (15600-55500)	5.9 (2.9-10.3)
Klebsiella pneumoniae	Resistance to one or more antibiotics	42500 (30100-58400)	7.9 (5.6-10.9)	918000 (634000-1280000)	171.1 (118.3-238.1)	11900 (7830-17200)	2.2 (1.5-3.2)	257000 (166000-376000)	48.0 (31.0-70.1)
Morganella spp.	Fluoroquinolones	243 (165-346)	0.0 (0.0-0.1)	4300 (2870-6200)	0.8 (0.5-1.2)	44 (17-81)	0.0 (0.0-0.0)	778 (304-1440)	0.1 (0.1-0.3)
Morganella spp.	Fourth-generation cephalosporins	67 (45-96)	0.0 (0.0-0.0)	1140 (755-1630)	0.2 (0.1-0.3)	13 (5-24)	0.0 (0.0-0.0)	216 (92-403)	0.0 (0.0-0.1)
Morganella spp.	Third-generation cephalosporins	88 (58-127)	0.0 (0.0-0.0)	1470 (967-2110)	0.3 (0.2-0.4)	11 (5-23)	0.0 (0.0-0.0)	191 (76-393)	0.0 (0.0-0.1)
Morganella spp.	Resistance to one or more antibiotics	294 (205-412)	0.1 (0.0-0.1)	5160 (3530-7330)	1.0 (0.7-1.4)	69 (37-111)	0.0 (0.0-0.0)	1180 (641-1960)	0.2 (0.1-0.4)
Mycobacterium tuberculosis	Extensive drug resistance in TB	195 (78-400)	0.0 (0.0-0.1)	6660 (2650-13800)	1.2 (0.5-2.6)	119 (48-245)	0.0 (0.0-0.0)	3890 (1500-8020)	0.7 (0.3-1.5)
Mycobacterium tuberculosis	Isoniazid mono-resistance	627 (410-911)	0.1 (0.1-0.2)	23400 (15800-33200)	4.4 (2.9-6.2)	100 (0-344)	0.0 (0.0-0.1)	3420 (0-11700)	0.6 (0.0-2.2)
Mycobacterium tuberculosis	Multi-drug resistance excluding extensive drug resistance in TB	1180 (479-2430)	0.2 (0.1-0.5)	41700 (17100-86100)	7.8 (3.2-16.1)	698 (72-1900)	0.1 (0.0-0.4)	22700 (2190-61900)	4.2 (0.4-11.5)
Mycobacterium tuberculosis	Rifampicin mono-resistance	159 (110-221)	0.0 (0.0-0.0)	5870 (4120-8040)	1.1 (0.8-1.5)	45 (7-91)	0.0 (0.0-0.0)	1550 (295-3110)	0.3 (0.1-0.6)
Mycobacterium tuberculosis	Resistance to one or more antibiotics	2160 (1320-3550)	0.4 (0.2-0.7)	77700 (48900-126000)	14.5 (9.1-23.5)	961 (224-2260)	0.2 (0.0-0.4)	31600 (7430-74000)	5.9 (1.4-13.8)
Neisseria gonorrhoeae	Fluoroquinolones	--	--	2980 (1730-4690)	0.6 (0.3-0.9)	--	--	287 (78-572)	0.1 (0.0-0.1)
Neisseria gonorrhoeae	Third-generation cephalosporins	--	--	27 (10-66)	0.0 (0.0-0.0)	--	--	7 (2-18)	0.0 (0.0-0.0)
Neisseria gonorrhoeae	Resistance to one or more antibiotics	--	--	2980 (1730-4710)	0.6 (0.3-0.9)	--	--	295 (85-578)	0.1 (0.0-0.1)
Proteus spp.	Aminoglycosides	382 (261-530)	0.1 (0.0-0.1)	7730 (5160-11000)	1.4 (1.0-2.1)	22 (2-48)	0.0 (0.0-0.0)	455 (54-988)	0.1 (0.0-0.2)
Proteus spp.	Aminopenicillin	6270 (4410-8580)	1.2 (0.8-1.6)	125000 (85700-176000)	23.3 (16.0-32.8)	147 (0-435)	0.0 (0.0-0.1)	2930 (0-8680)	0.5 (0.0-1.6)
Proteus spp.	Fluoroquinolones	2460 (1740-3360)	0.5 (0.3-0.6)	49100 (33700-68500)	9.2 (6.3-12.8)	184 (73-356)	0.0 (0.0-0.1)	3620 (1430-7030)	0.7 (0.3-1.3)
Proteus spp.	Third-generation cephalosporins	1470 (1010-2070)	0.3 (0.2-0.4)	29500 (19400-42200)	5.5 (3.6-7.9)	341 (118-649)	0.1 (0.0-0.1)	6830 (2350-13100)	1.3 (0.4-2.4)
Proteus spp.	Trimethoprim-Sulfamethoxazole	3350 (2350-4660)	0.6 (0.4-0.9)	67700 (46100-96100)	12.6 (8.6-17.9)	132 (0-292)	0.0 (0.0-0.1)	2660 (2-5860)	0.5 (0.0-1.1)
Proteus spp.	Resistance to one or more antibiotics	6740 (4750-9200)	1.3 (0.9-1.7)	135000 (92000-190000)	25.1 (17.2-35.4)	826 (438-1340)	0.2 (0.1-0.2)	16500 (8800-26900)	3.1 (1.6-5.0)
Pseudomonas aeruginosa	Aminoglycosides	8070 (5480-11500)	1.5 (1.0-2.1)	176000 (116000-255000)	32.7 (21.6-47.5)	201 (0-505)	0.0 (0.0-0.1)	4450 (45-11100)	0.8 (0.0-2.1)
Pseudomonas aeruginosa	Anti-pseudomonal penicillin/Beta-Lactamase inhibitors	13700 (9390-19400)	2.5 (1.8-3.6)	296000 (197000-431000)	55.1 (36.8-80.3)	743 (446-1200)	0.1 (0.1-0.2)	15900 (9340-26300)	3.0 (1.7-4.9)
Pseudomonas aeruginosa	Carbapenem	19800 (13500-28100)	3.7 (2.5-5.2)	430000 (284000-623000)	80.2 (53.0-116.1)	3500 (2270-5210)	0.7 (0.4-1.0)	76200 (47800-115000)	14.2 (8.9-21.4)
Pseudomonas aeruginosa	Fluoroquinolones	16100 (11200-22700)	3.0 (2.1-4.2)	349000 (234000-499000)	65.1 (43.7-93.1)	1720 (1100-2610)	0.3 (0.2-0.5)	37300 (23400-57500)	7.0 (4.4-10.7)
Pseudomonas aeruginosa	Fourth-generation cephalosporins	11900 (8150-16600)	2.2 (1.5-3.1)	258000 (172000-367000)	48.0 (32.0-68.4)	172 (99-284)	0.0 (0.0-0.1)	3710 (2110-6180)	0.7 (0.4-1.2)
Pseudomonas aeruginosa	Third-generation cephalosporins	13100 (8770-18600)	2.5 (1.6-3.5)	284000 (184000-411000)	52.9 (34.3-76.7)	357 (133-686)	0.1 (0.0-0.1)	7670 (3010-14700)	1.4 (0.6-2.7)
Pseudomonas aeruginosa	Resistance to one or more antibiotics	27400 (19200-38200)	5.1 (3.6-7.1)	594000 (401000-844000)	110.8 (74.8-157.4)	6690 (4370-10100)	1.2 (0.8-1.9)	145000 (92500-220000)	27.1 (17.2-41.1)
Salmonella Paratyphi	Fluoroquinolones	3 (2-5)	0.0 (0.0-0.0)	122 (65-222)	0.0 (0.0-0.0)	1 (0-1)	0.0 (0.0-0.0)	24 (4-55)	0.0 (0.0-0.0)
Salmonella Paratyphi	Multi-drug resistance in Salmonella Typhi and Paratyphi	0 (0-0)	0.0 (0.0-0.0)	2 (1-5)	0.0 (0.0-0.0)	0 (0-0)	0.0 (0.0-0.0)	0 (0-1)	0.0 (0.0-0.0)
Salmonella Paratyphi	Resistance to one or more antibiotics	3 (2-5)	0.0 (0.0-0.0)	124 (66-226)	0.0 (0.0-0.0)	1 (0-1)	0.0 (0.0-0.0)	25 (4-56)	0.0 (0.0-0.0)
Salmonella Typhi	Fluoroquinolones	472 (215-924)	0.1 (0.0-0.2)	17800 (8190-34600)	3.3 (1.5-6.4)	96 (13-232)	0.0 (0.0-0.0)	3610 (489-8730)	0.7 (0.1-1.6)
Salmonella Typhi	Multi-drug resistance in Salmonella Typhi and Paratyphi	82 (40-160)	0.0 (0.0-0.0)	3100 (1540-6100)	0.6 (0.3-1.1)	10 (0-31)	0.0 (0.0-0.0)	384 (0-1150)	0.1 (0.0-0.2)
Salmonella Typhi	Resistance to one or more antibiotics	541 (263-1000)	0.1 (0.0-0.2)	20400 (10200-37700)	3.8 (1.9-7.0)	107 (22-245)	0.0 (0.0-0.0)	4000 (854-9240)	0.7 (0.2-1.7)
Non-typhoidal *Salmonella*	Fluoroquinolones	95 (40-193)	0.0 (0.0-0.0)	4150 (1300-10700)	0.8 (0.2-2.0)	20 (2-54)	0.0 (0.0-0.0)	660 (108-1790)	0.1 (0.0-0.3)
Non-typhoidal *Salmonella*	Resistance to one or more antibiotics	95 (40-193)	0.0 (0.0-0.0)	4150 (1300-10700)	0.8 (0.2-2.0)	20 (2-54)	0.0 (0.0-0.0)	660 (108-1790)	0.1 (0.0-0.3)
Serratia spp.	Aminoglycosides	638 (371-1010)	0.1 (0.1-0.2)	16000 (9200-25200)	3.0 (1.7-4.7)	37 (0-102)	0.0 (0.0-0.0)	946 (0-2550)	0.2 (0.0-0.5)
Serratia spp.	Anti-pseudomonal penicillin/Beta-Lactamase inhibitors	644 (372-1050)	0.1 (0.1-0.2)	16000 (9180-26200)	3.0 (1.7-4.9)	119 (56-218)	0.0 (0.0-0.0)	2960 (1380-5400)	0.6 (0.3-1.0)
Serratia spp.	Carbapenem	776 (442-1230)	0.1 (0.1-0.2)	19200 (10900-30700)	3.6 (2.0-5.7)	211 (106-374)	0.0 (0.0-0.1)	5230 (2630-9250)	1.0 (0.5-1.7)
Serratia spp.	Fluoroquinolones	451 (258-718)	0.1 (0.0-0.1)	11300 (6520-18000)	2.1 (1.2-3.4)	47 (10-104)	0.0 (0.0-0.0)	1190 (267-2650)	0.2 (0.0-0.5)
Serratia spp.	Fourth-generation cephalosporins	986 (580-1590)	0.2 (0.1-0.3)	24700 (14700-40100)	4.6 (2.7-7.5)	83 (36-171)	0.0 (0.0-0.0)	2090 (908-4280)	0.4 (0.2-0.8)

Serratia spp.	Third-generation cephalosporins	196 (125-301)	26.5 (16.9-40.7)	17400 (11100-26700)	2356.5 (1506.5-3615.5)	15 (6-29)	2.1 (0.8-4.0)	1360 (494-2620)	183.5 (66.8-354.3)	57 (34-92)	0.6 (0.4-1.0)	5030 (3030-8120)	56.7 (34.2-91.6)	4 (2-9)	0.0 (0.0-0.1)	386 (136-783)	4.4 (1.5-8.8)	13 (7-23)	0.0 (0.0-0.1)	1160 (579-2020)	3.0 (1.5-5.3)	1 (0-2)	0.0 (0.0-0.0)	89 (30-192)	0.2 (0.1-0.5)	1680 (1000-2680)	0.3 (0.2-0.5)	41900 (24600-66600)	7.8 (4.6-12.4)	138 (48-276)	0.0 (0.0-0.1)	3440 (1270-6890)	0.6 (0.2-1.3)
Serratia spp.	Resistance to one or more antibiotics	285 (186-414)	38.5 (25.1-55.9)	25300 (16500-36800)	3424.2 (2234.0-4975.2)	76 (43-123)	10.3 (5.8-16.6)	6740 (3780-10900)	911.6 (511.6-1475.9)	80 (49-127)	0.9 (0.5-1.4)	7090 (4310-11300)	80.0 (48.6-127.0)	22 (12-36)	0.2 (0.1-0.4)	1920 (1070-3210)	21.6 (12.0-36.2)	20 (10-34)	0.1 (0.0-0.1)	1710 (905-2960)	4.4 (2.4-7.7)	5 (3-10)	0.0 (0.0-0.0)	453 (220-824)	1.2 (0.6-2.1)	2350 (1420-3700)	0.4 (0.3-0.7)	58800 (35300-92400)	11.0 (6.6-17.2)	636 (344-1100)	0.1 (0.1-0.2)	15900 (8470-27500)	3.0 (1.6-5.1)
Shigella spp.	Fluoroquinolones	5 (1-12)	0.6 (0.2-1.6)	432 (129-1050)	58.4 (17.4-141.3)	1 (0-3)	0.1 (0.0-0.4)	89 (10-279)	12.0 (1.4-37.8)	44 (15-96)	0.5 (0.2-1.1)	4060 (1390-8800)	45.8 (15.7-99.2)	9 (1-26)	0.1 (0.0-0.3)	837 (103-2270)	9.4 (1.2-25.6)	21 (7-46)	0.1 (0.0-0.1)	2230 (758-4690)	5.8 (2.0-12.2)	4 (0-12)	0.0 (0.0-0.0)	427 (69-1170)	1.1 (0.2-3.0)	56 (21-118)	0.0 (0.0-0.0)	2670 (1040-5590)	0.5 (0.2-1.0)	12 (1-30)	0.0 (0.0-0.0)	426 (78-1060)	0.1 (0.0-0.2)
Shigella spp.	Resistance to one or more antibiotics	5 (1-12)	0.6 (0.2-1.6)	432 (129-1050)	58.4 (17.4-141.3)	1 (0-3)	0.1 (0.0-0.4)	89 (10-279)	12.0 (1.4-37.8)	44 (15-96)	0.5 (0.2-1.1)	4060 (1390-8800)	45.8 (15.7-99.2)	9 (1-26)	0.1 (0.0-0.3)	837 (103-2270)	9.4 (1.2-25.6)	21 (7-46)	0.1 (0.0-0.1)	2230 (758-4690)	5.8 (2.0-12.2)	4 (0-12)	0.0 (0.0-0.0)	427 (69-1170)	1.1 (0.2-3.0)	56 (21-118)	0.0 (0.0-0.0)	2670 (1040-5590)	0.5 (0.2-1.0)	12 (1-30)	0.0 (0.0-0.0)	426 (78-1060)	0.1 (0.0-0.2)
Staphylococcus aureus	Fluoroquinolones	766 (508-1090)	103.6 (68.6-147.5)	68200 (45100-97100)	9217.0 (6103.1-13125.8)	35 (14-61)	4.7 (2.0-8.3)	3090 (1290-5470)	417.4 (174.0-739.1)	642 (442-888)	7.2 (5.0-10.0)	56900 (39200-78600)	641.5 (441.8-887.0)	29 (12-50)	0.3 (0.1-0.6)	2590 (1100-4420)	29.2 (12.5-49.9)	254 (179-348)	0.7 (0.5-0.9)	22200 (15700-30300)	57.7 (40.7-78.8)	11 (5-20)	0.0 (0.0-0.1)	996 (418-1700)	2.6 (1.1-4.4)	20200 (14900-27100)	3.8 (2.8-5.1)	442000 (313000-611000)	82.4 (58.4-113.9)	928 (409-1570)	0.2 (0.1-0.3)	20200 (8970-34900)	3.8 (1.7-6.5)
Staphylococcus aureus	Macrolide	1540 (1020-2180)	207.7 (138.4-295.1)	137000 (91000-194000)	18480.2 (12307.6-26249.8)	62 (23-112)	8.3 (3.1-15.1)	5470 (2070-9950)	739.9 (280.1-1345.2)	1310 (942-1790)	14.8 (10.6-20.2)	116000 (83400-158000)	1313.5 (941.1-1783.9)	53 (21-93)	0.6 (0.2-1.0)	4710 (1830-8240)	53.1 (20.6-92.9)	465 (335-632)	1.2 (0.9-1.6)	40700 (29600-55200)	105.7 (76.9-143.6)	18 (7-32)	0.0 (0.0-0.1)	1610 (637-2780)	4.2 (1.7-7.2)	44600 (33700-58000)	8.3 (6.3-10.8)	956000 (695000-1280000)	178.3 (129.7-238.7)	1830 (748-3180)	0.3 (0.1-0.6)	39100 (15800-69000)	7.3 (2.9-12.9)
Staphylococcus aureus	Methicillin	1490 (981-2130)	202.0 (132.6-288.4)	133000 (87300-190000)	17970.9 (11798.4-25656.0)	399 (173-688)	53.9 (23.4-93.1)	35500 (15400-61200)	4795.9 (2082.4-8272.2)	1200 (845-1660)	13.6 (9.5-18.7)	107000 (74800-147000)	1203.0 (843.5-1660.0)	321 (143-546)	3.6 (1.6-6.2)	28400 (12700-48300)	320.5 (143.3-544.5)	455 (324-631)	1.2 (0.8-1.6)	39800 (28400-55100)	103.5 (73.7-143.2)	120 (53-207)	0.3 (0.1-0.5)	10500 (4700-18000)	27.3 (12.2-46.8)	40600 (29700-54800)	7.6 (5.5-10.2)	880000 (629000-1220000)	164.1 (117.2-227.0)	10900 (4890-18100)	2.0 (0.9-3.4)	236000 (105000-399000)	44.0 (19.7-74.4)
Staphylococcus aureus	Trimethoprim-Sulfamethoxazole	233 (159-321)	31.6 (21.5-43.4)	20800 (14200-28600)	2808.4 (1915.9-3866.1)	22 (11-35)	2.9 (1.5-4.7)	1920 (955-3110)	259.6 (129.1-420.4)	161 (114-219)	1.8 (1.3-2.5)	14300 (10100-19400)	161.0 (113.6-219.3)	15 (7-24)	0.2 (0.1-0.3)	1310 (649-2100)	14.8 (7.3-23.6)	90 (63-123)	0.2 (0.2-0.3)	7810 (5440-10800)	20.3 (14.1-28.1)	8 (4-13)	0.0 (0.0-0.0)	732 (364-1140)	1.9 (0.9-3.0)	3250 (2370-4330)	0.6 (0.4-0.8)	74500 (52600-103000)	13.9 (9.8-19.2)	281 (146-441)	0.1 (0.0-0.1)	6450 (3340-10300)	1.2 (0.6-1.9)
Staphylococcus aureus	Vancomycin	31 (20-46)	4.2 (2.7-6.2)	2770 (1790-4110)	374.4 (242.4-555.7)	8 (4-16)	1.1 (0.6-2.1)	752 (366-1410)	101.7 (49.5-190.6)	26 (18-37)	0.3 (0.2-0.4)	2290 (1580-3260)	25.9 (17.8-36.8)	7 (3-12)	0.1 (0.0-0.1)	624 (303-1080)	7.0 (3.4-12.2)	10 (7-14)	0.0 (0.0-0.0)	851 (589-1200)	2.2 (1.5-3.1)	3 (1-5)	0.0 (0.0-0.0)	231 (114-407)	0.6 (0.3-1.1)	869 (618-1220)	0.2 (0.1-0.2)	18700 (13000-26600)	3.5 (2.4-5.0)	237 (120-427)	0.0 (0.0-0.1)	5100 (2580-9450)	1.0 (0.5-1.8)
Staphylococcus aureus	Resistance to one or more antibiotics	2070 (1370-2900)	280.1 (185.9-391.8)	184000 (122000-258000)	24921.2 (16537.4-34857.9)	525 (276-848)	71.0 (37.4-114.6)	46700 (24600-75400)	6314.4 (3324.8-10194.5)	1720 (1230-2330)	19.4 (13.8-26.3)	152000 (109000-206000)	1714.5 (1226.2-2325.1)	425 (239-681)	4.8 (2.7-7.7)	37600 (21100-60200)	424.6 (238.4-679.4)	640 (461-877)	1.7 (1.2-2.3)	56000 (40400-76400)	145.5 (105.0-198.6)	161 (92-254)	0.4 (0.2-0.7)	14000 (8030-22400)	36.5 (20.9-58.3)	57000 (42700-75200)	10.6 (8.0-14.0)	1230000 (896000-1680000)	229.6 (167.1-312.5)	14200 (7820-22000)	2.6 (1.5-4.1)	307000 (169000-489000)	57.3 (31.5-91.2)
Streptococcus pneumoniae	Beta Lactam/Beta-lactamase inhibitors	314 (198-474)	42.4 (26.7-64.0)	27900 (17600-42200)	3774.4 (2379.2-5698.2)	7 (0-18)	0.9 (0.0-2.4)	621 (16-1570)	83.9 (2.2-212.3)	719 (501-1000)	8.1 (5.6-11.3)	63600 (44300-88500)	717.9 (499.7-997.6)	16 (0-41)	0.2 (0.0-0.5)	1450 (39-3650)	16.3 (0.4-41.1)	295 (208-405)	0.8 (0.5-1.1)	25500 (18000-35000)	66.3 (46.8-91.0)	7 (0-18)	0.0 (0.0-0.0)	609 (14-1530)	1.6 (0.0-4.0)	5460 (4040-7180)	1.0 (0.8-1.3)	133000 (97300-178000)	24.8 (18.2-33.1)	111 (3-270)	0.0 (0.0-0.1)	2680 (95-6610)	0.5 (0.0-1.2)
Streptococcus pneumoniae	Carbapenem	507 (319-751)	68.6 (43.1-101.5)	45100 (28400-66800)	6102.9 (3836.7-9036.0)	111 (48-202)	15.0 (6.5-27.4)	9870 (4280-18000)	1334.4 (578.4-2435.7)	1160 (804-1610)	13.0 (9.1-18.1)	102000 (71100-142000)	1153.9 (802.1-1604.3)	254 (114-443)	2.9 (1.3-5.0)	22400 (10200-39200)	253.1 (114.6-442.1)	431 (305-578)	1.1 (0.8-1.5)	37400 (26400-50100)	97.1 (68.6-130.1)	93 (41-166)	0.2 (0.1-0.4)	8100 (3620-14300)	21.1 (9.4-37.1)	9860 (7470-12900)	1.8 (1.4-2.4)	242000 (183000-322000)	45.1 (34.1-60.0)	2190 (1060-3740)	0.4 (0.2-0.7)	53600 (26200-92600)	10.0 (4.9-17.3)
Streptococcus pneumoniae	Fluoroquinolones	168 (109-245)	22.7 (14.8-33.2)	15000 (9720-21800)	2021.1 (1314.1-2951.2)	20 (5-46)	2.8 (0.7-6.2)	1810 (444-4070)	245.0 (60.1-549.8)	373 (248-528)	4.2 (2.8-6.0)	33000 (21900-46700)	372.2 (247.1-527.0)	45 (10-95)	0.5 (0.1-1.1)	4000 (897-8380)	45.1 (10.1-94.6)	198 (132-279)	0.5 (0.3-0.7)	17100 (11400-24100)	44.4 (29.6-62.7)	24 (6-52)	0.1 (0.0-0.1)	2090 (485-4490)	5.4 (1.3-11.7)	1700 (1280-2280)	0.3 (0.2-0.4)	44300 (32900-60200)	8.3 (6.1-11.2)	197 (46-408)	0.0 (0.0-0.1)	5150 (1220-10800)	1.0 (0.2-2.0)
Streptococcus pneumoniae	Macrolide	1030 (651-1510)	139.0 (88.0-204.5)	91500 (57900-135000)	12364.4 (7825.2-18196.3)	41 (0-115)	5.5 (0.0-15.5)	3630 (0-10200)	490.8 (0.0-1376.0)	2420 (1760-3180)	27.2 (19.9-35.9)	214000 (156000-282000)	2410.6 (1757.0-3177.4)	97 (0-247)	1.1 (0.0-2.8)	8550 (0-21800)	96.5 (0.0-245.9)	866 (624-1140)	2.3 (1.6-3.0)	75100 (54200-98700)	195.2 (140.9-256.5)	34 (0-86)	0.1 (0.0-0.2)	2920 (0-7400)	7.6 (0.0-19.2)	22000 (17600-27400)	4.1 (3.3-5.1)	522000 (413000-663000)	97.3 (76.9-123.7)	899 (0-2330)	0.2 (0.0-0.4)	20900 (0-54400)	3.9 (0.0-10.1)
Streptococcus pneumoniae	Penicillin	787 (502-1160)	106.4 (67.8-156.3)	70100 (44600-103000)	9469.7 (6034.8-13902.2)	53 (26-96)	7.1 (3.5-13.0)	4680 (2280-8520)	633.2 (308.7-1152.3)	1800 (1290-2440)	20.3 (14.5-27.5)	159000 (114000-216000)	1798.2 (1282.5-2430.7)	121 (63-200)	1.4 (0.7-2.3)	10700 (5530-17700)	121.1 (62.4-199.4)	636 (461-845)	1.7 (1.2-2.2)	55100 (40000-73100)	143.3 (104.0-190.1)	40 (21-68)	0.1 (0.1-0.2)	3470 (1850-5880)	9.0 (4.8-15.3)	15800 (12300-20200)	2.9 (2.3-3.8)	386000 (298000-503000)	71.9 (55.6-93.8)	1110 (567-1870)	0.2 (0.1-0.3)	27000 (14000-45000)	5.0 (2.6-8.4)
Streptococcus pneumoniae	Third-generation cephalosporins	238 (149-357)	32.1 (20.1-48.3)	21100 (13200-31800)	2859.1 (1788.0-4297.6)	8 (3-17)	1.1 (0.4-2.3)	725 (268-1490)	98.0 (36.2-200.8)	553 (382-773)	6.2 (4.3-8.7)	48900 (33800-68400)	552.1 (380.8-771.3)	19 (7-37)	0.2 (0.1-0.4)	1700 (653-3290)	19.1 (7.4-37.1)	219 (153-304)	0.6 (0.4-0.8)	19000 (13300-26400)	49.3 (34.5-68.5)	8 (3-16)	0.0 (0.0-0.0)	684 (261-1360)	1.8 (0.7-3.5)	4490 (3390-5910)	0.8 (0.6-1.1)	108000 (79900-144000)	20.1 (14.9-26.9)	156 (63-290)	0.0 (0.0-0.1)	3630 (1440-6800)	0.7 (0.3-1.3)
Streptococcus pneumoniae	Trimethoprim-Sulfamethoxazole	1200 (787-1730)	162.6 (106.4-234.1)	107000 (70100-154000)	14467.5 (9470.0-20838.4)	101 (15-212)	13.7 (2.0-28.7)	9020 (1300-18900)	1219.7 (176.1-2552.8)	2820 (2050-3710)	31.8 (23.1-41.8)	249000 (181000-328000)	2812.5 (2040.4-3699.3)	240 (34-495)	2.7 (0.4-5.6)	21200 (2970-43800)	239.5 (33.5-493.7)	1150 (854-1480)	3.0 (2.2-3.8)	99300 (74000-128000)	258.2 (192.5-331.7)	99 (14-194)	0.3 (0.0-0.5)	8500 (1210-16800)	22.1 (3.1-43.6)	21100 (17000-26500)	3.9 (3.2-4.9)	516000 (407000-653000)	96.2 (76.0-121.8)	1760 (252-3500)	0.3 (0.0-0.7)	42700 (6330-85200)	8.0 (1.2-15.9)
Streptococcus pneumoniae	Resistance to one or more antibiotics	1670 (1090-2400)	225.5 (147.5-324.4)	148000 (97100-213000)	20065.9 (13120.0-28857.3)	341 (204-523)	46.2 (27.6-70.6)	30400 (18200-46500)	4105.0 (2457.8-6282.5)	3910 (2870-5120)	44.2 (32.3-57.8)	346000 (254000-453000)	3906.9 (2859.5-5111.5)	793 (521-1130)	8.9 (5.9-12.8)	70100 (46100-100000)	790.7 (519.5-1130.7)	1510 (1130-1930)	3.9 (2.9-5.0)	131000 (97900-167000)	339.9 (254.6-434.0)	305 (198-429)	0.8 (0.5-1.1)	26400 (17300-37000)	68.6 (45.0-96.3)	31700 (25600-39300)	5.9 (4.8-7.3)	768000 (612000-968000)	143.2 (114.2-180.5)	6420 (4440-8780)	1.2 (0.8-1.6)	156000 (106000-213000)	29.0 (19.8-39.8)

Table S17. Deaths and DALYs (in counts and age-specific rates) associated with and attributable to bacterial antimicrobial resistance by pathogen–drug combination, by age group, 2019, north Africa and the Middle East

Pathogen	Antibiotic class	Neonatal							
		Associated with resistance				Attributable to resistance			
		Deaths		DALYs		Deaths		DALYs	
		Counts	Rate per 100 000	Counts	Rate per 100 000	Counts	Rate per 100 000	Counts	Rate per 100 000
All pathogens	Resistance to one or more antibiotics	29200 (19700-40700)	3172.7 (2142.4-4426.7)	2600000 (1750000-3620000)	282346.5 (190551.1-393829.1)	7230 (4770-10200)	786.0 (518.2-1107.1)	643000 (424000-907000)	69926.7 (46089.3-98543.4)
Acinetobacter baumannii	Aminoglycosides	1010 (616-1550)	110.0 (67.0-168.8)	90000 (54800-138000)	9784.4 (5954.3-15008.3)	42 (0-106)	4.6 (0.0-11.5)	3780 (6-9440)	410.5 (0.7-1025.9)
Acinetobacter baumannii	Anti-pseudomonal penicillin/Beta-Lactamase inhibitors	1330 (820-2030)	145.1 (89.1-220.6)	119000 (72900-180000)	12900.8 (7923.4-19614.0)	50 (25-88)	5.4 (2.7-9.6)	4440 (2240-7830)	482.6 (243.6-850.5)
Acinetobacter baumannii	Beta Lactam/Beta-lactamase inhibitors	1230 (757-1880)	133.9 (82.3-204.7)	110000 (67300-167000)	11904.7 (7318.9-18202.8)	1 (0-2)	0.1 (0.0-0.2)	61 (13-146)	6.6 (1.4-15.8)
Acinetobacter baumannii	Carbapenem	1090 (668-1670)	118.8 (72.6-181.9)	97200 (59400-149000)	10562.8 (6456.9-16174.6)	177 (91-303)	19.3 (9.8-33.0)	15800 (8050-26900)	1712.5 (874.8-2929.1)
Acinetobacter baumannii	Fluoroquinolones	1250 (763-1890)	135.9 (83.0-205.8)	111000 (67900-168000)	12080.8 (7377.6-18296.3)	159 (79-286)	17.3 (8.5-31.1)	14100 (6990-25400)	1534.9 (759.7-2762.3)
Acinetobacter baumannii	Fourth-generation cephalosporins	1300 (792-1980)	141.0 (86.1-215.3)	115000 (70400-176000)	12541.7 (7650.2-19140.2)	2 (1-4)	0.2 (0.1-0.4)	136 (48-342)	14.8 (5.2-37.2)
Acinetobacter baumannii	Third-generation cephalosporins	1270 (784-1940)	138.1 (85.2-210.8)	113000 (69700-172000)	12283.4 (7573.8-18744.4)	4 (1-8)	0.4 (0.1-0.8)	327 (111-680)	35.5 (12.1-73.9)
Acinetobacter baumannii	Resistance to one or more antibiotics	1370 (841-2080)	148.4 (91.4-225.9)	121000 (74800-185000)	13196.1 (8131.1-20085.6)	434 (254-683)	47.2 (27.6-74.3)	38600 (22600-60700)	4197.5 (2451.1-6602.2)
Citrobacter spp.	Aminoglycosides	24 (12-41)	2.6 (1.3-4.5)	2100 (1100-3680)	227.7 (119.5-400.5)	1 (0-3)	0.1 (0.0-0.4)	110 (0-289)	11.9 (0.0-31.4)
Citrobacter spp.	Anti-pseudomonal penicillin/Beta-Lactamase inhibitors	65 (34-114)	7.0 (3.7-12.3)	5760 (3000-10100)	626.4 (326.4-1097.7)	11 (5-21)	1.2 (0.5-2.3)	969 (408-1870)	105.3 (44.4-202.8)
Citrobacter spp.	Carbapenem	38 (19-66)	4.1 (2.1-7.2)	3350 (1710-5920)	363.9 (186.3-642.9)	9 (4-17)	0.9 (0.4-1.9)	765 (334-1520)	83.1 (36.3-164.8)
Citrobacter spp.	Fluoroquinolones	91 (49-160)	9.9 (5.4-17.4)	8130 (4380-14300)	883.3 (476.5-1549.7)	14 (6-29)	1.6 (0.6-3.2)	1280 (499-2600)	139.4 (54.2-282.2)
Citrobacter spp.	Fourth-generation cephalosporins	65 (34-116)	7.1 (3.7-12.6)	5820 (3060-10300)	632.3 (332.5-1116.8)	5 (2-10)	0.6 (0.2-1.1)	452 (163-919)	49.1 (17.7-99.8)
Citrobacter spp.	Third-generation cephalosporins	114 (58-199)	12.4 (6.3-21.7)	10200 (5160-17700)	1104.6 (560.4-1927.9)	8 (3-18)	0.9 (0.3-1.9)	729 (255-1580)	79.3 (27.7-172.0)
Citrobacter spp.	Resistance to one or more antibiotics	168 (89-290)	18.2 (9.7-31.6)	14900 (7920-25900)	1620.2 (861.1-2809.7)	48 (24-87)	5.3 (2.6-9.5)	4310 (2170-7760)	468.2 (235.4-843.2)
Enterobacter spp.	Aminoglycosides	309 (192-466)	33.6 (20.8-50.7)	27500 (17100-41500)	2987.0 (1853.3-4505.5)	20 (5-39)	2.1 (0.6-4.3)	1750 (458-3500)	189.7 (49.8-380.0)
Enterobacter spp.	Anti-pseudomonal penicillin/Beta-Lactamase inhibitors	780 (486-1180)	84.8 (52.8-128.2)	69400 (43200-105000)	7542.7 (4696.1-11396.0)	91 (49-150)	9.9 (5.3-16.3)	8060 (4350-13400)	876.3 (472.3-1451.5)
Enterobacter spp.	Carbapenem	343 (215-517)	37.3 (23.4-56.2)	30500 (19100-46000)	3318.6 (2078.3-4996.8)	84 (49-136)	9.2 (5.3-14.8)	7500 (4330-12100)	815.6 (470.3-1313.5)
Enterobacter spp.	Fluoroquinolones	467 (283-707)	50.8 (30.7-76.8)	41500 (25100-62800)	4513.7 (2731.6-6829.1)	51 (24-92)	5.6 (2.7-10.0)	4550 (2170-8200)	494.9 (236.1-891.1)
Enterobacter spp.	Fourth-generation cephalosporins	700 (444-1030)	76.1 (48.3-112.3)	62300 (39500-91900)	6771.3 (4293.9-9991.2)	34 (16-62)	3.7 (1.7-6.7)	3040 (1440-5500)	330.8 (156.0-597.5)
Enterobacter spp.	Trimethoprim-Sulfamethoxazole	614 (378-939)	66.7 (41.0-102.0)	54600 (33600-83500)	5933.9 (3650.6-9073.2)	34 (0-75)	3.7 (0.0-8.2)	3050 (0-6680)	331.8 (0.0-726.2)
Enterobacter spp.	Resistance to one or more antibiotics	1330 (835-1980)	144.2 (90.7-215.1)	118000 (74300-176000)	12820.4 (8071.4-19132.4)	314 (193-470)	34.2 (21.0-51.1)	28000 (17200-41800)	3039.1 (1867.6-4538.7)
Enterococcus faecalis	Fluoroquinolones	262 (148-425)	28.5 (16.1-46.2)	23300 (13200-37800)	2533.4 (1431.7-4108.5)	65 (28-121)	7.1 (3.1-13.2)	5790 (2510-10800)	628.9 (272.4-1172.7)
Enterococcus faecalis	Vancomycin	41 (23-67)	4.5 (2.5-7.3)	3660 (2090-5950)	397.9 (226.7-646.4)	14 (6-27)	1.5 (0.6-2.9)	1220 (522-2370)	132.5 (56.8-257.5)
Enterococcus faecalis	Resistance to one or more antibiotics	284 (163-457)	30.9 (17.7-49.7)	25300 (14500-40700)	2748.6 (1580.2-4420.8)	79 (38-141)	8.6 (4.1-15.4)	7010 (3390-12600)	761.4 (368.2-1364.2)
Enterococcus faecium	Fluoroquinolones	439 (261-701)	47.7 (28.4-76.2)	39100 (23300-62400)	4246.6 (2528.1-6778.5)	75 (21-144)	8.1 (2.3-15.6)	6650 (1900-12800)	722.7 (206.3-1391.0)
Enterococcus faecium	Vancomycin	223 (133-359)	24.3 (14.5-39.1)	19900 (11900-32100)	2162.7 (1288.6-3483.8)	51 (24-96)	5.6 (2.6-10.4)	4570 (2170-8530)	497.2 (235.3-927.3)
Enterococcus faecium	Resistance to one or more antibiotics	442 (263-706)	48.1 (28.6-76.7)	39400 (23400-62800)	4278.4 (2547.6-6828.0)	126 (69-212)	13.7 (7.5-23.0)	11200 (6180-18900)	1219.9 (671.4-2050.6)
Other enterococci	Fluoroquinolones	452 (243-754)	49.2 (26.4-81.9)	40200 (21600-67000)	4371.2 (2342.4-7280.6)	81 (12-175)	8.8 (1.3-19.1)	7240 (1040-15600)	786.5 (113.3-1694.0)
Other enterococci	Vancomycin	113 (62-191)	12.3 (6.7-20.7)	10000 (5480-17000)	1091.1 (596.1-1842.5)	19 (9-38)	2.1 (1.0-4.2)	1730 (780-3410)	187.8 (84.8-370.3)
Other enterococci	Resistance to one or more antibiotics	456 (244-759)	49.6 (26.6-82.5)	40600 (21700-67500)	4408.3 (2361.1-7336.9)	101 (38-196)	11.0 (4.1-21.3)	8960 (3380-17400)	974.2 (366.9-1890.8)
Escherichia coli	Aminoglycosides	816 (529-1180)	88.7 (57.5-127.8)	72700 (47200-105000)	7898.4 (5129.1-11372.2)	47 (28-73)	5.2 (3.0-7.9)	4220 (2490-6460)	459.0 (270.3-702.0)
Escherichia coli	Aminopenicillin	4360 (2860-6210)	473.8 (310.9-675.2)	388000 (255000-554000)	42204.5 (27710.0-60200.9)	50 (29-76)	5.4 (3.2-8.3)	4440 (2630-6810)	482.1 (285.9-740.2)
Escherichia coli	Beta Lactam/Beta-lactamase inhibitors	3120 (2050-4480)	339.3 (222.7-486.9)	278000 (183000-399000)	30231.3 (19837.0-43396.6)	77 (47-117)	8.4 (5.1-12.7)	6870 (4180-10400)	746.5 (454.5-1134.0)
Escherichia coli	Carbapenem	763 (483-1130)	82.9 (52.5-123.1)	68000 (43000-101000)	7386.1 (4672.0-10966.2)	161 (85-271)	17.5 (9.3-29.5)	14300 (7600-24200)	1559.6 (825.8-2627.6)
Escherichia coli	Fluoroquinolones	2970 (1960-4240)	323.0 (213.0-460.6)	265000 (174000-377000)	28776.1 (18965.1-40984.8)	301 (192-441)	32.7 (20.8-47.9)	26800 (17100-39300)	2911.9 (1858.1-4266.6)
Escherichia coli	Third-generation cephalosporins	3370 (2220-4780)	366.3 (241.3-519.9)	300000 (198000-426000)	32631.0 (21480.8-46292.2)	448 (192-810)	48.7 (20.9-88.0)	39900 (17200-72000)	4339.2 (1870.4-7823.6)
Escherichia coli	Trimethoprim-Sulfamethoxazole	3300 (2180-4670)	358.4 (237.2-507.8)	294000 (194000-416000)	31923.4 (21113.7-45223.2)	181 (112-273)	19.7 (12.1-29.6)	16100 (9910-24200)	1750.4 (1076.9-2634.6)
Escherichia coli	Resistance to one or more antibiotics	4610 (3010-6580)	500.6 (327.3-715.4)	410000 (268000-586000)	44596.8 (29152.0-63743.0)	1270 (811-1910)	137.6 (88.1-207.9)	113000 (72200-170000)	12248.6 (7844.5-18515.1)
Group A *Streptococcus*	Macrolide	52 (31-85)	5.6 (3.4-9.2)	4580 (2770-7540)	498.1 (301.0-820.0)	5 (0-17)	0.5 (0.0-1.8)	439 (0-1510)	47.7 (0.0-163.9)
Group A *Streptococcus*	Resistance to one or more antibiotics	52 (31-85)	5.6 (3.4-9.2)	4580 (2770-7540)	498.1 (301.0-820.0)	5 (0-17)	0.5 (0.0-1.8)	439 (0-1510)	47.7 (0.0-163.9)
Group B *Streptococcus*	Fluoroquinolones	864 (540-1290)	93.9 (58.7-140.3)	76800 (48000-115000)	8351.2 (5218.4-12488.0)	135 (21-332)	14.6 (2.3-36.1)	12000 (1910-29500)	1301.8 (207.6-3209.5)
Group B *Streptococcus*	Macrolide	3280 (2120-4780)	357.0 (230.9-519.5)	292000 (189000-426000)	31770.0 (20556.0-46252.3)	321 (0-903)	34.9 (0.0-98.1)	28600 (0-80400)	3108.3 (0.0-8733.5)
Group B *Streptococcus*	Penicillin	54 (32-83)	5.9 (3.5-9.0)	4820 (2890-7340)	524.2 (313.8-798.2)	12 (2-28)	1.3 (0.2-3.0)	1100 (199-2480)	119.7 (21.7-269.2)
Group B *Streptococcus*	Resistance to one or more antibiotics	3480 (2240-5040)	378.6 (243.3-547.8)	310000 (199000-448000)	33693.5 (21644.1-48741.4)	469 (21-1050)	50.9 (2.2-113.9)	41700 (1810-93200)	4529.7 (197.2-10134.2)
Haemophilus influenzae	Aminopenicillin	247 (146-376)	26.8 (15.8-40.8)	22000 (13000-33500)	2391.1 (1410.3-3639.0)	38 (3-85)	4.1 (0.3-9.2)	3370 (264-7540)	366.5 (28.7-819.3)
Haemophilus influenzae	Third-generation cephalosporins	102 (59-160)	11.1 (6.4-17.4)	9100 (5250-14200)	988.6 (571.2-1548.0)	30 (12-55)	3.2 (1.3-6.0)	2640 (1060-4930)	286.8 (114.7-536.1)
Haemophilus influenzae	Resistance to one or more antibiotics	303 (181-458)	33.0 (19.7-49.8)	27100 (16200-40900)	2940.5 (1761.0-4441.9)	67 (24-124)	7.3 (2.6-13.5)	6010 (2170-11000)	653.4 (236.1-1200.1)
Klebsiella pneumoniae	Aminoglycosides	2420 (1560-3540)	263.4 (169.7-384.7)	216000 (139000-315000)	23432.8 (15094.6-34229.3)	189 (108-294)	20.5 (11.8-31.9)	16800 (9620-26100)	1826.4 (1046.0-2840.3)
Klebsiella pneumoniae	Beta Lactam/Beta-lactamase inhibitors	4820 (3210-6830)	524.3 (348.7-741.8)	429000 (285000-607000)	46634.4 (31014.3-65993.3)	53 (14-130)	5.8 (1.5-14.1)	4710 (1230-11500)	511.8 (133.9-1253.6)
Klebsiella pneumoniae	Carbapenem	1110 (535-1980)	120.3 (58.1-215.0)	98400 (47600-176000)	10698.6 (5168.9-19114.4)	261 (124-467)	28.4 (13.5-50.8)	23200 (11000-41500)	2523.7 (1200.5-4514.8)
Klebsiella pneumoniae	Fluoroquinolones	2950 (1910-4170)	320.4 (207.2-453.7)	262000 (170000-371000)	28501.1 (18430.5-40373.7)	204 (114-321)	22.2 (12.4-34.8)	18200 (10200-28500)	1975.2 (1105.0-3101.4)
Klebsiella pneumoniae	Third-generation cephalosporins	5010 (3250-7120)	544.6 (352.8-773.3)	446000 (289000-633000)	48443.2 (31384.3-68793.2)	697 (269-1330)	75.8 (29.2-144.5)	62000 (24000-118000)	6739.5 (2603.5-12849.7)
Klebsiella pneumoniae	Trimethoprim-Sulfamethoxazole	4230 (2790-5980)	459.3 (303.7-649.7)	376000 (249000-532000)	40853.6 (27014.8-57809.0)	211 (99-368)	23.0 (10.8-40.0)	18800 (8800-32700)	2042.4 (956.2-3552.4)
Klebsiella pneumoniae	Resistance to one or more antibiotics	5600 (3700-7890)	608.6 (401.6-857.2)	498000 (329000-702000)	54131.1 (35724.5-76253.5)	1620 (949-2480)	175.6 (103.1-269.0)	144000 (84400-220000)	15619.1 (9171.2-23922.5)
Morganella spp.	Fluoroquinolones	1 (0-3)	0.1 (0.0-0.3)	118 (30-255)	12.8 (3.2-27.7)	0 (0-1)	0.0 (0.0-0.1)	19 (4-46)	2.1 (0.4-5.0)
Morganella spp.	Fourth-generation cephalosporins	1 (0-2)	0.1 (0.0-0.2)	81 (21-182)	8.8 (2.3-19.8)	0 (0-0)	0.0 (0.0-0.0)	13 (3-37)	1.5 (0.3-4.0)
Morganella spp.	Third-generation cephalosporins	1 (0-1)	0.1 (0.0-0.2)	58 (14-132)	6.3 (1.5-14.3)	0 (0-0)	0.0 (0.0-0.0)	4 (1-10)	0.4 (0.1-1.1)
Morganella spp.	Resistance to one or more antibiotics	2 (0-4)	0.2 (0.0-0.4)	148 (38-330)	16.1 (4.1-35.8)	0 (0-1)	0.0 (0.0-0.1)	36 (8-85)	3.9 (0.9-9.3)
Mycobacterium tuberculosis	Extensive drug resistance in TB	--	--	0 (0-0)	0.0 (0.0-0.0)	--	--	0 (0-0)	0.0 (0.0-0.0)
Mycobacterium tuberculosis	Isoniazid mono-resistance	--	--	0 (0-0)	0.0 (0.0-0.0)	--	--	0 (0-0)	0.0 (0.0-0.0)
Mycobacterium tuberculosis	Multi-drug resistance excluding extensive drug resistance in TB	--	--	0 (0-0)	0.0 (0.0-0.0)	--	--	0 (0-0)	0.0 (0.0-0.0)
Mycobacterium tuberculosis	Rifampicin mono-resistance	--	--	0 (0-0)	0.0 (0.0-0.0)	--	--	0 (0-0)	0.0 (0.0-0.0)
Mycobacterium tuberculosis	Resistance to one or more antibiotics	--	--	0 (0-0)	0.0 (0.0-0.0)	--	--	0 (0-0)	0.0 (0.0-0.0)
Neisseria gonorrhoeae	Fluoroquinolones	--	--	--	--	--	--	--	--
Neisseria gonorrhoeae	Third-generation cephalosporins	--	--	--	--	--	--	--	--
Neisseria gonorrhoeae	Resistance to one or more antibiotics	--	--	--	--	--	--	--	--
Proteus spp.	Aminoglycosides	51 (29-82)	5.5 (3.2-8.9)	4540 (2620-7320)	493.2 (284.9-795.4)	2 (0-5)	0.2 (0.0-0.6)	201 (23-460)	21.9 (2.5-50.0)
Proteus spp.	Aminopenicillin	137 (79-221)	14.9 (8.6-24.0)	12300 (7020-19700)	1331.8 (763.2-2142.7)	2 (0-7)	0.2 (0.0-0.7)	189 (0-586)	20.5 (0.0-63.7)
Proteus spp.	Fluoroquinolones	69 (40-111)	7.5 (4.3-12.1)	6160 (3540-9910)	669.5 (384.5-1076.8)	4 (2-9)	0.5 (0.2-1.0)	393 (147-781)	42.7 (16.0-84.9)
Proteus spp.	Third-generation cephalosporins	60 (34-98)	6.5 (3.7-10.7)	5360 (3030-8750)	582.8 (329.4-950.7)	12 (3-25)	1.3 (0.4-2.7)	1030 (294-2190)	112.4 (32.0-238.2)
Proteus spp.	Trimethoprim-Sulfamethoxazole	106 (61-171)	11.5 (6.6-18.6)	9460 (5450-15200)	1028.6 (592.0-1651.3)	4 (0-9)	0.4 (0.0-0.9)	329 (0-762)	35.8 (0.0-82.8)
Proteus spp.	Resistance to one or more antibiotics	148 (85-236)	16.1 (9.2-25.6)	13200 (7550-21000)	1432.9 (820.4-2283.3)	24 (12-44)	2.6 (1.3-4.7)	2150 (1030-3880)	233.2 (112.1-422.1)
Pseudomonas aeruginosa	Aminoglycosides	984 (631-1410)	107.0 (68.6-153.5)	87600 (56200-126000)	9522.1 (6105.7-13659.7)	26 (0-64)	2.9 (0.0-7.0)	2360 (0-5740)	256.2 (0.0-623.5)
Pseudomonas aeruginosa	Anti-pseudomonal penicillin/Beta-Lactamase inhibitors	1110 (718-1560)	120.2 (78.0-169.3)	98400 (63900-139000)	10698.7 (6942.1-15066.0)	83 (48-134)	9.0 (5.2-14.6)	7350 (4270-11900)	798.5 (463.9-1294.2)
Pseudomonas aeruginosa	Carbapenem	1410 (911-1980)	152.7 (99.0-215.3)	125000 (81100-176000)	13593.6 (8810.8-19165.5)	252 (153-387)	27.4 (16.6-42.1)	22500 (13600-34400)	2441.9 (1479.2-3743.8)
Pseudomonas aeruginosa	Fluoroquinolones	1270 (819-1810)	137.8 (89.0-196.5)	113000 (72900-161000)	12267.7 (7920.0-17512.2)	134 (78-209)	14.6 (8.5-22.7)	11900 (6990-18700)	1298.8 (760.0-2029.8)
Pseudomonas aeruginosa	Fourth-generation cephalosporins	1230 (790-1790)	134.2 (85.8-195.0)	110000 (70300-160000)	11939.9 (7638.0-17375.7)	43 (22-74)	4.7 (2.4-8.1)	3810 (1960-6620)	414.3 (212.5-720.0)
Pseudomonas aeruginosa	Third-generation cephalosporins	1810 (1170-2600)	196.4 (126.8-283.1)	161000 (104000-232000)	17481.1 (11282.4-25194.7)	117 (50-208)	12.7 (5.4-22.6)	10400 (4440-18500)	1132.8 (482.7-2010.3)
Pseudomonas aeruginosa	Resistance to one or more antibiotics	2560 (1670-3630)	278.7 (181.3-394.4)	228000 (148000-323000)	24801.4 (16134.0-35105.8)	656 (385-1020)	71.2 (41.9-111.3)	58400 (34300-91100)	6342.5 (3726.9-9904.6)
Salmonella Paratyphi	Fluoroquinolones	--	--	0 (0-0)	0.0 (0.0-0.0)	--	--	0 (0-0)	0.0 (0.0-0.0)
Salmonella Paratyphi	Multi-drug resistance in Salmonella Typhi and Paratyphi	--	--	0 (0-0)	0.0 (0.0-0.0)	--	--	0 (0-0)	0.0 (0.0-0.0)
Salmonella Paratyphi	Resistance to one or more antibiotics	--	--	0 (0-0)	0.0 (0.0-0.0)	--	--	0 (0-0)	0.0 (0.0-0.0)
Salmonella Typhi	Fluoroquinolones	467 (137-1040)	50.8 (14.9-113.6)	41500 (12200-92900)	4515.4 (1324.8-10093.3)	95 (10-272)	10.3 (1.1-29.6)	8450 (902-24200)	918.0 (98.0-2631.3)
Salmonella Typhi	Multi-drug resistance in Salmonella Typhi and Paratyphi	179 (72-394)	19.5 (7.8-42.8)	15900 (6360-35000)	1731.0 (690.9-3801.0)	22 (0-71)	2.4 (0.0-7.7)	1950 (0-6310)	211.4 (0.0-685.5)
Salmonella Typhi	Resistance to one or more antibiotics	608 (236-1210)	66.1 (25.7-132.0)	54100 (21000-108000)	5877.0 (2281.3-11735.5)	117 (20-291)	12.7 (2.2-31.7)	10400 (1820-25900)	1129.4 (198.0-2814.5)
Non-typhoidal *Salmonella*	Fluoroquinolones	9 (4-17)	1.0 (0.4-1.9)	790 (363-1560)	85.9 (39.5-169.3)	2 (0-5)	0.2 (0.0-0.5)	164 (25-440)	17.8 (2.7-47.9)
Non-typhoidal *Salmonella*	Resistance to one or more antibiotics	9 (4-17)	1.0 (0.4-1.9)	790 (363-1560)	85.9 (39.5-169.3)	2 (0-5)	0.2 (0.0-0.5)	164 (25-440)	17.8 (2.7-47.9)
Serratia spp.	Aminoglycosides	101 (54-173)	11.0 (5.9-18.8)	9020 (4820-15300)	980.4 (523.5-1667.9)	6 (0-18)	0.7 (0.0-1.9)	542 (0-1590)	58.9 (0.0-172.5)
Serratia spp.	Anti-pseudomonal penicillin/Beta-Lactamase inhibitors	77 (42-132)	8.4 (4.6-14.4)	6880 (3750-11700)	748.0 (407.4-1276.6)	15 (7-30)	1.7 (0.7-3.3)	1350 (605-2680)	147.0 (65.8-291.0)
Serratia spp.	Carbapenem	64 (36-104)	6.9 (3.9-11.3)	5670 (3210-9200)	616.6 (348.4-1000.2)	17 (8-31)	1.9 (0.9-3.4)	1530 (720-2780)	166.2 (78.3-301.6)
Serratia spp.	Fluoroquinolones	68 (36-116)	7.4 (3.9-12.6)	6040 (3210-10300)	656.3 (349.1-1124.8)	7 (1-17)	0.8 (0.1-1.9)	653 (120-1530)	70.9 (13.0-166.8)
Serratia spp.	Fourth-generation cephalosporins	130 (71-215)	14.1 (7.7-23.3)	11600 (6280-19100)	1256.3 (682.8-2075.9)	13 (5-26)	1.4 (0.5-2.9)	1130 (431-2350)	123.3 (46.8-254.9)
Serratia spp.	Third-generation cephalosporins	163 (85-280)	17.7 (9.3-30.5)	14500 (7590-24900)	1572.7 (824.9-2709.9)	11 (3-25)	1.2 (0.3-2.7)	966 (282-2220)	105.0 (30.6-241.4)
Serratia spp.	Resistance to one or more antibiotics	282 (157-463)	30.7 (17.0-50.3)	25100 (13900-41100)	2727.4 (1513.6-4469.0)	69 (34-128)	7.5 (3.7-13.9)	6180 (3060-11300)	671.3 (333.1-1232.7)
Shigella spp.	Fluoroquinolones	35 (8-96)	3.8 (0.9-10.5)	3170 (741-8620)	345.0 (80.6-937.1)	7 (1-23)	0.8 (0.1-2.5)	649 (55-2070)	70.6 (6.0-225.1)
Shigella spp.	Resistance to one or more antibiotics	35 (8-96)	3.8 (0.9-10.5)	3170 (741-8620)	345.0 (80.6-937.1)	7 (1-23)	0.8 (0.1-2.5)	649 (55-2070)	70.6 (6.0-225.1)
Staphylococcus aureus	Fluoroquinolones	961 (611-1370)	104.4 (66.4-148.4)	85500 (54400-121000)	9291.0 (5909.6-13199.4)	42 (17-74)	4.6 (1.8-8.0)	3750 (1490-6580)	408.0 (162.3-715.5)

Pathogen	Antibiotic class	Post Neonatal							
		Associated with resistance				Attributable to resistance			
		Deaths		DALYs		Deaths		DALYs	
		Counts	Rate per 100 000	Counts	Rate per 100 000	Counts	Rate per 100 000	Counts	Rate per 100 000
All pathogens	Resistance to one or more antibiotics	18900 (14400-24400)	172.1 (131.2-222.3)	1680000 (1280000-2170000)	15257.4 (11627.7-19717.5)	4750 (3520-6290)	43.2 (32.0-57.2)	421000 (312000-557000)	3827.1 (2840.2-5070.0)
Acinetobacter baumannii	Aminoglycosides	489 (283-760)	4.4 (2.6-6.9)	43200 (25100-67200)	393.4 (228.0-611.7)	20 (0-51)	0.2 (0.0-0.5)	1810 (2-4500)	16.4 (0.0-40.9)
Acinetobacter baumannii	Anti-pseudomonal penicillin/Beta-Lactamase inhibitors	652 (383-996)	5.9 (3.5-9.1)	57600 (33900-88100)	524.3 (308.1-801.0)	25 (12-44)	0.2 (0.1-0.4)	2200 (1100-3880)	20.0 (10.0-35.3)
Acinetobacter baumannii	Beta Lactam/Beta-lactamase inhibitors	599 (353-924)	5.5 (3.2-8.4)	53000 (31200-81700)	482.3 (283.6-743.5)	0 (0-1)	0.0 (0.0-0.0)	29 (6-72)	0.3 (0.1-0.7)
Acinetobacter baumannii	Carbapenem	533 (310-819)	4.8 (2.8-7.5)	47100 (27400-72500)	428.6 (249.1-659.4)	86 (44-151)	0.8 (0.4-1.4)	7640 (3910-13400)	69.5 (35.5-121.6)
Acinetobacter baumannii	Fluoroquinolones	608 (353-931)	5.5 (3.2-8.5)	53800 (31300-82300)	489.4 (284.3-748.9)	77 (38-133)	0.7 (0.3-1.2)	6840 (3370-11800)	62.2 (30.7-107.2)
Acinetobacter baumannii	Fourth-generation cephalosporins	632 (370-972)	5.7 (3.4-8.8)	55900 (32700-86000)	508.4 (297.5-782.2)	1 (0-2)	0.0 (0.0-0.0)	60 (21-157)	0.5 (0.2-1.4)
Acinetobacter baumannii	Third-generation cephalosporins	621 (364-952)	5.7 (3.3-8.7)	55000 (32200-84200)	499.9 (292.8-766.0)	2 (1-4)	0.0 (0.0-0.0)	168 (57-362)	1.5 (0.5-3.3)
Acinetobacter baumannii	Resistance to one or more antibiotics	667 (393-1020)	6.1 (3.6-9.3)	59000 (34700-90500)	536.5 (315.9-823.4)	212 (122-335)	1.9 (1.1-3.1)	18700 (10800-29700)	170.5 (98.1-269.8)
Citrobacter spp.	Aminoglycosides	18 (9-32)	0.2 (0.1-0.3)	1570 (767-2850)	14.3 (7.0-25.9)	1 (0-2)	0.0 (0.0-0.0)	82 (0-219)	0.7 (0.0-2.0)
Citrobacter spp.	Anti-pseudomonal penicillin/Beta-Lactamase inhibitors	51 (25-94)	0.5 (0.2-0.9)	4520 (2170-8350)	41.1 (19.8-75.9)	9 (3-17)	0.1 (0.0-0.2)	770 (309-1540)	7.0 (2.8-14.0)
Citrobacter spp.	Carbapenem	30 (15-56)	0.3 (0.1-0.5)	2690 (1310-4920)	24.5 (12.0-44.8)	7 (3-14)	0.1 (0.0-0.1)	610 (250-1260)	5.5 (2.3-11.4)
Citrobacter spp.	Fluoroquinolones	82 (41-148)	0.7 (0.4-1.3)	7250 (3620-13100)	65.9 (32.9-119.2)	13 (5-27)	0.1 (0.0-0.2)	1160 (453-2420)	10.5 (4.1-22.0)
Citrobacter spp.	Fourth-generation cephalosporins	55 (26-106)	0.5 (0.2-1.0)	4890 (2270-9340)	44.5 (20.7-85.0)	5 (2-10)	0.0 (0.0-0.1)	399 (134-851)	3.6 (1.2-7.7)
Citrobacter spp.	Third-generation cephalosporins	100 (50-179)	0.9 (0.5-1.6)	8810 (4430-15800)	80.1 (40.3-144.1)	8 (3-16)	0.1 (0.0-0.1)	698 (255-1440)	6.4 (2.3-13.1)
Citrobacter spp.	Resistance to one or more antibiotics	146 (72-265)	1.3 (0.7-2.4)	12900 (6390-23500)	117.4 (58.1-213.5)	42 (20-80)	0.4 (0.2-0.7)	3720 (1790-7100)	33.8 (16.3-64.6)
Enterobacter spp.	Aminoglycosides	87 (56-127)	0.8 (0.5-1.2)	7720 (4940-11200)	70.2 (44.9-101.9)	6 (2-11)	0.1 (0.0-0.1)	490 (138-968)	4.5 (1.3-8.8)
Enterobacter spp.	Anti-pseudomonal penicillin/Beta-Lactamase inhibitors	201 (131-293)	1.8 (1.2-2.7)	17800 (11600-25900)	161.9 (105.1-235.6)	23 (12-38)	0.2 (0.1-0.3)	2050 (1090-3330)	18.6 (10.0-30.3)
Enterobacter spp.	Carbapenem	89 (57-133)	0.8 (0.5-1.2)	7890 (5030-11700)	71.7 (45.8-106.7)	22 (13-35)	0.2 (0.1-0.3)	1920 (1110-3070)	17.5 (10.1-27.9)
Enterobacter spp.	Fluoroquinolones	121 (75-184)	1.1 (0.7-1.7)	10700 (6630-16300)	97.0 (60.3-148.4)	13 (6-23)	0.1 (0.1-0.2)	1140 (564-2030)	10.4 (5.1-18.5)
Enterobacter spp.	Fourth-generation cephalosporins	190 (126-282)	1.7 (1.1-2.6)	16800 (11200-24900)	153.2 (101.5-226.7)	10 (5-17)	0.1 (0.0-0.2)	855 (398-1540)	7.8 (3.6-14.0)
Enterobacter spp.	Trimethoprim-Sulfamethoxazole	163 (101-244)	1.5 (0.9-2.2)	14400 (8980-21600)	131.2 (81.6-196.7)	9 (0-20)	0.1 (0.0-0.2)	800 (0-1750)	7.3 (0.0-15.9)
Enterobacter spp.	Resistance to one or more antibiotics	345 (230-500)	3.1 (2.1-4.6)	30600 (20300-44300)	278.2 (185.0-402.8)	82 (52-124)	0.7 (0.5-1.1)	7260 (4600-11000)	66.0 (41.9-99.6)
Enterococcus faecalis	Fluoroquinolones	50 (27-82)	0.5 (0.2-0.7)	4430 (2400-7340)	40.3 (21.8-66.7)	12 (5-23)	0.1 (0.0-0.2)	1090 (460-2020)	9.9 (4.2-18.3)
Enterococcus faecalis	Vancomycin	8 (4-14)	0.1 (0.0-0.1)	728 (389-1270)	6.6 (3.5-11.6)	3 (1-6)	0.0 (0.0-0.1)	244 (102-492)	2.2 (0.9-4.5)
Enterococcus faecalis	Resistance to one or more antibiotics	54 (30-90)	0.5 (0.3-0.8)	4830 (2640-7990)	43.9 (24.0-72.7)	15 (7-27)	0.1 (0.1-0.2)	1340 (636-2350)	12.2 (5.8-21.4)
Enterococcus faecium	Fluoroquinolones	183 (99-300)	1.7 (0.9-2.7)	16200 (8810-26600)	147.2 (80.2-241.8)	31 (9-64)	0.3 (0.1-0.6)	2750 (776-5680)	25.0 (7.1-51.7)
Enterococcus faecium	Vancomycin	94 (51-158)	0.9 (0.5-1.4)	8350 (4500-14000)	75.9 (40.9-127.3)	22 (10-42)	0.2 (0.1-0.4)	1920 (846-3690)	17.5 (7.7-33.5)
Enterococcus faecium	Resistance to one or more antibiotics	184 (101-302)	1.7 (0.9-2.7)	16300 (8930-26800)	148.3 (81.2-243.4)	53 (27-96)	0.5 (0.2-0.9)	4670 (2380-8520)	42.5 (21.7-77.5)
Other enterococci	Fluoroquinolones	134 (67-247)	1.2 (0.6-2.2)	11900 (5950-21800)	108.0 (54.1-198.7)	24 (3-53)	0.2 (0.0-0.5)	2120 (310-4690)	19.3 (2.8-42.7)
Other enterococci	Vancomycin	36 (19-66)	0.3 (0.2-0.6)	3210 (1640-5880)	29.2 (14.9-53.5)	6 (3-13)	0.1 (0.0-0.1)	556 (237-1110)	5.1 (2.2-10.1)
Other enterococci	Resistance to one or more antibiotics	135 (68-249)	1.2 (0.6-2.3)	12000 (6020-22000)	109.2 (54.7-200.3)	30 (10-61)	0.3 (0.1-0.6)	2670 (918-5420)	24.3 (8.3-49.3)
Escherichia coli	Aminoglycosides	403 (278-580)	3.7 (2.5-5.3)	36200 (24900-52500)	329.1 (226.5-477.8)	23 (15-35)	0.2 (0.1-0.3)	2080 (1330-3120)	18.9 (12.1-28.4)
Escherichia coli	Aminopenicillin	2060 (1480-2820)	18.8 (13.5-25.6)	185000 (132000-253000)	1684.3 (1203.0-2302.0)	21 (14-31)	0.2 (0.1-0.3)	1910 (1230-2770)	17.4 (11.2-25.2)
Escherichia coli	Beta Lactam/Beta-lactamase inhibitors	1530 (1090-2100)	13.9 (9.9-19.1)	137000 (97700-189000)	1249.0 (888.2-1717.6)	35 (23-51)	0.3 (0.2-0.5)	3160 (2040-4580)	28.8 (18.6-41.7)
Escherichia coli	Carbapenem	367 (235-547)	3.3 (2.1-5.0)	33000 (21200-49100)	300.0 (192.9-446.4)	76 (41-125)	0.7 (0.4-1.1)	6800 (3680-11200)	61.8 (33.5-101.9)
Escherichia coli	Fluoroquinolones	1440 (1040-1960)	13.1 (9.4-17.8)	129000 (92800-176000)	1177.5 (843.9-1602.3)	146 (101-206)	1.3 (0.9-1.9)	13100 (9020-18500)	118.9 (82.0-168.6)
Escherichia coli	Third-generation cephalosporins	1610 (1150-2240)	14.7 (10.5-20.3)	145000 (103000-201000)	1316.9 (936.6-1828.4)	212 (94-373)	1.9 (0.9-3.4)	19000 (8550-33300)	172.4 (77.7-303.0)
Escherichia coli	Trimethoprim-Sulfamethoxazole	1580 (1120-2150)	14.4 (10.2-19.5)	142000 (100000-195000)	1288.2 (912.9-1774.4)	86 (56-124)	0.8 (0.5-1.1)	7640 (5000-11000)	69.5 (45.5-100.4)
Escherichia coli	Resistance to one or more antibiotics	2170 (1550-2960)	19.7 (14.1-26.9)	195000 (139000-267000)	1770.0 (1264.2-2424.2)	599 (396-894)	5.4 (3.6-8.1)	53600 (35500-79900)	487.6 (322.8-726.4)
Group A *Streptococcus*	Macrolide	44 (24-73)	0.4 (0.2-0.7)	3910 (2130-6510)	35.5 (19.4-59.3)	4 (0-14)	0.0 (0.0-0.1)	375 (0-1220)	3.4 (0.0-11.1)
Group A *Streptococcus*	Resistance to one or more antibiotics	44 (24-73)	0.4 (0.2-0.7)	3910 (2130-6510)	35.5 (19.4-59.3)	4 (0-14)	0.0 (0.0-0.1)	375 (0-1220)	3.4 (0.0-11.1)
Group B *Streptococcus*	Fluoroquinolones	185 (125-267)	1.7 (1.1-2.4)	16400 (11000-23600)	149.1 (100.4-214.6)	29 (5-66)	0.3 (0.0-0.6)	2540 (410-5830)	23.1 (3.7-53.0)
Group B *Streptococcus*	Macrolide	688 (492-959)	6.3 (4.5-8.7)	60900 (43600-84800)	553.6 (396.4-771.7)	67 (0-182)	0.6 (0.0-1.7)	5950 (0-16100)	54.1 (0.0-146.6)
Group B *Streptococcus*	Penicillin	11 (7-16)	0.1 (0.1-0.1)	981 (639-1460)	8.9 (5.8-13.3)	3 (1-6)	0.0 (0.0-0.1)	225 (44-506)	2.0 (0.4-4.6)
Group B *Streptococcus*	Resistance to one or more antibiotics	730 (521-1020)	6.6 (4.7-9.2)	64600 (46100-89900)	587.9 (419.3-817.5)	99 (5-210)	0.9 (0.0-1.9)	8720 (408-18600)	79.3 (3.7-169.3)
Haemophilus influenzae	Aminopenicillin	277 (194-378)	2.5 (1.8-3.4)	24500 (17100-33500)	222.9 (156.0-304.4)	43 (4-88)	0.4 (0.0-0.8)	3820 (337-7770)	34.8 (3.1-70.7)
Haemophilus influenzae	Third-generation cephalosporins	97 (68-133)	0.9 (0.6-1.2)	8580 (6040-11800)	78.1 (54.9-107.3)	28 (12-49)	0.3 (0.1-0.4)	2490 (1060-4370)	22.6 (9.7-39.8)
Haemophilus influenzae	Resistance to one or more antibiotics	327 (238-437)	3.0 (2.2-4.0)	28900 (21000-38700)	263.1 (191.4-352.2)	71 (27-123)	0.6 (0.2-1.1)	6310 (2380-10900)	57.4 (21.6-98.8)
Klebsiella pneumoniae	Aminoglycosides	1310 (949-1800)	12.0 (8.6-16.4)	116000 (84000-159000)	1058.1 (763.9-1447.0)	102 (64-153)	0.9 (0.6-1.4)	9030 (5700-13600)	82.1 (51.9-123.3)
Klebsiella pneumoniae	Beta Lactam/Beta-lactamase inhibitors	2490 (1790-3370)	22.6 (16.3-30.6)	220000 (159000-298000)	2001.5 (1442.1-2709.6)	26 (7-66)	0.2 (0.1-0.6)	2330 (593-5830)	21.2 (5.4-53.0)
Klebsiella pneumoniae	Carbapenem	672 (348-1210)	6.1 (3.2-11.0)	59400 (30800-107000)	540.4 (280.4-973.0)	158 (81-279)	1.4 (0.7-2.5)	14000 (7130-24700)	127.0 (64.9-224.3)
Klebsiella pneumoniae	Fluoroquinolones	1580 (1160-2140)	14.4 (10.5-19.4)	140000 (102000-189000)	1275.1 (932.0-1718.6)	109 (66-165)	1.0 (0.6-1.5)	9690 (5880-14600)	88.1 (53.5-132.7)
Klebsiella pneumoniae	Third-generation cephalosporins	2590 (1740-3600)	23.6 (15.8-32.7)	229000 (154000-318000)	2084.4 (1397.6-2894.4)	341 (131-638)	3.1 (1.2-5.8)	30200 (11600-56400)	274.5 (105.2-512.9)
Klebsiella pneumoniae	Trimethoprim-Sulfamethoxazole	2170 (1600-2950)	19.8 (14.6-26.8)	192000 (142000-261000)	1749.4 (1289.8-2370.2)	107 (53-178)	1.0 (0.5-1.6)	9490 (4670-15700)	86.3 (42.5-142.8)
Klebsiella pneumoniae	Resistance to one or more antibiotics	2890 (2110-3840)	26.2 (19.2-34.9)	255000 (187000-339000)	2322.2 (1699.2-3087.3)	844 (539-1230)	7.7 (4.9-11.2)	74700 (47700-109000)	679.4 (433.6-990.5)
Morganella spp.	Fluoroquinolones	1 (0-2)	0.0 (0.0-0.0)	74 (20-191)	0.7 (0.2-1.7)	0 (0-0)	0.0 (0.0-0.0)	12 (3-34)	0.1 (0.0-0.3)
Morganella spp.	Fourth-generation cephalosporins	0 (0-1)	0.0 (0.0-0.0)	37 (10-94)	0.3 (0.1-0.9)	0 (0-0)	0.0 (0.0-0.0)	6 (1-19)	0.1 (0.0-0.2)
Morganella spp.	Third-generation cephalosporins	0 (0-1)	0.0 (0.0-0.0)	32 (9-82)	0.3 (0.1-0.7)	0 (0-0)	0.0 (0.0-0.0)	3 (1-8)	0.0 (0.0-0.1)
Morganella spp.	Resistance to one or more antibiotics	1 (0-3)	0.0 (0.0-0.0)	88 (25-224)	0.8 (0.2-2.0)	0 (0-1)	0.0 (0.0-0.0)	21 (5-57)	0.2 (0.0-0.5)
Mycobacterium tuberculosis	Extensive drug resistance in TB	2 (0-6)	0.0 (0.0-0.1)	165 (35-498)	1.5 (0.3-4.5)	1 (0-3)	0.0 (0.0-0.0)	101 (22-304)	0.9 (0.2-2.8)
Mycobacterium tuberculosis	Isoniazid mono-resistance	6 (3-13)	0.1 (0.0-0.1)	566 (274-1140)	5.1 (2.5-10.3)	1 (0-4)	0.0 (0.0-0.0)	89 (0-330)	0.8 (0.0-3.0)
Mycobacterium tuberculosis	Multi-drug resistance excluding extensive drug resistance in TB	25 (6-73)	0.2 (0.1-0.7)	2210 (517-6470)	20.1 (4.7-58.9)	15 (1-48)	0.1 (0.0-0.4)	1310 (106-4260)	11.9 (1.0-38.7)
Mycobacterium tuberculosis	Rifampicin mono-resistance	1 (0-1)	0.0 (0.0-0.0)	52 (28-90)	0.5 (0.3-0.8)	0 (0-0)	0.0 (0.0-0.0)	15 (2-34)	0.1 (0.0-0.3)
Mycobacterium tuberculosis	Resistance to one or more antibiotics	34 (12-86)	0.3 (0.1-0.8)	2990 (1070-7660)	27.2 (9.7-69.7)	17 (2-53)	0.2 (0.0-0.5)	1510 (187-4650)	13.7 (1.7-42.3)
Neisseria gonorrhoeae	Fluoroquinolones	--	--	--	--	--	--	--	--
Neisseria gonorrhoeae	Third-generation cephalosporins	--	--	--	--	--	--	--	--
Neisseria gonorrhoeae	Resistance to one or more antibiotics	--	--	--	--	--	--	--	--
Proteus spp.	Aminoglycosides	26 (14-43)	0.2 (0.1-0.4)	2350 (1250-3850)	21.4 (11.4-35.0)	1 (0-3)	0.0 (0.0-0.0)	104 (12-232)	0.9 (0.1-2.1)
Proteus spp.	Aminopenicillin	65 (36-105)	0.6 (0.3-1.0)	5800 (3210-9280)	52.7 (29.2-84.4)	1 (0-3)	0.0 (0.0-0.0)	83 (0-258)	0.8 (0.0-2.4)
Proteus spp.	Fluoroquinolones	35 (19-57)	0.3 (0.2-0.5)	3100 (1710-5030)	28.2 (15.6-45.7)	2 (1-5)	0.0 (0.0-0.0)	196 (73-412)	1.8 (0.7-3.7)
Proteus spp.	Third-generation cephalosporins	31 (16-50)	0.3 (0.1-0.5)	2750 (1440-4480)	25.0 (13.1-40.8)	6 (2-12)	0.1 (0.0-0.1)	517 (155-1080)	4.7 (1.4-9.8)
Proteus spp.	Trimethoprim-Sulfamethoxazole	51 (28-80)	0.5 (0.3-0.7)	4490 (2520-7090)	40.9 (22.9-64.5)	2 (0-4)	0.0 (0.0-0.0)	155 (0-363)	1.4 (0.0-3.3)
Proteus spp.	Resistance to one or more antibiotics	71 (39-112)	0.6 (0.4-1.0)	6280 (3500-9970)	57.1 (31.8-90.7)	12 (5-21)	0.1 (0.0-0.2)	1060 (461-1890)	9.6 (4.2-17.2)
Pseudomonas aeruginosa	Aminoglycosides	386 (272-537)	3.5 (2.5-4.9)	34100 (24100-47500)	310.6 (219.4-432.3)	10 (0-24)	0.1 (0.0-0.2)	916 (0-2170)	8.3 (0.0-19.7)
Pseudomonas aeruginosa	Anti-pseudomonal penicillin/Beta-Lactamase inhibitors	439 (320-585)	4.0 (2.9-5.3)	38900 (28300-51700)	353.6 (257.8-470.5)	34 (21-53)	0.3 (0.2-0.5)	2960 (1820-4670)	27.0 (16.6-42.5)
Pseudomonas aeruginosa	Carbapenem	533 (384-720)	4.9 (3.5-6.5)	47200 (34000-63700)	429.3 (309.2-579.1)	96 (63-139)	0.9 (0.6-1.3)	8460 (5560-12300)	76.9 (50.6-112.2)
Pseudomonas aeruginosa	Fluoroquinolones	487 (350-662)	4.4 (3.2-6.0)	43100 (31000-58600)	391.8 (281.9-533.2)	51 (33-76)	0.5 (0.3-0.7)	4560 (2940-6730)	41.5 (26.8-61.2)
Pseudomonas aeruginosa	Fourth-generation cephalosporins	469 (338-640)	4.3 (3.1-5.8)	41500 (29900-56600)	377.9 (271.9-514.9)	16 (8-27)	0.1 (0.1-0.2)	1400 (738-2420)	12.8 (6.7-22.0)
Pseudomonas aeruginosa	Third-generation cephalosporins	708 (515-944)	6.4 (4.7-8.6)	62700 (45600-83500)	570.3 (414.7-759.6)	46 (21-77)	0.4 (0.2-0.7)	4030 (1820-6810)	36.7 (16.6-61.9)
Pseudomonas aeruginosa	Resistance to one or more antibiotics	981 (727-1310)	8.9 (6.6-11.9)	86800 (64300-116000)	789.5 (585.1-1051.6)	252 (166-375)	2.3 (1.5-3.4)	22300 (14700-33200)	203.1 (133.5-302.0)
Salmonella Paratyphi	Fluoroquinolones	0 (0-0)	0.0 (0.0-0.0)	3 (1-7)	0.0 (0.0-0.1)	0 (0-0)	0.0 (0.0-0.0)	1 (0-2)	0.0 (0.0-0.0)
Salmonella Paratyphi	Multi-drug resistance in Salmonella Typhi and Paratyphi	0 (0-0)	0.0 (0.0-0.0)	0 (0-0)	0.0 (0.0-0.0)	0 (0-0)	0.0 (0.0-0.0)	0 (0-0)	0.0 (0.0-0.0)
Salmonella Paratyphi	Resistance to one or more antibiotics	0 (0-0)	0.0 (0.0-0.0)	3 (1-7)	0.0 (0.0-0.1)	0 (0-0)	0.0 (0.0-0.0)	1 (0-2)	0.0 (0.0-0.0)
Salmonella Typhi	Fluoroquinolones	166 (55-359)	1.5 (0.5-3.3)	14700 (4830-31800)	133.3 (43.9-289.0)	33 (4-91)	0.3 (0.0-0.8)	2960 (380-8070)	26.9 (3.5-73.4)
Salmonella Typhi	Multi-drug resistance in Salmonella Typhi and Paratyphi	67 (27-142)	0.6 (0.2-1.3)	5890 (2380-12500)	53.5 (21.7-114.0)	8 (0-25)	0.1 (0.0-0.2)	701 (0-2240)	6.4 (0.0-20.4)
Salmonella Typhi	Resistance to one or more antibiotics	217 (89-434)	2.0 (0.8-3.9)	19200 (7840-38400)	174.7 (71.4-348.8)	41 (9-102)	0.4 (0.1-0.9)	3660 (783-8980)	33.3 (7.1-81.7)
Non-typhoidal *Salmonella*	Fluoroquinolones	12 (4-28)	0.1 (0.0-0.3)	1200 (408-2930)	10.9 (3.7-26.6)	2 (0-7)	0.0 (0.0-0.1)	227 (32-632)	2.1 (0.3-5.7)
Non-typhoidal *Salmonella*	Resistance to one or more antibiotics	12 (4-28)	0.1 (0.0-0.3)	1200 (408-2930)	10.9 (3.7-26.6)	2 (0-7)	0.0 (0.0-0.1)	227 (32-632)	2.1 (0.3-5.7)
Serratia spp.	Aminoglycosides	27 (14-49)	0.2 (0.1-0.4)	2380 (1240-4300)	21.6 (11.3-39.1)	2 (0-5)	0.0 (0.0-0.0)	144 (0-443)	1.3 (0.0-4.0)
Serratia spp.	Anti-pseudomonal penicillin/Beta-Lactamase inhibitors	20 (11-36)	0.2 (0.1-0.3)	1800 (970-3170)	16.4 (8.8-28.8)	4 (2-8)	0.0 (0.0-0.1)	353 (154-697)	3.2 (1.4-6.3)
Serratia spp.	Carbapenem	17 (9-30)	0.2 (0.1-0.3)	1520 (798-2630)	13.9 (7.3-24.0)	5 (2-9)	0.0 (0.0-0.1)	410 (187-811)	3.7 (1.7-7.4)
Serratia spp.	Fluoroquinolones	18 (10-31)	0.2 (0.1-0.3)	1600 (844-2790)	14.6 (7.7-25.4)	2 (0-5)	0.0 (0.0-0.0)	172 (31-404)	1.6 (0.3-3.7)
Serratia spp.	Fourth-generation cephalosporins	35 (18-61)	0.3 (0.2-0.6)	3060 (1620-5360)	27.8 (14.8-48.8)	3 (1-7)	0.0 (0.0-0.1)	299 (106-640)	2.7 (1.0-5.8)
Serratia spp.	Third-generation cephalosporins	43 (23-75)	0.4 (0.2-0.7)	3850 (2040-6680)	35.0 (18.6-60.8)	3 (1-7)	0.0 (0.0-0.1)	259 (78-614)	2.4 (0.7-5.6)
Serratia spp.	Resistance to one or more antibiotics	75 (41-126)	0.7 (0.4-1.1)	6640 (3670-11100)	60.4 (33.4-101.4)	18 (9-34)	0.2 (0.1-0.3)	1640 (813-3010)	14.9 (7.4-27.4)
Shigella spp.	Fluoroquinolones	160 (45-410)	1.5 (0.4-3.7)	14600 (4060-37100)	132.5 (37.0-337.3)	33 (3-98)	0.3 (0.0-0.9)	2980 (285-8740)	27.1 (2.6-79.5)
Shigella spp.	Resistance to one or more antibiotics	160 (45-410)	1.5 (0.4-3.7)	14600 (4060-37100)	132.5 (37.0-337.3)	33 (3-98)	0.3 (0.0-0.9)	2980 (285-8740)	27.1 (2.6-79.5)
Staphylococcus aureus	Fluoroquinolones	648 (463-871)	5.9 (4.2-7.9)	57400 (40900-77100)	522.3 (372.5-701.3)	28 (11-48)	0.3 (0.1-0.4)	2480 (1020-4290)	22.6 (9.3-39.0)

Pathogen	Antibiotic class	1 to 4							
		Associated with resistance				Attributable to resistance			
		Deaths		DALYs		Deaths		DALYs	
		Counts	Rate per 100 000	Counts	Rate per 100 000	Counts	Rate per 100 000	Counts	Rate per 100 000
All pathogens	Resistance to one or more antibiotics	7310 (5150-10100)	15.3 (10.8-21.1)	641000 (452000-882000)	1340.8 (946.4-1845.7)	1840 (1250-2570)	3.8 (2.6-5.4)	160000 (110000-223000)	335.5 (229.3-467.5)
Acinetobacter baumannii	Aminoglycosides	159 (84-270)	0.3 (0.2-0.6)	13700 (7250-23300)	28.7 (15.2-48.7)	7 (0-18)	0.0 (0.0-0.0)	577 (2-1530)	1.2 (0.0-3.2)
Acinetobacter baumannii	Anti-pseudomonal penicillin/Beta-Lactamase inhibitors	208 (112-349)	0.4 (0.2-0.7)	18000 (9670-30000)	37.6 (20.2-62.8)	8 (3-14)	0.0 (0.0-0.0)	664 (299-1240)	1.4 (0.6-2.6)
Acinetobacter baumannii	Beta Lactam/Beta-lactamase inhibitors	193 (103-323)	0.4 (0.2-0.7)	16700 (8920-27800)	34.8 (18.7-58.2)	0 (0-0)	0.0 (0.0-0.0)	10 (2-25)	0.0 (0.0-0.1)
Acinetobacter baumannii	Carbapenem	171 (92-291)	0.4 (0.2-0.6)	14700 (7910-25100)	30.8 (16.6-52.4)	28 (13-52)	0.1 (0.0-0.1)	2380 (1090-4500)	5.0 (2.3-9.4)
Acinetobacter baumannii	Fluoroquinolones	196 (105-332)	0.4 (0.2-0.7)	16900 (9030-28600)	35.4 (18.9-59.8)	25 (11-45)	0.1 (0.0-0.1)	2140 (946-3840)	4.5 (2.0-8.0)
Acinetobacter baumannii	Fourth-generation cephalosporins	203 (109-337)	0.4 (0.2-0.7)	17500 (9360-29100)	36.6 (19.6-60.9)	0 (0-1)	0.0 (0.0-0.0)	20 (7-52)	0.0 (0.0-0.1)
Acinetobacter baumannii	Third-generation cephalosporins	198 (106-329)	0.4 (0.2-0.7)	17100 (9170-28300)	35.8 (19.2-59.3)	1 (0-1)	0.0 (0.0-0.0)	51 (16-115)	0.1 (0.0-0.2)
Acinetobacter baumannii	Resistance to one or more antibiotics	213 (115-357)	0.4 (0.2-0.7)	18400 (9910-30800)	38.5 (20.7-64.4)	68 (35-119)	0.1 (0.1-0.2)	5850 (3010-10200)	12.2 (6.3-21.4)
Citrobacter spp.	Aminoglycosides	5 (2-10)	0.0 (0.0-0.0)	443 (189-859)	0.9 (0.4-1.8)	0 (0-1)	0.0 (0.0-0.0)	23 (0-64)	0.0 (0.0-0.1)
Citrobacter spp.	Anti-pseudomonal penicillin/Beta-Lactamase inhibitors	15 (6-30)	0.0 (0.0-0.1)	1290 (544-2540)	2.7 (1.1-5.3)	3 (1-5)	0.0 (0.0-0.0)	221 (78-465)	0.5 (0.2-1.0)
Citrobacter spp.	Carbapenem	9 (4-18)	0.0 (0.0-0.0)	786 (329-1520)	1.6 (0.7-3.2)	2 (1-4)	0.0 (0.0-0.0)	179 (66-380)	0.4 (0.1-0.8)
Citrobacter spp.	Fluoroquinolones	24 (10-46)	0.1 (0.0-0.1)	2090 (896-4000)	4.4 (1.9-8.4)	4 (1-8)	0.0 (0.0-0.0)	333 (112-707)	0.7 (0.2-1.5)
Citrobacter spp.	Fourth-generation cephalosporins	17 (7-33)	0.0 (0.0-0.1)	1480 (617-2870)	3.1 (1.3-6.0)	1 (0-3)	0.0 (0.0-0.0)	125 (38-282)	0.3 (0.1-0.6)
Citrobacter spp.	Third-generation cephalosporins	30 (13-59)	0.1 (0.0-0.1)	2600 (1110-5060)	5.4 (2.3-10.6)	2 (1-5)	0.0 (0.0-0.0)	211 (69-470)	0.4 (0.1-1.0)
Citrobacter spp.	Resistance to one or more antibiotics	44 (19-85)	0.1 (0.0-0.2)	3790 (1640-7330)	7.9 (3.4-15.3)	13 (5-25)	0.0 (0.0-0.1)	1090 (458-2140)	2.3 (1.0-4.5)
Enterobacter spp.	Aminoglycosides	32 (18-50)	0.1 (0.0-0.1)	2740 (1590-4330)	5.7 (3.3-9.0)	2 (1-4)	0.0 (0.0-0.0)	174 (47-356)	0.4 (0.1-0.7)
Enterobacter spp.	Anti-pseudomonal penicillin/Beta-Lactamase inhibitors	77 (47-126)	0.2 (0.1-0.3)	6660 (4030-10800)	13.9 (8.4-22.6)	9 (4-15)	0.0 (0.0-0.0)	764 (386-1300)	1.6 (0.8-2.7)
Enterobacter spp.	Carbapenem	35 (21-56)	0.1 (0.0-0.1)	3020 (1840-4790)	6.3 (3.8-10.0)	9 (5-15)	0.0 (0.0-0.0)	742 (405-1260)	1.6 (0.8-2.6)
Enterobacter spp.	Fluoroquinolones	46 (27-76)	0.1 (0.1-0.2)	3960 (2320-6540)	8.3 (4.9-13.7)	5 (2-9)	0.0 (0.0-0.0)	429 (203-794)	0.9 (0.4-1.7)
Enterobacter spp.	Fourth-generation cephalosporins	70 (42-113)	0.1 (0.1-0.2)	6060 (3630-9770)	12.7 (7.6-20.4)	3 (1-6)	0.0 (0.0-0.0)	294 (130-559)	0.6 (0.3-1.2)
Enterobacter spp.	Trimethoprim-Sulfamethoxazole	62 (37-99)	0.1 (0.1-0.2)	5350 (3210-8570)	11.2 (6.7-17.9)	3 (0-8)	0.0 (0.0-0.0)	298 (0-669)	0.6 (0.0-1.4)
Enterobacter spp.	Resistance to one or more antibiotics	132 (81-208)	0.3 (0.2-0.4)	11400 (7010-18000)	23.8 (14.7-37.6)	31 (19-50)	0.1 (0.0-0.1)	2700 (1610-4340)	5.6 (3.4-9.1)
Enterococcus faecalis	Fluoroquinolones	22 (10-39)	0.0 (0.0-0.1)	1910 (928-3380)	4.0 (1.9-7.1)	5 (2-11)	0.0 (0.0-0.0)	466 (173-923)	1.0 (0.4-1.9)
Enterococcus faecalis	Vancomycin	4 (2-7)	0.0 (0.0-0.0)	321 (157-597)	0.7 (0.3-1.2)	1 (0-3)	0.0 (0.0-0.0)	108 (42-231)	0.2 (0.1-0.5)
Enterococcus faecalis	Resistance to one or more antibiotics	24 (11-43)	0.0 (0.0-0.1)	2090 (1020-3730)	4.4 (2.1-7.8)	7 (3-13)	0.0 (0.0-0.0)	574 (246-1100)	1.2 (0.5-2.3)
Enterococcus faecium	Fluoroquinolones	80 (38-154)	0.2 (0.1-0.3)	6920 (3290-13200)	14.5 (6.9-27.7)	14 (4-32)	0.0 (0.0-0.1)	1180 (315-2760)	2.5 (0.7-5.8)
Enterococcus faecium	Vancomycin	41 (19-78)	0.1 (0.0-0.2)	3530 (1650-6720)	7.4 (3.5-14.1)	9 (4-20)	0.0 (0.0-0.0)	811 (313-1730)	1.7 (0.7-3.6)
Enterococcus faecium	Resistance to one or more antibiotics	81 (38-154)	0.2 (0.1-0.3)	6970 (3310-13300)	14.6 (6.9-27.8)	23 (10-49)	0.0 (0.0-0.1)	1990 (887-4180)	4.2 (1.9-8.7)
Other enterococci	Fluoroquinolones	40 (18-79)	0.1 (0.0-0.2)	3540 (1660-6930)	7.4 (3.5-14.5)	7 (1-17)	0.0 (0.0-0.0)	621 (104-1440)	1.3 (0.2-3.0)
Other enterococci	Vancomycin	11 (5-22)	0.0 (0.0-0.0)	988 (454-1960)	2.1 (0.9-4.1)	2 (1-4)	0.0 (0.0-0.0)	174 (70-392)	0.4 (0.1-0.8)
Other enterococci	Resistance to one or more antibiotics	41 (19-80)	0.1 (0.0-0.2)	3580 (1670-7030)	7.5 (3.5-14.7)	9 (3-20)	0.0 (0.0-0.0)	795 (271-1730)	1.7 (0.6-3.6)
Escherichia coli	Aminoglycosides	164 (109-241)	0.3 (0.2-0.5)	15200 (10200-22800)	31.9 (21.3-47.7)	9 (6-15)	0.0 (0.0-0.0)	871 (536-1330)	1.8 (1.1-2.8)
Escherichia coli	Aminopenicillin	821 (559-1190)	1.7 (1.2-2.5)	75900 (51400-111000)	158.9 (107.5-231.9)	8 (5-13)	0.0 (0.0-0.0)	785 (486-1220)	1.6 (1.0-2.5)
Escherichia coli	Beta Lactam/Beta-lactamase inhibitors	606 (417-885)	1.3 (0.9-1.9)	56000 (37900-81700)	117.2 (79.3-170.9)	14 (9-21)	0.0 (0.0-0.0)	1310 (808-1990)	2.7 (1.7-4.2)
Escherichia coli	Carbapenem	155 (101-237)	0.3 (0.2-0.5)	14400 (9380-22100)	30.2 (19.6-46.2)	32 (17-56)	0.1 (0.0-0.1)	2950 (1630-5020)	6.2 (3.4-10.5)
Escherichia coli	Fluoroquinolones	570 (389-826)	1.2 (0.8-1.7)	52500 (35300-75900)	109.9 (73.9-158.8)	58 (37-86)	0.1 (0.1-0.2)	5250 (3400-7930)	11.0 (7.1-16.6)
Escherichia coli	Third-generation cephalosporins	642 (435-933)	1.3 (0.9-2.0)	59200 (39700-86400)	123.8 (83.1-180.8)	84 (36-149)	0.2 (0.1-0.3)	7630 (3420-13400)	16.0 (7.2-28.1)
Escherichia coli	Trimethoprim-Sulfamethoxazole	628 (426-908)	1.3 (0.9-1.9)	58100 (39200-84000)	121.5 (82.1-175.7)	34 (22-52)	0.1 (0.0-0.1)	3060 (1900-4670)	6.4 (4.0-9.8)
Escherichia coli	Resistance to one or more antibiotics	865 (589-1250)	1.8 (1.2-2.6)	80100 (54300-117000)	167.5 (113.5-243.8)	240 (150-370)	0.5 (0.3-0.8)	21900 (13700-33400)	45.7 (28.6-69.9)
Group A *Streptococcus*	Macrolide	13 (6-23)	0.0 (0.0-0.0)	1170 (573-2070)	2.5 (1.2-4.3)	1 (0-4)	0.0 (0.0-0.0)	112 (0-365)	0.2 (0.0-0.8)
Group A *Streptococcus*	Resistance to one or more antibiotics	13 (6-23)	0.0 (0.0-0.0)	1170 (573-2070)	2.5 (1.2-4.3)	1 (0-4)	0.0 (0.0-0.0)	112 (0-365)	0.2 (0.0-0.8)
Group B *Streptococcus*	Fluoroquinolones	67 (40-105)	0.1 (0.1-0.2)	5830 (3520-9130)	12.2 (7.4-19.1)	10 (2-25)	0.0 (0.0-0.1)	904 (133-2170)	1.9 (0.3-4.5)
Group B *Streptococcus*	Macrolide	257 (164-379)	0.5 (0.3-0.8)	22400 (14400-32900)	46.8 (30.0-68.8)	25 (0-69)	0.1 (0.0-0.1)	2190 (0-5950)	4.6 (0.0-12.5)
Group B *Streptococcus*	Penicillin	4 (2-6)	0.0 (0.0-0.0)	349 (202-537)	0.7 (0.4-1.1)	1 (0-2)	0.0 (0.0-0.0)	81 (15-181)	0.2 (0.0-0.4)
Group B *Streptococcus*	Resistance to one or more antibiotics	273 (174-401)	0.6 (0.4-0.8)	23700 (15200-34800)	49.6 (31.8-72.8)	37 (1-83)	0.1 (0.0-0.2)	3180 (92-7170)	6.6 (0.2-15.0)
Haemophilus influenzae	Aminopenicillin	106 (69-158)	0.2 (0.1-0.3)	9210 (6050-13700)	19.3 (12.7-28.7)	16 (1-35)	0.0 (0.0-0.1)	1420 (124-3010)	3.0 (0.3-6.3)
Haemophilus influenzae	Third-generation cephalosporins	40 (26-57)	0.1 (0.1-0.1)	3490 (2300-4980)	7.3 (4.8-10.4)	12 (5-20)	0.0 (0.0-0.0)	1010 (423-1770)	2.1 (0.9-3.7)
Haemophilus influenzae	Resistance to one or more antibiotics	127 (85-183)	0.3 (0.2-0.4)	11100 (7430-15900)	23.2 (15.5-33.3)	28 (11-50)	0.1 (0.0-0.1)	2430 (951-4300)	5.1 (2.0-9.0)
Klebsiella pneumoniae	Aminoglycosides	470 (302-704)	1.0 (0.6-1.5)	40600 (26100-60800)	84.9 (54.7-127.1)	37 (21-59)	0.1 (0.0-0.1)	3160 (1830-5090)	6.6 (3.8-10.7)
Klebsiella pneumoniae	Beta Lactam/Beta-lactamase inhibitors	915 (611-1340)	1.9 (1.3-2.8)	79000 (52900-115000)	165.3 (110.6-241.5)	9 (3-24)	0.0 (0.0-0.1)	814 (229-2070)	1.7 (0.5-4.3)
Klebsiella pneumoniae	Carbapenem	250 (131-444)	0.5 (0.3-0.9)	21600 (11300-38300)	45.2 (23.7-80.2)	59 (30-105)	0.1 (0.1-0.2)	5070 (2600-9030)	10.6 (5.4-18.9)
Klebsiella pneumoniae	Fluoroquinolones	581 (387-844)	1.2 (0.8-1.8)	50300 (33400-72800)	105.1 (70.0-152.4)	40 (23-65)	0.1 (0.0-0.1)	3500 (2010-5580)	7.3 (4.2-11.7)
Klebsiella pneumoniae	Third-generation cephalosporins	954 (619-1440)	2.0 (1.3-3.0)	82400 (53500-124000)	172.3 (111.8-259.6)	126 (50-246)	0.3 (0.1-0.5)	10900 (4370-21200)	22.8 (9.1-44.4)
Klebsiella pneumoniae	Trimethoprim-Sulfamethoxazole	797 (534-1170)	1.7 (1.1-2.5)	68800 (46200-101000)	143.9 (96.5-211.9)	39 (18-69)	0.1 (0.0-0.1)	3390 (1590-5910)	7.1 (3.3-12.4)
Klebsiella pneumoniae	Resistance to one or more antibiotics	1060 (709-1560)	2.2 (1.5-3.3)	91500 (61200-134000)	191.4 (127.9-280.8)	311 (184-501)	0.7 (0.4-1.0)	26800 (15800-43200)	56.1 (33.1-90.3)
Morganella spp.	Fluoroquinolones	0 (0-1)	0.0 (0.0-0.0)	40 (14-96)	0.1 (0.0-0.2)	0 (0-0)	0.0 (0.0-0.0)	7 (2-17)	0.0 (0.0-0.0)
Morganella spp.	Fourth-generation cephalosporins	0 (0-0)	0.0 (0.0-0.0)	16 (5-39)	0.0 (0.0-0.1)	0 (0-0)	0.0 (0.0-0.0)	3 (1-8)	0.0 (0.0-0.0)
Morganella spp.	Third-generation cephalosporins	0 (0-0)	0.0 (0.0-0.0)	16 (5-39)	0.0 (0.0-0.1)	0 (0-0)	0.0 (0.0-0.0)	2 (0-5)	0.0 (0.0-0.0)
Morganella spp.	Resistance to one or more antibiotics	1 (0-1)	0.0 (0.0-0.0)	47 (16-110)	0.1 (0.0-0.2)	0 (0-0)	0.0 (0.0-0.0)	11 (3-27)	0.0 (0.0-0.1)
Mycobacterium tuberculosis	Extensive drug resistance in TB	1 (0-4)	0.0 (0.0-0.0)	124 (29-368)	0.3 (0.1-0.8)	1 (0-3)	0.0 (0.0-0.0)	74 (17-222)	0.2 (0.0-0.5)
Mycobacterium tuberculosis	Isoniazid mono-resistance	5 (3-10)	0.0 (0.0-0.0)	524 (278-969)	1.1 (0.6-2.0)	1 (0-3)	0.0 (0.0-0.0)	77 (0-284)	0.2 (0.0-0.6)
Mycobacterium tuberculosis	Multi-drug resistance excluding extensive drug resistance in TB	19 (5-54)	0.0 (0.0-0.1)	1700 (443-4790)	3.6 (0.9-10.0)	11 (1-35)	0.0 (0.0-0.0)	954 (73-3040)	2.0 (0.2-6.4)
Mycobacterium tuberculosis	Rifampicin mono-resistance	1 (0-1)	0.0 (0.0-0.0)	61 (36-97)	0.1 (0.1-0.2)	0 (0-0)	0.0 (0.0-0.0)	15 (3-33)	0.0 (0.0-0.1)
Mycobacterium tuberculosis	Resistance to one or more antibiotics	26 (10-66)	0.1 (0.0-0.1)	2410 (951-5930)	5.1 (2.0-12.4)	13 (2-39)	0.0 (0.0-0.0)	1120 (150-3320)	2.3 (0.3-6.9)
Neisseria gonorrhoeae	Fluoroquinolones	--	--	--	--	--	--	--	--
Neisseria gonorrhoeae	Third-generation cephalosporins	--	--	--	--	--	--	--	--
Neisseria gonorrhoeae	Resistance to one or more antibiotics	--	--	--	--	--	--	--	--
Proteus spp.	Aminoglycosides	11 (5-20)	0.0 (0.0-0.0)	978 (486-1730)	2.0 (1.0-3.6)	0 (0-1)	0.0 (0.0-0.0)	43 (5-99)	0.1 (0.0-0.2)
Proteus spp.	Aminopenicillin	28 (14-48)	0.1 (0.0-0.1)	2440 (1230-4210)	5.1 (2.6-8.8)	0 (0-1)	0.0 (0.0-0.0)	35 (0-113)	0.1 (0.0-0.2)
Proteus spp.	Fluoroquinolones	15 (7-26)	0.0 (0.0-0.1)	1310 (643-2280)	2.7 (1.3-4.8)	1 (0-2)	0.0 (0.0-0.0)	81 (28-181)	0.2 (0.1-0.4)
Proteus spp.	Third-generation cephalosporins	13 (6-24)	0.0 (0.0-0.0)	1170 (573-2080)	2.5 (1.2-4.4)	2 (1-5)	0.0 (0.0-0.0)	221 (63-473)	0.5 (0.1-1.0)
Proteus spp.	Trimethoprim-Sulfamethoxazole	21 (10-37)	0.0 (0.0-0.1)	1880 (965-3280)	3.9 (2.0-6.9)	1 (0-2)	0.0 (0.0-0.0)	65 (1-157)	0.1 (0.0-0.3)
Proteus spp.	Resistance to one or more antibiotics	30 (15-52)	0.1 (0.0-0.1)	2640 (1330-4540)	5.5 (2.8-9.5)	5 (2-10)	0.0 (0.0-0.0)	445 (183-863)	0.9 (0.4-1.8)
Pseudomonas aeruginosa	Aminoglycosides	146 (98-212)	0.3 (0.2-0.4)	12600 (8520-18300)	26.4 (17.8-38.2)	4 (0-9)	0.0 (0.0-0.0)	341 (0-817)	0.7 (0.0-1.7)
Pseudomonas aeruginosa	Anti-pseudomonal penicillin/Beta-Lactamase inhibitors	168 (114-243)	0.4 (0.2-0.5)	14500 (9890-21000)	30.3 (20.7-44.0)	12 (7-20)	0.0 (0.0-0.0)	1070 (622-1740)	2.2 (1.3-3.6)
Pseudomonas aeruginosa	Carbapenem	206 (141-298)	0.4 (0.3-0.6)	17900 (12200-25800)	37.4 (25.5-53.9)	37 (23-55)	0.1 (0.0-0.1)	3190 (1980-4770)	6.7 (4.1-10.0)
Pseudomonas aeruginosa	Fluoroquinolones	189 (127-274)	0.4 (0.3-0.6)	16400 (11000-23800)	34.2 (23.0-49.7)	20 (12-31)	0.0 (0.0-0.1)	1740 (1070-2690)	3.6 (2.2-5.6)
Pseudomonas aeruginosa	Fourth-generation cephalosporins	176 (118-254)	0.4 (0.2-0.5)	15200 (10300-21900)	31.8 (21.4-45.8)	6 (3-10)	0.0 (0.0-0.0)	491 (246-888)	1.0 (0.5-1.9)
Pseudomonas aeruginosa	Third-generation cephalosporins	266 (176-376)	0.6 (0.4-0.8)	23000 (15300-32500)	48.1 (31.9-68.0)	17 (7-29)	0.0 (0.0-0.1)	1450 (632-2540)	3.0 (1.3-5.3)
Pseudomonas aeruginosa	Resistance to one or more antibiotics	373 (252-536)	0.8 (0.5-1.1)	32200 (21900-46300)	67.6 (45.8-96.9)	96 (59-147)	0.2 (0.1-0.3)	8290 (5100-12700)	17.3 (10.7-26.6)
Salmonella Paratyphi	Fluoroquinolones	2 (1-6)	0.0 (0.0-0.0)	185 (46-486)	0.4 (0.1-1.0)	0 (0-1)	0.0 (0.0-0.0)	37 (4-115)	0.1 (0.0-0.2)
Salmonella Paratyphi	Multi-drug resistance in Salmonella Typhi and Paratyphi	0 (0-0)	0.0 (0.0-0.0)	6 (2-17)	0.0 (0.0-0.0)	0 (0-0)	0.0 (0.0-0.0)	1 (0-2)	0.0 (0.0-0.0)
Salmonella Paratyphi	Resistance to one or more antibiotics	2 (1-6)	0.0 (0.0-0.0)	187 (46-490)	0.4 (0.1-1.0)	0 (0-1)	0.0 (0.0-0.0)	38 (5-117)	0.1 (0.0-0.2)
Salmonella Typhi	Fluoroquinolones	109 (42-220)	0.2 (0.1-0.5)	9410 (3600-19100)	19.7 (7.5-39.9)	22 (2-56)	0.0 (0.0-0.1)	1880 (201-4840)	3.9 (0.4-10.1)
Salmonella Typhi	Multi-drug resistance in Salmonella Typhi and Paratyphi	45 (19-87)	0.1 (0.0-0.2)	3850 (1610-7510)	8.1 (3.4-15.7)	5 (0-16)	0.0 (0.0-0.0)	451 (0-1350)	0.9 (0.0-2.8)
Salmonella Typhi	Resistance to one or more antibiotics	142 (59-268)	0.3 (0.1-0.6)	12300 (5110-23100)	25.6 (10.7-48.4)	27 (6-66)	0.1 (0.0-0.1)	2330 (480-5700)	4.9 (1.0-11.9)
Non-typhoidal *Salmonella*	Fluoroquinolones	7 (2-17)	0.0 (0.0-0.0)	894 (193-2520)	1.9 (0.4-5.3)	1 (0-4)	0.0 (0.0-0.0)	141 (17-455)	0.3 (0.0-1.0)
Non-typhoidal *Salmonella*	Resistance to one or more antibiotics	7 (2-17)	0.0 (0.0-0.0)	894 (193-2520)	1.9 (0.4-5.3)	1 (0-4)	0.0 (0.0-0.0)	141 (17-455)	0.3 (0.0-1.0)
Serratia spp.	Aminoglycosides	8 (4-16)	0.0 (0.0-0.0)	730 (340-1370)	1.5 (0.7-2.9)	1 (0-2)	0.0 (0.0-0.0)	44 (0-134)	0.1 (0.0-0.3)
Serratia spp.	Anti-pseudomonal penicillin/Beta-Lactamase inhibitors	6 (3-12)	0.0 (0.0-0.0)	549 (260-1080)	1.1 (0.5-2.3)	1 (0-3)	0.0 (0.0-0.0)	107 (42-217)	0.2 (0.1-0.5)
Serratia spp.	Carbapenem	6 (3-10)	0.0 (0.0-0.0)	477 (220-874)	1.0 (0.5-1.8)	1 (1-3)	0.0 (0.0-0.0)	128 (53-259)	0.3 (0.1-0.5)
Serratia spp.	Fluoroquinolones	6 (3-11)	0.0 (0.0-0.0)	500 (238-954)	1.0 (0.5-2.0)	1 (0-2)	0.0 (0.0-0.0)	54 (9-131)	0.1 (0.0-0.3)
Serratia spp.	Fourth-generation cephalosporins	11 (5-20)	0.0 (0.0-0.0)	939 (447-1760)	2.0 (0.9-3.7)	1 (0-2)	0.0 (0.0-0.0)	91 (30-205)	0.2 (0.1-0.4)
Serratia spp.	Third-generation cephalosporins	14 (6-26)	0.0 (0.0-0.1)	1180 (545-2240)	2.5 (1.1-4.7)	1 (0-2)	0.0 (0.0-0.0)	79 (23-184)	0.2 (0.0-0.4)
Serratia spp.	Resistance to one or more antibiotics	24 (11-44)	0.0 (0.0-0.1)	2040 (980-3820)	4.3 (2.0-8.0)	6 (3-11)	0.0 (0.0-0.0)	503 (222-966)	1.1 (0.5-2.0)
Shigella spp.	Fluoroquinolones	93 (23-236)	0.2 (0.0-0.5)	9390 (2590-23400)	19.7 (5.4-49.0)	19 (2-60)	0.0 (0.0-0.1)	1810 (245-5430)	3.8 (0.5-11.4)
Shigella spp.	Resistance to one or more antibiotics	93 (23-236)	0.2 (0.0-0.5)	9390 (2590-23400)	19.7 (5.4-49.0)	19 (2-60)	0.0 (0.0-0.1)	1810 (245-5430)	3.8 (0.5-11.4)
Staphylococcus aureus	Fluoroquinolones	263 (177-369)	0.6 (0.4-0.8)	23000 (15600-32200)	48.0 (32.5-67.4)	11 (5-20)	0.0 (0.0-0.0)	993 (419-1760)	2.1 (0.9-3.7)

Pathogen	Antibiotic class	5 and over							
		Associated with resistance				Attributable to resistance			
		Deaths		DALYs		Deaths		DALYs	
		Counts	Rate per 100 000	Counts	Rate per 100 000	Counts	Rate per 100 000	Counts	Rate per 100 000
All pathogens	Resistance to one or more antibiotics	200000 (133000-290000)	36.5 (24.3-52.8)	5170000 (3390000-7580000)	941.3 (617.9-1380.0)	54500 (35500-80600)	9.9 (6.5-14.7)	1390000 (895000-2070000)	252.6 (163.0-377.4)
Acinetobacter baumannii	Aminoglycosides	18600 (10600-29400)	3.4 (1.9-5.4)	455000 (250000-728000)	82.8 (45.5-132.5)	779 (0-1990)	0.1 (0.0-0.4)	19100 (130-49600)	3.5 (0.0-9.0)
Acinetobacter baumannii	Anti-pseudomonal penicillin/Beta-Lactamase inhibitors	22300 (12800-35100)	4.1 (2.3-6.4)	548000 (304000-884000)	99.8 (55.4-161.1)	620 (274-1150)	0.1 (0.0-0.2)	15400 (6640-28700)	2.8 (1.2-5.2)
Acinetobacter baumannii	Beta Lactam/Beta-lactamase inhibitors	21300 (12200-33700)	3.9 (2.2-6.1)	522000 (290000-845000)	95.1 (52.9-154.0)	15 (3-32)	0.0 (0.0-0.0)	382 (90-818)	0.1 (0.0-0.1)
Acinetobacter baumannii	Carbapenem	19000 (10800-29700)	3.5 (2.0-5.4)	465000 (258000-742000)	84.7 (47.0-135.2)	3060 (1510-5470)	0.6 (0.3-1.0)	74700 (36200-137000)	13.6 (6.6-25.0)
Acinetobacter baumannii	Fluoroquinolones	21800 (12400-34600)	4.0 (2.3-6.3)	536000 (297000-860000)	97.6 (54.0-156.7)	2760 (1290-4740)	0.5 (0.2-0.9)	67600 (31200-120000)	12.3 (5.7-21.9)
Acinetobacter baumannii	Fourth-generation cephalosporins	22000 (12500-34600)	4.0 (2.3-6.3)	538000 (299000-862000)	98.0 (54.5-157.1)	29 (11-63)	0.0 (0.0-0.0)	724 (268-1600)	0.1 (0.0-0.3)
Acinetobacter baumannii	Third-generation cephalosporins	21800 (12400-34400)	4.0 (2.2-6.3)	533000 (297000-860000)	97.1 (54.1-156.6)	68 (25-134)	0.0 (0.0-0.0)	1720 (620-3450)	0.3 (0.1-0.6)
Acinetobacter baumannii	Resistance to one or more antibiotics	23000 (13100-36300)	4.2 (2.4-6.6)	564000 (315000-911000)	102.7 (57.4-165.9)	7330 (3950-11900)	1.3 (0.7-2.2)	180000 (94600-301000)	32.7 (17.2-54.7)
Citrobacter spp.	Aminoglycosides	153 (87-249)	0.0 (0.0-0.0)	4000 (2200-6690)	0.7 (0.4-1.2)	8 (0-20)	0.0 (0.0-0.0)	208 (0-536)	0.0 (0.0-0.1)
Citrobacter spp.	Anti-pseudomonal penicillin/Beta-Lactamase inhibitors	415 (234-665)	0.1 (0.0-0.1)	10700 (5850-17500)	2.0 (1.1-3.2)	73 (34-138)	0.0 (0.0-0.0)	1890 (859-3570)	0.3 (0.2-0.7)
Citrobacter spp.	Carbapenem	273 (153-433)	0.0 (0.0-0.1)	6960 (3820-11200)	1.3 (0.7-2.0)	62 (30-114)	0.0 (0.0-0.0)	1590 (730-2900)	0.3 (0.1-0.5)
Citrobacter spp.	Fluoroquinolones	802 (452-1300)	0.1 (0.1-0.2)	20400 (11200-33500)	3.7 (2.0-6.1)	129 (54-240)	0.0 (0.0-0.0)	3280 (1370-6170)	0.6 (0.2-1.1)
Citrobacter spp.	Fourth-generation cephalosporins	498 (272-807)	0.1 (0.0-0.1)	13000 (6880-21500)	2.4 (1.3-3.9)	44 (16-85)	0.0 (0.0-0.0)	1140 (428-2290)	0.2 (0.1-0.4)
Citrobacter spp.	Third-generation cephalosporins	990 (576-1610)	0.2 (0.1-0.3)	25000 (14200-42000)	4.6 (2.6-7.7)	95 (37-192)	0.0 (0.0-0.0)	2350 (897-4800)	0.4 (0.2-0.9)
Citrobacter spp.	Resistance to one or more antibiotics	1430 (822-2320)	0.3 (0.1-0.4)	36400 (20400-59700)	6.6 (3.7-10.9)	411 (219-705)	0.1 (0.0-0.1)	10400 (5480-18100)	1.9 (1.0-3.3)
Enterobacter spp.	Aminoglycosides	1670 (969-2600)	0.3 (0.2-0.5)	44100 (25000-69000)	8.0 (4.5-12.6)	108 (31-223)	0.0 (0.0-0.0)	2860 (811-5950)	0.5 (0.1-1.1)
Enterobacter spp.	Anti-pseudomonal penicillin/Beta-Lactamase inhibitors	5080 (3030-7830)	0.9 (0.6-1.4)	131000 (76600-203000)	23.8 (13.9-37.0)	598 (302-1020)	0.1 (0.1-0.2)	15300 (7620-26800)	2.8 (1.4-4.9)
Enterobacter spp.	Carbapenem	2110 (1260-3240)	0.4 (0.2-0.6)	54000 (31000-83400)	9.8 (5.7-15.2)	531 (294-859)	0.1 (0.1-0.2)	13500 (7160-22300)	2.5 (1.3-4.1)
Enterobacter spp.	Fluoroquinolones	2660 (1580-4190)	0.5 (0.3-0.8)	68900 (39400-111000)	12.6 (7.2-20.3)	295 (144-534)	0.1 (0.0-0.1)	7570 (3650-14000)	1.4 (0.7-2.6)
Enterobacter spp.	Fourth-generation cephalosporins	3560 (2170-5410)	0.6 (0.4-1.0)	92900 (55000-145000)	16.9 (10.0-26.4)	137 (61-258)	0.0 (0.0-0.0)	3620 (1590-7000)	0.7 (0.3-1.3)
Enterobacter spp.	Trimethoprim-Sulfamethoxazole	4080 (2400-6330)	0.7 (0.4-1.2)	106000 (60900-169000)	19.3 (11.1-30.8)	239 (0-520)	0.0 (0.0-0.1)	6200 (0-13700)	1.1 (0.0-2.5)
Enterobacter spp.	Resistance to one or more antibiotics	8250 (4970-12600)	1.5 (0.9-2.3)	213000 (125000-332000)	38.7 (22.7-60.6)	1910 (1110-2950)	0.3 (0.2-0.5)	49100 (27800-77500)	8.9 (5.1-14.1)
Enterococcus faecalis	Fluoroquinolones	2590 (1390-4230)	0.5 (0.3-0.8)	69300 (37400-114000)	12.6 (6.8-20.7)	641 (266-1240)	0.1 (0.0-0.2)	17000 (7150-32500)	3.1 (1.3-5.9)
Enterococcus faecalis	Vancomycin	489 (266-830)	0.1 (0.0-0.2)	12800 (6860-21200)	2.3 (1.3-3.9)	165 (70-329)	0.0 (0.0-0.1)	4300 (1820-8290)	0.8 (0.3-1.5)
Enterococcus faecalis	Resistance to one or more antibiotics	2870 (1570-4680)	0.5 (0.3-0.9)	76500 (41600-125000)	13.9 (7.6-22.8)	807 (374-1470)	0.1 (0.1-0.3)	21300 (10100-39600)	3.9 (1.8-7.2)
Enterococcus faecium	Fluoroquinolones	11200 (6550-17900)	2.0 (1.2-3.3)	279000 (159000-454000)	50.8 (29.0-82.7)	1890 (554-3720)	0.3 (0.1-0.7)	47100 (13500-94300)	8.6 (2.5-17.2)
Enterococcus faecium	Vancomycin	5820 (3390-9320)	1.1 (0.6-1.7)	147000 (83600-239000)	26.8 (15.2-43.6)	1340 (633-2410)	0.2 (0.1-0.4)	33800 (15500-62500)	6.2 (2.8-11.4)
Enterococcus faecium	Resistance to one or more antibiotics	11300 (6590-18000)	2.1 (1.2-3.3)	281000 (160000-458000)	51.2 (29.2-83.4)	3230 (1750-5530)	0.6 (0.3-1.0)	80800 (42700-141000)	14.7 (7.8-25.7)
Other enterococci	Fluoroquinolones	3450 (2020-5380)	0.6 (0.4-1.0)	78100 (44900-125000)	14.2 (8.2-22.7)	594 (93-1220)	0.1 (0.0-0.2)	13400 (2210-28000)	2.4 (0.4-5.1)
Other enterococci	Vancomycin	1220 (718-1900)	0.2 (0.1-0.3)	26500 (15300-42100)	4.8 (2.8-7.7)	214 (103-403)	0.0 (0.0-0.1)	4710 (2270-8910)	0.9 (0.4-1.6)
Other enterococci	Resistance to one or more antibiotics	3510 (2060-5460)	0.6 (0.4-1.0)	79600 (45800-127000)	14.5 (8.3-23.1)	807 (359-1430)	0.1 (0.1-0.3)	18100 (7740-32800)	3.3 (1.4-6.0)
Escherichia coli	Aminoglycosides	7680 (4940-11200)	1.4 (0.9-2.0)	182000 (114000-269000)	33.1 (20.8-49.0)	441 (265-668)	0.1 (0.0-0.1)	10400 (6130-15900)	1.9 (1.1-2.9)
Escherichia coli	Aminopenicillin	31900 (20500-47000)	5.8 (3.7-8.6)	768000 (491000-1160000)	139.9 (89.5-210.7)	360 (218-563)	0.1 (0.0-0.1)	8440 (5040-13400)	1.5 (0.9-2.4)
Escherichia coli	Beta Lactam/Beta-lactamase inhibitors	24900 (16100-36800)	4.5 (2.9-6.7)	599000 (380000-902000)	109.1 (69.2-164.3)	691 (428-1050)	0.1 (0.1-0.2)	16100 (9970-25000)	2.9 (1.8-4.6)
Escherichia coli	Carbapenem	6980 (4330-10600)	1.3 (0.8-1.9)	170000 (104000-260000)	31.0 (19.0-47.3)	1450 (765-2450)	0.3 (0.1-0.4)	35000 (18500-60300)	6.4 (3.4-11.0)
Escherichia coli	Fluoroquinolones	21500 (13900-31900)	3.9 (2.5-5.8)	521000 (327000-781000)	94.9 (59.6-142.3)	2160 (1320-3220)	0.4 (0.2-0.6)	52100 (31200-79000)	9.5 (5.7-14.4)
Escherichia coli	Third-generation cephalosporins	23000 (14700-33500)	4.2 (2.7-6.1)	558000 (351000-831000)	101.6 (63.9-151.4)	2790 (1180-5160)	0.5 (0.2-0.9)	67500 (28900-125000)	12.3 (5.3-22.7)
Escherichia coli	Trimethoprim-Sulfamethoxazole	24800 (16000-36600)	4.5 (2.9-6.7)	596000 (379000-897000)	108.5 (69.0-163.4)	1370 (825-2100)	0.2 (0.2-0.4)	32300 (19300-50800)	5.9 (3.5-9.3)
Escherichia coli	Resistance to one or more antibiotics	33800 (21800-49700)	6.2 (4.0-9.1)	812000 (517000-1220000)	147.9 (94.2-222.1)	9260 (5700-14300)	1.7 (1.0-2.6)	222000 (136000-347000)	40.4 (24.7-63.3)
Group A *Streptococcus*	Macrolide	433 (186-893)	0.1 (0.0-0.2)	12700 (6130-24400)	2.3 (1.1-4.5)	42 (0-172)	0.0 (0.0-0.0)	1210 (0-4420)	0.2 (0.0-0.8)
Group A *Streptococcus*	Resistance to one or more antibiotics	433 (186-893)	0.1 (0.0-0.2)	12700 (6130-24400)	2.3 (1.1-4.5)	42 (0-172)	0.0 (0.0-0.0)	1210 (0-4420)	0.2 (0.0-0.8)
Group B *Streptococcus*	Fluoroquinolones	779 (479-1200)	0.1 (0.1-0.2)	23400 (14500-35800)	4.3 (2.6-6.5)	121 (19-295)	0.0 (0.0-0.1)	3580 (587-8570)	0.7 (0.1-1.6)
Group B *Streptococcus*	Macrolide	4760 (3060-7210)	0.9 (0.6-1.3)	139000 (88700-214000)	25.4 (16.2-39.1)	480 (0-1360)	0.1 (0.0-0.2)	14000 (0-40000)	2.6 (0.0-7.3)
Group B *Streptococcus*	Penicillin	48 (29-76)	0.0 (0.0-0.0)	1530 (934-2410)	0.3 (0.2-0.4)	13 (3-30)	0.0 (0.0-0.0)	402 (84-897)	0.1 (0.0-0.2)
Group B *Streptococcus*	Resistance to one or more antibiotics	4930 (3170-7450)	0.9 (0.6-1.4)	145000 (92500-222000)	26.3 (16.9-40.5)	614 (0-1510)	0.1 (0.0-0.3)	18000 (0-43700)	3.3 (0.0-8.0)
Haemophilus influenzae	Aminopenicillin	431 (312-578)	0.1 (0.1-0.1)	12900 (9290-17500)	2.4 (1.7-3.2)	68 (6-133)	0.0 (0.0-0.0)	2000 (244-3850)	0.4 (0.0-0.7)
Haemophilus influenzae	Third-generation cephalosporins	160 (113-222)	0.0 (0.0-0.0)	5070 (3620-7020)	0.9 (0.7-1.3)	47 (20-83)	0.0 (0.0-0.0)	1470 (676-2560)	0.3 (0.1-0.5)
Haemophilus influenzae	Resistance to one or more antibiotics	524 (390-695)	0.1 (0.1-0.1)	15900 (11700-21100)	2.9 (2.1-3.8)	115 (44-198)	0.0 (0.0-0.0)	3470 (1450-5960)	0.6 (0.3-1.1)
Klebsiella pneumoniae	Aminoglycosides	10600 (6830-15800)	1.9 (1.2-2.9)	263000 (168000-397000)	47.9 (30.5-72.3)	844 (497-1370)	0.2 (0.1-0.2)	20900 (12100-34300)	3.8 (2.2-6.3)
Klebsiella pneumoniae	Beta Lactam/Beta-lactamase inhibitors	24200 (15800-35400)	4.4 (2.9-6.4)	591000 (381000-881000)	107.6 (69.3-160.5)	294 (139-542)	0.1 (0.0-0.1)	6950 (3240-13000)	1.3 (0.6-2.4)
Klebsiella pneumoniae	Carbapenem	11200 (7020-16600)	2.0 (1.3-3.0)	267000 (164000-404000)	48.7 (29.9-73.6)	2650 (1540-4180)	0.5 (0.3-0.8)	63100 (36500-99500)	11.5 (6.7-18.1)
Klebsiella pneumoniae	Fluoroquinolones	17100 (11100-25200)	3.1 (2.0-4.6)	415000 (262000-623000)	75.7 (47.7-113.5)	1230 (699-2010)	0.2 (0.1-0.4)	30100 (17100-50000)	5.5 (3.1-9.1)
Klebsiella pneumoniae	Third-generation cephalosporins	24200 (15900-35900)	4.4 (2.9-6.5)	595000 (376000-906000)	108.3 (68.6-165.1)	2520 (959-4780)	0.5 (0.2-0.9)	62900 (23900-120000)	11.5 (4.3-21.9)
Klebsiella pneumoniae	Trimethoprim-Sulfamethoxazole	20100 (13100-29800)	3.7 (2.4-5.4)	494000 (315000-745000)	90.1 (57.4-135.7)	1000 (478-1800)	0.2 (0.1-0.3)	24600 (11400-44200)	4.5 (2.1-8.1)
Klebsiella pneumoniae	Resistance to one or more antibiotics	27600 (18100-40400)	5.0 (3.3-7.4)	676000 (436000-1010000)	123.1 (79.3-184.7)	8550 (5300-13200)	1.6 (1.0-2.4)	209000 (128000-325000)	38.0 (23.3-59.1)
Morganella spp.	Fluoroquinolones	98 (45-172)	0.0 (0.0-0.0)	1970 (902-3520)	0.4 (0.2-0.6)	17 (5-37)	0.0 (0.0-0.0)	343 (106-749)	0.1 (0.0-0.1)
Morganella spp.	Fourth-generation cephalosporins	27 (12-48)	0.0 (0.0-0.0)	551 (248-984)	0.1 (0.0-0.2)	4 (1-10)	0.0 (0.0-0.0)	92 (28-211)	0.0 (0.0-0.0)
Morganella spp.	Third-generation cephalosporins	37 (17-67)	0.0 (0.0-0.0)	742 (339-1370)	0.1 (0.1-0.2)	4 (1-10)	0.0 (0.0-0.0)	84 (26-195)	0.0 (0.0-0.0)
Morganella spp.	Resistance to one or more antibiotics	111 (51-194)	0.0 (0.0-0.0)	2230 (1020-3960)	0.4 (0.2-0.7)	26 (10-53)	0.0 (0.0-0.0)	520 (201-1050)	0.1 (0.0-0.2)
Mycobacterium tuberculosis	Extensive drug resistance in TB	70 (25-151)	0.0 (0.0-0.0)	2550 (888-5720)	0.5 (0.2-1.0)	43 (15-92)	0.0 (0.0-0.0)	1470 (518-3240)	0.3 (0.1-0.6)
Mycobacterium tuberculosis	Isoniazid mono-resistance	353 (233-526)	0.1 (0.0-0.1)	13900 (9440-20500)	2.5 (1.7-3.7)	56 (0-191)	0.0 (0.0-0.0)	1910 (0-6370)	0.3 (0.0-1.2)
Mycobacterium tuberculosis	Multi-drug resistance excluding extensive drug resistance in TB	938 (364-2020)	0.2 (0.1-0.4)	36000 (13500-79600)	6.6 (2.5-14.5)	557 (47-1560)	0.1 (0.0-0.3)	19100 (1510-52100)	3.5 (0.3-9.5)
Mycobacterium tuberculosis	Rifampicin mono-resistance	92 (60-145)	0.0 (0.0-0.0)	4000 (2580-6270)	0.7 (0.5-1.1)	26 (4-54)	0.0 (0.0-0.0)	991 (236-2030)	0.2 (0.0-0.4)
Mycobacterium tuberculosis	Resistance to one or more antibiotics	1450 (805-2630)	0.3 (0.1-0.5)	56500 (31300-102000)	10.3 (5.7-18.6)	682 (138-1740)	0.1 (0.0-0.3)	23400 (4680-59600)	4.3 (0.9-10.8)
Neisseria gonorrhoeae	Fluoroquinolones	--	--	3730 (2190-6130)	0.7 (0.4-1.1)	--	--	356 (97-730)	0.1 (0.0-0.1)
Neisseria gonorrhoeae	Third-generation cephalosporins	--	--	239 (86-553)	0.0 (0.0-0.1)	--	--	46 (12-128)	0.0 (0.0-0.0)
Neisseria gonorrhoeae	Resistance to one or more antibiotics	--	--	3780 (2210-6200)	0.7 (0.4-1.1)	--	--	402 (131-788)	0.1 (0.0-0.1)
Proteus spp.	Aminoglycosides	1890 (1120-2910)	0.3 (0.2-0.5)	43200 (25000-68000)	7.9 (4.6-12.4)	81 (9-181)	0.0 (0.0-0.0)	1860 (212-4180)	0.3 (0.0-0.8)
Proteus spp.	Aminopenicillin	4080 (2480-6180)	0.7 (0.5-1.1)	90500 (53200-142000)	16.5 (9.7-25.9)	50 (0-156)	0.0 (0.0-0.0)	1080 (0-3390)	0.2 (0.0-0.6)
Proteus spp.	Fluoroquinolones	2510 (1510-3810)	0.5 (0.3-0.7)	56600 (32900-88000)	10.3 (6.0-16.0)	158 (66-299)	0.0 (0.0-0.1)	3520 (1470-6660)	0.6 (0.3-1.2)
Proteus spp.	Third-generation cephalosporins	2340 (1420-3530)	0.4 (0.3-0.6)	52600 (31000-81700)	9.6 (5.6-14.9)	419 (124-844)	0.1 (0.0-0.2)	9390 (2850-19000)	1.7 (0.5-3.5)
Proteus spp.	Trimethoprim-Sulfamethoxazole	3080 (1900-4740)	0.6 (0.3-0.9)	68700 (40900-108000)	12.5 (7.4-19.7)	104 (0-236)	0.0 (0.0-0.0)	2310 (5-5240)	0.4 (0.0-1.0)
Proteus spp.	Resistance to one or more antibiotics	4450 (2740-6770)	0.8 (0.5-1.2)	98800 (58800-154000)	18.0 (10.7-28.0)	813 (384-1370)	0.1 (0.1-0.2)	18200 (8650-31600)	3.3 (1.6-5.7)
Pseudomonas aeruginosa	Aminoglycosides	6140 (3890-9130)	1.1 (0.7-1.7)	154000 (95300-234000)	28.0 (17.4-42.7)	158 (0-406)	0.0 (0.0-0.1)	4030 (0-10500)	0.7 (0.0-1.9)
Pseudomonas aeruginosa	Anti-pseudomonal penicillin/Beta-Lactamase inhibitors	7700 (4800-11400)	1.4 (0.9-2.1)	189000 (116000-285000)	34.5 (21.1-51.9)	543 (310-910)	0.1 (0.1-0.2)	13100 (7430-22400)	2.4 (1.4-4.1)
Pseudomonas aeruginosa	Carbapenem	9520 (6170-14100)	1.7 (1.1-2.6)	234000 (148000-354000)	42.6 (26.9-64.6)	1680 (1010-2700)	0.3 (0.2-0.5)	41300 (24100-67300)	7.5 (4.4-12.3)
Pseudomonas aeruginosa	Fluoroquinolones	8950 (5790-13400)	1.6 (1.1-2.4)	220000 (140000-336000)	40.0 (25.5-61.2)	945 (562-1520)	0.2 (0.1-0.3)	23300 (13500-38100)	4.3 (2.5-6.9)
Pseudomonas aeruginosa	Fourth-generation cephalosporins	7170 (4680-10600)	1.3 (0.9-1.9)	176000 (112000-263000)	32.0 (20.4-47.8)	202 (108-342)	0.0 (0.0-0.1)	4850 (2540-8300)	0.9 (0.5-1.5)
Pseudomonas aeruginosa	Third-generation cephalosporins	11900 (7700-17700)	2.2 (1.4-3.2)	292000 (183000-442000)	53.3 (33.3-80.5)	794 (344-1410)	0.1 (0.1-0.3)	19100 (8270-33900)	3.5 (1.5-6.2)
Pseudomonas aeruginosa	Resistance to one or more antibiotics	16800 (11000-24900)	3.1 (2.0-4.5)	410000 (260000-620000)	74.7 (47.3-112.9)	4330 (2540-6980)	0.8 (0.5-1.3)	106000 (60900-171000)	19.3 (11.1-31.2)
Salmonella Paratyphi	Fluoroquinolones	20 (7-44)	0.0 (0.0-0.0)	1410 (499-3070)	0.3 (0.1-0.6)	4 (1-11)	0.0 (0.0-0.0)	282 (36-737)	0.1 (0.0-0.1)
Salmonella Paratyphi	Multi-drug resistance in Salmonella Typhi and Paratyphi	1 (0-1)	0.0 (0.0-0.0)	44 (15-100)	0.0 (0.0-0.0)	0 (0-0)	0.0 (0.0-0.0)	4 (0-12)	0.0 (0.0-0.0)
Salmonella Paratyphi	Resistance to one or more antibiotics	20 (7-44)	0.0 (0.0-0.0)	1420 (503-3100)	0.3 (0.1-0.6)	4 (1-11)	0.0 (0.0-0.0)	286 (41-744)	0.1 (0.0-0.1)
Salmonella Typhi	Fluoroquinolones	1070 (542-1880)	0.2 (0.1-0.3)	62100 (30000-110000)	11.3 (5.5-20.1)	214 (31-513)	0.0 (0.0-0.1)	12300 (1820-29600)	2.2 (0.3-5.4)
Salmonella Typhi	Multi-drug resistance in Salmonella Typhi and Paratyphi	402 (211-704)	0.1 (0.0-0.1)	23800 (11900-43200)	4.3 (2.2-7.9)	47 (0-130)	0.0 (0.0-0.0)	2760 (0-7590)	0.5 (0.0-1.4)
Salmonella Typhi	Resistance to one or more antibiotics	1360 (733-2270)	0.2 (0.1-0.4)	79600 (40800-138000)	14.5 (7.4-25.2)	261 (60-555)	0.0 (0.0-0.1)	15100 (3580-32900)	2.8 (0.7-6.0)
Non-typhoidal *Salmonella*	Fluoroquinolones	42 (22-77)	0.0 (0.0-0.0)	2710 (935-6870)	0.5 (0.2-1.3)	9 (1-21)	0.0 (0.0-0.0)	410 (65-1050)	0.1 (0.0-0.2)
Non-typhoidal *Salmonella*	Resistance to one or more antibiotics	42 (22-77)	0.0 (0.0-0.0)	2710 (935-6870)	0.5 (0.2-1.3)	9 (1-21)	0.0 (0.0-0.0)	410 (65-1050)	0.1 (0.0-0.2)
Serratia spp.	Aminoglycosides	436 (242-716)	0.1 (0.0-0.1)	11900 (6610-19400)	2.2 (1.2-3.5)	26 (0-71)	0.0 (0.0-0.0)	716 (0-1940)	0.1 (0.0-0.4)
Serratia spp.	Anti-pseudomonal penicillin/Beta-Lactamase inhibitors	442 (242-751)	0.1 (0.0-0.1)	11900 (6500-20400)	2.2 (1.2-3.7)	88 (41-164)	0.0 (0.0-0.0)	2380 (1110-4450)	0.4 (0.2-0.8)
Serratia spp.	Carbapenem	360 (200-591)	0.1 (0.0-0.1)	9650 (5240-15700)	1.8 (1.0-2.9)	98 (46-182)	0.0 (0.0-0.0)	2630 (1220-4890)	0.5 (0.2-0.9)
Serratia spp.	Fluoroquinolones	329 (178-565)	0.1 (0.0-0.1)	8870 (4770-15400)	1.6 (0.9-2.8)	35 (6-81)	0.0 (0.0-0.0)	960 (187-2200)	0.2 (0.0-0.4)
Serratia spp.	Fourth-generation cephalosporins	762 (429-1240)	0.1 (0.1-0.2)	20300 (11400-34100)	3.7 (2.1-6.2)	77 (32-156)	0.0 (0.0-0.0)	2050 (839-4220)	0.4 (0.2-0.8)
Serratia spp.	Third-generation cephalosporins	936 (511-1530)	0.2 (0.1-0.3)	25200 (13600-41500)	4.6 (2.5-7.6)	62 (20-136)	0.0 (0.0-0.0)	1680 (543-3650)	0.3 (0.1-0.7)
Serratia spp.	Resistance to one or more antibiotics	1520 (867-2490)	0.3 (0.2-0.5)	40900 (23000-67000)	7.4 (4.2-12.2)	387 (198-674)	0.1 (0.0-0.1)	10400 (5240-18100)	1.9 (1.0-3.3)
Shigella spp.	Fluoroquinolones	86 (31-185)	0.0 (0.0-0.0)	9140 (3230-21900)	1.7 (0.6-4.0)	18 (2-48)	0.0 (0.0-0.0)	1260 (56-3530)	0.2 (0.0-0.6)
Shigella spp.	Resistance to one or more antibiotics	86 (31-185)	0.0 (0.0-0.0)	9140 (3230-21900)	1.7 (0.6-4.0)	18 (2-48)	0.0 (0.0-0.0)	1260 (56-3530)	0.2 (0.0-0.6)
Staphylococcus aureus	Fluoroquinolones	10700 (7120-15600)	2.0 (1.3-2.8)	274000 (179000-405000)	49.9 (32.6-73.8)	467 (194-837)	0.1 (0.0-0.2)	11800 (4960-21700)	2.2 (0.9-4.0)

Staphylococcus aureus	Macrolide	3120 (2030-4390)	339.6 (220.8-477.6)	278000 (181000-391000)	30218.5 (19639.3-42476.0)	117 (42-211)	12.7 (4.6-23.0)	10400 (3750-18800)	1134.5 (407.2-2041.8)	1970 (1500-2600)	18.0 (13.6-23.6)	175000 (133000-230000)	1590.2 (1205.4-2093.7)	74 (31-128)	0.7 (0.3-1.2)	6540 (2740-11300)	59.5 (24.9-103.0)
Staphylococcus aureus	Methicillin	2850 (1850-4050)	309.5 (201.5-440.0)	253000 (165000-360000)	27540.1 (17932.1-39141.1)	741 (325-1310)	80.5 (35.4-141.9)	65900 (29000-116000)	7162.9 (3148.3-12621.3)	1770 (1350-2370)	16.1 (12.2-21.5)	157000 (119000-210000)	1428.5 (1085.2-1906.1)	459 (212-759)	4.2 (1.9-6.9)	40600 (18800-67100)	369.4 (170.7-610.7)
Staphylococcus aureus	Trimethoprim-Sulfamethoxazole	1090 (707-1530)	119.0 (76.8-165.8)	97400 (62900-136000)	10588.3 (6835.5-14755.3)	95 (47-155)	10.3 (5.1-16.8)	8450 (4160-13700)	918.3 (452.1-1493.0)	687 (515-913)	6.2 (4.7-8.3)	60800 (45600-80800)	553.3 (414.7-735.0)	59 (30-93)	0.5 (0.3-0.8)	5260 (2680-8210)	47.8 (24.4-74.6)
Staphylococcus aureus	Vancomycin	86 (53-129)	9.4 (5.8-14.0)	7700 (4740-11500)	836.4 (515.5-1249.4)	21 (10-39)	2.2 (1.1-4.2)	1840 (889-3450)	199.5 (96.6-375.2)	45 (31-66)	0.4 (0.3-0.6)	4010 (2700-5890)	36.5 (24.6-53.6)	11 (6-20)	0.1 (0.1-0.2)	977 (491-1750)	8.9 (4.5-15.9)
Staphylococcus aureus	Resistance to one or more antibiotics	3930 (2540-5540)	426.9 (275.7-602.0)	350000 (226000-493000)	37986.5 (24518.1-53544.7)	1020 (566-1660)	110.4 (61.5-180.0)	90400 (50300-147000)	9823.2 (5472.3-16006.1)	2450 (1850-3250)	22.3 (16.8-29.6)	217000 (164000-288000)	1972.1 (1487.6-2617.9)	631 (369-969)	5.7 (3.4-8.8)	55900 (32700-85800)	508.2 (297.4-780.2)
Streptococcus pneumoniae	Beta Lactam/Beta-lactamase inhibitors	660 (422-976)	71.7 (45.8-106.1)	58700 (37500-86800)	6385.1 (4079.3-9436.0)	6 (0-16)	0.6 (0.0-1.7)	511 (12-1430)	55.6 (1.3-154.9)	1420 (1030-1910)	12.9 (9.4-17.3)	126000 (91600-169000)	1145.6 (832.9-1533.7)	13 (0-35)	0.1 (0.0-0.3)	1130 (23-3080)	10.3 (0.2-28.0)
Streptococcus pneumoniae	Carbapenem	1590 (1020-2310)	172.9 (110.4-251.2)	142000 (90400-206000)	15390.7 (9822.0-22399.1)	329 (139-595)	35.7 (15.1-64.7)	29300 (12400-53000)	3179.6 (1344.8-5758.1)	3300 (2460-4320)	30.0 (22.4-39.3)	292000 (218000-383000)	2657.5 (1983.1-3479.4)	674 (322-1170)	6.1 (2.9-10.7)	59700 (28500-104000)	542.7 (259.4-942.9)
Streptococcus pneumoniae	Fluoroquinolones	690 (451-1020)	75.0 (49.1-110.5)	61500 (40200-90500)	6680.5 (4365.5-9837.9)	78 (18-170)	8.5 (1.9-18.5)	6920 (1580-15200)	752.1 (171.4-1647.4)	1730 (1220-2390)	15.7 (11.1-21.8)	153000 (108000-212000)	1389.3 (980.6-1924.3)	196 (48-406)	1.8 (0.4-3.7)	17400 (4250-35900)	157.9 (38.6-326.5)
Streptococcus pneumoniae	Macrolide	1960 (1260-2840)	213.1 (137.4-308.8)	175000 (113000-253000)	18967.7 (12252.7-27509.3)	69 (0-184)	7.5 (0.0-20.0)	6150 (0-16300)	668.1 (0.0-1776.2)	3890 (2940-5030)	35.4 (26.8-45.8)	344000 (260000-445000)	3132.7 (2369.0-4049.3)	136 (0-340)	1.2 (0.0-3.1)	12000 (0-30000)	109.1 (0.0-273.0)
Streptococcus pneumoniae	Penicillin	2090 (1340-3030)	227.1 (146.0-329.2)	186000 (119000-270000)	20217.8 (12985.0-29308.8)	112 (56-197)	12.2 (6.0-21.4)	9960 (4950-17500)	1082.2 (537.5-1903.6)	4320 (3220-5650)	39.3 (29.3-51.4)	382000 (285000-499000)	3477.1 (2594.2-4543.3)	227 (115-381)	2.1 (1.0-3.5)	20100 (10200-33700)	182.6 (92.3-306.7)
Streptococcus pneumoniae	Third-generation cephalosporins	398 (247-594)	43.3 (26.8-64.5)	35400 (21900-52800)	3852.1 (2384.3-5742.7)	3 (1-6)	0.3 (0.1-0.6)	225 (74-526)	24.5 (8.0-57.1)	789 (556-1060)	7.2 (5.1-9.7)	69900 (49200-93900)	635.5 (447.9-854.2)	4 (1-10)	0.0 (0.0-0.1)	371 (126-867)	3.4 (1.1-7.9)
Streptococcus pneumoniae	Trimethoprim-Sulfamethoxazole	2780 (1790-3920)	302.1 (194.6-425.6)	247000 (159000-349000)	26893.2 (17319.5-37909.0)	219 (32-452)	23.8 (3.4-49.1)	19500 (2880-40200)	2114.0 (312.6-4365.2)	5660 (4320-7300)	51.5 (39.3-66.4)	501000 (382000-646000)	4555.4 (3478.9-5871.5)	438 (62-822)	4.0 (0.6-7.5)	38800 (5520-72700)	352.6 (50.2-661.3)
Streptococcus pneumoniae	Resistance to one or more antibiotics	3530 (2300-4950)	383.5 (250.0-537.7)	314000 (205000-441000)	34142.4 (22255.9-47881.2)	814 (486-1220)	88.5 (52.8-132.3)	72500 (43300-108000)	7875.9 (4701.1-11773.5)	7230 (5650-9200)	65.8 (51.4-83.6)	640000 (500000-814000)	5819.8 (4549.5-7401.8)	1690 (1150-2350)	15.4 (10.5-21.4)	149000 (102000-208000)	1358.5 (927.5-1889.5)

Staphylococcus aureus	Macrolide	791 (549-1120)	1.7 (1.1-2.4)	68900 (47700-97300)	144.0 (99.7-203.5)	29 (11-53)	0.1 (0.0-0.1)	2570 (1020-4560)	5.4 (2.1-9.5)	26200 (17900-37300)	4.8 (3.3-6.8)	672000 (448000-975000)	122.3 (81.5-177.5)	966 (366-1740)	0.2 (0.1-0.3)	24700 (9420-45100)	4.5 (1.7-8.2)
Staphylococcus aureus	Methicillin	736 (508-1040)	1.5 (1.1-2.2)	64100 (44600-90100)	134.1 (93.4-188.4)	191 (86-329)	0.4 (0.2-0.7)	16600 (7480-28500)	34.7 (15.7-59.6)	29100 (20000-41800)	5.3 (3.7-7.6)	721000 (482000-1050000)	131.3 (87.8-191.4)	7780 (3460-13200)	1.4 (0.6-2.4)	191000 (84400-330000)	34.7 (15.4-60.2)
Staphylococcus aureus	Trimethoprim-Sulfamethoxazole	273 (185-392)	0.6 (0.4-0.8)	23700 (16000-33900)	49.6 (33.5-71.0)	23 (11-38)	0.0 (0.0-0.1)	2020 (986-3290)	4.2 (2.1-6.9)	7500 (5100-10800)	1.4 (0.9-2.0)	201000 (134000-297000)	36.6 (24.5-54.1)	626 (314-1020)	0.1 (0.1-0.2)	16800 (8440-28000)	3.1 (1.5-5.1)
Staphylococcus aureus	Vancomycin	20 (13-29)	0.0 (0.0-0.1)	1740 (1150-2550)	3.6 (2.4-5.3)	5 (2-9)	0.0 (0.0-0.0)	419 (207-767)	0.9 (0.4-1.6)	572 (383-849)	0.1 (0.1-0.2)	15000 (9810-22800)	2.7 (1.8-4.2)	148 (72-282)	0.0 (0.0-0.1)	3830 (1870-7330)	0.7 (0.3-1.3)
Staphylococcus aureus	Resistance to one or more antibiotics	996 (690-1410)	2.1 (1.4-2.9)	86600 (60200-123000)	181.2 (126.0-257.1)	261 (145-413)	0.5 (0.3-0.9)	22600 (12800-35800)	47.3 (26.9-74.8)	36000 (24800-51000)	6.6 (4.5-9.3)	904000 (606000-1320000)	164.7 (110.3-241.0)	9990 (5360-16300)	1.8 (1.0-3.0)	248000 (131000-410000)	45.1 (23.9-74.7)
Streptococcus pneumoniae	Beta Lactam/Beta-lactamase inhibitors	530 (350-766)	1.1 (0.7-1.6)	45900 (30300-66300)	96.0 (63.4-138.7)	4 (0-13)	0.0 (0.0-0.0)	385 (10-1140)	0.8 (0.0-2.4)	3810 (2720-5250)	0.7 (0.5-1.0)	121000 (87500-168000)	22.1 (15.9-30.6)	27 (1-69)	0.0 (0.0-0.0)	878 (42-2240)	0.2 (0.0-0.4)
Streptococcus pneumoniae	Carbapenem	1280 (896-1770)	2.7 (1.9-3.7)	111000 (78000-153000)	232.2 (163.2-319.6)	265 (122-471)	0.6 (0.3-1.0)	23000 (10700-40800)	48.1 (22.3-85.4)	10100 (7430-13500)	1.8 (1.4-2.5)	312000 (230000-419000)	56.9 (42.0-76.2)	2170 (1040-3680)	0.4 (0.2-0.7)	67000 (32300-113000)	12.2 (5.9-20.6)
Streptococcus pneumoniae	Fluoroquinolones	577 (335-927)	1.2 (0.7-1.9)	49900 (29000-80000)	104.3 (60.6-167.3)	65 (13-144)	0.1 (0.0-0.3)	5620 (1120-12400)	11.8 (2.3-25.9)	1940 (1390-2680)	0.4 (0.3-0.5)	68300 (49800-95500)	12.4 (9.1-17.4)	205 (49-439)	0.0 (0.0-0.1)	7260 (1770-15600)	1.3 (0.3-2.8)
Streptococcus pneumoniae	Macrolide	1520 (1090-2090)	3.2 (2.3-4.4)	132000 (94700-182000)	276.5 (198.0-379.9)	54 (0-139)	0.1 (0.0-0.3)	4630 (0-11900)	9.7 (0.0-25.0)	13000 (9800-17300)	2.4 (1.8-3.2)	402000 (302000-532000)	73.1 (55.1-96.8)	472 (0-1220)	0.1 (0.0-0.2)	14200 (0-37300)	2.6 (0.0-6.8)
Streptococcus pneumoniae	Penicillin	1670 (1180-2370)	3.5 (2.5-5.0)	145000 (102000-205000)	303.1 (213.6-429.1)	89 (46-152)	0.2 (0.1-0.3)	7660 (3970-13100)	16.0 (8.3-27.4)	13500 (10200-18000)	2.5 (1.9-3.3)	414000 (314000-552000)	75.3 (57.2-100.5)	742 (394-1210)	0.1 (0.1-0.2)	22300 (11900-36500)	4.1 (2.2-6.6)
Streptococcus pneumoniae	Third-generation cephalosporins	313 (213-441)	0.7 (0.4-0.9)	27200 (18500-38200)	56.8 (38.7-80.0)	2 (1-4)	0.0 (0.0-0.0)	148 (50-334)	0.3 (0.1-0.7)	3040 (2200-4150)	0.6 (0.4-0.8)	94300 (68100-129000)	17.2 (12.4-23.4)	38 (15-77)	0.0 (0.0-0.0)	1240 (473-2550)	0.2 (0.1-0.5)
Streptococcus pneumoniae	Trimethoprim-Sulfamethoxazole	2130 (1500-2940)	4.5 (3.1-6.2)	184000 (130000-255000)	385.9 (272.0-533.9)	165 (23-347)	0.3 (0.0-0.7)	14300 (1990-29800)	29.8 (4.2-62.4)	15700 (12100-20500)	2.9 (2.2-3.7)	490000 (374000-647000)	89.3 (68.2-117.9)	1230 (178-2430)	0.2 (0.0-0.4)	37900 (5650-74500)	6.9 (1.0-13.6)
Streptococcus pneumoniae	Resistance to one or more antibiotics	2750 (1970-3750)	5.8 (4.1-7.8)	238000 (170000-325000)	498.6 (356.2-679.4)	644 (404-928)	1.3 (0.8-1.9)	55700 (35100-80200)	116.4 (73.3-167.8)	20800 (16000-27100)	3.8 (2.9-4.9)	647000 (493000-854000)	117.9 (89.8-155.5)	4880 (3270-6780)	0.9 (0.6-1.2)	151000 (102000-211000)	27.5 (18.6-38.5)

Table S18. Deaths and DALYs (in counts and age-specific rates) associated with and attributable to bacterial antimicrobial resistance by pathogen–drug combination, by age group, 2019, south Asia

Pathogen	Antibiotic Class	Neonatal: Associated with resistance: Deaths: Counts	Neonatal: Associated with resistance: Deaths: Rate per 100 000	Neonatal: Associated with resistance: DALYs: Counts	Neonatal: Associated with resistance: DALYs: Rate per 100 000	Neonatal: Attributable to resistance: Deaths: Counts	Neonatal: Attributable to resistance: Deaths: Rate per 100 000	Neonatal: Attributable to resistance: DALYs: Counts	Neonatal: Attributable to resistance: DALYs: Rate per 100 000	Post Neonatal: Associated with resistance: Deaths: Counts	Post Neonatal: Associated with resistance: Deaths: Rate per 100 000	Post Neonatal: Associated with resistance: DALYs: Counts	Post Neonatal: Associated with resistance: DALYs: Rate per 100 000	Post Neonatal: Attributable to resistance: Deaths: Counts	Post Neonatal: Attributable to resistance: Deaths: Rate per 100 000	Post Neonatal: Attributable to resistance: DALYs: Counts	Post Neonatal: Attributable to resistance: DALYs: Rate per 100 000
All pathogens	Resistance to one or more antibiotics	207000 (147000-285000)	8211.0 (5838.0-11294.4)	18500000 (13100000-25400000)	732677.0 (520682.0-1006273.0)	55400 (38800-76700)	2196.5 (1539.6-3041.0)	4940000 (3460000-6840000)	195813.6 (137145.1-271125.1)	68000 (52500-88800)	227.0 (175.1-296.6)	6020000 (4650000-7870000)	20110.0 (15508.9-26273.4)	17500 (13000-23500)	58.5 (43.6-78.4)	1550000 (1160000-2080000)	5182.8 (3856.7-6935.7)
Acinetobacter baumannii	Aminoglycosides	8580 (5340-13000)	340.2 (211.9-514.1)	764000 (476000-1150000)	30288.3 (18860.5-45757.6)	354 (0-878)	14.0 (0.0-34.8)	31600 (121-78200)	1252.3 (4.8-3103.1)	1400 (908-2020)	4.7 (3.0-6.7)	124000 (80300-178000)	413.4 (268.2-595.0)	57 (0-134)	0.2 (0.0-0.4)	5080 (5-11800)	17.0 (0.0-39.5)
Acinetobacter baumannii	Anti-pseudomonal penicillin/Beta-Lactamase inhibitors	10600 (6600-15800)	420.4 (261.7-626.3)	944000 (588000-1410000)	37429.7 (23326.7-55840.7)	170 (76-317)	6.8 (3.0-12.6)	15200 (6800-28200)	601.6 (269.7-1117.1)	1740 (1130-2550)	5.8 (3.8-8.5)	154000 (100000-225000)	512.6 (334.8-752.0)	28 (13-52)	0.1 (0.0-0.2)	2490 (1130-4560)	8.3 (3.8-15.2)
Acinetobacter baumannii	Beta Lactam/Beta-lactamase inhibitors	9440 (5860-14000)	374.2 (232.3-554.0)	840000 (522000-1240000)	33318.3 (20688.3-49333.0)	8 (2-17)	0.3 (0.1-0.7)	695 (187-1480)	27.6 (7.4-58.6)	1550 (1010-2270)	5.2 (3.4-7.6)	138000 (89000-201000)	459.0 (297.1-671.6)	1 (0-3)	0.0 (0.0-0.0)	124 (30-271)	0.4 (0.1-0.9)
Acinetobacter baumannii	Carbapenem	9840 (6150-14700)	390.3 (244.1-583.7)	876000 (548000-1310000)	34747.5 (21736.2-51983.1)	1600 (841-2710)	63.3 (33.3-107.4)	142000 (74900-241000)	5638.7 (2969.7-9555.7)	1610 (1050-2330)	5.4 (3.5-7.8)	142000 (92800-206000)	475.6 (309.7-689.3)	261 (142-436)	0.9 (0.5-1.5)	23100 (12500-38500)	77.2 (41.8-128.7)
Acinetobacter baumannii	Fluoroquinolones	10600 (6630-15900)	419.4 (262.9-629.5)	941000 (590000-1410000)	37337.7 (23396.3-56042.9)	1330 (668-2280)	52.8 (26.5-90.3)	118000 (59400-202000)	4695.7 (2357.8-8022.2)	1730 (1130-2520)	5.8 (3.8-8.4)	153000 (100000-223000)	511.6 (334.2-743.8)	218 (116-382)	0.7 (0.4-1.3)	19300 (10300-33800)	64.5 (34.3-112.8)
Acinetobacter baumannii	Fourth-generation cephalosporins	10700 (6730-16000)	426.2 (266.8-634.4)	957000 (599000-1420000)	37946.9 (23741.8-56466.8)	51 (17-105)	2.0 (0.7-4.2)	4540 (1550-9320)	180.1 (61.6-369.7)	1760 (1150-2570)	5.9 (3.9-8.6)	156000 (102000-227000)	519.6 (341.2-758.8)	8 (3-17)	0.0 (0.0-0.1)	750 (270-1490)	2.5 (0.9-5.0)
Acinetobacter baumannii	Third-generation cephalosporins	10900 (6780-16300)	431.7 (268.9-648.2)	969000 (603000-1450000)	38430.9 (23926.3-57692.7)	84 (31-163)	3.3 (1.2-6.5)	7470 (2720-14600)	296.3 (107.8-577.2)	1780 (1160-2600)	6.0 (3.9-8.7)	158000 (103000-230000)	526.7 (343.0-767.2)	14 (5-28)	0.0 (0.0-0.1)	1240 (481-2450)	4.1 (1.6-8.2)
Acinetobacter baumannii	Resistance to one or more antibiotics	11200 (6970-16800)	444.6 (276.6-665.9)	998000 (621000-1490000)	39579.1 (24634.1-59271.6)	3600 (2150-5590)	142.6 (85.3-221.7)	320000 (191000-497000)	12692.3 (7593.5-19727.1)	1840 (1200-2690)	6.1 (4.0-9.0)	163000 (106000-237000)	542.7 (354.4-792.8)	589 (365-923)	2.0 (1.2-3.1)	52100 (32300-81600)	174.0 (107.9-272.4)
Citrobacter spp.	Aminoglycosides	942 (517-1610)	37.3 (20.5-63.7)	83700 (46000-143000)	3319.6 (1824.9-5659.4)	42 (0-114)	1.7 (0.0-4.5)	3780 (0-10100)	149.8 (0.0-402.4)	209 (106-377)	0.7 (0.4-1.3)	18500 (9350-33300)	61.6 (31.2-111.2)	9 (0-26)	0.0 (0.0-0.1)	837 (0-2290)	2.8 (0.0-7.6)
Citrobacter spp.	Anti-pseudomonal penicillin/Beta-Lactamase inhibitors	1050 (571-1780)	41.6 (22.6-70.5)	93200 (50700-158000)	3695.2 (2012.4-6271.3)	111 (47-220)	4.4 (1.9-8.7)	9910 (4180-19600)	393.1 (165.9-776.9)	234 (118-421)	0.8 (0.4-1.4)	20700 (10400-37200)	69.1 (34.8-124.2)	25 (10-49)	0.1 (0.0-0.2)	2210 (850-4350)	7.4 (2.8-14.5)
Citrobacter spp.	Carbapenem	973 (523-1670)	38.6 (20.7-66.3)	86500 (46500-149000)	3431.2 (1842.8-5897.1)	198 (86-384)	7.8 (3.4-15.2)	17600 (7670-34200)	696.5 (304.1-1354.7)	215 (108-389)	0.7 (0.4-1.3)	19000 (9560-34400)	63.5 (31.9-114.9)	44 (17-90)	0.1 (0.1-0.3)	3860 (1540-7940)	12.9 (5.1-26.5)
Citrobacter spp.	Fluoroquinolones	1650 (911-2810)	65.6 (36.1-111.4)	147000 (81000-250000)	5828.6 (3213.3-9904.5)	224 (91-437)	8.9 (3.6-17.3)	19900 (8110-38800)	789.8 (321.8-1539.7)	368 (187-659)	1.2 (0.6-2.2)	32600 (16500-58300)	108.7 (55.2-194.6)	50 (20-102)	0.2 (0.1-0.3)	4420 (1730-8990)	14.7 (5.8-30.0)
Citrobacter spp.	Fourth-generation cephalosporins	1620 (888-2760)	64.2 (35.2-109.4)	144000 (78900-245000)	5707.7 (3131.0-9722.6)	83 (32-171)	3.3 (1.3-6.8)	7410 (2820-15200)	294.1 (111.8-602.9)	359 (178-644)	1.2 (0.6-2.1)	31800 (15800-56900)	106.0 (52.6-190.1)	18 (6-39)	0.1 (0.0-0.1)	1630 (559-3410)	5.5 (1.9-11.4)
Citrobacter spp.	Third-generation cephalosporins	1840 (1020-3100)	73.1 (40.4-122.8)	164000 (90500-275000)	6498.7 (3589.9-10919.5)	56 (20-110)	2.2 (0.8-4.4)	4960 (1780-9790)	196.8 (70.4-388.2)	410 (208-730)	1.4 (0.7-2.4)	36300 (18400-64500)	121.1 (61.4-215.5)	12 (4-26)	0.0 (0.0-0.1)	1100 (394-2270)	3.7 (1.3-7.6)
Citrobacter spp.	Resistance to one or more antibiotics	2350 (1320-3990)	93.3 (52.2-158.1)	209000 (117000-354000)	8297.4 (4641.3-14053.1)	715 (369-1220)	28.3 (14.6-48.6)	63500 (32800-109000)	2519.9 (1301.6-4317.3)	523 (262-925)	1.7 (0.9-3.1)	46300 (23200-81800)	154.5 (77.3-273.1)	159 (78-293)	0.5 (0.3-1.0)	14100 (6930-25900)	46.9 (23.1-86.4)
Enterobacter spp.	Aminoglycosides	4520 (2850-6940)	179.4 (112.9-275.2)	403000 (253000-618000)	15968.7 (10039.7-24508.9)	274 (71-541)	10.9 (2.8-21.5)	24500 (6320-48100)	970.0 (250.7-1908.7)	425 (285-619)	1.4 (1.0-2.1)	37600 (25200-54800)	125.4 (84.2-182.8)	26 (7-48)	0.1 (0.0-0.2)	2270 (643-4260)	7.6 (2.1-14.2)
Enterobacter spp.	Anti-pseudomonal penicillin/Beta-Lactamase inhibitors	7350 (4770-11300)	291.4 (189.1-447.9)	654000 (424000-1000000)	25928.0 (16831.6-39838.3)	520 (282-857)	20.6 (11.2-34.0)	46300 (25100-76300)	1837.2 (996.6-3024.5)	719 (474-1040)	2.4 (1.6-3.5)	63600 (41900-91900)	212.4 (140.0-306.9)	51 (29-83)	0.2 (0.1-0.3)	4530 (2530-7310)	15.1 (8.5-24.4)
Enterobacter spp.	Carbapenem	6900 (4420-10800)	273.5 (175.3-427.9)	614000 (393000-960000)	24338.6 (15602.1-38077.4)	1690 (954-2730)	67.1 (37.9-108.5)	150000 (84900-243000)	5965.1 (3366.5-9639.8)	657 (437-949)	2.2 (1.5-3.2)	58100 (38600-84000)	194.1 (128.9-280.3)	161 (94-248)	0.5 (0.3-0.8)	14200 (8320-21900)	47.4 (27.8-73.2)
Enterobacter spp.	Fluoroquinolones	5660 (3640-8780)	224.3 (144.4-348.3)	503000 (324000-781000)	19959.9 (12855.7-30972.2)	567 (294-987)	22.5 (11.7-39.2)	50400 (26200-87700)	1998.5 (1038.7-3480.2)	541 (356-792)	1.8 (1.2-2.6)	47900 (31500-70100)	159.8 (105.1-233.9)	54 (29-90)	0.2 (0.1-0.3)	4800 (2560-7960)	16.0 (8.5-26.6)
Enterobacter spp.	Fourth-generation cephalosporins	9620 (6240-14400)	381.7 (247.7-572.3)	856000 (556000-1280000)	33967.1 (22041.5-50935.6)	333 (140-618)	13.2 (5.5-24.5)	29700 (12500-55000)	1177.1 (494.0-2179.6)	923 (629-1310)	3.1 (2.1-4.4)	81700 (55700-115000)	272.6 (185.8-385.5)	31 (14-56)	0.1 (0.0-0.2)	2770 (1280-5000)	9.2 (4.3-16.7)
Enterobacter spp.	Trimethoprim-Sulfamethoxazole	3290 (1940-5160)	130.3 (76.9-204.5)	292000 (172000-459000)	11594.8 (6837.7-18202.6)	132 (0-309)	5.2 (0.0-12.3)	11800 (0-27500)	466.5 (0.0-1088.9)	319 (204-477)	1.1 (0.7-1.6)	28300 (18000-42200)	94.3 (60.2-140.8)	13 (0-29)	0.0 (0.0-0.1)	1140 (0-2580)	3.8 (0.0-8.6)
Enterobacter spp.	Resistance to one or more antibiotics	12300 (8010-18500)	487.2 (317.8-733.8)	1090000 (713000-1650000)	43356.6 (28294.4-65300.7)	3520 (2250-5350)	139.5 (89.1-212.0)	313000 (200000-475000)	12414.5 (7922.4-18854.0)	1170 (799-1650)	3.9 (2.7-5.5)	104000 (70800-146000)	346.8 (236.2-487.8)	336 (221-485)	1.1 (0.7-1.6)	29700 (19600-42900)	99.2 (65.3-143.2)
Enterococcus faecalis	Fluoroquinolones	5210 (3210-8130)	206.5 (127.4-322.4)	463000 (285000-723000)	18360.0 (11321.9-28657.6)	1270 (619-2210)	50.4 (24.6-87.6)	113000 (55000-196000)	4478.7 (2182.8-7779.9)	358 (219-555)	1.2 (0.7-1.9)	31800 (19400-49200)	106.0 (64.7-164.2)	87 (39-151)	0.3 (0.1-0.5)	7730 (3490-13400)	25.8 (11.6-44.6)
Enterococcus faecalis	Vancomycin	588 (344-953)	23.3 (13.6-37.8)	52300 (30600-84800)	2072.6 (1213.3-3361.5)	139 (53-290)	5.5 (2.1-11.5)	12400 (4730-25800)	491.9 (187.5-1021.9)	42 (25-68)	0.1 (0.1-0.2)	3710 (2170-6030)	12.4 (7.3-20.1)	10 (4-21)	0.0 (0.0-0.1)	880 (353-1860)	2.9 (1.2-6.2)
Enterococcus faecalis	Resistance to one or more antibiotics	5250 (3240-8200)	208.3 (128.4-325.2)	467000 (288000-729000)	18522.2 (11414.7-28906.7)	1410 (728-2420)	55.9 (28.9-95.9)	125000 (64700-215000)	4970.5 (2566.1-8523.4)	362 (221-558)	1.2 (0.7-1.9)	32100 (19600-49500)	107.0 (65.5-165.4)	97 (49-165)	0.3 (0.2-0.6)	8610 (4360-14600)	28.7 (14.6-48.8)
Enterococcus faecium	Fluoroquinolones	2340 (1430-3690)	92.9 (56.8-146.3)	208000 (127000-328000)	8256.3 (5046.4-13004.8)	454 (128-873)	18.0 (5.1-34.6)	40300 (11400-77600)	1599.8 (453.7-3076.4)	283 (158-474)	0.9 (0.5-1.6)	25000 (13900-42000)	83.6 (46.6-140.1)	54 (16-114)	0.2 (0.1-0.4)	4810 (1390-10100)	16.1 (4.6-33.7)
Enterococcus faecium	Vancomycin	564 (331-928)	22.4 (13.1-36.8)	50100 (29400-82500)	1987.8 (1166.8-3272.9)	131 (59-244)	5.2 (2.4-9.7)	11600 (5290-21700)	461.4 (209.9-861.7)	74 (41-126)	0.2 (0.1-0.4)	6590 (3600-11100)	22.0 (12.0-37.2)	17 (7-35)	0.1 (0.0-0.1)	1530 (645-3070)	5.1 (2.2-10.2)
Enterococcus faecium	Resistance to one or more antibiotics	2350 (1440-3700)	93.3 (57.1-146.7)	209000 (128000-329000)	8294.3 (5073.8-13047.4)	585 (286-1020)	23.2 (11.3-40.6)	52000 (25400-91000)	2061.2 (1007.4-3611.2)	284 (158-476)	0.9 (0.5-1.6)	25200 (14000-42200)	84.0 (46.7-140.7)	72 (32-137)	0.2 (0.1-0.5)	6340 (2850-12100)	21.2 (9.5-40.4)
Other enterococci	Fluoroquinolones	3480 (1950-5710)	138.0 (77.4-226.4)	309000 (173000-507000)	12265.3 (6876.5-20120.0)	675 (95-1410)	26.8 (3.8-56.0)	60000 (8490-126000)	2378.3 (336.6-4978.0)	326 (180-550)	1.1 (0.6-1.8)	28900 (16000-48700)	96.4 (53.2-162.6)	63 (11-132)	0.2 (0.0-0.4)	5600 (935-11700)	18.7 (3.1-39.1)
Other enterococci	Vancomycin	203 (112-336)	8.1 (4.5-13.3)	18100 (9980-29900)	716.8 (396.0-1186.3)	37 (16-78)	1.5 (0.6-3.1)	3270 (1450-6920)	129.8 (57.6-274.6)	19 (10-33)	0.1 (0.0-0.1)	1690 (900-2930)	5.6 (3.0-9.8)	3 (1-7)	0.0 (0.0-0.0)	306 (132-628)	1.0 (0.4-2.1)
Other enterococci	Resistance to one or more antibiotics	3480 (1950-5710)	138.1 (77.5-226.7)	310000 (174000-508000)	12276.4 (6885.0-20145.4)	712 (153-1470)	28.2 (6.1-58.4)	63200 (13600-131000)	2508.0 (538.1-5188.2)	326 (180-551)	1.1 (0.6-1.8)	28900 (16000-48800)	96.4 (53.3-162.9)	67 (15-136)	0.2 (0.1-0.5)	5910 (1370-12100)	19.7 (4.6-40.3)
Escherichia coli	Aminoglycosides	8610 (5920-12400)	341.7 (235.0-492.1)	770000 (529000-1110000)	30521.8 (20982.6-44038.4)	483 (305-721)	19.1 (12.1-28.6)	43100 (27200-64400)	1709.8 (1078.7-2553.5)	2520 (1740-3630)	8.4 (5.8-12.1)	224000 (154000-323000)	749.0 (514.5-1078.3)	141 (92-215)	0.5 (0.3-0.7)	12600 (8200-19200)	41.9 (27.4-64.0)
Escherichia coli	Aminopenicillin	27300 (19000-38600)	1083.3 (755.0-1530.3)	2440000 (1700000-3440000)	96761.7 (67364.8-136545.8)	90 (55-142)	3.6 (2.2-5.6)	8050 (4910-12700)	319.3 (194.9-503.0)	8010 (5480-11700)	26.7 (18.3-38.9)	712000 (487000-1040000)	2377.9 (1624.6-3468.0)	26 (16-42)	0.1 (0.1-0.1)	2350 (1420-3750)	7.9 (4.7-12.5)
Escherichia coli	Beta Lactam/Beta-lactamase inhibitors	22700 (15600-32600)	902.2 (619.4-1294.7)	2030000 (1390000-2910000)	80579.5 (55263.1-115529.6)	279 (177-420)	11.1 (7.0-16.7)	24900 (15800-37500)	987.7 (627.9-1488.4)	6670 (4570-9620)	22.2 (15.2-32.1)	593000 (406000-858000)	1978.3 (1356.8-2863.5)	81 (52-126)	0.3 (0.2-0.4)	7230 (4590-11200)	24.1 (15.3-37.5)
Escherichia coli	Carbapenem	11900 (8100-17100)	472.4 (321.2-676.4)	1060000 (723000-1520000)	42192.6 (28664.5-60373.7)	2460 (1370-3890)	97.7 (54.3-154.4)	220000 (122000-347000)	8717.9 (4847.7-13765.0)	3510 (2430-4990)	11.7 (8.1-16.7)	312000 (216000-443000)	1040.1 (720.7-1479.2)	725 (408-1170)	2.4 (1.4-3.9)	64400 (36200-104000)	214.8 (120.8-345.5)
Escherichia coli	Fluoroquinolones	24300 (16900-34300)	964.8 (668.6-1360.2)	2170000 (1500000-3070000)	86172.5 (59658.3-121777.0)	2570 (1730-3680)	102.0 (68.4-146.0)	230000 (154000-329000)	9105.2 (6099.2-13054.9)	7130 (4880-10300)	23.8 (16.3-34.4)	634000 (433000-919000)	2117.2 (1444.0-3066.5)	754 (509-1110)	2.5 (1.7-3.7)	67000 (45200-98600)	223.5 (150.8-329.2)
Escherichia coli	Third-generation cephalosporins	24400 (16800-34500)	967.1 (667.2-1369.1)	2180000 (1500000-3080000)	86378.1 (59602.6-122346.8)	2320 (1050-4200)	91.9 (41.7-166.5)	207000 (94400-374000)	8204.3 (3745.8-14852.8)	7160 (4890-10400)	23.9 (16.3-34.6)	636000 (435000-923000)	2123.5 (1452.0-3080.2)	677 (293-1280)	2.3 (1.0-4.3)	60200 (26200-113000)	200.9 (87.4-377.5)
Escherichia coli	Trimethoprim-Sulfamethoxazole	16700 (11500-23900)	661.8 (457.3-948.5)	1490000 (1030000-2130000)	59097.6 (40795.2-84615.3)	792 (508-1200)	31.4 (20.2-47.6)	70500 (45200-107000)	2797.8 (1792.1-4235.6)	4890 (3330-7180)	16.3 (11.1-24.0)	435000 (295000-640000)	1452.2 (985.8-2137.8)	232 (148-351)	0.8 (0.5-1.2)	20600 (13100-31400)	68.7 (43.9-104.7)
Escherichia coli	Resistance to one or more antibiotics	29100 (20300-41200)	1154.7 (806.0-1634.7)	2600000 (1810000-3680000)	103146.2 (71957.7-145930.7)	9000 (5840-13000)	356.9 (231.5-515.8)	803000 (521000-1160000)	31842.1 (20672.9-45970.3)	8540 (5860-12400)	28.5 (19.5-41.3)	759000 (520000-1110000)	2534.4 (1734.5-3689.6)	2640 (1730-3950)	8.8 (5.8-13.2)	234000 (153000-351000)	781.8 (511.4-1170.8)
Group A *Streptococcus*	Macrolide	353 (210-578)	14.0 (8.3-22.9)	31400 (18600-51400)	1244.9 (739.5-2038.6)	34 (0-111)	1.3 (0.0-4.4)	3000 (0-9850)	118.9 (0.0-390.7)	100 (58-157)	0.3 (0.2-0.5)	8920 (5200-14000)	29.8 (17.4-46.6)	10 (0-31)	0.0 (0.0-0.1)	850 (0-2730)	2.8 (0.0-9.1)
Group A *Streptococcus*	Resistance to one or more antibiotics	353 (210-578)	14.0 (8.3-22.9)	31400 (18600-51400)	1244.9 (739.5-2038.6)	34 (0-111)	1.3 (0.0-4.4)	3000 (0-9850)	118.9 (0.0-390.7)	100 (58-157)	0.3 (0.2-0.5)	8920 (5200-14000)	29.8 (17.4-46.6)	10 (0-31)	0.0 (0.0-0.1)	850 (0-2730)	2.8 (0.0-9.1)
Group B *Streptococcus*	Fluoroquinolones	10200 (6570-15100)	402.7 (260.5-597.0)	908000 (588000-1350000)	36009.1 (23307.2-53355.0)	1720 (283-3970)	68.1 (11.2-157.4)	153000 (25700-354000)	6067.8 (1019.9-14021.7)	1020 (718-1410)	3.4 (2.4-4.7)	90300 (63600-125000)	301.6 (212.1-417.4)	172 (27-376)	0.6 (0.1-1.3)	15200 (2400-33200)	50.7 (8.0-110.9)
Group B *Streptococcus*	Macrolide	15400 (10200-22100)	609.3 (403.8-877.2)	1370000 (909000-1970000)	54489.9 (36071.7-78320.4)	1400 (0-3680)	55.4 (0.0-145.9)	125000 (0-328000)	4951.0 (0.0-13000.1)	1600 (1110-2210)	5.3 (3.7-7.4)	142000 (98500-196000)	472.5 (328.8-653.8)	146 (0-384)	0.5 (0.0-1.3)	12900 (0-33900)	43.2 (0.0-113.3)
Group B *Streptococcus*	Penicillin	265 (169-393)	10.5 (6.7-15.6)	23700 (15100-35200)	938.9 (599.1-1396.8)	67 (14-147)	2.7 (0.6-5.8)	5990 (1240-13100)	237.7 (49.1-519.8)	29 (19-41)	0.1 (0.1-0.1)	2520 (1650-3610)	8.4 (5.5-12.0)	7 (1-16)	0.0 (0.0-0.1)	633 (126-1370)	2.1 (0.4-4.6)
Group B *Streptococcus*	Resistance to one or more antibiotics	19600 (13200-27700)	777.8 (523.8-1099.1)	1750000 (1180000-2480000)	69558.1 (46868.3-98345.2)	3180 (690-6390)	126.2 (27.4-253.3)	284000 (62700-568000)	11256.6 (2487.2-22539.4)	2030 (1450-2780)	6.8 (4.8-9.3)	179000 (128000-246000)	598.5 (428.0-822.7)	325 (73-602)	1.1 (0.2-2.0)	28700 (6470-53300)	96.0 (21.6-177.8)
Haemophilus influenzae	Aminopenicillin	413 (249-640)	16.4 (9.9-25.4)	37200 (22400-57900)	1477.3 (890.0-2294.8)	57 (4-122)	2.2 (0.2-4.8)	5080 (430-11000)	201.6 (17.0-435.2)	265 (166-417)	0.9 (0.6-1.4)	23400 (14700-36900)	78.2 (49.2-123.3)	36 (3-79)	0.1 (0.0-0.3)	3220 (240-7020)	10.7 (0.8-23.4)
Haemophilus influenzae	Third-generation cephalosporins	510 (314-746)	20.2 (12.4-29.6)	46100 (28200-67200)	1826.5 (1120.3-2664.8)	151 (58-275)	6.0 (2.3-10.9)	13600 (5210-24700)	538.2 (206.5-981.4)	325 (227-457)	1.1 (0.8-1.5)	28800 (20100-40400)	96.0 (67.0-134.9)	96 (40-169)	0.3 (0.1-0.6)	8490 (3550-14900)	28.3 (11.9-49.9)
Haemophilus influenzae	Resistance to one or more antibiotics	810 (515-1180)	32.1 (20.4-46.8)	73100 (46600-107000)	2900.4 (1846.6-4236.5)	207 (91-362)	8.2 (3.6-14.4)	18700 (8250-32600)	739.8 (327.1-1292.0)	517 (364-732)	1.7 (1.2-2.4)	45700 (32300-64800)	152.7 (107.7-216.5)	132 (58-227)	0.4 (0.2-0.8)	11700 (5160-20100)	39.1 (17.2-67.1)
Klebsiella pneumoniae	Aminoglycosides	27600 (18900-38700)	1096.7 (748.2-1535.7)	2470000 (1680000-3450000)	97782.8 (66686.9-136770.1)	2110 (1270-3190)	83.8 (50.4-126.3)	188000 (114000-284000)	7473.1 (4505.1-11257.9)	6930 (5130-9250)	23.1 (17.1-30.9)	613000 (454000-818000)	2046.2 (1514.7-2732.2)	530 (328-763)	1.8 (1.1-2.5)	46800 (29000-67400)	156.3 (96.9-225.1)
Klebsiella pneumoniae	Beta Lactam/Beta-lactamase inhibitors	35400 (23900-48600)	1402.6 (949.3-1927.9)	3150000 (2130000-4340000)	125052.5 (84563.2-171994.2)	395 (205-639)	15.7 (8.1-25.3)	35200 (18300-56800)	1397.0 (724.3-2254.3)	8860 (6590-11800)	29.6 (22.0-39.4)	784000 (583000-1040000)	2616.6 (1946.7-3483.3)	99 (53-167)	0.3 (0.2-0.6)	8800 (4680-14800)	29.4 (15.6-49.3)
Klebsiella pneumoniae	Carbapenem	27600 (18600-38800)	1096.4 (737.9-1539.6)	2470000 (1660000-3460000)	97749.4 (65712.7-137088.3)	6410 (4160-9380)	254.2 (165.2-372.0)	571000 (371000-835000)	22628.8 (14701.5-33107.9)	6920 (5240-9110)	23.1 (17.5-30.4)	612000 (464000-806000)	2043.4 (1547.3-2689.1)	1600 (1110-2240)	5.4 (3.7-7.5)	142000 (98000-198000)	473.6 (327.0-661.8)
Klebsiella pneumoniae	Fluoroquinolones	30400 (20900-42100)	1206.3 (827.7-1670.1)	2710000 (1860000-3760000)	107567.8 (73748.1-149142.5)	2050 (1200-3150)	81.1 (47.6-124.9)	183000 (107000-281000)	7254.6 (4259.1-11149.1)	7620 (5580-10300)	25.4 (18.6-34.2)	674000 (494000-907000)	2248.9 (1649.0-3028.4)	513 (310-773)	1.7 (1.0-2.6)	45400 (27500-68400)	151.5 (91.7-228.4)
Klebsiella pneumoniae	Third-generation cephalosporins	33200 (22700-46400)	1316.6 (899.6-1839.4)	2960000 (2020000-4140000)	117405.1 (80131.5-164190.1)	1350 (449-2770)	53.5 (17.8-109.9)	120000 (40100-246000)	4762.2 (1589.9-9776.4)	8320 (6230-11100)	27.8 (20.8-37.0)	736000 (551000-982000)	2455.5 (1840.2-3276.6)	339 (120-688)	1.1 (0.4-2.3)	30000 (10600-60800)	100.1 (35.3-202.9)
Klebsiella pneumoniae	Trimethoprim-Sulfamethoxazole	26700 (18300-37100)	1058.4 (724.2-1472.9)	2380000 (1630000-3310000)	94361.4 (64519.3-131227.7)	1130 (520-1960)	44.7 (20.6-77.8)	100000 (46500-174000)	3983.6 (1845.2-6920.7)	6680 (4970-8900)	22.3 (16.6-29.7)	591000 (440000-787000)	1972.5 (1468.4-2627.6)	282 (134-460)	0.9 (0.4-1.5)	25000 (11900-40700)	83.3 (39.6-135.8)
Klebsiella pneumoniae	Resistance to one or more antibiotics	38800 (26500-53900)	1540.4 (1049.6-2137.2)	3460000 (2360000-4810000)	137349.6 (93524.9-190757.4)	13400 (9180-18800)	533.0 (363.9-746.5)	1200000 (817000-1680000)	47499.4 (32419.8-66575.2)	9740 (7330-12900)	32.5 (24.5-42.9)	861000 (648000-1140000)	2874.5 (2164.3-3798.3)	3370 (2470-4600)	11.2 (8.2-15.4)	298000 (218000-407000)	994.2 (729.4-1357.7)
Morganella spp.	Fluoroquinolones	5 (2-12)	0.2 (0.1-0.5)	453 (161-1110)	18.0 (6.4-44.1)	1 (0-2)	0.0 (0.0-0.1)	71 (20-182)	2.8 (0.8-7.2)	11 (5-19)	0.0 (0.0-0.1)	949 (463-1720)	3.2 (1.5-5.7)	2 (1-4)	0.0 (0.0-0.0)	150 (57-315)	0.5 (0.2-1.1)
Morganella spp.	Fourth-generation cephalosporins	2 (1-6)	0.1 (0.0-0.2)	221 (77-549)	8.8 (3.0-21.8)	0 (0-1)	0.0 (0.0-0.0)	29 (6-91)	1.2 (0.2-3.6)	5 (2-9)	0.0 (0.0-0.0)	454 (218-843)	1.5 (0.7-2.8)	1 (0-2)	0.0 (0.0-0.0)	60 (15-146)	0.2 (0.0-0.5)
Morganella spp.	Third-generation cephalosporins	3 (1-6)	0.1 (0.0-0.3)	226 (78-577)	9.0 (3.1-22.9)	0 (0-1)	0.0 (0.0-0.0)	15 (4-45)	0.6 (0.1-1.8)	5 (3-10)	0.0 (0.0-0.0)	473 (225-845)	1.6 (0.7-2.8)	0 (0-1)	0.0 (0.0-0.0)	31 (9-72)	0.1 (0.0-0.2)
Morganella spp.	Resistance to one or more antibiotics	5 (2-13)	0.2 (0.1-0.5)	459 (163-1120)	18.2 (6.5-44.5)	1 (0-3)	0.1 (0.0-0.1)	115 (38-289)	4.6 (1.5-11.5)	11 (5-20)	0.0 (0.0-0.1)	961 (468-1730)	3.2 (1.6-5.8)	3 (1-5)	0.0 (0.0-0.0)	240 (110-457)	0.8 (0.4-1.5)
Mycobacterium tuberculosis	Extensive drug resistance in TB	--	--	0 (0-0)	0.0 (0.0-0.0)	--	--	0 (0-0)	0.0 (0.0-0.0)	37 (10-85)	0.1 (0.0-0.3)	3290 (929-7560)	11.0 (3.1-25.2)	23 (6-52)	0.1 (0.0-0.2)	2010 (562-4600)	6.7 (1.9-15.3)
Mycobacterium tuberculosis	Isoniazid mono-resistance	--	--	1 (1-2)	0.0 (0.0-0.1)	--	--	0 (0-0)	0.0 (0.0-0.0)	501 (309-796)	1.7 (1.0-2.7)	44500 (27600-70600)	148.6 (92.0-235.7)	79 (0-288)	0.3 (0.0-1.0)	7010 (0-25500)	23.4 (0.0-85.1)
Mycobacterium tuberculosis	Multi-drug resistance excluding extensive drug resistance in TB	--	--	1 (0-2)	0.0 (0.0-0.1)	--	--	0 (0-0)	0.0 (0.0-0.0)	722 (210-1560)	2.4 (0.7-5.2)	64000 (18700-139000)	213.6 (62.3-462.4)	424 (42-1130)	1.4 (0.1-3.8)	37500 (3740-100000)	125.0 (12.5-334.4)
Mycobacterium tuberculosis	Rifampicin mono-resistance	--	--	0 (0-0)	0.0 (0.0-0.0)	--	--	0 (0-0)	0.0 (0.0-0.0)	50 (24-92)	0.2 (0.1-0.3)	4400 (2150-8200)	14.7 (7.2-27.4)	14 (2-33)	0.0 (0.0-0.1)	1230 (197-2950)	4.1 (0.7-9.9)
Mycobacterium tuberculosis	Resistance to one or more antibiotics	--	--	2 (1-3)	0.1 (0.0-0.1)	--	--	0 (0-0)	0.0 (0.0-0.0)	1310 (728-2220)	4.4 (2.4-7.4)	116000 (64700-197000)	387.9 (216.1-656.7)	540 (108-1280)	1.8 (0.4-4.3)	47700 (9550-113000)	159.3 (31.9-378.0)
Neisseria gonorrhoeae	Fluoroquinolones	--	--	--	--	--	--	--	--	--	--	--	--	--	--	--	--
Neisseria gonorrhoeae	Third-generation cephalosporins	--	--	--	--	--	--	--	--	--	--	--	--	--	--	--	--
Neisseria gonorrhoeae	Resistance to one or more antibiotics	--	--	--	--	--	--	--	--	--	--	--	--	--	--	--	--
Proteus spp.	Aminoglycosides	398 (235-647)	15.8 (9.3-25.7)	35400 (20900-57600)	1404.7 (828.4-2282.7)	17 (2-38)	0.7 (0.1-1.5)	1520 (178-3400)	60.2 (7.1-135.0)	80 (50-123)	0.3 (0.2-0.4)	7130 (4440-10900)	23.8 (14.8-36.4)	3 (0-7)	0.0 (0.0-0.0)	306 (37-662)	1.0 (0.1-2.2)
Proteus spp.	Aminopenicillin	819 (484-1340)	32.5 (19.2-53.0)	72800 (43100-119000)	2886.9 (1707.9-4709.0)	13 (0-41)	0.5 (0.0-1.6)	1190 (0-3640)	47.1 (0.0-144.3)	167 (106-258)	0.6 (0.4-0.9)	14800 (9370-22900)	49.5 (31.3-76.6)	3 (0-8)	0.0 (0.0-0.0)	242 (0-724)	0.8 (0.0-2.4)
Proteus spp.	Fluoroquinolones	679 (397-1110)	26.9 (15.8-44.2)	60300 (35300-99100)	2392.9 (1401.5-3930.7)	49 (19-95)	1.9 (0.8-3.8)	4330 (1720-8480)	171.6 (68.2-336.5)	141 (89-217)	0.5 (0.3-0.7)	12500 (7860-19300)	41.8 (26.2-64.3)	10 (4-20)	0.0 (0.0-0.1)	905 (341-1810)	3.0 (1.1-6.0)
Proteus spp.	Third-generation cephalosporins	287 (165-475)	11.4 (6.5-18.8)	25600 (14700-42200)	1013.5 (581.2-1674.1)	53 (14-114)	2.1 (0.6-4.5)	4700 (1270-10100)	186.4 (50.3-401.9)	57 (35-89)	0.2 (0.1-0.3)	5080 (3080-7890)	16.9 (10.3-26.3)	10 (3-22)	0.0 (0.0-0.1)	930 (264-1960)	3.1 (0.9-6.5)
Proteus spp.	Trimethoprim-Sulfamethoxazole	525 (306-856)	20.8 (12.1-34.0)	46700 (27200-76100)	1850.5 (1079.3-3019.4)	16 (0-36)	0.6 (0.0-1.4)	1400 (0-3230)	55.3 (0.0-128.1)	107 (67-164)	0.4 (0.2-0.5)	9510 (5970-14600)	31.7 (19.9-48.8)	3 (0-7)	0.0 (0.0-0.0)	285 (0-614)	1.0 (0.0-2.0)
Proteus spp.	Resistance to one or more antibiotics	937 (552-1530)	37.2 (21.9-60.6)	83300 (49100-136000)	3304.8 (1948.1-5382.6)	148 (75-263)	5.9 (3.0-10.4)	13100 (6640-23400)	520.7 (263.2-926.3)	193 (122-296)	0.6 (0.4-1.0)	17100 (10800-26300)	57.1 (36.2-87.8)	30 (16-51)	0.1 (0.1-0.2)	2670 (1390-4550)	8.9 (4.7-15.2)
Pseudomonas aeruginosa	Aminoglycosides	8470 (5850-11900)	336.1 (231.9-470.0)	758000 (522000-1060000)	30054.2 (20709.3-42048.4)	215 (0-544)	8.5 (0.0-21.6)	19500 (161-48600)	772.7 (6.4-1928.3)	1660 (1240-2170)	5.5 (4.1-7.3)	147000 (109000-192000)	490.0 (365.3-642.4)	42 (0-98)	0.1 (0.0-0.3)	3720 (0-8670)	12.4 (0.0-28.9)
Pseudomonas aeruginosa	Anti-pseudomonal penicillin/Beta-Lactamase inhibitors	8980 (6060-12500)	356.1 (240.4-496.7)	802000 (541000-1120000)	31820.6 (21476.4-44340.0)	263 (135-442)	10.4 (5.3-17.5)	23400 (12000-39600)	929.6 (475.8-1570.4)	1730 (1300-2310)	5.8 (4.3-7.7)	153000 (115000-204000)	510.8 (384.8-681.6)	50 (26-83)	0.2 (0.1-0.3)	4380 (2290-7330)	14.6 (7.6-24.5)
Pseudomonas aeruginosa	Carbapenem	15500 (10800-21300)	615.0 (426.9-843.0)	1390000 (962000-1900000)	54982.0 (38152.0-75476.0)	2860 (1790-4250)	113.4 (71.1-168.4)	255000 (160000-380000)	10133.1 (6356.5-15054.0)	2990 (2220-3940)	10.0 (7.4-13.1)	265000 (196000-348000)	883.2 (655.5-1162.7)	550 (370-799)	1.8 (1.2-2.7)	48700 (32800-70700)	162.5 (109.4-235.9)
Pseudomonas aeruginosa	Fluoroquinolones	10600 (7190-14700)	419.6 (285.3-584.9)	946000 (643000-1320000)	37531.9 (25505.6-52311.0)	1080 (672-1640)	42.6 (26.6-65.1)	96600 (60500-147000)	3830.4 (2398.5-5827.0)	2050 (1510-2700)	6.8 (5.0-9.0)	182000 (133000-239000)	606.1 (445.3-798.8)	209 (136-308)	0.7 (0.5-1.0)	18500 (12000-27300)	61.7 (40.1-91.1)
Pseudomonas aeruginosa	Fourth-generation cephalosporins	14400 (9860-19800)	572.3 (391.2-786.6)	1290000 (880000-1770000)	51156.4 (34919.0-70335.8)	490 (285-795)	19.4 (11.3-31.5)	43800 (25400-71400)	1736.5 (1007.6-2832.6)	2760 (2070-3600)	9.2 (6.9-12.0)	244000 (183000-318000)	813.7 (611.8-1062.8)	92 (56-140)	0.3 (0.2-0.5)	8100 (4920-12400)	27.0 (16.4-41.4)
Pseudomonas aeruginosa	Third-generation cephalosporins	16000 (10800-22300)	632.8 (427.7-883.1)	1430000 (962000-1990000)	56531.2 (38145.1-78939.2)	631 (285-1090)	25.0 (11.3-43.4)	56300 (25400-97300)	2232.0 (1008.4-3859.6)	3060 (2240-4080)	10.2 (7.5-13.6)	271000 (198000-361000)	904.0 (661.4-1203.9)	119 (57-208)	0.4 (0.2-0.7)	10500 (5010-18400)	35.1 (16.7-61.5)
Pseudomonas aeruginosa	Resistance to one or more antibiotics	21000 (14400-28900)	833.0 (573.0-1147.2)	1880000 (1300000-2590000)	74450.6 (51416.6-102713.8)	5530 (3500-8440)	219.5 (138.8-334.9)	495000 (312000-756000)	19634.4 (12389.2-29971.8)	4030 (3010-5250)	13.4 (10.1-17.5)	356000 (266000-464000)	1189.3 (889.2-1550.1)	1060 (710-1560)	3.5 (2.4-5.2)	93900 (62800-138000)	313.3 (209.8-460.2)
Salmonella Paratyphi	Fluoroquinolones	--	--	0 (0-0)	0.0 (0.0-0.0)	--	--	0 (0-0)	0.0 (0.0-0.0)	10 (3-27)	0.0 (0.0-0.1)	898 (259-2420)	3.0 (0.9-8.1)	2 (0-6)	0.0 (0.0-0.0)	179 (23-563)	0.6 (0.1-1.9)
Salmonella Paratyphi	Multi-drug resistance in Salmonella Typhi and Paratyphi	--	--	0 (0-0)	0.0 (0.0-0.0)	--	--	0 (0-0)	0.0 (0.0-0.0)	0 (0-1)	0.0 (0.0-0.0)	42 (11-117)	0.1 (0.0-0.4)	0 (0-0)	0.0 (0.0-0.0)	3 (0-12)	0.0 (0.0-0.0)
Salmonella Paratyphi	Resistance to one or more antibiotics	--	--	0 (0-0)	0.0 (0.0-0.0)	--	--	0 (0-0)	0.0 (0.0-0.0)	10 (3-27)	0.0 (0.0-0.1)	903 (261-2430)	3.0 (0.9-8.1)	2 (0-6)	0.0 (0.0-0.0)	182 (26-571)	0.6 (0.1-1.9)
Salmonella Typhi	Fluoroquinolones	6760 (3470-11800)	268.1 (137.8-467.9)	601000 (309000-1050000)	23835.6 (12246.9-41595.2)	1290 (231-2930)	51.0 (9.2-116.3)	114000 (20500-261000)	4534.1 (814.2-10333.7)	805 (406-1410)	2.7 (1.4-4.7)	71200 (35900-125000)	237.7 (119.9-417.0)	153 (31-360)	0.5 (0.1-1.2)	13500 (2750-31800)	45.1 (9.2-106.2)
Salmonella Typhi	Multi-drug resistance in Salmonella Typhi and Paratyphi	2480 (1240-4460)	98.4 (49.0-176.8)	220000 (110000-396000)	8743.9 (4354.5-15710.8)	235 (0-683)	9.3 (0.0-27.1)	20900 (0-60700)	828.9 (0.0-2406.4)	294 (140-543)	1.0 (0.5-1.8)	26000 (12400-48100)	86.8 (41.3-160.4)	28 (0-79)	0.1 (0.0-0.3)	2440 (0-7010)	8.1 (0.0-23.4)
Salmonella Typhi	Resistance to one or more antibiotics	7610 (4030-12800)	301.7 (159.9-509.1)	676000 (358000-1140000)	26821.4 (14211.6-45258.9)	1520 (387-3210)	60.3 (15.4-127.4)	135000 (34400-285000)	5363.0 (1365.8-11318.9)	906 (465-1590)	3.0 (1.6-5.3)	80200 (41100-141000)	267.6 (137.2-469.1)	180 (48-389)	0.6 (0.2-1.3)	15900 (4230-34400)	53.2 (14.1-114.8)
Non-typhoidal *Salmonella*	Fluoroquinolones	4050 (2010-6800)	160.6 (79.7-269.7)	360000 (179000-605000)	14280.6 (7085.9-23981.9)	836 (134-1930)	33.2 (5.3-76.7)	74300 (11900-172000)	2948.5 (472.9-6816.4)	1350 (561-2700)	4.5 (1.9-9.0)	122000 (50400-245000)	407.4 (168.4-817.6)	279 (40-687)	0.9 (0.1-2.3)	24800 (3680-60600)	82.7 (12.3-202.2)
Non-typhoidal *Salmonella*	Resistance to one or more antibiotics	4050 (2010-6800)	160.6 (79.7-269.7)	360000 (179000-605000)	14280.6 (7085.9-23981.9)	836 (134-1930)	33.2 (5.3-76.7)	74300 (11900-172000)	2948.5 (472.9-6816.4)	1350 (561-2700)	4.5 (1.9-9.0)	122000 (50400-245000)	407.4 (168.4-817.6)	279 (40-687)	0.9 (0.1-2.3)	24800 (3680-60600)	82.7 (12.3-202.2)
Serratia spp.	Aminoglycosides	1310 (743-2130)	52.1 (29.5-84.3)	117000 (66100-189000)	4629.8 (2620.1-7492.3)	78 (0-219)	3.1 (0.0-8.7)	6930 (0-19500)	275.0 (0.0-771.5)	97 (52-165)	0.3 (0.2-0.6)	8620 (4600-14600)	28.8 (15.4-48.8)	6 (0-18)	0.0 (0.0-0.1)	512 (0-1610)	1.7 (0.0-5.4)
Serratia spp.	Anti-pseudomonal penicillin/Beta-Lactamase inhibitors	1040 (585-1760)	41.1 (23.2-69.8)	92200 (52000-156000)	3655.2 (2060.7-6206.6)	163 (72-329)	6.5 (2.8-13.1)	14500 (6360-29200)	574.0 (252.4-1160.0)	76 (40-132)	0.3 (0.1-0.4)	6740 (3550-11700)	22.5 (11.9-39.0)	12 (5-23)	0.0 (0.0-0.1)	1050 (441-2040)	3.5 (1.5-6.8)
Serratia spp.	Carbapenem	803 (465-1300)	31.8 (18.4-51.4)	71300 (41300-115000)	2829.8 (1639.0-4569.8)	212 (101-384)	8.4 (4.0-15.2)	18800 (8960-34100)	746.0 (355.6-1353.7)	59 (33-98)	0.2 (0.1-0.3)	5220 (2890-8710)	17.4 (9.6-29.1)	16 (7-30)	0.1 (0.0-0.1)	1370 (627-2660)	4.6 (2.1-8.9)
Serratia spp.	Fluoroquinolones	745 (424-1230)	29.5 (16.8-48.7)	66200 (37700-109000)	2625.5 (1497.0-4330.0)	76 (12-172)	3.0 (0.5-6.8)	6780 (1110-15300)	269.0 (43.9-606.1)	56 (29-93)	0.2 (0.1-0.3)	4940 (2590-8270)	16.5 (8.7-27.6)	6 (1-13)	0.0 (0.0-0.0)	503 (88-1180)	1.7 (0.3-4.0)
Serratia spp.	Fourth-generation cephalosporins	1290 (756-2110)	51.2 (30.0-83.6)	115000 (67200-187000)	4553.7 (2664.0-7430.0)	98 (33-199)	3.9 (1.3-7.9)	8690 (2900-17600)	344.8 (115.0-699.8)	97 (52-157)	0.3 (0.2-0.5)	8600 (4640-13900)	28.7 (15.5-46.3)	7 (3-15)	0.0 (0.0-0.1)	658 (228-1370)	2.2 (0.8-4.6)
Serratia spp.	Third-generation cephalosporins	1240 (710-2070)	49.2 (28.1-82.3)	110000 (63100-184000)	4374.2 (2502.0-7314.6)	29 (7-75)	1.1 (0.3-3.0)	2540 (629-6640)	100.7 (25.0-263.2)	93 (51-156)	0.3 (0.2-0.5)	8270 (4490-13800)	27.6 (15.0-46.0)	2 (0-5)	0.0 (0.0-0.0)	183 (42-467)	0.6 (0.1-1.6)
Serratia spp.	Resistance to one or more antibiotics	2790 (1650-4470)	110.5 (65.6-177.2)	248000 (147000-397000)	9824.5 (5828.7-15748.2)	655 (346-1120)	26.0 (13.7-44.2)	58200 (30800-99100)	2309.4 (1221.4-3931.5)	205 (113-334)	0.7 (0.4-1.1)	18100 (10000-29500)	60.5 (33.4-98.6)	48 (25-83)	0.2 (0.1-0.3)	4280 (2180-7370)	14.3 (7.3-24.6)
Shigella spp.	Fluoroquinolones	592 (184-1410)	23.5 (7.3-55.9)	52800 (16400-126000)	2092.8 (651.3-4980.8)	122 (13-355)	4.8 (0.5-14.1)	10900 (1180-31600)	430.5 (46.7-1254.5)	2190 (710-4840)	7.3 (2.4-16.2)	195000 (63600-431000)	651.1 (212.2-1439.7)	450 (51-1260)	1.5 (0.2-4.2)	40000 (4510-112000)	133.4 (15.0-374.4)
Shigella spp.	Resistance to one or more antibiotics	592 (184-1410)	23.5 (7.3-55.9)	52800 (16400-126000)	2092.8 (651.3-4980.8)	122 (13-355)	4.8 (0.5-14.1)	10900 (1180-31600)	430.5 (46.7-1254.5)	2190 (710-4840)	7.3 (2.4-16.2)	195000 (63600-431000)	651.1 (212.2-1439.7)	450 (51-1260)	1.5 (0.2-4.2)	40000 (4510-112000)	133.4 (15.0-374.4)
Staphylococcus aureus	Fluoroquinolones	20100 (14100-27800)	796.6 (559.5-1102.6)	1790000 (1260000-2480000)	71016.3 (49812.2-98240.2)	806 (331-1400)	31.9 (13.1-55.7)	71800 (29700-125000)	2845.8 (1176.6-4953.4)	7310 (5500-9670)	24.4 (18.4-32.3)	647000 (487000-856000)	2159.0 (1625.1-2856.3)	293 (129-487)	1.0 (0.4-1.6)	25900 (11400-43100)	86.6 (38.1-143.7)
Staphylococcus aureus	Macrolide	17100 (12100-23300)	679.6 (478.0-925.3)	1530000 (1070000-2080000)	60577.6 (42587.4-82438.7)	530 (202-926)	21.0 (8.0-36.7)	47200 (18000-82500)	1872.1 (714.5-3270.5)	6320 (4730-8410)	21.1 (15.8-28.1)	559000 (418000-745000)	1867.0 (1396.9-2486.6)	196 (79-346)	0.7 (0.3-1.2)	17400 (7020-30600)	58.1 (23.4-102.3)
Staphylococcus aureus	Methicillin	15200 (10600-21000)	603.0 (420.2-832.2)	1350000 (943000-1870000)	53739.4 (37420.9-74142.4)	3590 (1470-6300)	142.6 (58.3-249.9)	320000 (131000-560000)	12695.8 (5195.6-22223.7)	5590 (4110-7510)	18.7 (13.7-25.1)	495000 (364000-664000)	1652.5 (1214.0-2217.9)	1330 (560-2280)	4.4 (1.9-7.6)	117000 (49600-202000)	391.9 (165.7-672.7)

Pathogen	Antibiotic Class	1 to 4: Associated with resistance: Deaths: Counts	1 to 4: Associated with resistance: Deaths: Rate per 100 000	1 to 4: Associated with resistance: DALYs: Counts	1 to 4: Associated with resistance: DALYs: Rate per 100 000	1 to 4: Attributable to resistance: Deaths: Counts	1 to 4: Attributable to resistance: Deaths: Rate per 100 000	1 to 4: Attributable to resistance: DALYs: Counts	1 to 4: Attributable to resistance: DALYs: Rate per 100 000	5 and over: Associated with resistance: Deaths: Counts	5 and over: Associated with resistance: Deaths: Rate per 100 000	5 and over: Associated with resistance: DALYs: Counts	5 and over: Associated with resistance: DALYs: Rate per 100 000	5 and over: Attributable to resistance: Deaths: Counts	5 and over: Attributable to resistance: Deaths: Rate per 100 000	5 and over: Attributable to resistance: DALYs: Counts	5 and over: Attributable to resistance: DALYs: Rate per 100 000
All pathogens	Resistance to one or more antibiotics	45400 (34900-59100)	34.4 (26.4-44.8)	3940000 (3040000-5130000)	2989.7 (2303.5-3888.7)	11100 (8020-15000)	8.4 (6.1-11.4)	959000 (693000-1300000)	727.1 (525.1-983.6)	1070000 (773000-1430000)	65.0 (47.1-87.3)	31500000 (23300000-41700000)	1917.2 (1422.7-2541.1)	305000 (208000-438000)	18.6 (12.7-26.7)	8650000 (5740000-12500000)	527.4 (349.6-763.4)
Acinetobacter baumannii	Aminoglycosides	563 (355-859)	0.4 (0.3-0.7)	48600 (30600-74100)	36.8 (23.2-56.1)	23 (0-55)	0.0 (0.0-0.0)	2000 (7-4740)	1.5 (0.0-3.6)	84000 (48700-135000)	5.1 (3.0-8.2)	1810000 (1040000-2950000)	110.3 (63.3-179.6)	3480 (0-8890)	0.2 (0.0-0.5)	75500 (682-193000)	4.6 (0.0-11.8)
Acinetobacter baumannii	Anti-pseudomonal penicillin/Beta-Lactamase inhibitors	710 (442-1080)	0.5 (0.3-0.8)	61200 (38100-93500)	46.4 (28.9-70.9)	12 (5-22)	0.0 (0.0-0.0)	1010 (451-1910)	0.8 (0.3-1.4)	102000 (59300-162000)	6.2 (3.6-9.9)	2200000 (1260000-3560000)	133.9 (76.6-217.2)	1600 (634-3210)	0.1 (0.0-0.2)	34600 (13900-70100)	2.1 (0.8-4.3)
Acinetobacter baumannii	Beta Lactam/Beta-lactamase inhibitors	647 (406-987)	0.5 (0.3-0.7)	55800 (35000-85200)	42.3 (26.5-64.6)	1 (0-2)	0.0 (0.0-0.0)	70 (19-145)	0.1 (0.0-0.1)	88600 (51000-141000)	5.4 (3.1-8.6)	1920000 (1100000-3120000)	116.9 (67.0-190.0)	33 (9-71)	0.0 (0.0-0.0)	820 (218-1800)	0.0 (0.0-0.1)
Acinetobacter baumannii	Carbapenem	657 (409-991)	0.5 (0.3-0.8)	56600 (35300-85400)	42.9 (26.7-64.8)	106 (57-179)	0.1 (0.0-0.1)	9180 (4940-15400)	7.0 (3.7-11.7)	94700 (54500-152000)	5.8 (3.3-9.3)	2050000 (1160000-3330000)	124.7 (71.0-203.1)	15400 (7630-27500)	0.9 (0.5-1.7)	332000 (161000-596000)	20.2 (9.8-36.3)
Acinetobacter baumannii	Fluoroquinolones	710 (445-1080)	0.5 (0.3-0.8)	61200 (38400-92900)	46.4 (29.1-70.4)	90 (46-155)	0.1 (0.0-0.1)	7720 (3990-13300)	5.9 (3.0-10.1)	101000 (59000-161000)	6.2 (3.6-9.8)	2180000 (1250000-3520000)	133.2 (76.0-214.6)	12600 (6030-21500)	0.8 (0.4-1.3)	273000 (129000-472000)	16.6 (7.9-28.8)
Acinetobacter baumannii	Fourth-generation cephalosporins	718 (454-1080)	0.5 (0.3-0.8)	61900 (39100-93500)	46.9 (29.6-70.9)	3 (1-7)	0.0 (0.0-0.0)	288 (101-598)	0.2 (0.1-0.5)	104000 (60300-165000)	6.3 (3.7-10.1)	2230000 (1280000-3600000)	136.2 (78.0-219.3)	517 (174-1120)	0.0 (0.0-0.1)	11100 (3730-24300)	0.7 (0.2-1.5)
Acinetobacter baumannii	Third-generation cephalosporins	730 (457-1110)	0.6 (0.3-0.8)	63000 (39400-95500)	47.7 (29.8-72.4)	6 (2-12)	0.0 (0.0-0.0)	545 (209-1060)	0.4 (0.2-0.8)	104000 (60500-165000)	6.4 (3.7-10.1)	2250000 (1290000-3640000)	137.3 (78.8-221.8)	694 (237-1430)	0.0 (0.0-0.1)	15300 (5320-31800)	0.9 (0.3-1.9)
Acinetobacter baumannii	Resistance to one or more antibiotics	755 (474-1140)	0.6 (0.4-0.9)	65100 (40800-98300)	49.3 (30.9-74.5)	241 (145-376)	0.2 (0.1-0.3)	20800 (12600-32300)	15.8 (9.5-24.5)	107000 (62300-169000)	6.5 (3.8-10.3)	2310000 (1330000-3730000)	140.9 (80.9-227.3)	34300 (19200-56600)	2.1 (1.2-3.5)	741000 (415000-1250000)	45.2 (25.3-75.9)
Citrobacter spp.	Aminoglycosides	89 (43-155)	0.1 (0.0-0.1)	7620 (3740-13400)	5.8 (2.8-10.1)	4 (0-11)	0.0 (0.0-0.0)	344 (0-948)	0.3 (0.0-0.7)	3700 (2150-5940)	0.2 (0.1-0.4)	96300 (56500-156000)	5.9 (3.4-9.5)	167 (0-422)	0.0 (0.0-0.0)	4350 (0-11000)	0.3 (0.0-0.7)
Citrobacter spp.	Anti-pseudomonal penicillin/Beta-Lactamase inhibitors	102 (49-182)	0.1 (0.0-0.1)	8800 (4260-15600)	6.7 (3.2-11.9)	11 (4-24)	0.0 (0.0-0.0)	976 (380-2040)	0.7 (0.3-1.5)	3950 (2320-6330)	0.2 (0.1-0.4)	104000 (59800-168000)	6.3 (3.6-10.2)	392 (185-735)	0.0 (0.0-0.0)	10400 (4850-19700)	0.6 (0.3-1.2)
Citrobacter spp.	Carbapenem	82 (38-146)	0.1 (0.0-0.1)	7030 (3260-12600)	5.3 (2.5-9.5)	16 (6-33)	0.0 (0.0-0.0)	1420 (555-2870)	1.1 (0.4-2.2)	4470 (2590-7210)	0.3 (0.2-0.4)	115000 (65500-184000)	7.0 (4.0-11.2)	915 (432-1660)	0.1 (0.0-0.1)	23400 (11100-42100)	1.4 (0.7-2.6)
Citrobacter spp.	Fluoroquinolones	154 (74-272)	0.1 (0.1-0.2)	13200 (6400-23400)	10.0 (4.9-17.7)	21 (8-43)	0.0 (0.0-0.0)	1810 (695-3740)	1.4 (0.5-2.8)	6690 (3810-10700)	0.4 (0.2-0.7)	174000 (99700-283000)	10.6 (6.1-17.2)	899 (373-1700)	0.1 (0.0-0.1)	23400 (9860-44000)	1.4 (0.6-2.7)
Citrobacter spp.	Fourth-generation cephalosporins	144 (66-257)	0.1 (0.1-0.2)	12400 (5720-22100)	9.4 (4.3-16.8)	7 (2-15)	0.0 (0.0-0.0)	610 (200-1320)	0.5 (0.2-1.0)	6950 (4010-11100)	0.4 (0.2-0.7)	180000 (103000-288000)	10.9 (6.3-17.6)	378 (149-703)	0.0 (0.0-0.0)	9710 (3820-18100)	0.6 (0.2-1.1)
Citrobacter spp.	Third-generation cephalosporins	168 (82-298)	0.1 (0.1-0.2)	14500 (7110-25700)	11.0 (5.4-19.5)	5 (2-10)	0.0 (0.0-0.0)	417 (140-851)	0.3 (0.1-0.6)	7640 (4450-12400)	0.5 (0.3-0.8)	198000 (116000-322000)	12.1 (7.1-19.6)	249 (98-497)	0.0 (0.0-0.0)	6410 (2540-12900)	0.4 (0.2-0.8)
Citrobacter spp.	Resistance to one or more antibiotics	213 (104-380)	0.2 (0.1-0.3)	18400 (8930-32800)	13.9 (6.8-24.8)	65 (30-121)	0.0 (0.0-0.1)	5570 (2620-10400)	4.2 (2.0-7.9)	9850 (5730-15900)	0.6 (0.3-1.0)	255000 (147000-411000)	15.6 (9.0-25.0)	3000 (1650-5130)	0.2 (0.1-0.3)	77600 (42800-132000)	4.7 (2.6-8.1)
Enterobacter spp.	Aminoglycosides	201 (132-299)	0.2 (0.1-0.2)	17300 (11400-25800)	13.1 (8.7-19.5)	12 (3-24)	0.0 (0.0-0.0)	1040 (303-2030)	0.8 (0.2-1.5)	14100 (8570-22700)	0.9 (0.5-1.4)	367000 (218000-598000)	22.4 (13.3-36.4)	863 (242-1720)	0.1 (0.0-0.1)	22600 (6270-45300)	1.4 (0.4-2.8)
Enterobacter spp.	Anti-pseudomonal penicillin/Beta-Lactamase inhibitors	347 (231-496)	0.3 (0.2-0.4)	30000 (19900-42700)	22.7 (15.1-32.4)	24 (14-39)	0.0 (0.0-0.0)	2080 (1170-3330)	1.6 (0.9-2.5)	22700 (13500-34300)	1.4 (0.8-2.1)	589000 (346000-901000)	35.9 (21.1-54.9)	1670 (876-2770)	0.1 (0.1-0.2)	43300 (22400-72700)	2.6 (1.4-4.4)
Enterobacter spp.	Carbapenem	316 (210-451)	0.2 (0.2-0.3)	27300 (18100-38900)	20.7 (13.7-29.5)	77 (45-120)	0.1 (0.0-0.1)	6650 (3920-10300)	5.0 (3.0-7.8)	21200 (12500-32000)	1.3 (0.8-2.0)	551000 (323000-849000)	33.6 (19.7-51.7)	5200 (2750-8500)	0.3 (0.2-0.5)	135000 (71400-222000)	8.2 (4.4-13.6)
Enterobacter spp.	Fluoroquinolones	254 (167-382)	0.2 (0.1-0.3)	21900 (14400-32900)	16.6 (10.9-24.9)	25 (14-42)	0.0 (0.0-0.0)	2180 (1170-3630)	1.7 (0.9-2.7)	18000 (10700-28000)	1.1 (0.7-1.7)	466000 (274000-736000)	28.4 (16.7-44.9)	1830 (935-3230)	0.1 (0.1-0.2)	47000 (23900-83900)	2.9 (1.5-5.1)
Enterobacter spp.	Fourth-generation cephalosporins	439 (300-639)	0.3 (0.2-0.5)	37900 (25900-55100)	28.7 (19.6-41.7)	14 (6-26)	0.0 (0.0-0.0)	1220 (554-2220)	0.9 (0.4-1.7)	30200 (18100-46100)	1.8 (1.1-2.8)	783000 (462000-1210000)	47.7 (28.2-74.0)	1110 (475-2160)	0.1 (0.0-0.1)	28700 (12400-55900)	1.7 (0.8-3.4)
Enterobacter spp.	Trimethoprim-Sulfamethoxazole	154 (97-234)	0.1 (0.1-0.2)	13200 (8350-20100)	10.0 (6.3-15.3)	6 (0-14)	0.0 (0.0-0.0)	531 (0-1190)	0.4 (0.0-0.9)	10200 (5690-16800)	0.6 (0.3-1.0)	265000 (146000-436000)	16.1 (8.9-26.6)	414 (0-945)	0.0 (0.0-0.1)	10700 (56-24500)	0.7 (0.0-1.5)
Enterobacter spp.	Resistance to one or more antibiotics	553 (380-800)	0.4 (0.3-0.6)	47700 (32800-69100)	36.1 (24.8-52.4)	159 (105-231)	0.1 (0.1-0.2)	13700 (9090-19900)	10.4 (6.9-15.1)	39000 (23400-58400)	2.4 (1.4-3.6)	1010000 (599000-1550000)	61.6 (36.5-94.3)	11100 (6490-17100)	0.7 (0.4-1.0)	287000 (168000-445000)	17.5 (10.2-27.1)
Enterococcus faecalis	Fluoroquinolones	224 (136-363)	0.2 (0.1-0.3)	19500 (11900-31600)	14.8 (9.0-24.0)	55 (25-94)	0.0 (0.0-0.1)	4720 (2150-8130)	3.6 (1.6-6.2)	33700 (20300-52200)	2.1 (1.2-3.2)	984000 (592000-1520000)	60.0 (36.1-92.7)	8190 (3810-14500)	0.5 (0.2-0.9)	238000 (115000-411000)	14.5 (7.0-25.1)
Enterococcus faecalis	Vancomycin	26 (15-44)	0.0 (0.0-0.0)	2230 (1260-3830)	1.7 (1.0-2.9)	6 (2-13)	0.0 (0.0-0.0)	528 (206-1140)	0.4 (0.2-0.9)	4190 (2430-6750)	0.3 (0.1-0.4)	122000 (70700-194000)	7.4 (4.3-11.8)	995 (385-2020)	0.1 (0.0-0.1)	28900 (11400-57400)	1.8 (0.7-3.5)
Enterococcus faecalis	Resistance to one or more antibiotics	226 (137-366)	0.2 (0.1-0.3)	19700 (12000-32000)	14.9 (9.1-24.2)	61 (31-103)	0.0 (0.0-0.1)	5250 (2650-8910)	4.0 (2.0-6.8)	34000 (20500-52600)	2.1 (1.3-3.2)	994000 (598000-1530000)	60.6 (36.4-93.3)	9180 (4690-15800)	0.6 (0.3-1.0)	267000 (136000-453000)	16.2 (8.3-27.6)
Enterococcus faecium	Fluoroquinolones	176 (91-319)	0.1 (0.1-0.2)	15200 (7810-27500)	11.5 (5.9-20.8)	34 (9-75)	0.0 (0.0-0.1)	2940 (782-6490)	2.2 (0.6-4.9)	33300 (19600-53200)	2.0 (1.2-3.2)	906000 (524000-1480000)	55.2 (31.9-90.3)	6270 (1790-12700)	0.4 (0.1-0.8)	171000 (47800-349000)	10.4 (2.9-21.3)
Enterococcus faecium	Vancomycin	44 (21-83)	0.0 (0.0-0.1)	3820 (1810-7110)	2.9 (1.4-5.4)	10 (4-22)	0.0 (0.0-0.0)	885 (334-1900)	0.7 (0.3-1.4)	10300 (5660-17600)	0.6 (0.3-1.1)	277000 (148000-485000)	16.9 (9.0-29.5)	2380 (1010-4530)	0.1 (0.1-0.3)	64000 (26500-122000)	3.9 (1.6-7.4)
Enterococcus faecium	Resistance to one or more antibiotics	177 (91-320)	0.1 (0.1-0.2)	15300 (7840-27600)	11.6 (5.9-20.9)	44 (19-92)	0.0 (0.0-0.1)	3820 (1620-7910)	2.9 (1.2-6.0)	33500 (19700-53500)	2.0 (1.2-3.3)	912000 (527000-1490000)	55.6 (32.1-90.7)	8650 (4240-15200)	0.5 (0.3-0.9)	235000 (114000-419000)	14.3 (7.0-25.5)
Other enterococci	Fluoroquinolones	154 (88-255)	0.1 (0.1-0.2)	13400 (7750-22500)	10.2 (5.9-17.0)	30 (5-60)	0.0 (0.0-0.0)	2580 (429-5230)	2.0 (0.3-4.0)	18200 (11600-26700)	1.1 (0.7-1.6)	410000 (265000-608000)	25.0 (16.1-37.0)	3530 (538-7130)	0.2 (0.0-0.4)	78800 (12900-158000)	4.8 (0.8-9.6)
Other enterococci	Vancomycin	9 (5-15)	0.0 (0.0-0.0)	781 (438-1330)	0.6 (0.3-1.0)	2 (1-3)	0.0 (0.0-0.0)	144 (65-293)	0.1 (0.0-0.2)	1060 (665-1640)	0.1 (0.0-0.1)	23900 (15200-36800)	1.5 (0.9-2.2)	191 (92-372)	0.0 (0.0-0.0)	4390 (2160-8300)	0.3 (0.1-0.5)
Other enterococci	Resistance to one or more antibiotics	154 (88-255)	0.1 (0.1-0.2)	13500 (7760-22500)	10.2 (5.9-17.1)	31 (7-62)	0.0 (0.0-0.0)	2730 (649-5380)	2.1 (0.5-4.1)	18200 (11700-26800)	1.1 (0.7-1.6)	410000 (265000-608000)	25.0 (16.2-37.1)	3720 (848-7300)	0.2 (0.1-0.4)	83200 (19500-162000)	5.1 (1.2-9.8)
Escherichia coli	Aminoglycosides	1300 (911-1840)	1.0 (0.7-1.4)	115000 (80100-165000)	87.2 (60.7-125.2)	73 (47-108)	0.1 (0.0-0.1)	6420 (4170-9520)	4.9 (3.2-7.2)	52000 (36700-70600)	3.2 (2.2-4.3)	1300000 (917000-1790000)	79.1 (55.9-108.8)	2920 (1920-4180)	0.2 (0.1-0.3)	72600 (47300-104000)	4.4 (2.9-6.4)
Escherichia coli	Aminopenicillin	4370 (3140-6090)	3.3 (2.4-4.6)	385000 (276000-540000)	291.7 (209.4-409.1)	14 (9-21)	0.0 (0.0-0.0)	1250 (794-1880)	0.9 (0.6-1.4)	149000 (108000-203000)	9.1 (6.6-12.3)	3770000 (2710000-5150000)	229.6 (165.4-313.8)	506 (308-772)	0.0 (0.0-0.0)	12700 (7700-19800)	0.8 (0.5-1.2)
Escherichia coli	Beta Lactam/Beta-lactamase inhibitors	3650 (2570-5080)	2.8 (2.0-3.9)	322000 (225000-449000)	243.8 (170.9-340.7)	44 (29-65)	0.0 (0.0-0.0)	3890 (2530-5720)	2.9 (1.9-4.3)	123000 (88200-168000)	7.5 (5.4-10.2)	3110000 (2230000-4260000)	189.5 (136.0-259.4)	1540 (1000-2210)	0.1 (0.1-0.1)	38700 (25300-55500)	2.4 (1.5-3.4)
Escherichia coli	Carbapenem	1760 (1230-2560)	1.3 (0.9-1.9)	156000 (108000-227000)	118.0 (82.0-171.8)	362 (201-580)	0.3 (0.2-0.4)	31700 (17600-51000)	24.1 (13.4-38.6)	75200 (53600-103000)	4.6 (3.3-6.3)	1870000 (1330000-2600000)	113.8 (81.3-158.4)	15700 (9030-24400)	1.0 (0.6-1.5)	387000 (226000-592000)	23.6 (13.8-36.1)
Escherichia coli	Fluoroquinolones	3890 (2780-5430)	3.0 (2.1-4.1)	343000 (245000-482000)	260.0 (185.8-365.6)	412 (285-586)	0.3 (0.2-0.4)	36100 (25000-51900)	27.4 (18.9-39.4)	133000 (95000-181000)	8.1 (5.8-11.0)	3350000 (2400000-4610000)	204.0 (146.2-280.8)	14000 (9720-19900)	0.9 (0.6-1.2)	352000 (244000-501000)	21.4 (14.9-30.6)
Escherichia coli	Third-generation cephalosporins	3920 (2790-5560)	3.0 (2.1-4.2)	345000 (245000-488000)	261.4 (185.8-369.7)	388 (177-713)	0.3 (0.1-0.5)	34100 (15700-62500)	25.8 (11.9-47.4)	132000 (94000-180000)	8.1 (5.7-11.0)	3340000 (2390000-4600000)	203.3 (145.5-280.5)	11400 (5130-21000)	0.7 (0.3-1.3)	291000 (135000-523000)	17.7 (8.2-31.8)
Escherichia coli	Trimethoprim-Sulfamethoxazole	2710 (1940-3770)	2.1 (1.5-2.9)	239000 (170000-331000)	180.9 (129.2-251.1)	129 (83-187)	0.1 (0.1-0.1)	11200 (7190-16300)	8.5 (5.5-12.4)	88000 (63700-119000)	5.4 (3.9-7.2)	2220000 (1600000-3040000)	135.6 (97.5-185.3)	4150 (2710-5860)	0.3 (0.2-0.4)	104000 (67500-148000)	6.3 (4.1-9.0)
Escherichia coli	Resistance to one or more antibiotics	4640 (3330-6510)	3.5 (2.5-4.9)	409000 (292000-574000)	309.9 (221.5-434.8)	1420 (959-2060)	1.1 (0.7-1.6)	125000 (84000-182000)	94.6 (63.7-138.0)	160000 (116000-219000)	9.8 (7.0-13.4)	4040000 (2910000-5530000)	246.1 (177.2-336.8)	50300 (34100-72100)	3.1 (2.1-4.4)	1260000 (866000-1800000)	76.6 (52.8-109.4)
Group A *Streptococcus*	Macrolide	44 (25-71)	0.0 (0.0-0.1)	4160 (2460-6580)	3.2 (1.9-5.0)	4 (0-14)	0.0 (0.0-0.0)	395 (0-1240)	0.3 (0.0-0.9)	2430 (1050-4890)	0.1 (0.1-0.3)	63400 (31700-119000)	3.9 (1.9-7.3)	234 (0-888)	0.0 (0.0-0.1)	6070 (0-21900)	0.4 (0.0-1.3)
Group A *Streptococcus*	Resistance to one or more antibiotics	44 (25-71)	0.0 (0.0-0.1)	4160 (2460-6580)	3.2 (1.9-5.0)	4 (0-14)	0.0 (0.0-0.0)	395 (0-1240)	0.3 (0.0-0.9)	2430 (1050-4890)	0.1 (0.1-0.3)	63400 (31700-119000)	3.9 (1.9-7.3)	234 (0-888)	0.0 (0.0-0.1)	6070 (0-21900)	0.4 (0.0-1.3)
Group B *Streptococcus*	Fluoroquinolones	479 (341-670)	0.4 (0.3-0.5)	41700 (29800-58200)	31.6 (22.6-44.1)	81 (14-181)	0.1 (0.0-0.1)	6960 (1260-15600)	5.3 (1.0-11.9)	11300 (7450-17200)	0.7 (0.5-1.0)	298000 (204000-435000)	18.2 (12.4-26.5)	1900 (319-4320)	0.1 (0.0-0.3)	49400 (8990-107000)	3.0 (0.5-6.5)
Group B *Streptococcus*	Macrolide	823 (596-1100)	0.6 (0.5-0.8)	71500 (51900-95900)	54.2 (39.4-72.7)	76 (0-202)	0.1 (0.0-0.2)	6630 (0-17400)	5.0 (0.0-13.2)	15600 (10600-23800)	1.0 (0.6-1.4)	422000 (294000-609000)	25.7 (17.9-37.1)	1390 (0-3780)	0.1 (0.0-0.2)	37600 (0-98800)	2.3 (0.0-6.0)
Group B *Streptococcus*	Penicillin	15 (10-21)	0.0 (0.0-0.0)	1300 (891-1830)	1.0 (0.7-1.4)	4 (1-8)	0.0 (0.0-0.0)	324 (65-698)	0.2 (0.0-0.5)	272 (176-422)	0.0 (0.0-0.0)	7320 (4890-11100)	0.4 (0.3-0.7)	68 (15-147)	0.0 (0.0-0.0)	1810 (411-3780)	0.1 (0.0-0.2)
Group B *Streptococcus*	Resistance to one or more antibiotics	1020 (756-1360)	0.8 (0.6-1.0)	88800 (65800-119000)	67.3 (49.9-89.9)	161 (33-305)	0.1 (0.0-0.2)	13900 (2890-26400)	10.5 (2.2-20.0)	20400 (13900-30900)	1.2 (0.8-1.9)	547000 (388000-785000)	33.3 (23.6-47.8)	3360 (821-6600)	0.2 (0.1-0.4)	88800 (22100-169000)	5.4 (1.3-10.3)
Haemophilus influenzae	Aminopenicillin	146 (98-214)	0.1 (0.1-0.2)	12700 (8490-18500)	9.6 (6.4-14.0)	20 (2-43)	0.0 (0.0-0.0)	1750 (146-3740)	1.3 (0.1-2.8)	471 (312-690)	0.0 (0.0-0.0)	13600 (9520-19100)	0.8 (0.6-1.2)	64 (5-136)	0.0 (0.0-0.0)	1820 (230-3830)	0.1 (0.0-0.2)
Haemophilus influenzae	Third-generation cephalosporins	166 (120-226)	0.1 (0.1-0.2)	14400 (10500-19600)	10.9 (7.9-14.9)	49 (21-86)	0.0 (0.0-0.1)	4230 (1790-7480)	3.2 (1.4-5.7)	683 (480-943)	0.0 (0.0-0.1)	19200 (14100-25700)	1.2 (0.9-1.6)	202 (82-357)	0.0 (0.0-0.0)	5610 (2450-9640)	0.3 (0.1-0.6)
Haemophilus influenzae	Resistance to one or more antibiotics	272 (200-367)	0.2 (0.2-0.3)	23600 (17400-31700)	17.9 (13.2-24.0)	69 (33-115)	0.1 (0.0-0.1)	5980 (2850-9930)	4.5 (2.2-7.5)	1020 (733-1390)	0.1 (0.0-0.1)	29000 (21700-38300)	1.8 (1.3-2.3)	266 (126-428)	0.0 (0.0-0.0)	7440 (3770-11900)	0.5 (0.2-0.7)
Klebsiella pneumoniae	Aminoglycosides	3480 (2620-4540)	2.6 (2.0-3.4)	300000 (226000-392000)	227.7 (171.1-296.8)	265 (170-375)	0.2 (0.1-0.3)	22800 (14700-32400)	17.3 (11.1-24.6)	85100 (56700-124000)	5.2 (3.5-7.6)	2120000 (1420000-3110000)	129.1 (86.6-189.3)	6570 (3840-10300)	0.4 (0.2-0.6)	163000 (97100-257000)	10.0 (5.9-15.7)
Klebsiella pneumoniae	Beta Lactam/Beta-lactamase inhibitors	4480 (3370-5790)	3.4 (2.6-4.4)	386000 (291000-500000)	292.8 (220.2-378.8)	56 (32-87)	0.0 (0.0-0.1)	4820 (2740-7480)	3.7 (2.1-5.7)	108000 (72400-155000)	6.6 (4.4-9.5)	2680000 (1800000-3850000)	163.5 (109.8-234.8)	922 (463-1550)	0.1 (0.0-0.1)	23600 (11900-39500)	1.4 (0.7-2.4)
Klebsiella pneumoniae	Carbapenem	3300 (2430-4380)	2.5 (1.8-3.3)	284000 (210000-378000)	215.6 (159.5-286.3)	751 (507-1060)	0.6 (0.4-0.8)	64700 (43700-91500)	49.0 (33.1-69.4)	94000 (63000-141000)	5.7 (3.8-8.6)	2320000 (1550000-3450000)	141.3 (94.5-210.2)	22400 (13900-35000)	1.4 (0.8-2.1)	549000 (339000-862000)	33.5 (20.7-52.6)
Klebsiella pneumoniae	Fluoroquinolones	3860 (2910-4990)	2.9 (2.2-3.8)	333000 (251000-431000)	252.7 (190.6-326.4)	259 (159-384)	0.2 (0.1-0.3)	22400 (13700-33100)	17.0 (10.4-25.1)	92000 (61600-133000)	5.6 (3.8-8.1)	2300000 (1540000-3370000)	139.9 (93.7-205.1)	6230 (3620-9970)	0.4 (0.2-0.6)	157000 (91000-249000)	9.5 (5.5-15.2)
Klebsiella pneumoniae	Third-generation cephalosporins	4100 (3080-5400)	3.1 (2.3-4.1)	354000 (265000-466000)	268.0 (201.2-353.0)	179 (64-348)	0.1 (0.0-0.3)	15400 (5510-30000)	11.7 (4.2-22.8)	106000 (70700-154000)	6.5 (4.3-9.4)	2630000 (1750000-3830000)	160.6 (107.0-233.6)	3720 (1230-7860)	0.2 (0.1-0.5)	93300 (31400-196000)	5.7 (1.9-11.9)
Klebsiella pneumoniae	Trimethoprim-Sulfamethoxazole	3380 (2570-4370)	2.6 (1.9-3.3)	292000 (222000-377000)	221.2 (168.3-285.5)	142 (69-227)	0.1 (0.1-0.2)	12200 (5990-19500)	9.3 (4.5-14.8)	81000 (54500-116000)	4.9 (3.3-7.1)	2020000 (1370000-2920000)	123.1 (83.8-177.9)	3460 (1610-6030)	0.2 (0.1-0.4)	85800 (40300-150000)	5.2 (2.5-9.2)
Klebsiella pneumoniae	Resistance to one or more antibiotics	4850 (3670-6270)	3.7 (2.8-4.8)	418000 (316000-541000)	317.0 (239.8-410.1)	1650 (1230-2180)	1.3 (0.9-1.6)	142000 (106000-188000)	108.0 (80.3-142.3)	122000 (82700-176000)	7.4 (5.0-10.7)	3030000 (2060000-4370000)	184.4 (125.4-266.1)	43300 (28900-63800)	2.6 (1.8-3.9)	1070000 (719000-1590000)	65.3 (43.8-97.1)
Morganella spp.	Fluoroquinolones	8 (4-15)	0.0 (0.0-0.0)	733 (379-1270)	0.6 (0.3-1.0)	1 (1-3)	0.0 (0.0-0.0)	114 (44-231)	0.1 (0.0-0.2)	1530 (962-2260)	0.1 (0.1-0.1)	34200 (21600-50000)	2.1 (1.3-3.0)	245 (102-449)	0.0 (0.0-0.0)	5400 (2290-9660)	0.3 (0.1-0.6)
Morganella spp.	Fourth-generation cephalosporins	4 (2-7)	0.0 (0.0-0.0)	364 (186-632)	0.3 (0.1-0.5)	1 (0-1)	0.0 (0.0-0.0)	48 (12-119)	0.0 (0.0-0.1)	674 (406-1010)	0.0 (0.0-0.1)	15100 (9310-22600)	0.9 (0.6-1.4)	88 (23-189)	0.0 (0.0-0.0)	1980 (548-4180)	0.1 (0.0-0.3)
Morganella spp.	Third-generation cephalosporins	4 (2-7)	0.0 (0.0-0.0)	367 (185-634)	0.3 (0.1-0.5)	0 (0-1)	0.0 (0.0-0.0)	23 (8-53)	0.0 (0.0-0.0)	758 (454-1160)	0.0 (0.0-0.1)	16900 (10500-25100)	1.0 (0.6-1.5)	54 (20-113)	0.0 (0.0-0.0)	1190 (451-2470)	0.1 (0.0-0.2)
Morganella spp.	Resistance to one or more antibiotics	9 (4-15)	0.0 (0.0-0.0)	743 (386-1280)	0.6 (0.3-1.0)	2 (1-4)	0.0 (0.0-0.0)	186 (89-340)	0.1 (0.1-0.3)	1550 (969-2280)	0.1 (0.1-0.1)	34500 (21800-50500)	2.1 (1.3-3.1)	386 (200-650)	0.0 (0.0-0.0)	8570 (4550-13900)	0.5 (0.3-0.8)
Mycobacterium tuberculosis	Extensive drug resistance in TB	29 (9-63)	0.0 (0.0-0.0)	2520 (759-5560)	1.9 (0.6-4.2)	17 (5-39)	0.0 (0.0-0.0)	1490 (448-3300)	1.1 (0.3-2.5)	3290 (770-7830)	0.2 (0.0-0.5)	113000 (27100-268000)	6.9 (1.7-16.3)	2020 (473-4800)	0.1 (0.0-0.3)	66700 (15900-158000)	4.1 (1.0-9.7)
Mycobacterium tuberculosis	Isoniazid mono-resistance	395 (246-615)	0.3 (0.2-0.5)	37400 (24000-58000)	28.4 (18.2-44.0)	62 (0-221)	0.0 (0.0-0.2)	5510 (0-19200)	4.2 (0.0-14.5)	45900 (30000-65400)	2.8 (1.8-4.0)	1700000 (1160000-2370000)	103.3 (70.6-144.6)	7220 (0-25000)	0.4 (0.0-1.5)	247000 (0-835000)	15.0 (0.0-50.9)
Mycobacterium tuberculosis	Multi-drug resistance excluding extensive drug resistance in TB	554 (176-1190)	0.4 (0.1-0.9)	50500 (15800-109000)	38.3 (12.0-82.4)	326 (35-895)	0.2 (0.0-0.7)	27800 (2930-76500)	21.1 (2.2-58.0)	63800 (15000-151000)	3.9 (0.9-9.2)	2260000 (542000-5220000)	137.8 (33.0-318.3)	37400 (3570-103000)	2.3 (0.2-6.3)	1240000 (115000-3430000)	75.4 (7.0-209.1)
Mycobacterium tuberculosis	Rifampicin mono-resistance	47 (23-87)	0.0 (0.0-0.1)	4330 (2170-7880)	3.3 (1.6-6.0)	13 (2-31)	0.0 (0.0-0.0)	1160 (202-2750)	0.9 (0.2-2.1)	2280 (1420-3570)	0.1 (0.1-0.2)	89500 (55300-138000)	5.5 (3.4-8.4)	639 (102-1390)	0.0 (0.0-0.1)	23600 (4580-50200)	1.4 (0.3-3.1)
Mycobacterium tuberculosis	Resistance to one or more antibiotics	1020 (585-1660)	0.8 (0.4-1.3)	94800 (54300-154000)	71.8 (41.1-116.7)	419 (85-1020)	0.3 (0.1-0.8)	36000 (7380-86900)	27.3 (5.6-65.8)	115000 (62800-197000)	7.0 (3.8-12.0)	4160000 (2320000-6960000)	253.5 (141.6-424.2)	47200 (8820-116000)	2.9 (0.5-7.1)	1570000 (303000-3810000)	95.9 (18.5-232.3)
Neisseria gonorrhoeae	Fluoroquinolones	--	--	--	--	--	--	--	--	--	--	17700 (10100-28600)	1.1 (0.6-1.7)	--	--	1700 (447-3480)	0.1 (0.0-0.2)
Neisseria gonorrhoeae	Third-generation cephalosporins	--	--	--	--	--	--	--	--	--	--	195 (34-755)	0.0 (0.0-0.0)	--	--	45 (11-155)	0.0 (0.0-0.0)
Neisseria gonorrhoeae	Resistance to one or more antibiotics	--	--	--	--	--	--	--	--	--	--	17700 (10100-28700)	1.1 (0.6-1.7)	--	--	1750 (502-3560)	0.1 (0.0-0.2)
Proteus spp.	Aminoglycosides	51 (32-80)	0.0 (0.0-0.1)	4500 (2770-6920)	3.4 (2.1-5.2)	2 (0-5)	0.0 (0.0-0.0)	194 (22-415)	0.1 (0.0-0.3)	8180 (5280-12000)	0.5 (0.3-0.7)	195000 (125000-286000)	11.9 (7.6-17.5)	347 (33-740)	0.0 (0.0-0.0)	8280 (890-17700)	0.5 (0.1-1.1)
Proteus spp.	Aminopenicillin	105 (66-162)	0.1 (0.1-0.1)	9240 (5840-14100)	7.0 (4.4-10.7)	2 (0-5)	0.0 (0.0-0.0)	150 (0-445)	0.1 (0.0-0.3)	17500 (11300-26000)	1.1 (0.7-1.6)	416000 (263000-622000)	25.4 (16.0-37.9)	294 (0-889)	0.0 (0.0-0.1)	6950 (0-21200)	0.4 (0.0-1.3)
Proteus spp.	Fluoroquinolones	88 (55-136)	0.1 (0.0-0.1)	7720 (4850-11900)	5.9 (3.7-9.1)	6 (3-12)	0.0 (0.0-0.0)	548 (216-1080)	0.4 (0.2-0.8)	15200 (9890-22400)	0.9 (0.6-1.4)	360000 (227000-532000)	21.9 (13.8-32.4)	1120 (467-2100)	0.1 (0.0-0.1)	26100 (10700-49600)	1.6 (0.7-3.0)
Proteus spp.	Third-generation cephalosporins	36 (22-56)	0.0 (0.0-0.0)	3190 (1950-4910)	2.4 (1.5-3.7)	7 (2-14)	0.0 (0.0-0.0)	585 (168-1230)	0.4 (0.1-0.9)	5770 (3620-8570)	0.4 (0.2-0.5)	137000 (84900-206000)	8.4 (5.2-12.5)	1060 (320-2130)	0.1 (0.0-0.1)	25200 (7740-51000)	1.5 (0.5-3.1)
Proteus spp.	Trimethoprim-Sulfamethoxazole	68 (41-103)	0.1 (0.0-0.1)	5920 (3600-9020)	4.5 (2.7-6.8)	2 (0-4)	0.0 (0.0-0.0)	178 (2-388)	0.1 (0.0-0.3)	11200 (7170-16600)	0.7 (0.4-1.0)	267000 (170000-395000)	16.3 (10.3-24.1)	338 (0-739)	0.0 (0.0-0.0)	8010 (81-17400)	0.5 (0.0-1.1)
Proteus spp.	Resistance to one or more antibiotics	121 (75-186)	0.1 (0.1-0.1)	10600 (6590-16300)	8.0 (5.0-12.3)	19 (10-32)	0.0 (0.0-0.0)	1650 (843-2810)	1.3 (0.6-2.1)	20400 (13300-30000)	1.2 (0.8-1.8)	484000 (308000-713000)	29.5 (18.8-43.4)	3150 (1770-5080)	0.2 (0.1-0.3)	74500 (41200-122000)	4.5 (2.5-7.5)
Pseudomonas aeruginosa	Aminoglycosides	705 (510-927)	0.5 (0.4-0.7)	60900 (44100-80000)	46.2 (33.4-60.6)	18 (0-44)	0.0 (0.0-0.0)	1560 (6-3770)	1.2 (0.0-2.9)	32500 (21400-47900)	2.0 (1.3-2.9)	773000 (504000-1160000)	47.1 (30.7-70.6)	815 (0-2130)	0.0 (0.0-0.1)	19900 (367-51000)	1.2 (0.0-3.1)
Pseudomonas aeruginosa	Anti-pseudomonal penicillin/Beta-Lactamase inhibitors	742 (543-998)	0.6 (0.4-0.8)	64000 (47000-86300)	48.5 (35.6-65.4)	24 (13-39)	0.0 (0.0-0.0)	2080 (1150-3370)	1.6 (0.9-2.6)	33600 (21600-51100)	2.1 (1.3-3.1)	803000 (512000-1240000)	48.9 (31.2-75.4)	753 (342-1370)	0.0 (0.0-0.1)	18400 (8380-34100)	1.1 (0.5-2.1)
Pseudomonas aeruginosa	Carbapenem	1290 (942-1700)	1.0 (0.7-1.3)	111000 (81500-146000)	84.2 (61.8-110.9)	237 (158-348)	0.2 (0.1-0.3)	20500 (13700-30000)	15.5 (10.4-22.7)	58200 (37600-85900)	3.5 (2.3-5.2)	1390000 (893000-2090000)	84.7 (54.4-127.5)	10700 (6400-17000)	0.7 (0.4-1.0)	255000 (154000-408000)	15.5 (9.4-24.9)
Pseudomonas aeruginosa	Fluoroquinolones	894 (659-1190)	0.7 (0.5-0.9)	77200 (57000-102000)	58.5 (43.2-77.6)	91 (61-135)	0.1 (0.0-0.1)	7900 (5260-11800)	6.0 (4.0-8.9)	38800 (25300-56300)	2.4 (1.5-3.4)	928000 (599000-1380000)	56.6 (36.5-83.9)	3920 (2270-6350)	0.2 (0.1-0.4)	94400 (54900-155000)	5.8 (3.3-9.5)
Pseudomonas aeruginosa	Fourth-generation cephalosporins	1220 (895-1590)	0.9 (0.7-1.2)	105000 (77400-138000)	79.6 (58.6-104.3)	44 (26-67)	0.0 (0.0-0.1)	3780 (2230-5830)	2.9 (1.7-4.4)	51700 (33600-75800)	3.2 (2.0-4.6)	1240000 (797000-1840000)	75.6 (48.6-112.2)	1490 (828-2520)	0.1 (0.1-0.2)	36400 (20000-61400)	2.2 (1.2-3.7)
Pseudomonas aeruginosa	Third-generation cephalosporins	1420 (1040-1850)	1.1 (0.8-1.4)	122000 (89900-159000)	92.8 (68.2-120.8)	63 (31-104)	0.0 (0.0-0.1)	5450 (2670-8910)	4.1 (2.0-6.8)	52300 (33800-76600)	3.2 (2.1-4.7)	1260000 (813000-1890000)	77.0 (49.6-115.0)	1450 (613-2710)	0.1 (0.0-0.2)	36700 (15600-68500)	2.2 (0.9-4.2)
Pseudomonas aeruginosa	Resistance to one or more antibiotics	1800 (1330-2370)	1.4 (1.0-1.8)	156000 (115000-204000)	117.9 (87.2-154.6)	478 (322-702)	0.4 (0.2-0.5)	41300 (27900-60600)	31.3 (21.1-45.9)	73500 (48200-107000)	4.5 (2.9-6.5)	1770000 (1150000-2610000)	107.6 (69.9-159.2)	19100 (11400-30600)	1.2 (0.7-1.9)	461000 (272000-742000)	28.1 (16.6-45.2)
Salmonella Paratyphi	Fluoroquinolones	2220 (688-5400)	1.7 (0.5-4.1)	191000 (59300-466000)	145.1 (44.9-353.0)	445 (60-1260)	0.3 (0.0-1.0)	38300 (5200-109000)	29.0 (3.9-82.4)	17400 (7160-33500)	1.1 (0.4-2.0)	1190000 (485000-2320000)	72.4 (29.6-141.1)	3510 (533-8950)	0.2 (0.0-0.5)	238000 (36000-604000)	14.5 (2.2-36.8)
Salmonella Paratyphi	Multi-drug resistance in Salmonella Typhi and Paratyphi	91 (29-226)	0.1 (0.0-0.2)	7860 (2470-19500)	6.0 (1.9-14.8)	7 (0-22)	0.0 (0.0-0.0)	627 (0-1860)	0.5 (0.0-1.4)	656 (260-1300)	0.0 (0.0-0.1)	44800 (18000-90100)	2.7 (1.1-5.5)	53 (0-153)	0.0 (0.0-0.0)	3600 (0-10400)	0.2 (0.0-0.6)
Salmonella Paratyphi	Resistance to one or more antibiotics	2230 (691-5430)	1.7 (0.5-4.1)	192000 (59600-468000)	145.8 (45.1-354.9)	452 (66-1280)	0.3 (0.0-1.0)	38900 (5680-110000)	29.5 (4.3-83.3)	17500 (7210-33700)	1.1 (0.4-2.1)	1190000 (487000-2330000)	72.8 (29.7-141.9)	3560 (572-9040)	0.2 (0.0-0.6)	241000 (39000-608000)	14.7 (2.4-37.1)
Salmonella Typhi	Fluoroquinolones	9120 (3740-17700)	6.9 (2.8-13.4)	788000 (323000-1530000)	597.5 (245.0-1162.5)	1760 (273-4310)	1.3 (0.2-3.3)	152000 (23600-371000)	114.9 (17.9-281.4)	51000 (25700-89100)	3.1 (1.6-5.4)	3420000 (1630000-6110000)	208.7 (99.4-372.7)	9890 (1570-22600)	0.6 (0.1-1.4)	659000 (106000-1530000)	40.2 (6.5-93.2)
Salmonella Typhi	Multi-drug resistance in Salmonella Typhi and Paratyphi	2870 (1190-5690)	2.2 (0.9-4.3)	248000 (102000-491000)	187.9 (77.6-371.8)	277 (0-798)	0.2 (0.0-0.6)	23900 (0-68700)	18.1 (0.0-52.1)	15500 (7940-26500)	0.9 (0.5-1.6)	1040000 (511000-1810000)	63.6 (31.1-110.2)	1520 (0-4380)	0.1 (0.0-0.3)	102000 (0-288000)	6.2 (0.0-17.6)
Salmonella Typhi	Resistance to one or more antibiotics	10300 (4230-19900)	7.8 (3.2-15.1)	886000 (365000-1720000)	671.7 (276.5-1301.0)	2040 (495-4810)	1.5 (0.4-3.6)	175000 (42600-415000)	133.0 (32.3-314.8)	57700 (29800-99200)	3.5 (1.8-6.0)	3870000 (1850000-6740000)	235.9 (113.0-410.5)	11400 (2890-24700)	0.7 (0.2-1.5)	761000 (189000-1670000)	46.4 (11.5-101.6)
Non-typhoidal *Salmonella*	Fluoroquinolones	736 (272-1680)	0.6 (0.2-1.3)	68100 (23400-162000)	51.7 (17.7-123.1)	153 (20-458)	0.1 (0.0-0.3)	13400 (2040-39900)	10.2 (1.5-30.2)	14700 (6050-29600)	0.9 (0.4-1.8)	474000 (197000-993000)	28.9 (12.0-60.5)	3050 (416-7790)	0.2 (0.0-0.5)	90300 (14600-225000)	5.5 (0.9-13.7)
Non-typhoidal *Salmonella*	Resistance to one or more antibiotics	736 (272-1680)	0.6 (0.2-1.3)	68100 (23400-162000)	51.7 (17.7-123.1)	153 (20-458)	0.1 (0.0-0.3)	13400 (2040-39900)	10.2 (1.5-30.2)	14700 (6050-29600)	0.9 (0.4-1.8)	474000 (197000-993000)	28.9 (12.0-60.5)	3050 (416-7790)	0.2 (0.0-0.5)	90300 (14600-225000)	5.5 (0.9-13.7)
Serratia spp.	Aminoglycosides	47 (25-80)	0.0 (0.0-0.1)	4050 (2170-6880)	3.1 (1.6-5.2)	3 (0-8)	0.0 (0.0-0.0)	243 (0-726)	0.2 (0.0-0.6)	3240 (1860-5370)	0.2 (0.1-0.3)	88000 (50200-145000)	5.4 (3.1-8.9)	185 (0-532)	0.0 (0.0-0.0)	5090 (0-14100)	0.3 (0.0-0.9)
Serratia spp.	Anti-pseudomonal penicillin/Beta-Lactamase inhibitors	33 (17-58)	0.0 (0.0-0.0)	2840 (1460-5020)	2.2 (1.1-3.8)	5 (2-10)	0.0 (0.0-0.0)	449 (179-903)	0.3 (0.1-0.7)	3720 (1930-6440)	0.2 (0.1-0.4)	97300 (51200-170000)	5.9 (3.1-10.3)	572 (238-1130)	0.0 (0.0-0.1)	15000 (6160-28500)	0.9 (0.4-1.7)
Serratia spp.	Carbapenem	25 (13-43)	0.0 (0.0-0.0)	2140 (1120-3740)	1.6 (0.9-2.8)	7 (3-13)	0.0 (0.0-0.0)	561 (245-1120)	0.4 (0.2-0.9)	3090 (1680-5250)	0.2 (0.1-0.3)	80300 (43800-137000)	4.9 (2.7-8.3)	829 (384-1530)	0.1 (0.0-0.1)	21500 (9870-40100)	1.3 (0.6-2.4)
Serratia spp.	Fluoroquinolones	25 (13-43)	0.0 (0.0-0.0)	2120 (1080-3710)	1.6 (0.8-2.8)	3 (0-6)	0.0 (0.0-0.0)	216 (35-500)	0.2 (0.0-0.4)	2340 (1250-3970)	0.1 (0.1-0.2)	62100 (33400-105000)	3.8 (2.0-6.4)	239 (43-537)	0.0 (0.0-0.0)	6390 (1220-14600)	0.4 (0.1-0.9)
Serratia spp.	Fourth-generation cephalosporins	40 (21-71)	0.0 (0.0-0.1)	3470 (1810-6140)	2.6 (1.4-4.7)	3 (1-7)	0.0 (0.0-0.0)	262 (85-567)	0.2 (0.1-0.4)	4650 (2540-7850)	0.3 (0.2-0.5)	121000 (68000-207000)	7.4 (4.1-12.6)	342 (115-745)	0.0 (0.0-0.0)	8950 (3040-19800)	0.5 (0.2-1.2)
Serratia spp.	Third-generation cephalosporins	41 (21-74)	0.0 (0.0-0.1)	3500 (1810-6340)	2.7 (1.4-4.8)	1 (0-3)	0.0 (0.0-0.0)	91 (21-246)	0.1 (0.0-0.2)	3940 (2220-6720)	0.2 (0.1-0.4)	104000 (59100-175000)	6.3 (3.6-10.7)	60 (16-146)	0.0 (0.0-0.0)	1650 (463-4020)	0.1 (0.0-0.2)
Serratia spp.	Resistance to one or more antibiotics	93 (51-156)	0.1 (0.0-0.1)	7970 (4360-13400)	6.0 (3.3-10.2)	21 (10-37)	0.0 (0.0-0.0)	1820 (896-3190)	1.4 (0.7-2.4)	8910 (5110-14400)	0.5 (0.3-0.9)	235000 (135000-386000)	14.3 (8.2-23.5)	2230 (1160-3860)	0.1 (0.1-0.2)	58500 (31100-105000)	3.6 (1.9-6.4)
Shigella spp.	Fluoroquinolones	1450 (472-3200)	1.1 (0.4-2.4)	130000 (41800-286000)	98.5 (31.7-217.1)	298 (36-819)	0.2 (0.0-0.6)	26200 (3390-71600)	19.9 (2.6-54.3)	13400 (4010-33000)	0.8 (0.2-2.0)	371000 (118000-919000)	22.6 (7.2-56.0)	2730 (275-7890)	0.2 (0.0-0.5)	70300 (10000-191000)	4.3 (0.6-11.7)
Shigella spp.	Resistance to one or more antibiotics	1450 (472-3200)	1.1 (0.4-2.4)	130000 (41800-286000)	98.5 (31.7-217.1)	298 (36-819)	0.2 (0.0-0.6)	26200 (3390-71600)	19.9 (2.6-54.3)	13400 (4010-33000)	0.8 (0.2-2.0)	371000 (118000-919000)	22.6 (7.2-56.0)	2730 (275-7890)	0.2 (0.0-0.5)	70300 (10000-191000)	4.3 (0.6-11.7)
Staphylococcus aureus	Fluoroquinolones	3660 (2770-4680)	2.8 (2.1-3.5)	318000 (242000-407000)	241.2 (183.2-308.2)	145 (63-245)	0.1 (0.0-0.2)	12600 (5570-21200)	9.5 (4.2-16.0)	101000 (70500-144000)	6.2 (4.3-8.8)	2630000 (1820000-3800000)	160.3 (111.0-231.5)	4150 (1840-7160)	0.3 (0.1-0.4)	107000 (47800-188000)	6.5 (2.9-11.5)
Staphylococcus aureus	Macrolide	3230 (2470-4100)	2.4 (1.9-3.1)	281000 (214000-357000)	212.7 (162.5-270.2)	99 (41-163)	0.1 (0.0-0.1)	8650 (3650-14200)	6.6 (2.8-10.8)	83100 (58700-117000)	5.1 (3.6-7.1)	2160000 (1520000-3080000)	131.7 (92.7-187.7)	2620 (1040-4700)	0.2 (0.1-0.3)	68000 (27500-122000)	4.1 (1.7-7.4)
Staphylococcus aureus	Methicillin	2830 (2130-3650)	2.1 (1.6-2.8)	246000 (185000-316000)	186.3 (139.9-239.7)	664 (287-1130)	0.5 (0.2-0.9)	57600 (25200-98100)	43.7 (19.1-74.4)	75100 (51900-106000)	4.6 (3.2-6.5)	1950000 (1340000-2810000)	118.8 (81.9-171.5)	18100 (7630-31900)	1.1 (0.5-1.9)	466000 (199000-822000)	28.4 (12.2-50.1)

Staphylococcus aureus	Trimethoprim-Sulfamethoxazole	10600 (7460-14400)	422.4 (295.9-572.9)	949000 (664000-1290000)	37641.0 (26326.2-51069.9)	838 (415-1340)	33.2 (16.5-53.0)	74600 (36900-119000)	2957.8 (1464.9-4712.0)
Staphylococcus aureus	Vancomycin	254 (173-362)	10.1 (6.8-14.4)	22600 (15400-32200)	897.6 (609.8-1278.5)	74 (35-134)	2.9 (1.4-5.3)	6550 (3170-11900)	260.0 (125.9-473.7)
Staphylococcus aureus	Resistance to one or more antibiotics	24000 (16900-32900)	950.8 (671.6-1305.6)	2140000 (1510000-2930000)	84758.9 (59837.0-116346.8)	5840 (3450-9050)	231.7 (136.7-358.9)	520000 (307000-806000)	20631.5 (12193.8-31954.7)
Streptococcus pneumoniae	Beta Lactam/Beta-lactamase inhibitors	2960 (1840-4450)	117.6 (72.9-176.5)	266000 (165000-400000)	10541.6 (6537.0-15849.1)	30 (1-98)	1.2 (0.0-3.9)	2700 (94-8680)	107.2 (3.7-344.3)
Streptococcus pneumoniae	Carbapenem	8280 (5350-11900)	328.4 (212.0-472.1)	742000 (480000-1070000)	29430.8 (19056.3-42365.2)	1880 (853-3330)	74.5 (33.8-132.3)	169000 (76900-299000)	6684.6 (3052.0-11839.9)
Streptococcus pneumoniae	Fluoroquinolones	3120 (1980-4700)	123.8 (78.7-186.5)	280000 (178000-421000)	11105.2 (7042.1-16678.1)	408 (86-865)	16.2 (3.4-34.3)	36400 (7700-77500)	1443.9 (305.4-3074.0)
Streptococcus pneumoniae	Macrolide	13600 (9180-19200)	537.8 (364.0-761.5)	1220000 (821000-1720000)	48222.9 (32568.5-68288.2)	578 (0-1560)	22.9 (0.0-61.8)	51300 (0-139000)	2035.5 (0.0-5494.0)
Streptococcus pneumoniae	Penicillin	7930 (4980-12200)	314.4 (197.5-485.1)	710000 (446000-1100000)	28175.0 (17680.0-43508.3)	359 (127-799)	14.2 (5.0-31.7)	32100 (11500-71200)	1272.1 (456.4-2823.8)
Streptococcus pneumoniae	Third-generation cephalosporins	3990 (2530-6080)	158.1 (100.5-241.1)	357000 (227000-544000)	14178.5 (9021.4-21581.3)	100 (21-250)	4.0 (0.8-9.9)	8960 (1860-22400)	355.4 (73.7-889.4)
Streptococcus pneumoniae	Trimethoprim-Sulfamethoxazole	11700 (7910-16700)	463.4 (313.6-662.2)	1050000 (709000-1490000)	41564.7 (28133.6-59291.7)	974 (138-2080)	38.6 (5.5-82.6)	87000 (13300-186000)	3449.2 (527.9-7364.6)
Streptococcus pneumoniae	Resistance to one or more antibiotics	20400 (14100-27900)	809.4 (560.7-1106.1)	1830000 (1260000-2500000)	72599.9 (50104.8-99228.9)	4330 (2640-6360)	171.7 (104.6-252.2)	387000 (236000-568000)	15347.9 (9378.7-22531.5)

Staphylococcus aureus	Trimethoprim-Sulfamethoxazole	3900 (2850-5230)	13.0 (9.5-17.5)	345000 (252000-463000)	1151.3 (842.4-1546.0)	308 (151-497)	1.0 (0.5-1.7)	27200 (13400-43900)	90.9 (44.7-146.6)
Staphylococcus aureus	Vancomycin	93 (68-128)	0.3 (0.2-0.4)	8270 (6000-11300)	27.6 (20.0-37.8)	27 (13-47)	0.1 (0.0-0.2)	2390 (1180-4160)	8.0 (3.9-13.9)
Staphylococcus aureus	Resistance to one or more antibiotics	8830 (6640-11600)	29.5 (22.2-38.6)	782000 (588000-1020000)	2608.8 (1961.9-3419.7)	2150 (1270-3260)	7.2 (4.3-10.9)	190000 (113000-288000)	635.4 (376.4-961.8)
Streptococcus pneumoniae	Beta Lactam/Beta-lactamase inhibitors	3410 (2330-4940)	11.4 (7.8-16.5)	301000 (206000-437000)	1006.0 (688.1-1458.8)	35 (1-111)	0.1 (0.0-0.4)	3060 (102-9840)	10.2 (0.3-32.9)
Streptococcus pneumoniae	Carbapenem	9590 (6710-13100)	32.0 (22.4-43.8)	849000 (594000-1160000)	2833.3 (1982.4-3871.3)	2180 (997-3730)	7.3 (3.3-12.5)	193000 (88200-330000)	643.8 (294.6-1102.3)
Streptococcus pneumoniae	Fluoroquinolones	3570 (2470-5060)	11.9 (8.2-16.9)	316000 (218000-448000)	1055.2 (728.4-1494.4)	465 (106-918)	1.6 (0.4-3.1)	41100 (9400-81200)	137.4 (31.4-270.9)
Streptococcus pneumoniae	Macrolide	15600 (11400-21100)	52.2 (38.0-70.4)	1380000 (1010000-1870000)	4614.4 (3360.9-6225.8)	667 (0-1800)	2.2 (0.0-6.0)	59000 (0-159000)	196.9 (0.0-530.0)
Streptococcus pneumoniae	Penicillin	9170 (5840-13700)	30.6 (19.5-45.7)	811000 (516000-1210000)	2708.5 (1723.7-4042.6)	414 (151-875)	1.4 (0.5-2.9)	36600 (13400-77400)	122.2 (44.7-258.2)
Streptococcus pneumoniae	Third-generation cephalosporins	4650 (3220-6580)	15.5 (10.7-22.0)	412000 (285000-582000)	1373.6 (950.5-1942.4)	118 (27-295)	0.4 (0.1-1.0)	10500 (2350-26100)	35.0 (7.9-87.2)
Streptococcus pneumoniae	Trimethoprim-Sulfamethoxazole	13500 (9800-17900)	45.0 (32.7-59.7)	1190000 (867000-1580000)	3983.0 (2894.4-5282.3)	1120 (163-2320)	3.8 (0.5-7.8)	99500 (14400-205000)	332.0 (48.0-685.9)
Streptococcus pneumoniae	Resistance to one or more antibiotics	23500 (18000-30800)	78.6 (60.2-102.8)	2080000 (1590000-2720000)	6952.6 (5323.8-9094.7)	5000 (3340-7130)	16.7 (11.2-23.8)	443000 (296000-631000)	1477.4 (987.1-2104.9)

Staphylococcus aureus	Trimethoprim-Sulfamethoxazole	2020 (1530-2580)	1.5 (1.2-2.0)	176000 (132000-224000)	133.1 (100.3-169.6)	159 (82-246)	0.1 (0.1-0.2)	13700 (7090-21200)	10.4 (5.4-16.1)
Staphylococcus aureus	Vancomycin	46 (34-61)	0.0 (0.0-0.0)	3980 (2940-5320)	3.0 (2.2-4.0)	13 (7-23)	0.0 (0.0-0.0)	1150 (580-2000)	0.9 (0.4-1.5)
Staphylococcus aureus	Resistance to one or more antibiotics	4400 (3360-5610)	3.3 (2.5-4.3)	383000 (293000-486000)	290.5 (222.3-368.7)	1080 (649-1620)	0.8 (0.5-1.2)	93700 (56300-140000)	71.0 (42.7-106.0)
Streptococcus pneumoniae	Beta Lactam/Beta-lactamase inhibitors	1410 (961-2030)	1.1 (0.7-1.5)	122000 (83000-175000)	92.4 (62.9-132.7)	13 (0-40)	0.0 (0.0-0.0)	1140 (43-3410)	0.9 (0.0-2.6)
Streptococcus pneumoniae	Carbapenem	4420 (3170-5800)	3.3 (2.4-4.4)	382000 (274000-500000)	289.3 (207.8-379.3)	1020 (474-1720)	0.8 (0.4-1.3)	87700 (41000-148000)	66.5 (31.1-112.4)
Streptococcus pneumoniae	Fluoroquinolones	1430 (961-2020)	1.1 (0.7-1.5)	123000 (83000-175000)	93.5 (62.9-132.4)	185 (41-371)	0.1 (0.0-0.3)	16000 (3580-31900)	12.1 (2.7-24.2)
Streptococcus pneumoniae	Macrolide	6950 (5100-9230)	5.3 (3.9-7.0)	600000 (441000-797000)	455.0 (334.0-604.1)	298 (0-785)	0.2 (0.0-0.6)	25600 (0-67500)	19.4 (0.0-51.2)
Streptococcus pneumoniae	Penicillin	4120 (2730-5890)	3.1 (2.1-4.5)	355000 (236000-510000)	269.4 (178.8-386.5)	182 (70-357)	0.1 (0.1-0.3)	15700 (6050-30700)	11.9 (4.6-23.3)
Streptococcus pneumoniae	Third-generation cephalosporins	1990 (1400-2860)	1.5 (1.1-2.2)	172000 (121000-248000)	130.4 (91.5-187.9)	46 (11-116)	0.0 (0.0-0.1)	3990 (927-10000)	3.0 (0.7-7.6)
Streptococcus pneumoniae	Trimethoprim-Sulfamethoxazole	5760 (4150-7670)	4.4 (3.1-5.8)	497000 (358000-662000)	376.9 (271.7-502.0)	478 (68-988)	0.4 (0.1-0.7)	41200 (5810-85000)	31.2 (4.4-64.4)
Streptococcus pneumoniae	Resistance to one or more antibiotics	10300 (7840-13300)	7.8 (5.9-10.1)	892000 (677000-1150000)	675.9 (513.5-872.5)	2220 (1470-3160)	1.7 (1.1-2.4)	191000 (127000-272000)	145.0 (96.1-206.2)

Staphylococcus aureus	Trimethoprim-Sulfamethoxazole	49700 (34900-70700)	3.0 (2.1-4.3)	1300000 (911000-1870000)	79.2 (55.5-113.7)	3990 (1970-6590)	0.2 (0.1-0.4)	103000 (50700-171000)	6.3 (3.1-10.4)
Staphylococcus aureus	Vancomycin	1350 (913-1970)	0.1 (0.1-0.1)	34800 (23400-51400)	2.1 (1.4-3.1)	390 (185-750)	0.0 (0.0-0.0)	10100 (4870-19300)	0.6 (0.3-1.2)
Staphylococcus aureus	Resistance to one or more antibiotics	122000 (85500-172000)	7.4 (5.2-10.5)	3160000 (2210000-4510000)	192.7 (134.9-274.6)	29200 (16700-46100)	1.8 (1.0-2.8)	754000 (431000-1190000)	46.0 (26.3-72.4)
Streptococcus pneumoniae	Beta Lactam/Beta-lactamase inhibitors	11400 (7370-16800)	0.7 (0.4-1.0)	317000 (212000-462000)	19.3 (12.9-28.1)	123 (4-387)	0.0 (0.0-0.0)	3320 (140-10500)	0.2 (0.0-0.6)
Streptococcus pneumoniae	Carbapenem	28100 (19700-38900)	1.7 (1.2-2.4)	811000 (582000-1110000)	49.4 (35.5-67.8)	6300 (2900-11000)	0.4 (0.2-0.7)	183000 (88100-308000)	11.1 (5.4-18.7)
Streptococcus pneumoniae	Fluoroquinolones	12400 (8260-17800)	0.8 (0.5-1.1)	343000 (235000-486000)	20.9 (14.3-29.6)	1620 (361-3400)	0.1 (0.0-0.2)	44200 (10300-92500)	2.7 (0.6-5.6)
Streptococcus pneumoniae	Macrolide	47900 (34600-66000)	2.9 (2.1-4.0)	1370000 (1010000-1850000)	83.4 (61.4-113.0)	2030 (0-5430)	0.1 (0.0-0.3)	56300 (0-150000)	3.4 (0.0-9.1)
Streptococcus pneumoniae	Penicillin	27900 (17300-42600)	1.7 (1.1-2.6)	796000 (505000-1190000)	48.5 (30.8-72.4)	1290 (426-2880)	0.1 (0.0-0.2)	36200 (12700-79900)	2.2 (0.8-4.9)
Streptococcus pneumoniae	Third-generation cephalosporins	15100 (10200-22000)	0.9 (0.6-1.3)	427000 (295000-605000)	26.1 (18.0-36.9)	433 (99-1080)	0.0 (0.0-0.1)	12000 (2770-29200)	0.7 (0.2-1.8)
Streptococcus pneumoniae	Trimethoprim-Sulfamethoxazole	43500 (30400-60900)	2.7 (1.9-3.7)	1230000 (881000-1700000)	74.8 (53.7-103.4)	3650 (534-7400)	0.2 (0.0-0.5)	101000 (15800-207000)	6.2 (1.0-12.6)
Streptococcus pneumoniae	Resistance to one or more antibiotics	73600 (55300-97700)	4.5 (3.4-6.0)	2090000 (1620000-2720000)	127.5 (98.6-165.9)	15400 (10000-21900)	0.9 (0.6-1.3)	436000 (291000-609000)	26.6 (17.7-37.1)

Table S19. Deaths and DALYs (in counts and age-specific rates) associated with and attributable to bacterial antimicrobial resistance by pathogen–drug combination; by age group; 2019; southeast Asia, east Asia, and Oceania

Pathogen	Antibiotic Class	Neonatal								Post Neonatal								1 to 4								5 and over							
		Associated with resistance				Attributable to resistance				Associated with resistance				Attributable to resistance				Associated with resistance				Attributable to resistance				Associated with resistance				Attributable to resistance			
		Deaths		DALYs		Deaths		DALYs		Deaths		DALYs		Deaths		DALYs		Deaths		DALYs		Deaths		DALYs		Deaths		DALYs		Deaths		DALYs	
		Counts	Rate per 100 000	Counts	Rate per 100 000	Counts	Rate per 100 000	Counts	Rate per 100 000	Counts	Rate per 100 000	Counts	Rate per 100 000	Counts	Rate per 100 000	Counts	Rate per 100 000	Counts	Rate per 100 000	Counts	Rate per 100 000	Counts	Rate per 100 000	Counts	Rate per 100 000	Counts	Rate per 100 000	Counts	Rate per 100 000	Counts	Rate per 100 000	Counts	Rate per 100 000
All pathogens	Resistance to one or more antibiotics	31500 (22600-42600)	1546.8 (1112.0-2093.3)	2810000 (2010000-3800000)	137875.2 (98985.1-186490.4)	7800 (5570-10700)	383.1 (273.8-527.4)	695000 (496000-956000)	34122.9 (24383.7-46961.3)	24900 (20100-30700)	100.8 (81.5-124.4)	2210000 (1790000-2730000)	8960.0 (7246.7-11047.5)	5810 (4510-7380)	23.5 (18.3-29.9)	515000 (400000-654000)	2084.9 (1618.1-2648.4)	9880 (7540-13000)	8.7 (6.6-11.4)	878000 (667000-1130000)	771.6 (586.1-997.1)	2320 (1700-3090)	2.0 (1.5-2.7)	202000 (148000-268000)	177.7 (130.5-235.5)	950000 (622000-1380000)	47.0 (30.8-68.5)	22000000 (14400000-32200000)	1090.7 (711.7-1595.1)	238000 (153000-348000)	11.8 (7.6-17.2)	5460000 (3510000-8090000)	270.5 (173.9-400.8)
Acinetobacter baumannii	Aminoglycosides	1170 (762-1730)	57.5 (37.5-84.9)	104000 (68100-154000)	5131.8 (3343.6-7576.5)	54 (0-127)	2.7 (0.0-6.3)	4850 (36-11300)	238.1 (1.8-557.6)	439 (265-672)	1.8 (1.1-2.7)	38800 (23400-59500)	157.2 (94.7-240.7)	21 (0-50)	0.1 (0.0-0.2)	1820 (2-4420)	7.4 (0.0-17.9)	165 (87-283)	0.1 (0.1-0.2)	14200 (7510-24400)	12.5 (6.6-21.4)	8 (0-20)	0.0 (0.0-0.0)	666 (2-1740)	0.6 (0.0-1.5)	55500 (30000-92600)	2.8 (1.5-4.6)	1240000 (668000-2080000)	61.5 (33.1-102.9)	2600 (0-6810)	0.1 (0.0-0.3)	58400 (320-152000)	2.9 (0.0-7.5)
Acinetobacter baumannii	Anti-pseudomonal penicillin/Beta-Lactamase inhibitors	2120 (1410-3050)	104.1 (69.2-149.7)	189000 (125000-272000)	9283.6 (6160.4-13353.0)	130 (69-219)	6.4 (3.4-10.8)	11500 (6110-19500)	567.3 (300.1-959.4)	858 (519-1280)	3.5 (2.1-5.2)	75900 (45900-114000)	307.1 (185.9-460.2)	40 (20-68)	0.2 (0.1-0.3)	3500 (1740-5990)	14.2 (7.1-24.2)	322 (174-533)	0.3 (0.2-0.5)	27700 (15000-45900)	24.4 (13.2-40.3)	15 (7-26)	0.0 (0.0-0.0)	1260 (575-2260)	1.1 (0.5-2.0)	108000 (60400-175000)	5.3 (3.0-8.6)	2410000 (1340000-3870000)	119.3 (66.2-191.9)	2940 (1300-5530)	0.1 (0.1-0.3)	66700 (29300-126000)	3.3 (1.4-6.3)
Acinetobacter baumannii	Beta Lactam/Beta-lactamase inhibitors	1720 (1130-2490)	84.5 (55.5-122.3)	153000 (101000-222000)	7535.2 (4945.0-10916.4)	6 (1-12)	0.3 (0.1-0.6)	496 (109-1100)	24.4 (5.4-54.2)	709 (438-1060)	2.9 (1.8-4.3)	62700 (38700-93400)	254.0 (156.8-378.2)	2 (0-5)	0.0 (0.0-0.0)	179 (37-408)	0.7 (0.2-1.7)	267 (145-440)	0.2 (0.1-0.4)	23000 (12500-37900)	20.2 (11.0-33.4)	1 (0-2)	0.0 (0.0-0.0)	68 (14-159)	0.1 (0.0-0.1)	88800 (49800-145000)	4.4 (2.5-7.2)	1980000 (1100000-3270000)	97.9 (54.4-161.8)	114 (22-335)	0.0 (0.0-0.0)	2660 (503-7720)	0.1 (0.0-0.4)
Acinetobacter baumannii	Carbapenem	1720 (1120-2500)	84.7 (55.1-122.6)	154000 (100000-223000)	7563.9 (4919.1-10956.3)	317 (173-521)	15.6 (8.5-25.6)	28300 (15400-46500)	1391.5 (758.5-2282.7)	817 (490-1240)	3.3 (2.0-5.0)	72300 (43300-110000)	292.7 (175.5-445.4)	159 (85-264)	0.6 (0.3-1.1)	14000 (7520-23400)	56.8 (30.4-94.7)	308 (164-518)	0.3 (0.1-0.5)	26500 (14200-44600)	23.3 (12.5-39.2)	60 (28-109)	0.1 (0.0-0.1)	5130 (2420-9400)	4.5 (2.1-8.3)	118000 (66600-187000)	5.9 (3.3-9.3)	2630000 (1480000-4180000)	130.2 (73.2-206.9)	23500 (11700-41100)	1.2 (0.6-2.0)	521000 (260000-913000)	25.8 (12.9-45.2)
Acinetobacter baumannii	Fluoroquinolones	1590 (1060-2340)	78.1 (52.0-115.0)	142000 (94400-209000)	6972.5 (4637.7-10273.6)	203 (108-343)	10.0 (5.3-16.8)	18100 (9650-30500)	888.6 (474.3-1499.3)	674 (405-1010)	2.7 (1.6-4.1)	59700 (35800-89600)	241.5 (144.9-362.6)	86 (43-143)	0.4 (0.2-0.6)	7650 (3820-12600)	31.0 (15.5-51.1)	255 (134-421)	0.2 (0.1-0.4)	21900 (11600-36300)	19.3 (10.2-31.9)	33 (14-56)	0.0 (0.0-0.0)	2810 (1240-4810)	2.5 (1.1-4.2)	91100 (50200-145000)	4.5 (2.5-7.2)	2030000 (1110000-3270000)	100.4 (55.1-162.0)	11600 (5150-20300)	0.6 (0.3-1.0)	259000 (116000-452000)	12.8 (5.7-22.4)
Acinetobacter baumannii	Fourth-generation cephalosporins	2000 (1320-2870)	98.1 (64.8-141.0)	178000 (118000-256000)	8764.1 (5782.1-12587.3)	19 (8-39)	0.9 (0.4-1.9)	1730 (723-3480)	85.1 (35.5-170.8)	918 (562-1400)	3.7 (2.3-5.6)	81200 (49700-123000)	328.8 (201.3-499.9)	13 (5-26)	0.1 (0.0-0.1)	1160 (449-2290)	4.7 (1.8-9.3)	343 (184-567)	0.3 (0.2-0.5)	29600 (15900-48800)	26.0 (14.0-42.9)	5 (2-10)	0.0 (0.0-0.0)	424 (153-901)	0.4 (0.1-0.8)	126000 (72000-199000)	6.2 (3.6-9.9)	2800000 (1560000-4400000)	138.9 (77.4-218.0)	2180 (753-4550)	0.1 (0.0-0.2)	48200 (16900-100000)	2.4 (0.8-5.0)
Acinetobacter baumannii	Third-generation cephalosporins	2000 (1320-2900)	98.0 (64.7-142.6)	178000 (118000-259000)	8753.8 (5776.2-12738.7)	35 (13-71)	1.7 (0.7-3.5)	3150 (1200-6360)	155.0 (58.9-312.3)	896 (547-1380)	3.6 (2.2-5.6)	79300 (48400-122000)	320.9 (195.9-493.5)	19 (7-37)	0.1 (0.0-0.1)	1660 (594-3260)	6.7 (2.4-13.2)	338 (183-563)	0.3 (0.2-0.5)	29100 (15800-48500)	25.6 (13.9-42.6)	7 (2-15)	0.0 (0.0-0.0)	617 (208-1260)	0.5 (0.2-1.1)	123000 (69100-198000)	6.1 (3.4-9.8)	2720000 (1540000-4380000)	135.0 (76.1-217.0)	2540 (863-5230)	0.1 (0.0-0.3)	56600 (19500-118000)	2.8 (1.0-5.9)
Acinetobacter baumannii	Resistance to one or more antibiotics	2480 (1640-3610)	122.0 (80.7-177.3)	222000 (146000-322000)	10892.8 (7193.3-15823.6)	764 (474-1160)	37.5 (23.3-57.1)	68200 (42300-104000)	3350.0 (2076.8-5094.1)	1090 (674-1650)	4.4 (2.7-6.7)	96800 (59600-146000)	391.8 (241.5-591.8)	339 (202-528)	1.4 (0.8-2.1)	30000 (17800-46700)	121.3 (72.2-188.9)	411 (223-683)	0.4 (0.2-0.6)	35400 (19200-58800)	31.2 (16.9-51.7)	127 (67-215)	0.1 (0.1-0.2)	11000 (5780-18500)	9.6 (5.1-16.3)	145000 (82900-227000)	7.2 (4.1-11.3)	3240000 (1820000-5150000)	160.3 (90.3-254.9)	45500 (24600-75800)	2.3 (1.2-3.8)	1010000 (552000-1700000)	50.1 (27.4-84.2)
Citrobacter spp.	Aminoglycosides	40 (24-64)	2.0 (1.2-3.2)	3600 (2160-5730)	176.8 (106.3-281.5)	2 (0-5)	0.1 (0.0-0.3)	196 (0-485)	9.6 (0.0-23.8)	25 (13-43)	0.1 (0.1-0.2)	2200 (1180-3830)	8.9 (4.8-15.5)	1 (0-4)	0.0 (0.0-0.0)	120 (0-326)	0.5 (0.0-1.3)	9 (4-16)	0.0 (0.0-0.0)	752 (343-1410)	0.7 (0.3-1.2)	0 (0-1)	0.0 (0.0-0.0)	41 (0-114)	0.0 (0.0-0.1)	618 (352-1000)	0.0 (0.0-0.0)	15400 (8580-25200)	0.8 (0.4-1.2)	33 (0-83)	0.0 (0.0-0.0)	821 (0-2110)	0.0 (0.0-0.1)
Citrobacter spp.	Anti-pseudomonal penicillin/Beta-Lactamase inhibitors	118 (70-194)	5.8 (3.4-9.5)	10500 (6240-17300)	516.8 (306.7-849.4)	21 (10-38)	1.0 (0.5-1.9)	1860 (890-3390)	91.4 (43.7-166.8)	94 (49-165)	0.4 (0.2-0.7)	8330 (4350-14600)	33.7 (17.6-59.0)	17 (7-33)	0.1 (0.0-0.1)	1510 (651-2910)	6.1 (2.6-11.8)	35 (16-64)	0.0 (0.0-0.1)	3000 (1360-5500)	2.6 (1.2-4.8)	6 (3-13)	0.0 (0.0-0.0)	543 (219-1080)	0.5 (0.2-1.0)	2970 (1670-4910)	0.1 (0.1-0.2)	72100 (40200-120000)	3.6 (2.0-6.0)	535 (258-985)	0.0 (0.0-0.0)	13000 (6200-23800)	0.6 (0.3-1.2)
Citrobacter spp.	Carbapenem	70 (42-115)	3.5 (2.0-5.7)	6280 (3720-10300)	308.6 (182.7-504.7)	17 (8-30)	0.8 (0.4-1.5)	1480 (725-2670)	72.7 (35.6-131.1)	65 (34-113)	0.3 (0.1-0.5)	5750 (3040-10000)	23.3 (12.3-40.6)	16 (7-31)	0.1 (0.0-0.1)	1420 (649-2720)	5.7 (2.6-11.0)	24 (11-44)	0.0 (0.0-0.0)	2060 (926-3770)	1.8 (0.8-3.3)	6 (2-12)	0.0 (0.0-0.0)	507 (201-1030)	0.4 (0.2-0.9)	2250 (1260-3740)	0.1 (0.1-0.2)	54400 (30300-91000)	2.7 (1.5-4.5)	563 (263-1050)	0.0 (0.0-0.1)	13600 (6360-25300)	0.7 (0.3-1.3)
Citrobacter spp.	Fluoroquinolones	142 (84-232)	7.0 (4.1-11.4)	12600 (7470-20600)	620.2 (366.8-1014.5)	21 (9-39)	1.0 (0.4-1.9)	1830 (796-3440)	90.0 (39.1-169.1)	102 (55-178)	0.4 (0.2-0.7)	9040 (4880-15800)	36.6 (19.8-63.8)	15 (6-31)	0.1 (0.0-0.1)	1340 (562-2740)	5.4 (2.3-11.1)	38 (18-71)	0.0 (0.0-0.1)	3240 (1530-6070)	2.8 (1.3-5.3)	6 (2-11)	0.0 (0.0-0.0)	478 (174-975)	0.4 (0.2-0.9)	3070 (1710-4960)	0.2 (0.1-0.2)	74800 (41500-121000)	3.7 (2.1-6.0)	456 (195-843)	0.0 (0.0-0.0)	11100 (4730-20500)	0.5 (0.2-1.0)
Citrobacter spp.	Fourth-generation cephalosporins	191 (112-318)	9.4 (5.5-15.6)	17000 (10000-28300)	834.1 (492.4-1391.7)	20 (8-38)	1.0 (0.4-1.9)	1780 (755-3400)	87.2 (37.1-166.9)	114 (60-199)	0.5 (0.2-0.8)	10100 (5320-17600)	41.0 (21.5-71.2)	11 (4-22)	0.0 (0.0-0.1)	964 (366-1930)	3.9 (1.5-7.8)	42 (19-79)	0.0 (0.0-0.1)	3640 (1680-6790)	3.2 (1.5-6.0)	4 (1-8)	0.0 (0.0-0.0)	346 (125-713)	0.3 (0.1-0.6)	2950 (1680-4750)	0.1 (0.1-0.2)	72600 (41200-118000)	3.6 (2.0-5.9)	242 (102-462)	0.0 (0.0-0.0)	6010 (2510-11500)	0.3 (0.1-0.6)
Citrobacter spp.	Third-generation cephalosporins	273 (160-446)	13.4 (7.8-21.9)	24300 (14300-39800)	1195.5 (700.4-1953.1)	23 (9-46)	1.1 (0.4-2.2)	2080 (813-4060)	102.1 (39.9-199.6)	222 (119-376)	0.9 (0.5-1.5)	19700 (10600-33200)	79.6 (42.7-134.5)	22 (8-44)	0.1 (0.0-0.2)	1950 (751-3860)	7.9 (3.0-15.6)	81 (38-149)	0.1 (0.0-0.1)	7000 (3230-12800)	6.2 (2.8-11.3)	8 (3-17)	0.0 (0.0-0.0)	684 (232-1430)	0.6 (0.2-1.3)	6980 (4050-11400)	0.3 (0.2-0.6)	169000 (95700-276000)	8.4 (4.7-13.7)	729 (290-1400)	0.0 (0.0-0.1)	17600 (6880-33600)	0.9 (0.3-1.7)
Citrobacter spp.	Resistance to one or more antibiotics	358 (213-575)	17.6 (10.4-28.3)	31900 (19000-51200)	1568.5 (932.8-2517.1)	103 (56-176)	5.1 (2.7-8.7)	9220 (4950-15700)	453.0 (243.4-770.4)	282 (152-481)	1.1 (0.6-1.9)	25000 (13500-42600)	101.0 (54.6-172.3)	82 (41-152)	0.3 (0.2-0.6)	7300 (3590-13500)	29.5 (14.5-54.5)	103 (48-185)	0.1 (0.0-0.2)	8870 (4100-15900)	7.8 (3.6-14.0)	30 (13-57)	0.0 (0.0-0.1)	2600 (1140-4910)	2.3 (1.0-4.3)	8650 (4970-14100)	0.4 (0.2-0.7)	210000 (119000-341000)	10.4 (5.9-16.9)	2560 (1340-4460)	0.1 (0.1-0.2)	62100 (32100-107000)	3.1 (1.6-5.3)
Enterobacter spp.	Aminoglycosides	530 (352-772)	26.0 (17.3-37.9)	47200 (31300-68700)	2317.2 (1540.2-3375.3)	32 (8-63)	1.6 (0.4-3.1)	2850 (754-5630)	139.8 (37.0-276.7)	116 (78-164)	0.5 (0.3-0.7)	10300 (6920-14500)	41.5 (28.0-58.8)	7 (2-14)	0.0 (0.0-0.1)	627 (178-1210)	2.5 (0.7-4.9)	46 (29-71)	0.0 (0.0-0.1)	4000 (2540-6120)	3.5 (2.2-5.4)	3 (1-6)	0.0 (0.0-0.1)	242 (67-494)	0.2 (0.1-0.4)	7090 (4290-11100)	0.4 (0.2-0.6)	175000 (104000-276000)	8.6 (5.1-13.6)	448 (120-917)	0.0 (0.0-0.0)	11100 (2980-22500)	0.5 (0.1-1.1)
Enterobacter spp.	Anti-pseudomonal penicillin/Beta-Lactamase inhibitors	1220 (813-1770)	59.7 (39.9-87.1)	108000 (72400-158000)	5315.6 (3555.7-7754.4)	125 (68-205)	6.1 (3.4-10.1)	11100 (6080-18200)	546.1 (298.5-896.5)	289 (198-411)	1.2 (0.8-1.7)	25600 (17600-36400)	103.5 (71.2-147.4)	31 (17-50)	0.1 (0.1-0.2)	2720 (1500-4430)	11.0 (6.1-17.9)	114 (71-175)	0.1 (0.1-0.2)	9820 (6090-15100)	8.6 (5.4-13.2)	12 (6-20)	0.0 (0.0-0.0)	1030 (528-1740)	0.9 (0.5-1.5)	23500 (14000-37000)	1.2 (0.7-1.8)	570000 (331000-906000)	28.2 (16.4-44.9)	2600 (1280-4560)	0.1 (0.1-0.2)	63000 (31100-110000)	3.1 (1.5-5.5)
Enterobacter spp.	Carbapenem	766 (509-1120)	37.6 (25.0-55.2)	68100 (45300-100000)	3347.8 (2226.0-4921.6)	190 (116-300)	9.3 (5.7-14.8)	16900 (10300-26700)	831.6 (506.6-1313.5)	179 (122-259)	0.7 (0.5-1.0)	15800 (10800-22900)	64.1 (43.6-92.9)	45 (27-72)	0.2 (0.1-0.3)	4010 (2390-6350)	16.2 (9.7-25.7)	72 (45-111)	0.1 (0.0-0.1)	6170 (3860-9580)	5.4 (3.4-8.4)	18 (10-30)	0.0 (0.0-0.0)	1550 (853-2580)	1.4 (0.8-2.3)	14800 (8620-23400)	0.7 (0.4-1.2)	359000 (206000-582000)	17.8 (10.2-28.8)	3920 (2100-6630)	0.2 (0.1-0.3)	95000 (50400-164000)	4.7 (2.5-8.1)
Enterobacter spp.	Fluoroquinolones	1130 (732-1700)	55.7 (36.0-83.5)	101000 (65100-151000)	4952.9 (3199.6-7431.1)	130 (66-229)	6.4 (3.2-11.2)	11500 (5860-20300)	566.0 (288.1-998.3)	248 (167-358)	1.0 (0.7-1.5)	21900 (14800-31700)	88.8 (60.0-128.4)	29 (15-49)	0.1 (0.1-0.2)	2530 (1320-4370)	10.2 (5.3-17.7)	102 (64-161)	0.1 (0.1-0.1)	8830 (5500-13800)	7.8 (4.8-12.2)	12 (6-22)	0.0 (0.0-0.0)	1020 (501-1850)	0.9 (0.4-1.6)	16400 (9600-26300)	0.8 (0.5-1.3)	401000 (231000-651000)	19.9 (11.4-32.3)	1930 (931-3520)	0.1 (0.0-0.2)	47000 (22400-86600)	2.3 (1.1-4.3)
Enterobacter spp.	Fourth-generation cephalosporins	1310 (876-1940)	64.1 (43.0-95.2)	116000 (78000-172000)	5707.1 (3830.2-8474.9)	64 (31-115)	3.2 (1.5-5.7)	5710 (2720-10300)	280.7 (133.5-504.5)	276 (190-390)	1.1 (0.8-1.6)	24400 (16800-34600)	98.9 (68.0-140.0)	13 (6-24)	0.1 (0.0-0.1)	1150 (553-2100)	4.7 (2.2-8.5)	113 (73-171)	0.1 (0.1-0.2)	9790 (6310-14700)	8.6 (5.6-12.9)	5 (2-10)	0.0 (0.0-0.0)	468 (215-873)	0.4 (0.2-0.8)	16900 (10000-26400)	0.8 (0.5-1.3)	416000 (242000-649000)	20.6 (12.0-32.2)	619 (271-1170)	0.0 (0.0-0.1)	15400 (6760-29300)	0.8 (0.3-1.5)
Enterobacter spp.	Trimethoprim-Sulfamethoxazole	1460 (955-2160)	71.8 (46.9-106.0)	130000 (85000-192000)	6394.1 (4178.1-9431.4)	85 (0-183)	4.2 (0.0-9.0)	7600 (0-16300)	373.5 (0.0-799.0)	321 (222-452)	1.3 (0.9-1.8)	28400 (19600-40000)	115.0 (79.4-161.9)	19 (0-42)	0.1 (0.0-0.2)	1680 (0-3680)	6.8 (0.0-14.9)	131 (84-202)	0.1 (0.1-0.2)	11300 (7220-17400)	10.0 (6.3-15.3)	8 (0-17)	0.0 (0.0-0.0)	667 (0-1430)	0.6 (0.0-1.3)	22000 (12800-34500)	1.1 (0.6-1.7)	540000 (309000-856000)	26.8 (15.3-42.4)	1360 (0-3090)	0.1 (0.0-0.2)	33200 (0-76100)	1.6 (0.0-3.8)
Enterobacter spp.	Resistance to one or more antibiotics	2640 (1790-3830)	129.6 (87.8-188.0)	235000 (159000-341000)	11535.1 (7817.8-16733.6)	626 (410-921)	30.8 (20.1-45.3)	55700 (36500-82000)	2737.7 (1791.3-4028.5)	605 (425-847)	2.5 (1.7-3.4)	53600 (37700-75000)	217.1 (152.4-303.8)	144 (96-207)	0.6 (0.4-0.8)	12700 (8520-18300)	51.5 (34.5-74.1)	243 (154-367)	0.2 (0.1-0.3)	21000 (13300-31600)	18.4 (11.7-27.8)	58 (35-89)	0.1 (0.0-0.1)	4980 (3050-7710)	4.4 (2.7-6.8)	45700 (26900-70300)	2.3 (1.3-3.5)	1110000 (651000-1730000)	55.2 (32.2-85.8)	10900 (6370-17000)	0.5 (0.3-0.8)	265000 (153000-423000)	13.1 (7.6-21.0)
Enterococcus faecalis	Fluoroquinolones	574 (393-851)	28.2 (19.3-41.8)	51200 (35000-76000)	2516.7 (1719.8-3732.1)	143 (67-247)	7.0 (3.3-12.1)	12700 (5990-21900)	626.0 (294.4-1077.9)	128 (74-210)	0.5 (0.3-0.9)	11400 (6540-18700)	46.0 (26.5-75.7)	32 (14-59)	0.1 (0.1-0.2)	2820 (1230-5210)	11.4 (5.0-21.1)	64 (35-113)	0.1 (0.0-0.1)	5540 (3020-9770)	4.9 (2.7-8.6)	16 (7-31)	0.0 (0.0-0.0)	1370 (567-2700)	1.2 (0.5-2.4)	19400 (11000-31300)	1.0 (0.5-1.5)	496000 (284000-801000)	24.6 (14.1-39.7)	4840 (2160-9160)	0.2 (0.1-0.5)	123000 (54900-231000)	6.1 (2.7-11.5)
Enterococcus faecalis	Vancomycin	51 (34-77)	2.5 (1.7-3.8)	4570 (3000-6830)	224.6 (147.2-335.3)	15 (7-28)	0.7 (0.3-1.4)	1340 (592-2490)	66.1 (29.1-122.2)	12 (7-20)	0.0 (0.0-0.1)	1040 (606-1760)	4.2 (2.5-7.1)	4 (2-7)	0.0 (0.0-0.0)	315 (135-645)	1.3 (0.5-2.6)	6 (3-10)	0.0 (0.0-0.0)	484 (262-902)	0.4 (0.2-0.8)	2 (1-4)	0.0 (0.0-0.0)	145 (59-314)	0.1 (0.1-0.3)	1780 (972-3030)	0.1 (0.0-0.1)	45300 (25500-75200)	2.2 (1.3-3.7)	539 (224-1040)	0.0 (0.0-0.1)	13700 (5730-26700)	0.7 (0.3-1.3)
Enterococcus faecalis	Resistance to one or more antibiotics	591 (404-868)	29.1 (19.8-42.7)	52800 (35900-77500)	2591.7 (1765.6-3809.4)	158 (84-265)	7.8 (4.1-13.0)	14100 (7450-23500)	692.1 (366.0-1156.9)	132 (77-217)	0.5 (0.3-0.9)	11700 (6820-19300)	47.6 (27.6-78.2)	35 (17-64)	0.1 (0.1-0.3)	3130 (1490-5650)	12.7 (6.0-22.9)	66 (36-116)	0.1 (0.0-0.1)	5710 (3110-10000)	5.0 (2.7-8.8)	18 (8-34)	0.0 (0.0-0.0)	1520 (679-2900)	1.3 (0.6-2.6)	20100 (11400-32300)	1.0 (0.6-1.6)	513000 (296000-830000)	25.4 (14.7-41.1)	5380 (2520-9730)	0.3 (0.1-0.5)	137000 (66200-246000)	6.8 (3.3-12.2)
Enterococcus faecium	Fluoroquinolones	527 (358-770)	25.9 (17.6-37.8)	47100 (32000-68700)	2315.0 (1570.3-3374.6)	107 (33-198)	5.3 (1.6-9.7)	9550 (2940-17600)	469.3 (144.5-866.1)	292 (166-480)	1.2 (0.7-1.9)	26000 (14800-42600)	105.1 (59.8-172.6)	60 (17-128)	0.2 (0.1-0.5)	5340 (1530-11400)	21.6 (6.2-46.1)	134 (68-237)	0.1 (0.1-0.2)	11500 (5890-20400)	10.1 (5.2-18.0)	27 (8-62)	0.0 (0.0-0.1)	2360 (653-5340)	2.1 (0.6-4.7)	49500 (29500-78200)	2.5 (1.5-3.9)	1200000 (700000-1920000)	59.6 (34.7-95.0)	10200 (2940-19900)	0.5 (0.1-1.0)	248000 (71100-486000)	12.3 (3.5-24.1)
Enterococcus faecium	Vancomycin	70 (45-105)	3.4 (2.2-5.2)	6220 (4000-9360)	305.4 (196.7-460.0)	17 (8-31)	0.8 (0.4-1.5)	1530 (740-2790)	75.2 (36.4-137.0)	29 (16-48)	0.1 (0.1-0.2)	2560 (1440-4240)	10.4 (5.8-17.2)	7 (3-14)	0.0 (0.0-0.1)	638 (281-1280)	2.6 (1.1-5.2)	15 (7-26)	0.0 (0.0-0.0)	1250 (615-2250)	1.1 (0.5-2.0)	4 (1-8)	0.0 (0.0-0.0)	311 (124-651)	0.3 (0.1-0.6)	4430 (2600-6870)	0.2 (0.1-0.3)	108000 (62500-173000)	5.4 (3.1-8.6)	1120 (506-2030)	0.1 (0.0-0.1)	27300 (12400-50600)	1.4 (0.6-2.5)
Enterococcus faecium	Resistance to one or more antibiotics	529 (359-772)	26.0 (17.6-38.0)	47300 (32000-69000)	2322.7 (1574.6-3389.4)	124 (53-216)	6.1 (2.6-10.6)	11100 (4760-19300)	544.5 (234.0-946.7)	293 (167-480)	1.2 (0.7-1.9)	26000 (14800-42700)	105.4 (59.9-173.0)	67 (26-137)	0.3 (0.1-0.6)	5980 (2290-12100)	24.2 (9.3-49.0)	134 (69-238)	0.1 (0.1-0.2)	11500 (5910-20500)	10.1 (5.2-18.0)	31 (11-66)	0.0 (0.0-0.1)	2670 (975-5680)	2.4 (0.9-5.0)	49600 (29600-78500)	2.5 (1.5-3.9)	1210000 (701000-1920000)	59.7 (34.7-95.3)	11300 (4410-21200)	0.6 (0.2-1.1)	275000 (106000-516000)	13.6 (5.3-25.6)
Other enterococci	Fluoroquinolones	621 (389-968)	30.5 (19.1-47.6)	55300 (34600-86200)	2715.4 (1700.9-4235.1)	117 (18-235)	5.7 (0.9-11.5)	10400 (1570-20800)	509.1 (76.9-1024.3)	143 (79-240)	0.6 (0.3-1.0)	12700 (6990-21200)	51.4 (28.3-86.0)	27 (4-57)	0.1 (0.0-0.2)	2370 (349-5020)	9.6 (1.4-20.3)	52 (26-94)	0.0 (0.0-0.1)	4560 (2260-8140)	4.0 (2.0-7.2)	10 (1-21)	0.0 (0.0-0.0)	844 (130-1830)	0.7 (0.1-1.6)	13000 (7810-20300)	0.6 (0.4-1.0)	279000 (169000-438000)	13.8 (8.4-21.7)	2380 (349-4850)	0.1 (0.0-0.2)	51100 (7730-102000)	2.5 (0.4-5.1)
Other enterococci	Vancomycin	103 (64-161)	5.0 (3.1-7.9)	9160 (5670-14400)	450.1 (278.8-705.7)	19 (9-36)	0.9 (0.5-1.7)	1650 (840-3170)	81.3 (41.3-155.8)	30 (16-53)	0.1 (0.1-0.2)	2660 (1410-4690)	10.8 (5.7-19.0)	6 (3-11)	0.0 (0.0-0.0)	502 (231-982)	2.0 (0.9-4.0)	11 (5-21)	0.0 (0.0-0.0)	970 (463-1790)	0.9 (0.4-1.6)	2 (1-4)	0.0 (0.0-0.0)	185 (82-390)	0.2 (0.1-0.3)	3590 (2110-5660)	0.2 (0.1-0.3)	75900 (44100-122000)	3.8 (2.2-6.0)	689 (343-1250)	0.0 (0.0-0.1)	14600 (7250-26600)	0.7 (0.4-1.3)
Other enterococci	Resistance to one or more antibiotics	629 (394-984)	30.9 (19.4-48.3)	56000 (35100-87600)	2751.2 (1723.3-4303.2)	135 (43-258)	6.6 (2.1-12.7)	12000 (3820-22900)	590.4 (187.6-1126.4)	148 (82-246)	0.6 (0.3-1.0)	13100 (7260-21800)	53.0 (29.4-88.1)	32 (11-64)	0.1 (0.0-0.3)	2870 (960-5650)	11.6 (3.9-22.9)	54 (27-98)	0.0 (0.0-0.1)	4720 (2370-8480)	4.1 (2.1-7.5)	12 (4-25)	0.0 (0.0-0.0)	1030 (332-2130)	0.9 (0.3-1.9)	13700 (8220-21000)	0.7 (0.4-1.0)	294000 (177000-460000)	14.6 (8.8-22.8)	3070 (1210-5680)	0.2 (0.1-0.3)	65700 (25800-121000)	3.3 (1.3-6.0)
Escherichia coli	Aminoglycosides	1060 (717-1520)	52.2 (35.2-74.6)	94800 (63900-135000)	4658.5 (3140.1-6655.8)	60 (36-90)	2.9 (1.8-4.4)	5330 (3250-7980)	261.9 (159.8-391.9)	528 (381-717)	2.1 (1.5-2.9)	47100 (33900-64100)	190.8 (137.1-259.6)	30 (20-43)	0.1 (0.1-0.2)	2650 (1750-3870)	10.7 (7.1-15.7)	218 (153-300)	0.2 (0.1-0.3)	19400 (13500-26900)	17.1 (11.9-23.7)	12 (8-18)	0.0 (0.0-0.0)	1100 (705-1610)	1.0 (0.6-1.4)	21000 (13900-30300)	1.0 (0.7-1.5)	485000 (317000-708000)	24.0 (15.7-35.1)	1240 (763-1850)	0.1 (0.0-0.1)	28500 (17100-42700)	1.4 (0.8-2.1)
Escherichia coli	Aminopenicillin	4620 (3160-6520)	227.2 (155.5-320.5)	413000 (283000-582000)	20268.5 (13885.0-28590.3)	52 (34-75)	2.5 (1.6-3.7)	4620 (2990-6680)	227.2 (147.0-328.0)	2460 (1860-3250)	10.0 (7.5-13.2)	219000 (165000-291000)	888.2 (668.9-1177.5)	29 (21-40)	0.1 (0.1-0.2)	2610 (1840-3590)	10.5 (7.5-14.5)	1060 (776-1420)	0.9 (0.7-1.2)	94200 (68500-127000)	82.9 (60.2-111.4)	12 (8-17)	0.0 (0.0-0.0)	1080 (746-1520)	0.9 (0.7-1.3)	136000 (89300-198000)	6.7 (4.4-9.8)	3060000 (1970000-4510000)	151.5 (97.8-223.2)	2120 (1250-3330)	0.1 (0.1-0.2)	46700 (27200-73200)	2.3 (1.3-3.6)
Escherichia coli	Beta Lactam/Beta-lactamase inhibitors	3490 (2390-4960)	171.5 (117.3-243.6)	311000 (213000-442000)	15296.4 (10452.1-21729.8)	101 (67-150)	5.0 (3.3-7.4)	9010 (5930-13300)	442.9 (291.3-655.5)	1840 (1360-2440)	7.4 (5.5-9.9)	164000 (121000-218000)	663.8 (490.5-882.7)	53 (38-75)	0.2 (0.2-0.3)	4760 (3390-6650)	19.3 (13.7-26.9)	810 (594-1090)	0.7 (0.5-1.0)	72100 (52300-97600)	63.4 (46.0-85.8)	25 (17-34)	0.0 (0.0-0.0)	2190 (1540-3040)	1.9 (1.4-2.7)	93900 (62000-137000)	4.7 (3.1-6.8)	2130000 (1380000-3140000)	105.4 (68.5-155.7)	3080 (1900-4600)	0.2 (0.1-0.2)	69100 (42200-105000)	3.4 (2.1-5.2)
Escherichia coli	Carbapenem	593 (380-872)	29.2 (18.7-42.9)	52900 (33900-77700)	2598.1 (1664.5-3818.3)	117 (59-193)	5.8 (2.9-9.5)	10400 (5290-17200)	512.6 (259.7-844.1)	277 (188-387)	1.1 (0.8-1.6)	24700 (16800-34600)	99.8 (67.8-140.0)	55 (30-88)	0.2 (0.1-0.4)	4890 (2710-7850)	19.8 (11.0-31.8)	114 (76-164)	0.1 (0.1-0.1)	10100 (6690-14700)	8.9 (5.9-12.9)	23 (13-38)	0.0 (0.0-0.0)	2020 (1120-3290)	1.8 (1.0-2.9)	10900 (7000-16200)	0.5 (0.3-0.8)	253000 (159000-377000)	12.5 (7.9-18.7)	2300 (1250-3810)	0.1 (0.1-0.2)	53000 (28500-88900)	2.6 (1.4-4.4)
Escherichia coli	Fluoroquinolones	3540 (2410-5000)	173.8 (118.4-245.7)	316000 (215000-446000)	15509.7 (10570.5-21936.9)	372 (247-540)	18.3 (12.1-26.5)	33100 (22000-48200)	1628.1 (1081.9-2365.8)	1880 (1390-2470)	7.6 (5.6-10.0)	167000 (124000-222000)	677.6 (501.6-897.5)	198 (143-271)	0.8 (0.6-1.1)	17600 (12700-24100)	71.4 (51.6-97.7)	797 (580-1070)	0.7 (0.5-0.9)	70900 (51400-96600)	62.3 (45.1-84.9)	84 (60-116)	0.1 (0.1-0.1)	7440 (5220-10300)	6.5 (4.6-9.0)	99300 (65300-144000)	4.9 (3.2-7.1)	2240000 (1450000-3300000)	111.0 (71.8-163.4)	10700 (6790-15900)	0.5 (0.3-0.8)	240000 (150000-360000)	11.9 (7.4-17.8)
Escherichia coli	Third-generation cephalosporins	3420 (2310-4860)	167.9 (113.5-238.6)	305000 (206000-433000)	14976.8 (10121.1-21288.3)	448 (194-837)	22.0 (9.5-41.1)	40000 (17300-74500)	1963.5 (852.3-3659.2)	1820 (1320-2450)	7.4 (5.3-9.9)	162000 (118000-219000)	657.3 (477.1-886.7)	244 (110-430)	1.0 (0.4-1.7)	21700 (9800-38100)	88.0 (39.7-154.3)	777 (567-1060)	0.7 (0.5-0.9)	69100 (50400-94900)	60.8 (44.3-83.4)	105 (47-190)	0.1 (0.0-0.2)	9340 (4250-16700)	8.2 (3.7-14.7)	97300 (65300-143000)	4.8 (3.2-7.1)	2200000 (1440000-3300000)	108.8 (71.5-163.3)	13900 (5970-25700)	0.7 (0.3-1.3)	312000 (134000-584000)	15.5 (6.6-28.9)
Escherichia coli	Trimethoprim-Sulfamethoxazole	3260 (2250-4610)	160.3 (110.4-226.3)	291000 (200000-411000)	14297.4 (9849.1-20176.7)	170 (108-257)	8.4 (5.3-12.6)	15200 (9620-23000)	745.1 (472.7-1127.7)	1710 (1280-2270)	6.9 (5.2-9.2)	153000 (114000-204000)	619.2 (461.4-825.6)	90 (60-126)	0.4 (0.2-0.5)	7970 (5330-11200)	32.3 (21.6-45.3)	734 (539-984)	0.6 (0.5-0.9)	65200 (47500-87800)	57.3 (41.7-77.2)	39 (27-54)	0.0 (0.0-0.0)	3390 (2290-4770)	3.0 (2.0-4.2)	89800 (59000-130000)	4.4 (2.9-6.4)	2030000 (1310000-2980000)	100.5 (64.8-147.8)	4820 (2930-7230)	0.2 (0.1-0.4)	108000 (65000-164000)	5.4 (3.2-8.1)
Escherichia coli	Resistance to one or more antibiotics	4880 (3350-6900)	240.0 (164.5-338.9)	436000 (299000-616000)	21412.5 (14672.6-30286.0)	1320 (862-1960)	64.8 (42.4-96.1)	118000 (76900-174000)	5781.3 (3779.0-8571.1)	2600 (1960-3440)	10.5 (7.9-13.9)	232000 (175000-308000)	939.8 (708.3-1248.0)	699 (480-985)	2.8 (1.9-4.0)	62200 (42800-87500)	252.0 (173.1-354.2)	1120 (823-1510)	1.0 (0.7-1.3)	99900 (72800-134000)	87.9 (64.0-118.1)	301 (204-434)	0.3 (0.2-0.4)	26600 (18000-38400)	23.3 (15.8-33.7)	145000 (95100-211000)	7.2 (4.7-10.5)	3260000 (2100000-4790000)	161.3 (103.9-237.5)	38100 (24400-58600)	1.9 (1.2-2.9)	858000 (543000-1330000)	42.5 (26.9-66.0)
Group A *Streptococcus*	Macrolide	203 (128-320)	10.0 (6.3-15.7)	18200 (11500-28700)	891.8 (564.1-1408.0)	19 (0-60)	0.9 (0.0-3.0)	1660 (0-5350)	81.7 (0.0-262.8)	375 (230-589)	1.5 (0.9-2.4)	33700 (20700-52500)	136.4 (83.8-212.5)	34 (0-111)	0.1 (0.0-0.4)	3040 (0-9870)	12.3 (0.0-40.0)	143 (77-245)	0.1 (0.1-0.2)	13600 (7610-23000)	12.0 (6.7-20.2)	13 (0-42)	0.0 (0.0-0.0)	1220 (0-3840)	1.1 (0.0-3.4)	23900 (9440-50400)	1.2 (0.5-2.5)	564000 (251000-1100000)	28.0 (12.4-54.5)	2190 (0-8740)	0.1 (0.0-0.4)	51500 (0-197000)	2.6 (0.0-9.7)
Group A *Streptococcus*	Resistance to one or more antibiotics	203 (128-320)	10.0 (6.3-15.7)	18200 (11500-28700)	891.8 (564.1-1408.0)	19 (0-60)	0.9 (0.0-3.0)	1660 (0-5350)	81.7 (0.0-262.8)	375 (230-589)	1.5 (0.9-2.4)	33700 (20700-52500)	136.4 (83.8-212.5)	34 (0-111)	0.1 (0.0-0.4)	3040 (0-9870)	12.3 (0.0-40.0)	143 (77-245)	0.1 (0.1-0.2)	13600 (7610-23000)	12.0 (6.7-20.2)	13 (0-42)	0.0 (0.0-0.0)	1220 (0-3840)	1.1 (0.0-3.4)	23900 (9440-50400)	1.2 (0.5-2.5)	564000 (251000-1100000)	28.0 (12.4-54.5)	2190 (0-8740)	0.1 (0.0-0.4)	51500 (0-197000)	2.6 (0.0-9.7)
Group B *Streptococcus*	Fluoroquinolones	575 (366-842)	28.2 (18.0-41.4)	51200 (32500-74900)	2513.3 (1598.3-3681.9)	97 (14-236)	4.8 (0.7-11.6)	8620 (1280-21000)	423.7 (63.1-1030.6)	146 (106-199)	0.6 (0.4-0.8)	12900 (9370-17600)	52.4 (37.9-71.2)	24 (4-55)	0.1 (0.0-0.2)	2140 (356-4880)	8.7 (1.4-19.8)	60 (42-84)	0.1 (0.0-0.1)	5220 (3620-7270)	4.6 (3.2-6.4)	10 (2-23)	0.0 (0.0-0.0)	866 (148-1960)	0.8 (0.1-1.7)	3500 (2170-5460)	0.2 (0.1-0.3)	84700 (51900-134000)	4.2 (2.6-6.7)	550 (88-1350)	0.0 (0.0-0.1)	13300 (2190-32800)	0.7 (0.1-1.6)
Group B *Streptococcus*	Macrolide	2380 (1610-3440)	116.9 (79.3-169.2)	212000 (144000-307000)	10427.8 (7077.4-15088.3)	238 (0-648)	11.7 (0.0-31.9)	21200 (0-57700)	1041.4 (0.0-2835.6)	742 (567-973)	3.0 (2.3-3.9)	65700 (50300-86100)	265.9 (203.4-348.6)	74 (0-193)	0.3 (0.0-0.8)	6590 (0-17100)	26.7 (0.0-69.2)	286 (204-394)	0.3 (0.2-0.3)	24900 (17700-34200)	21.9 (15.5-30.1)	29 (0-76)	0.0 (0.0-0.1)	2490 (0-6580)	2.2 (0.0-5.8)	30900 (19100-48200)	1.5 (0.9-2.4)	729000 (442000-1150000)	36.1 (21.9-56.9)	3100 (0-8960)	0.2 (0.0-0.4)	73100 (0-213000)	3.6 (0.0-10.5)
Group B *Streptococcus*	Penicillin	184 (113-283)	9.0 (5.6-13.9)	16400 (10100-25200)	803.7 (494.3-1238.2)	35 (6-76)	1.7 (0.3-3.7)	3090 (554-6720)	151.6 (27.2-330.4)	37 (26-52)	0.2 (0.1-0.2)	3280 (2300-4560)	13.3 (9.3-18.5)	7 (1-15)	0.0 (0.0-0.1)	629 (126-1330)	2.5 (0.5-5.4)	15 (10-21)	0.0 (0.0-0.0)	1320 (883-1860)	1.2 (0.8-1.6)	3 (1-6)	0.0 (0.0-0.0)	251 (53-534)	0.2 (0.0-0.5)	791 (496-1230)	0.0 (0.0-0.1)	19300 (12000-30000)	1.0 (0.6-1.5)	160 (32-354)	0.0 (0.0-0.0)	3870 (797-8250)	0.2 (0.0-0.4)
Group B *Streptococcus*	Resistance to one or more antibiotics	2680 (1810-3820)	131.7 (89.0-187.9)	239000 (161000-341000)	11738.0 (7934.1-16759.7)	369 (29-816)	18.2 (1.4-40.1)	32900 (2700-72500)	1616.7 (132.8-3564.5)	807 (620-1050)	3.3 (2.5-4.2)	71500 (54900-92600)	289.3 (222.1-374.9)	106 (0-226)	0.4 (0.0-0.9)	9360 (0-19900)	37.9 (0.0-80.8)	314 (225-430)	0.3 (0.2-0.4)	27300 (19600-37200)	24.0 (17.2-32.7)	42 (0-90)	0.0 (0.0-0.1)	3610 (35-7900)	3.2 (0.0-6.9)	31800 (19800-49400)	1.6 (1.0-2.4)	752000 (459000-1180000)	37.3 (22.7-58.7)	3810 (0-9730)	0.2 (0.0-0.5)	90200 (0-229000)	4.5 (0.0-11.3)
Haemophilus influenzae	Aminopenicillin	547 (354-816)	26.9 (17.4-40.1)	48800 (31600-72700)	2398.6 (1551.8-3570.9)	86 (7-175)	4.2 (0.4-8.6)	7620 (677-15500)	374.5 (33.3-763.4)	776 (627-962)	3.1 (2.5-3.9)	68700 (55500-85100)	278.1 (224.8-344.7)	123 (11-240)	0.5 (0.0-1.0)	10900 (1020-21200)	44.1 (4.1-85.9)	284 (219-367)	0.2 (0.2-0.3)	24900 (19200-32000)	21.9 (16.9-28.1)	44 (4-89)	0.0 (0.0-0.1)	3870 (350-7690)	3.4 (0.3-6.8)	6010 (4340-8310)	0.3 (0.2-0.4)	125000 (90600-173000)	6.2 (4.5-8.6)	998 (87-1970)	0.0 (0.0-0.1)	20500 (2070-40000)	1.0 (0.1-2.0)
Haemophilus influenzae	Third-generation cephalosporins	121 (75-179)	6.0 (3.7-8.8)	10800 (6670-16000)	530.6 (327.7-785.8)	34 (15-62)	1.7 (0.7-3.0)	3070 (1300-5520)	150.9 (64.0-271.2)	155 (116-202)	0.6 (0.5-0.8)	13700 (10300-17900)	55.6 (41.7-72.4)	44 (22-74)	0.2 (0.1-0.3)	3900 (1920-6530)	15.8 (7.8-26.4)	62 (45-82)	0.1 (0.0-0.1)	5400 (3930-7190)	4.7 (3.5-6.3)	18 (8-30)	0.0 (0.0-0.0)	1530 (720-2620)	1.3 (0.6-2.3)	749 (543-1030)	0.0 (0.0-0.1)	17200 (12500-23700)	0.9 (0.6-1.2)	216 (103-372)	0.0 (0.0-0.0)	4910 (2410-8370)	0.2 (0.1-0.4)
Haemophilus influenzae	Resistance to one or more antibiotics	581 (372-863)	28.5 (18.3-42.4)	51800 (33200-77000)	2544.6 (1630.6-3784.5)	120 (37-227)	5.9 (1.8-11.1)	10700 (3310-20200)	525.3 (162.6-991.3)	816 (663-1000)	3.3 (2.7-4.1)	72200 (58700-88900)	292.5 (237.7-360.1)	167 (55-294)	0.7 (0.2-1.2)	14800 (4870-26000)	59.9 (19.7-105.3)	300 (233-387)	0.3 (0.2-0.3)	26200 (20400-33800)	23.1 (17.9-29.7)	62 (21-111)	0.1 (0.0-0.1)	5400 (1870-9540)	4.7 (1.6-8.4)	6160 (4460-8520)	0.3 (0.2-0.4)	129000 (93400-178000)	6.4 (4.6-8.8)	1210 (324-2250)	0.1 (0.0-0.1)	25400 (7700-46400)	1.3 (0.4-2.3)
Klebsiella pneumoniae	Aminoglycosides	1200 (823-1670)	59.2 (40.5-82.0)	107000 (73300-149000)	5267.1 (3599.6-7299.8)	109 (65-161)	5.3 (3.2-7.9)	9680 (5810-14300)	475.6 (285.6-703.3)	645 (473-847)	2.6 (1.9-3.4)	57100 (41900-75000)	231.0 (169.5-303.5)	59 (37-83)	0.2 (0.2-0.3)	5190 (3290-7300)	21.0 (13.3-29.6)	251 (179-348)	0.2 (0.2-0.3)	21700 (15400-30000)	19.0 (13.6-26.4)	23 (14-34)	0.0 (0.0-0.0)	1970 (1210-2920)	1.7 (1.1-2.6)	13600 (8930-20500)	0.7 (0.4-1.0)	310000 (202000-469000)	15.3 (10.0-23.3)	1360 (776-2150)	0.1 (0.0-0.1)	30700 (17500-48900)	1.5 (0.9-2.4)
Klebsiella pneumoniae	Beta Lactam/Beta-lactamase inhibitors	3930 (2770-5290)	193.2 (136.2-259.8)	350000 (247000-471000)	17195.5 (12121.7-23124.4)	28 (11-53)	1.4 (0.6-2.6)	2490 (1020-4730)	122.5 (50.2-232.3)	2100 (1640-2670)	8.5 (6.7-10.8)	186000 (145000-236000)	752.7 (589.0-956.3)	16 (7-31)	0.1 (0.0-0.1)	1450 (583-2750)	5.9 (2.4-11.2)	819 (598-1110)	0.7 (0.5-1.0)	70700 (51600-95700)	62.2 (45.4-84.1)	6 (2-12)	0.0 (0.0-0.0)	514 (190-1030)	0.5 (0.2-0.9)	58700 (38900-85800)	2.9 (1.9-4.2)	1320000 (859000-1950000)	65.2 (42.5-96.7)	574 (197-1430)	0.0 (0.0-0.1)	12700 (4430-30500)	0.6 (0.2-1.5)
Klebsiella pneumoniae	Carbapenem	1840 (1270-2600)	90.4 (62.2-127.8)	164000 (113000-231000)	8046.5 (5533.3-11373.4)	456 (292-692)	22.4 (14.3-34.0)	40500 (26000-61500)	1991.4 (1275.7-3022.0)	980 (700-1400)	4.0 (2.8-5.7)	86700 (61900-124000)	351.1 (250.8-501.9)	245 (162-385)	1.0 (0.7-1.6)	21600 (14300-34000)	87.6 (57.9-137.8)	367 (246-543)	0.3 (0.2-0.5)	31700 (21200-46800)	27.8 (18.7-41.2)	93 (58-145)	0.1 (0.1-0.1)	7980 (5020-12500)	7.0 (4.4-11.0)	27600 (14400-54100)	1.4 (0.7-2.7)	618000 (324000-1180000)	30.6 (16.0-58.7)	7240 (3680-14800)	0.4 (0.2-0.7)	161000 (81600-324000)	8.0 (4.0-16.1)
Klebsiella pneumoniae	Fluoroquinolones	3620 (2530-4990)	178.0 (124.5-245.0)	322000 (226000-444000)	15841.4 (11082.4-21817.2)	282 (168-436)	13.9 (8.3-21.4)	25200 (15000-38800)	1236.6 (737.9-1908.0)	1950 (1550-2500)	7.9 (6.3-10.1)	173000 (137000-221000)	698.5 (554.3-895.0)	153 (96-217)	0.6 (0.4-0.9)	13500 (8490-19200)	54.7 (34.4-77.9)	743 (546-1030)	0.7 (0.5-0.9)	64200 (47100-88500)	56.4 (41.4-77.8)	58 (35-88)	0.1 (0.0-0.1)	5030 (3080-7610)	4.4 (2.7-6.7)	51100 (33800-76200)	2.5 (1.7-3.8)	1150000 (750000-1730000)	56.8 (37.2-85.8)	4030 (2330-6470)	0.2 (0.1-0.3)	91000 (52900-146000)	4.5 (2.6-7.2)
Klebsiella pneumoniae	Third-generation cephalosporins	4900 (3450-6660)	240.6 (169.5-327.1)	436000 (307000-592000)	21409.6 (15084.9-29107.3)	593 (230-1090)	29.1 (11.3-53.6)	52700 (20500-97100)	2591.3 (1008.4-4772.1)	2670 (2030-3550)	10.8 (8.2-14.4)	237000 (179000-315000)	957.9 (726.0-1273.5)	330 (124-600)	1.3 (0.5-2.4)	29200 (10900-53000)	118.2 (44.3-214.8)	1050 (753-1420)	0.9 (0.7-1.3)	90700 (65000-123000)	79.7 (57.1-107.9)	133 (50-247)	0.1 (0.0-0.2)	11500 (4350-21300)	10.1 (3.8-18.7)	76800 (45100-121000)	3.8 (2.2-6.0)	1710000 (1020000-2740000)	84.9 (50.3-135.8)	9930 (3140-21200)	0.5 (0.2-1.0)	221000 (71200-469000)	10.9 (3.5-23.2)
Klebsiella pneumoniae	Trimethoprim-Sulfamethoxazole	3790 (2650-5150)	186.4 (130.2-252.9)	338000 (236000-458000)	16592.6 (11584.7-22516.9)	203 (99-341)	10.0 (4.9-16.7)	18000 (8810-30300)	885.8 (432.8-1489.3)	2140 (1690-2720)	8.7 (6.8-11.0)	189000 (150000-241000)	765.5 (605.7-974.5)	117 (58-186)	0.5 (0.2-0.8)	10400 (5120-16500)	42.0 (20.7-66.6)	814 (599-1110)	0.7 (0.5-1.0)	70200 (51700-95500)	61.7 (45.4-84.0)	44 (22-74)	0.0 (0.0-0.1)	3830 (1860-6340)	3.4 (1.6-5.6)	67400 (43500-99600)	3.3 (2.2-4.9)	1490000 (952000-2210000)	73.9 (47.1-109.7)	4010 (1840-7250)	0.2 (0.1-0.4)	88000 (40700-160000)	4.4 (2.0-7.9)
Klebsiella pneumoniae	Resistance to one or more antibiotics	5530 (3950-7450)	271.8 (194.0-366.2)	492000 (351000-663000)	24192.9 (17267.6-32584.3)	1670 (1070-2430)	82.1 (52.7-119.2)	149000 (95400-216000)	7303.3 (4688.3-10601.0)	3090 (2440-3910)	12.5 (9.9-15.9)	273000 (216000-347000)	1106.0 (873.0-1403.3)	920 (630-1300)	3.7 (2.6-5.2)	81400 (55800-115000)	329.4 (225.9-464.1)	1200 (886-1600)	1.1 (0.8-1.4)	103000 (76400-138000)	90.9 (67.2-121.1)	357 (235-521)	0.3 (0.2-0.5)	30800 (20300-45000)	27.1 (17.9-39.5)	93900 (61100-138000)	4.6 (3.0-6.8)	2080000 (1330000-3120000)	103.3 (65.9-154.5)	27100 (15900-42700)	1.3 (0.8-2.1)	604000 (352000-965000)	29.9 (17.5-47.8)
Morganella spp.	Fluoroquinolones	0 (0-1)	0.0 (0.0-0.0)	22 (5-60)	1.1 (0.3-2.9)	0 (0-0)	0.0 (0.0-0.0)	4 (1-11)	0.2 (0.0-0.5)	1 (0-1)	0.0 (0.0-0.0)	46 (19-95)	0.2 (0.1-0.4)	0 (0-0)	0.0 (0.0-0.0)	8 (2-19)	0.0 (0.0-0.1)	0 (0-1)	0.0 (0.0-0.0)	39 (17-81)	0.0 (0.0-0.1)	0 (0-0)	0.0 (0.0-0.0)	7 (2-16)	0.0 (0.0-0.0)	306 (150-549)	0.0 (0.0-0.0)	6010 (3020-10800)	0.3 (0.1-0.5)	53 (17-114)	0.0 (0.0-0.0)	1040 (333-2240)	0.1 (0.0-0.1)
Morganella spp.	Fourth-generation cephalosporins	0 (0-0)	0.0 (0.0-0.0)	11 (3-29)	0.5 (0.1-1.4)	0 (0-0)	0.0 (0.0-0.0)	2 (0-6)	0.1 (0.0-0.3)	0 (0-1)	0.0 (0.0-0.0)	22 (9-46)	0.1 (0.0-0.2)	0 (0-0)	0.0 (0.0-0.0)	4 (1-11)	0.0 (0.0-0.0)	0 (0-0)	0.0 (0.0-0.0)	16 (7-32)	0.0 (0.0-0.0)	0 (0-0)	0.0 (0.0-0.0)	3 (1-8)	0.0 (0.0-0.0)	91 (45-161)	0.0 (0.0-0.0)	1820 (926-3210)	0.1 (0.0-0.2)	18 (7-39)	0.0 (0.0-0.0)	361 (140-772)	0.0 (0.0-0.0)
Morganella spp.	Third-generation cephalosporins	0 (0-0)	0.0 (0.0-0.0)	15 (4-41)	0.7 (0.2-2.0)	0 (0-0)	0.0 (0.0-0.0)	2 (0-7)	0.1 (0.0-0.3)	0 (0-1)	0.0 (0.0-0.0)	30 (12-63)	0.1 (0.0-0.3)	0 (0-0)	0.0 (0.0-0.0)	4 (1-11)	0.0 (0.0-0.0)	0 (0-1)	0.0 (0.0-0.0)	25 (11-52)	0.0 (0.0-0.0)	0 (0-0)	0.0 (0.0-0.0)	4 (1-9)	0.0 (0.0-0.0)	193 (96-345)	0.0 (0.0-0.0)	3790 (1950-6690)	0.2 (0.1-0.3)	30 (10-66)	0.0 (0.0-0.0)	590 (206-1300)	0.0 (0.0-0.1)
Morganella spp.	Resistance to one or more antibiotics	0 (0-1)	0.0 (0.0-0.0)	32 (8-90)	1.6 (0.4-4.4)	0 (0-0)	0.0 (0.0-0.0)	8 (2-22)	0.4 (0.1-1.1)	1 (0-2)	0.0 (0.0-0.0)	66 (28-137)	0.3 (0.1-0.6)	0 (0-0)	0.0 (0.0-0.0)	16 (6-36)	0.1 (0.0-0.1)	1 (0-1)	0.0 (0.0-0.0)	55 (24-111)	0.0 (0.0-0.1)	0 (0-0)	0.0 (0.0-0.0)	13 (5-29)	0.0 (0.0-0.0)	412 (208-725)	0.0 (0.0-0.0)	8120 (4200-14200)	0.4 (0.2-0.7)	101 (41-202)	0.0 (0.0-0.0)	1990 (823-3980)	0.1 (0.0-0.2)
Mycobacterium tuberculosis	Extensive drug resistance in TB	--	--	0 (0-0)	0.0 (0.0-0.0)	--	--	0 (0-0)	0.0 (0.0-0.0)	18 (7-40)	0.1 (0.0-0.2)	1630 (610-3520)	6.6 (2.5-14.3)	11 (4-24)	0.0 (0.0-0.1)	997 (376-2160)	4.0 (1.5-8.8)	11 (4-23)	0.0 (0.0-0.0)	1010 (395-2090)	0.9 (0.3-1.8)	7 (3-14)	0.0 (0.0-0.0)	564 (214-1160)	0.5 (0.2-1.0)	1730 (694-3670)	0.1 (0.0-0.2)	52000 (21100-111000)	2.6 (1.0-5.5)	1060 (432-2240)	0.1 (0.0-0.1)	29500 (11500-62800)	1.5 (0.6-3.1)
Mycobacterium tuberculosis	Isoniazid mono-resistance	--	--	0 (0-1)	0.0 (0.0-0.0)	--	--	0 (0-0)	0.0 (0.0-0.0)	88 (57-130)	0.4 (0.2-0.5)	7900 (5120-11600)	32.0 (20.7-47.1)	14 (0-48)	0.1 (0.0-0.2)	1240 (0-4200)	5.0 (0.0-17.0)	55 (35-83)	0.0 (0.0-0.1)	6100 (4130-8810)	5.4 (3.6-7.7)	9 (0-30)	0.0 (0.0-0.0)	801 (0-2590)	0.7 (0.0-2.3)	9580 (6560-13700)	0.5 (0.3-0.7)	329000 (238000-458000)	16.3 (11.8-22.7)	1500 (0-5180)	0.1 (0.0-0.3)	45200 (0-152000)	2.2 (0.0-7.5)
Mycobacterium tuberculosis	Multi-drug resistance excluding extensive drug resistance in TB	--	--	0 (0-0)	0.0 (0.0-0.0)	--	--	0 (0-0)	0.0 (0.0-0.0)	96 (39-201)	0.4 (0.2-0.8)	8550 (3480-17800)	34.6 (14.1-72.1)	57 (6-147)	0.2 (0.0-0.6)	5040 (537-13000)	20.4 (2.2-52.5)	57 (22-116)	0.1 (0.0-0.1)	5630 (2220-11400)	4.9 (2.0-10.1)	34 (4-87)	0.0 (0.0-0.1)	2850 (292-7380)	2.5 (0.3-6.5)	9040 (3870-18400)	0.4 (0.2-0.9)	287000 (121000-584000)	14.2 (6.0-28.9)	5350 (558-14100)	0.3 (0.0-0.7)	149000 (14600-388000)	7.4 (0.7-19.2)
Mycobacterium tuberculosis	Rifampicin mono-resistance	--	--	0 (0-0)	0.0 (0.0-0.0)	--	--	0 (0-0)	0.0 (0.0-0.0)	35 (24-49)	0.1 (0.1-0.2)	3120 (2110-4420)	12.6 (8.5-17.9)	10 (2-20)	0.0 (0.0-0.1)	870 (151-1810)	3.5 (0.6-7.3)	25 (16-37)	0.0 (0.0-0.0)	2710 (1780-3820)	2.4 (1.6-3.4)	7 (1-15)	0.0 (0.0-0.0)	658 (151-1340)	0.6 (0.1-1.2)	3520 (2560-4760)	0.2 (0.1-0.2)	123000 (91400-165000)	6.1 (4.5-8.2)	987 (162-1960)	0.0 (0.0-0.1)	31200 (6900-60200)	1.5 (0.3-3.0)
Mycobacterium tuberculosis	Resistance to one or more antibiotics	--	--	1 (0-1)	0.0 (0.0-0.1)	--	--	0 (0-0)	0.0 (0.0-0.0)	238 (157-368)	1.0 (0.6-1.5)	21200 (14000-32800)	85.9 (56.9-132.9)	92 (25-201)	0.4 (0.1-0.8)	8140 (2250-17800)	33.0 (9.1-71.9)	148 (96-225)	0.1 (0.1-0.2)	15400 (10200-23400)	13.6 (9.0-20.6)	56 (16-123)	0.0 (0.0-0.1)	4870 (1490-10600)	4.3 (1.3-9.3)	23900 (16800-34700)	1.2 (0.8-1.7)	791000 (573000-1150000)	39.2 (28.4-57.0)	8890 (2660-19000)	0.4 (0.1-0.9)	255000 (80900-537000)	12.6 (4.0-26.6)
Neisseria gonorrhoeae	Fluoroquinolones	--	--	--	--	--	--	--	--	--	--	--	--	--	--	--	--	--	--	--	--	--	--	--	--	--	--	15300 (8540-26000)	0.8 (0.4-1.3)	--	--	1450 (370-3180)	0.1 (0.0-0.2)
Neisseria gonorrhoeae	Third-generation cephalosporins	--	--	--	--	--	--	--	--	--	--	--	--	--	--	--	--	--	--	--	--	--	--	--	--	--	--	1110 (459-2220)	0.1 (0.0-0.1)	--	--	196 (49-451)	0.0 (0.0-0.0)
Neisseria gonorrhoeae	Resistance to one or more antibiotics	--	--	--	--	--	--	--	--	--	--	--	--	--	--	--	--	--	--	--	--	--	--	--	--	--	--	15300 (8560-26100)	0.8 (0.4-1.3)	--	--	1640 (564-3420)	0.1 (0.0-0.2)
Proteus spp.	Aminoglycosides	16 (10-24)	0.8 (0.5-1.2)	1450 (924-2190)	71.3 (45.4-107.5)	1 (0-2)	0.0 (0.0-0.1)	77 (8-170)	3.8 (0.4-8.3)	9 (5-13)	0.0 (0.0-0.1)	759 (451-1200)	3.1 (1.8-4.9)	0 (0-1)	0.0 (0.0-0.0)	39 (4-89)	0.2 (0.0-0.4)	4 (2-8)	0.0 (0.0-0.0)	391 (218-670)	0.3 (0.2-0.6)	0 (0-1)	0.0 (0.0-0.0)	20 (2-48)	0.0 (0.0-0.0)	1770 (1100-2620)	0.1 (0.1-0.1)	37800 (23000-56400)	1.9 (1.1-2.8)	88 (10-195)	0.0 (0.0-0.0)	1890 (225-4200)	0.1 (0.0-0.2)
Proteus spp.	Aminopenicillin	132 (85-197)	6.5 (4.2-9.7)	11800 (7570-17600)	580.0 (372.0-864.2)	3 (0-8)	0.1 (0.0-0.4)	235 (0-719)	11.5 (0.0-35.3)	72 (44-112)	0.3 (0.2-0.5)	6380 (3870-10000)	25.8 (15.7-40.6)	1 (0-4)	0.0 (0.0-0.0)	117 (0-368)	0.5 (0.0-1.5)	36 (20-60)	0.0 (0.0-0.1)	3140 (1780-5190)	2.8 (1.6-4.6)	1 (0-2)	0.0 (0.0-0.0)	59 (0-194)	0.1 (0.0-0.2)	14600 (9120-21900)	0.7 (0.5-1.1)	311000 (193000-472000)	15.4 (9.6-23.4)	259 (0-805)	0.0 (0.0-0.0)	5550 (0-17400)	0.3 (0.0-0.9)
Proteus spp.	Fluoroquinolones	43 (27-64)	2.1 (1.3-3.1)	3820 (2460-5710)	187.5 (120.7-280.4)	3 (1-6)	0.2 (0.1-0.3)	281 (114-546)	13.8 (5.6-26.8)	32 (19-52)	0.1 (0.1-0.2)	2830 (1670-4650)	11.4 (6.8-18.8)	2 (1-5)	0.0 (0.0-0.0)	214 (84-437)	0.9 (0.3-1.8)	14 (8-25)	0.0 (0.0-0.0)	1270 (719-2160)	1.1 (0.6-1.9)	1 (0-2)	0.0 (0.0-0.0)	95 (35-197)	0.1 (0.0-0.2)	7690 (4720-11700)	0.4 (0.2-0.6)	161000 (96700-248000)	8.0 (4.8-12.3)	588 (239-1110)	0.0 (0.0-0.1)	12200 (4930-23200)	0.6 (0.2-1.1)
Proteus spp.	Third-generation cephalosporins	48 (30-73)	2.4 (1.5-3.6)	4310 (2710-6530)	211.8 (133.4-320.8)	11 (3-21)	0.5 (0.2-1.0)	956 (304-1850)	47.0 (14.9-91.0)	30 (18-49)	0.1 (0.1-0.2)	2700 (1590-4370)	10.9 (6.4-17.7)	6 (2-13)	0.0 (0.0-0.1)	577 (181-1160)	2.3 (0.7-4.7)	14 (8-24)	0.0 (0.0-0.0)	1260 (691-2130)	1.1 (0.6-1.9)	3 (1-6)	0.0 (0.0-0.0)	271 (86-561)	0.2 (0.1-0.5)	6510 (3920-10200)	0.3 (0.2-0.5)	137000 (81000-215000)	6.8 (4.0-10.6)	1360 (436-2750)	0.1 (0.0-0.1)	28800 (9310-57500)	1.4 (0.5-2.8)
Proteus spp.	Trimethoprim-Sulfamethoxazole	86 (55-131)	4.2 (2.7-6.4)	7730 (4960-11700)	379.9 (243.7-572.8)	3 (0-8)	0.2 (0.0-0.4)	312 (0-705)	15.3 (0.0-34.6)	43 (26-67)	0.2 (0.1-0.3)	3850 (2340-6000)	15.6 (9.5-24.3)	2 (0-4)	0.0 (0.0-0.0)	148 (0-344)	0.6 (0.0-1.4)	23 (13-38)	0.0 (0.0-0.0)	2000 (1130-3320)	1.8 (1.0-2.9)	1 (0-2)	0.0 (0.0-0.0)	79 (0-187)	0.1 (0.0-0.2)	8260 (5100-12300)	0.4 (0.3-0.6)	177000 (108000-268000)	8.8 (5.4-13.3)	304 (0-683)	0.0 (0.0-0.0)	6560 (0-14900)	0.3 (0.0-0.7)
Proteus spp.	Resistance to one or more antibiotics	149 (96-221)	7.3 (4.7-10.8)	13300 (8570-19700)	653.2 (420.9-969.7)	21 (10-36)	1.0 (0.5-1.8)	1860 (916-3220)	91.4 (45.0-158.2)	82 (49-127)	0.3 (0.2-0.5)	7270 (4380-11400)	29.4 (17.7-46.2)	12 (6-22)	0.0 (0.0-0.1)	1100 (517-1930)	4.4 (2.1-7.8)	41 (23-68)	0.0 (0.0-0.1)	3570 (2020-5880)	3.1 (1.8-5.2)	6 (3-11)	0.0 (0.0-0.0)	523 (233-967)	0.5 (0.2-0.9)	16600 (10400-24600)	0.8 (0.5-1.2)	354000 (218000-536000)	17.5 (10.8-26.6)	2600 (1300-4450)	0.1 (0.1-0.2)	55000 (27400-94100)	2.7 (1.4-4.7)
Pseudomonas aeruginosa	Aminoglycosides	653 (453-922)	32.1 (22.3-45.3)	58100 (40400-82100)	2855.5 (1984.4-4035.5)	19 (0-47)	0.9 (0.0-2.3)	1690 (0-4220)	83.1 (0.0-207.4)	239 (182-312)	1.0 (0.7-1.3)	21100 (16100-27600)	85.6 (65.2-111.7)	7 (0-16)	0.0 (0.0-0.1)	613 (0-1450)	2.5 (0.0-5.9)	93 (66-134)	0.1 (0.1-0.1)	8070 (5710-11600)	7.1 (5.0-10.2)	3 (0-7)	0.0 (0.0-0.0)	235 (0-573)	0.2 (0.0-0.5)	9080 (5790-13500)	0.4 (0.3-0.7)	206000 (128000-311000)	10.2 (6.3-15.4)	265 (0-674)	0.0 (0.0-0.0)	6070 (0-15400)	0.3 (0.0-0.8)
Pseudomonas aeruginosa	Anti-pseudomonal penicillin/Beta-Lactamase inhibitors	948 (652-1320)	46.6 (32.0-64.9)	84400 (58000-118000)	4147.2 (2850.7-5782.1)	83 (51-131)	4.1 (2.5-6.4)	7430 (4550-11600)	364.9 (223.5-571.4)	410 (314-523)	1.7 (1.3-2.1)	36200 (27800-46300)	146.7 (112.5-187.5)	36 (23-54)	0.1 (0.1-0.2)	3210 (2070-4750)	13.0 (8.4-19.2)	157 (113-219)	0.1 (0.1-0.2)	13600 (9780-18900)	12.0 (8.6-16.6)	14 (9-21)	0.0 (0.0-0.0)	1190 (737-1830)	1.0 (0.6-1.6)	20200 (12700-30400)	1.0 (0.6-1.5)	443000 (273000-670000)	22.0 (13.5-33.2)	1630 (873-2810)	0.1 (0.0-0.1)	35900 (19000-62200)	1.8 (0.9-3.1)
Pseudomonas aeruginosa	Carbapenem	1270 (882-1760)	62.4 (43.3-86.6)	113000 (78500-157000)	5556.1 (3857.6-7716.9)	243 (158-357)	11.9 (7.8-17.6)	21600 (14100-31800)	1063.2 (691.9-1562.2)	556 (433-711)	2.3 (1.8-2.9)	49300 (38400-62900)	199.4 (155.3-254.8)	107 (76-150)	0.4 (0.3-0.6)	9510 (6700-13300)	38.5 (27.1-53.9)	215 (154-297)	0.2 (0.1-0.3)	18600 (13300-25700)	16.4 (11.7-22.6)	41 (27-61)	0.0 (0.0-0.1)	3580 (2380-5310)	3.1 (2.1-4.7)	31700 (20000-46900)	1.6 (1.0-2.3)	692000 (426000-1040000)	34.3 (21.1-51.7)	6210 (3720-9680)	0.3 (0.2-0.5)	136000 (79200-213000)	6.7 (3.9-10.5)
Pseudomonas aeruginosa	Fluoroquinolones	842 (573-1180)	41.4 (28.2-58.0)	75000 (51100-105000)	3687.0 (2509.1-5167.9)	90 (58-136)	4.4 (2.8-6.7)	8070 (5150-12100)	396.3 (253.2-594.7)	366 (282-466)	1.5 (1.1-1.9)	32400 (25000-41200)	131.3 (101.3-166.9)	40 (28-54)	0.2 (0.1-0.2)	3520 (2450-4820)	14.2 (9.9-19.5)	142 (101-198)	0.1 (0.1-0.2)	12300 (8750-17100)	10.8 (7.7-15.1)	15 (10-23)	0.0 (0.0-0.0)	1330 (877-1970)	1.2 (0.8-1.7)	20200 (12800-30200)	1.0 (0.6-1.5)	442000 (272000-670000)	21.9 (13.5-33.2)	2230 (1270-3590)	0.1 (0.1-0.2)	48800 (27100-79400)	2.4 (1.3-3.9)
Pseudomonas aeruginosa	Fourth-generation cephalosporins	793 (549-1120)	39.0 (27.0-55.2)	70600 (48800-100000)	3470.9 (2399.1-4918.1)	18 (10-31)	0.9 (0.5-1.5)	1600 (877-2720)	78.7 (43.1-133.9)	337 (257-435)	1.4 (1.0-1.8)	29800 (22800-38600)	120.8 (92.3-156.1)	8 (4-12)	0.0 (0.0-0.0)	667 (383-1060)	2.7 (1.6-4.3)	130 (93-181)	0.1 (0.1-0.2)	11200 (8020-15600)	9.9 (7.0-13.7)	3 (2-5)	0.0 (0.0-0.0)	245 (134-403)	0.2 (0.1-0.4)	17000 (10600-25800)	0.8 (0.5-1.3)	374000 (231000-574000)	18.5 (11.4-28.4)	391 (196-718)	0.0 (0.0-0.0)	8620 (4320-15900)	0.4 (0.2-0.8)
Pseudomonas aeruginosa	Third-generation cephalosporins	1050 (704-1470)	51.4 (34.6-72.4)	93300 (62800-131000)	4584.2 (3083.7-6451.4)	58 (25-103)	2.9 (1.2-5.1)	5180 (2220-9200)	254.4 (108.9-451.9)	491 (370-638)	2.0 (1.5-2.6)	43500 (32800-56500)	176.2 (132.6-228.6)	32 (15-53)	0.1 (0.1-0.2)	2830 (1290-4730)	11.5 (5.2-19.1)	186 (129-260)	0.2 (0.1-0.2)	16100 (11200-22400)	14.1 (9.9-19.7)	11 (5-19)	0.0 (0.0-0.0)	991 (427-1660)	0.9 (0.4-1.5)	32600 (20000-49700)	1.6 (1.0-2.5)	705000 (423000-1090000)	34.9 (20.9-53.9)	2730 (1100-5030)	0.1 (0.1-0.2)	58200 (23100-107000)	2.9 (1.1-5.3)
Pseudomonas aeruginosa	Resistance to one or more antibiotics	2120 (1470-2910)	104.0 (72.3-143.1)	189000 (131000-260000)	9269.4 (6440.1-12759.3)	512 (332-762)	25.1 (16.3-37.4)	45600 (29700-67900)	2240.6 (1457.2-3337.6)	938 (740-1190)	3.8 (3.0-4.8)	83100 (65500-105000)	336.3 (265.1-425.0)	230 (163-319)	0.9 (0.7-1.3)	20400 (14500-28200)	82.4 (58.6-114.4)	357 (259-493)	0.3 (0.2-0.4)	30900 (22400-42600)	27.2 (19.7-37.5)	87 (57-131)	0.1 (0.0-0.1)	7570 (4930-11300)	6.7 (4.3-9.9)	53500 (34100-79500)	2.6 (1.7-3.9)	1170000 (720000-1760000)	57.8 (35.7-86.9)	13500 (7730-21600)	0.7 (0.4-1.1)	293000 (165000-472000)	14.5 (8.2-23.4)
Salmonella Paratyphi	Fluoroquinolones	--	--	0 (0-0)	0.0 (0.0-0.0)	--	--	0 (0-0)	0.0 (0.0-0.0)	1 (0-2)	0.0 (0.0-0.0)	55 (16-148)	0.2 (0.1-0.6)	0 (0-0)	0.0 (0.0-0.0)	11 (1-35)	0.0 (0.0-0.1)	50 (15-121)	0.0 (0.0-0.1)	4300 (1260-10400)	3.8 (1.1-9.1)	10 (1-30)	0.0 (0.0-0.0)	880 (111-2580)	0.8 (0.1-2.3)	265 (104-543)	0.0 (0.0-0.0)	17500 (6670-36200)	0.9 (0.3-1.8)	55 (7-141)	0.0 (0.0-0.0)	3580 (458-9070)	0.2 (0.0-0.4)
Salmonella Paratyphi	Multi-drug resistance in Salmonella Typhi and Paratyphi	--	--	0 (0-0)	0.0 (0.0-0.0)	--	--	0 (0-0)	0.0 (0.0-0.0)	0 (0-0)	0.0 (0.0-0.0)	1 (0-2)	0.0 (0.0-0.0)	0 (0-0)	0.0 (0.0-0.0)	0 (0-0)	0.0 (0.0-0.0)	1 (0-3)	0.0 (0.0-0.0)	107 (29-265)	0.1 (0.0-0.2)	0 (0-1)	0.0 (0.0-0.0)	15 (0-49)	0.0 (0.0-0.0)	9 (3-18)	0.0 (0.0-0.0)	605 (227-1290)	0.0 (0.0-0.1)	1 (0-4)	0.0 (0.0-0.0)	86 (0-259)	0.0 (0.0-0.0)
Salmonella Paratyphi	Resistance to one or more antibiotics	--	--	0 (0-0)	0.0 (0.0-0.0)	--	--	0 (0-0)	0.0 (0.0-0.0)	1 (0-2)	0.0 (0.0-0.0)	56 (16-150)	0.2 (0.1-0.6)	0 (0-0)	0.0 (0.0-0.0)	11 (1-36)	0.0 (0.0-0.1)	51 (15-123)	0.0 (0.0-0.1)	4410 (1290-10600)	3.9 (1.1-9.3)	10 (1-30)	0.0 (0.0-0.0)	895 (129-2630)	0.8 (0.1-2.3)	274 (107-563)	0.0 (0.0-0.0)	18100 (6890-37300)	0.9 (0.3-1.8)	56 (8-143)	0.0 (0.0-0.0)	3660 (540-9230)	0.2 (0.0-0.5)
Salmonella Typhi	Fluoroquinolones	231 (114-421)	11.4 (5.6-20.7)	20600 (10200-37500)	1011.1 (499.4-1840.1)	46 (7-116)	2.3 (0.4-5.7)	4110 (648-10300)	201.8 (31.9-505.8)	70 (34-128)	0.3 (0.1-0.5)	6160 (2970-11300)	24.9 (12.0-45.7)	14 (2-34)	0.1 (0.0-0.1)	1230 (199-2980)	5.0 (0.8-12.1)	299 (100-699)	0.3 (0.1-0.6)	25800 (8620-60300)	22.7 (7.6-53.0)	59 (7-168)	0.1 (0.0-0.1)	5120 (568-14500)	4.5 (0.5-12.7)	2530 (1200-4800)	0.1 (0.1-0.2)	151000 (66700-294000)	7.5 (3.3-14.6)	506 (73-1270)	0.0 (0.0-0.1)	29900 (4120-76900)	1.5 (0.2-3.8)
Salmonella Typhi	Multi-drug resistance in Salmonella Typhi and Paratyphi	63 (32-111)	3.1 (1.6-5.5)	5640 (2880-9870)	277.1 (141.4-485.1)	7 (0-20)	0.3 (0.0-1.0)	612 (0-1750)	30.1 (0.0-85.9)	18 (9-32)	0.1 (0.0-0.1)	1560 (785-2800)	6.3 (3.2-11.3)	2 (0-6)	0.0 (0.0-0.0)	174 (0-512)	0.7 (0.0-2.1)	95 (33-217)	0.1 (0.0-0.2)	8250 (2880-18800)	7.3 (2.5-16.5)	11 (0-34)	0.0 (0.0-0.0)	939 (0-2910)	0.8 (0.0-2.6)	737 (356-1360)	0.0 (0.0-0.1)	44700 (19800-84500)	2.2 (1.0-4.2)	85 (0-237)	0.0 (0.0-0.0)	5120 (0-14500)	0.3 (0.0-0.7)
Salmonella Typhi	Resistance to one or more antibiotics	266 (138-460)	13.1 (6.8-22.6)	23600 (12300-40900)	1161.1 (604.8-2010.0)	53 (11-123)	2.6 (0.6-6.0)	4720 (1020-10900)	231.9 (50.0-535.9)	80 (42-141)	0.3 (0.2-0.6)	7080 (3670-12500)	28.7 (14.9-50.5)	16 (4-36)	0.1 (0.0-0.1)	1400 (317-3200)	5.7 (1.3-13.0)	362 (125-808)	0.3 (0.1-0.7)	31200 (10700-69800)	27.5 (9.5-61.4)	70 (13-196)	0.1 (0.0-0.2)	6060 (1080-16900)	5.3 (1.0-14.8)	3020 (1490-5530)	0.1 (0.1-0.3)	181000 (82400-341000)	9.0 (4.1-16.9)	591 (125-1350)	0.0 (0.0-0.1)	35100 (7250-85600)	1.7 (0.4-4.2)
Non-typhoidal *Salmonella*	Fluoroquinolones	141 (87-218)	6.9 (4.3-10.7)	13000 (7920-20000)	639.1 (389.2-981.3)	29 (5-64)	1.4 (0.2-3.1)	2620 (471-5670)	128.7 (23.1-278.7)	211 (106-374)	0.9 (0.4-1.5)	24500 (11000-44300)	99.1 (44.5-179.2)	44 (8-105)	0.2 (0.0-0.4)	4180 (913-9890)	16.9 (3.7-40.0)	109 (53-196)	0.1 (0.0-0.2)	21700 (4550-56800)	19.1 (4.0-50.0)	23 (4-54)	0.0 (0.0-0.0)	2660 (321-7570)	2.3 (0.3-6.7)	4100 (2290-6840)	0.2 (0.1-0.3)	188000 (68800-454000)	9.3 (3.4-22.5)	847 (141-1890)	0.0 (0.0-0.1)	27800 (5330-64300)	1.4 (0.3-3.2)
Non-typhoidal *Salmonella*	Resistance to one or more antibiotics	141 (87-218)	6.9 (4.3-10.7)	13000 (7920-20000)	639.1 (389.2-981.3)	29 (5-64)	1.4 (0.2-3.1)	2620 (471-5670)	128.7 (23.1-278.7)	211 (106-374)	0.9 (0.4-1.5)	24500 (11000-44300)	99.1 (44.5-179.2)	44 (8-105)	0.2 (0.0-0.4)	4180 (913-9890)	16.9 (3.7-40.0)	109 (53-196)	0.1 (0.0-0.2)	21700 (4550-56800)	19.1 (4.0-50.0)	23 (4-54)	0.0 (0.0-0.0)	2660 (321-7570)	2.3 (0.3-6.7)	4100 (2290-6840)	0.2 (0.1-0.3)	188000 (68800-454000)	9.3 (3.4-22.5)	847 (141-1890)	0.0 (0.0-0.1)	27800 (5330-64300)	1.4 (0.3-3.2)
Serratia spp.	Aminoglycosides	100 (61-161)	4.9 (3.0-7.9)	8940 (5400-14300)	439.0 (265.1-703.9)	6 (0-16)	0.3 (0.0-0.8)	519 (0-1450)	25.5 (0.0-71.2)	21 (12-34)	0.1 (0.0-0.1)	1870 (1080-3030)	7.6 (4.4-12.3)	1 (0-4)	0.0 (0.0-0.0)	110 (0-319)	0.4 (0.0-1.3)	8 (4-14)	0.0 (0.0-0.0)	692 (360-1220)	0.6 (0.3-1.1)	0 (0-1)	0.0 (0.0-0.0)	40 (0-118)	0.0 (0.0-0.1)	1400 (763-2320)	0.1 (0.0-0.1)	35200 (19200-58200)	1.7 (0.9-2.9)	83 (0-232)	0.0 (0.0-0.0)	2090 (0-5830)	0.1 (0.0-0.3)
Serratia spp.	Anti-pseudomonal penicillin/Beta-Lactamase inhibitors	103 (63-167)	5.1 (3.1-8.2)	9180 (5600-14800)	451.1 (275.2-729.4)	18 (9-33)	0.9 (0.4-1.6)	1600 (782-2900)	78.6 (38.4-142.5)	27 (15-44)	0.1 (0.1-0.2)	2420 (1370-3890)	9.8 (5.6-15.8)	5 (2-10)	0.0 (0.0-0.0)	449 (210-856)	1.8 (0.8-3.5)	10 (5-18)	0.0 (0.0-0.0)	885 (458-1580)	0.8 (0.4-1.4)	2 (1-4)	0.0 (0.0-0.0)	164 (70-325)	0.1 (0.1-0.3)	2410 (1300-4070)	0.1 (0.1-0.2)	59600 (31800-99900)	3.0 (1.6-4.9)	479 (218-914)	0.0 (0.0-0.0)	11800 (5380-22200)	0.6 (0.3-1.1)
Serratia spp.	Carbapenem	134 (83-206)	6.6 (4.1-10.1)	11900 (7420-18300)	586.9 (364.4-899.5)	37 (19-64)	1.8 (0.9-3.1)	3280 (1690-5680)	161.4 (82.8-279.0)	28 (16-44)	0.1 (0.1-0.2)	2470 (1440-3900)	10.0 (5.8-15.8)	8 (4-14)	0.0 (0.0-0.1)	682 (329-1240)	2.8 (1.3-5.0)	10 (5-17)	0.0 (0.0-0.0)	874 (452-1500)	0.8 (0.4-1.3)	3 (1-5)	0.0 (0.0-0.0)	241 (107-460)	0.2 (0.1-0.4)	1670 (940-2800)	0.1 (0.0-0.1)	42100 (24000-70000)	2.1 (1.2-3.5)	462 (220-879)	0.0 (0.0-0.0)	11700 (5590-21700)	0.6 (0.3-1.1)
Serratia spp.	Fluoroquinolones	115 (67-190)	5.7 (3.3-9.3)	10300 (5970-16900)	504.0 (293.2-831.6)	13 (2-29)	0.6 (0.1-1.4)	1170 (191-2620)	57.6 (9.4-128.7)	24 (14-40)	0.1 (0.1-0.2)	2140 (1240-3520)	8.7 (5.0-14.3)	3 (0-6)	0.0 (0.0-0.0)	242 (43-542)	1.0 (0.2-2.2)	9 (5-16)	0.0 (0.0-0.0)	792 (410-1420)	0.7 (0.4-1.2)	1 (0-3)	0.0 (0.0-0.0)	90 (17-215)	0.1 (0.0-0.2)	1630 (888-2790)	0.1 (0.0-0.1)	40800 (22400-70000)	2.0 (1.1-3.5)	184 (28-428)	0.0 (0.0-0.0)	4640 (751-10600)	0.2 (0.0-0.5)
Serratia spp.	Fourth-generation cephalosporins	126 (75-203)	6.2 (3.7-10.0)	11300 (6650-18100)	553.2 (326.6-887.7)	9 (4-20)	0.5 (0.2-1.0)	843 (317-1790)	41.4 (15.6-88.1)	28 (16-43)	0.1 (0.1-0.2)	2440 (1380-3840)	9.9 (5.6-15.5)	2 (1-4)	0.0 (0.0-0.0)	181 (70-373)	0.7 (0.3-1.5)	10 (5-18)	0.0 (0.0-0.0)	898 (458-1560)	0.8 (0.4-1.4)	1 (0-2)	0.0 (0.0-0.0)	67 (24-146)	0.1 (0.0-0.1)	1950 (1060-3280)	0.1 (0.1-0.2)	48800 (26200-82500)	2.4 (1.3-4.1)	152 (58-329)	0.0 (0.0-0.0)	3800 (1470-8160)	0.2 (0.1-0.4)

Serratia spp.	Third-generation cephalosporins	182 (107-297)	9.0 (5.2-14.6)	16200 (9500-26500)	797.8 (466.9-1300.5)	12 (4-28)	0.6 (0.2-1.4)	1030 (338-2470)	50.6 (16.6-121.1)	45 (27-72)	0.2 (0.1-0.3)	3970 (2360-6400)	16.1 (9.6-25.9)	3 (1-6)	0.0 (0.0-0.0)	251 (86-538)	1.0 (0.3-2.2)	16 (8-29)	0.0 (0.0-0.0)	1410 (728-2500)	1.2 (0.6-2.2)	1 (0-2)	0.0 (0.0-0.0)	85 (26-195)	0.1 (0.0-0.2)	3680 (2040-6110)	0.2 (0.1-0.3)	91300 (49800-151000)	4.5 (2.5-7.5)	236 (71-514)	0.0 (0.0-0.0)	5850 (1820-13000)	0.3 (0.1-0.6)
Serratia spp.	Resistance to one or more antibiotics	373 (240-569)	18.4 (11.8-28.0)	33200 (21400-50600)	1633.5 (1049.3-2487.3)	95 (55-152)	4.7 (2.7-7.5)	8450 (4900-13500)	415.1 (240.8-665.6)	84 (52-132)	0.3 (0.2-0.5)	7480 (4610-11700)	30.3 (18.7-47.4)	22 (12-35)	0.1 (0.0-0.1)	1920 (1070-3130)	7.8 (4.3-12.7)	31 (17-54)	0.0 (0.0-0.0)	2700 (1420-4640)	2.4 (1.3-4.1)	8 (4-14)	0.0 (0.0-0.0)	687 (336-1230)	0.6 (0.3-1.1)	6210 (3540-10100)	0.3 (0.2-0.5)	155000 (88500-250000)	7.7 (4.4-12.4)	1600 (832-2800)	0.1 (0.0-0.1)	39800 (21100-69400)	2.0 (1.0-3.4)
Shigella spp.	Fluoroquinolones	11 (4-24)	0.5 (0.2-1.2)	1050 (364-2300)	51.6 (17.9-112.9)	2 (0-6)	0.1 (0.0-0.3)	210 (26-559)	10.3 (1.3-27.5)	96 (34-200)	0.4 (0.1-0.8)	9460 (3330-19700)	38.3 (13.5-79.7)	20 (2-54)	0.1 (0.0-0.2)	1850 (251-5050)	7.5 (1.0-20.4)	38 (13-84)	0.0 (0.0-0.1)	6380 (1950-14900)	5.6 (1.7-13.1)	8 (1-21)	0.0 (0.0-0.0)	1020 (187-2840)	0.9 (0.2-2.5)	257 (85-595)	0.0 (0.0-0.0)	24500 (7710-61400)	1.2 (0.4-3.0)	52 (5-142)	0.0 (0.0-0.0)	3280 (127-9720)	0.2 (0.0-0.5)
Shigella spp.	Resistance to one or more antibiotics	11 (4-24)	0.5 (0.2-1.2)	1050 (364-2300)	51.6 (17.9-112.9)	2 (0-6)	0.1 (0.0-0.3)	210 (26-559)	10.3 (1.3-27.5)	96 (34-200)	0.4 (0.1-0.8)	9460 (3330-19700)	38.3 (13.5-79.7)	20 (2-54)	0.1 (0.0-0.2)	1850 (251-5050)	7.5 (1.0-20.4)	38 (13-84)	0.0 (0.0-0.1)	6380 (1950-14900)	5.6 (1.7-13.1)	8 (1-21)	0.0 (0.0-0.0)	1020 (187-2840)	0.9 (0.2-2.5)	257 (85-595)	0.0 (0.0-0.0)	24500 (7710-61400)	1.2 (0.4-3.0)	52 (5-142)	0.0 (0.0-0.0)	3280 (127-9720)	0.2 (0.0-0.5)
Staphylococcus aureus	Fluoroquinolones	1220 (846-1730)	60.0 (41.6-85.0)	109000 (75500-154000)	5353.4 (3708.0-7580.1)	56 (24-97)	2.7 (1.2-4.8)	4960 (2130-8670)	243.6 (104.8-425.9)	1020 (783-1330)	4.1 (3.2-5.4)	90500 (69400-118000)	366.3 (281.0-478.8)	46 (20-78)	0.2 (0.1-0.3)	4110 (1820-6930)	16.6 (7.4-28.0)	402 (293-559)	0.4 (0.3-0.5)	35100 (25500-48600)	30.8 (22.4-42.7)	18 (8-32)	0.0 (0.0-0.0)	1600 (724-2790)	1.4 (0.6-2.4)	58900 (37200-88700)	2.9 (1.8-4.4)	1330000 (822000-2030000)	65.9 (40.7-100.4)	2630 (1120-4710)	0.1 (0.1-0.2)	59400 (25100-108000)	2.9 (1.2-5.4)
Staphylococcus aureus	Macrolide	1800 (1270-2510)	88.2 (62.4-123.3)	160000 (113000-224000)	7871.1 (5565.1-11005.9)	72 (28-129)	3.5 (1.4-6.4)	6390 (2480-11500)	314.1 (121.6-567.1)	1830 (1440-2360)	7.4 (5.8-9.5)	163000 (127000-209000)	658.1 (516.0-845.8)	74 (30-128)	0.3 (0.1-0.5)	6590 (2700-11300)	26.7 (10.9-45.9)	657 (481-904)	0.6 (0.4-0.8)	57300 (42200-78500)	50.4 (37.1-69.0)	26 (10-47)	0.0 (0.0-0.0)	2310 (921-4090)	2.0 (0.8-3.6)	123000 (78900-185000)	6.1 (3.9-9.1)	2720000 (1690000-4170000)	134.5 (83.6-206.5)	4990 (1770-9770)	0.2 (0.1-0.5)	110000 (38200-217000)	5.5 (1.9-10.8)
Staphylococcus aureus	Methicillin	2570 (1810-3610)	126.4 (88.7-177.2)	229000 (161000-321000)	11268.2 (7911.0-15783.0)	706 (340-1230)	34.7 (16.7-60.3)	62900 (30300-109000)	3089.2 (1487.6-5366.7)	2110 (1650-2680)	8.5 (6.7-10.8)	187000 (146000-237000)	757.1 (591.6-960.4)	576 (272-935)	2.3 (1.1-3.8)	51000 (24000-82700)	206.6 (97.3-334.9)	877 (647-1180)	0.8 (0.6-1.0)	76200 (56300-103000)	67.0 (49.5-90.1)	242 (115-402)	0.2 (0.1-0.4)	20900 (10000-34700)	18.4 (8.8-30.6)	102000 (66900-151000)	5.1 (3.3-7.5)	2290000 (1460000-3460000)	113.6 (72.5-171.4)	27000 (11600-48000)	1.3 (0.6-2.4)	607000 (259000-1090000)	30.1 (12.8-54.0)
Staphylococcus aureus	Trimethoprim-Sulfamethoxazole	610 (425-846)	30.0 (20.9-41.5)	54300 (37800-75300)	2669.1 (1858.2-3700.6)	57 (29-93)	2.8 (1.4-4.6)	5100 (2550-8260)	250.5 (125.2-406.0)	424 (330-536)	1.7 (1.3-2.2)	37600 (29200-47400)	152.1 (118.4-192.1)	39 (21-61)	0.2 (0.1-0.2)	3470 (1850-5370)	14.1 (7.5-21.7)	179 (130-247)	0.2 (0.1-0.2)	15500 (11300-21400)	13.6 (10.0-18.8)	17 (8-26)	0.0 (0.0-0.0)	1430 (728-2280)	1.3 (0.6-2.0)	17400 (11600-25100)	0.9 (0.6-1.2)	400000 (259000-592000)	19.8 (12.8-29.3)	1540 (774-2540)	0.1 (0.0-0.1)	35300 (17500-58600)	1.7 (0.9-2.9)
Staphylococcus aureus	Vancomycin	54 (37-76)	2.6 (1.8-3.7)	4800 (3300-6800)	235.7 (161.9-333.8)	16 (8-28)	0.8 (0.4-1.4)	1400 (697-2500)	68.9 (34.2-122.8)	44 (34-59)	0.2 (0.1-0.2)	3940 (2980-5270)	15.9 (12.1-21.3)	13 (7-22)	0.1 (0.0-0.1)	1150 (614-1980)	4.7 (2.5-8.0)	18 (13-25)	0.0 (0.0-0.0)	1560 (1130-2180)	1.4 (1.0-1.9)	5 (3-9)	0.0 (0.0-0.0)	450 (237-791)	0.4 (0.2-0.7)	2670 (1730-4000)	0.1 (0.1-0.2)	61300 (39000-93300)	3.0 (1.9-4.6)	787 (385-1460)	0.0 (0.0-0.1)	18100 (8850-33800)	0.9 (0.4-1.7)
Staphylococcus aureus	Resistance to one or more antibiotics	3400 (2420-4720)	167.1 (118.7-231.8)	303000 (215000-421000)	14893.3 (10578.7-20668.1)	907 (505-1460)	44.5 (24.8-71.5)	80700 (45100-130000)	3966.3 (2213.8-6366.2)	2940 (2330-3700)	11.9 (9.4-15.0)	260000 (207000-328000)	1053.5 (837.0-1328.9)	749 (429-1140)	3.0 (1.7-4.6)	66400 (38000-101000)	268.7 (153.9-408.8)	1170 (877-1540)	1.0 (0.8-1.4)	102000 (76200-134000)	89.6 (67.0-117.9)	308 (170-492)	0.3 (0.1-0.4)	26700 (14900-42500)	23.5 (13.1-37.4)	156000 (102000-229000)	7.7 (5.0-11.3)	3490000 (2240000-5240000)	173.1 (110.8-259.5)	37000 (19700-61100)	1.8 (1.0-3.0)	830000 (431000-1390000)	41.1 (21.4-68.8)
Streptococcus pneumoniae	Beta Lactam/Beta-lactamase inhibitors	732 (498-1090)	36.0 (24.5-53.3)	65200 (44300-96700)	3204.1 (2177.9-4750.9)	17 (0-47)	0.9 (0.0-2.3)	1540 (27-4140)	75.6 (1.3-203.6)	2070 (1610-2660)	8.4 (6.5-10.7)	183000 (143000-235000)	742.2 (577.5-951.0)	48 (1-124)	0.2 (0.0-0.5)	4220 (64-10900)	17.1 (0.3-44.3)	712 (530-962)	0.6 (0.5-0.8)	61700 (46000-83200)	54.2 (40.4-73.2)	18 (0-45)	0.0 (0.0-0.0)	1520 (25-3880)	1.3 (0.0-3.4)	22700 (15400-32800)	1.1 (0.8-1.6)	495000 (331000-718000)	24.5 (16.4-35.5)	339 (4-985)	0.0 (0.0-0.0)	7620 (127-21500)	0.4 (0.0-1.1)
Streptococcus pneumoniae	Carbapenem	1060 (702-1520)	51.9 (34.5-74.7)	94000 (62500-135000)	4619.3 (3071.6-6652.5)	231 (103-412)	11.4 (5.1-20.2)	20600 (9200-36700)	1011.6 (451.8-1801.6)	2990 (2350-3720)	12.1 (9.5-15.1)	265000 (208000-330000)	1071.7 (842.0-1334.1)	649 (309-1100)	2.6 (1.2-4.4)	57500 (27400-97000)	232.6 (110.7-392.7)	979 (727-1280)	0.9 (0.6-1.1)	85000 (63300-112000)	74.7 (55.6-98.0)	215 (102-373)	0.2 (0.1-0.3)	18700 (8910-32300)	16.4 (7.8-28.4)	39700 (28300-56500)	2.0 (1.4-2.8)	854000 (608000-1220000)	42.3 (30.1-60.6)	8550 (3920-15000)	0.4 (0.2-0.7)	185000 (86200-325000)	9.2 (4.3-16.1)
Streptococcus pneumoniae	Fluoroquinolones	283 (184-413)	13.9 (9.0-20.3)	25200 (16300-36800)	1236.5 (802.9-1806.0)	35 (8-75)	1.7 (0.4-3.7)	3080 (692-6650)	151.5 (34.0-327.0)	699 (531-904)	2.8 (2.2-3.7)	61900 (47000-80000)	250.4 (190.2-323.8)	84 (19-174)	0.3 (0.1-0.7)	7410 (1680-15400)	30.0 (6.8-62.4)	252 (183-340)	0.2 (0.2-0.3)	21800 (15800-29400)	19.2 (13.9-25.9)	31 (7-64)	0.0 (0.0-0.1)	2670 (636-5540)	2.3 (0.6-4.9)	5580 (3780-8100)	0.3 (0.2-0.4)	128000 (87100-183000)	6.3 (4.3-9.1)	601 (140-1310)	0.0 (0.0-0.1)	14000 (3260-30500)	0.7 (0.2-1.5)
Streptococcus pneumoniae	Macrolide	2150 (1450-3030)	105.5 (71.5-148.9)	191000 (130000-270000)	9401.2 (6366.6-13265.0)	81 (0-215)	4.0 (0.0-10.6)	7240 (0-19100)	355.5 (0.0-937.3)	6250 (5220-7530)	25.3 (21.1-30.5)	553000 (462000-666000)	2240.9 (1869.8-2696.6)	239 (0-591)	1.0 (0.0-2.4)	21100 (0-52200)	85.5 (0.0-211.5)	2020 (1590-2590)	1.8 (1.4-2.3)	176000 (138000-225000)	154.6 (121.3-197.6)	77 (0-197)	0.1 (0.0-0.2)	6630 (0-17000)	5.8 (0.0-14.9)	82900 (60800-116000)	4.1 (3.0-5.7)	1770000 (1270000-2460000)	87.7 (62.7-121.7)	3190 (0-8600)	0.2 (0.0-0.4)	67000 (0-183000)	3.3 (0.0-9.1)
Streptococcus pneumoniae	Penicillin	1470 (949-2140)	72.0 (46.6-105.1)	131000 (84500-191000)	6415.4 (4153.5-9363.9)	82 (40-149)	4.0 (2.0-7.3)	7310 (3560-13200)	359.1 (175.0-649.9)	3970 (3080-4970)	16.1 (12.5-20.1)	352000 (272000-440000)	1423.7 (1103.2-1779.5)	215 (111-359)	0.9 (0.4-1.5)	19000 (9830-31700)	77.0 (39.8-128.5)	1340 (991-1770)	1.2 (0.9-1.6)	116000 (86000-154000)	101.8 (75.6-135.2)	73 (37-126)	0.1 (0.0-0.1)	6350 (3190-10900)	5.6 (2.8-9.6)	43700 (30300-63200)	2.2 (1.5-3.1)	957000 (661000-1390000)	47.4 (32.7-68.8)	1930 (857-3580)	0.1 (0.0-0.2)	43200 (19600-79100)	2.1 (1.0-3.9)
Streptococcus pneumoniae	Third-generation cephalosporins	569 (375-841)	28.0 (18.4-41.3)	50700 (33400-74900)	2489.0 (1640.9-3678.3)	27 (11-57)	1.3 (0.5-2.8)	2450 (954-5060)	120.1 (46.9-248.4)	1450 (1130-1900)	5.9 (4.6-7.7)	128000 (100000-168000)	519.5 (406.6-680.7)	61 (25-115)	0.2 (0.1-0.5)	5370 (2190-10200)	21.7 (8.9-41.1)	516 (381-711)	0.5 (0.3-0.6)	44700 (33100-61400)	39.3 (29.1-54.0)	25 (10-50)	0.0 (0.0-0.0)	2170 (875-4290)	1.9 (0.8-3.8)	15900 (11200-22800)	0.8 (0.6-1.1)	355000 (246000-507000)	17.6 (12.2-25.1)	409 (157-818)	0.0 (0.0-0.0)	10200 (4090-20600)	0.5 (0.2-1.0)
Streptococcus pneumoniae	Trimethoprim-Sulfamethoxazole	3280 (2210-4560)	161.0 (108.7-224.2)	292000 (197000-406000)	14339.4 (9682.4-19963.9)	295 (42-612)	14.5 (2.1-30.1)	26200 (3800-54600)	1288.6 (186.9-2681.0)	8100 (6660-9870)	32.8 (27.0-40.0)	717000 (589000-873000)	2903.5 (2386.0-3534.5)	704 (100-1410)	2.8 (0.4-5.7)	62200 (8850-125000)	252.0 (35.8-504.1)	2850 (2200-3700)	2.5 (1.9-3.3)	247000 (191000-320000)	217.4 (168.0-281.6)	252 (36-516)	0.2 (0.0-0.5)	21800 (3100-44400)	19.1 (2.7-39.0)	78800 (57800-109000)	3.9 (2.9-5.4)	1760000 (1280000-2430000)	87.1 (63.2-120.2)	6330 (926-13200)	0.3 (0.0-0.7)	142000 (21200-295000)	7.0 (1.0-14.6)
Streptococcus pneumoniae	Resistance to one or more antibiotics	3910 (2660-5430)	192.3 (130.8-266.9)	349000 (237000-484000)	17130.4 (11654.4-23758.9)	769 (466-1150)	37.8 (22.9-56.4)	68400 (41500-102000)	3362.1 (2036.7-5020.5)	9990 (8400-12000)	40.4 (34.0-48.6)	884000 (744000-1060000)	3578.2 (3010.6-4299.3)	2000 (1410-2700)	8.1 (5.7-10.9)	177000 (125000-239000)	716.0 (504.9-966.9)	3480 (2710-4490)	3.1 (2.4-3.9)	302000 (236000-388000)	265.1 (207.2-341.3)	691 (465-973)	0.6 (0.4-0.9)	59800 (40400-84200)	52.5 (35.5-74.0)	102000 (75600-140000)	5.1 (3.7-7.0)	2270000 (1650000-3120000)	112.2 (82.0-154.7)	21400 (13900-30700)	1.1 (0.7-1.5)	469000 (305000-674000)	23.2 (15.1-33.4)

Table S20. Deaths and DALYs (in counts and age-specific rates) associated with and attributable to bacterial antimicrobial resistance by pathogen–drug combination, by age group, 2019, sub-Saharan Africa

Pathogen	Antibiotic Class	Neonatal								Post Neonatal								1 to 4								5 and over							
		Associated with resistance				Attributable to resistance				Associated with resistance				Attributable to resistance				Associated with resistance				Attributable to resistance				Associated with resistance				Attributable to resistance			
		Deaths		DALYs		Deaths		DALYs		Deaths		DALYs		Deaths		DALYs		Deaths		DALYs		Deaths		DALYs		Deaths		DALYs		Deaths		DALYs	
		Counts	Rate per 100 000	Counts	Rate per 100 000	Counts	Rate per 100 000	Counts	Rate per 100 000	Counts	Rate per 100 000	Counts	Rate per 100 000	Counts	Rate per 100 000	Counts	Rate per 100 000	Counts	Rate per 100 000	Counts	Rate per 100 000	Counts	Rate per 100 000	Counts	Rate per 100 000	Counts	Rate per 100 000	Counts	Rate per 100 000	Counts	Rate per 100 000	Counts	Rate per 100 000
All pathogens	Resistance to one or more antibiotics	275000 (206000-360000)	9983.9 (7499.9-13081.7)	24400000 (18400000-32000000)	888896.8 (667550.2-1165254.9)	63600 (46600-85400)	2312.3 (1693.5-3104.3)	5660000 (4140000-7590000)	205793.5 (150674.2-276205.9)	160000 (124000-205000)	498.3 (386.8-637.1)	14200000 (11000000-18100000)	44102.9 (34230.9-56403.7)	36500 (27100-48600)	113.6 (84.3-151.1)	3230000 (2400000-4290000)	10052.4 (7460.4-13368.1)	126000 (93100-167000)	96.7 (71.2-127.5)	10900000 (8050000-14400000)	8358.3 (6157.0-11034.0)	28800 (20200-38900)	22.0 (15.4-29.8)	2490000 (1740000-3370000)	1902.2 (1333.1-2576.0)	505000 (390000-651000)	55.3 (42.8-71.3)	16700000 (13000000-21500000)	1830.2 (1428.1-2360.6)	126000 (92600-168000)	13.8 (10.1-18.4)	4110000 (2980000-5530000)	450.9 (326.5-606.1)
Acinetobacter baumannii	Aminoglycosides	3790 (2530-5560)	137.9 (92.1-202.1)	337000 (225000-494000)	12260.8 (8188.1-17969.1)	187 (0-438)	6.8 (0.0-15.9)	16600 (30-39000)	605.2 (1.1-1417.8)	1170 (740-1770)	3.6 (2.3-5.5)	103000 (65500-156000)	321.9 (203.8-486.0)	58 (0-131)	0.2 (0.0-0.4)	5100 (3-11600)	15.9 (0.0-36.2)	913 (526-1470)	0.7 (0.4-1.1)	78600 (45300-127000)	60.1 (34.6-97.0)	45 (0-105)	0.0 (0.0-0.1)	3910 (6-9040)	3.0 (0.0-6.9)	14300 (8500-22200)	1.6 (0.9-2.4)	380000 (225000-594000)	41.7 (24.7-65.1)	691 (0-1700)	0.1 (0.0-0.2)	18500 (104-45500)	2.0 (0.0-5.0)
Acinetobacter baumannii	Anti-pseudomonal penicillin/Beta-Lactamase inhibitors	8440 (5650-12300)	306.9 (205.3-446.9)	751000 (502000-1090000)	27295.2 (18257.9-39744.5)	1120 (625-1830)	40.8 (22.7-66.7)	99900 (55600-163000)	3631.4 (2020.3-5933.3)	2690 (1700-4070)	8.4 (5.3-12.7)	238000 (150000-360000)	739.5 (466.6-1121.3)	363 (190-620)	1.1 (0.6-1.9)	32100 (16800-54800)	99.9 (52.2-170.6)	2040 (1180-3380)	1.6 (0.9-2.6)	176000 (102000-291000)	134.5 (78.0-222.1)	277 (135-496)	0.2 (0.1-0.4)	23800 (11600-42700)	18.2 (8.9-32.7)	32700 (19600-50000)	3.6 (2.1-5.5)	871000 (523000-1350000)	95.5 (57.3-147.4)	4060 (2090-6870)	0.4 (0.2-0.8)	109000 (55800-183000)	12.0 (6.1-20.1)
Acinetobacter baumannii	Beta Lactam/Beta-lactamase inhibitors	5090 (3350-7490)	185.0 (121.7-272.3)	452000 (297000-666000)	16455.5 (10818.6-24212.8)	5 (1-12)	0.2 (0.1-0.5)	482 (126-1100)	17.5 (4.6-40.1)	1650 (1040-2490)	5.1 (3.2-7.8)	146000 (91600-220000)	453.0 (285.2-685.6)	3 (1-6)	0.0 (0.0-0.0)	227 (52-542)	0.7 (0.2-1.7)	1200 (681-1980)	0.9 (0.5-1.5)	103000 (58700-170000)	79.1 (44.9-130.2)	1 (0-3)	0.0 (0.0-0.0)	119 (26-293)	0.1 (0.0-0.2)	20800 (12500-31900)	2.3 (1.4-3.5)	552000 (335000-857000)	60.5 (36.7-93.9)	31 (7-69)	0.0 (0.0-0.0)	848 (205-1880)	0.1 (0.0-0.2)
Acinetobacter baumannii	Carbapenem	3420 (2240-4930)	124.2 (81.4-179.1)	304000 (199000-438000)	11046.7 (7232.2-15927.3)	592 (326-965)	21.5 (11.8-35.1)	52600 (29000-85800)	1914.3 (1053.3-3118.8)	1060 (668-1630)	3.3 (2.1-5.1)	94100 (59100-144000)	293.0 (183.9-449.1)	186 (98-321)	0.6 (0.3-1.0)	16400 (8630-28400)	51.1 (26.9-88.3)	807 (465-1320)	0.6 (0.4-1.0)	69500 (40200-114000)	53.1 (30.7-87.0)	141 (70-260)	0.1 (0.1-0.2)	12200 (6060-22400)	9.3 (4.6-17.1)	14500 (8630-22100)	1.6 (0.9-2.4)	382000 (228000-586000)	41.9 (25.0-64.2)	2480 (1250-4440)	0.3 (0.1-0.5)	65600 (33300-115000)	7.2 (3.7-12.6)
Acinetobacter baumannii	Fluoroquinolones	4630 (3030-6940)	168.5 (110.3-252.4)	412000 (270000-617000)	14984.9 (9804.6-22451.7)	612 (316-1080)	22.2 (11.5-39.2)	54400 (28100-95800)	1977.6 (1023.2-3482.7)	1460 (916-2210)	4.5 (2.9-6.9)	129000 (81000-196000)	401.3 (252.2-608.6)	193 (99-338)	0.6 (0.3-1.1)	17100 (8790-29900)	53.1 (27.4-93.1)	1080 (624-1750)	0.8 (0.5-1.3)	92800 (53700-151000)	70.9 (41.1-115.1)	142 (66-265)	0.1 (0.1-0.2)	12200 (5720-22800)	9.3 (4.4-17.4)	19600 (11700-29900)	2.1 (1.3-3.3)	517000 (309000-794000)	56.6 (33.9-87.0)	2580 (1290-4460)	0.3 (0.1-0.5)	68000 (34100-117000)	7.5 (3.7-12.8)
Acinetobacter baumannii	Fourth-generation cephalosporins	5100 (3320-7480)	185.4 (120.7-272.0)	453000 (295000-665000)	16484.4 (10733.7-24189.9)	5 (1-11)	0.2 (0.0-0.4)	405 (104-942)	14.7 (3.8-34.3)	1560 (988-2350)	4.9 (3.1-7.3)	138000 (87400-208000)	430.7 (272.0-648.0)	2 (0-5)	0.0 (0.0-0.0)	170 (40-408)	0.5 (0.1-1.3)	1200 (699-1940)	0.9 (0.5-1.5)	103000 (60200-167000)	78.8 (46.0-128.0)	1 (0-3)	0.0 (0.0-0.0)	98 (20-256)	0.1 (0.0-0.2)	20100 (11800-31100)	2.2 (1.3-3.4)	532000 (315000-817000)	58.3 (34.5-89.5)	30 (7-75)	0.0 (0.0-0.0)	836 (205-2090)	0.1 (0.0-0.2)
Acinetobacter baumannii	Third-generation cephalosporins	6490 (4300-9530)	236.0 (156.4-346.7)	577000 (382000-848000)	20986.5 (13906.5-30849.2)	65 (26-126)	2.4 (0.9-4.6)	5800 (2270-11200)	211.1 (82.6-407.1)	2100 (1350-3170)	6.5 (4.2-9.9)	186000 (119000-280000)	577.6 (370.7-871.8)	28 (11-55)	0.1 (0.0-0.2)	2480 (1010-4850)	7.7 (3.1-15.1)	1540 (885-2510)	1.2 (0.7-1.9)	132000 (76200-216000)	101.1 (58.3-165.2)	16 (6-33)	0.0 (0.0-0.0)	1390 (502-2870)	1.1 (0.4-2.2)	27000 (16200-41300)	3.0 (1.8-4.5)	716000 (433000-1100000)	78.5 (47.5-120.6)	388 (151-703)	0.0 (0.0-0.1)	10400 (4130-19200)	1.1 (0.5-2.1)
Acinetobacter baumannii	Resistance to one or more antibiotics	8780 (5830-12800)	319.2 (212.0-465.4)	781000 (518000-1140000)	28385.5 (18844.8-41387.3)	2590 (1630-3970)	94.1 (59.2-144.2)	230000 (145000-353000)	8371.8 (5267.0-12823.1)	2830 (1790-4250)	8.8 (5.6-13.2)	250000 (158000-376000)	777.9 (492.1-1170.7)	832 (497-1330)	2.6 (1.5-4.1)	73600 (44000-118000)	228.9 (136.8-366.5)	2130 (1230-3500)	1.6 (0.9-2.7)	183000 (106000-301000)	140.0 (81.0-230.2)	624 (342-1080)	0.5 (0.3-0.8)	53700 (29500-92800)	41.1 (22.5-70.9)	34600 (20800-52800)	3.8 (2.3-5.8)	922000 (555000-1420000)	101.0 (60.8-155.3)	10300 (5890-16000)	1.1 (0.6-1.8)	273000 (157000-430000)	30.0 (17.3-47.1)
Citrobacter spp.	Aminoglycosides	730 (419-1220)	26.6 (15.2-44.3)	65000 (37200-108000)	2362.3 (1354.4-3941.2)	37 (0-96)	1.4 (0.0-3.5)	3330 (0-8500)	121.1 (0.0-309.2)	256 (128-470)	0.8 (0.4-1.5)	22700 (11400-41600)	70.5 (35.4-129.5)	13 (0-36)	0.0 (0.0-0.1)	1150 (0-3190)	3.6 (0.0-9.9)	204 (94-391)	0.2 (0.1-0.3)	17600 (8120-33700)	13.4 (6.2-25.8)	10 (0-29)	0.0 (0.0-0.0)	904 (0-2480)	0.7 (0.0-1.9)	932 (543-1500)	0.1 (0.1-0.2)	28300 (16400-46300)	3.1 (1.8-5.1)	47 (0-120)	0.0 (0.0-0.0)	1430 (0-3690)	0.2 (0.0-0.4)
Citrobacter spp.	Anti-pseudomonal penicillin/Beta-Lactamase inhibitors	947 (544-1540)	34.4 (19.8-56.2)	84200 (48400-137000)	3062.3 (1760.7-4992.7)	153 (73-273)	5.6 (2.6-9.9)	13600 (6460-24200)	495.5 (234.8-881.5)	334 (164-597)	1.0 (0.5-1.9)	29600 (14500-52800)	92.1 (45.2-164.4)	54 (22-106)	0.2 (0.1-0.3)	4780 (1980-9360)	14.9 (6.2-29.1)	254 (117-479)	0.2 (0.1-0.4)	21900 (10100-41200)	16.7 (7.7-31.5)	41 (16-83)	0.0 (0.0-0.1)	3490 (1340-7170)	2.7 (1.0-5.5)	1280 (727-2110)	0.1 (0.1-0.2)	38800 (22000-65400)	4.3 (2.4-7.2)	212 (96-386)	0.0 (0.0-0.0)	6410 (2900-11700)	0.7 (0.3-1.3)
Citrobacter spp.	Carbapenem	413 (236-672)	15.0 (8.6-24.4)	36700 (21000-59800)	1334.9 (763.0-2173.8)	89 (43-162)	3.2 (1.6-5.9)	7870 (3830-14400)	286.3 (139.4-525.3)	144 (72-260)	0.4 (0.2-0.8)	12800 (6350-23000)	39.7 (19.8-71.7)	31 (13-63)	0.1 (0.0-0.2)	2740 (1170-5540)	8.5 (3.6-17.3)	122 (55-230)	0.1 (0.0-0.2)	10500 (4750-19800)	8.0 (3.6-15.1)	26 (10-55)	0.0 (0.0-0.0)	2270 (880-4710)	1.7 (0.7-3.6)	529 (304-863)	0.1 (0.0-0.1)	16100 (9060-26200)	1.8 (1.0-2.9)	115 (55-206)	0.0 (0.0-0.0)	3470 (1650-6260)	0.4 (0.2-0.7)
Citrobacter spp.	Fluoroquinolones	1020 (580-1700)	37.1 (21.1-61.9)	90700 (51600-151000)	3298.9 (1878.0-5506.3)	133 (57-254)	4.8 (2.1-9.2)	11800 (5090-22600)	430.8 (185.2-821.4)	366 (180-661)	1.1 (0.6-2.1)	32400 (15900-58500)	100.8 (49.5-182.2)	48 (19-105)	0.1 (0.1-0.3)	4250 (1680-9250)	13.2 (5.2-28.8)	259 (118-486)	0.2 (0.1-0.4)	22300 (10200-41900)	17.1 (7.8-32.0)	33 (13-74)	0.0 (0.0-0.1)	2860 (1080-6340)	2.2 (0.8-4.8)	1420 (832-2320)	0.2 (0.1-0.3)	42900 (24600-70100)	4.7 (2.7-7.7)	189 (81-363)	0.0 (0.0-0.0)	5690 (2410-11000)	0.6 (0.3-1.2)
Citrobacter spp.	Fourth-generation cephalosporins	1620 (930-2640)	58.9 (33.8-96.1)	144000 (82700-235000)	5237.2 (3008.0-8550.6)	152 (67-281)	5.5 (2.4-10.2)	13500 (5960-25000)	491.6 (216.9-909.2)	567 (279-1030)	1.8 (0.9-3.2)	50200 (24700-91500)	156.3 (77.0-284.9)	53 (20-106)	0.2 (0.1-0.3)	4680 (1750-9410)	14.6 (5.5-29.3)	453 (208-840)	0.3 (0.2-0.6)	39000 (17900-72300)	29.8 (13.7-55.3)	43 (15-89)	0.0 (0.0-0.1)	3710 (1330-7650)	2.8 (1.0-5.8)	2110 (1200-3420)	0.2 (0.1-0.4)	64000 (36000-106000)	7.0 (3.9-11.6)	197 (85-379)	0.0 (0.0-0.0)	5990 (2570-11500)	0.7 (0.3-1.3)
Citrobacter spp.	Third-generation cephalosporins	1890 (1100-3100)	68.6 (40.0-112.8)	168000 (98100-276000)	6098.3 (3567.2-10027.8)	77 (29-146)	2.8 (1.1-5.3)	6890 (2570-12900)	250.4 (93.4-470.4)	663 (334-1190)	2.1 (1.0-3.7)	58700 (29500-106000)	182.7 (91.9-328.7)	27 (10-57)	0.1 (0.0-0.2)	2420 (893-5080)	7.5 (2.8-15.8)	513 (236-964)	0.4 (0.2-0.7)	44200 (20300-83000)	33.8 (15.5-63.5)	20 (7-41)	0.0 (0.0-0.0)	1680 (571-3500)	1.3 (0.4-2.7)	2530 (1440-4190)	0.3 (0.2-0.5)	76600 (43400-127000)	8.4 (4.8-14.0)	114 (46-222)	0.0 (0.0-0.0)	3390 (1380-6650)	0.4 (0.2-0.7)
Citrobacter spp.	Resistance to one or more antibiotics	2160 (1270-3560)	78.6 (46.2-129.5)	192000 (113000-317000)	6992.9 (4115.9-11514.7)	642 (340-1110)	23.3 (12.4-40.5)	57100 (30200-98900)	2075.7 (1099.5-3597.8)	762 (385-1380)	2.4 (1.2-4.3)	67500 (34100-122000)	210.0 (106.2-378.9)	226 (105-440)	0.7 (0.3-1.4)	20000 (9340-38900)	62.3 (29.1-121.0)	581 (271-1080)	0.4 (0.2-0.8)	50000 (23300-92600)	38.3 (17.8-70.8)	173 (74-347)	0.1 (0.1-0.3)	14900 (6350-29900)	11.4 (4.9-22.8)	2950 (1710-4830)	0.3 (0.2-0.5)	89200 (51000-148000)	9.8 (5.6-16.2)	873 (460-1540)	0.1 (0.1-0.2)	26400 (13800-46800)	2.9 (1.5-5.1)
Enterobacter spp.	Aminoglycosides	6310 (4430-8840)	229.7 (160.9-321.6)	562000 (394000-787000)	20439.6 (14324.5-28621.3)	338 (95-656)	12.3 (3.4-23.9)	30100 (8520-58400)	1094.9 (309.8-2125.5)	1430 (960-2050)	4.5 (3.0-6.4)	127000 (85000-181000)	395.1 (264.6-564.2)	77 (22-146)	0.2 (0.1-0.5)	6780 (1940-13000)	21.1 (6.0-40.3)	1150 (721-1730)	0.9 (0.6-1.3)	99000 (62100-149000)	75.7 (47.5-113.9)	61 (16-121)	0.0 (0.0-0.1)	5290 (1410-10400)	4.0 (1.1-8.0)	8250 (5470-12100)	0.9 (0.6-1.3)	242000 (157000-360000)	26.5 (17.2-39.5)	440 (122-844)	0.0 (0.0-0.1)	13000 (3690-25200)	1.4 (0.4-2.8)
Enterobacter spp.	Anti-pseudomonal penicillin/Beta-Lactamase inhibitors	5000 (3530-7010)	181.8 (128.4-255.1)	445000 (314000-624000)	16174.0 (11423.7-22703.9)	402 (224-665)	14.6 (8.2-24.2)	35700 (19900-59200)	1299.9 (725.2-2151.5)	1140 (762-1620)	3.5 (2.4-5.1)	101000 (67400-144000)	313.9 (209.8-447.6)	91 (50-153)	0.3 (0.2-0.5)	8080 (4390-13600)	25.2 (13.7-42.3)	901 (553-1340)	0.7 (0.4-1.0)	77600 (47700-116000)	59.4 (36.4-88.5)	71 (37-121)	0.1 (0.0-0.1)	6150 (3200-10500)	4.7 (2.4-8.0)	6860 (4570-9950)	0.8 (0.5-1.1)	201000 (131000-300000)	22.0 (14.3-32.8)	559 (303-959)	0.1 (0.0-0.1)	16400 (8700-28300)	1.8 (1.0-3.1)
Enterobacter spp.	Carbapenem	2040 (1420-2890)	74.3 (51.8-105.3)	182000 (127000-258000)	6607.7 (4605.2-9365.9)	406 (237-652)	14.8 (8.6-23.7)	36100 (21100-58100)	1313.2 (767.7-2114.0)	469 (313-690)	1.5 (1.0-2.1)	41500 (27700-61000)	129.2 (86.1-189.9)	93 (54-152)	0.3 (0.2-0.5)	8230 (4750-13400)	25.6 (14.8-41.8)	373 (234-568)	0.3 (0.2-0.4)	32100 (20100-48900)	24.5 (15.4-37.4)	73 (40-122)	0.1 (0.0-0.1)	6300 (3440-10500)	4.8 (2.6-8.0)	2920 (1920-4250)	0.3 (0.2-0.5)	85800 (55200-126000)	9.4 (6.0-13.8)	595 (337-956)	0.1 (0.0-0.1)	17400 (9640-28300)	1.9 (1.1-3.1)
Enterobacter spp.	Fluoroquinolones	7450 (5260-10600)	271.0 (191.3-386.9)	663000 (468000-947000)	24109.1 (17019.2-34443.0)	691 (359-1190)	25.1 (13.0-43.3)	61400 (31900-106000)	2234.1 (1160.2-3851.1)	1710 (1140-2520)	5.3 (3.5-7.8)	152000 (101000-223000)	471.8 (313.5-692.7)	159 (80-281)	0.5 (0.2-0.9)	14100 (7090-24800)	43.8 (22.1-77.2)	1360 (854-2070)	1.0 (0.7-1.6)	117000 (73600-178000)	89.7 (56.3-136.4)	126 (61-228)	0.1 (0.0-0.2)	10900 (5280-19600)	8.3 (4.0-15.0)	10100 (6730-14700)	1.1 (0.7-1.6)	297000 (194000-436000)	32.6 (21.2-47.7)	952 (500-1620)	0.1 (0.1-0.2)	27900 (14600-48800)	3.1 (1.6-5.4)
Enterobacter spp.	Fourth-generation cephalosporins	10600 (7580-15000)	387.3 (275.6-545.1)	948000 (674000-1330000)	34460.2 (24513.4-48501.5)	729 (363-1280)	26.5 (13.2-46.5)	64900 (32400-114000)	2359.7 (1177.4-4141.0)	2440 (1630-3480)	7.6 (5.1-10.8)	216000 (145000-308000)	672.4 (450.6-959.0)	167 (81-287)	0.5 (0.3-0.9)	14800 (7170-25400)	46.1 (22.3-79.1)	1920 (1210-2870)	1.5 (0.9-2.2)	165000 (104000-247000)	126.5 (79.7-189.2)	130 (62-228)	0.1 (0.0-0.2)	11200 (5340-19600)	8.6 (4.1-15.0)	14600 (9650-21000)	1.6 (1.1-2.3)	428000 (281000-624000)	46.9 (30.8-68.4)	1010 (488-1720)	0.1 (0.1-0.2)	29700 (14400-51200)	3.3 (1.6-5.6)
Enterobacter spp.	Trimethoprim-Sulfamethoxazole	10100 (7190-14100)	367.3 (261.5-511.0)	899000 (640000-1250000)	32687.0 (23260.9-45452.6)	462 (0-943)	16.8 (0.0-34.3)	41100 (0-83800)	1493.9 (0.0-3047.9)	2300 (1560-3310)	7.2 (4.9-10.3)	204000 (139000-293000)	634.4 (431.2-912.9)	105 (0-221)	0.3 (0.0-0.7)	9280 (0-19600)	28.9 (0.0-60.9)	1810 (1150-2710)	1.4 (0.9-2.1)	156000 (99100-234000)	119.2 (75.8-178.7)	82 (0-178)	0.1 (0.0-0.1)	7030 (0-15300)	5.4 (0.0-11.7)	14300 (9720-20600)	1.6 (1.1-2.3)	417000 (277000-602000)	45.7 (30.3-66.0)	690 (0-1460)	0.1 (0.0-0.2)	20000 (0-42900)	2.2 (0.0-4.7)
Enterobacter spp.	Resistance to one or more antibiotics	12700 (9070-17700)	462.4 (330.0-643.7)	1130000 (807000-1570000)	41149.6 (29362.0-57260.7)	3030 (2040-4420)	110.1 (74.0-160.6)	269000 (181000-393000)	9795.7 (6584.2-14289.2)	2900 (1970-4150)	9.0 (6.1-12.9)	257000 (175000-368000)	799.5 (543.3-1143.9)	692 (449-1020)	2.2 (1.4-3.2)	61300 (39700-90600)	190.7 (123.6-282.1)	2270 (1440-3380)	1.7 (1.1-2.6)	196000 (124000-291000)	149.5 (94.7-222.8)	544 (336-841)	0.4 (0.3-0.6)	46800 (29000-72400)	35.8 (22.1-55.4)	18100 (12200-26000)	2.0 (1.3-2.9)	529000 (352000-764000)	58.0 (38.6-83.8)	4240 (2690-6360)	0.5 (0.3-0.7)	124000 (76100-191000)	13.6 (8.3-21.0)
Enterococcus faecalis	Fluoroquinolones	5700 (3910-8180)	207.2 (142.3-297.3)	507000 (348000-727000)	18425.8 (12654.9-26439.3)	1420 (685-2410)	51.8 (24.9-87.6)	127000 (61000-214000)	4601.3 (2216.7-7782.5)	529 (311-821)	1.6 (1.0-2.6)	46900 (27600-73000)	146.1 (85.8-227.3)	132 (61-226)	0.4 (0.2-0.7)	11700 (5410-20000)	36.4 (16.8-62.3)	451 (237-760)	0.3 (0.2-0.6)	38900 (20400-65600)	29.8 (15.6-50.1)	113 (46-209)	0.1 (0.0-0.2)	9700 (3970-18000)	7.4 (3.0-13.8)	10900 (6270-17000)	1.2 (0.7-1.9)	351000 (204000-559000)	38.4 (22.3-61.2)	2730 (1190-4960)	0.3 (0.1-0.5)	87200 (38500-155000)	9.6 (4.2-17.0)
Enterococcus faecalis	Vancomycin	370 (237-553)	13.5 (8.6-20.1)	32900 (21100-49100)	1197.1 (766.2-1787.0)	101 (43-190)	3.7 (1.6-6.9)	8950 (3830-16900)	325.6 (139.4-614.1)	34 (20-56)	0.1 (0.1-0.2)	3040 (1790-4940)	9.5 (5.6-15.4)	9 (4-19)	0.0 (0.0-0.1)	822 (340-1720)	2.6 (1.1-5.4)	29 (15-52)	0.0 (0.0-0.0)	2470 (1270-4520)	1.9 (1.0-3.5)	8 (3-18)	0.0 (0.0-0.0)	670 (254-1540)	0.5 (0.2-1.2)	721 (410-1170)	0.1 (0.0-0.1)	23100 (13300-38200)	2.5 (1.5-4.2)	198 (82-394)	0.0 (0.0-0.0)	6330 (2590-12800)	0.7 (0.3-1.4)
Enterococcus faecalis	Resistance to one or more antibiotics	5780 (3980-8310)	210.1 (144.6-302.4)	514000 (354000-739000)	18691.9 (12856.5-26885.9)	1520 (787-2530)	55.4 (28.6-92.2)	135000 (70000-225000)	4926.9 (2546.0-8200.2)	536 (316-835)	1.7 (1.0-2.6)	47600 (28100-74100)	148.1 (87.3-230.7)	141 (69-239)	0.4 (0.2-0.7)	12500 (6120-21200)	38.9 (19.0-65.8)	457 (240-768)	0.3 (0.2-0.6)	39500 (20700-66300)	30.2 (15.9-50.7)	120 (53-220)	0.1 (0.0-0.2)	10400 (4570-18900)	7.9 (3.5-14.5)	11100 (6370-17200)	1.2 (0.7-1.9)	356000 (207000-568000)	39.0 (22.7-62.3)	2930 (1340-5210)	0.3 (0.1-0.6)	93600 (43000-166000)	10.3 (4.7-18.1)
Enterococcus faecium	Fluoroquinolones	3240 (2190-4700)	117.9 (79.5-171.0)	288000 (195000-418000)	10491.6 (7073.6-15204.9)	627 (180-1160)	22.8 (6.5-42.3)	55800 (16000-103000)	2028.6 (582.9-3763.6)	620 (352-1030)	1.9 (1.1-3.2)	55000 (31200-90900)	171.1 (97.2-282.8)	120 (34-257)	0.4 (0.1-0.8)	10600 (3010-22700)	33.1 (9.4-70.7)	561 (284-1050)	0.4 (0.2-0.8)	48300 (24500-90200)	36.9 (18.7-69.0)	109 (30-256)	0.1 (0.0-0.2)	9360 (2550-22000)	7.2 (1.9-16.8)	14500 (8310-24200)	1.6 (0.9-2.6)	436000 (243000-743000)	47.8 (26.7-81.4)	2830 (780-5760)	0.3 (0.1-0.6)	84800 (23600-177000)	9.3 (2.6-19.4)
Enterococcus faecium	Vancomycin	801 (518-1180)	29.1 (18.8-42.9)	71300 (46100-105000)	2592.1 (1677.8-3817.1)	189 (91-347)	6.9 (3.3-12.6)	16900 (8070-30900)	613.2 (293.5-1122.4)	154 (86-263)	0.5 (0.3-0.8)	13700 (7620-23400)	42.5 (23.7-72.7)	36 (16-73)	0.1 (0.1-0.2)	3230 (1440-6440)	10.1 (4.5-20.0)	140 (68-269)	0.1 (0.1-0.2)	12000 (5870-23200)	9.2 (4.5-17.7)	33 (12-73)	0.0 (0.0-0.1)	2840 (1080-6310)	2.2 (0.8-4.8)	3460 (1980-5920)	0.4 (0.2-0.6)	104000 (57900-183000)	11.4 (6.3-20.1)	820 (356-1600)	0.1 (0.0-0.2)	24700 (10500-48400)	2.7 (1.1-5.3)
Enterococcus faecium	Resistance to one or more antibiotics	3270 (2200-4770)	118.7 (80.0-173.3)	291000 (196000-424000)	10567.3 (7120.6-15409.9)	816 (431-1370)	29.7 (15.7-49.8)	72600 (38400-122000)	2641.8 (1395.9-4426.0)	625 (356-1030)	1.9 (1.1-3.2)	55400 (31500-91600)	172.3 (98.1-285.2)	157 (71-303)	0.5 (0.2-0.9)	13900 (6260-26800)	43.2 (19.5-83.4)	565 (287-1050)	0.4 (0.2-0.8)	48600 (24700-90700)	37.2 (18.9-69.3)	142 (57-302)	0.1 (0.0-0.2)	12200 (4870-26000)	9.3 (3.7-19.9)	14600 (8370-24300)	1.6 (0.9-2.7)	439000 (245000-746000)	48.1 (26.8-81.7)	3650 (1640-6890)	0.4 (0.2-0.8)	110000 (49700-207000)	12.0 (5.4-22.7)
Other enterococci	Fluoroquinolones	3500 (2120-5490)	127.2 (77.0-199.8)	311000 (188000-488000)	11308.9 (6847.4-17755.4)	683 (93-1400)	24.8 (3.4-51.0)	60700 (8280-124000)	2205.9 (301.2-4527.8)	440 (230-798)	1.4 (0.7-2.5)	38900 (20400-70700)	121.2 (63.5-220.0)	86 (13-189)	0.3 (0.0-0.6)	7620 (1150-16700)	23.7 (3.6-52.0)	316 (152-595)	0.2 (0.1-0.5)	27400 (13300-51600)	21.0 (10.2-39.5)	62 (8-143)	0.0 (0.0-0.1)	5340 (730-12400)	4.1 (0.6-9.4)	4300 (2580-7150)	0.5 (0.3-0.8)	114000 (69900-180000)	12.5 (7.7-19.8)	840 (133-1800)	0.1 (0.0-0.2)	22000 (3720-46000)	2.4 (0.4-5.0)
Other enterococci	Vancomycin	196 (117-317)	7.1 (4.2-11.5)	17400 (10400-28200)	632.8 (376.9-1024.4)	39 (19-75)	1.4 (0.7-2.7)	3440 (1670-6690)	125.0 (60.7-243.2)	25 (13-44)	0.1 (0.0-0.1)	2180 (1110-3890)	6.8 (3.5-12.1)	5 (2-10)	0.0 (0.0-0.0)	432 (181-910)	1.3 (0.6-2.8)	16 (8-31)	0.0 (0.0-0.0)	1430 (687-2700)	1.1 (0.5-2.1)	3 (1-7)	0.0 (0.0-0.0)	286 (113-646)	0.2 (0.1-0.5)	264 (156-434)	0.0 (0.0-0.0)	6960 (4210-11300)	0.8 (0.5-1.2)	51 (24-101)	0.0 (0.0-0.0)	1380 (680-2590)	0.2 (0.1-0.3)
Other enterococci	Resistance to one or more antibiotics	3510 (2120-5510)	127.6 (77.2-200.4)	312000 (189000-490000)	11337.7 (6864.9-17808.4)	721 (140-1450)	26.2 (5.1-52.7)	64100 (12500-129000)	2330.9 (453.8-4679.6)	441 (231-800)	1.4 (0.7-2.5)	39000 (20500-70900)	121.5 (63.7-220.5)	91 (19-195)	0.3 (0.1-0.6)	8060 (1720-17300)	25.1 (5.4-53.8)	317 (153-596)	0.2 (0.1-0.5)	27500 (13300-51800)	21.0 (10.2-39.6)	65 (12-148)	0.0 (0.0-0.1)	5620 (1060-12800)	4.3 (0.8-9.8)	4320 (2580-7190)	0.5 (0.3-0.8)	114000 (70000-181000)	12.5 (7.7-19.8)	891 (199-1870)	0.1 (0.0-0.2)	23400 (5610-48000)	2.6 (0.6-5.3)
Escherichia coli	Aminoglycosides	14000 (9930-19100)	510.7 (361.1-696.3)	1250000 (885000-1710000)	45511.0 (32195.5-62039.6)	812 (516-1170)	29.5 (18.8-42.7)	72400 (45900-105000)	2631.3 (1670.7-3804.8)	7070 (4940-9850)	22.0 (15.4-30.6)	627000 (438000-873000)	1952.7 (1362.7-2717.5)	408 (266-601)	1.3 (0.8-1.9)	36200 (23600-53300)	112.7 (73.4-165.8)	5890 (3890-8590)	4.5 (3.0-6.6)	512000 (338000-746000)	391.2 (258.1-570.1)	339 (210-511)	0.3 (0.2-0.4)	29400 (18300-44300)	22.5 (14.0-33.9)	22500 (16500-30100)	2.5 (1.8-3.3)	688000 (499000-936000)	75.4 (54.7-102.6)	1310 (863-1840)	0.1 (0.1-0.2)	40200 (26200-56600)	4.4 (2.9-6.2)
Escherichia coli	Aminopenicillin	37700 (26900-51900)	1372.2 (979.6-1887.7)	3360000 (2400000-4630000)	122276.7 (87422.0-168222.3)	438 (289-639)	15.9 (10.5-23.3)	39000 (25800-56900)	1418.6 (937.4-2068.9)	18600 (13300-26000)	57.9 (41.5-81.0)	1650000 (1180000-2310000)	5137.7 (3688.2-7193.4)	214 (142-309)	0.7 (0.4-1.0)	19000 (12600-27500)	59.1 (39.2-85.7)	15200 (10200-22300)	11.6 (7.8-17.0)	1320000 (882000-1940000)	1012.5 (674.1-1486.4)	166 (103-250)	0.1 (0.1-0.2)	14500 (9040-21900)	11.1 (6.9-16.8)	66900 (49700-88200)	7.3 (5.4-9.7)	2030000 (1510000-2690000)	222.4 (165.3-295.1)	944 (641-1330)	0.1 (0.1-0.1)	28200 (19100-39500)	3.1 (2.1-4.3)
Escherichia coli	Beta Lactam/Beta-lactamase inhibitors	30100 (21300-41400)	1093.1 (776.4-1506.5)	2680000 (1900000-3690000)	97410.6 (69150.1-134201.3)	992 (695-1430)	36.1 (25.3-51.9)	88400 (62000-127000)	3215.7 (2253.3-4626.5)	14800 (10700-20800)	46.1 (33.3-64.8)	1310000 (947000-1850000)	4090.4 (2948.5-[illegible])	481 (323-704)	1.5 (1.0-2.2)	42700 (28600-62500)	133.0 (89.1-194.5)	12200 (8180-18000)	9.3 (6.3-13.8)	1060000 (710000-[illegible])	811.4 (542.8-1192.1)	385 (249-591)	0.3 (0.2-0.5)	33600 (21800-51500)	25.7 (16.6-39.4)	51500 (38500-68300)	5.6 (4.2-7.5)	1570000 (1160000-[illegible])	171.9 (127.0-231.1)	1850 (1310-2590)	0.2 (0.1-0.3)	56500 (39200-79300)	6.2 (4.3-8.7)
Escherichia coli	Carbapenem	4450 (2980-6420)	161.8 (108.5-233.5)	396000 (266000-572000)	14411.0 (9661.2-20807.8)	893 (488-1470)	32.5 (17.7-53.5)	79500 (43400-131000)	2892.3 (1579.6-4762.7)	2210 (1460-3240)	6.9 (4.5-10.1)	196000 ([illegible]-288000)	[illegible]	[illegible]	[illegible]	[illegible]	[illegible]	[illegible]	[illegible]	[illegible]	[illegible]	[illegible]	[illegible]	[illegible]	[illegible]	[illegible]	[illegible]	[illegible]	[illegible]	[illegible]	[illegible]	[illegible]	[illegible]
Escherichia coli	Fluoroquinolones	23100 (16400-31300)	840.7 (597.2-1139.3)	2060000 (1460000-2800000)	74922.0 (53193.5-101652.2)	2170 (1450-3060)	79.0 (52.8-111.4)	194000 (129000-273000)	7041.0 (4699.2-9931.3)	11400 (8170-16100)	35.6 (25.4-50.0)	1010000 ([illegible]-1430000)	[illegible]	[illegible]	[illegible]	[illegible]	[illegible]	[illegible]	[illegible]	[illegible]	[illegible]	[illegible]	[illegible]	[illegible]	[illegible]	[illegible]	[illegible]	[illegible]	[illegible]	[illegible]	[illegible]	[illegible]	[illegible]
Escherichia coli	Third-generation cephalosporins	23000 (16300-31900)	837.5 (594.3-1159.2)	2050000 (1460000-2840000)	74640.6 (53004.4-103322.3)	2790 (1200-5130)	101.4 (43.6-186.4)	248000 (108000-457000)	9035.9 (3918.5-16612.9)	11400 (8030-16100)	35.6 (25.0-50.0)	1010000 ([illegible]-1430000)	[illegible]	[illegible]	[illegible]	[illegible]	[illegible]	[illegible]	[illegible]	[illegible]	[illegible]	[illegible]	[illegible]	[illegible]	[illegible]	[illegible]	[illegible]	[illegible]	[illegible]	[illegible]	[illegible]	[illegible]	[illegible]
Escherichia coli	Trimethoprim-Sulfamethoxazole	36200 (25900-49600)	1316.1 (942.8-1805.3)	3220000 (2310000-4430000)	117273.6 (84024.6-160963.0)	2140 (1390-3090)	77.8 (50.6-112.3)	190000 (124000-274000)	6922.5 (4499.5-9978.3)	17700 (12700-24900)	55.2 (39.7-77.6)	1580000 ([illegible]-2210000)	[illegible]	[illegible]	[illegible]	[illegible]	[illegible]	[illegible]	[illegible]	[illegible]	[illegible]	[illegible]	[illegible]	[illegible]	[illegible]	[illegible]	[illegible]	[illegible]	[illegible]	[illegible]	[illegible]	[illegible]	[illegible]
Escherichia coli	Resistance to one or more antibiotics	40400 (28900-55500)	1469.6 (1052.0-2017.3)	3600000 (2580000-4940000)	130953.2 (93749.8-179750.6)	10200 (6840-14800)	372.3 (248.6-536.6)	912000 (609000-1310000)	33157.3 (22141.0-47803.7)	19800 (14200-27800)	61.6 (44.3-86.5)	1760000 ([illegible]-2460000)	[illegible]	[illegible]	[illegible]	[illegible]	[illegible]	[illegible]	[illegible]	[illegible]	[illegible]	[illegible]	[illegible]	[illegible]	[illegible]	[illegible]	[illegible]	[illegible]	[illegible]	[illegible]	[illegible]	[illegible]	[illegible]
Group A *Streptococcus*	Macrolide	1100 (708-1700)	40.1 (25.8-61.9)	98100 (63000-151000)	3567.9 (2290.4-5503.5)	105 (0-332)	3.8 (0.0-12.1)	9290 (0-29500)	337.8 (0.0-1074.6)	458 (274-722)	1.4 (0.9-2.2)	40700 (24400-[illegible])	[illegible]	[illegible]	[illegible]	[illegible]	[illegible]	[illegible]	[illegible]	[illegible]	[illegible]	[illegible]	[illegible]	[illegible]	[illegible]	[illegible]	[illegible]	[illegible]	[illegible]	[illegible]	[illegible]	[illegible]	[illegible]
Group A *Streptococcus*	Resistance to one or more antibiotics	1100 (708-1700)	40.1 (25.8-61.9)	98100 (63000-151000)	3567.9 (2290.4-5503.5)	105 (0-332)	3.8 (0.0-12.1)	9290 (0-29500)	337.8 (0.0-1074.6)	458 (274-722)	1.4 (0.9-2.2)	40700 (24400-[illegible])	[illegible]	[illegible]	[illegible]	[illegible]	[illegible]	[illegible]	[illegible]	[illegible]	[illegible]	[illegible]	[illegible]	[illegible]	[illegible]	[illegible]	[illegible]	[illegible]	[illegible]	[illegible]	[illegible]	[illegible]	[illegible]
Group B *Streptococcus*	Fluoroquinolones	18300 (12600-26200)	665.9 (457.3-953.3)	1630000 (1120000-2330000)	59319.3 (40718.6-84897.5)	3190 (527-6980)	115.9 (19.2-253.9)	283000 (47100-621000)	10306.9 (1711.9-22584.6)	3650 (2590-4980)	11.3 (8.1-15.5)	323000 ([illegible]-440000)	[illegible]	[illegible]	[illegible]	[illegible]	[illegible]	[illegible]	[illegible]	[illegible]	[illegible]	[illegible]	[illegible]	[illegible]	[illegible]	[illegible]	[illegible]	[illegible]	[illegible]	[illegible]	[illegible]	[illegible]	[illegible]
Group B *Streptococcus*	Macrolide	22300 (15400-31400)	811.1 (560.1-1141.4)	[illegible]	[illegible]	[illegible]	[illegible]	[illegible]	[illegible]	[illegible]	[illegible]	[illegible]	[illegible]	[illegible]	[illegible]	[illegible]	[illegible]	[illegible]	[illegible]	[illegible]	[illegible]	[illegible]	[illegible]	[illegible]	[illegible]	[illegible]	[illegible]	[illegible]	[illegible]	[illegible]	[illegible]	[illegible]	[illegible]
Group B *Streptococcus*	Penicillin	748 (503-1090)	27.2 (18.3-39.5)	[illegible]	[illegible]	[illegible]	[illegible]	[illegible]	[illegible]	[illegible]	[illegible]	[illegible]	[illegible]	[illegible]	[illegible]	[illegible]	[illegible]	[illegible]	[illegible]	[illegible]	[illegible]	[illegible]	[illegible]	[illegible]	[illegible]	[illegible]	[illegible]	[illegible]	[illegible]	[illegible]	[illegible]	[illegible]	[illegible]
Group B *Streptococcus*	Resistance to one or more antibiotics	31500 (22500-43800)	1146.4 (817.4-1594.3)	[illegible]	[illegible]	[illegible]	[illegible]	[illegible]	[illegible]	[illegible]	[illegible]	[illegible]	[illegible]	[illegible]	[illegible]	[illegible]	[illegible]	[illegible]	[illegible]	[illegible]	[illegible]	[illegible]	[illegible]	[illegible]	[illegible]	[illegible]	[illegible]	[illegible]	[illegible]	[illegible]	[illegible]	[illegible]	[illegible]
Haemophilus influenzae	Aminopenicillin	3030 (2040-4340)	110.1 (74.0-157.8)	[illegible]	[illegible]	[illegible]	[illegible]	[illegible]	[illegible]	[illegible]	[illegible]	[illegible]	[illegible]	[illegible]	[illegible]	[illegible]	[illegible]	[illegible]	[illegible]	[illegible]	[illegible]	[illegible]	[illegible]	[illegible]	[illegible]	[illegible]	[illegible]	[illegible]	[illegible]	[illegible]	[illegible]	[illegible]	[illegible]
Haemophilus influenzae	Third-generation cephalosporins	1140 (753-1660)	41.5 (27.4-60.5)	[illegible]	[illegible]	[illegible]	[illegible]	[illegible]	[illegible]	[illegible]	[illegible]	[illegible]	[illegible]	[illegible]	[illegible]	[illegible]	[illegible]	[illegible]	[illegible]	[illegible]	[illegible]	[illegible]	[illegible]	[illegible]	[illegible]	[illegible]	[illegible]	[illegible]	[illegible]	[illegible]	[illegible]	[illegible]	[illegible]
Haemophilus influenzae	Resistance to one or more antibiotics	3520 (2390-5020)	128.0 (86.9-182.6)	[illegible]	[illegible]	[illegible]	[illegible]	[illegible]	[illegible]	[illegible]	[illegible]	[illegible]	[illegible]	[illegible]	[illegible]	[illegible]	[illegible]	[illegible]	[illegible]	[illegible]	[illegible]	[illegible]	[illegible]	[illegible]	[illegible]	[illegible]	[illegible]	[illegible]	[illegible]	[illegible]	[illegible]	[illegible]	[illegible]
Klebsiella pneumoniae	Aminoglycosides	42000 (30500-55800)	1528.7 (1107.5-2029.1)	[illegible]	[illegible]	[illegible]	[illegible]	[illegible]	[illegible]	[illegible]	[illegible]	[illegible]	[illegible]	[illegible]	[illegible]	[illegible]	[illegible]	[illegible]	[illegible]	[illegible]	[illegible]	[illegible]	[illegible]	[illegible]	[illegible]	[illegible]	[illegible]	[illegible]	[illegible]	[illegible]	[illegible]	[illegible]	[illegible]
Klebsiella pneumoniae	Beta Lactam/Beta-lactamase inhibitors	54900 (39900-72600)	1996.9 (1451.8-2641.5)	[illegible]	[illegible]	[illegible]	[illegible]	[illegible]	[illegible]	[illegible]	[illegible]	[illegible]	[illegible]	[illegible]	[illegible]	[illegible]	[illegible]	[illegible]	[illegible]	[illegible]	[illegible]	[illegible]	[illegible]	[illegible]	[illegible]	[illegible]	[illegible]	[illegible]	[illegible]	[illegible]	[illegible]	[illegible]	[illegible]
Klebsiella pneumoniae	Carbapenem	6830 (3460-13100)	248.5 (125.7-475.6)	[illegible]	[illegible]	[illegible]	[illegible]	[illegible]	[illegible]	[illegible]	[illegible]	[illegible]	[illegible]	[illegible]	[illegible]	[illegible]	[illegible]	[illegible]	[illegible]	[illegible]	[illegible]	[illegible]	[illegible]	[illegible]	[illegible]	[illegible]	[illegible]	[illegible]	[illegible]	[illegible]	[illegible]	[illegible]	[illegible]
Klebsiella pneumoniae	Fluoroquinolones	41500 (30200-55200)	1508.4 (1098.9-2008.0)	[illegible]	[illegible]	[illegible]	[illegible]	[illegible]	[illegible]	[illegible]	[illegible]	[illegible]	[illegible]	[illegible]	[illegible]	[illegible]	[illegible]	[illegible]	[illegible]	[illegible]	[illegible]	[illegible]	[illegible]	[illegible]	[illegible]	[illegible]	[illegible]	[illegible]	[illegible]	[illegible]	[illegible]	[illegible]	[illegible]
Klebsiella pneumoniae	Third-generation cephalosporins	49800 (36500-67000)	1812.7 (1327.3-2435.5)	[illegible]	[illegible]	[illegible]	[illegible]	[illegible]	[illegible]	[illegible]	[illegible]	[illegible]	[illegible]	[illegible]	[illegible]	[illegible]	[illegible]	[illegible]	[illegible]	[illegible]	[illegible]	[illegible]	[illegible]	[illegible]	[illegible]	[illegible]	[illegible]	[illegible]	[illegible]	[illegible]	[illegible]	[illegible]	[illegible]
Klebsiella pneumoniae	Trimethoprim-Sulfamethoxazole	54800 (40000-72800)	1994.4 (1454.9-2646.2)	[illegible]	[illegible]	[illegible]	[illegible]	[illegible]	[illegible]	[illegible]	[illegible]	[illegible]	[illegible]	[illegible]	[illegible]	[illegible]	[illegible]	[illegible]	[illegible]	[illegible]	[illegible]	[illegible]	[illegible]	[illegible]	[illegible]	[illegible]	[illegible]	[illegible]	[illegible]	[illegible]	[illegible]	[illegible]	[illegible]
Klebsiella pneumoniae	Resistance to one or more antibiotics	59200 (43100-78300)	2153.4 (1567.0-2848.1)	[illegible]	[illegible]	[illegible]	[illegible]	[illegible]	[illegible]	[illegible]	[illegible]	[illegible]	[illegible]	[illegible]	[illegible]	[illegible]	[illegible]	[illegible]	[illegible]	[illegible]	[illegible]	[illegible]	[illegible]	[illegible]	[illegible]	[illegible]	[illegible]	[illegible]	[illegible]	[illegible]	[illegible]	[illegible]	[illegible]
Morganella spp.	Fluoroquinolones	1 (0-3)	0.0 (0.0-0.1)	[illegible]	[illegible]	[illegible]	[illegible]	[illegible]	[illegible]	[illegible]	[illegible]	[illegible]	[illegible]	[illegible]	[illegible]	[illegible]	[illegible]	[illegible]	[illegible]	[illegible]	[illegible]	[illegible]	[illegible]	[illegible]	[illegible]	[illegible]	[illegible]	[illegible]	[illegible]	[illegible]	[illegible]	[illegible]	[illegible]
Morganella spp.	Fourth-generation cephalosporins	1 (0-2)	0.0 (0.0-0.1)	[illegible]	[illegible]	[illegible]	[illegible]	[illegible]	[illegible]	[illegible]	[illegible]	[illegible]	[illegible]	[illegible]	[illegible]	[illegible]	[illegible]	[illegible]	[illegible]	[illegible]	[illegible]	[illegible]	[illegible]	[illegible]	[illegible]	[illegible]	[illegible]	[illegible]	[illegible]	[illegible]	[illegible]	[illegible]	[illegible]
Morganella spp.	Third-generation cephalosporins	0 (0-1)	0.0 (0.0-0.0)	[illegible]	[illegible]	[illegible]	[illegible]	[illegible]	[illegible]	[illegible]	[illegible]	[illegible]	[illegible]	[illegible]	[illegible]	[illegible]	[illegible]	[illegible]	[illegible]	[illegible]	[illegible]	[illegible]	[illegible]	[illegible]	[illegible]	[illegible]	[illegible]	[illegible]	[illegible]	[illegible]	[illegible]	[illegible]	[illegible]
Morganella spp.	Resistance to one or more antibiotics	2 (1-4)	0.1 (0.0-0.2)	[illegible]	[illegible]	[illegible]	[illegible]	[illegible]	[illegible]	[illegible]	[illegible]	[illegible]	[illegible]	[illegible]	[illegible]	[illegible]	[illegible]	[illegible]	[illegible]	[illegible]	[illegible]	[illegible]	[illegible]	[illegible]	[illegible]	[illegible]	[illegible]	[illegible]	[illegible]	[illegible]	[illegible]	[illegible]	[illegible]
Mycobacterium tuberculosis	Extensive drug resistance in TB	–	–	0 (0-0)	[illegible]	[illegible]	[illegible]	[illegible]	[illegible]	[illegible]	[illegible]	[illegible]	[illegible]	[illegible]	[illegible]	[illegible]	[illegible]	[illegible]	[illegible]	[illegible]	[illegible]	[illegible]	[illegible]	[illegible]	[illegible]	[illegible]	[illegible]	[illegible]	[illegible]	[illegible]	[illegible]	[illegible]	[illegible]
Mycobacterium tuberculosis	Isoniazid mono-resistance	–	–	1 (0-1)	[illegible]	[illegible]	[illegible]	[illegible]	[illegible]	[illegible]	[illegible]	[illegible]	[illegible]	[illegible]	[illegible]	[illegible]	[illegible]	[illegible]	[illegible]	[illegible]	[illegible]	[illegible]	[illegible]	[illegible]	[illegible]	[illegible]	[illegible]	[illegible]	[illegible]	[illegible]	[illegible]	[illegible]	[illegible]
Mycobacterium tuberculosis	Multi-drug resistance excluding extensive drug resistance in TB	–	–	0 (0-1)	[illegible]	[illegible]	[illegible]	[illegible]	[illegible]	[illegible]	[illegible]	[illegible]	[illegible]	[illegible]	[illegible]	[illegible]	[illegible]	[illegible]	[illegible]	[illegible]	[illegible]	[illegible]	[illegible]	[illegible]	[illegible]	[illegible]	[illegible]	[illegible]	[illegible]	[illegible]	[illegible]	[illegible]	[illegible]
Mycobacterium tuberculosis	Rifampicin mono-resistance	–	–	0 (0-0)	[illegible]	[illegible]	[illegible]	[illegible]	[illegible]	[illegible]	[illegible]	[illegible]	[illegible]	[illegible]	[illegible]	[illegible]	[illegible]	[illegible]	[illegible]	[illegible]	[illegible]	[illegible]	[illegible]	[illegible]	[illegible]	[illegible]	[illegible]	[illegible]	[illegible]	[illegible]	[illegible]	[illegible]	[illegible]
Mycobacterium tuberculosis	Resistance to one or more antibiotics	–	–	1 (1-2)	[illegible]	[illegible]	[illegible]	[illegible]	[illegible]	[illegible]	[illegible]	[illegible]	[illegible]	[illegible]	[illegible]	[illegible]	[illegible]	[illegible]	[illegible]	[illegible]	[illegible]	[illegible]	[illegible]	[illegible]	[illegible]	[illegible]	[illegible]	[illegible]	[illegible]	[illegible]	[illegible]	[illegible]	[illegible]
Neisseria gonorrhoeae	Fluoroquinolones	–	–	–	[illegible]	[illegible]	[illegible]	[illegible]	[illegible]	[illegible]	[illegible]	[illegible]	[illegible]	[illegible]	[illegible]	[illegible]	[illegible]	[illegible]	[illegible]	[illegible]	[illegible]	[illegible]	[illegible]	[illegible]	[illegible]	[illegible]	[illegible]	[illegible]	[illegible]	[illegible]	[illegible]	[illegible]	[illegible]
Neisseria gonorrhoeae	Third-generation cephalosporins	–	–	–	[illegible]	[illegible]	[illegible]	[illegible]	[illegible]	[illegible]	[illegible]	[illegible]	[illegible]	[illegible]	[illegible]	[illegible]	[illegible]	[illegible]	[illegible]	[illegible]	[illegible]	[illegible]	[illegible]	[illegible]	[illegible]	[illegible]	[illegible]	[illegible]	[illegible]	[illegible]	[illegible]	[illegible]	[illegible]
Neisseria gonorrhoeae	Resistance to one or more antibiotics	–	–	–	[illegible]	[illegible]	[illegible]	[illegible]	[illegible]	[illegible]	[illegible]	[illegible]	[illegible]	[illegible]	[illegible]	[illegible]	[illegible]	[illegible]	[illegible]	[illegible]	[illegible]	[illegible]	[illegible]	[illegible]	[illegible]	[illegible]	[illegible]	[illegible]	[illegible]	[illegible]	[illegible]	[illegible]	[illegible]
Proteus spp.	Aminoglycosides	441 (286-675)	16.0 (10.4-24.5)	[illegible]	[illegible]	[illegible]	[illegible]	[illegible]	[illegible]	[illegible]	[illegible]	[illegible]	[illegible]	[illegible]	[illegible]	[illegible]	[illegible]	[illegible]	[illegible]	[illegible]	[illegible]	[illegible]	[illegible]	[illegible]	[illegible]	[illegible]	[illegible]	[illegible]	[illegible]	[illegible]	[illegible]	[illegible]	[illegible]
Proteus spp.	Aminopenicillin	1250 (818-1890)	45.6 (29.8-68.9)	[illegible]	[illegible]	[illegible]	[illegible]	[illegible]	[illegible]	[illegible]	[illegible]	[illegible]	[illegible]	[illegible]	[illegible]	[illegible]	[illegible]	[illegible]	[illegible]	[illegible]	[illegible]	[illegible]	[illegible]	[illegible]	[illegible]	[illegible]	[illegible]	[illegible]	[illegible]	[illegible]	[illegible]	[illegible]	[illegible]
Proteus spp.	Fluoroquinolones	679 (439-1040)	24.7 (15.9-37.6)	[illegible]	[illegible]	[illegible]	[illegible]	[illegible]	[illegible]	[illegible]	[illegible]	[illegible]	[illegible]	[illegible]	[illegible]	[illegible]	[illegible]	[illegible]	[illegible]	[illegible]	[illegible]	[illegible]	[illegible]	[illegible]	[illegible]	[illegible]	[illegible]	[illegible]	[illegible]	[illegible]	[illegible]	[illegible]	[illegible]
Proteus spp.	Third-generation cephalosporins	336 (208-528)	12.2 (7.6-19.2)	[illegible]	[illegible]	[illegible]	[illegible]	[illegible]	[illegible]	[illegible]	[illegible]	[illegible]	[illegible]	[illegible]	[illegible]	[illegible]	[illegible]	[illegible]	[illegible]	[illegible]	[illegible]	[illegible]	[illegible]	[illegible]	[illegible]	[illegible]	[illegible]	[illegible]	[illegible]	[illegible]	[illegible]	[illegible]	[illegible]
Proteus spp.	Trimethoprim-Sulfamethoxazole	1180 (769-1780)	43.0 (28.0-64.7)	[illegible]	[illegible]	[illegible]	[illegible]	[illegible]	[illegible]	[illegible]	[illegible]	[illegible]	[illegible]	[illegible]	[illegible]	[illegible]	[illegible]	[illegible]	[illegible]	[illegible]	[illegible]	[illegible]	[illegible]	[illegible]	[illegible]	[illegible]	[illegible]	[illegible]	[illegible]	[illegible]	[illegible]	[illegible]	[illegible]
Proteus spp.	Resistance to one or more antibiotics	1450 (942-2180)	52.6 (34.3-79.1)	[illegible]	[illegible]	[illegible]	[illegible]	[illegible]	[illegible]	[illegible]	[illegible]	[illegible]	[illegible]	[illegible]	[illegible]	[illegible]	[illegible]	[illegible]	[illegible]	[illegible]	[illegible]	[illegible]	[illegible]	[illegible]	[illegible]	[illegible]	[illegible]	[illegible]	[illegible]	[illegible]	[illegible]	[illegible]	[illegible]
Pseudomonas aeruginosa	Aminoglycosides	8520 (6180-11400)	309.7 (224.9-415.3)	[illegible]	[illegible]	[illegible]	[illegible]	[illegible]	[illegible]	[illegible]	[illegible]	[illegible]	[illegible]	[illegible]	[illegible]	[illegible]	[illegible]	[illegible]	[illegible]	[illegible]	[illegible]	[illegible]	[illegible]	[illegible]	[illegible]	[illegible]	[illegible]	[illegible]	[illegible]	[illegible]	[illegible]	[illegible]	[illegible]
Pseudomonas aeruginosa	Anti-pseudomonal penicillin/Beta-Lactamase inhibitors	8950 (6380-11900)	325.5 (231.8-432.3)	[illegible]	[illegible]	[illegible]	[illegible]	[illegible]	[illegible]	[illegible]	[illegible]	[illegible]	[illegible]	[illegible]	[illegible]	[illegible]	[illegible]	[illegible]	[illegible]	[illegible]	[illegible]	[illegible]	[illegible]	[illegible]	[illegible]	[illegible]	[illegible]	[illegible]	[illegible]	[illegible]	[illegible]	[illegible]	[illegible]
Pseudomonas aeruginosa	Carbapenem	7300 (5260-9830)	265.6 (191.1-357.4)	[illegible]	[illegible]	[illegible]	[illegible]	[illegible]	[illegible]	[illegible]	[illegible]	[illegible]	[illegible]	[illegible]	[illegible]	[illegible]	[illegible]	[illegible]	[illegible]	[illegible]	[illegible]	[illegible]	[illegible]	[illegible]	[illegible]	[illegible]	[illegible]	[illegible]	[illegible]	[illegible]	[illegible]	[illegible]	[illegible]
Pseudomonas aeruginosa	Fluoroquinolones	9510 (6890-12500)	346.0 (250.5-455.0)	[illegible]	[illegible]	[illegible]	[illegible]	[illegible]	[illegible]	[illegible]	[illegible]	[illegible]	[illegible]	[illegible]	[illegible]	[illegible]	[illegible]	[illegible]	[illegible]	[illegible]	[illegible]	[illegible]	[illegible]	[illegible]	[illegible]	[illegible]	[illegible]	[illegible]	[illegible]	[illegible]	[illegible]	[illegible]	[illegible]
Pseudomonas aeruginosa	Fourth-generation cephalosporins	8340 (6060-11200)	303.3 (220.4-406.2)	[illegible]	[illegible]	[illegible]	[illegible]	[illegible]	[illegible]	[illegible]	[illegible]	[illegible]	[illegible]	[illegible]	[illegible]	[illegible]	[illegible]	[illegible]	[illegible]	[illegible]	[illegible]	[illegible]	[illegible]	[illegible]	[illegible]	[illegible]	[illegible]	[illegible]	[illegible]	[illegible]	[illegible]	[illegible]	[illegible]
Pseudomonas aeruginosa	Third-generation cephalosporins	12700 (9180-17200)	462.6 (333.9-625.3)	[illegible]	[illegible]	[illegible]	[illegible]	[illegible]	[illegible]	[illegible]	[illegible]	[illegible]	[illegible]	[illegible]	[illegible]	[illegible]	[illegible]	[illegible]	[illegible]	[illegible]	[illegible]	[illegible]	[illegible]	[illegible]	[illegible]	[illegible]	[illegible]	[illegible]	[illegible]	[illegible]	[illegible]	[illegible]	[illegible]
Pseudomonas aeruginosa	Resistance to one or more antibiotics	18700 (13500-24700)	678.9 (489.9-899.6)	[illegible]	[illegible]	[illegible]	[illegible]	[illegible]	[illegible]	[illegible]	[illegible]	[illegible]	[illegible]	[illegible]	[illegible]	[illegible]	[illegible]	[illegible]	[illegible]	[illegible]	[illegible]	[illegible]	[illegible]	[illegible]	[illegible]	[illegible]	[illegible]	[illegible]	[illegible]	[illegible]	[illegible]	[illegible]	[illegible]
Salmonella Paratyphi	Fluoroquinolones	–	–	0 (0-0)	[illegible]	[illegible]	[illegible]	[illegible]	[illegible]	[illegible]	[illegible]	[illegible]	[illegible]	[illegible]	[illegible]	[illegible]	[illegible]	[illegible]	[illegible]	[illegible]	[illegible]	[illegible]	[illegible]	[illegible]	[illegible]	[illegible]	[illegible]	[illegible]	[illegible]	[illegible]	[illegible]	[illegible]	[illegible]
Salmonella Paratyphi	Multi-drug resistance in Salmonella Typhi and Paratyphi	–	–	0 (0-0)	[illegible]	[illegible]	[illegible]	[illegible]	[illegible]	[illegible]	[illegible]	[illegible]	[illegible]	[illegible]	[illegible]	[illegible]	[illegible]	[illegible]	[illegible]	[illegible]	[illegible]	[illegible]	[illegible]	[illegible]	[illegible]	[illegible]	[illegible]	[illegible]	[illegible]	[illegible]	[illegible]	[illegible]	[illegible]
Salmonella Paratyphi	Resistance to one or more antibiotics	–	–	0 (0-0)	[illegible]	[illegible]	[illegible]	[illegible]	[illegible]	[illegible]	[illegible]	[illegible]	[illegible]	[illegible]	[illegible]	[illegible]	[illegible]	[illegible]	[illegible]	[illegible]	[illegible]	[illegible]	[illegible]	[illegible]	[illegible]	[illegible]	[illegible]	[illegible]	[illegible]	[illegible]	[illegible]	[illegible]	[illegible]
Salmonella Typhi	Fluoroquinolones	6550 (3660-10900)	238.2 (132.9-397.1)	[illegible]	[illegible]	[illegible]	[illegible]	[illegible]	[illegible]	[illegible]	[illegible]	[illegible]	[illegible]	[illegible]	[illegible]	[illegible]	[illegible]	[illegible]	[illegible]	[illegible]	[illegible]	[illegible]	[illegible]	[illegible]	[illegible]	[illegible]	[illegible]	[illegible]	[illegible]	[illegible]	[illegible]	[illegible]	[illegible]
Salmonella Typhi	Multi-drug resistance in Salmonella Typhi and Paratyphi	13700 (8190-21100)	497.9 (297.9-767.3)	[illegible]	[illegible]	[illegible]	[illegible]	[illegible]	[illegible]	[illegible]	[illegible]	[illegible]	[illegible]	[illegible]	[illegible]	[illegible]	[illegible]	[illegible]	[illegible]	[illegible]	[illegible]	[illegible]	[illegible]	[illegible]	[illegible]	[illegible]	[illegible]	[illegible]	[illegible]	[illegible]	[illegible]	[illegible]	[illegible]
Salmonella Typhi	Resistance to one or more antibiotics	16800 (10100-26000)	612.1 (368.1-946.6)	[illegible]	[illegible]	[illegible]	[illegible]	[illegible]	[illegible]	[illegible]	[illegible]	[illegible]	[illegible]	[illegible]	[illegible]	[illegible]	[illegible]	[illegible]	[illegible]	[illegible]	[illegible]	[illegible]	[illegible]	[illegible]	[illegible]	[illegible]	[illegible]	[illegible]	[illegible]	[illegible]	[illegible]	[illegible]	[illegible]
Non-typhoidal *Salmonella*	Fluoroquinolones	273 (135-486)	9.9 (4.9-17.7)	[illegible]	[illegible]	[illegible]	[illegible]	[illegible]	[illegible]	[illegible]	[illegible]	[illegible]	[illegible]	[illegible]	[illegible]	[illegible]	[illegible]	[illegible]	[illegible]	[illegible]	[illegible]	[illegible]	[illegible]	[illegible]	[illegible]	[illegible]	[illegible]	[illegible]	[illegible]	[illegible]	[illegible]	[illegible]	[illegible]
Non-typhoidal *Salmonella*	Resistance to one or more antibiotics	273 (135-486)	9.9 (4.9-17.7)	[illegible]	[illegible]	[illegible]	[illegible]	[illegible]	[illegible]	[illegible]	[illegible]	[illegible]	[illegible]	[illegible]	[illegible]	[illegible]	[illegible]	[illegible]	[illegible]	[illegible]	[illegible]	[illegible]	[illegible]	[illegible]	[illegible]	[illegible]	[illegible]	[illegible]	[illegible]	[illegible]	[illegible]	[illegible]	[illegible]
Serratia spp.	Aminoglycosides	3470 (2160-5190)	126.3 (78.6-188.6)	[illegible]	[illegible]	[illegible]	[illegible]	[illegible]	[illegible]	[illegible]	[illegible]	[illegible]	[illegible]	[illegible]	[illegible]	[illegible]	[illegible]	[illegible]	[illegible]	[illegible]	[illegible]	[illegible]	[illegible]	[illegible]	[illegible]	[illegible]	[illegible]	[illegible]	[illegible]	[illegible]	[illegible]	[illegible]	[illegible]
Serratia spp.	Anti-pseudomonal penicillin/Beta-Lactamase inhibitors	1510 (927-2320)	54.9 (33.7-84.3)	[illegible]	[illegible]	[illegible]	[illegible]	[illegible]	[illegible]	[illegible]	[illegible]	[illegible]	[illegible]	[illegible]	[illegible]	[illegible]	[illegible]	[illegible]	[illegible]	[illegible]	[illegible]	[illegible]	[illegible]	[illegible]	[illegible]	[illegible]	[illegible]	[illegible]	[illegible]	[illegible]	[illegible]	[illegible]	[illegible]
Serratia spp.	Carbapenem	392 (241-607)	14.3 (8.8-22.1)	[illegible]	[illegible]	[illegible]	[illegible]	[illegible]	[illegible]	[illegible]	[illegible]	[illegible]	[illegible]	[illegible]	[illegible]	[illegible]	[illegible]	[illegible]	[illegible]	[illegible]	[illegible]	[illegible]	[illegible]	[illegible]	[illegible]	[illegible]	[illegible]	[illegible]	[illegible]	[illegible]	[illegible]	[illegible]	[illegible]
Serratia spp.	Fluoroquinolones	1680 (1050-2550)	61.2 (38.3-92.7)	[illegible]	[illegible]	[illegible]	[illegible]	[illegible]	[illegible]	[illegible]	[illegible]	[illegible]	[illegible]	[illegible]	[illegible]	[illegible]	[illegible]	[illegible]	[illegible]	[illegible]	[illegible]	[illegible]	[illegible]	[illegible]	[illegible]	[illegible]	[illegible]	[illegible]	[illegible]	[illegible]	[illegible]	[illegible]	[illegible]
Serratia spp.	Fourth-generation cephalosporins	5430 (3450-8190)	197.6 (125.4-297.7)	[illegible]	[illegible]	[illegible]	[illegible]	[illegible]	[illegible]	[illegible]	[illegible]	[illegible]	[illegible]	[illegible]	[illegible]	[illegible]	[illegible]	[illegible]	[illegible]	[illegible]	[illegible]	[illegible]	[illegible]	[illegible]	[illegible]	[illegible]	[illegible]	[illegible]	[illegible]	[illegible]	[illegible]	[illegible]	[illegible]
Serratia spp.	Third-generation cephalosporins	4560 (2880-6820)	165.7 (104.6-247.9)	[illegible]	[illegible]	[illegible]	[illegible]	[illegible]	[illegible]	[illegible]	[illegible]	[illegible]	[illegible]	[illegible]	[illegible]	[illegible]	[illegible]	[illegible]	[illegible]	[illegible]	[illegible]	[illegible]	[illegible]	[illegible]	[illegible]	[illegible]	[illegible]	[illegible]	[illegible]	[illegible]	[illegible]	[illegible]	[illegible]
Serratia spp.	Resistance to one or more antibiotics	6840 (4330-10200)	248.8 (157.4-371.9)	[illegible]	[illegible]	[illegible]	[illegible]	[illegible]	[illegible]	[illegible]	[illegible]	[illegible]	[illegible]	[illegible]	[illegible]	[illegible]	[illegible]	[illegible]	[illegible]	[illegible]	[illegible]	[illegible]	[illegible]	[illegible]	[illegible]	[illegible]	[illegible]	[illegible]	[illegible]	[illegible]	[illegible]	[illegible]	[illegible]
Shigella spp.	Fluoroquinolones	434 (167-861)	15.8 (6.1-31.3)	[illegible]	[illegible]	[illegible]	[illegible]	[illegible]	[illegible]	[illegible]	[illegible]	[illegible]	[illegible]	[illegible]	[illegible]	[illegible]	[illegible]	[illegible]	[illegible]	[illegible]	[illegible]	[illegible]	[illegible]	[illegible]	[illegible]	[illegible]	[illegible]	[illegible]	[illegible]	[illegible]	[illegible]	[illegible]	[illegible]
Shigella spp.	Resistance to one or more antibiotics	434 (167-861)	15.8 (6.1-31.3)	[illegible]	[illegible]	[illegible]	[illegible]	[illegible]	[illegible]	[illegible]	[illegible]	[illegible]	[illegible]	[illegible]	[illegible]	[illegible]	[illegible]	[illegible]	[illegible]	[illegible]	[illegible]	[illegible]	[illegible]	[illegible]	[illegible]	[illegible]	[illegible]	[illegible]	[illegible]	[illegible]	[illegible]	[illegible]	[illegible]
Staphylococcus aureus	Fluoroquinolones	7420 (5300-10100)	269.7 (192.8-367.7)	[illegible]	[illegible]	[illegible]	[illegible]	[illegible]	[illegible]	[illegible]	[illegible]	[illegible]	[illegible]	[illegible]	[illegible]	[illegible]	[illegible]	[illegible]	[illegible]	[illegible]	[illegible]	[illegible]	[illegible]	[illegible]	[illegible]	[illegible]	[illegible]	[illegible]	[illegible]	[illegible]	[illegible]	[illegible]	[illegible]

Staphylococcus aureus	Macrolide	15600 (11500-21000)	566.1 (417.0-762.5)	1390000 (1020000-1870000)	50396.1 (37112.1-67888.2)	543 (216-946)	19.7 (7.9-34.4)	48300 (19300-84200)	1758.1 (701.6-3060.9)	9980 (7560-13000)	31.0 (23.5-40.4)	883000 (668000-1150000)	2747.9 (2080.4-3573.5)	347 (150-606)	1.1 (0.5-1.9)	30700 (13300-53600)	95.5 (41.3-166.8)
Staphylococcus aureus	Methicillin	14100 (10100-19200)	514.3 (365.9-697.7)	1260000 (896000-1710000)	45781.8 (32575.2-62185.0)	3290 (1350-5690)	119.5 (49.0-206.9)	292000 (120000-507000)	10635.7 (4359.0-18428.2)	9090 (6810-12000)	28.3 (21.2-37.4)	805000 (603000-1060000)	2504.4 (1875.5-3307.6)	2110 (862-3700)	6.6 (2.7-11.5)	187000 (76400-327000)	581.2 (237.8-1019.3)
Staphylococcus aureus	Trimethoprim-Sulfamethoxazole	20200 (14800-27200)	735.3 (538.9-990.5)	1800000 (1320000-2420000)	65453.7 (47950.3-88185.9)	2100 (1090-3270)	76.3 (39.6-119.1)	186000 (96900-291000)	6782.5 (3525.5-10591.4)	13100 (9890-17200)	40.7 (30.8-53.4)	1160000 (875000-1520000)	3601.2 (2722.9-4726.0)	1350 (711-2080)	4.2 (2.2-6.5)	120000 (62900-184000)	372.8 (195.8-573.5)
Staphylococcus aureus	Vancomycin	460 (327-637)	16.7 (11.9-23.2)	41000 (29100-56700)	1489.7 (1058.2-2061.5)	124 (62-211)	4.5 (2.3-7.7)	11000 (5530-18800)	399.8 (201.0-683.4)	309 (227-417)	1.0 (0.7-1.3)	27400 (20100-36900)	85.1 (62.5-114.8)	83 (42-138)	0.3 (0.1-0.4)	7310 (3720-12200)	22.8 (11.6-38.0)
Staphylococcus aureus	Resistance to one or more antibiotics	28800 (21200-38600)	1048.4 (772.4-1403.3)	2570000 (1890000-3440000)	93327.5 (68740.0-124945.7)	6350 (3920-9460)	231.0 (142.7-343.9)	565000 (349000-841000)	20553.3 (12692.9-30602.9)	18500 (13900-24100)	57.6 (43.3-75.0)	1640000 (1230000-2130000)	5100.3 (3829.7-6638.9)	4090 (2500-5980)	12.7 (7.8-18.6)	362000 (222000-529000)	1125.6 (689.7-1647.5)
Streptococcus pneumoniae	Beta Lactam/Beta-lactamase inhibitors	5820 (4160-7990)	211.6 (151.1-290.5)	518000 (370000-711000)	18855.9 (13470.9-25870.9)	162 (0-425)	5.9 (0.0-15.4)	14400 (46-37800)	524.7 (1.7-1375.3)	11700 (8550-15500)	36.4 (26.6-48.3)	1040000 (756000-1370000)	3223.0 (2352.4-4273.0)	323 (1-821)	1.0 (0.0-2.6)	28600 (67-72600)	88.9 (0.2-226.0)
Streptococcus pneumoniae	Carbapenem	6600 (4670-9140)	240.2 (169.8-332.3)	589000 (416000-815000)	21410.3 (15123.0-29631.9)	1400 (613-2480)	50.7 (22.3-90.1)	124000 (54700-221000)	4525.4 (1990.3-8026.4)	13500 (10100-17800)	41.9 (31.4-55.5)	1190000 (892000-1580000)	3702.8 (2775.3-4912.3)	2830 (1280-5050)	8.8 (4.0-15.7)	251000 (113000-447000)	779.9 (352.5-1390.4)
Streptococcus pneumoniae	Fluoroquinolones	6840 (4940-9280)	248.6 (179.6-337.4)	609000 (440000-826000)	22148.4 (15990.9-30051.2)	830 (193-1690)	30.2 (7.0-61.6)	73800 (17200-151000)	2685.0 (624.9-5480.2)	14300 (10600-18800)	44.5 (33.1-58.6)	1270000 (940000-1670000)	3938.6 (2926.5-5186.2)	1740 (418-3530)	5.4 (1.3-11.0)	154000 (37000-312000)	479.7 (115.2-971.1)
Streptococcus pneumoniae	Macrolide	6720 (4800-9150)	244.5 (174.7-332.7)	599000 (428000-815000)	21793.6 (15571.7-29623.0)	234 (0-583)	8.5 (0.0-21.2)	20800 (0-51800)	756.1 (0.0-1883.4)	13900 (10500-18200)	43.2 (32.6-56.8)	1230000 (926000-1610000)	3817.9 (2881.5-5021.7)	484 (0-1220)	1.5 (0.0-3.8)	42800 (0-108000)	133.2 (0.0-336.8)
Streptococcus pneumoniae	Penicillin	9540 (6800-13400)	347.0 (247.3-486.3)	851000 (607000-1190000)	30949.6 (22081.5-43391.4)	540 (271-944)	19.6 (9.9-34.3)	48100 (24200-84100)	1748.9 (879.6-3060.1)	19000 (13900-25600)	59.2 (43.2-79.8)	1680000 (1230000-2270000)	5238.4 (3817.4-7055.0)	1080 (529-1910)	3.4 (1.6-5.9)	95500 (46700-169000)	297.2 (145.5-524.5)
Streptococcus pneumoniae	Third-generation cephalosporins	4320 (2990-6200)	157.0 (108.9-225.3)	385000 (267000-552000)	13992.8 (9703.7-20090.0)	208 (79-435)	7.6 (2.9-15.8)	18600 (7060-38800)	676.1 (256.8-1410.6)	8840 (6380-12000)	27.5 (19.9-37.4)	782000 (564000-1060000)	2434.6 (1755.5-3310.1)	424 (159-857)	1.3 (0.5-2.7)	37500 (14100-75800)	116.6 (43.8-235.8)
Streptococcus pneumoniae	Trimethoprim-Sulfamethoxazole	27100 (20000-36600)	984.7 (728.7-1329.6)	2410000 (1790000-3260000)	87784.6 (64932.8-118547.6)	2470 (351-5070)	89.8 (12.8-184.4)	220000 (31400-451000)	7993.8 (1143.1-16403.3)	55300 (42300-70800)	172.0 (131.8-220.4)	4890000 (3740000-6260000)	15213.9 (11655.2-19490.1)	5040 (738-10200)	15.7 (2.3-31.8)	446000 (65700-904000)	1388.3 (204.5-2813.5)
Streptococcus pneumoniae	Resistance to one or more antibiotics	29200 (21800-39300)	1063.0 (791.4-1430.0)	2610000 (1940000-3510000)	94763.5 (70465.1-127503.9)	5840 (3670-8590)	212.4 (133.4-312.2)	520000 (326000-763000)	18909.9 (11864.9-27766.4)	59700 (45800-76100)	185.9 (142.6-236.7)	5280000 (4050000-6730000)	16441.0 (12619.3-20940.3)	11900 (7540-17200)	37.1 (23.5-53.6)	1060000 (667000-1520000)	3283.8 (2074.9-4742.9)

Staphylococcus aureus	Macrolide	7730 (5560-10500)	5.9 (4.3-8.0)	669000 (481000-906000)	511.2 (367.7-693.1)	268 (110-476)	0.2 (0.1-0.4)	23200 (9700-41200)	17.7 (7.4-31.5)	39300 (31200-50600)	4.3 (3.4-5.5)	1190000 (926000-1560000)	130.0 (101.5-170.9)	1410 (593-2340)	0.2 (0.1-0.3)	42400 (18200-70900)	4.6 (2.0-7.8)
Staphylococcus aureus	Methicillin	7090 (4960-9780)	5.4 (3.8-7.5)	613000 (429000-844000)	468.4 (328.2-645.0)	1650 (665-2930)	1.3 (0.5-2.2)	142000 (57600-253000)	108.9 (44.1-193.1)	34100 (26200-44700)	3.7 (2.9-4.9)	1030000 (780000-1370000)	112.6 (85.4-150.0)	7940 (3430-13700)	0.9 (0.4-1.5)	238000 (102000-410000)	26.1 (11.1-45.0)
Staphylococcus aureus	Trimethoprim-Sulfamethoxazole	9850 (7140-13300)	7.5 (5.5-10.2)	852000 (617000-1150000)	651.5 (471.8-881.6)	1010 (514-1580)	0.8 (0.4-1.2)	87300 (44100-136000)	66.7 (33.7-103.8)	49700 (39000-64300)	5.4 (4.3-7.0)	1520000 (1160000-2000000)	166.2 (127.6-219.2)	5250 (2800-8150)	0.6 (0.3-0.9)	159000 (84400-250000)	17.4 (9.3-27.4)
Staphylococcus aureus	Vancomycin	246 (169-346)	0.2 (0.1-0.3)	21300 (14600-29900)	16.3 (11.2-22.9)	65 (32-113)	0.0 (0.0-0.1)	5650 (2810-9750)	4.3 (2.2-7.5)	1140 (858-1550)	0.1 (0.1-0.2)	34700 (25600-48100)	3.8 (2.8-5.3)	312 (163-534)	0.0 (0.0-0.1)	9480 (4960-16300)	1.0 (0.5-1.8)
Staphylococcus aureus	Resistance to one or more antibiotics	14000 (10100-18900)	10.7 (7.7-14.4)	1210000 (872000-1640000)	926.0 (666.5-1251.7)	3130 (1850-4730)	2.4 (1.4-3.6)	270000 (160000-408000)	206.7 (122.1-312.2)	73700 (58600-94700)	8.1 (6.4-10.4)	2230000 (1740000-2930000)	244.8 (190.9-321.6)	15800 (10200-23000)	1.7 (1.1-2.5)	475000 (302000-706000)	52.1 (33.1-77.3)
Streptococcus pneumoniae	Beta Lactam/Beta-lactamase inhibitors	8440 (5900-11500)	6.5 (4.5-8.8)	728000 (509000-989000)	556.3 (388.8-756.5)	232 (0-590)	0.2 (0.0-0.5)	20000 (43-50800)	15.3 (0.0-38.8)	13800 (10800-17200)	1.5 (1.2-1.9)	526000 (404000-664000)	57.6 (44.3-72.7)	380 (1-955)	0.0 (0.0-0.1)	14500 (91-36500)	1.6 (0.0-4.0)
Streptococcus pneumoniae	Carbapenem	9750 (6920-13500)	7.5 (5.3-10.3)	841000 (597000-1160000)	642.9 (456.3-888.5)	2070 (915-3690)	1.6 (0.7-2.8)	178000 (79000-318000)	136.2 (60.4-243.1)	16700 (13400-20800)	1.8 (1.5-2.3)	623000 (493000-783000)	68.3 (54.1-85.8)	3520 (1650-5930)	0.4 (0.2-0.6)	131000 (62500-222000)	14.4 (6.8-24.4)
Streptococcus pneumoniae	Fluoroquinolones	9650 (6880-13100)	7.4 (5.3-10.0)	832000 (593000-1130000)	636.2 (453.7-863.3)	1170 (281-2410)	0.9 (0.2-1.8)	101000 (24200-208000)	77.2 (18.5-158.8)	16700 (13300-20800)	1.8 (1.5-2.3)	653000 (519000-812000)	71.5 (56.9-89.0)	2030 (497-4020)	0.2 (0.1-0.4)	78800 (19500-158000)	8.6 (2.1-17.3)
Streptococcus pneumoniae	Macrolide	10400 (7390-14200)	7.9 (5.7-10.9)	894000 (637000-1220000)	683.5 (487.0-936.2)	362 (0-929)	0.3 (0.0-0.7)	31200 (0-79900)	23.8 (0.0-61.1)	15900 (12900-19600)	1.7 (1.4-2.1)	595000 (479000-731000)	65.2 (52.5-80.1)	560 (0-1380)	0.1 (0.0-0.2)	20600 (0-50700)	2.3 (0.0-5.6)
Streptococcus pneumoniae	Penicillin	14000 (9480-19300)	10.7 (7.2-14.8)	1200000 (817000-1670000)	920.2 (624.7-1273.6)	820 (391-1480)	0.6 (0.3-1.1)	70600 (33700-127000)	54.0 (25.8-97.2)	21700 (17700-26600)	2.4 (1.9-2.9)	804000 (647000-995000)	88.1 (70.9-109.0)	1130 (631-1890)	0.1 (0.1-0.2)	41300 (23100-68100)	4.5 (2.5-7.5)
Streptococcus pneumoniae	Third-generation cephalosporins	6450 (4430-9100)	4.9 (3.4-7.0)	556000 (382000-785000)	425.0 (291.9-599.9)	314 (116-650)	0.2 (0.1-0.5)	27000 (9990-56000)	20.7 (7.6-42.8)	9840 (7750-12300)	1.1 (0.8-1.4)	370000 (287000-467000)	40.5 (31.5-51.2)	414 (165-785)	0.0 (0.0-0.1)	15700 (6410-29200)	1.7 (0.7-3.2)
Streptococcus pneumoniae	Trimethoprim-Sulfamethoxazole	40600 (29800-53700)	31.0 (22.8-41.0)	3500000 (2570000-4620000)	2676.0 (1964.3-3534.9)	3730 (544-7740)	2.9 (0.4-5.9)	322000 (47000-666000)	245.9 (35.9-508.9)	64000 (54100-76200)	7.0 (5.9-8.4)	2410000 (2000000-2920000)	264.6 (219.6-319.6)	5850 (814-11400)	0.6 (0.1-1.2)	218000 (30000-430000)	23.9 (3.3-47.1)
Streptococcus pneumoniae	Resistance to one or more antibiotics	43800 (32200-57800)	33.5 (24.6-44.2)	3780000 (2780000-4980000)	2887.7 (2124.1-3810.8)	8700 (5300-12800)	6.7 (4.1-9.8)	750000 (457000-1110000)	573.1 (349.2-845.1)	69300 (58900-82200)	7.6 (6.5-9.0)	2610000 (2180000-3140000)	286.4 (238.9-343.9)	13900 (9170-19000)	1.5 (1.0-2.1)	521000 (340000-710000)	57.0 (37.3-77.8)

Table S21. Limitations of each primary modelling component					
**Type of limitation**	**1. Fraction of sepsis and infectious syndromes**	**2. Case fatality ratio**	**3. Pathogen distribution**	**4. Fraction of resistance**	**5. Relative risk**
**Input data**	Limited geographic coverage in the data. Analysis of the fraction of sepsis included multiple cause of death (MCoD), hospital discharge, linkage, and CHAMPS surveillance data which represented 16 countries	Almost all of the input data relies on hospital-based sampling, excluding all community infections and deaths from the analysis.	Many data sources like microbial databases and hospital discharges rely on hospital-based sampling, excluding community-based infections.	There is heterogeneity in the interpretation guidelines used for antimicrobial susceptibility test and in most cases it was not possible to harmonise this interpretation to the most recent CLSI guidelines.	There was limited data on the fatalities due to resistant Neisseria gonorrhoeae so we did not produce a fatal estimate for this pathogen.
		Literature review of case fatality ratios available for central nervous system infections only.	We excluded from analysis all records of infection where no pathogen was detected. This assumes that all pathogens are equally like to be detected, which may be untrue to due issues like variable diagnostic methods (PCR, culture, etc) and irregular testing.	There is heterogineity in the breath of data gathered for the 17 core pathogen-drug combinations, for which we implemented a systematic review, and the remaining 69 combinations for which no systematic review was implemented.	There was limited data on fatalities due to resistant Shigella and Salmonella Parathyphi, for which we based our results on estimates for Salmonella Typhi.
		Because each data source generally reported only a set of the pathogens we evaluated in our research, the input data for the pathogens varied in geographic coverage; nearly all pathogens were well reported in high-income areas, but some pathogens were not well represented in the smaller subset of data we collected from low- and middle-income locations.	Lack of diagnostic data linked with microbiology lab results for some data sources forces us to rely only on specimen type to assign infectious syndrome, obscuring the primary site of infection for samples such as blood.	Were unable to collect data on resistance prevalence more recently than 2018; we estimated resistance for 2018 and assumed no change in prevalence of resistance for 2019.	There was limited availability of outcomes dissagregated by age and gender groups and different anatomical sites of infection.
		For some pathogen-syndrome combinations, we had no data available with outcomes that could be used to model CFRs; for these, we defaulted to a general bacteria model of CFR.		Some facilities could not be classified as tertiary/non-tertiary due to vague facilities names and/or lack of sufficient website information (see appendix section 7.2).	
				When data providers did not describe a facility as tertiary or non-tertiary and the name of the facility was not descriptive, we used self-assignments of tertiary/non-tertiary from facility websites. This form of classification is vulnerable to differences in tertiary/non-tertiary definitions (such as describing a facility as tertiary when only a select ward offers tertiary care).	

	Tuberculosis, Gonorrhoea and chlamydia, and Typhoid, paratyphoid, and invasive non-typhoidal Salmonella were not included as infectious syndrome models. We used GBD 2019 results by setting the fraction of sepsis to 100% for all deaths in these causes prior to multiplying onto the sepsis-related mortality envelope	Pathogen-syndrome-specific case fatality ratios assumed to be constant across underlying cause of illness.	The distribution of pathogens causing a given infectious syndrome is assumed to be constant across the underlying cause of illness.	We assumed the same fraction of resistance for different age and gender groups, for different sites of infection and hospital or community acquired settings.	We assumed the same relative risk for different age and gender groups, different anatomical sites of infection, and different locations.
**Modelling**	We assumed that infections were community acquired if they were listed as primary diagnoses or underlying causes of death. Otherwise, the infection was assumed to be hospital acquired.	Geographic distribution of CFR based solely on Healthcare Access and Quality Index.	Co-infection of pathogens is assumed to be random and not correlated for certain pathogens.	We assumed that the correlation structure of co-resistance of multiple drugs for the same pathogen was the same as the average across regions	We assumed that the relative risk of a co-resistant infection is the same as the highest mono-resistant relative risk among the antibiotics considered, which is likely an underestimation of the true co-resistant relative risk.
	Data stochasticity. At the most granular age-, sex-, location-, year-, cause-specific level, the dataset for each individual model is stochastic and there are few clusters to guide the model fit.		Use of expert-opinion Gaussian priors with mean 0 and non-zero variance on model coefficients to bias the models away from spurious effects driven by data sparsity.	We included designations of "mixed/unknown" in the crosswalk to attenuate the effect of tertiary samples in our results. This was due to the low proportion of definitively non-tertiary samples in several super regions for which we produce estimates.	We assumed that the relative length of stay has the same impact duration for all sequelae, which impacts specifically bloodstream infections and intraabdominal infections
			Pathogen distribution of cardiac infections is assumed to the same as bloodstream infections.		

Table S22. Global deaths and DALYs (in counts and all-age rates) associated with and attributable to bacterial antimicrobial resistance by pathogen–drug combination, 2019

Pathogen	Antibiotic Class	Associated with resistance				Attributable to resistance			
		Deaths		DALYs		Deaths		DALYs	
		Counts (thousands)	Rate per 100 000	Counts (thousands)	Rate per 100 000	Counts (thousands)	Rate per 100 000	Counts (thousands)	Rate per 100 000
All pathogens	Resistance to one or more antibiotics	4,950 (3,620-6,570)	64.0 (46.8-84.9)	192,000 (146,000-248,000)	2,477.7 (1,889.9-3,199.1)	1,270 (911-1,710)	16.4 (11.8-22.0)	47,900 (35,300-63,700)	618.7 (455.7-823.2)
Acinetobacter baumannii	Aminoglycosides	239 (140-374)	3.1 (1.8-4.8)	6,730 (4,120-10,300)	86.9 (53.3-133.5)	10.4 (0-26.7)	0.1 (0.0-0.3)	296 (1.84-737)	3.8 (0.0-9.5)
Acinetobacter baumannii	Anti-pseudomonal penicillin/Beta-Lactamase inhibitors	359 (213-551)	4.6 (2.8-7.1)	10,300 (6,400-15,600)	132.7 (82.7-202.2)	13.3 (6.88-22.6)	0.2 (0.1-0.3)	463 (243-753)	6.0 (3.1-9.7)
Acinetobacter baumannii	Beta Lactam/Beta-lactamase inhibitors	306 (182-469)	3.9 (2.4-6.1)	8,490 (5,260-13,000)	109.8 (68.0-167.5)	0.811 (0.219-1.65)	0.0 (0.0-0.0)	17.3 (4.64-35.1)	0.2 (0.1-0.5)
Acinetobacter baumannii	Carbapenem	326 (192-504)	4.2 (2.5-6.5)	8,790 (5,380-13,400)	113.6 (69.5-173.5)	57.7 (30.3-102)	0.7 (0.4-1.3)	1,540 (817-2,630)	19.9 (10.6-34.0)
Acinetobacter baumannii	Fluoroquinolones	316 (186-488)	4.1 (2.4-6.3)	8,800 (5,390-13,300)	113.7 (69.7-172.0)	40 (19.8-66.6)	0.5 (0.3-0.9)	1,120 (557-1,880)	14.4 (7.2-24.3)
Acinetobacter baumannii	Fourth-generation cephalosporins	360 (212-554)	4.7 (2.7-7.2)	9,880 (6,090-15,000)	127.7 (78.7-194.4)	3.28 (1.36-6.27)	0.0 (0.0-0.1)	79.1 (33.7-150)	1.0 (0.4-1.9)
Acinetobacter baumannii	Third-generation cephalosporins	377 (222-575)	4.9 (2.9-7.4)	10,400 (6,430-15,800)	134.3 (83.1-204.6)	6.86 (2.66-12.9)	0.1 (0.0-0.2)	163 (64.7-306)	2.1 (0.8-4.0)
Acinetobacter baumannii	Resistance to one or more antibiotics	423 (252-647)	5.5 (3.3-8.4)	11,800 (7,290-17,800)	152.3 (94.2-229.8)	132 (75.7-213)	1.7 (1.0-2.8)	3,670 (2,150-5,760)	47.5 (27.8-74.5)
Citrobacter spp.	Aminoglycosides	8.48 (5.23-12.8)	0.1 (0.1-0.2)	384 (234-584)	5.0 (3.0-7.5)	0.411 (0-1)	0.0 (0.0-0.0)	18.6 (0-45.2)	0.2 (0.0-0.6)
Citrobacter spp.	Anti-pseudomonal penicillin/Beta-Lactamase inhibitors	14.1 (8.65-21.7)	0.2 (0.1-0.3)	568 (348-867)	7.3 (4.5-11.2)	2.17 (1-3.78)	0.0 (0.0-0.0)	83.9 (41.8-147)	1.1 (0.5-1.9)
Citrobacter spp.	Carbapenem	10.4 (6.18-16.1)	0.1 (0.1-0.2)	404 (242-621)	5.2 (3.1-8.0)	2.3 (1.15-4)	0.0 (0.0-0.1)	87.5 (44.1-151)	1.1 (0.6-1.9)
Citrobacter spp.	Fluoroquinolones	17.9 (10.9-27.3)	0.2 (0.1-0.4)	734 (451-1,110)	9.5 (5.8-14.4)	2.51 (1.12-4.58)	0.0 (0.0-0.1)	102 (46.4-191)	1.3 (0.6-2.5)
Citrobacter spp.	Fourth-generation cephalosporins	18.7 (11.4-28.4)	0.2 (0.1-0.4)	821 (498-1,260)	10.6 (6.4-16.2)	1.34 (0.577-2.53)	0.0 (0.0-0.0)	60.7 (27-109)	0.8 (0.3-1.4)
Citrobacter spp.	Third-generation cephalosporins	28.1 (17.3-43)	0.4 (0.2-0.6)	1,120 (689-1,700)	14.5 (8.9-21.9)	1.84 (0.762-3.38)	0.0 (0.0-0.0)	63.4 (26-115)	0.8 (0.3-1.5)
Citrobacter spp.	Resistance to one or more antibiotics	35.5 (21.7-52.9)	0.5 (0.3-0.7)	1,400 (861-2,110)	18.1 (11.1-27.2)	10.6 (5.93-17.3)	0.1 (0.1-0.2)	416 (237-668)	5.4 (3.1-8.6)
Enterobacter spp.	Aminoglycosides	51.6 (34.9-73.6)	0.7 (0.5-1.0)	2,310 (1,600-3,280)	29.9 (20.7-42.3)	3 (0.879-5.87)	0.0 (0.0-0.1)	135 (40.2-253)	1.7 (0.5-3.3)
Enterobacter spp.	Anti-pseudomonal penicillin/Beta-Lactamase inhibitors	99.2 (65.3-142)	1.3 (0.8-1.8)	3,640 (2,460-5,230)	47.0 (31.8-67.6)	9.95 (5.61-15.9)	0.1 (0.1-0.2)	337 (190-532)	4.4 (2.5-6.9)
Enterobacter spp.	Carbapenem	61.6 (40-90.1)	0.8 (0.5-1.2)	2,340 (1,550-3,350)	30.2 (20.1-43.3)	15.3 (8.87-24.2)	0.2 (0.1-0.3)	568 (334-884)	7.3 (4.3-11.4)
Enterobacter spp.	Fluoroquinolones	74.7 (49.2-107)	1.0 (0.6-1.4)	3,150 (2,160-4,560)	40.6 (27.9-59.0)	7.8 (4.15-13.2)	0.1 (0.1-0.2)	319 (176-544)	4.1 (2.3-7.0)
Enterobacter spp.	Fourth-generation cephalosporins	112 (74.5-159)	1.4 (1.0-2.1)	4,730 (3,270-6,720)	61.1 (42.2-86.9)	5.32 (2.66-9.29)	0.1 (0.0-0.1)	238 (119-412)	3.1 (1.5-5.3)
Enterobacter spp.	Trimethoprim-Sulfamethoxazole	87.5 (57.6-125)	1.1 (0.7-1.6)	3,580 (2,470-4,960)	46.3 (31.9-64.1)	4.65 (0-9.8)	0.1 (0.0-0.1)	180 (0-377)	2.3 (0.0-4.9)
Enterobacter spp.	Resistance to one or more antibiotics	185 (122-264)	2.4 (1.6-3.4)	7,070 (4,830-10,000)	91.4 (62.4-129.8)	46.1 (29.6-67.1)	0.6 (0.4-0.9)	1,780 (1,160-2,580)	23.0 (15.1-33.4)
Enterococcus faecalis	Fluoroquinolones	109 (66.6-162)	1.4 (0.9-2.1)	3,780 (2,460-5,450)	48.9 (31.8-70.5)	26.8 (12.4-47.3)	0.3 (0.2-0.6)	930 (455-1,560)	12.0 (5.9-20.2)
Enterococcus faecalis	Vancomycin	12.1 (7.25-19.6)	0.2 (0.1-0.3)	403 (253-611)	5.2 (3.3-7.9)	3.42 (1.46-6.48)	0.0 (0.0-0.1)	111 (48.2-209)	1.4 (0.6-2.7)
Enterococcus faecalis	Resistance to one or more antibiotics	112 (69.2-167)	1.4 (0.9-2.2)	3,880 (2,540-5,610)	50.2 (32.8-72.5)	30.2 (15.5-51.2)	0.4 (0.2-0.7)	1,040 (545-1,700)	13.4 (7.0-22.0)
Enterococcus faecium	Fluoroquinolones	198 (122-300)	2.6 (1.6-3.9)	5,390 (3,290-8,340)	69.6 (42.5-107.8)	37.2 (11-70.7)	0.5 (0.1-0.9)	1,020 (303-1,930)	13.1 (3.9-25.0)
Enterococcus faecium	Vancomycin	59.9 (37.3-92.1)	0.8 (0.5-1.2)	1,610 (990-2,490)	20.8 (12.8-32.2)	14.3 (7.19-24.7)	0.2 (0.1-0.3)	384 (192-667)	5.0 (2.5-8.6)
Enterococcus faecium	Resistance to one or more antibiotics	200 (123-303)	2.6 (1.6-3.9)	5,440 (3,320-8,420)	70.3 (42.9-108.8)	51.5 (27.1-87.7)	0.7 (0.4-1.1)	1,400 (734-2,400)	18.1 (9.5-31.1)
Other enterococci	Fluoroquinolones	65.2 (42.8-96)	0.8 (0.6-1.2)	2,110 (1,380-3,120)	27.2 (17.9-40.3)	12.2 (1.87-24)	0.2 (0.0-0.3)	398 (63.9-782)	5.1 (0.8-10.1)
Other enterococci	Vancomycin	11.8 (7.62-17.1)	0.2 (0.1-0.2)	301 (196-444)	3.9 (2.5-5.7)	2.22 (1.19-3.94)	0.0 (0.0-0.1)	56.5 (30.3-100)	0.7 (0.4-1.3)
Other enterococci	Resistance to one or more antibiotics	67.1 (44-98.4)	0.9 (0.6-1.3)	2,150 (1,410-3,170)	27.7 (18.3-41.0)	14.5 (4.9-26.5)	0.2 (0.1-0.3)	454 (144-841)	5.9 (1.9-10.9)
Escherichia coli	Aminoglycosides	200 (146-266)	2.6 (1.9-3.4)	7,560 (5,700-9,860)	97.7 (73.7-127.4)	11.7 (7.65-16.5)	0.2 (0.1-0.2)	437 (291-612)	5.6 (3.8-7.9)
Escherichia coli	Aminopenicillin	764 (554-1,030)	9.9 (7.2-13.3)	26,000 (19,600-34,200)	336.6 (253.3-442.0)	10.5 (6.94-15.1)	0.1 (0.1-0.2)	298 (201-416)	3.9 (2.6-5.4)
Escherichia coli	Beta Lactam/Beta-lactamase inhibitors	586 (425-788)	7.6 (5.5-10.2)	20,400 (15,400-26,800)	263.2 (198.6-346.1)	21.3 (14.7-30)	0.3 (0.2-0.4)	634 (452-873)	8.2 (5.8-11.3)
Escherichia coli	Carbapenem	140 (102-188)	1.8 (1.3-2.4)	5,270 (3,930-6,980)	68.1 (50.8-90.1)	29.5 (17.1-45)	0.4 (0.2-0.6)	1,090 (640-1,670)	14.1 (8.3-21.5)
Escherichia coli	Fluoroquinolones	532 (384-708)	6.9 (5.0-9.1)	18,400 (13,900-24,100)	238.0 (179.1-311.4)	56 (38.5-78.7)	0.7 (0.5-1.0)	1,890 (1,310-2,580)	24.4 (16.9-33.3)
Escherichia coli	Third-generation cephalosporins	499 (362-673)	6.5 (4.7-8.7)	17,900 (13,400-23,800)	231.2 (173.4-307.2)	59.9 (26.3-109)	0.8 (0.3-1.4)	2,080 (947-3,660)	26.9 (12.2-47.2)
Escherichia coli	Trimethoprim-Sulfamethoxazole	536 (390-713)	6.9 (5.0-9.2)	19,500 (14,700-25,500)	251.8 (190.2-329.9)	30.2 (20.2-42.8)	0.4 (0.3-0.6)	1,080 (718-1,520)	14.0 (9.3-19.6)
Escherichia coli	Resistance to one or more antibiotics	829 (601-1,120)	10.7 (7.8-14.4)	28,000 (21,000-36,900)	362.2 (272.0-476.8)	219 (152-316)	2.8 (2.0-4.1)	7,520 (5,270-10,500)	97.1 (68.2-136.2)
Group A *Streptococcus*	Macrolide	39 (18.3-77.1)	0.5 (0.2-1.0)	1,170 (653-2,090)	15.1 (8.4-27.0)	3.63 (0-13.9)	0.0 (0.0-0.2)	108 (0-376)	1.4 (0.0-4.9)
Group A *Streptococcus*	Resistance to one or more antibiotics	39 (18.3-77.1)	0.5 (0.2-1.0)	1,170 (653-2,090)	15.1 (8.4-27.0)	3.63 (0-13.9)	0.0 (0.0-0.2)	108 (0-376)	1.4 (0.0-4.9)
Group B *Streptococcus*	Fluoroquinolones	67.5 (49-91)	0.9 (0.6-1.2)	4,240 (3,070-5,750)	54.8 (39.6-74.4)	11.5 (1.92-24.9)	0.1 (0.0-0.3)	723 (125-1,580)	9.3 (1.6-20.4)
Group B *Streptococcus*	Macrolide	142 (102-193)	1.8 (1.3-2.5)	7,230 (5,230-9,740)	93.4 (67.6-125.9)	13.5 (0-36.3)	0.2 (0.0-0.5)	672 (0-1,780)	8.7 (0.0-23.0)
Group B *Streptococcus*	Penicillin	3.74 (2.59-5.25)	0.0 (0.0-0.1)	203 (141-281)	2.6 (1.8-3.6)	0.799 (0.165-1.7)	0.0 (0.0-0.0)	42.9 (8.52-91.5)	0.6 (0.1-1.2)
Group B *Streptococcus*	Resistance to one or more antibiotics	173 (125-232)	2.2 (1.6-3.0)	9,190 (6,770-12,300)	118.8 (87.5-158.4)	25.8 (3.92-51.8)	0.3 (0.1-0.7)	1,440 (281-2,780)	18.6 (3.6-36.0)
Haemophilus influenzae	Aminopenicillin	27.4 (21.8-34.2)	0.4 (0.3-0.4)	1,450 (1,120-1,840)	18.7 (14.4-23.8)	4.29 (0.393-8.3)	0.1 (0.0-0.1)	218 (21.5-431)	2.8 (0.3-5.6)
Haemophilus influenzae	Third-generation cephalosporins	8.57 (6.41-11.1)	0.1 (0.1-0.1)	560 (413-738)	7.2 (5.3-9.5)	2.47 (1.14-4.16)	0.0 (0.0-0.1)	161 (71.7-272)	2.1 (0.9-3.5)
Haemophilus influenzae	Resistance to one or more antibiotics	31.5 (25.5-39)	0.4 (0.3-0.5)	1,720 (1,340-2,180)	22.3 (17.4-28.2)	6.76 (2.63-11.3)	0.1 (0.0-0.1)	379 (156-619)	4.9 (2.0-8.0)
Klebsiella pneumoniae	Aminoglycosides	345 (254-463)	4.5 (3.3-6.0)	16,500 (12,400-21,600)	213.5 (160.2-279.4)	26.3 (16.2-38.9)	0.3 (0.2-0.5)	1,230 (773-1,780)	16.0 (10.0-23.0)
Klebsiella pneumoniae	Beta Lactam/Beta-lactamase inhibitors	545 (396-725)	7.0 (5.1-9.4)	24,100 (17,900-31,700)	311.4 (232.0-409.8)	7.93 (4.59-12.1)	0.1 (0.1-0.2)	316 (182-478)	4.1 (2.3-6.2)
Klebsiella pneumoniae	Carbapenem	234 (164-332)	3.0 (2.1-4.3)	9,280 (6,640-12,800)	119.9 (85.9-165.6)	55.7 (36.3-81.3)	0.7 (0.5-1.1)	2,170 (1,430-3,120)	28.0 (18.5-40.3)
Klebsiella pneumoniae	Fluoroquinolones	426 (312-576)	5.5 (4.0-7.4)	18,900 (14,100-24,900)	244.3 (181.8-322.1)	29 (17.4-44.1)	0.4 (0.2-0.6)	1,260 (761-1,880)	16.2 (9.8-24.3)
Klebsiella pneumoniae	Third-generation cephalosporins	526 (384-717)	6.8 (5.0-9.3)	22,900 (17,100-30,100)	296.4 (221.4-388.9)	50.1 (19-94.4)	0.6 (0.2-1.2)	2,210 (836-4,070)	28.6 (10.8-52.6)
Klebsiella pneumoniae	Trimethoprim-Sulfamethoxazole	488 (356-653)	6.3 (4.6-8.4)	21,900 (16,300-28,800)	282.4 (211.3-372.3)	23.5 (11.5-39.7)	0.3 (0.1-0.5)	1,010 (494-1,660)	13.1 (6.4-21.5)
Klebsiella pneumoniae	Resistance to one or more antibiotics	642 (465-863)	8.3 (6.0-11.2)	27,400 (20,300-36,100)	354.5 (261.8-466.0)	193 (130-272)	2.5 (1.7-3.5)	8,200 (5,550-11,400)	106.0 (71.8-147.5)
Morganella spp.	Fluoroquinolones	2.59 (1.64-3.94)	0.0 (0.0-0.1)	56.7 (36.4-85.8)	0.7 (0.5-1.1)	0.427 (0.174-0.78)	0.0 (0.0-0.0)	9.21 (3.76-16.8)	0.1 (0.0-0.2)
Morganella spp.	Fourth-generation cephalosporins	1 (0.621-1.55)	0.0 (0.0-0.0)	23.7 (14.7-36.2)	0.3 (0.2-0.5)	0.154 (0-0.321)	0.0 (0.0-0.0)	3.5 (1.14-7.23)	0.0 (0.0-0.1)
Morganella spp.	Third-generation cephalosporins	1.45 (0.907-2.19)	0.0 (0.0-0.0)	30.2 (19-45.6)	0.4 (0.2-0.6)	0.168 (0-0.342)	0.0 (0.0-0.0)	3.2 (1.28-6.52)	0.0 (0.0-0.1)
Morganella spp.	Resistance to one or more antibiotics	3 (1.93-4.59)	0.0 (0.0-0.1)	64.7 (41.5-98)	0.8 (0.5-1.3)	0.749 (0.394-1.27)	0.0 (0.0-0.0)	15.9 (8.56-27)	0.2 (0.1-0.3)
Mycobacterium tuberculosis	Extensive drug resistance in TB	8.5 (4-15.2)	0.1 (0.1-0.2)	311 (151-553)	4.0 (1.9-7.1)	5.21 (2.48-9.31)	0.1 (0.0-0.1)	181 (88.4-319)	2.3 (1.1-4.1)
Mycobacterium tuberculosis	Isoniazid mono-resistance	73.8 (50.7-103)	1.0 (0.7-1.3)	2,880 (2,040-3,930)	37.2 (26.4-50.7)	11.6 (0-40.2)	0.2 (0.0-0.5)	419 (0-1,410)	5.4 (0.0-18.3)
Mycobacterium tuberculosis	Multi-drug resistance excluding extensive drug resistance in TB	110 (43.6-210)	1.4 (0.6-2.7)	4,210 (1,740-7,990)	54.5 (22.5-103.3)	64.6 (6.6-160)	0.8 (0.1-2.1)	2,320 (225-5,660)	30.0 (2.9-73.2)
Mycobacterium tuberculosis	Rifampicin mono-resistance	12 (8.85-15.6)	0.2 (0.1-0.2)	494 (368-644)	6.4 (4.8-8.3)	3.35 (0.569-6.54)	0.0 (0.0-0.1)	130 (26.6-249)	1.7 (0.3-3.2)
Mycobacterium tuberculosis	Resistance to one or more antibiotics	204 (134-303)	2.6 (1.7-3.9)	7,900 (5,260-11,600)	102.0 (68.0-149.9)	84.8 (18.3-182)	1.1 (0.2-2.4)	3,050 (693-6,550)	39.4 (9.0-84.7)
Neisseria gonorrhoeae	Fluoroquinolones	--	--	53.7 (32.6-84.1)	0.7 (0.4-1.1)	--	--	5.14 (1.46-10.3)	0.1 (0.0-0.1)
Neisseria gonorrhoeae	Third-generation cephalosporins	--	--	1.95 (1-3.49)	0.0 (0.0-0.0)	--	--	0.374 (0.119-0.797)	0.0 (0.0-0.0)
Neisseria gonorrhoeae	Resistance to one or more antibiotics	--	--	53.9 (32.7-84.4)	0.7 (0.4-1.1)	--	--	5.51 (1.77-11)	0.1 (0.0-0.1)
Proteus spp.	Aminoglycosides	19.4 (12.6-28.1)	0.3 (0.2-0.4)	533 (345-774)	6.9 (4.5-10.0)	0.887 (0-1.91)	0.0 (0.0-0.0)	24.1 (2.79-51.4)	0.3 (0.0-0.7)
Proteus spp.	Aminopenicillin	70 (46.2-101)	0.9 (0.6-1.3)	1,750 (1,140-2,540)	22.6 (14.7-32.9)	1.33 (0-3.96)	0.0 (0.0-0.1)	32.4 (0-95.9)	0.4 (0.0-1.2)
Proteus spp.	Fluoroquinolones	40.7 (26.7-59)	0.5 (0.3-0.8)	1,040 (680-1,530)	13.5 (8.8-19.7)	2.97 (1.24-5.64)	0.0 (0.0-0.1)	74.7 (30.7-142)	1.0 (0.4-1.8)
Proteus spp.	Third-generation cephalosporins	23.4 (15-34.2)	0.3 (0.2-0.4)	582 (365-859)	7.5 (4.7-11.1)	4.73 (1.56-9.45)	0.1 (0.0-0.1)	116 (37.6-233)	1.5 (0.5-3.0)
Proteus spp.	Trimethoprim-Sulfamethoxazole	45.6 (30.3-66.2)	0.6 (0.4-0.9)	1,190 (780-1,750)	15.4 (10.1-22.6)	1.62 (0-3.57)	0.0 (0.0-0.0)	41.9 (0-90.6)	0.5 (0.0-1.2)
Proteus spp.	Resistance to one or more antibiotics	80 (52.7-115)	1.0 (0.7-1.5)	2,000 (1,310-2,900)	25.8 (16.9-37.5)	11.5 (6.33-18.6)	0.1 (0.1-0.2)	290 (159-466)	3.7 (2.1-6.0)
Pseudomonas aeruginosa	Aminoglycosides	115 (81.3-160)	1.5 (1.1-2.1)	4,530 (3,290-6,080)	58.5 (42.6-78.6)	3 (0-7.61)	0.0 (0.0-0.1)	120 (0-294)	1.6 (0.0-3.8)
Pseudomonas aeruginosa	Anti-pseudomonal penicillin/Beta-Lactamase inhibitors	156 (109-216)	2.0 (1.4-2.8)	5,540 (3,960-7,520)	71.7 (51.2-97.2)	10.3 (6.47-16)	0.1 (0.1-0.2)	378 (239-587)	4.9 (3.1-7.6)
Pseudomonas aeruginosa	Carbapenem	210 (146-292)	2.7 (1.9-3.8)	7,250 (5,140-9,840)	93.6 (66.4-127.1)	38.1 (24.3-57.2)	0.5 (0.3-0.7)	1,310 (848-1,940)	16.9 (11.0-25.0)
Pseudomonas aeruginosa	Fluoroquinolones	172 (120-236)	2.2 (1.6-3.1)	6,130 (4,380-8,210)	79.2 (56.6-106.1)	18.3 (11.7-27.8)	0.2 (0.2-0.4)	652 (422-967)	8.4 (5.5-12.5)
Pseudomonas aeruginosa	Fourth-generation cephalosporins	169 (118-231)	2.2 (1.5-3.0)	6,260 (4,550-8,420)	80.9 (58.8-108.9)	4.37 (2.62-6.9)	0.1 (0.0-0.1)	178 (108-279)	2.3 (1.4-3.6)
Pseudomonas aeruginosa	Third-generation cephalosporins	213 (146-297)	2.7 (1.9-3.8)	7,970 (5,660-10,900)	103.0 (73.2-141.0)	10.4 (4.61-18)	0.1 (0.1-0.2)	407 (186-689)	5.3 (2.4-8.9)
Pseudomonas aeruginosa	Resistance to one or more antibiotics	334 (234-457)	4.3 (3.0-5.9)	12,000 (8,630-16,100)	155.0 (111.5-208.5)	84.6 (53-127)	1.1 (0.7-1.6)	3,050 (1,980-4,530)	39.4 (25.5-58.5)
Salmonella Paratyphi	Fluoroquinolones	20.1 (8.5-38.9)	0.3 (0.1-0.5)	1,410 (584-2,750)	18.2 (7.5-35.5)	4 (0.622-10.2)	0.1 (0.0-0.1)	283 (42-711)	3.7 (0.5-9.2)
Salmonella Paratyphi	Multi-drug resistance in Salmonella Typhi and Paratyphi	0.779 (0.322-1.54)	0.0 (0.0-0.0)	55 (22.6-110)	0.7 (0.3-1.4)	0.066 (0-0.197)	0.0 (0.0-0.0)	4.53 (0-13.3)	0.1 (0.0-0.2)

Salmonella Paratyphi	Resistance to one or more antibiotics	20.2 (8.54-39.1)	0.3 (0.1-0.5)	1,420 (588-2,760)	18.3 (7.6-35.7)	4.11 (0.677-10.3)	0.1 (0.0-0.1)	287 (47.2-718)	3.7 (0.6-9.3)
Salmonella Typhi	Fluoroquinolones	90.6 (54.1-143)	1.2 (0.7-1.9)	6,570 (3,880-10,600)	84.9 (50.2-136.7)	17.2 (2.96-39.1)	0.2 (0.0-0.5)	1,240 (213-2,810)	16.0 (2.8-36.3)
Salmonella Typhi	Multi-drug resistance in Salmonella Typhi and Paratyphi	58.3 (38.2-84.2)	0.8 (0.5-1.1)	4,400 (2,860-6,410)	56.9 (37.0-82.8)	6.46 (0-16.5)	0.1 (0.0-0.2)	488 (0-1,240)	6.3 (0.0-16.0)
Salmonella Typhi	Resistance to one or more antibiotics	127 (80.9-193)	1.6 (1.0-2.5)	9,350 (5,890-14,200)	120.8 (76.1-183.9)	23.7 (7.55-47.2)	0.3 (0.1-0.6)	1,720 (536-3,400)	22.3 (6.9-43.9)
Non-typhoidal *Salmonella*	Fluoroquinolones	27.1 (14-46.8)	0.4 (0.2-0.6)	1,390 (717-2,450)	18.0 (9.3-31.7)	5.62 (0.809-13.1)	0.1 (0.0-0.2)	264 (46.3-597)	3.4 (0.6-7.7)
Non-typhoidal *Salmonella*	Resistance to one or more antibiotics	27.1 (14-46.8)	0.4 (0.2-0.6)	1,390 (717-2,450)	18.0 (9.3-31.7)	5.62 (0.809-13.1)	0.1 (0.0-0.2)	264 (46.3-597)	3.4 (0.6-7.7)
Serratia spp.	Aminoglycosides	16.5 (10.4-25)	0.2 (0.1-0.3)	830 (531-1,260)	10.7 (6.9-16.2)	0.953 (0-2.52)	0.0 (0.0-0.0)	48.1 (0-128)	0.6 (0.0-1.6)
Serratia spp.	Anti-pseudomonal penicillin/Beta-Lactamase inhibitors	13.7 (8.41-21.3)	0.2 (0.1-0.3)	567 (357-871)	7.3 (4.6-11.3)	2.48 (1.21-4.34)	0.0 (0.0-0.1)	103 (51.3-181)	1.3 (0.7-2.3)
Serratia spp.	Carbapenem	8.99 (5.35-14.3)	0.1 (0.1-0.2)	342 (211-524)	4.4 (2.7-6.8)	2.45 (1.23-4.35)	0.0 (0.0-0.1)	93.1 (47.9-164)	1.2 (0.6-2.1)
Serratia spp.	Fluoroquinolones	10.4 (6.55-16.1)	0.1 (0.1-0.2)	477 (308-736)	6.2 (4.0-9.5)	1 (0.224-2.31)	0.0 (0.0-0.0)	49.1 (9.48-109)	0.6 (0.1-1.4)
Serratia spp.	Fourth-generation cephalosporins	23.8 (14.9-36.6)	0.3 (0.2-0.5)	1,180 (764-1,820)	15.3 (9.9-23.5)	2.61 (1.12-4.9)	0.0 (0.0-0.1)	140 (59.8-254)	1.8 (0.8-3.3)
Serratia spp.	Third-generation cephalosporins	25.1 (16-38.1)	0.3 (0.2-0.5)	1,150 (736-1,710)	14.8 (9.5-22.1)	1.1 (0.403-2.13)	0.0 (0.0-0.0)	44.6 (16.9-85)	0.6 (0.2-1.1)
Serratia spp.	Resistance to one or more antibiotics	42.7 (27-65.3)	0.6 (0.3-0.8)	1,930 (1,260-2,900)	24.9 (16.3-37.5)	10.7 (6.11-17.5)	0.1 (0.1-0.2)	478 (274-760)	6.2 (3.5-9.8)
Shigella spp.	Fluoroquinolones	29.1 (11.8-57.7)	0.4 (0.2-0.7)	1,650 (705-3,070)	21.3 (9.1-39.7)	5.99 (0.689-15.4)	0.1 (0.0-0.2)	330 (48-803)	4.3 (0.6-10.4)
Shigella spp.	Resistance to one or more antibiotics	29.1 (11.8-57.7)	0.4 (0.2-0.7)	1,650 (705-3,070)	21.3 (9.1-39.7)	5.99 (0.689-15.4)	0.1 (0.0-0.2)	330 (48-803)	4.3 (0.6-10.4)
Staphylococcus aureus	Fluoroquinolones	366 (266-494)	4.7 (3.4-6.4)	11,800 (8,680-15,800)	152.8 (112.2-203.6)	15.9 (7-27.5)	0.2 (0.1-0.4)	501 (226-853)	6.5 (2.9-11.0)
Staphylococcus aureus	Macrolide	522 (380-704)	6.7 (4.9-9.1)	16,600 (12,300-22,000)	214.5 (158.9-284.3)	19.6 (7.78-35)	0.3 (0.1-0.5)	603 (245-1,050)	7.8 (3.2-13.5)
Staphylococcus aureus	Methicillin	473 (344-642)	6.1 (4.5-8.3)	15,200 (11,100-20,500)	195.9 (143.8-264.6)	121 (53.2-207)	1.6 (0.7-2.7)	3,790 (1,650-6,390)	48.9 (21.3-82.5)
Staphylococcus aureus	Trimethoprim-Sulfamethoxazole	201 (154-262)	2.6 (2.0-3.4)	9,310 (7,190-11,900)	120.3 (92.9-154.1)	18.7 (9.75-28.7)	0.2 (0.1-0.4)	878 (463-1,340)	11.3 (6.0-17.3)
Staphylococcus aureus	Vancomycin	10.3 (7.35-14.6)	0.1 (0.1-0.2)	358 (258-500)	4.6 (3.3-6.5)	3.12 (1.61-5.65)	0.0 (0.0-0.1)	103 (53.9-181)	1.3 (0.7-2.3)
Staphylococcus aureus	Resistance to one or more antibiotics	748 (554-1,000)	9.7 (7.2-13.0)	24,900 (18,600-32,700)	321.3 (240.4-423.2)	178 (104-280)	2.3 (1.3-3.6)	5,870 (3,550-9,220)	75.9 (45.9-119.2)
Streptococcus pneumoniae	Beta Lactam/Beta-lactamase inhibitors	107 (82-138)	1.4 (1.1-1.8)	5,430 (4,150-6,960)	70.2 (53.6-89.9)	2.65 (0.054-6.62)	0.0 (0.0-0.1)	114 (1.44-284)	1.5 (0.0-3.7)
Streptococcus pneumoniae	Carbapenem	191 (148-242)	2.5 (1.9-3.1)	9,100 (7,070-11,500)	117.6 (91.3-148.7)	41.9 (20.5-70)	0.5 (0.3-0.9)	1,990 (957-3,310)	25.7 (12.4-42.8)
Streptococcus pneumoniae	Fluoroquinolones	89.8 (70.8-113)	1.2 (0.9-1.5)	5,240 (4,100-6,680)	67.7 (53.0-86.3)	11.2 (2.64-22.3)	0.1 (0.0-0.3)	643 (153-1,280)	8.3 (2.0-16.5)
Streptococcus pneumoniae	Macrolide	313 (252-391)	4.0 (3.3-5.1)	13,300 (10,700-16,700)	172.2 (138.6-216.0)	12.5 (0-32.6)	0.2 (0.0-0.4)	517 (0-1,330)	6.7 (0.0-17.2)
Streptococcus pneumoniae	Penicillin	230 (184-290)	3.0 (2.4-3.8)	11,200 (8,910-14,000)	144.4 (115.1-181.2)	12.4 (6.69-20.3)	0.2 (0.1-0.3)	597 (327-980)	7.7 (4.2-12.7)
Streptococcus pneumoniae	Third-generation cephalosporins	94.2 (72.5-123)	1.2 (0.9-1.6)	4,720 (3,690-6,140)	61.0 (47.6-79.3)	3.33 (1.39-6.21)	0.0 (0.0-0.1)	177 (73.2-336)	2.3 (0.9-4.3)
Streptococcus pneumoniae	Trimethoprim-Sulfamethoxazole	446 (364-548)	5.8 (4.7-7.1)	23,600 (19,100-29,300)	305.3 (247.3-379.2)	38.7 (5.73-77.2)	0.5 (0.1-1.0)	2,070 (298-4,160)	26.8 (3.9-53.8)
Streptococcus pneumoniae	Resistance to one or more antibiotics	596 (490-727)	7.7 (6.3-9.4)	29,800 (24,400-36,700)	385.8 (314.7-473.8)	122 (82.4-166)	1.6 (1.1-2.1)	6,110 (4,050-8,330)	78.9 (52.4-107.7)

Figure S1 Flowchart of antimicrobial resistance fatal and non-fatal estimation steps

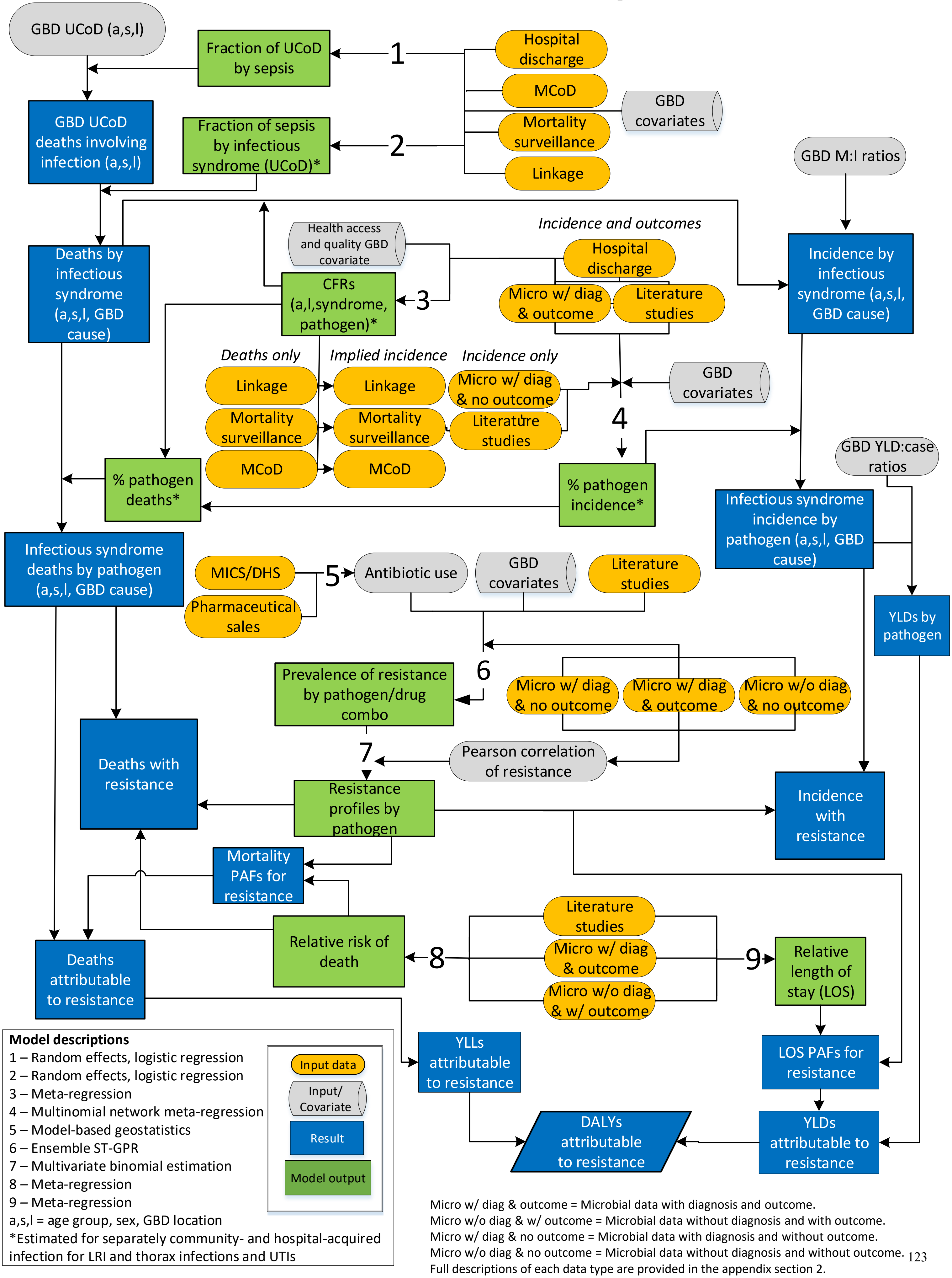

Micro w/ diag & outcome = Microbial data with diagnosis and outcome.
Micro w/o diag & w/ outcome = Microbial data without diagnosis and with outcome.
Micro w/ diag & no outcome = Microbial data with diagnosis and without outcome.
Micro w/o diag & no outcome = Microbial data without diagnosis and without outcome.
Full descriptions of each data type are provided in the appendix section 2.

Figure S2 All-age rate of DALYs attributable to and associated with bacterial antimicrobial resistance by GBD region, 2019

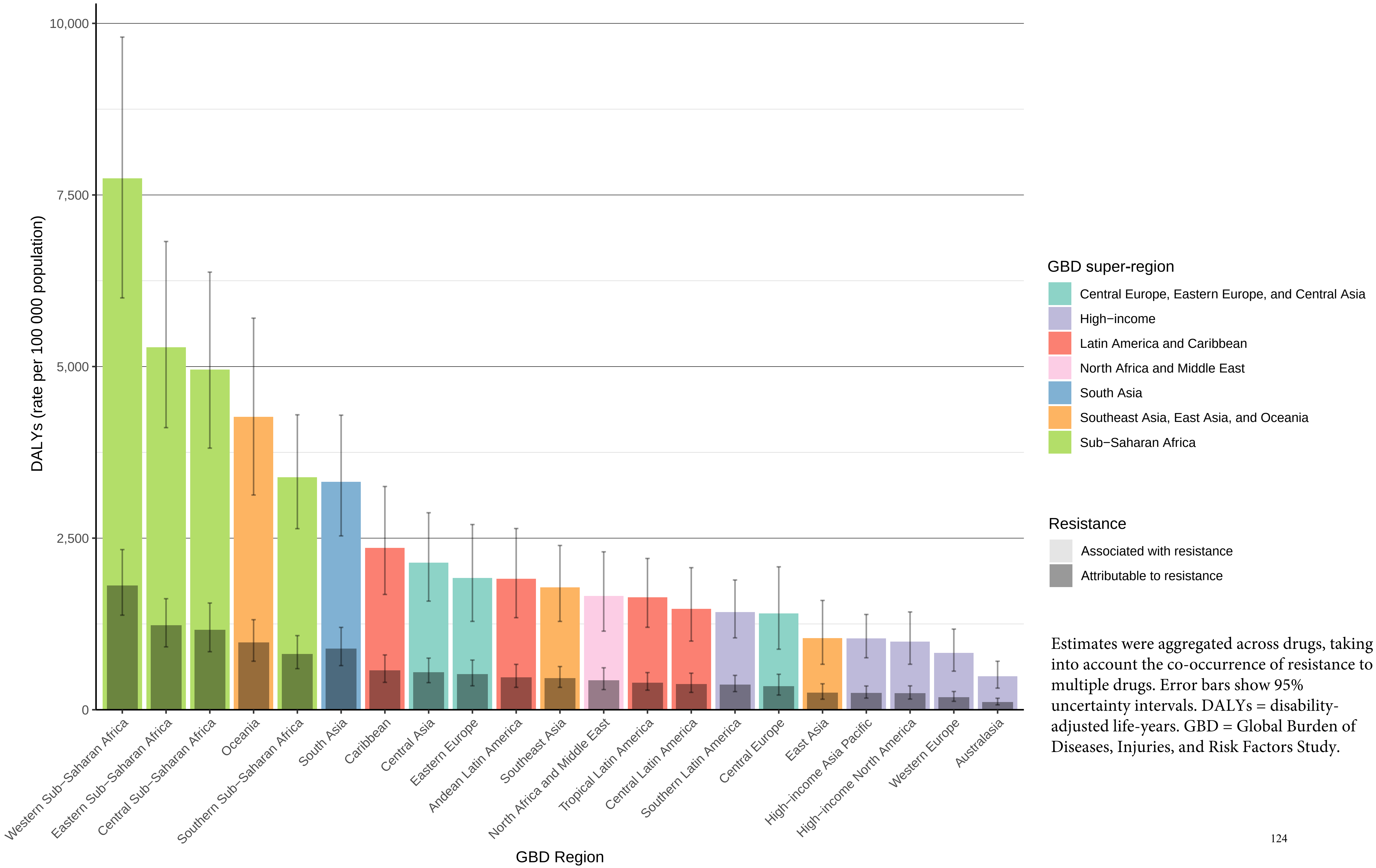

Estimates were aggregated across drugs, taking into account the co-occurrence of resistance to multiple drugs. Error bars show 95% uncertainty intervals. DALYs = disability-adjusted life-years. GBD = Global Burden of Diseases, Injuries, and Risk Factors Study.

Figure S3 Global DALYs (in counts) attributable to and associated with bacterial antimicrobial resistance by infectious syndrome, 2019

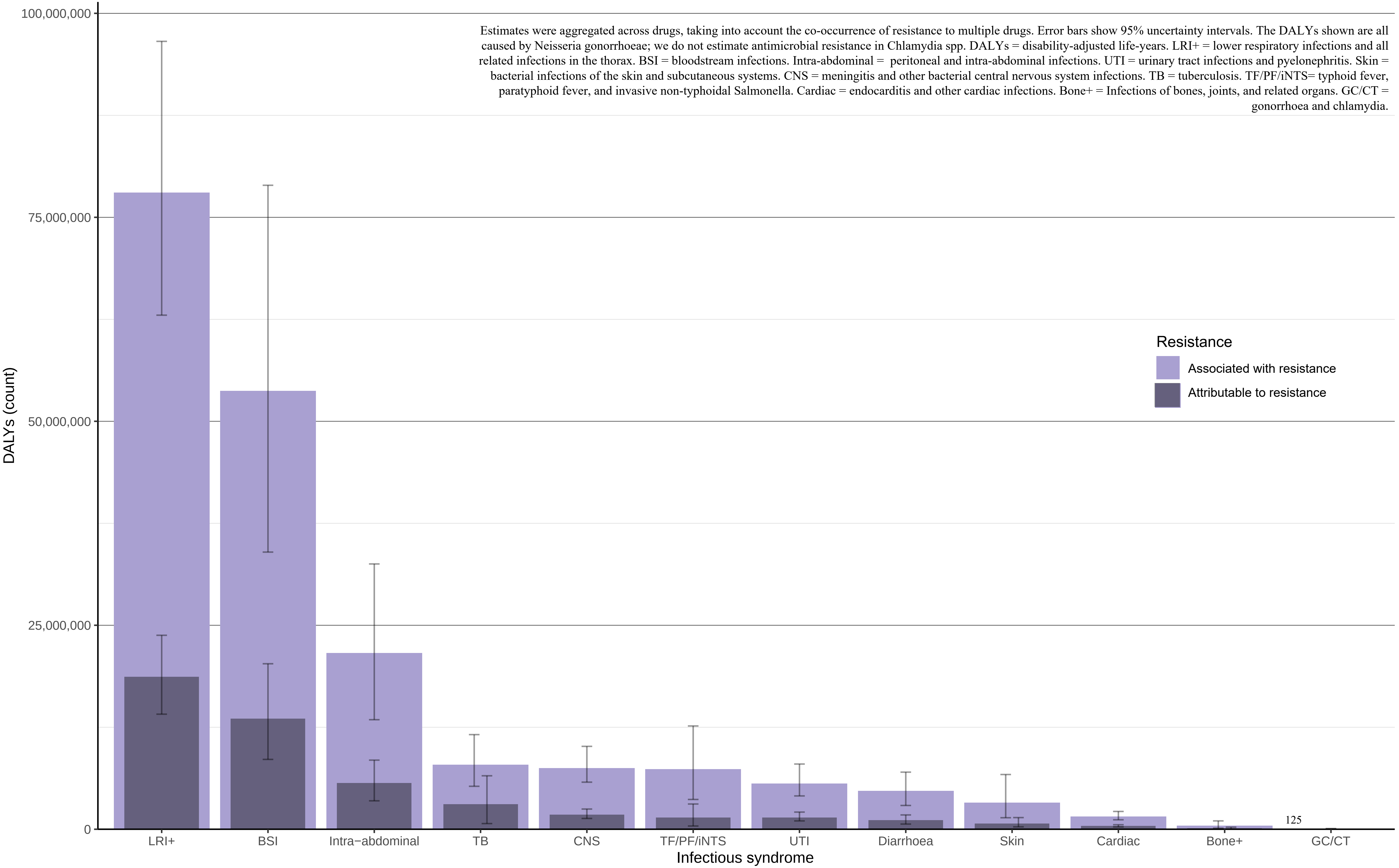

Figure S4 Global DALYs (in counts) attributable to and associated with bacterial antimicrobial resistance by pathogen, 2019

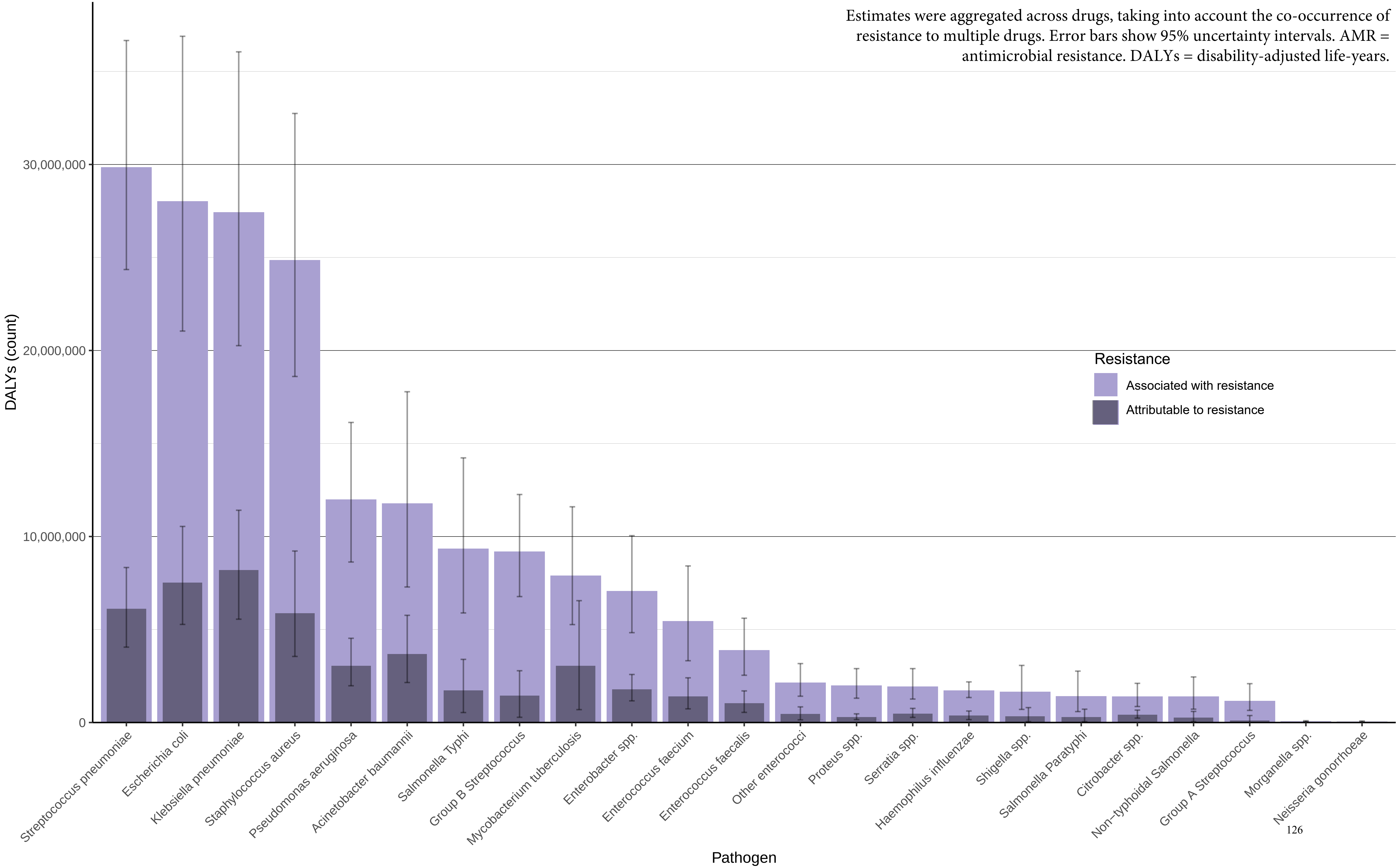

Figure S5A Pathogen−attributable fraction of DALYs attributable to bacterial antimicrobial resistance for the six leading pathogens by GBD super-region, 2019

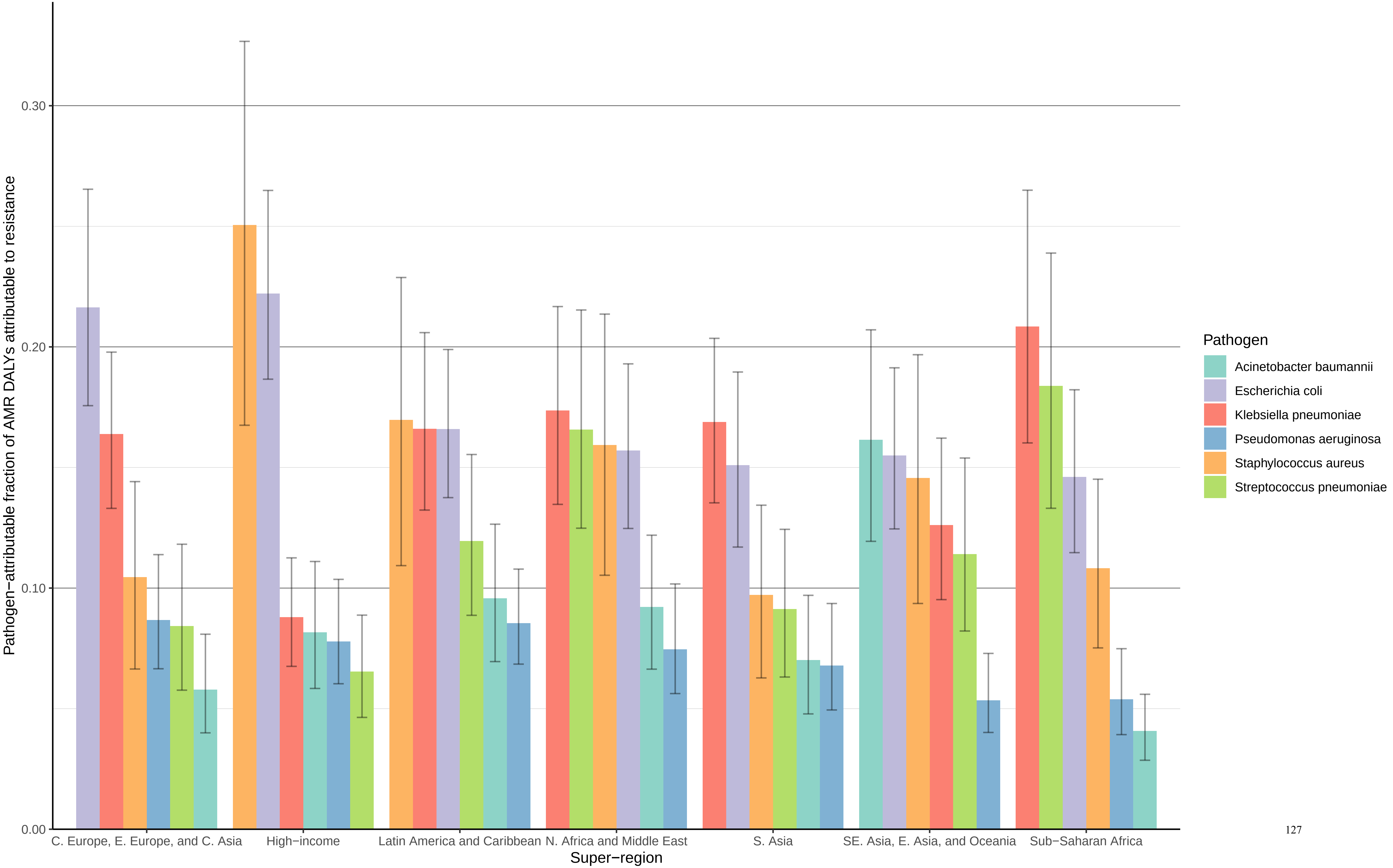

Figure S5B Pathogen−attributable fraction of DALYs associated with bacterial antimicrobial resistance for the six leading pathogens by GBD super-region, 2019

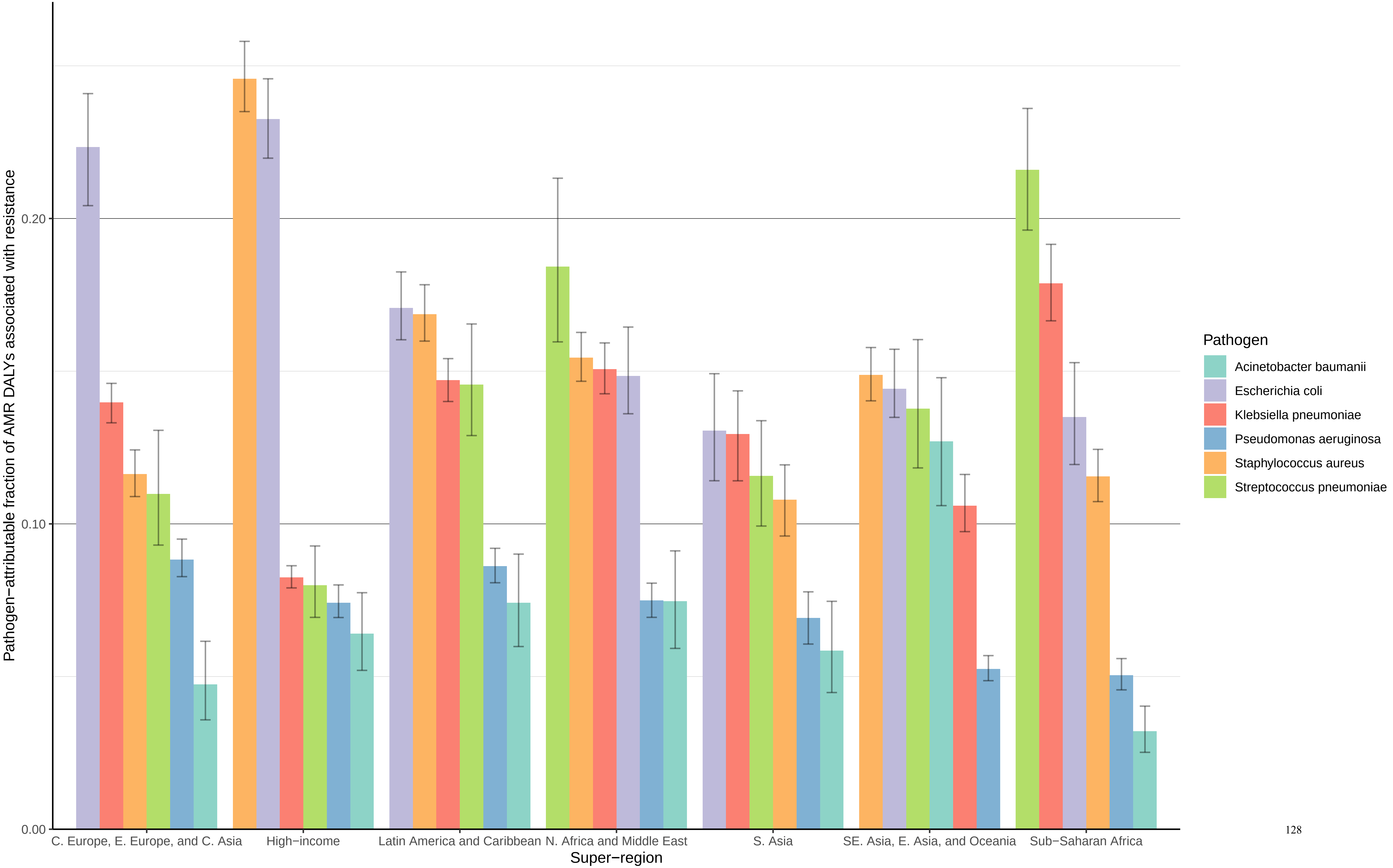

Figure S6 Global DALYs (in counts) attributable to bacterial antimicrobial resistance by pathogen−drug combination, 2019

	Resistance to 1+	3GC	4GC	AG	Aminopenicillin	Anti−pseudomonal	BL−BLI	CP	FQ	Macrolide	MDR excluding XDR in TB	MDR in S. Typhi and Paratyphi	Methicillin	Mono INH	Mono RIF	PCN	TMP−SMX	Vanco	XDR in TB
Acinetobacter baumannii	3,670,000	163,000	79,100	296,000		463,000	17,300	1,540,000	1,120,000										
Citrobacter spp.	416,000	63,400	60,700	18,600		83,900		87,500	102,000										
Enterobacter spp.	1,780,000		238,000	135,000		337,000		568,000	319,000								180,000		
Enterococcus faecalis	1,040,000								930,000									111,000	
Enterococcus faecium	1,400,000								1,020,000									384,000	
Other enterococci	454,000								398,000									56,500	
Escherichia coli	7,520,000	2,080,000		437,000	298,000		634,000	1,090,000	1,890,000								1,080,000		
Group A Streptococcus	108,000									108,000									
Group B Streptococcus	1,440,000								723,000	672,000						42,900			
Haemophilus influenzae	379,000	161,000			218,000														
Klebsiella pneumoniae	8,200,000	2,210,000		1,230,000			316,000	2,170,000	1,260,000								1,010,000		
Morganella spp.	15,900	3,200	3,500						9,210										
Mycobacterium tuberculosis	3,050,000										2,320,000			419,000	130,000				181,000
Neisseria gonorrhoeae	5,510	374							5,140										
Proteus spp.	290,000	116,000		24,100	32,400				74,700								41,900		
Pseudomonas aeruginosa	3,050,000	407,000	178,000	120,000		378,000		1,310,000	652,000										
Salmonella Paratyphi	287,000								283,000			4,530							
Salmonella Typhi	1,720,000								1,240,000			488,000							
Non−typhoidal Salmonella	264,000								264,000										
Serratia spp.	478,000	44,600	140,000	48,100		103,000		93,100	49,100										
Shigella spp.	330,000								330,000										
Staphylococcus aureus	5,870,000								501,000	603,000			3,790,000				878,000	103,000	
Streptococcus pneumoniae	6,110,000	177,000					114,000	1,990,000	643,000	517,000						597,000	2,070,000		
All pathogens	47,900,000	5,430,000	699,000	2,310,000	549,000	1,370,000	1,080,000	8,840,000	11,800,000	1,900,000	2,320,000	492,000	3,790,000	419,000	130,000	640,000	5,270,000	655,000	181,000

Counts
- ≥ 2M
- 1.5M− <2M
- 1M − <1.5M
- 500k − <1M
- 100k − <500k
- <100k
- NA

GBD = Global Burden of Diseases, Injuries, and Risk Factors Study. Error bars show 95% uncertainty intervals.

Figure S7 Vital Registration and Verbal Autopsy data availability by country and territory, 1980−2018

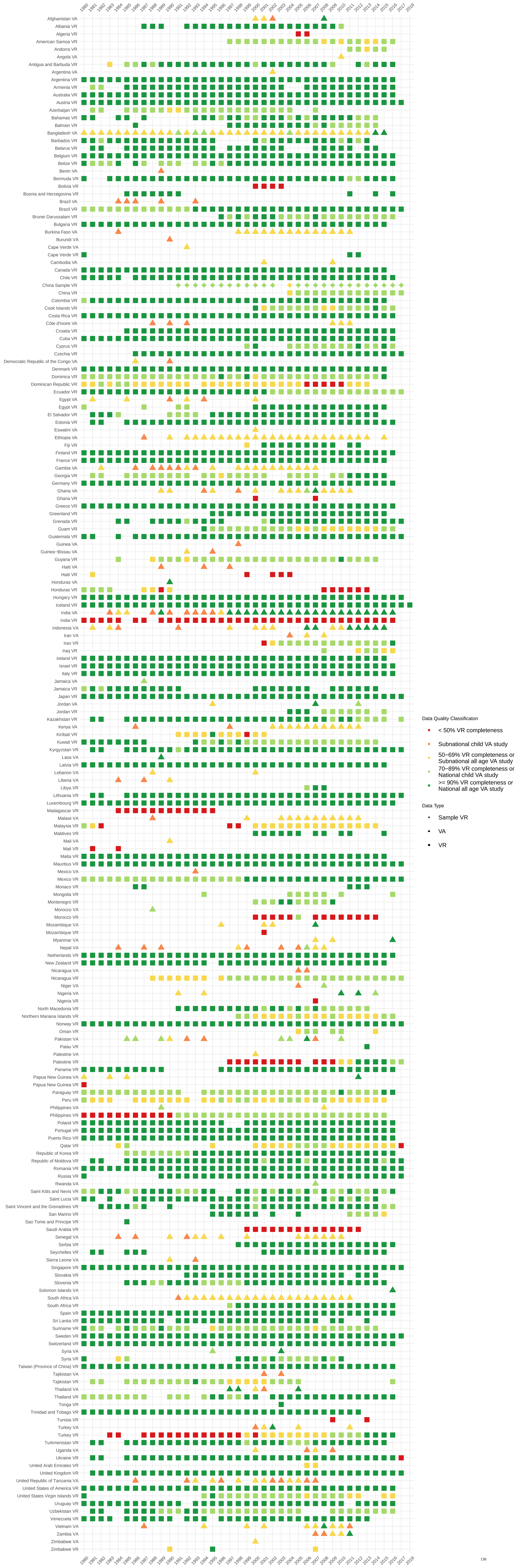

Supplement: Supplemental Material [file NIHMS1778072-supplement-Supplemental_Material.pdf]
